# Coordination sphere interactions drive isomer selection in heteroleptic Pd(ii) cages with low-symmetry ligands

**DOI:** 10.1039/d5sc04881h

**Published:** 2025-09-08

**Authors:** Paulina Molinska, Louise Male, James E. M. Lewis

**Affiliations:** a School of Chemistry, University of Birmingham Molecular Sciences Building, Edgbaston Birmingham B15 2TT UK j.e.m.lewis@bham.ac.uk

## Abstract

The targeted formation of low-symmetry coordination cages represents a significant design challenge but offers the potential to engineer bespoke molecular hosts with precision. In this work, we have combined the design principles of geometric complementarity and coordination sphere engineering to direct the site- and orientation-selective self-assembly of heteroleptic Pd_2_L^A^_2_L^B^_2_-type coordination cages from low-symmetry ligands. The effects of different combinations of heterocyclic donors and their locations within the cage structures on isomer distributions were studied, providing insights on shifts in the balance between non-covalent interactions in the first and second coordination spheres of the cages. For cages with one low-symmetry ligand, switching between selective formation of *syn*- (up to 77%) or *anti*-isomers (up to 76%) was achieved simply through minor structural changes (swapping a hydrogen atom for a fluorine) or changing the location of heterocycles within the cage structure between the different ligand scaffolds. Furthermore, the selective (up to ∼62%) assembly of particular isomers of heteroleptic cages formed from two low-symmetry ligand scaffolds was demonstrated and rationalised.

## Abstract 2

The isomer selective formation of heteroleptic Pd_2_L^A^_2_L^B^_2_-type coordination cages assembled from low-symmetry ligands was demonstrated through tuning of coordination sphere interactions.

## Graphical abstract

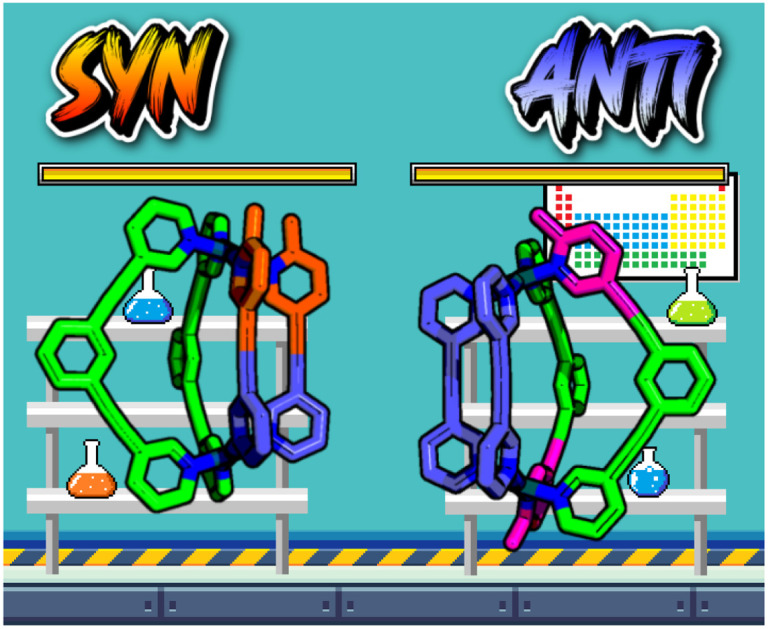

## Introduction

Coordination cages are discrete, three-dimensional, metal–organic assemblies with appreciable internal cavities capable of binding guest species.^1^ Host–guest chemistry within these confined spaces has been exploited for binding anions,^2^ pollutants,^3^ drugs^4^ and gases,^5^ and for use in catalysis,^6^ stabilisation of reactive species^7^ and molecular separations.^8^

**Fig. 1:**
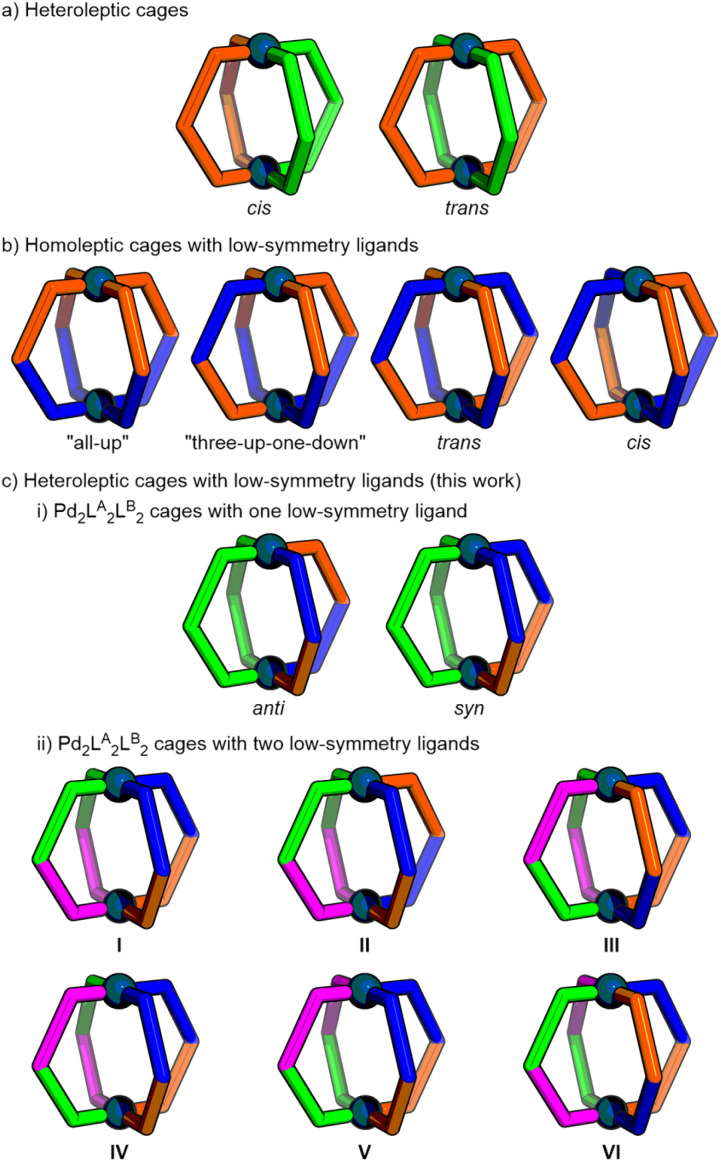
Approaches to reduced symmetry coordination cages include (a) heteroleptic cages with combinations of ligands, (b) homoleptic cages assembled from low-symmetry ligands, and (c) heteroleptic cages assembled from (i) one or (ii) two low-symmetry ligands in which isomer selectivity can be tuned through structural design (this work).

Most commonly, coordination cages are assembled from single, high-symmetry ligands, generally resulting in highly symmetrical architectures. More structurally sophisticated, low-symmetry cages, however, have the potential to exhibit bespoke properties and behaviours.^9^ As such, there has been interest in developing strategies for the site-selective assembly of heteroleptic (mixed-ligand) cages^10^ (Fig. 1a), and the orientation-selective assembly of cages from low-symmetry ligands^11^ (Fig. 1b). In both instances, without sufficient driving force, statistical mixtures of isomers (and other assemblies) can form. Very recently, solutions to the challenge of incorporating low-symmetry ligands into heteroleptic cages (Fig. 1c) have also begun to be investigated.^12^

Geometric complementarity between ligands has been used to drive the formation of heteroleptic structures,^13^ and similar design ideas have enabled the orientation-selective assembly of low-symmetry ligands.^14^ Coordination sphere engineering – using non-covalent interactions, such as steric bulk or hydrogen bonding (HB), to direct the coordination environment around metal ions^15^ – is another strategy that has been successfully employed within both heteroleptic^16^ and low-symmetry ligand systems.^17^

We recently investigated the self-assembly of homoleptic Pd_*n*_L_2*n*_ assemblies from low-symmetry ligands that incorporated either quinoline or picoline donors in combination with unsubstituted pyridines.^18^ Molecular modelling demonstrated that arranging the bulky quinoline/picoline donors *trans* to each other would give the lowest energy assemblies. Although experimentally this held true for ligands with picoline donors, quinolines unexpectedly favoured formation of *cis*-Pd_*n*_L_2*n*_ species. This difference in isomer selectivity was shown to be due to HB interactions between acidic protons of the coordinating donor units and solvent molecules. Such intermolecular non-covalent interactions within the second coordination spheres of the Pd(ii) ions of the cages could therefore override primary structural factors (*i.e.* steric bulk) in dictating self-assembly outcomes.

**Fig. 2:**
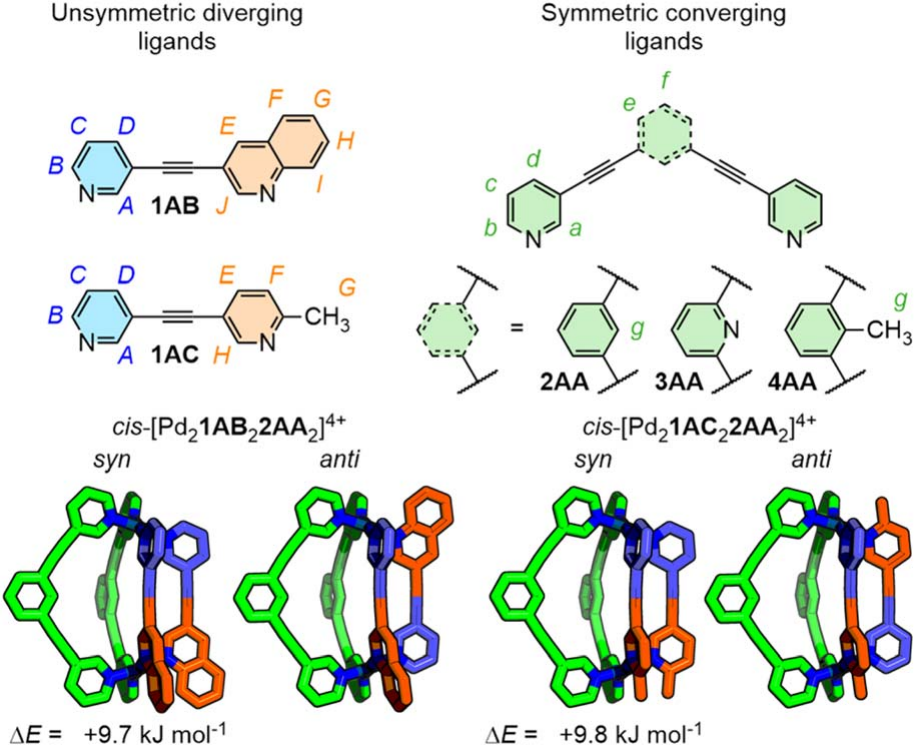
Self-assembly of low-symmetry ligands 1 and ligands 2/3/4 with Pd(ii) ions forms *syn*- and *anti*-isomers, the latter of which are calculated (GFN2-xTB) to be lower in energy.

Within this previous work we reported a preliminary investigation of the heteroleptic cage Pd_2_1AB_2_2AA_2_ (see below) which, due to the unsymmetrical structure of ligand 1AB, could exist as *syn*- and *anti*-isomers (Fig. 2). Chemical intuition, combined with molecular modelling, suggested the *anti*-isomer, with bulky quinoline groups situated far apart, would be lower in energy. The experimentally observed predominant formation of the more sterically encumbered *syn*-isomer, however, again suggested stabilising interactions in the second coordination sphere of the cage were superseding repulsive steric interactions in the first coordination sphere.

Stemming from this initial result, we were motivated to investigate a wider range of heteroleptic structures to observe the impact on isomer selectivity. In this regard, we wished to explore how isomer selectivity was influenced by (i) the identity of the symmetrical ligand; (ii) the combination of different heterocyclic donors on the unsymmetrical ligand, and (iii) the relative locations of the different donors within the cages. Finally, we also sought to achieve (iv) the selective formation of particular isomers of Pd_2_L^A^_2_L^B^_2_ cages assembled from two low-symmetry ligands. Gaining insights into how the, often subtle, balance of interactions that drive self-assembly outcomes can be shifted through design of structure and function will aid in the future development of structurally sophisticated metal–organic assemblies.

## Results and discussion

### System design and nomenclature

In this work we explored ligand scaffolds 1 and 2 (Fig. 2) that have previously been shown by Severin to be geometrically matched and able to undergo integrative self-assembly with Pd(ii) ions to form *cis*-[Pd_2_1_2_2_2_]^4+^ heteroleptic cages.^19^ Ligands 3 and 4 are isostructural to 2 except that the core benzene unit is replaced with a pyridine and toluene, respectively. Each ligand has two N-heterocyclic donors. A two letter combination is used within each ligand name to signify which heterocycles are incorporated into the ligand: pyridine (A), quinoline (B), 2-picoline (C), 2-fluoropyridine (D), and 8-fluoroquinoline (E). Ligand 2AA, for example, has a 1,3-diethynylbenzene core (2) and two pyridyl donors (A).

All ligands used in this work were synthesised using standard techniques and characterised by NMR spectroscopy and high resolution mass spectrometry (HR-MS). Details can be found in the SI.

All the cages in this work are tetracationic and prepared as the BF_4_^−^ salts. For clarity, however, the charge and counterions are generally omitted from the main text. For example, [Pd_2_1AA_2_2AA_2_](BF_4_)_4_ may be written as Pd_2_1AA_2_2AA_2_. Formation of the heteroleptic cages was confirmed by NMR spectroscopy, ^1^H DOSY and electrospray ionisation (ESI) MS. Details can be found in the SI.

### Quinoline donor on diverging ligand

**Fig. 3:**
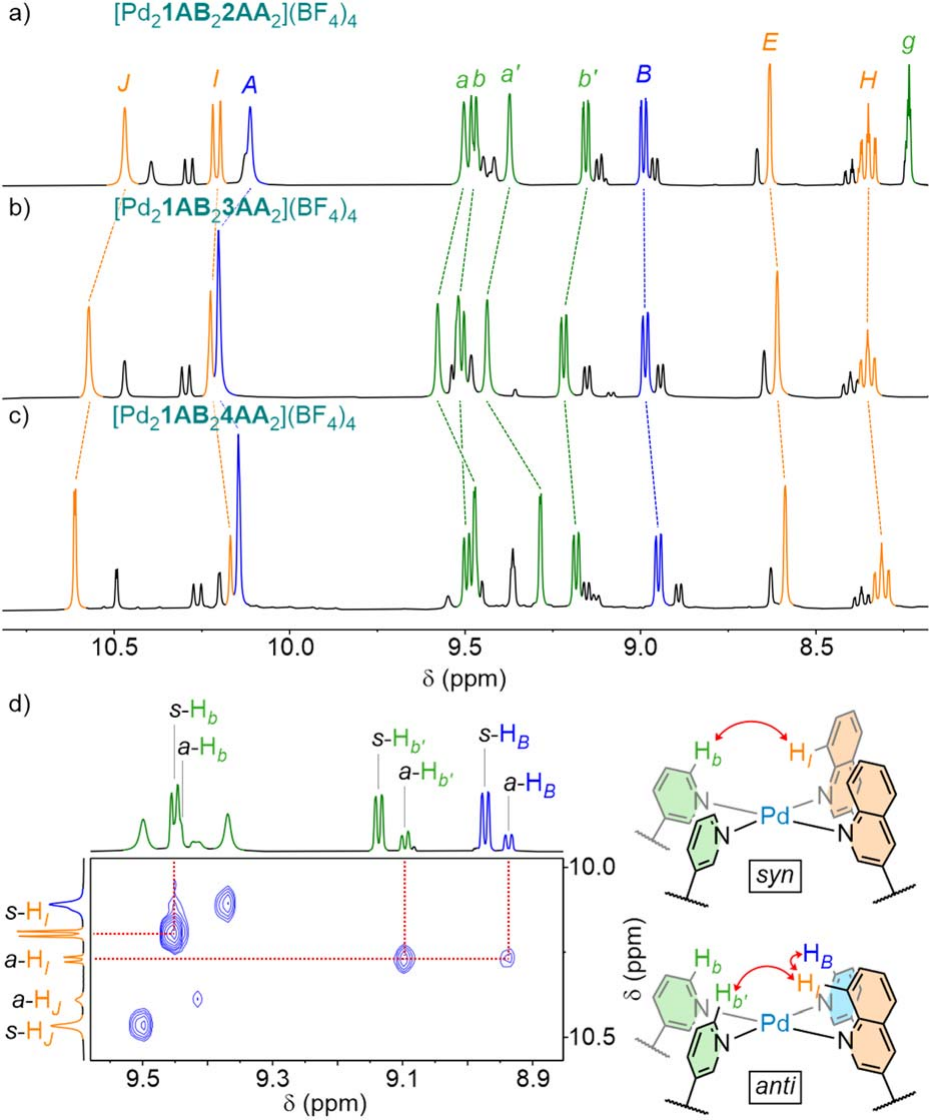
Partial ^1^H NMR spectra (400 MHz, CD_3_CN, 298 K), with major *syn*-isomer peaks labelled, of (a) [Pd_2_1AB_2_2AA_2_](BF_4_)_4_, (b) [Pd_2_1AB_2_3AA_2_](BF_4_)_4_, and (c) [Pd_2_1AB_2_4AA_2_](BF_4_)_4_. (d) Partial NOESY spectrum (600 MHz, CD_3_CN, 298 K) of [Pd_2_1AB_2_2AA_2_](BF_4_)_4_ with key peaks used to identify the *syn*- (*s*) and *anti*-isomers (*a*).

Ligand 1AB incorporates two different donors: an unsubstituted pyridine (A), and a bulkier quinoline (B). We previously reported the self-assembly of heteroleptic cage Pd_2_1AB_2_2AA_2_ (Fig. 3a) in CD_3_CN that formed predominantly as the intuitively more sterically encumbered *syn*-isomer.^18^ This result is contrary to chemical intuition and molecular modelling (GFN2-xTB/MeCN^20^ calculated in Orca^21^) of the cationic cage architecture alone (Fig. 2) which does not consider intermolecular interactions. This indicated that interactions beyond the primary structure of the cage were influencing isomer selectivity.

To explore the generality of this design, the self-assembly of 1AB with alternative symmetric ligands 3AA and 4AA (featuring pyridyl and tolyl core units, respectively) was investigated. To this end, 1AB and 3AA/4AA were combined in a 1 : 1 ratio with [Pd(CH_3_CN)_4_](BF_4_)_2_ (used as the source of Pd(ii) throughout this work) in MeCN and equilibrated at 70 °C for 24 h. Formation of the desired [Pd_2_1_2_3/4_2_]^4+^ cage structures was confirmed by ESI-MS, and the existence of both *syn*- and *anti*-isomers demonstrated by NMR spectroscopy (Fig. 3b and c).

The *syn*- and *anti*-isomers of these cages could be readily distinguished by NOESY (*e.g.*Fig. 3d); interactions between protons from the two ends of 1AB (*e.g.* H_*B*_⋯H_*I*_) would only be expected from the antiparallel arrangement present in the *anti*-isomer (and were only observed for the minor species in each instance).

Both Pd_2_1AB23AA_2_ and Pd_2_1AB_2_4AA_2_ formed the *syn*-isomer as the major product in similar amounts to the previously reported Pd_2_1AB_2_2AA_2_ (77 ± 5%; Fig. S292). The identity of the core unit in ligand 2/3/4 was therefore shown not to materially affect isomer selectivity.

### Picoline donor on diverging ligand

**Fig. 4:**
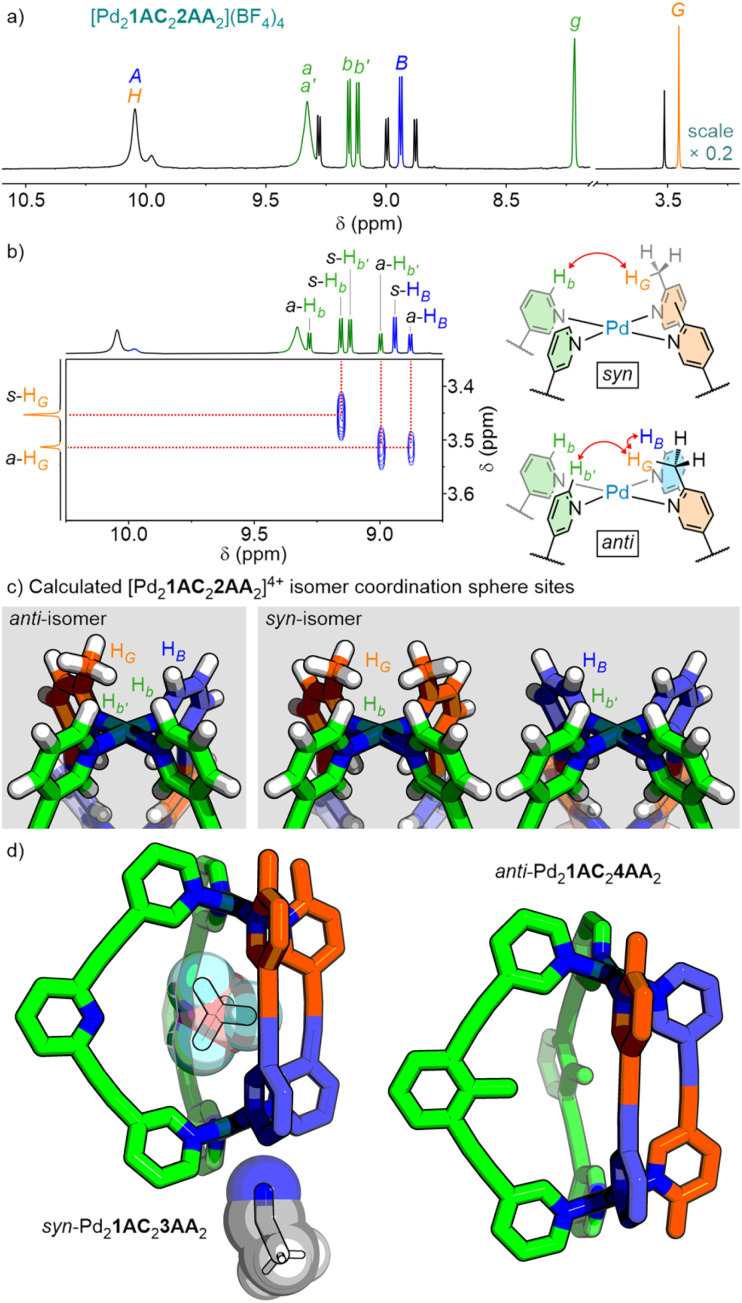
(a) Partial ^1^H NMR spectrum (600 MHz, CD_3_CN, 298 K), with major *syn*-isomer peaks labelled, of [Pd_2_1AC_2_2AA_2_](BF_4_)_4_. (b) Partial NOESY spectrum (600 MHz, CD_3_CN, 298 K) of [Pd_2_1AC_2_2AA_2_](BF_4_)_4_ with key peaks used to identify the *syn*- (*s*) and *anti*-isomers (*a*). (c) Visualisation of the coordination sphere sites of *anti*- and *syn*-Pd_2_1AC_2_2AA_2_ cage isomers from geometry-optimised models (GFN2-xTB). (d) SCXRD structures of *syn*-[Pd_2_1AC_2_3AA_2_]^4+^, showing endohedral BF_4_^−^ counterion and exohedral CH_3_CN solvent molecule interacting with the cage, and *anti*-[Pd_2_1AC_2_4AA_2_]^4+^.

Ligand 1AC features a 2-picolyl donor (C) in combination with an unsubstituted pyridine (A). In contrast to 1AB, 1AC does not possess acidic exohedral protons adjacent to the coordinating atoms of the bulky donor. Based on this, it was predicted that the heteroleptic cages with 2AA, 3AA and 4AA would all form predominantly as the *anti*-isomers to avoid steric clash between the picolyl units. It was somewhat surprising, therefore, that all three of the cages also formed the *syn*-isomers as the major products (∼66 ± 5%; Fig. S293) in CD_3_CN (Fig. 4a and b).

This selectivity was rationalised by comparing the coordination environments of the *syn*- and *anti*-isomers (Fig. 4c). A single picolyl unit on both faces of the *anti*-isomers is sufficient to significantly block interactions with both external coordination spheres of the cages. In contrast, the *syn*-isomers provide two different coordination spheres: one with two picolyl units, and a second, unencumbered, tetrapyridyl environment. The latter provides a single site with acidic protons H_*B*_ and H_*b′*_ that could engage in HB interactions to stabilise the otherwise unfavourable accumulation of steric bulk at the other end of the cage. This idea was supported by single crystal X-ray diffraction (SCXRD) studies (see below). As such, intermolecular non-covalent interactions on just one face of the cages are sufficient to overcome intramolecular steric interactions.

It is noted that Pd_2_1AC_2_4AA_2_ did not form exclusively as the heteroleptic assemblies. This was most likely the result of partial occlusion of the cavity by the tolyl methyl group, inhibiting access for anions/solvents necessary as templates.

The solid-state structures of Pd_2_1AC_2_3AA_2_ and Pd_2_1AC_2_4AA_2_ were determined by SCXRD (Fig. 4d). Intriguingly, Pd_2_1AC_2_4AA_2_ crystallised as the minor *anti*-isomer; this is presumably simply a facet of solid-state packing interactions. The structure of *syn*-Pd_2_1AC_2_3AA_2_ revealed an acetonitrile molecule engaging in quadfurcated hydrogen bond interactions with the tetrapyridyl face of the cage (C–H⋯N 2.62–2.72 Å), supporting the idea that such interactions could stabilise the *syn*-isomers in solution.^22^

### Alternative donor combinations on diverging ligand

**Fig. 5:**
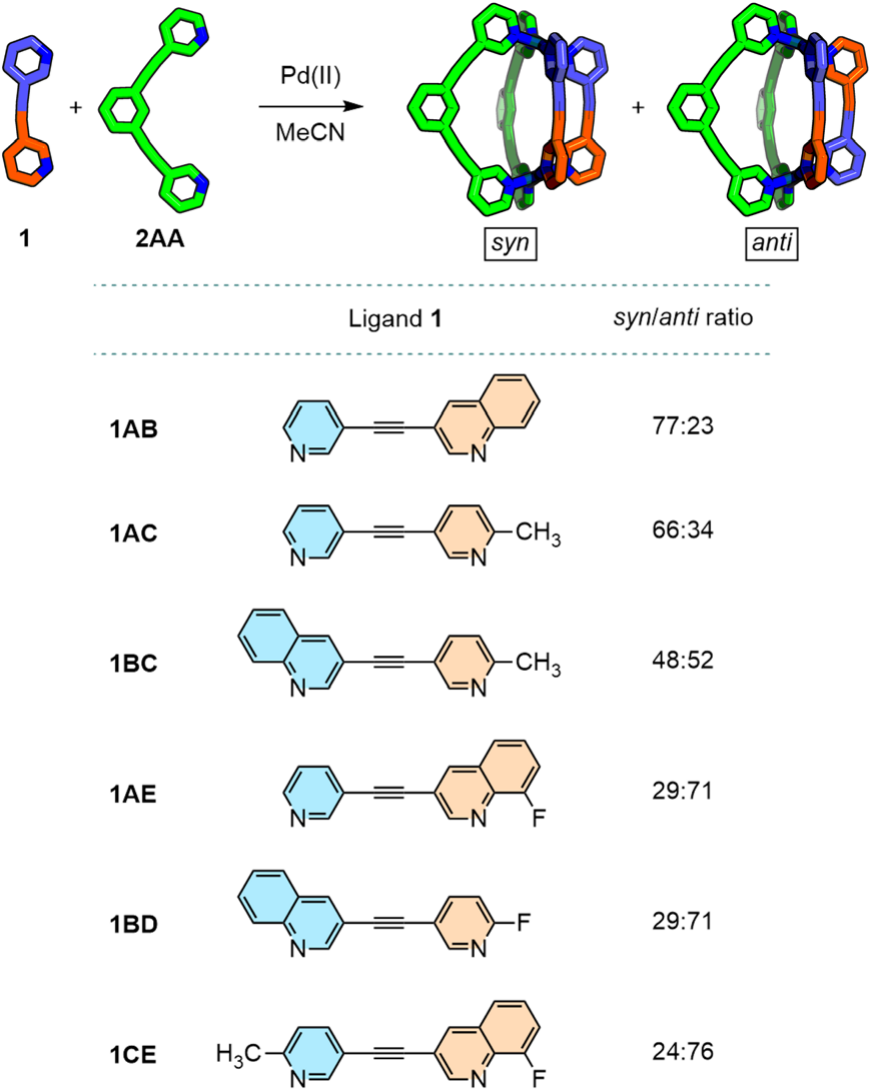
Summary of the observed *syn*/*anti* ratios in CD_3_CN for combinations of low-symmetry ligands 1, with various donor combinations, and symmetrical ligand 2AA.

To see how modifications to the donor units affected isomer selectivity, four variants of 1AB were prepared (Fig. 5). 1BC and 1BD possess quinoline donors (B) combined with 2-picoline (C) or 2-fluoropyridine (D), respectively, whilst an 8-fluoroquinoline (E) donor was incorporated with an unsubstituted pyridine (A) into ligand 1AE. In each of these ligands, compared to 1AB, acidic protons on either the pyridine (1BC and 1BD) or quinoline (1AE) were replaced with moieties that could not act as HB donors. Finally, 1CE, with both picoline and 8-fluoroquinoline donors, was also synthesised.

In the case of Pd_2_1BC_2_2AA_2_, a 1 : 1 mixture of the *syn*- and *anti*-isomers formed (Fig. S294), demonstrating a loss of selectivity. Computational modelling revealed an insignificant (∼1 kJ mol^−1^) energy difference between the two isomers. As such, it can be concluded that the sum of interaction energies in the first and second coordination spheres for each isomer are virtually identical.

**Fig. 6:**
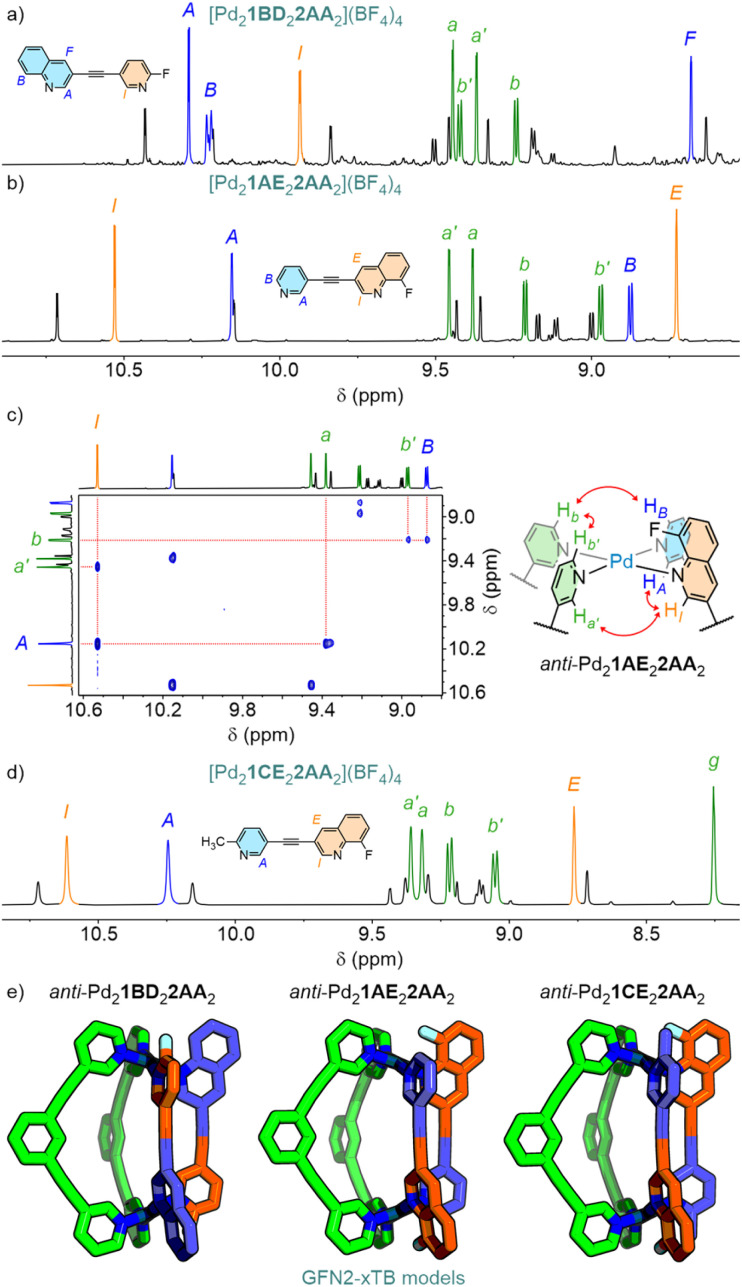
Partial ^1^H NMR spectra (600 MHz, CD_3_CN, 298 K) of (a) [Pd_2_1BD_2_2AA_2_](BF_4_)_4_ and (b) [Pd_2_1AE_2_2AA_2_](BF_4_)_4_ with major *anti*-isomer peaks labelled; partial NOESY spectrum (600 MHz, CD_3_CN, 298 K) of [Pd_2_1AE_2_2AA_2_](BF_4_)_4_ with key peaks used to identify the major *anti*-isomer; (d) partial ^1^H NMR spectrum (400 MHz, CD_3_CN, 298 K) of [Pd_2_1CE_2_2AA_2_](BF_4_)_4_ with major *anti*-isomer peaks labelled; (e) geometry-optimised models (GFN2-xTB) of the major *anti*-isomers of these cages.

Both Pd_2_1BD_2_2AA_2_ and Pd_2_1AE_2_2AA_2_ (Fig. 6a and b) formed predominantly (∼70% each) as the *anti*-isomers (confirmed by NOESY analysis, *e.g.*Fig. 6c). As the substitution of hydrogen atoms for fluorine is widely regarded to have minimal impact on steric bulk,^23^ additional steric hindrance beyond that of Pd_2_1AB_2_2AA_2_ would not seem to be a major factor in the observed inversion of isomer selectivity. The loss of acidic protons capable of forming HB interactions, combined with electrostatic repulsion between fluorine atoms, seem more likely to be the major driving forces in promoting formation of the *anti*-isomer with these systems. As such, Pd_2_1CE_2_2AA_2_, with no acidic exohedral protons on ligand 1, was expected to form almost exclusively as the *anti*-isomer. Although this was indeed the major product (∼76%), the formation of significant amounts of the *syn*-isomer suggested that it was still possible to achieve substantive non-covalent interactions in the second coordination spheres of the *syn*-isomer.

The combinations of donor heterocycles investigated demonstrated varying isomer ratios could be achieved (24 to 77% *syn*). The energy-raising steric interactions between quinoline and picoline units in 1AB and 1AC in *syn*-Pd_2_1_2_2AA_2_ could be offset by stabilising non-covalent interactions with the cages, whilst repulsive interactions between fluorinated heterocycles in 1BD and 1AE were sufficient to promote formation of *anti*-isomers. Indeed, inversion of the isomer selectivity between favouring *anti* and *syn* could be achieved through simply replacing a proton with a fluorine (1AE and 1BD*vs.*1AB), demonstrating the potential for minor structural modifications to be used to drastically alter self-assembly profiles.

### Bulky donors on converging ligand

**Fig. 7:**
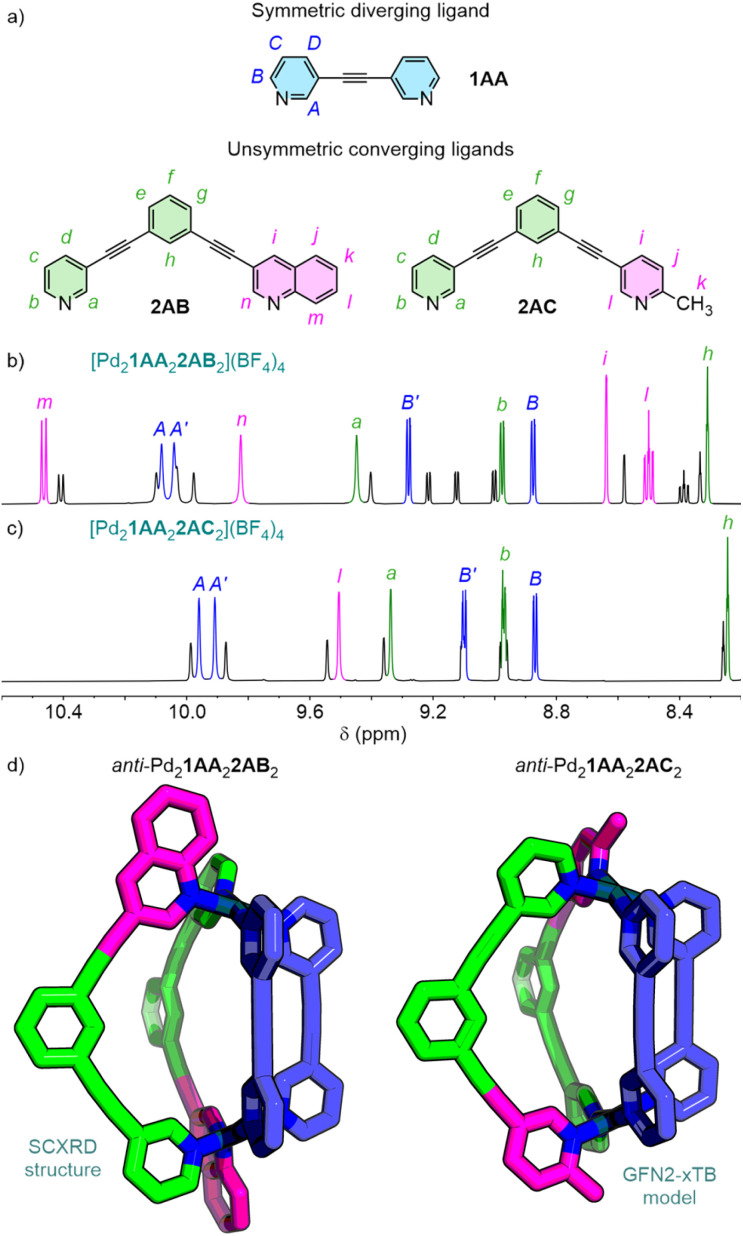
(a) Structure of ligands 1AA, 2AB and 2AC. Partial ^1^H NMR spectra (600 MHz, CD_3_CN, 298 K), with major *anti*-isomer peaks labelled, of (b) [Pd_2_1AA_2_2AB_2_]^4+^ and (c) [Pd_2_1AA_2_2AC_2_]^4+^. (d) Structures of *anti*-isomers of *P*-[Pd_2_1AA_2_2AB_2_]^4+^ (SCXRD) and *M*-[Pd_2_1AA_2_2AC_2_]^4+^ (GFN2-xTB geometry-optimised model).

Having observed preferential formation of the *syn*-isomers of Pd_2_1AB_2_2AA_2_ and Pd_2_1AC_2_2AA_2_, it was sought to determine whether there would be any impact on isomer selectivity from locating the bulky donors on the converging ligand, 2, instead of the diverging ligand. To this end, the heteroleptic cages assembled from 1AA and unsymmetric 2AB/2AC (Fig. 7a) were examined.

In contrast to the previously studied systems with 1AB and 1AC, NMR analysis (in CD_3_CN; Fig. 7b and c) of the Pd_2_1AA_2_2AB/2AC_2_ cages showed the *anti*-isomers to be the major species for both (∼73% (Fig. S298) and ∼70% (Fig. S299) with 2AB and 2AC, respectively). The solid-state structure of *anti*-[Pd_2_1AA_2_2AB_2_](BF_4_)_4_ was also determined by SCXRD (Fig. 7d; the cage crystallised as a racemic mixture of the *P* and *M* enantiomers).^24^ This subtle design change was thus sufficient to shift the balance between coordination sphere interactions driving the isomer selectivity. Geometry-optimised models of the *syn*- and *anti*-isomers of *cis*-[Pd_2_1AA_2_2AB/2AC_2_]^4+^ suggested the former were more sterically crowded compared to the *cis*-[Pd_2_1AB/1AC_2_2AA_2_]^4+^ systems, manifested as an increase in relative computed energy compared to the *anti*-isomers (Δ*E* = 18.3 and 16.1 kJ mol^−1^ for the 2AB and 2AC cages, respectively). Thus, by slightly increasing the steric hindrance between bulky donors in the *syn*-isomers, simply through changing their location within the cages, this became the major driving force in isomer selectivity.

### Cages assembled from two low-symmetry ligands

**Fig. 8:**
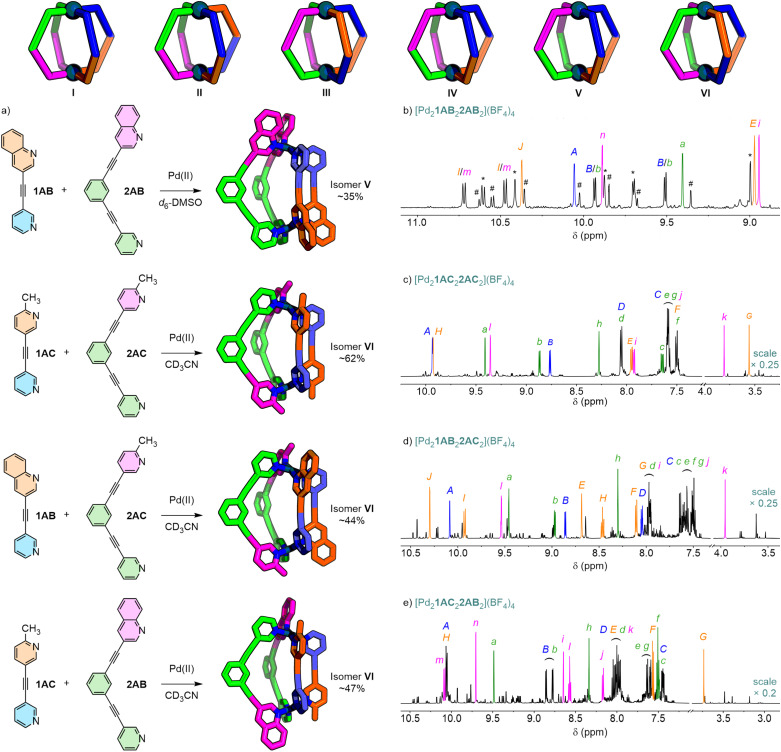
(a) The selective formation of particular isomers of Pd_2_L^A^_2_L^B^_2_-type cages (GXN2-xTB geometry-optimised models shown) from combinations of ligands 1AB/1AC and 2AB/2AC was observed. Partial ^1^H NMR spectra (600 MHz, 298 K) of (b) [Pd_2_1AB_2_2AB_2_](BF_4_)_4_ (*d*_6_-DMSO) with peaks assigned to major isomer V labelled (# = minor isomer; * = [Pd_2_2AB_4_]^4+^); and (c) [Pd_2_1AC_2_2AC_2_](BF_4_)_4_, (d) [Pd_2_1AB_2_2AC_2_](BF_4_)_4_, and (e) [Pd_2_1AC_2_2AB_2_](BF_4_)_4_ (all CD_3_CN) with peaks assigned to major product, isomer VI, labelled.

Finally, the self-assembly of four possible cages from pairs of low-symmetry ligands, namely 1AB, 1AC, 2AB and 2AC, were investigated (Fig. 8a). For such *cis*-Pd_2_L^A^_2_L^B^_2_ systems there are six possible isomers (excluding enantiomers, I–VI; Fig. 8) depending upon the relative orientation of the two different low-symmetry ligands. Based on previous results it was anticipated that isomer VI would inherently be the lowest energy isomer based on steric arguments due to the *trans* arrangement of bulky donor units. This was supported by molecular modelling (GFN2-xTB) that showed VI to be the lowest energy isomer by at least 13 kJ mol^−1^ for all four ligand combinations (Table S8–S11). Selective formation of this isomer would suggest minimising steric hindrance was the primary driving force dictating relative ligand orientation, whilst formation of other isomers would indicate alternative interactions were prominent.

For the combination of 1AB and 2AB an ill-defined mixture formed in CD_3_CN that defied analysis. Repeating the self-assembly in *d*_6_-DMSO resulted in more tractable NMR data (Fig. 8b). Signals in the ^1^H NMR spectrum could be readily identified for the homoleptic assembly of 2AB (∼30% yield). Two additional sets of signals, however, belonged to Pd_2_1AB_2_2AB_2_ cage isomers (the formation of which was confirmed by ESI-MS) forming approximately 35% and 13% of the mixture. NOESY allowed assignment of the major cage isomer as V. As such, preferential formation of a *cis* arrangement of quinoline donors was observed, suggesting that interactions in the second coordination sphere were the major drivers of isomer selectivity for this system.

**Fig. 9:**
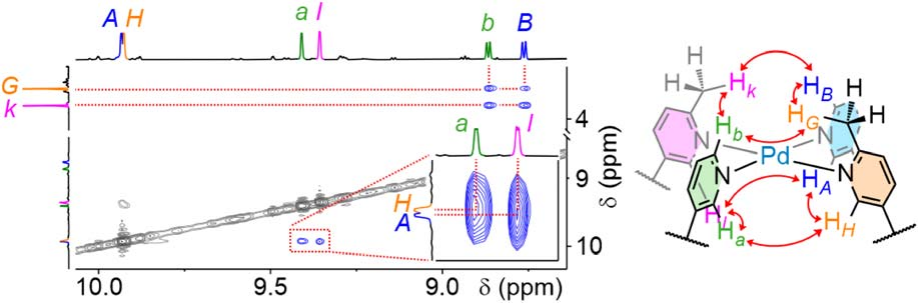
Partial NOESY spectrum (600 MHz, CD_3_CN, 298 K) of [Pd_2_1AC_2_2AC_2_](BF_4_)_4_ identifying the major product as isomer VI.

A single major species was observed to form in ∼62% yield (Fig. S301) from the equilibrated 1 : 1 : 1 mixture of 1AC, 2AC and [Pd(CH_3_CN)_4_](BF_4_)_2_ in CD_3_CN (Fig. 8c). ESI-MS confirmed the anticipated formulation of the assembled heteroleptic structure. NOE coupling (Fig. 9) of both methyl groups (H_*G*_ of 1AC and H_*k*_ of 2AC) with both external *ortho* pyridyl protons (H_*B*_ of 1AC and H_*b*_ of 2AC) was consistent with only two possible isomers, with the picolyl units arranged either *cis* (III) or *trans* (VI) to each other. NOE coupling (Fig. 9 inset) exclusively between internal protons H_*A*_ and H_*l*_ and between H_*H*_ and H_*a*_ (and not H_*A*_⋯H_*a*_ or H_*H*_⋯H_*l*_) and the lack of coupling between H_*b*_ and H_*B*_, however, confirmed that the major product formed was isomer VI. Thus, alleviation of steric hindrance appeared to be the primary driving force in isomer selectivity.

Similarly, both Pd_2_1AB_2_2AC_2_ and Pd_2_1AC_2_2AB_2_ (Fig. 8d and e, respectively) formed isomer VI as the major species (each ∼45% yield; Fig. S302 and S303), with pairs of quinoline and picoline donors arranged *trans* to each other on opposite faces of the cage. Again, this suggested minimising steric interactions was the major driving force at play.

These results are consistent with the earlier systems examined. For alternative isomers than VI to be formed, suitable sites for non-covalent interactions would need to be accessible. This is possible with isomer I, but having all four bulky heterocycles on one face raises the system energy too much (Δ*E* >40 kJ mol^−1^ relative to VI). Isomers that provide a *syn* orientation of 2AB or 2AC (II and V) were previously shown to be relatively unfavourable, while isomers III and IV would place picoline and quinoline units adjacent to each other, inhibiting access to the acidic quinolyl protons. Thus, the preferential formation of isomer VI can be rationalised based on the principles established in this work.

## Conclusions

We have investigated the self-assembly of heteroleptic Pd_2_L^A^_2_L^B^_2_-type coordination cages from low-symmetry ligands incorporating different combinations of heterocyclic donors. Integrative self-assembly of the two ligands is directed by geometric complementarity, while the relative orientation of the low-symmetry scaffolds (*i.e.* isomer selectivity) is driven by coordination sphere interactions. Isomer selectivity could be changed by relatively subtle structural variations, including exchanging a proton for a fluorine atom, or changing the relative locations of heterocycles within the cage structures.

In the case of cages assembled with one low-symmetry ligand, this allowed formation of cages primarily as the *syn*-isomer (up to ∼77%), primarily as the *anti*-isomer (up to ∼76%), or an approximately equal mixture of the two. Selective formation of particular isomers of heteroleptic cages assembled from two low-symmetry ligands (up to ∼62%) was also demonstrated, the assembly of which could be rationally explained from the underlying design principles delineated from this work. As such, we have shown how ligand design can be used to promote interactions in the first or second coordination spheres as the major drivers of isomer selectivity.

Through the combined computational and experimental investigations of the systems explored, it has been possible to gain insight into how structural designs can modulate the relative impact of both intramolecular (first coordination sphere) and intermolecular (second coordination sphere) interactions in directing self-assembly outcomes. In the continued pursuit of developing ever more structurally sophisticated metallo-supramolecular assemblies, understanding (i) how different directing strategies can be used in a synergistic manner, and (ii) the effects of environment (*e.g.* solvent) on thermodynamic self-assembly processes, will enable the design of increasingly structurally and functionally advanced, precision-engineered systems capable of exhibiting bespoke and nuanced properties and behaviours.

## Author contributions

PM carried out the synthesis, characterisation and data analysis. PM and LM collected and analysed the SCXRD data. JEML conceived and directed the project, secured funding, performed molecular modelling, aided data analysis and wrote the manuscript. All authors contributed to editing and approved the final manuscript.

## Conflicts of interest

There are no conflicts to declare.

## Supplementary Material

SC-OLF-D5SC04881H-s001

SC-OLF-D5SC04881H-s002

SC-OLF-D5SC04881H-s003

## Data Availability

CCDC 2390755 ([Pd_2_1AA_2_2AB_2_](BF_4_)_4_), 2390756 ([Pd_2_1AC_2_4AA_2_](BF_4_)_4_) and 2390758 ([Pd_2_1AC_2_3AA_2_](BF_4_)_4_) contain the supplementary crystallographic data for this paper.^25*a–c*^ The data supporting this article have been included as part of the SI. See DOI: https://doi.org/10.1039/d5sc04881h.
