# Supplementary material for: Coordination sphere interactions drive isomer selection in heteroleptic Pd(ii) cages with low-symmetry ligands
Source: Chem Sci. 2025 Sep 8;16(38):17939–47. doi: 10.1039/d5sc04881h (PMC12415893; doi:10.1039/d5sc04881h)
Supplement: SC-016-D5SC04881H-s002 [file SC-016-D5SC04881H-s002.pdf]

Supplementary Information (SI) for Chemical Science.
This journal is © The Royal Society of Chemistry 2025

Electronic Supporting Information

Coordination Sphere Interactions Drive Isomer Selection in Heteroleptic Pd(II) Cages with Low-Symmetry Ligands

Paulina Molinska, Louise Male and James E. M. Lewis*

School of Chemistry, Molecular Sciences Building, University of Birmingham
Edgbaston, Birmingham B15 2TT, United Kingdom
*j.e.m.lewis@bham.ac.uk

Contents

S1. General Experimental 5

S2. Synthetic Procedures 7

Synthesis of S1 7

Synthesis of S2 11

Synthesis of 1AA 15

Synthesis of 1BC 17

Synthesis of 1AE 22

Synthesis of 1BD 27

Synthesis of 1CE 32

Synthesis of 2AA 37

Synthesis of 3AA 40

Synthesis of 4AA 43

Synthesis of 2AB 47

Synthesis of 2AC 50

Synthesis of $[Pd_2(1AB)_2(2AA)_2](BF_4)_4$ 53

Major Isomer *syn*-$[Pd_2(1AB)_2(2AA)_2](BF_4)_4$ 54

Minor Isomer *anti*-$[Pd_2(1AB)_2(2AA)_2](BF_4)_4$ 55

Synthesis of $[Pd_2(1AB)_2(3AA)_2](BF_4)_4$ 61

Major Isomer *syn*-$[Pd_2(1AB)_2(3AA)_2](BF_4)_4$ 62

Minor Isomer *anti*-$[Pd_2(1AB)_2(3AA)_2](BF_4)_4$ 63

Synthesis of $[Pd_2(1AB)_2(4AA)_2](BF_4)_4$ 69

Major Isomer *syn*-$[Pd_2(1AB)_2(4AA)_2](BF_4)_4$ 70

Minor Isomer *anti*-$[Pd_2(1AB)_2(4AA)_2](BF_4)_4$ 71

Synthesis of $[Pd_2(1AC)_2(2AA)_2](BF_4)_4$ 77

Major Isomer *syn*-$[Pd_2(1AC)_2(2AA)_2](BF_4)_4$ 78

Minor Isomer *anti*-$[Pd_2(1AC)_2(2AA)_2](BF_4)_4$ 79

Synthesis of $[Pd_2(1AC)_2(3AA)_2](BF_4)_4$ 85

Major Isomer *syn*-$[Pd_2(1AC)_2(3AA)_2](BF_4)_4$ 86

Minor Isomer *anti*-$[Pd_2(1AC)_2(3AA)_2](BF_4)_4$ 87

Synthesis of $[Pd_2(1AC)_2(4AA)_2](BF_4)_4$ 93

Major Isomer *syn*-$[Pd_2(1AC)_2(4AA)_2](BF_4)_4$ 94

Minor Isomer *anti*-$[Pd_2(1AC)_2(4AA)_2](BF_4)_4$ 95

Synthesis of $[Pd_2(1BC)_2(2AA)_2](BF_4)_4$ 101

Isomer *syn*-$[Pd_2(1BC)_2(2AA)_2](BF_4)_4$ 102

Isomer *anti*-$[Pd_2(1BC)_2(2AA)_2](BF_4)_4$ 103

Synthesis of $[Pd_2(1BD)_2(2AA)_2](BF_4)_4$ 109
Major Isomer *anti*-$[Pd_2(1BD)_2(2AA)_2](BF_4)_4$: 110
Minor Isomer *syn*-$[Pd_2(1BD)_2(2AA)_2](BF_4)_4$ 111
Synthesis of $[Pd_2(1AE)_2(2AA)_2](BF_4)_4$ 117
Major Isomer *anti*-$[Pd_2(1AE)_2(2AA)_2](BF_4)_4$ 118
Synthesis of $[Pd_2(1CE)_2(2AA)_2](BF_4)_4$ 124
Major Isomer *anti*-$[Pd_2(1CE)_2(2AA)_2](BF_4)_4$: 125
Minor Isomer *syn*-$[Pd_2(1CE)_2(2AA)_2](BF_4)_4$: 126
Synthesis of $[Pd_2(1AA)_2(2AB)_2](BF_4)_4$ 133
Major Isomer *anti*-$[Pd_2(1AA)_2(2AB)_2](BF_4)_4$ 134
Minor Isomer *syn*-$[Pd_2(1AA)_2(2AB)_2](BF_4)_4$ 135
Synthesis of $[Pd_2(1AA)_2(2AC)_2](BF_4)_4$ 141
Major Isomer *anti*-$[Pd_2(1AA)_2(2AC)_2](BF_4)_4$ 142
Minor Isomer *syn*-$[Pd_2(1AA)_2(2AC)_2](BF_4)_4$ 143
Synthesis of $[Pd_2(1AB)_2(2AB)_2](BF_4)_4$ 149
Identification of major isomer V of $[Pd_2(1AB)_2(2AB)_2](BF_4)_4$ 151
Synthesis of $[Pd_2(1AC)_2(2AC)_2](BF_4)_4$ 157
Synthesis of $[Pd_2(1AC)_2(2AB)_2](BF_4)_4$ 163
Synthesis of $[Pd_2(1AB)_2(2AC)_2](BF_4)_4$ 169
S3. Isomer distribution comparisons 175
$[Pd_2(1AB)_2(2/3/4AA)_2](BF_4)_4$ 175
$[Pd_2(1AC)_2(2/3/4AA)_2](BF_4)_4$ 176
$[Pd_2(1BC)_2(2AA)_2](BF_4)_4$ 177
$[Pd_2(1BD)_2(2AA)_2](BF_4)_4$ 177
$[Pd_2(1AE)_2(2AA)_2](BF_4)_4$ 178
$[Pd_2(1CE)_2(2AA)_2](BF_4)_4$ 178
$[Pd_2(1AA)_2(2AB)_2](BF_4)_4$ 179
$[Pd_2(1AA)_2(2AC)_2](BF_4)_4$ 179
$[Pd_2(1AB)_2(2AB)_2](BF_4)_4$ 180
$[Pd_2(1AC)_2(2AC)_2](BF_4)_4$ 180
$[Pd_2(1AB)_2(2AC)_2](BF_4)_4$ 181
$[Pd_2(1AC)_2(2AB)_2](BF_4)_4$ 181
S4. Solvent and Anion Studies 182
S5. Concentration Studies 187
S6. Geometry Optimised Structures 189
S7. X-ray Crystallography 191
anti-$[Pd_2(1AA)_2(2AB)_2](BF_4)_4$ 191

anti-[$Pd_2(1AC)_2(4AA)_2$](BF_4)$_4$ 193
syn-[$Pd_2(1AC)_2(3AA)_2$](BF_4)$_4$ 195
S8. References 196

S1. General Experimental

Synthesis: **1AB**,[18] **1AC**,[18] 3-((3-iodophenyl)ethynyl)quinoline[18] and 3-((3-iodophenyl)ethynyl)pyridine[14b] were synthesised according to literature procedure. Unless otherwise stated, all reagents, including anhydrous solvents, were purchased from commercial sources and used without further purification. $CDCl_3$ was stored over 4 Å molecular sieves prior to use. All reactions were carried out under an atmosphere of N_2 using degassed, anhydrous solvents unless otherwise stated. Analytical TLC was performed on pre-coated silica gel plates (0.25 mm thick, 60F254, Merck, Germany) and observed under UV light. Sealed vial reactions were performed in CEM microwave vials, with crimped aluminium caps with PTFE septa. Flash column chromatography was performed on a Biotage Selekt Enkel using Biotage Sfär Silica cartridges.

Analysis: NMR spectra were recorded on Bruker 400 MHz or 600 MHz instrument, at a constant temperature of 298 K. Chemical shifts are reported in parts per million from low to high field and referenced to residual solvent. Standard abbreviations indicating multiplicity were used as follows: m = multiplet, quint = quintet, q = quartet, t = triplet, d = doublet, s = singlet, app. = apparent, br. = broad. Signal assignment was carried out using 2D NMR methods (HSQC, HMBC, COSY, NOESY) where necessary. In the case of some signals absolute assignment was not possible. Here indicative either/or assignments (e.g. H_A/H_B for H_A or H_B) are provided. Mass spectrometry was carried out by the Mass Spectrometry for Chemistry Research facility at the School of Chemistry, University of Birmingham using a Waters Synapt G2-S or Waters Xevo G2-XS.

^{1}H NMR signal assignment:

syn-[Pd$_2$**1AB**$_2$**2AA**$_2$]

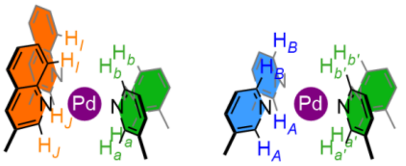

anti-[Pd$_2$**1AB**$_2$**2AA**$_2$]

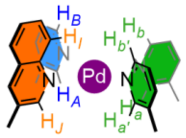

syn-[Pd$_2$**1AC**$_2$**2AA**$_2$]

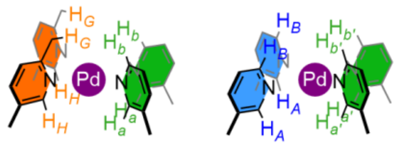

anti-[Pd$_2$**1AC**$_2$**2AA**$_2$]

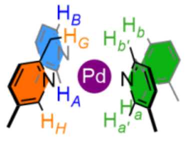

syn-[Pd$_2$**1AA**$_2$**2AB**$_2$]

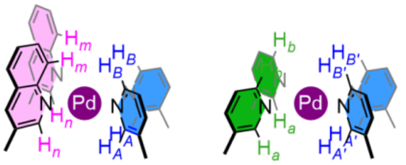

anti-[Pd$_2$**1AA**$_2$**2AB**$_2$]

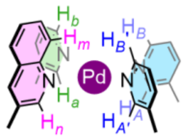

syn-[Pd$_2$**1AA**$_2$**2AC**$_2$]

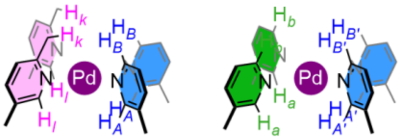

anti-[Pd$_2$**1AA**$_2$**2AC**$_2$]

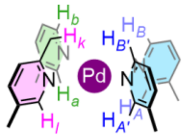

syn-[Pd$_2$**1BD**$_2$**2AA**$_2$]

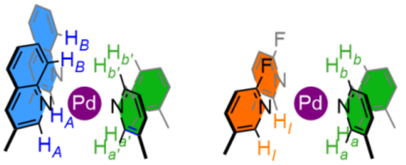

anti-[Pd$_2$**1BD**$_2$**2AA**$_2$]

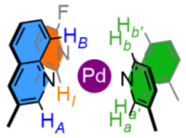

syn-[Pd$_2$**1AE**$_2$**2AA**$_2$]

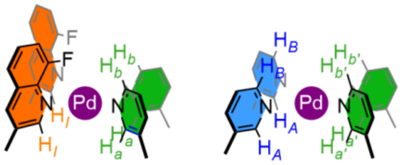

anti-[Pd$_2$**1AE**$_2$**2AA**$_2$]

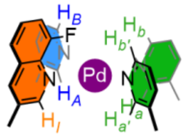

syn-[Pd$_2$**1CE**$_2$**2AA**$_2$]

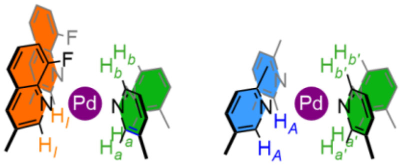

anti-[Pd$_2$**1CE**$_2$**2AA**$_2$]

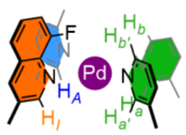

S2. Synthetic Procedures

Synthesis of S1

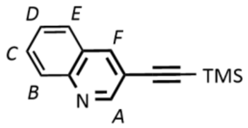

To a stirring solution of 3-bromoquinoline (0.628 g, 3.0 mmol, 1.0 eq.), $Pd(PPh_3)_2Cl_2$ (0.053 g, 0.08 mmol, 2.5 mol%), CuI (0.015 g, 0.08 mmol, 2.5 mol%) in THF (7.5 mL) and $^{i}Pr_2NH$ (7.5 mL) in a sealed vial was added trimethylsilylacetylene (0.64 mL, 4.5 mmol, 1.5 eq.) via syringe. The reaction mixture was stirred at 60 °C for 24 h. 0.1 M $EDTA_{(aq)}$ solution (35 mL) was added to the cooled reaction mixture, and the aqueous phase subsequently extracted with CH_2Cl_2 (3 × 35 mL). The combined organic phases were dried ($MgSO_4$), and the solvent removed *in vacuo*. After purification by column chromatography on silica gel (step gradient 0 to 3% EtOAc in hexane in 1% increments) the product was obtained as an orange oil (0.605 g, 90%).

^{1}H NMR (400 MHz, $CDCl_3$) δ: 8.92 (d, *J* = 2.1 Hz, 1H, H_A), 8.26 (dd, *J* = 2.2, 0.8 Hz, 1H, H_F), 8.08 (dd, *J* = 8.4, 1.0 Hz, 1H, H_B), 7.77 (dd, *J* = 8.1, 1.5 Hz, 1H, H_E), 7.71 (ddd, *J* = 8.5, 6.9, 1.5 Hz, 1H, H_C), 7.55 (ddd, *J* = 8.1, 6.9, 1.2 Hz, 1H, H_D), 0.30 (s, 9H, H_{TMS}).

^{13}C NMR (101 MHz, $CDCl_3$) δ: 152.46, 147.03, 139.04, 130.30, 129.55, 127.74, 127.41, 127.24, 117.44, 102.22, 98.36, 0.02.

HR-ESI-MS *m/z* = 226.1053 $[M+H]^+$ calc. 226.1052 (Δ = 0.44 ppm).

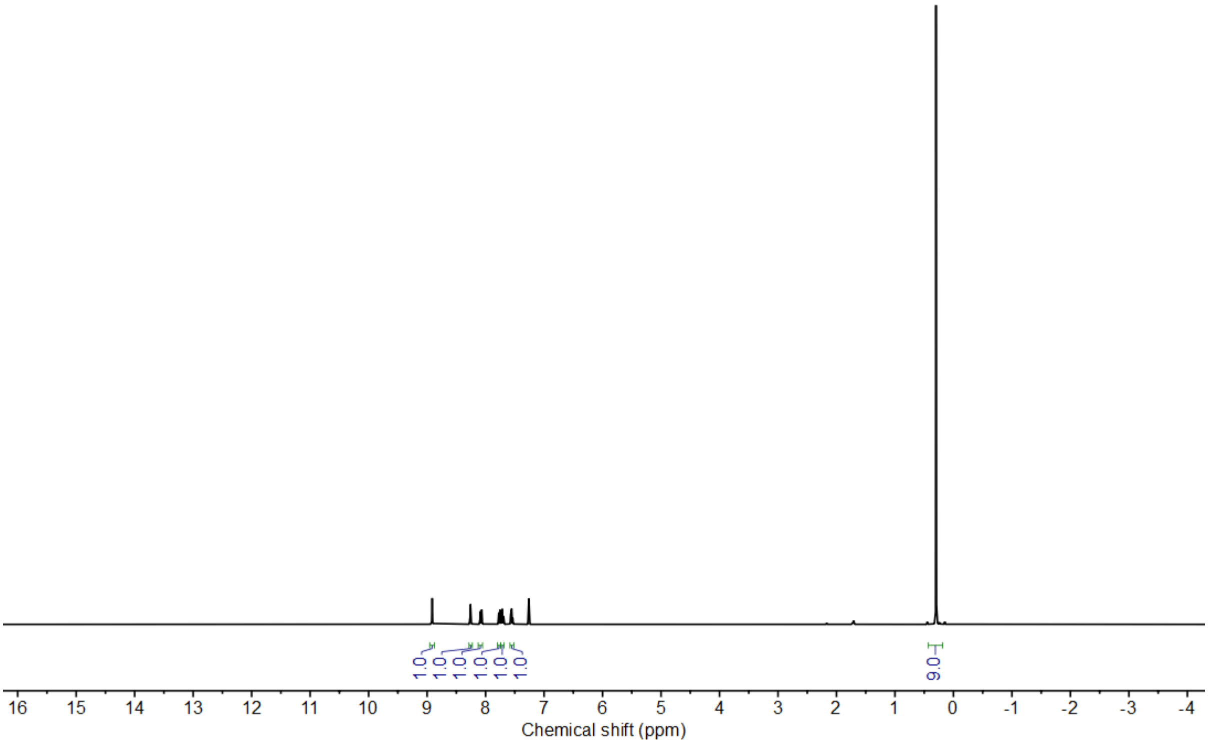

Figure S1 ^{1}H NMR (400 MHz, $CDCl_3$) of S1.

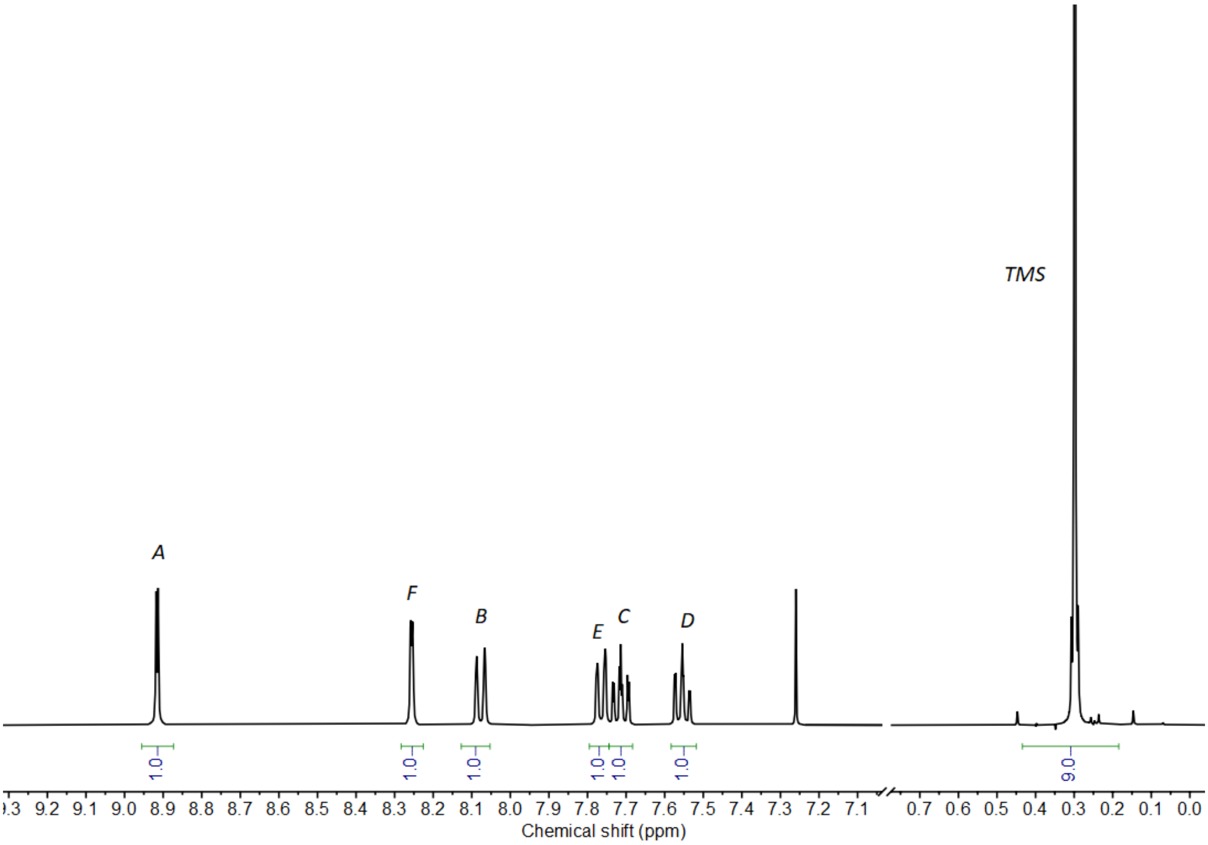

Figure S2 Partial ^{1}H NMR (400 MHz, $CDCl_3$) of S1.

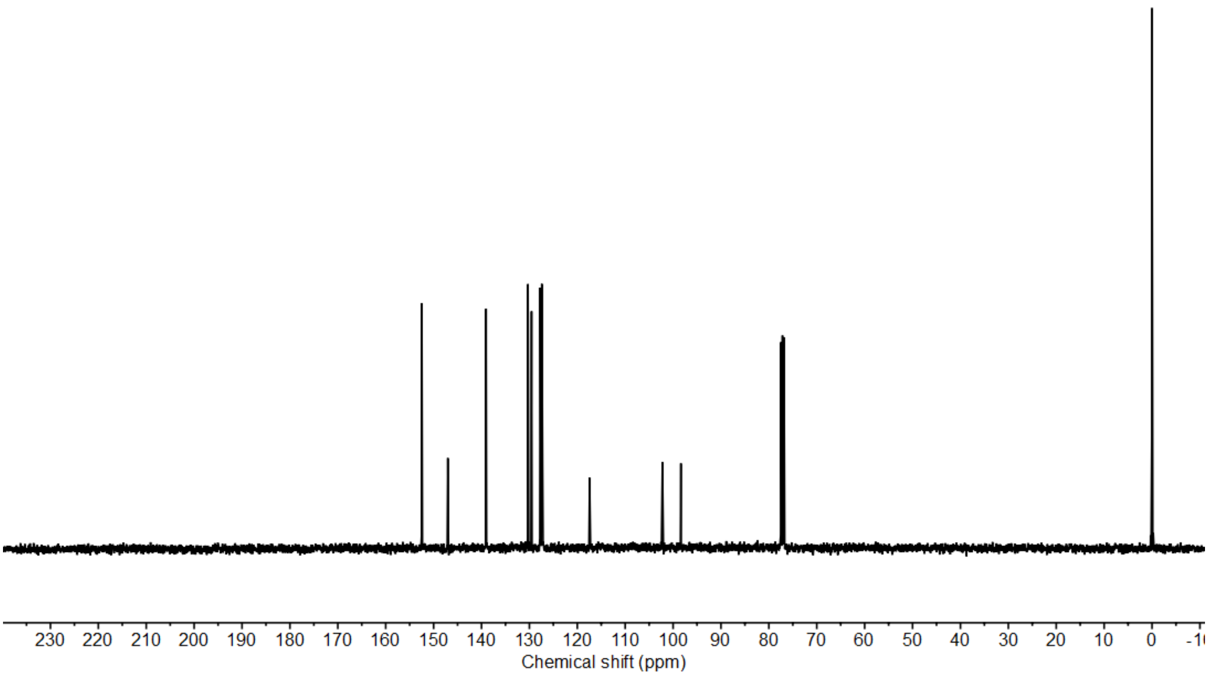

Figure S3 ^{13}C NMR (101 MHz, $CDCl_3$) of S1.

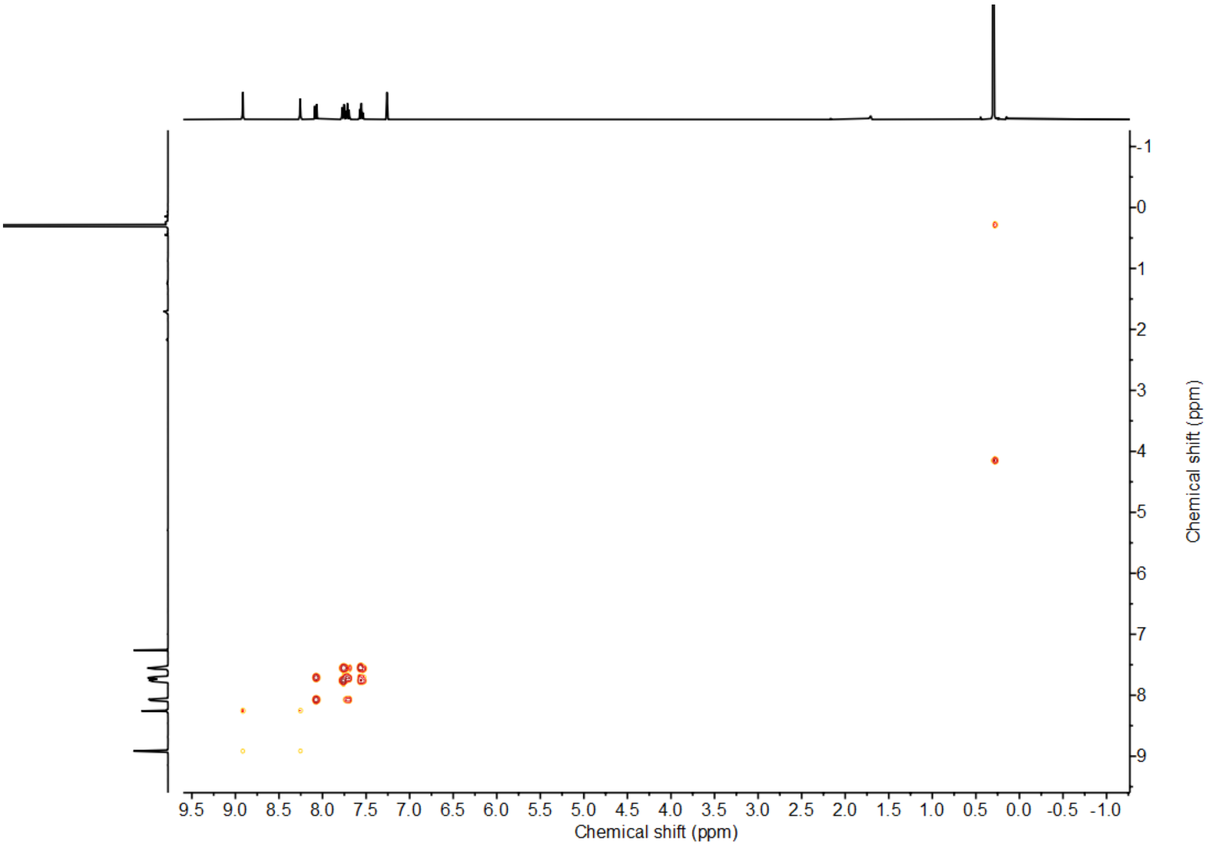

Figure S4 COSY ($CDCl_3$) of S1.

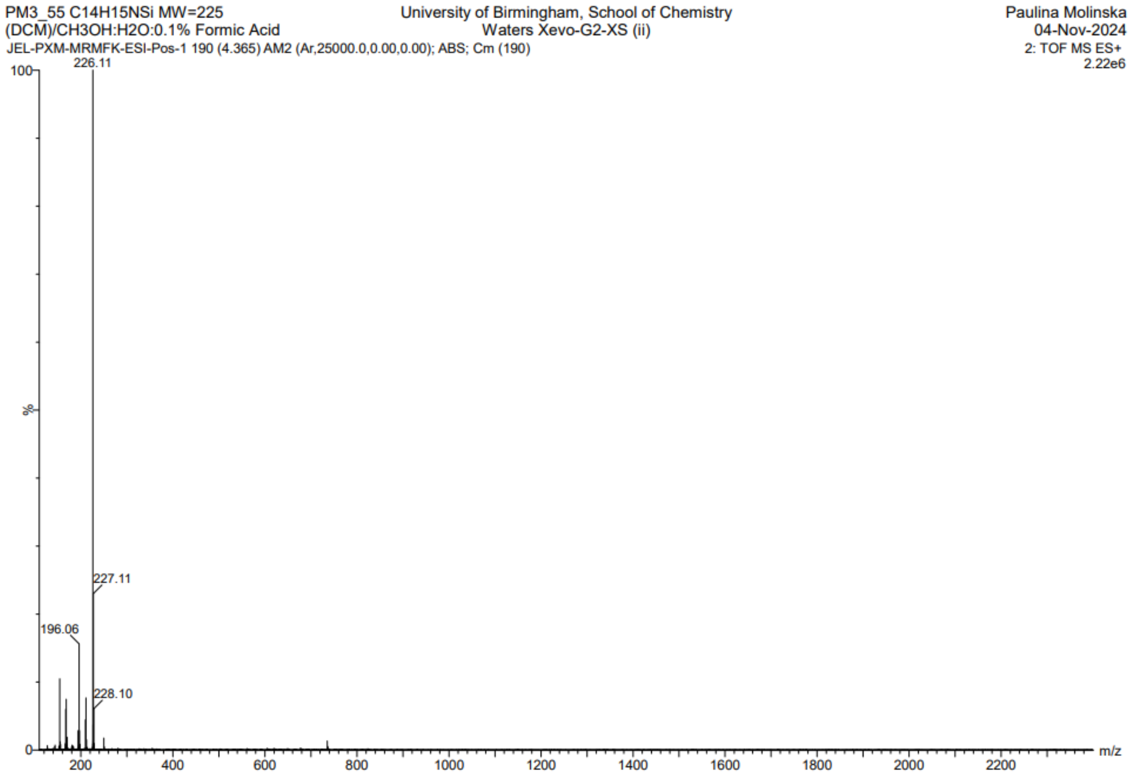

Figure S5 ESI-MS of S1.

Synthesis of S2

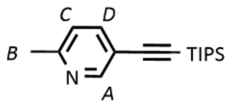

To a stirring solution of 5-bromo-2-methylpyridine (0.344 g, 2.0 mmol, 1.0 eq.), $Pd(PPh_3)_2Cl_2$ (0.071 g, 0.1 mmol, 5 mol%), CuI (0.038 g, 0.2 mmol, 10 mol%) in $^iPr_2NH$ (5 mL) in a sealed vial was added ethynyltriisopropylsilane (1.335 mL, 6.0 mmol, 3.0 eq.) via syringe. The reaction mixture was stirred at 100 °C for 1 week. 0.1 M $EDTA_{(aq)}$ solution (35 mL) was added to the cooled reaction mixture, and the aqueous phase subsequently extracted with CH_2Cl_2 (3 × 35 mL). The combined organic phases were dried ($MgSO_4$), and the solvent removed *in vacuo*. After purification by column chromatography on silica gel (step gradient 0 to 20% EtOAc in hexane in 5% increments) the product was obtained as a brown oil (0.509 g, 93%).

^{1}H NMR (400 MHz, $CDCl_3$) δ: 8.57 (dd, *J* = 2.2, 0.9 Hz, 1H, H_A), 7.63 (dd, *J* = 8.0, 2.2 Hz, 1H, H_D), 7.09 (d, *J* = 7.9 Hz, 1H, H_A), 2.55 (s, 3H, H_B), 1.12 (d, *J* = 1.8 Hz, 22H, H_{TIPS}).

^{13}C NMR (101 MHz, $CDCl_3$) δ: 157.87, 152.20 (C_A), 139.30 (C_D), 122.65 (C_C), 117.69, 103.90, 93.85, 24.64 (C_B), 18.78 (C_{TIPS}), 11.40 (C_{TIPS}).

HR-ESI-MS *m/z* = 274.2000 $[M+H]^+$ calc. 274.1991 (Δ = 3.3 ppm).

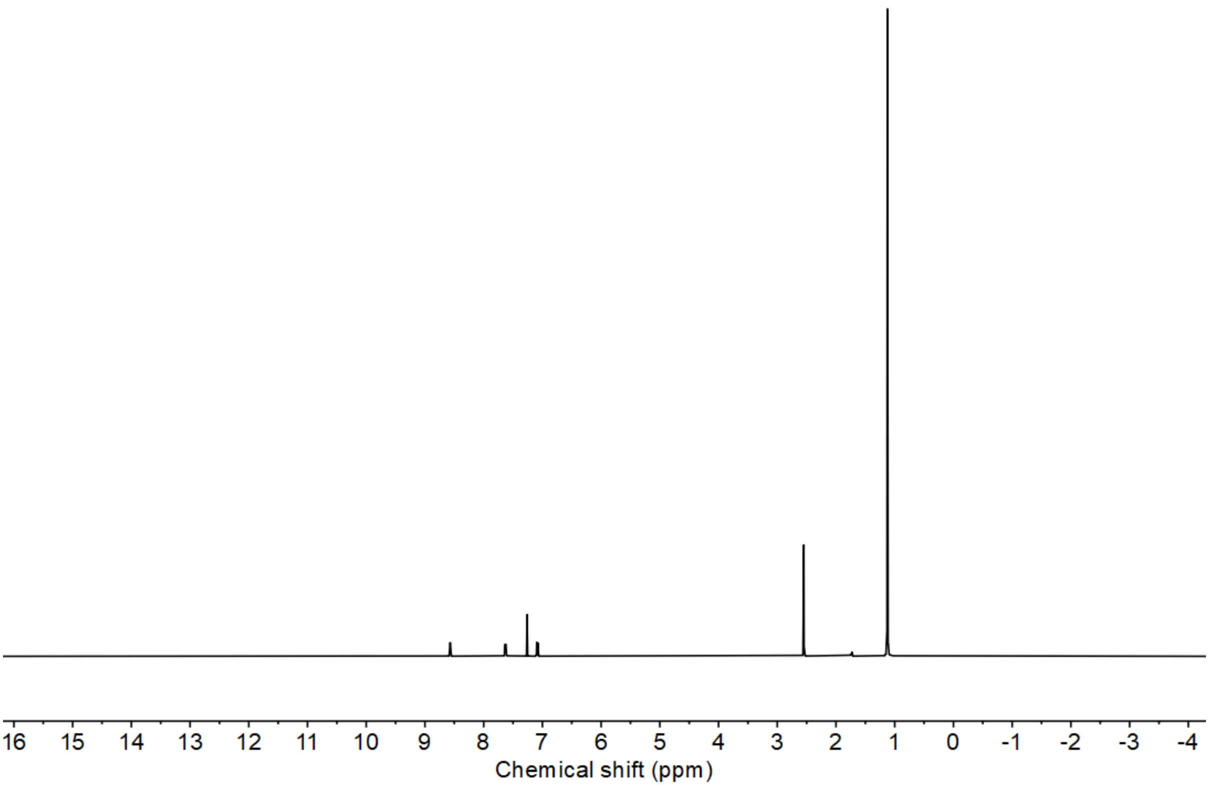

Figure S6 ^{1}H NMR (400 MHz, $CDCl_3$) of S2.

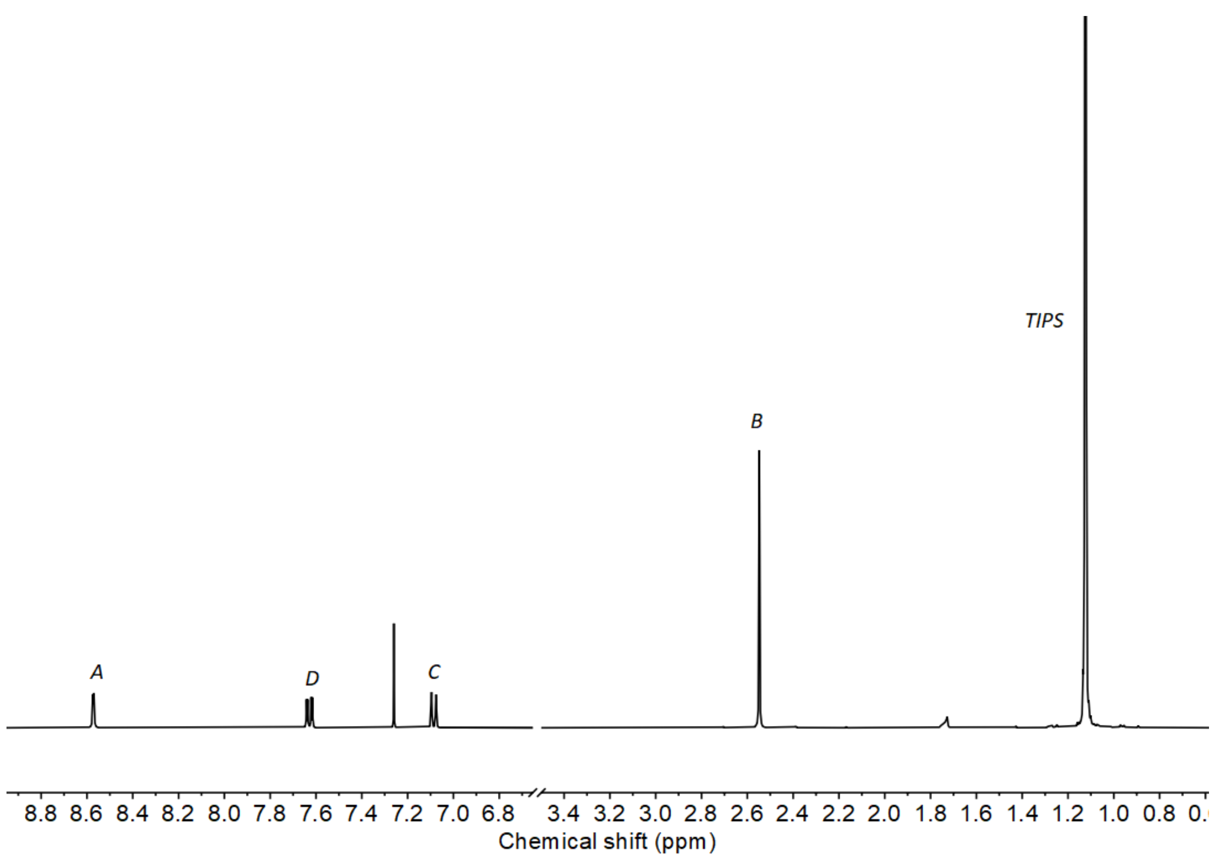

Figure S7 Partial ^{1}H NMR (400 MHz, $CDCl_3$) of S2.

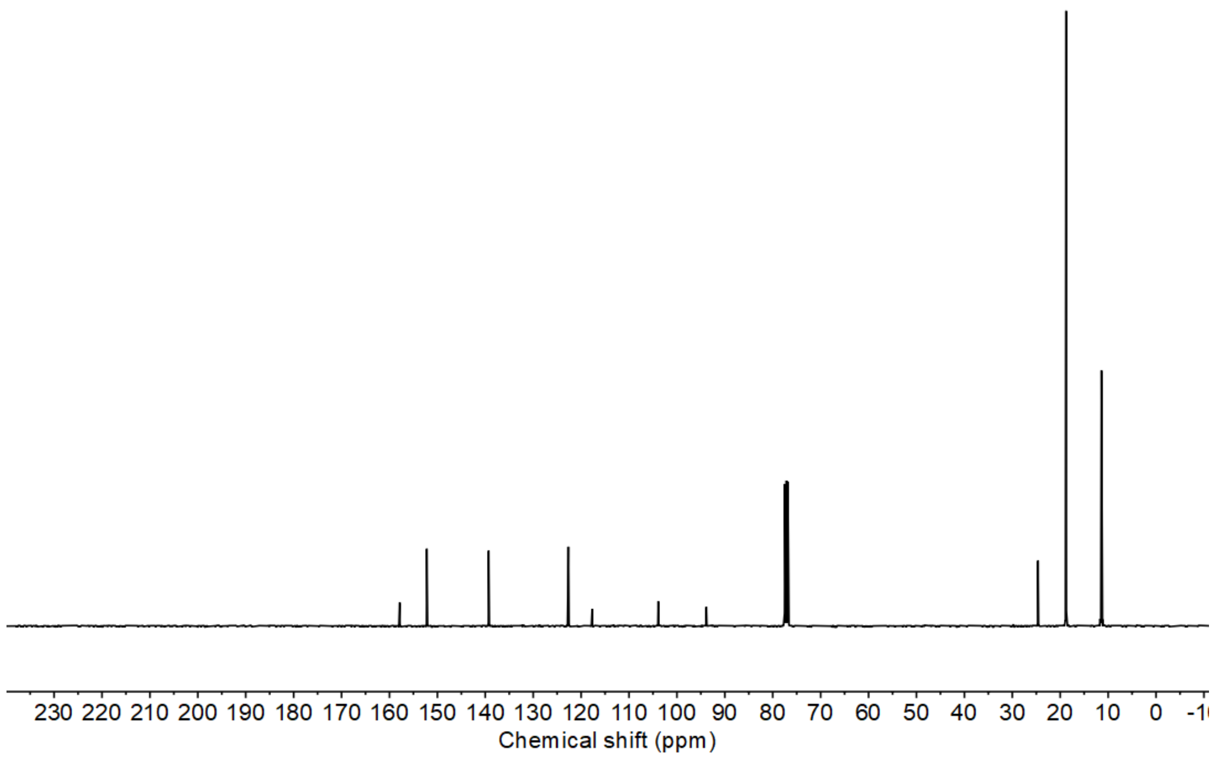

Figure S8 ^{13}C NMR (101 MHz, $CDCl_3$) of S2.

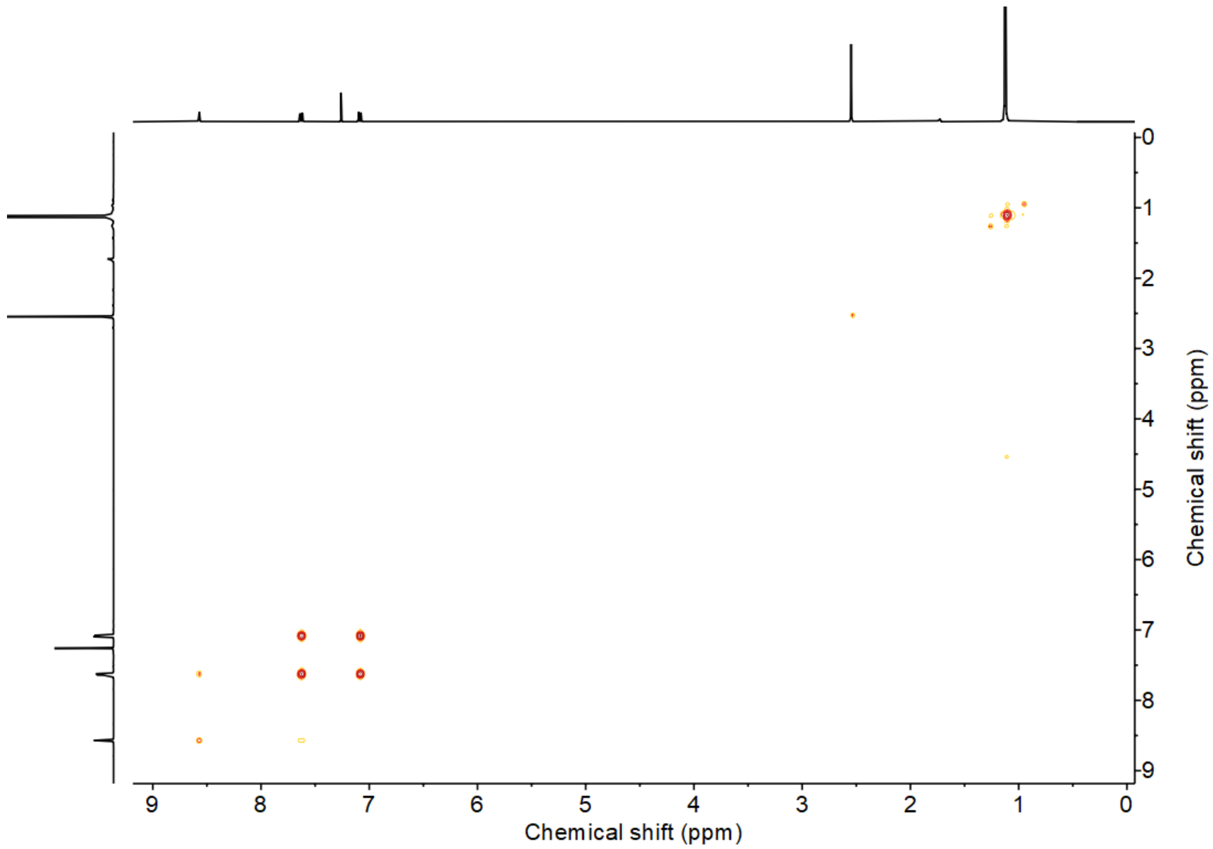

Figure S9 COSY ($CDCl_3$) of S2.

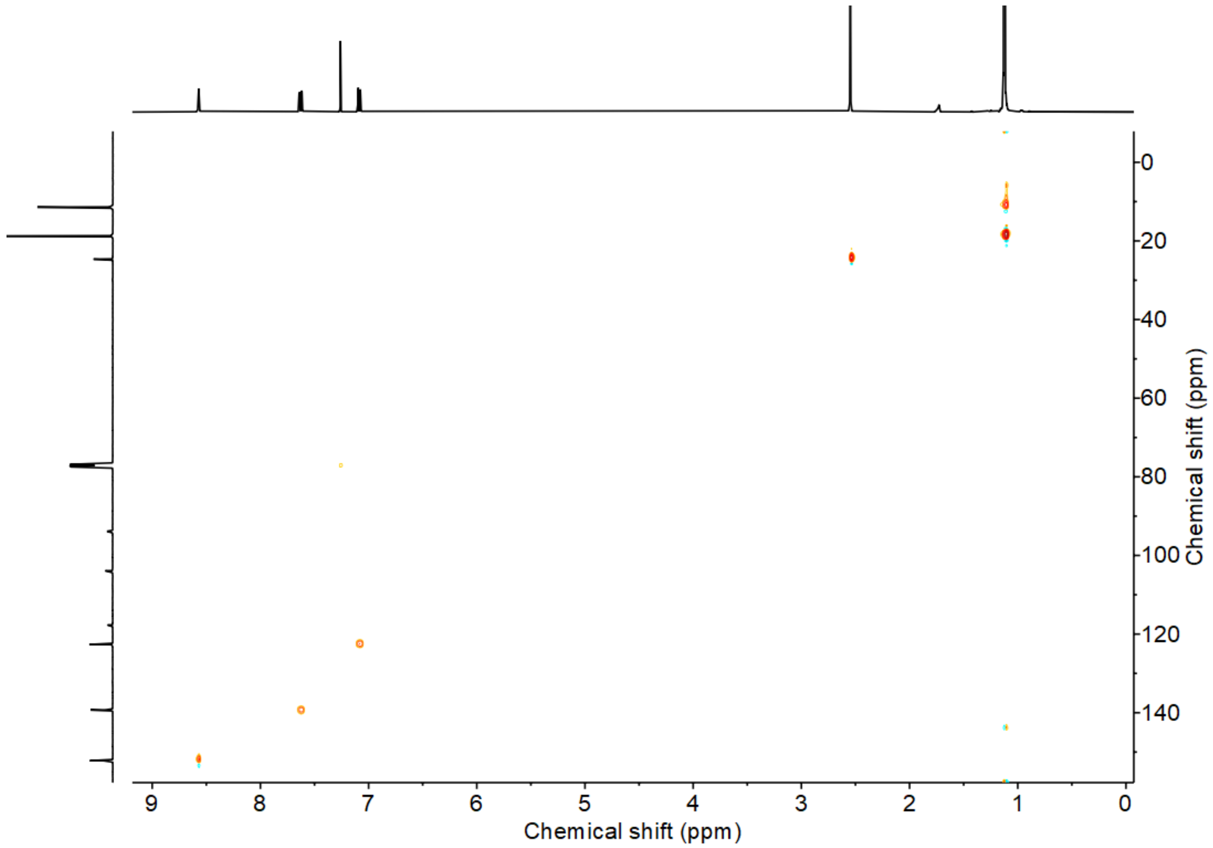

Figure S10 HSQC ($CDCl_3$) of S2.

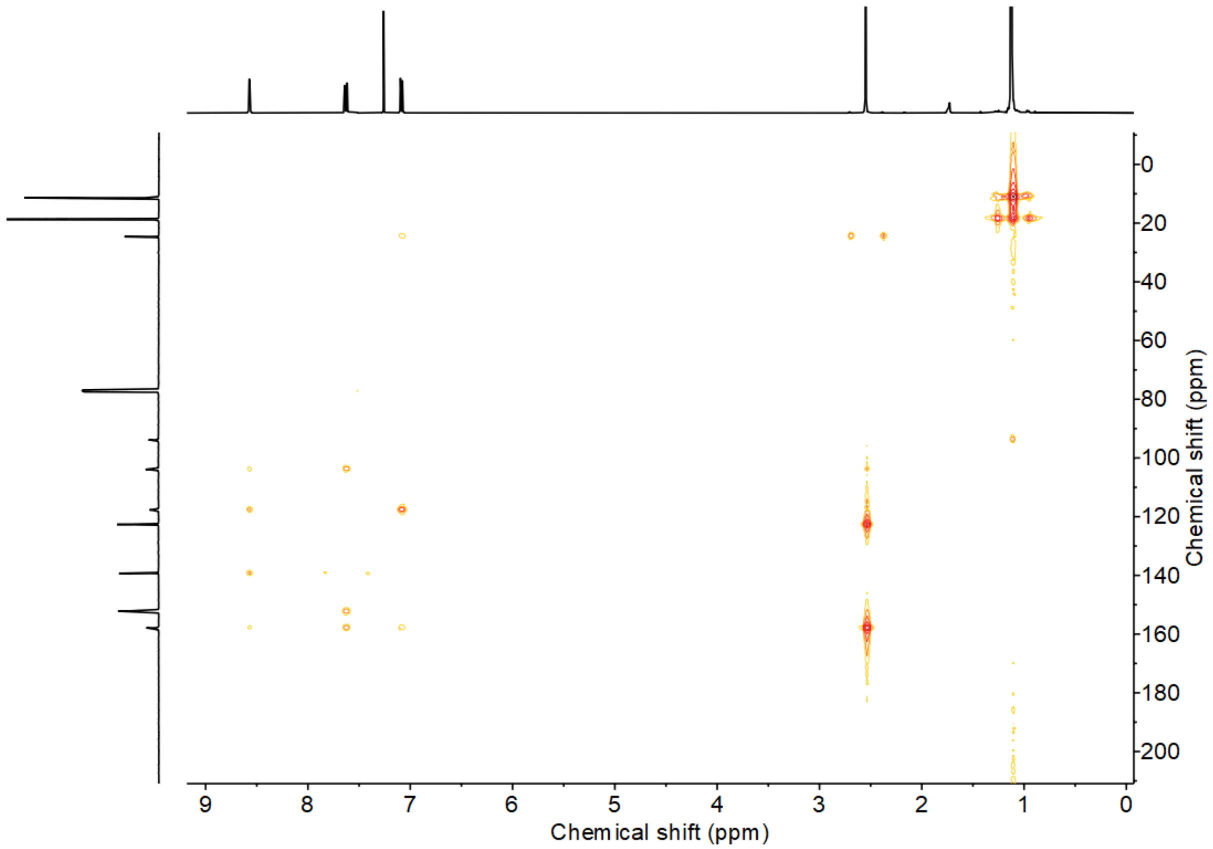

Figure S11 HMBC ($CDCl_3$) of S2.

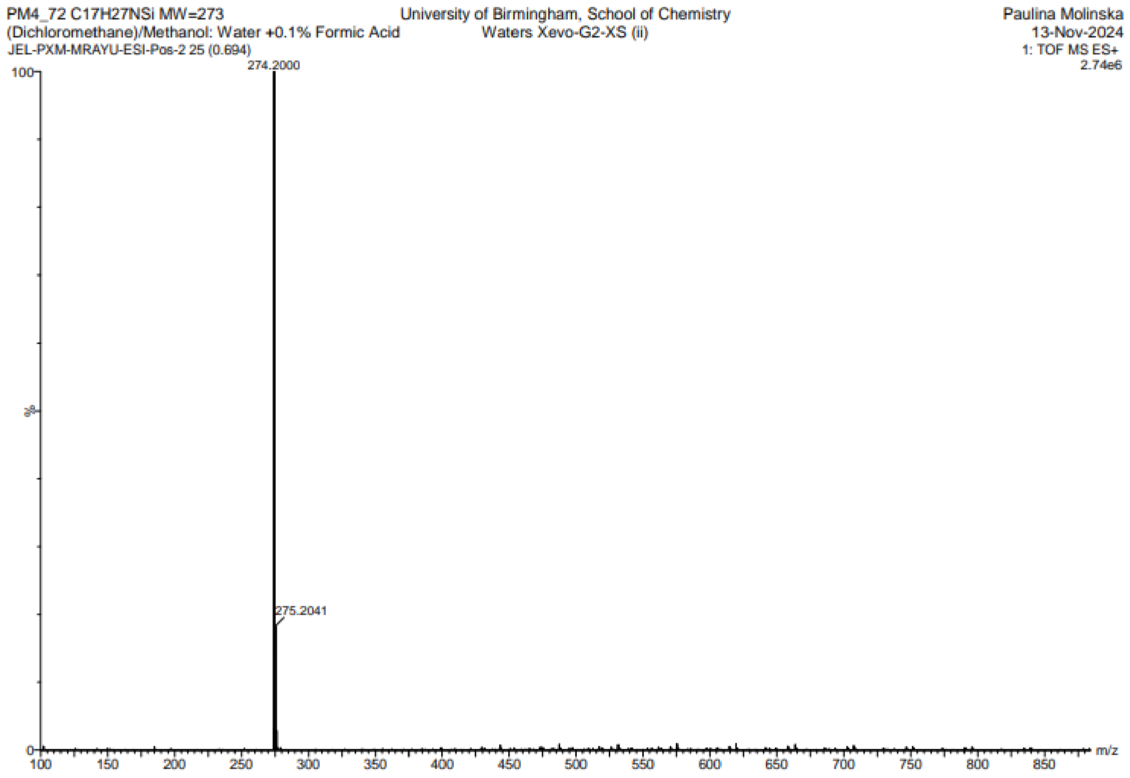

Figure S12 ESI-MS of S2.

Synthesis of 1AA

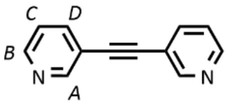

3-Iodopyridine (0.205 g, 1.0 mmol, 1.0 eq.), 3-ethynylpyridine (0.108 g, 1.0 mmol, 1.0 eq.) and Pd(dppf)$Cl_2 \cdot CH_2Cl_2$ (0.040 g, 0.05 mmol, 5 mol%) were stirred at 80 °C in 1.0 M TBAF in THF (5 mL) in a sealed vial for 24 h. Brine (30 mL) was added to the cooled reaction mixture, and the aqueous phase subsequently extracted with Et_2O (3 × 30 mL). The combined organic phases were washed with 5% wt. aq. LiCl solution (2 x 30 mL), dried ($MgSO_4$) and the solvent removed *in vacuo*. After purification by column chromatography on silica gel (gradient 0 to 20% acetone in CH_2Cl_2) the product was obtained as a brown solid (0.110 g, 57%).

Spectroscopic data were consistent with a previous literature report.[19]

^{1}H NMR (400 MHz, $CDCl_3$) δ: 8.79 (dd, *J* = 2.2, 0.9 Hz, 2H, H_A), 8.58 (dd, *J* = 4.9, 1.7 Hz, 2H, H_B), 7.83 (dt, *J* = 7.9, 1.9 Hz, 2H, H_D), 7.31 (ddd, *J* = 7.9, 4.9, 0.9 Hz, 2H, H_C).

^{13}C NMR (101 MHz, $CDCl_3$) δ: 152.46, 149.25, 138.68, 123.25, 119.90, 89.31.

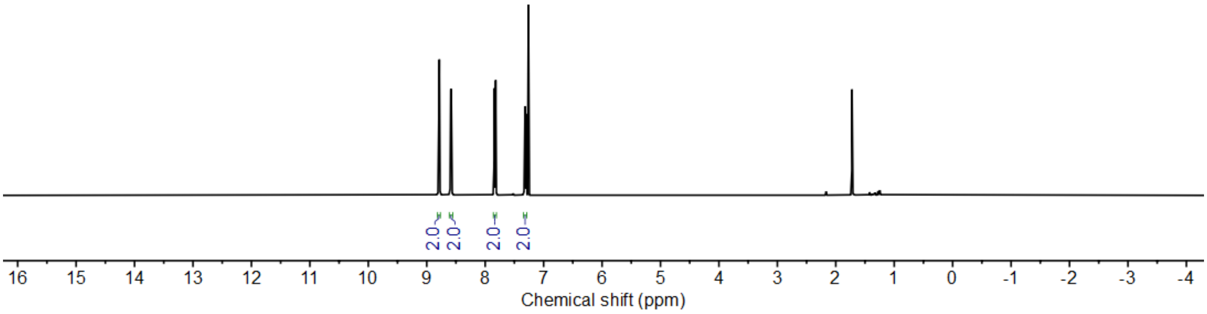

Figure S13 ^{1}H NMR (400 MHz, $CDCl_3$) of 1AA.

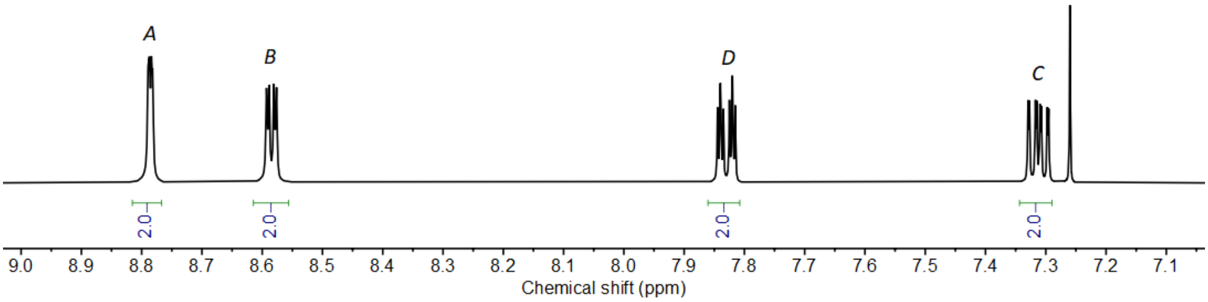

Figure S14 Partial ^{1}H NMR (400 MHz, $CDCl_3$) of 1AA.

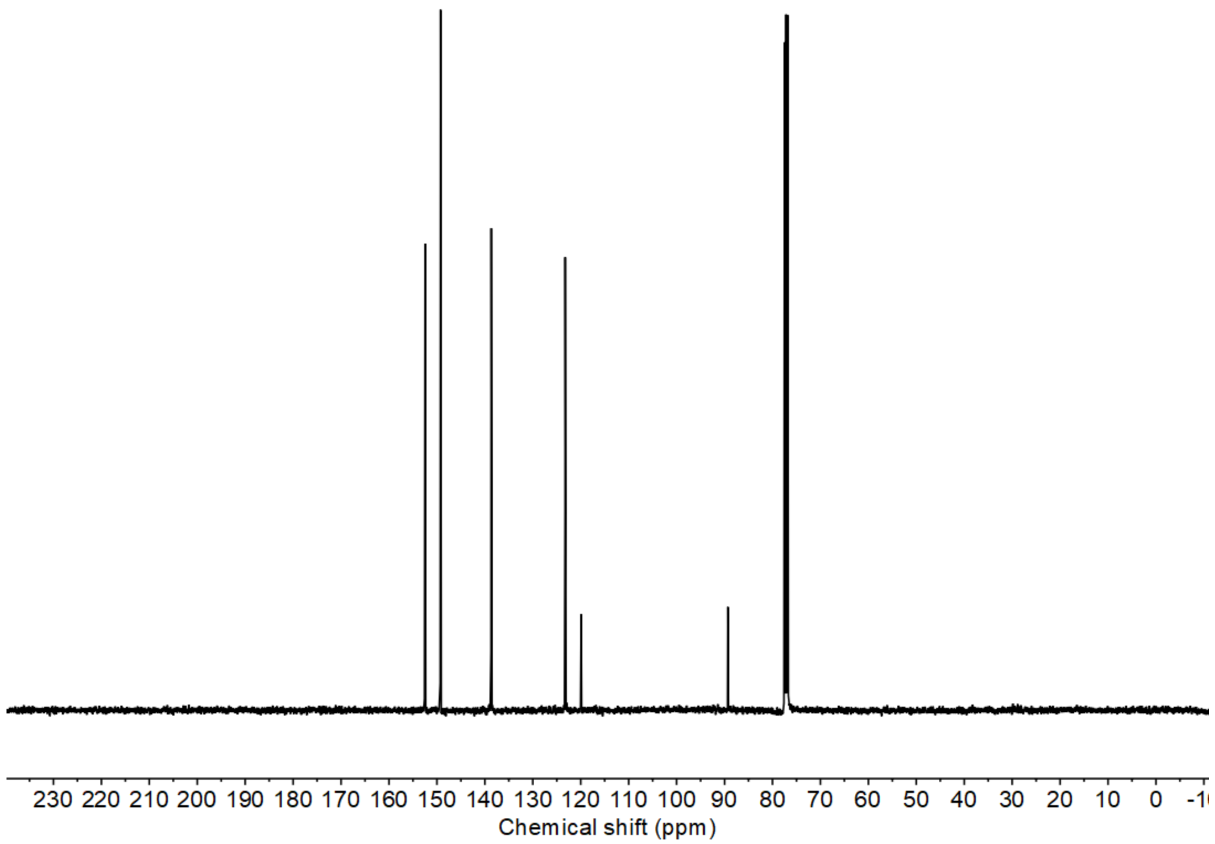

Figure S15 ^{13}C NMR (101 MHz, $CDCl_3$) of 1AA.

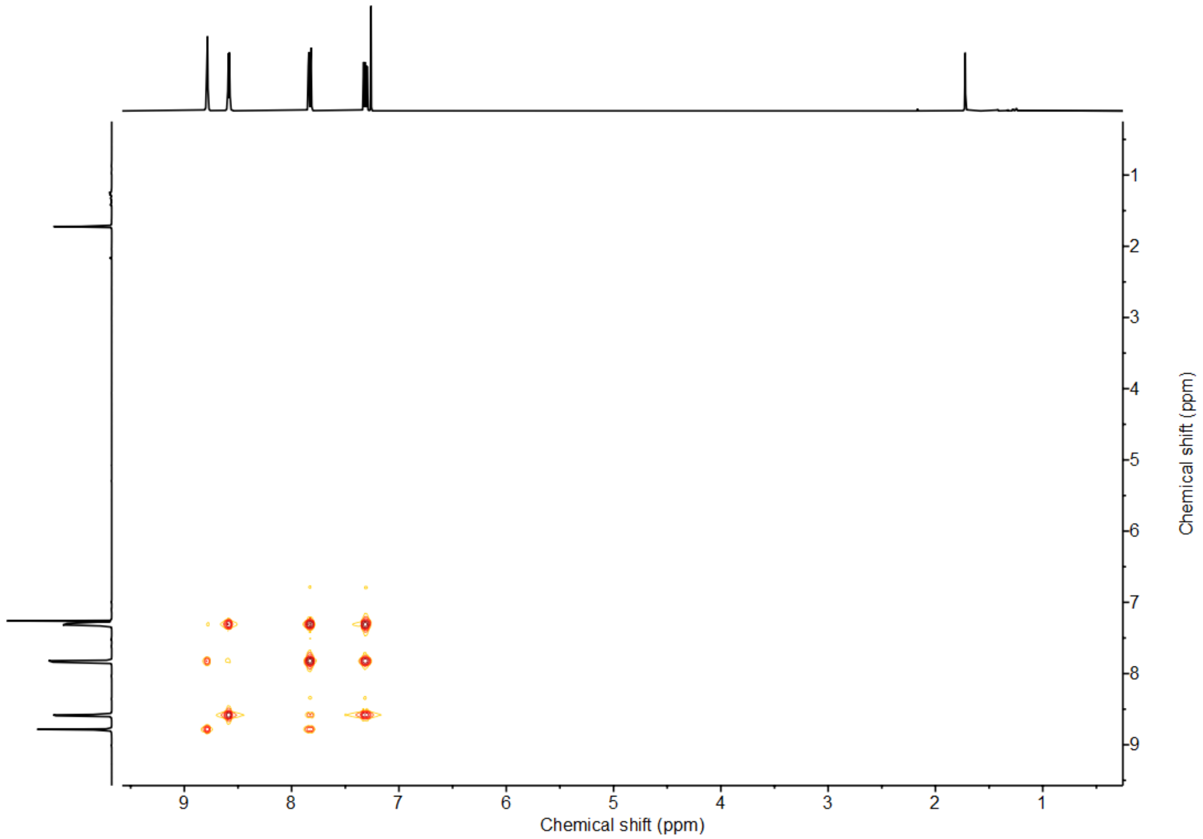

Figure S16 COSY ($CDCl_3$) of 1AA.

Synthesis of 1BC

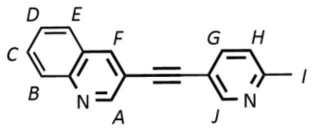

S1 (0.111 g, 0.5 mmol, 1.0 eq.), 5-bromo-2-methylpyridine (0.087 g, 0.5 mmol, 1.0 eq.) and Pd(dppf)$Cl_2 \cdot CH_2Cl_2$ (0.021 g, 0.025 mmol, 5 mol%) were stirred at 80 °C in 1.0 M TBAF in THF (5 mL) in a sealed vial for 24 h. Brine (30 mL) was added to the cooled reaction mixture, and the aqueous phase subsequently extracted with Et_2O (3 × 30 mL). The combined organic phases were washed with 5% wt. aq. LiCl solution (2 x 30 mL), dried ($MgSO_4$) and the solvent removed *in vacuo*. After purification by column chromatography on silica gel (gradient 0 to 23% acetone in CH_2Cl_2) the product was obtained as an off-white solid (0.045 g, 37%).

^{1}H NMR (400 MHz, $CDCl_3$) δ: 9.00 (d, *J* = 2.1 Hz, 1H, H_A), 8.72 (d, *J* = 2.4 Hz, 1H, H_J), 8.33 (dd, *J* = 2.1, 0.8 Hz, 1H, H_F), 8.11 (dd, *J* = 8.4, 1.0 Hz, 1H, H_B), 7.82 (m, 1H, H_E), 7.80-7.70 (m, 2H, H_G, H_C), 7.59 (ddd, *J* = 8.1, 6.9, 1.2 Hz, 1H, H_D), 7.19 (d, *J* = 8.0 Hz, 1H, H_H)), 2.60 (s, 3H, H_I).

^{13}C NMR (101 MHz, $CDCl_3$) δ: 158.54, 152.08 (C_J), 151.88 (C_A), 147.13, 138.95 (C_C), 138.62 (C_F), 130.45 (C_G), 129.62 (C_B), 127.81 (C_E), 127.57 (C_D), 127.35, 122.98 (C_H), 117.10, 116.88, 89.61, 89.28, 24.74.

HR-ESI-MS *m/z* = 245.1083 $[M+H]^+$ calc. 245.1079 (Δ = 1.6 ppm).

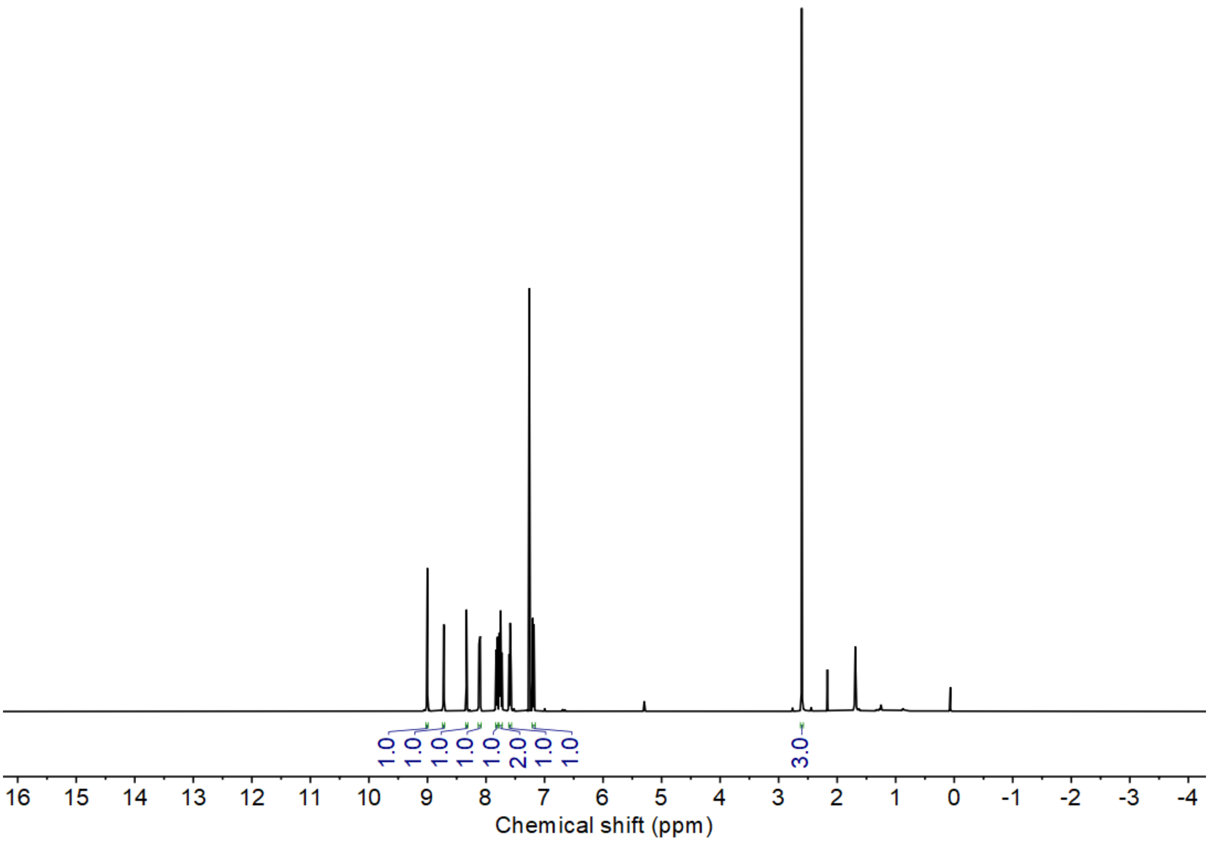

Figure S17 ^{1}H NMR (400 MHz, $CDCl_3$) of 1BC.

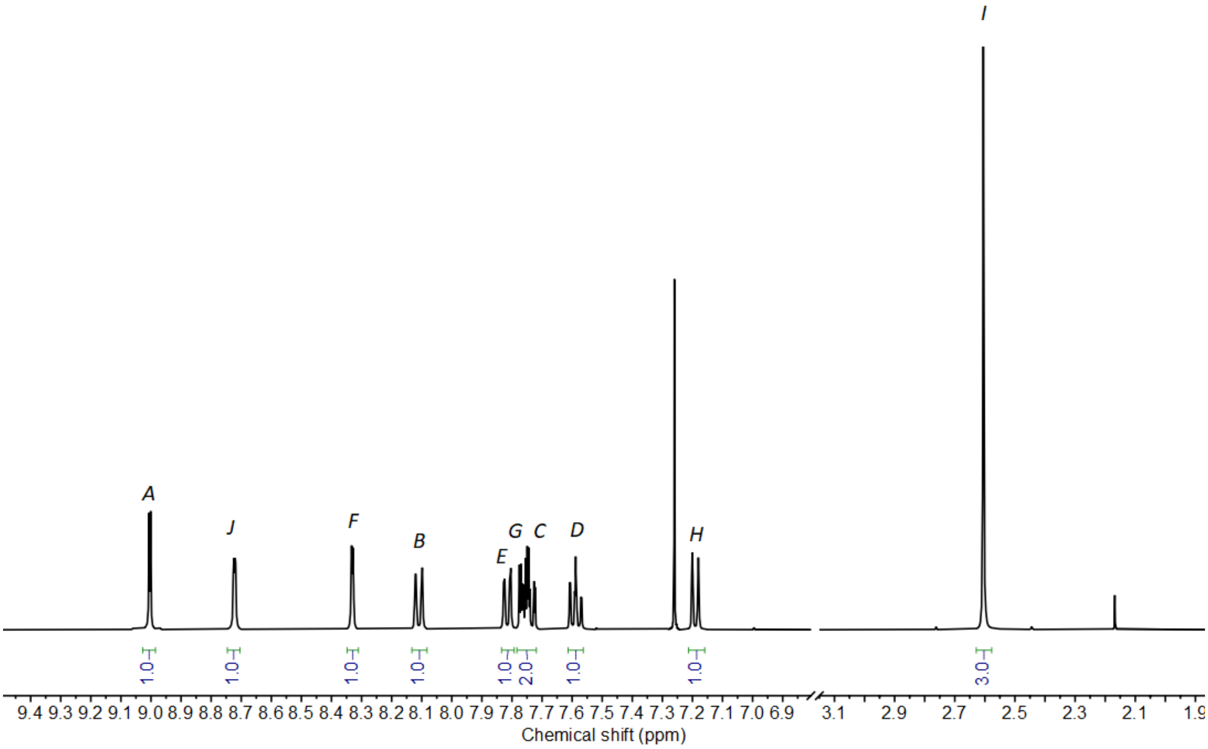

Figure S18 Partial ^{1}H NMR (400 MHz, $CDCl_3$) of 1BC.

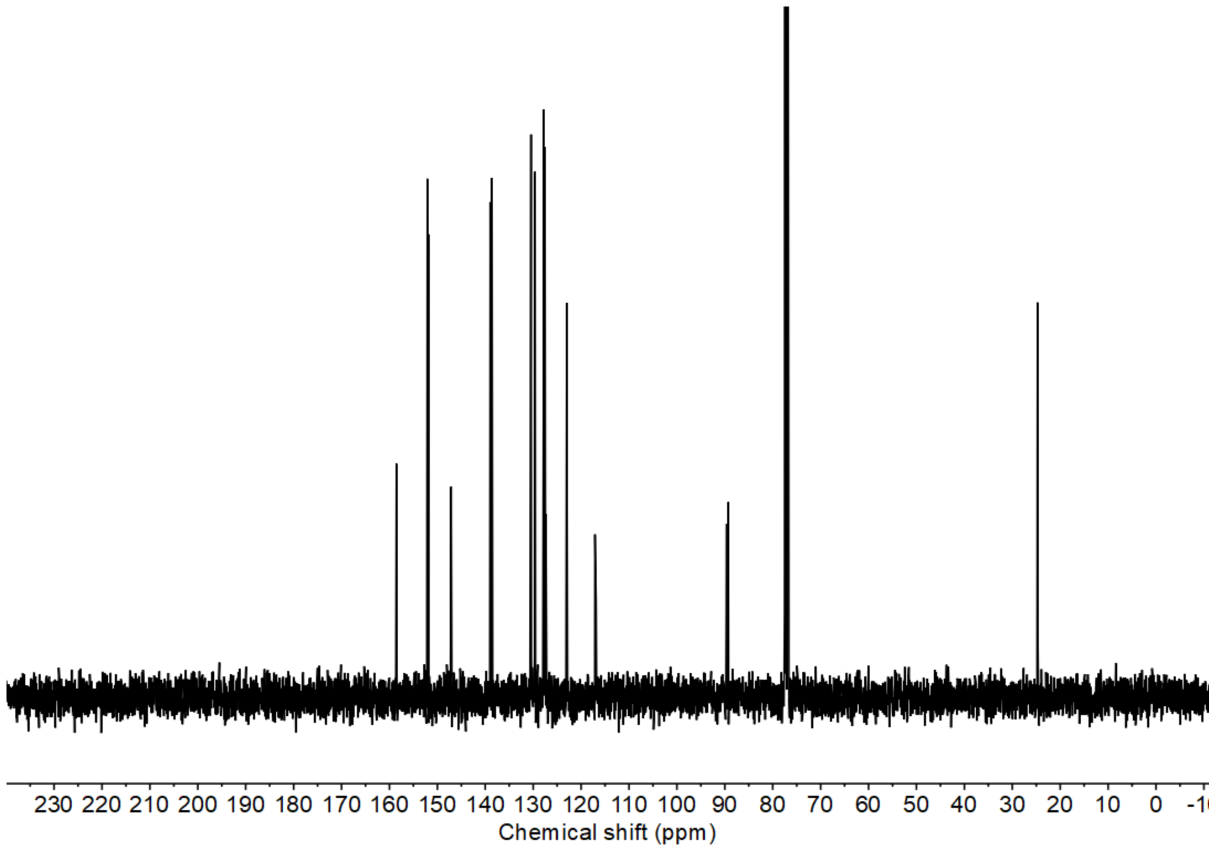

Figure S19 ^{13}C NMR (101 MHz, $CDCl_3$) of 1BC.

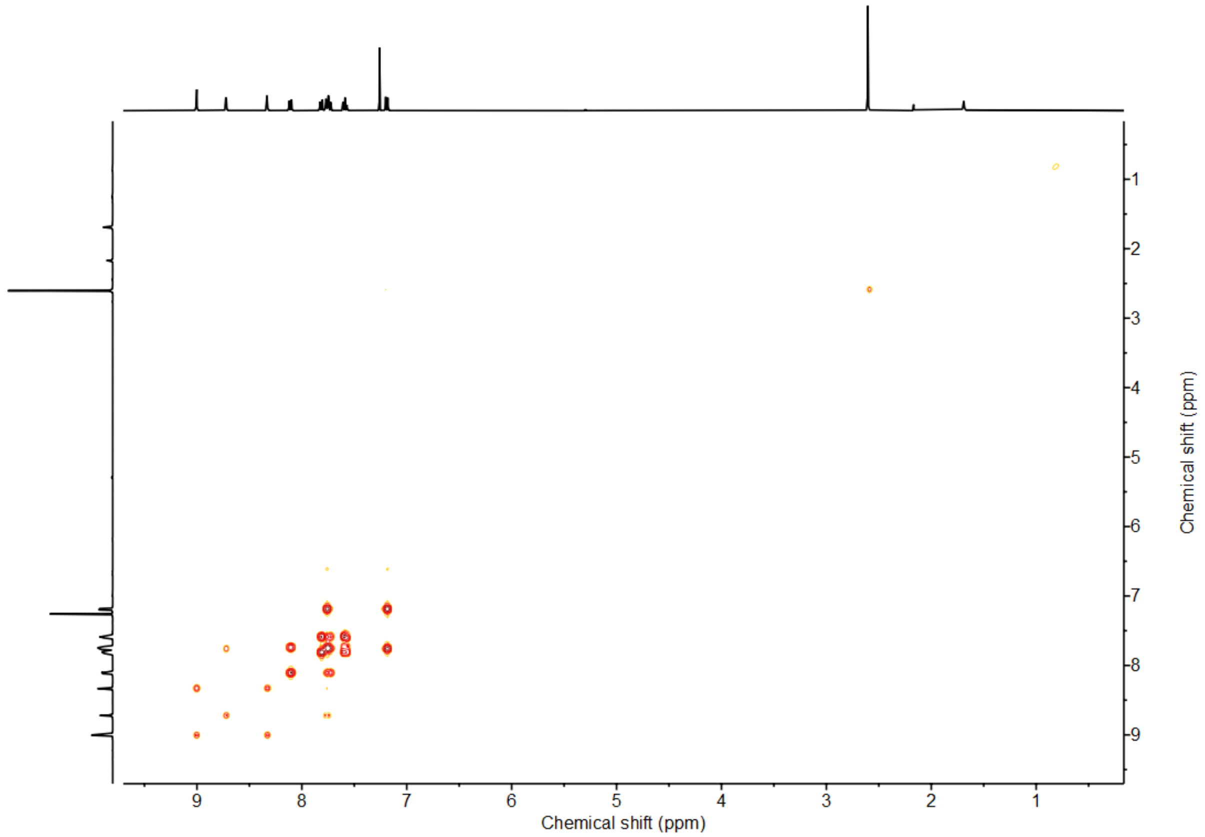

Figure S20 COSY ($CDCl_3$) of 1BC.

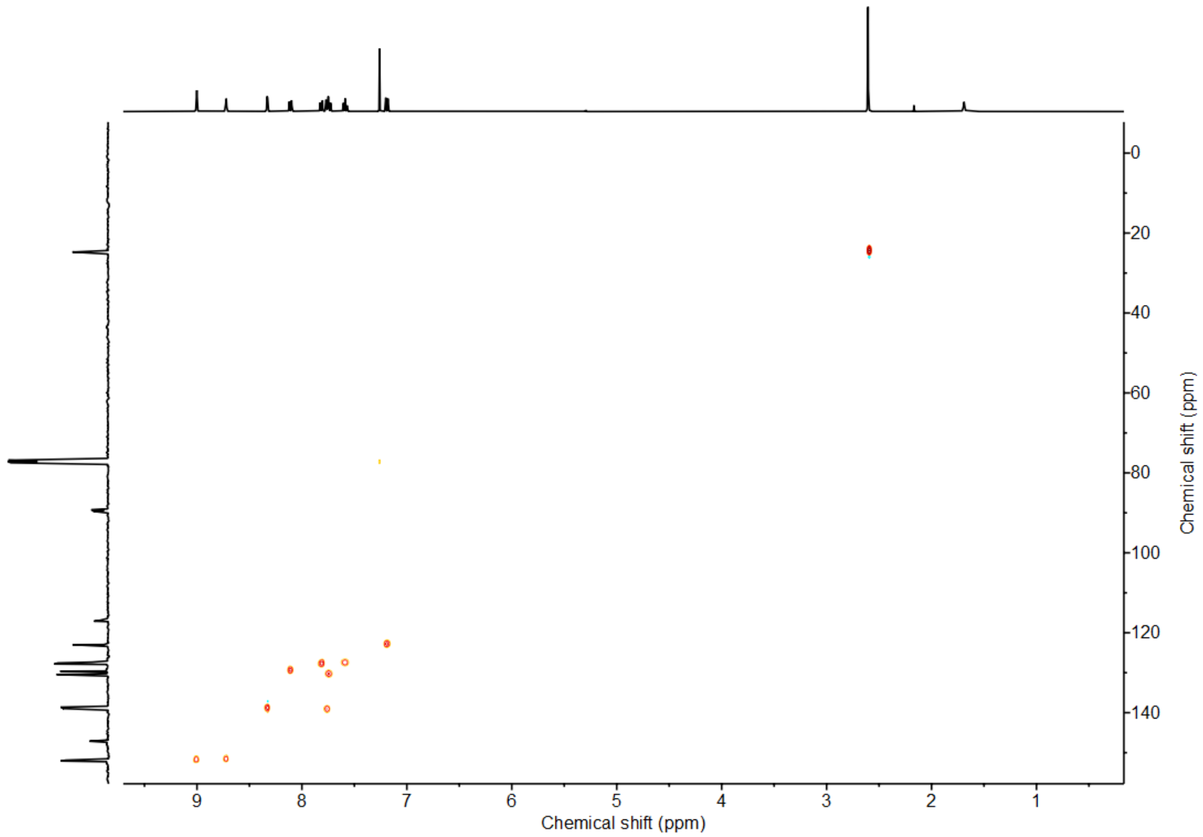

Figure S21 HSQC ($CDCl_3$) of 1BC.

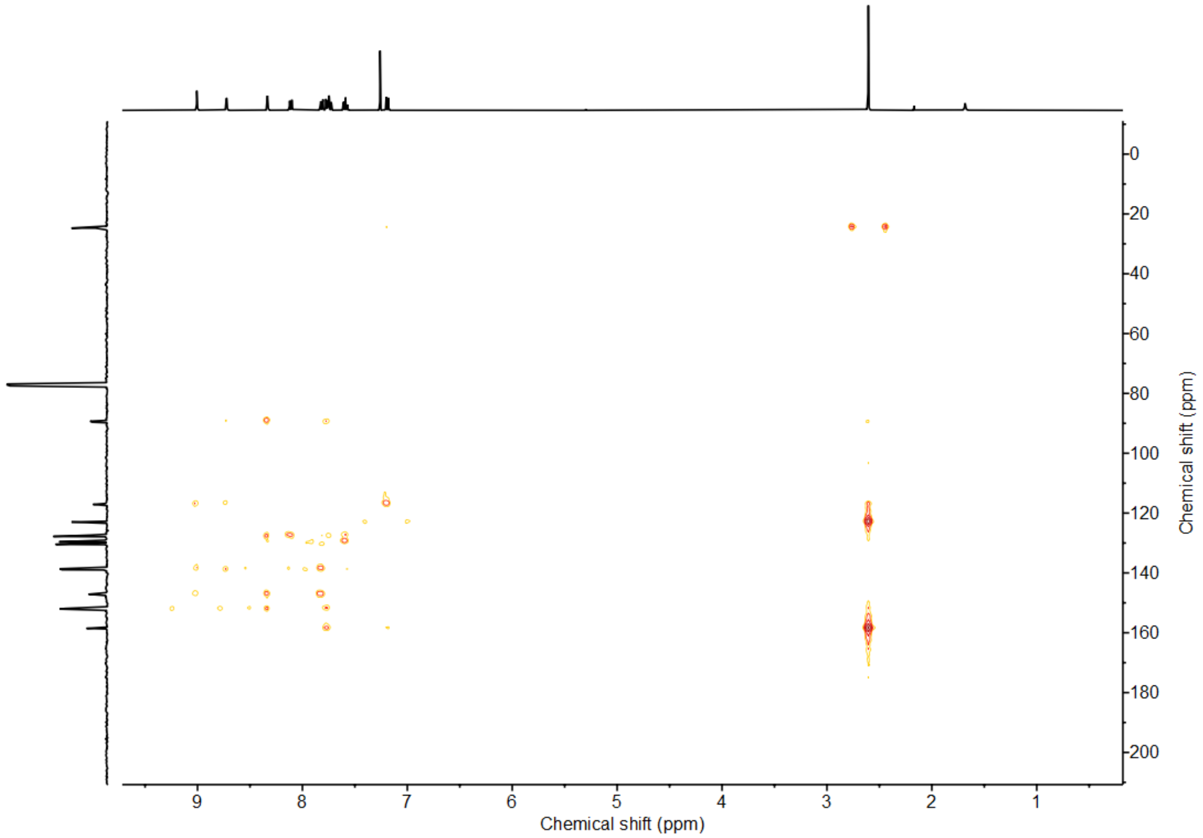

Figure S22 HMBC ($CDCl_3$) of 1BC.

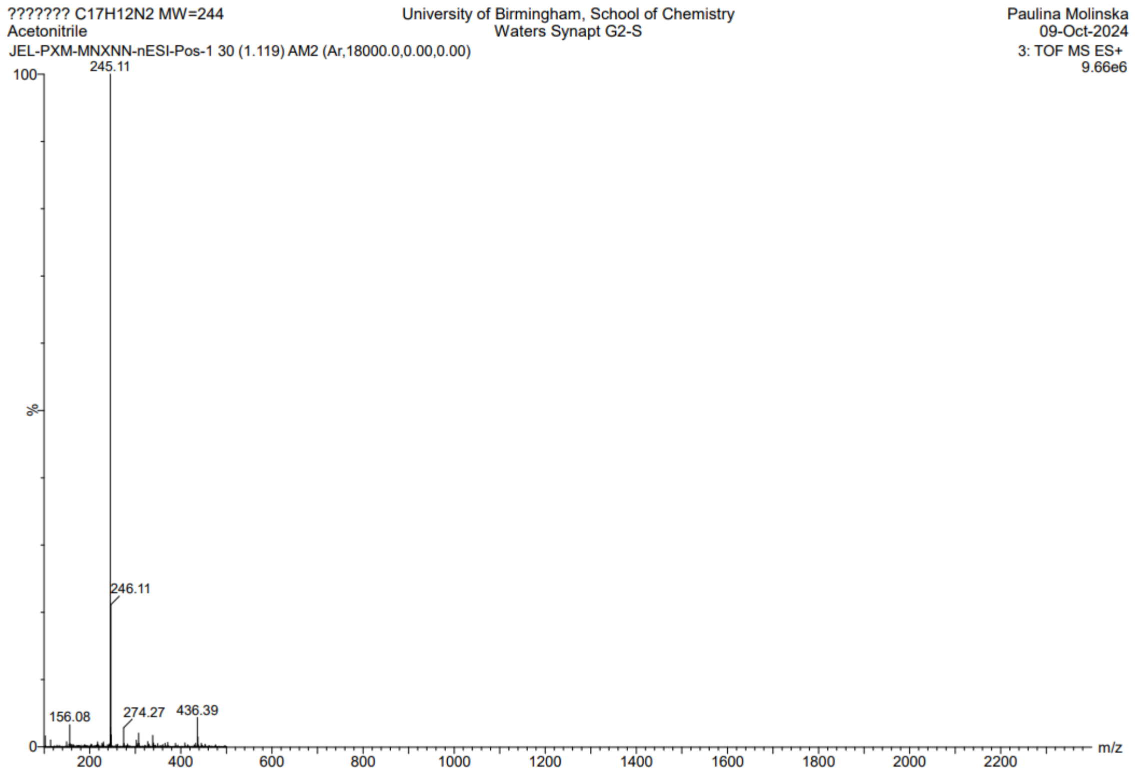

Figure S23 ESI-MS of 1BC.

Synthesis of 1AE

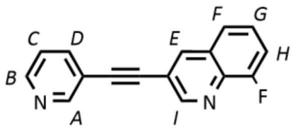

3-Bromo-8-fluoroquinoline (0.0565 g, 0.25 mmol, 1.0 eq.), 3-ethynylpyridine (0.0258 g, 0.25 mmol, 1.0 eq.) and Pd(dppf)$Cl_2 \cdot CH_2Cl_2$ (0.0113 g, 0.014 mmol, 5.5 mol%) were stirred at 80 °C in 1.0 M TBAF in THF (1 mL) in a sealed vial for 24 h. Brine (15 mL) was added to the cooled reaction mixture, and the aqueous phase subsequently extracted with Et_2O (3 × 20 mL). The combined organic phases were washed with 5% wt. aq. LiCl solution (2 x 25 mL), dried ($MgSO_4$) and the solvent removed *in vacuo*. After purification by column chromatography on silica gel (gradient 0 to 17% acetone in CH_2Cl_2) the product was obtained as a white solid (0.0401 g, 65%).

^{1}H NMR (400 MHz, $CDCl_3$) δ: 9.05 (d, *J* = 2.0 Hz, 1H, H_I), 8.84 (dd, *J* = 2.3, 0.9 Hz, 1H, H_A), 8.61 (dd, *J* = 4.9, 1.7 Hz, 1H, H_B), 8.37 (t, *J* = 1.8 Hz, 1H, H_E), 7.89 (ddd, *J* = 7.9, 2.2, 1.7 Hz, 1H, H_D), 7.63 (ddt, *J* = 8.2, 1.3, 0.5 Hz, 1H, H_F), 7.54 (td, *J* = 7.9, 4.8 Hz, 1H, H_G), 7.45 (ddd, *J* = 10.4, 7.7, 1.4 Hz, 1H, H_H), 7.34 (ddd, *J* = 7.9, 4.9, 0.9 Hz, 1H, H_C).

^{13}C NMR (101 MHz, $CDCl_3$) δ: 158.17 (d, *J* = 257.6 Hz), 152.55 (C_A), 152.17 (C_I), 149.43 (C_B), 138.79 (C_D), 138.43 (d, *J* = 2.9 Hz, C_E), 128.94, 128.92, 127.60 (d, *J* = 8.0 Hz, C_G), 123.47 (d, *J* = 4.8 Hz, C_F), 123.32 (C_C), 119.75, 118.05, 114.76 (d, *J* = 19.0 Hz, C_H), 90.08, 89.47.

^{19}F NMR (376 MHz, $CDCl_3$) δ: -125.04.

HR-ESI-MS *m/z* = 249.0831 $[M+H]^+$ calc. 249.0828 (Δ = 1.2 ppm).

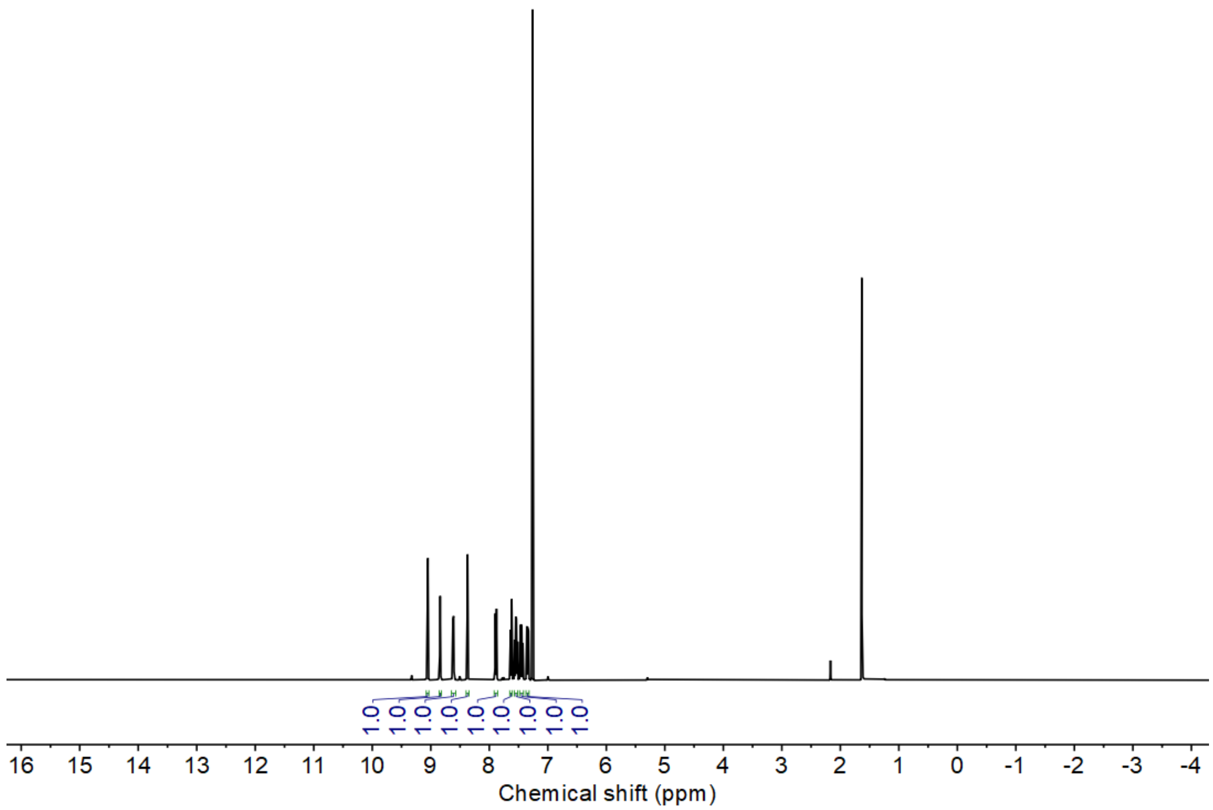

Figure S24 ^{1}H NMR (400 MHz, $CDCl_3$) of 1AE.

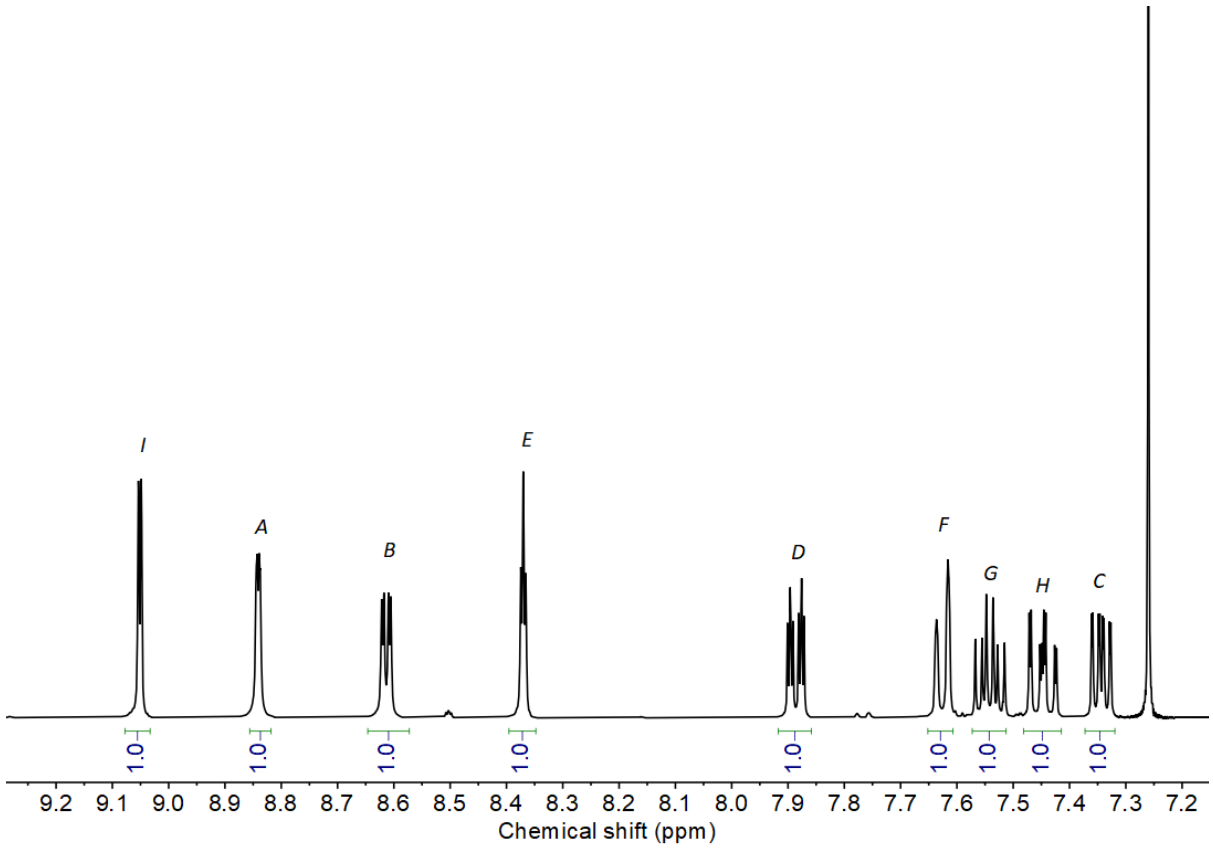

Figure S25 Partial ^{1}H NMR (400 MHz, $CDCl_3$) of 1AE.

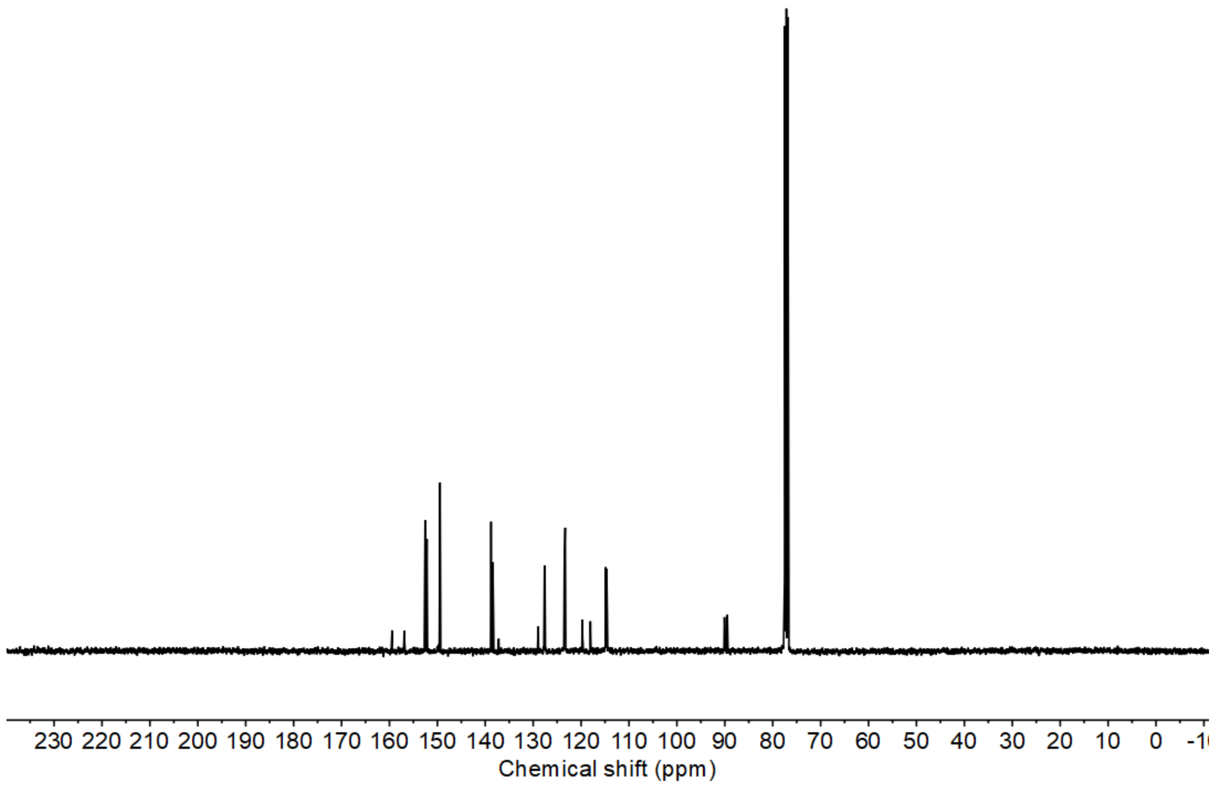

Figure S26 ^{13}C NMR (101 MHz, $CDCl_3$) of 1AE.

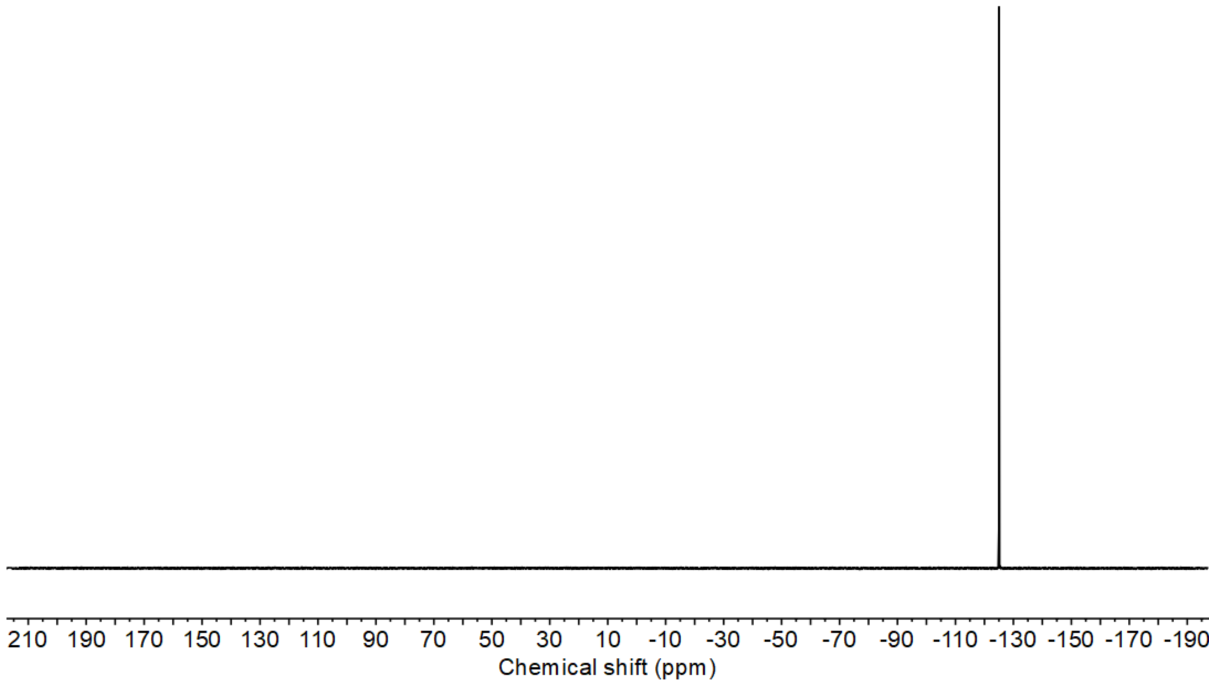

Figure S27 ^{19}F NMR (376 MHz, $CDCl_3$) of 1AE.

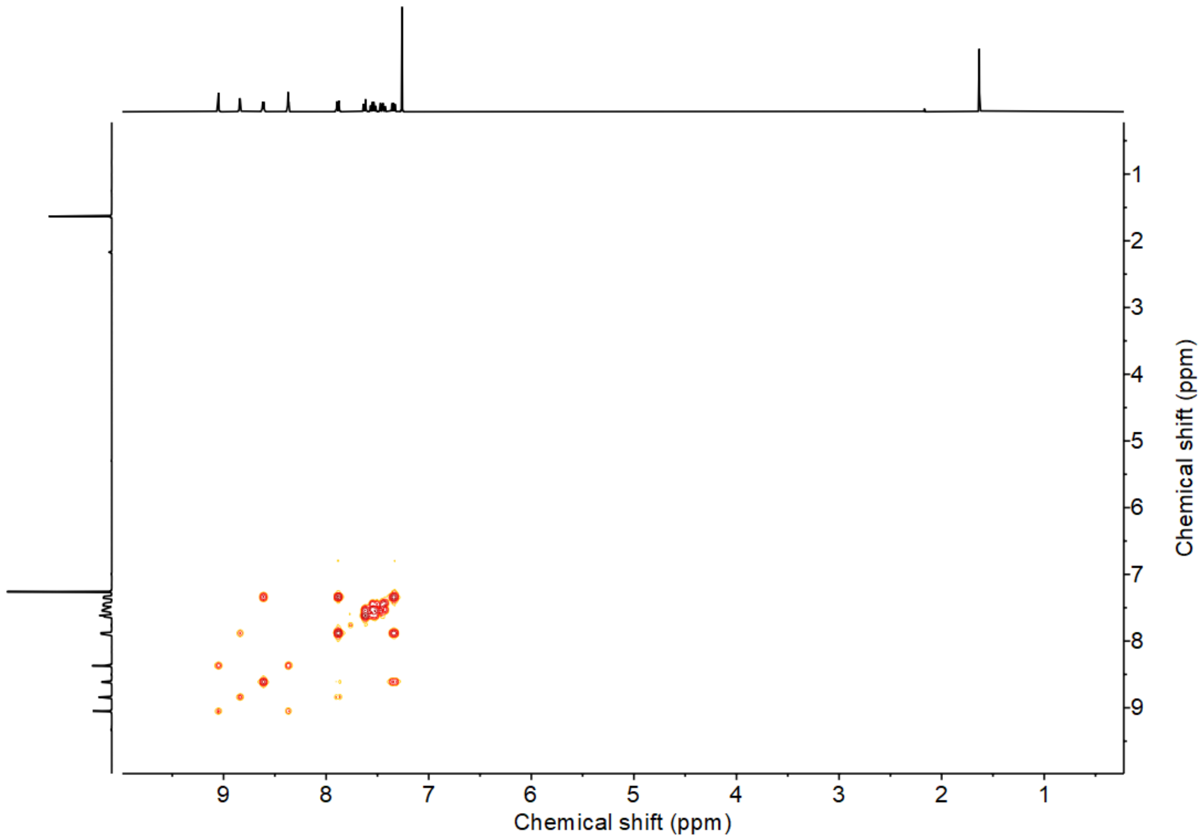

Figure S28 COSY ($CDCl_3$) of 1AE.

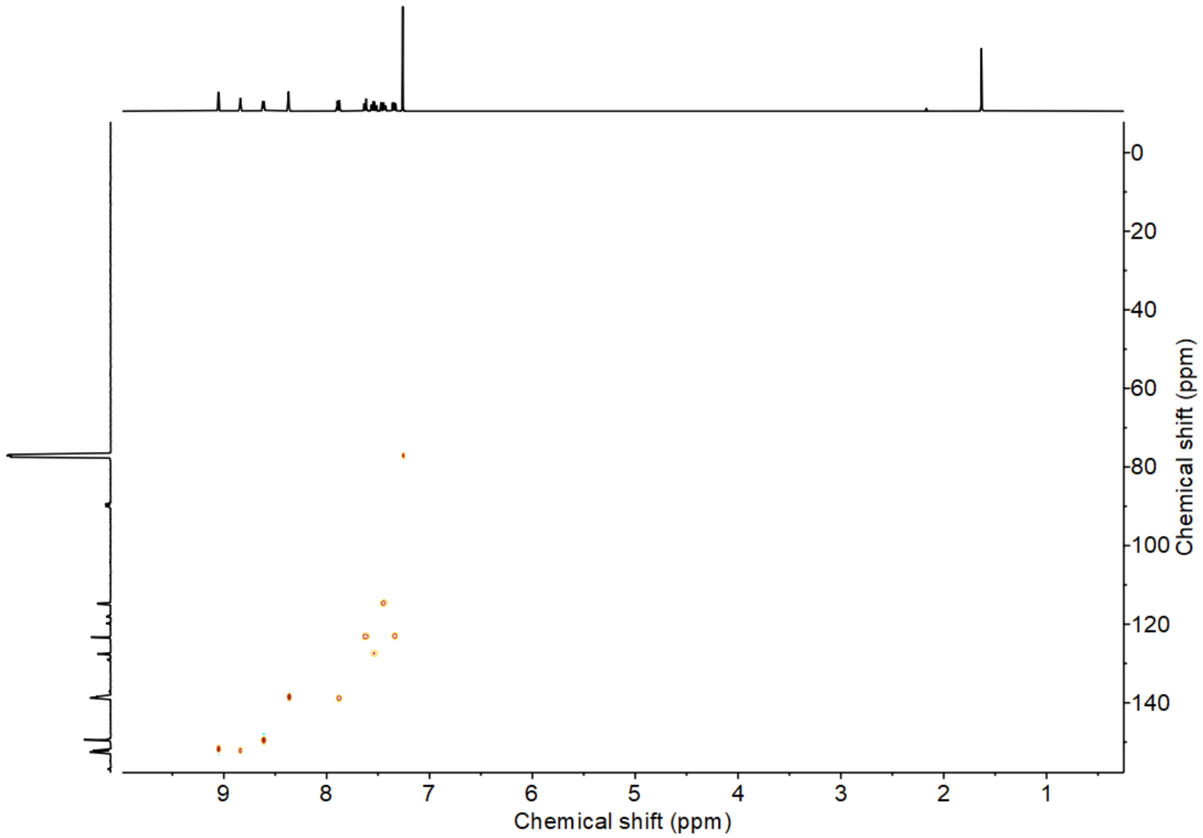

Figure S29 HSQC ($CDCl_3$) of 1AE.

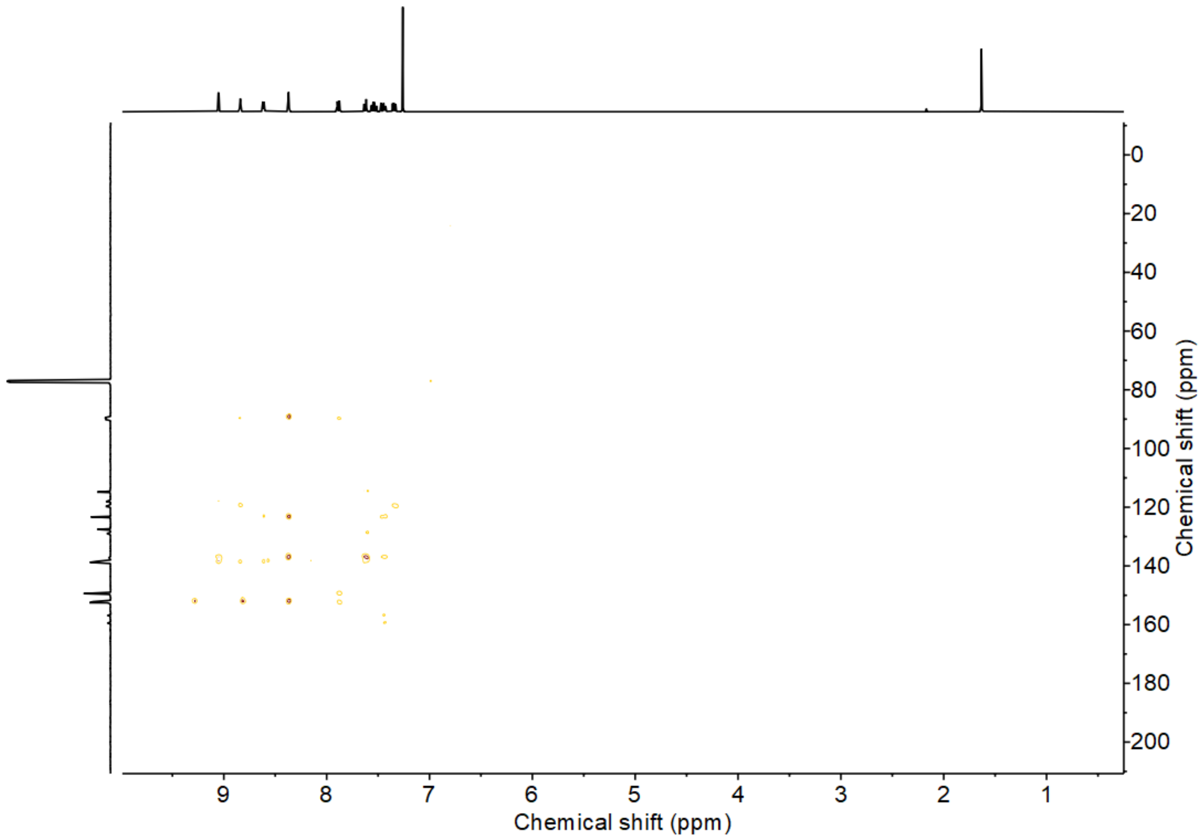

Figure S30 HMBC ($CDCl_3$) of 1AE.

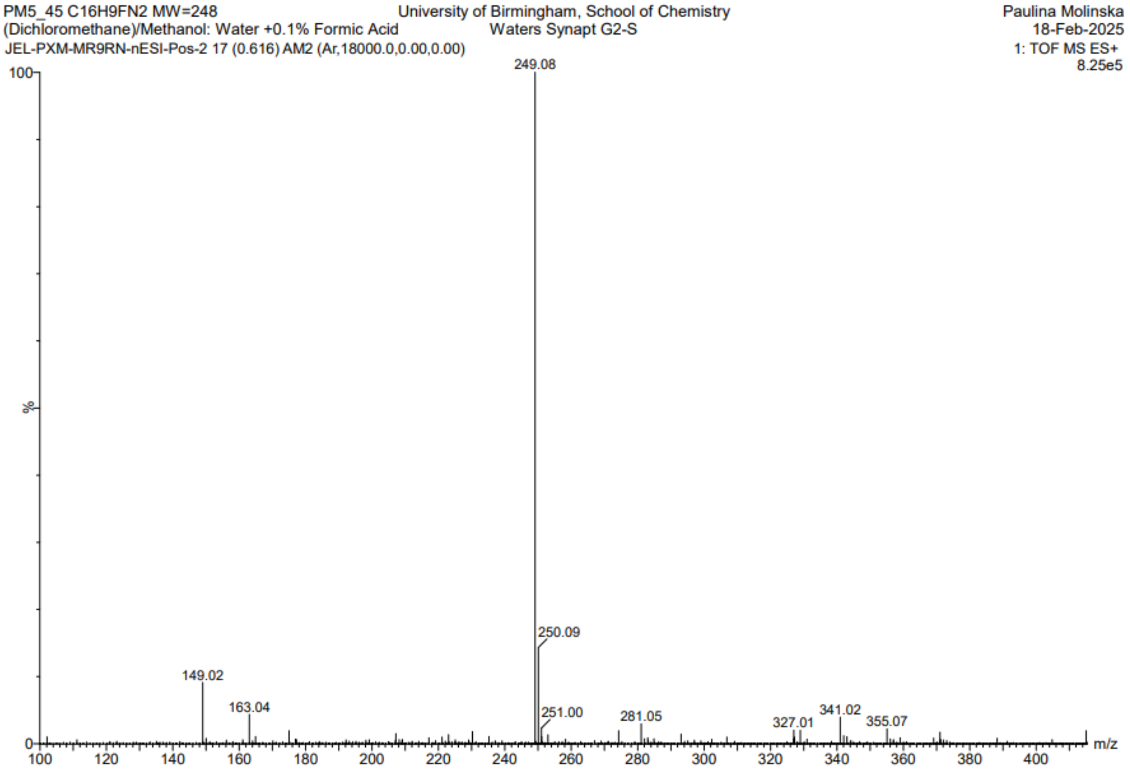

Figure S31 ESI-MS of 1AE.

Synthesis of 1BD

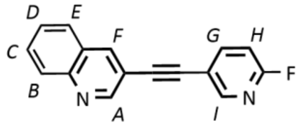

S1 (0.1136 g, 0.50 mmol, 1.0 eq.), 5-bromo-2-fluoropyridine (0.0939 g, 0.54 mmol, 1.1 eq.) and Pd(dppf)$Cl_2 \cdot CH_2Cl_2$ (0.0215 g, 0.026 mmol, 5.2 mol%) were stirred at 80 °C in 1.0 M TBAF in THF (2.5 mL) in a sealed vial for 24 h. Brine (20 mL) was added to the cooled reaction mixture, and the aqueous phase subsequently extracted with Et_2O (3 × 20 mL). The combined organic phases were washed with 5% wt. aq. LiCl solution (2 x 20 mL), dried ($MgSO_4$) and the solvent removed *in vacuo*. After purification by column chromatography on silica gel (gradient 0 to 6% acetone in CH_2Cl_2) the product was obtained as a beige crystalline solid that solidified upon standing (0.0543 g, 44%).

^{1}H NMR (400 MHz, $CDCl_3$) δ: 9.00 (H_A, d, J = 2.1 Hz, 1H), 8.46 (H_I, d, J = 2.1 Hz, 1H), 8.34 (H_F, d, J = 1.8 Hz, 1H), 8.12 (H_B, dd, J = 8.4, 1.0 Hz, 1H), 7.97 (H_G, ddd, J = 8.6, 7.5, 2.4 Hz, 1H), 7.82 (H_E, dd, J = 8.2, 1.4 Hz, 1H), 7.76 (H_C, ddd, J = 8.5, 6.9, 1.5 Hz, 1H), 7.60 (H_D, ddd, J = 8.1, 6.9, 1.2 Hz, 1H), 6.99 (H_H, ddd, J = 8.7, 3.0, 0.7 Hz, 1H).

^{13}C NMR (101 MHz, $CDCl_3$) δ: 163.00 (d, J = 243.1 Hz), 151.92 (C_A), 150.9 (d, J = 15.4 Hz, C_I), 147.25, 143.87 (d, J = 8.4 Hz, C_G), 138.81 (C_F), 130.64 (C_C), 129.64 (C_B), 127.84 (C_D/C_E), 127.66 (C_D/C_E), 127.27, 117.87 (d, J = 5.1 Hz), 116.64, 109.78 (d, J = 38.0 Hz, H_H), 89.83, 87.88.

^{19}F NMR (376 MHz, $CDCl_3$) δ: -65.17.

HR-ESI-MS *m/z* = 249.0834 $[M+H]^+$ calc. 249.0835 (Δ = 0.40 ppm).

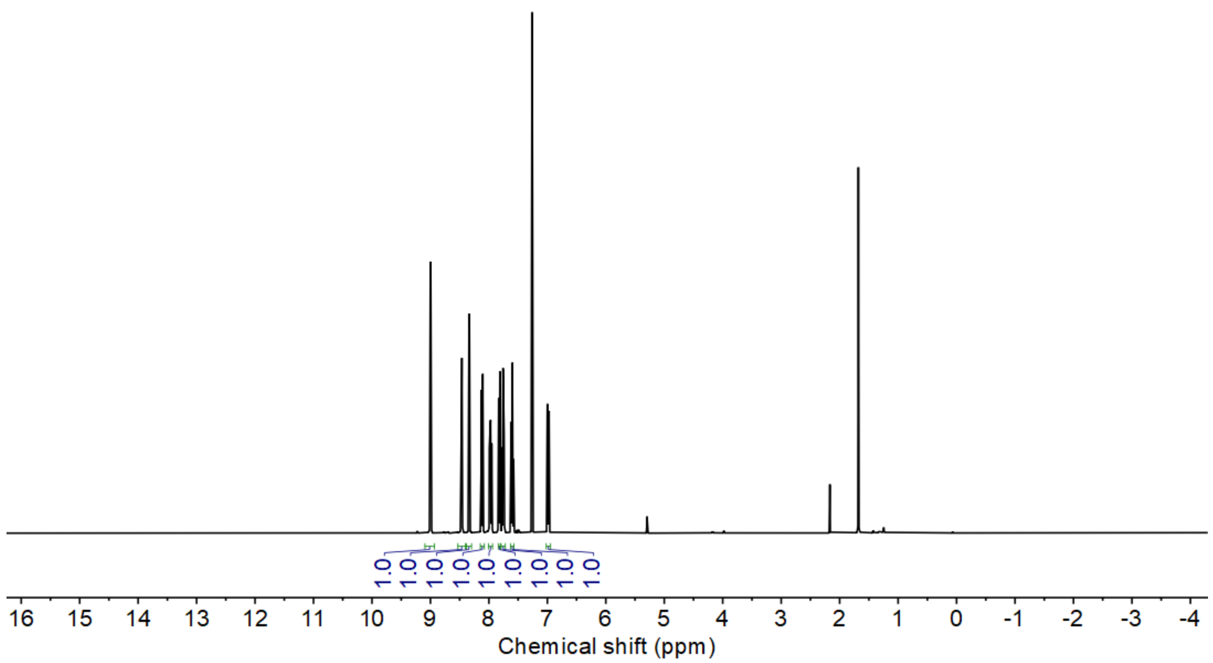

Figure S32 ^{1}H NMR (400 MHz, $CDCl_3$) of 1BD.

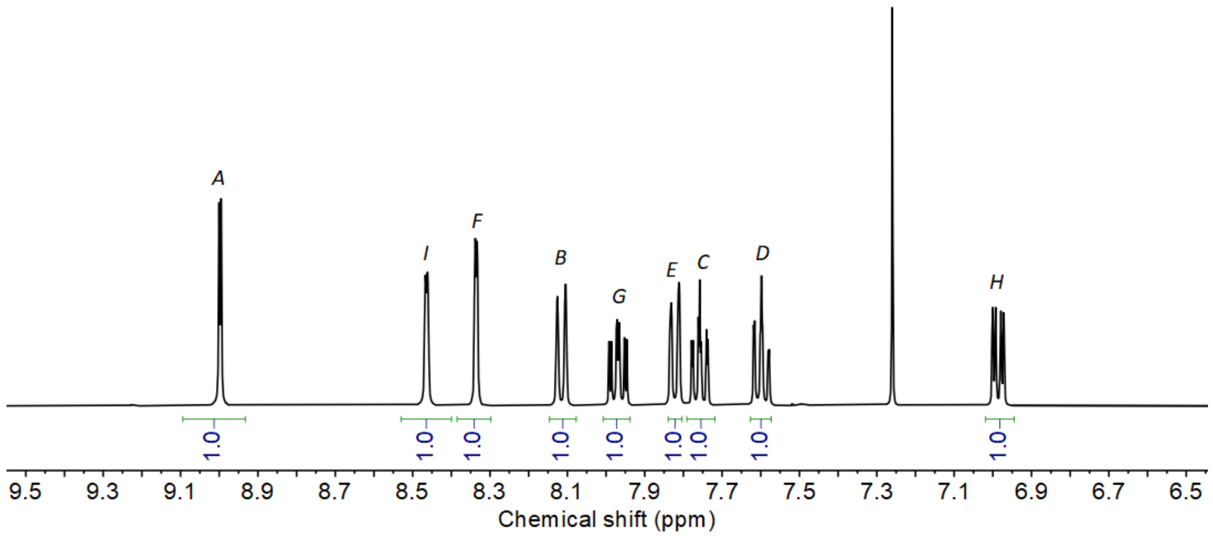

Figure S33 Partial ^{1}H NMR (400 MHz, $CDCl_3$) of 1BD.

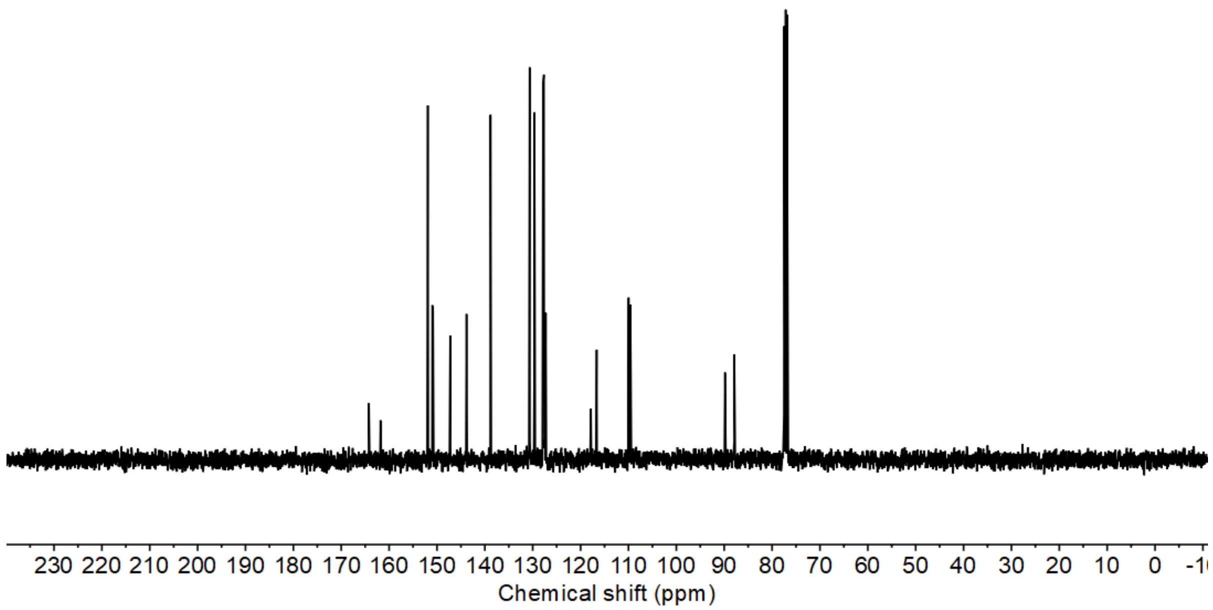

Figure S34 ^{13}C NMR (101 MHz, $CDCl_3$) of 1BD.

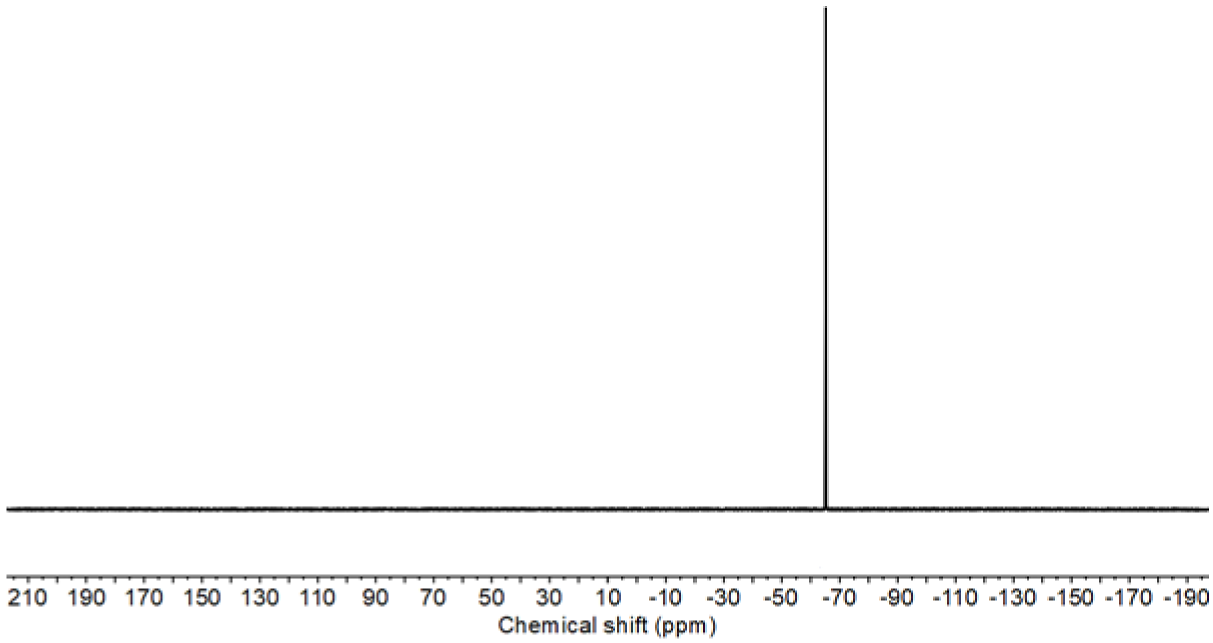

Figure S35 ^{19}F NMR (376 MHz, $CDCl_3$) of 1BD.

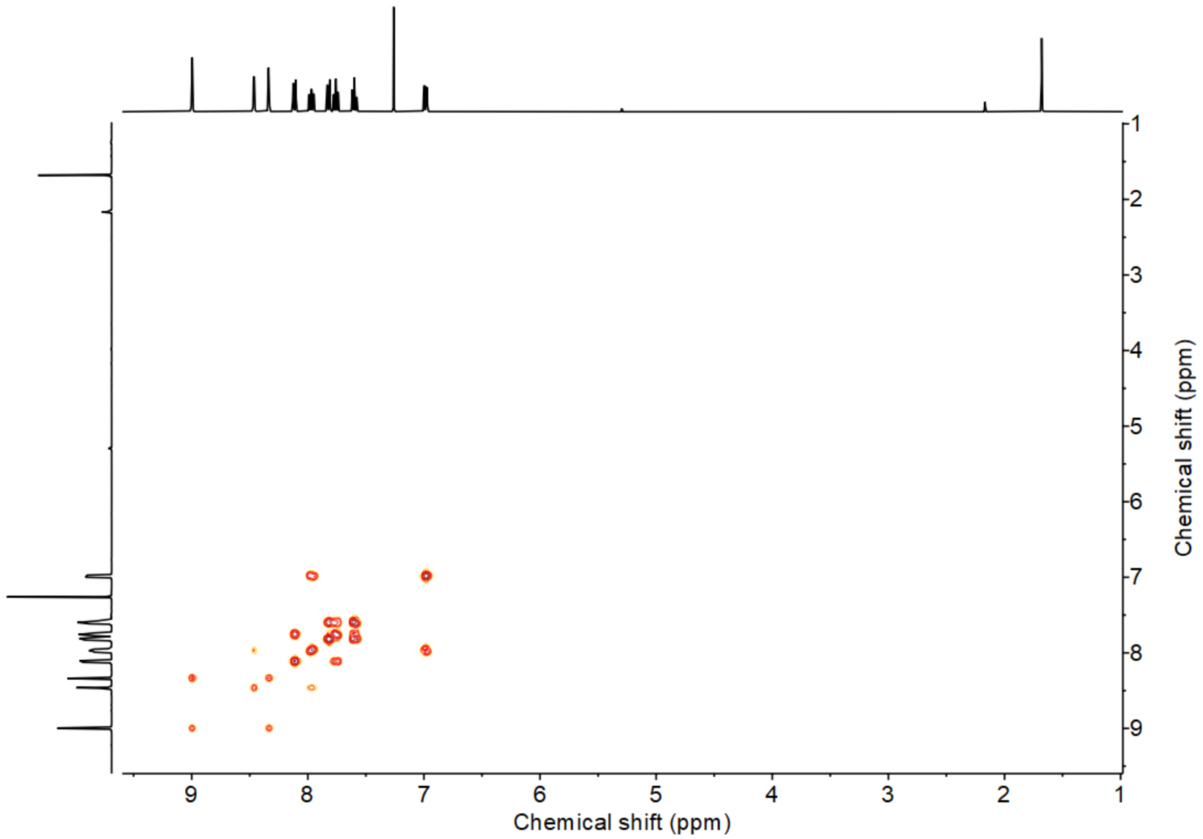

Figure S36 COSY (CDCl$_3$) of 1BD.

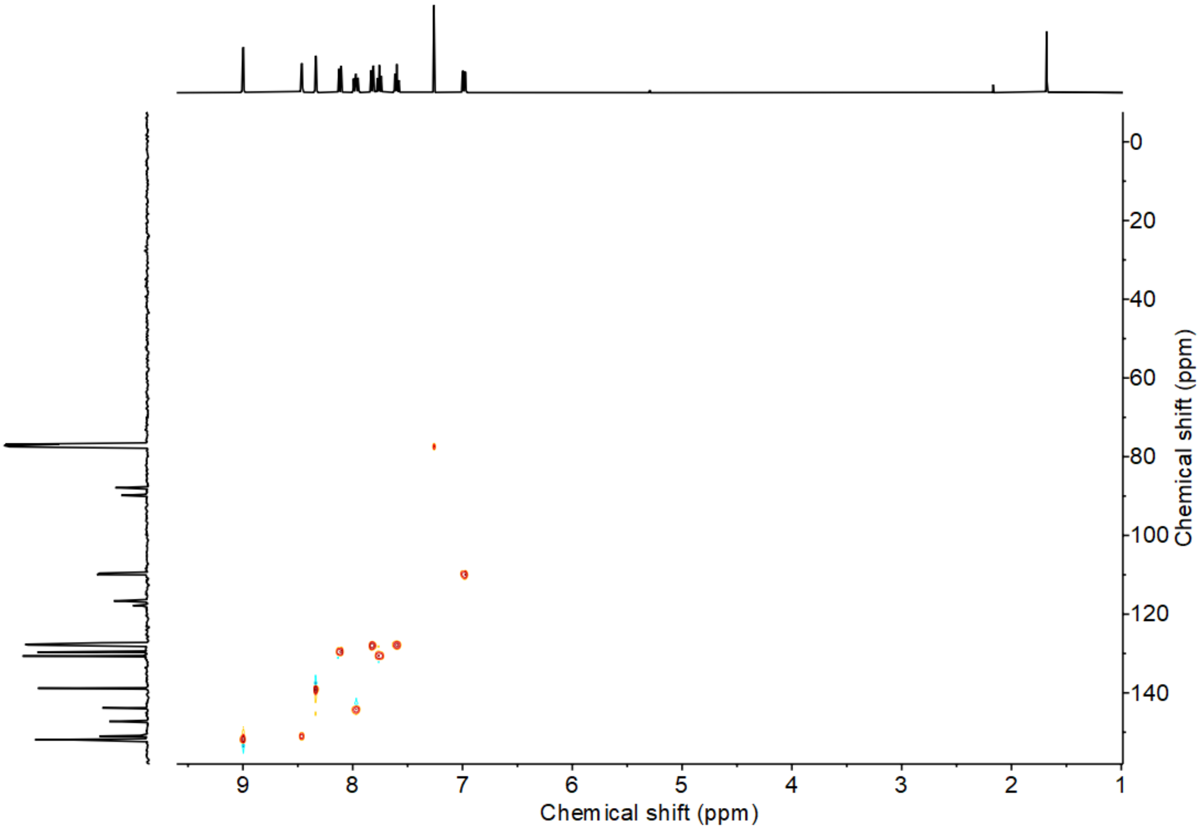

Figure S37 HSQC (CDCl$_3$) of 1BD.

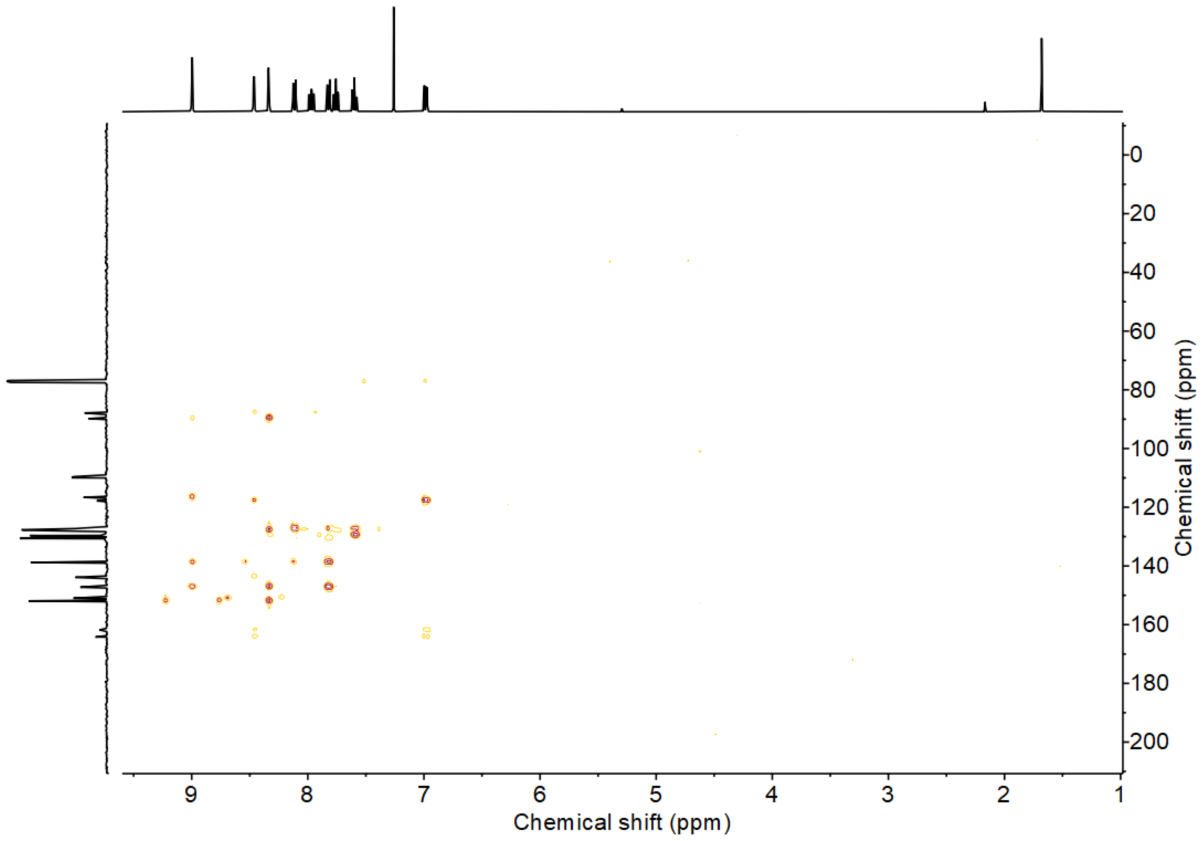

Figure S38 HMBC ($CDCl_3$) of 1BD.

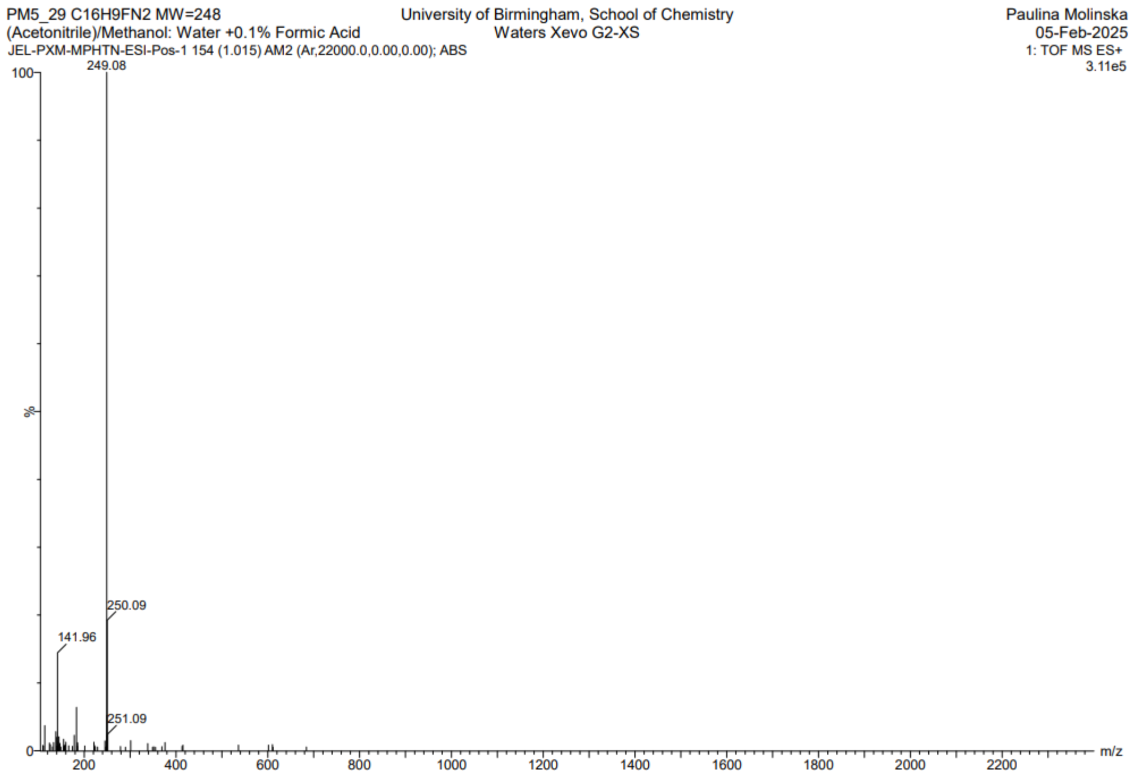

Figure S39 ESI-MS of 1BD.

Synthesis of 1CE

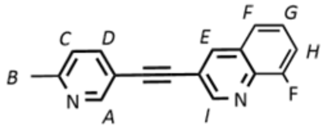

S1 (0.0688 g, 0.25 mmol, 1.0 eq.), 3-bromo-8-fluoropyridine (0.0588 g, 0.26 mmol, 1.0 eq.) and Pd(dppf)$Cl_2 \cdot CH_2Cl_2$ (0.0114 g, 0.014 mmol, 5.6 mol%) were stirred at 80 °C in 1.0 M TBAF in THF (2 mL) in a sealed vial for 16 h. Brine (15 mL) was added to the cooled reaction mixture, and the aqueous phase subsequently extracted with Et_2O (3 × 15 mL). The combined organic phases were washed with 5% wt. aq. LiCl solution (3 x 15 mL), dried ($MgSO_4$) and the solvent removed *in vacuo*. After purification by column chromatography on silica gel (gradient 0 to 36% acetone in CH_2Cl_2) the product was obtained as an off-white solid (0.0393 g, 60%).

^{1}H NMR (400 MHz, $CDCl_3$) δ: 9.04 (d, *J* = 1.6 Hz, 1H, H_I), 8.73 (s, 1H, H_A), 8.36 (s, 1H, H_E), 7.80 (d, *J* = 8.0, 1.8 Hz, 1H, H_D), 7.62 (d, *J* = 8.1 Hz, 1H, H_F), 7.53 (app. td, *J* = 7.9, 4.8 Hz, 1H, H_G), 7.44 (ddd, *J* = 10.3, 7.7, 1.2 Hz, 1H, H_H), 7.22 (d, *J* = 8.0 Hz, 1H, H_C), 2.63 (s, 3H, H_B).

^{13}C NMR (101 MHz, $CDCl_3$) δ: 158.50, 158.18 (d, *J* = 257.4 Hz), 156.90, 152.20 (C_I), 151.47 (C_A), 139.44 (C_D), 138.31 (d, *J* = 2.9 Hz, C_E), 128.96, 127.57 (d, *J* = 8.0 Hz C_G), 123.45 (d, *J* = 4.7 Hz, C_F), 123.29 (C_C), 118.19, 116.88, 114.68 (d, *J* = 19.0 Hz, C_H), 90.17, 89.07, 24.52 (C_B).

^{19}F NMR (376 MHz, $CDCl_3$) δ: -125.10.

HR-ESI-MS *m/z* = 263.0982 $[M+H]^+$ calc. 263.0984 (Δ = 0.76 ppm).

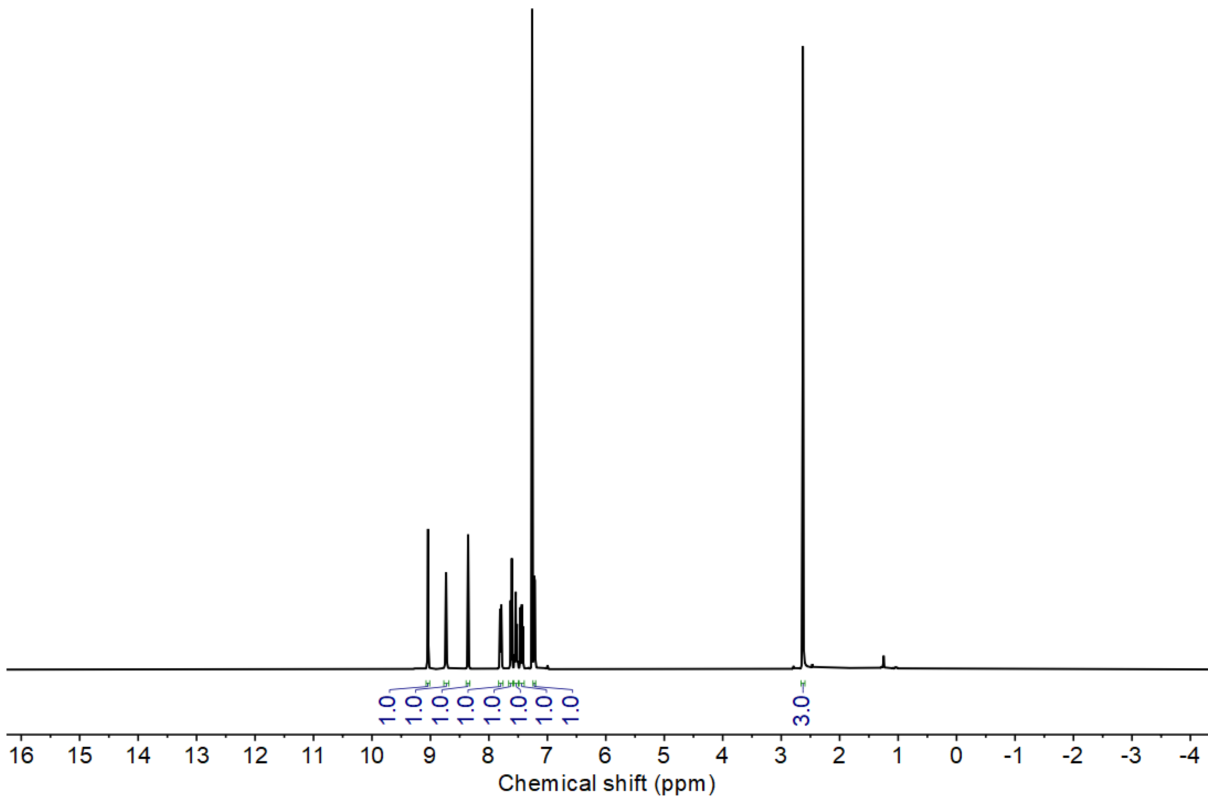

Figure S40 ^{1}H NMR (400 MHz, $CDCl_3$) of 1CE.

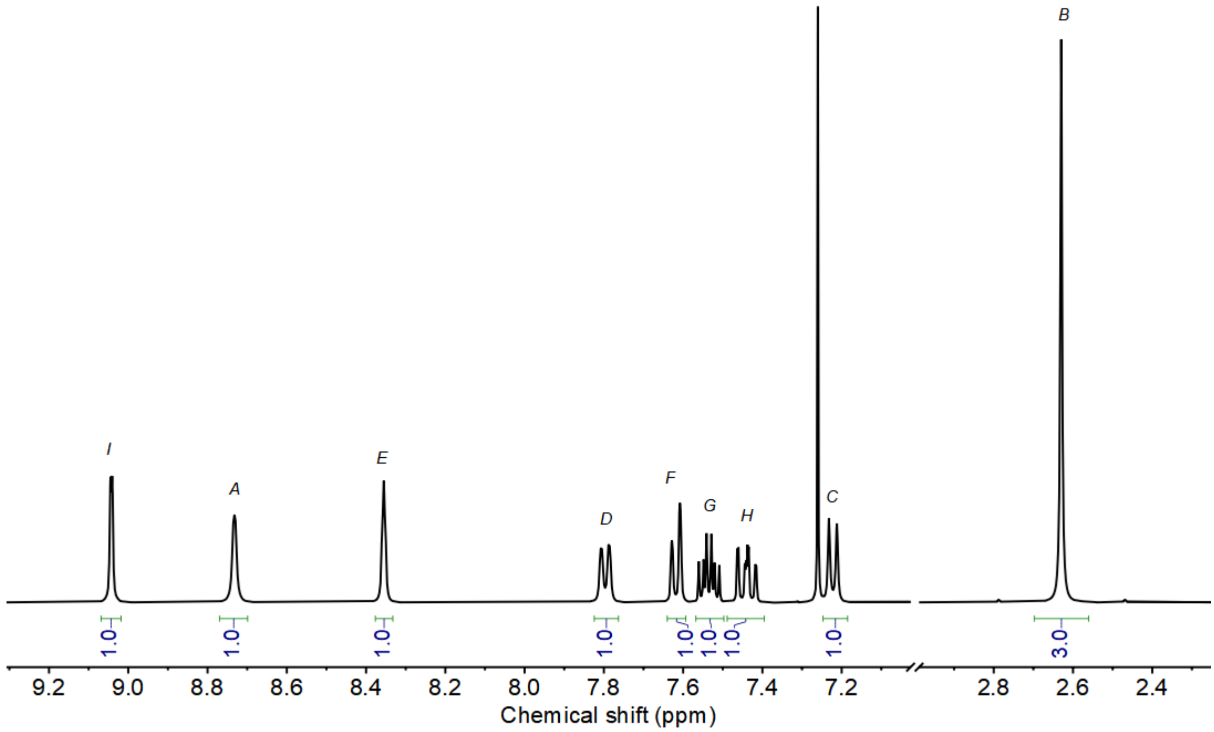

Figure S41 Partial ^{1}H NMR (400 MHz, $CDCl_3$) of 1CE.

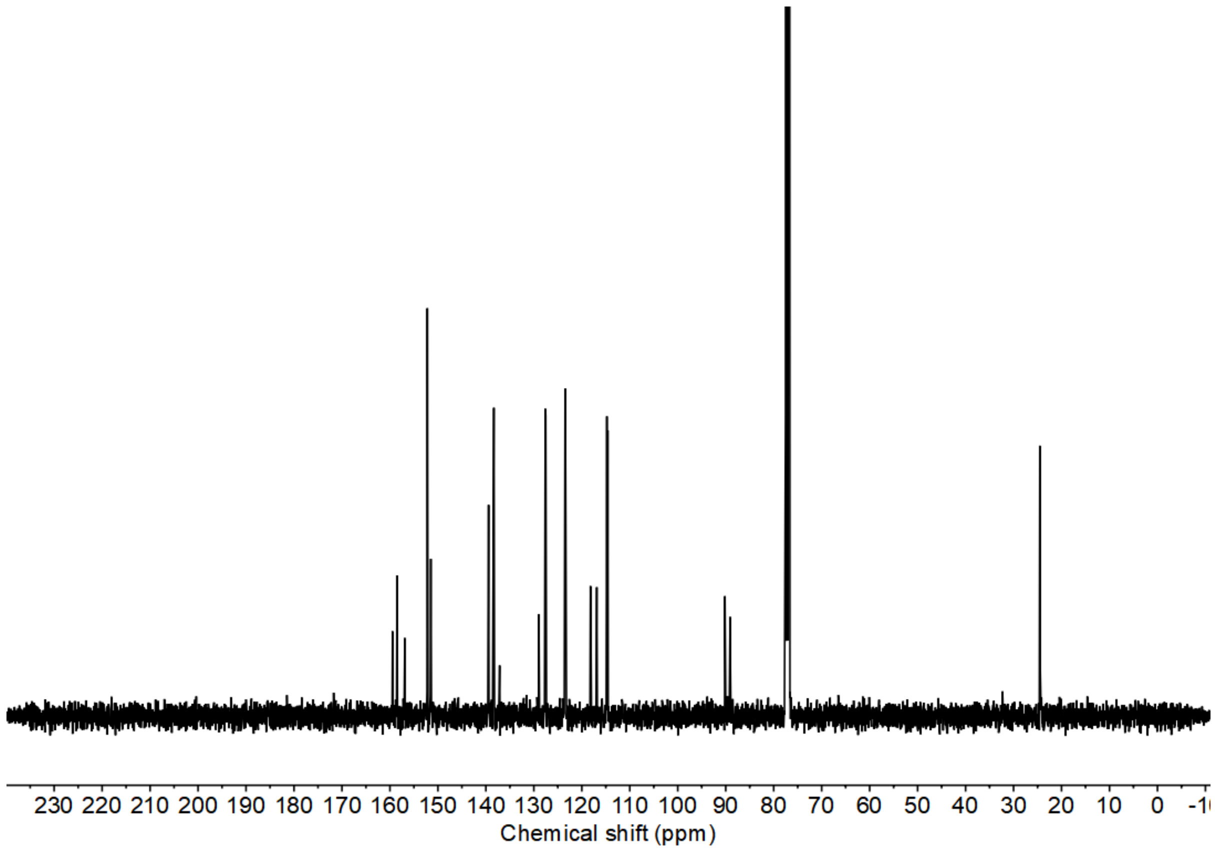

Figure S42 ^{13}C NMR (101 MHz, $CDCl_3$) of 1CE.

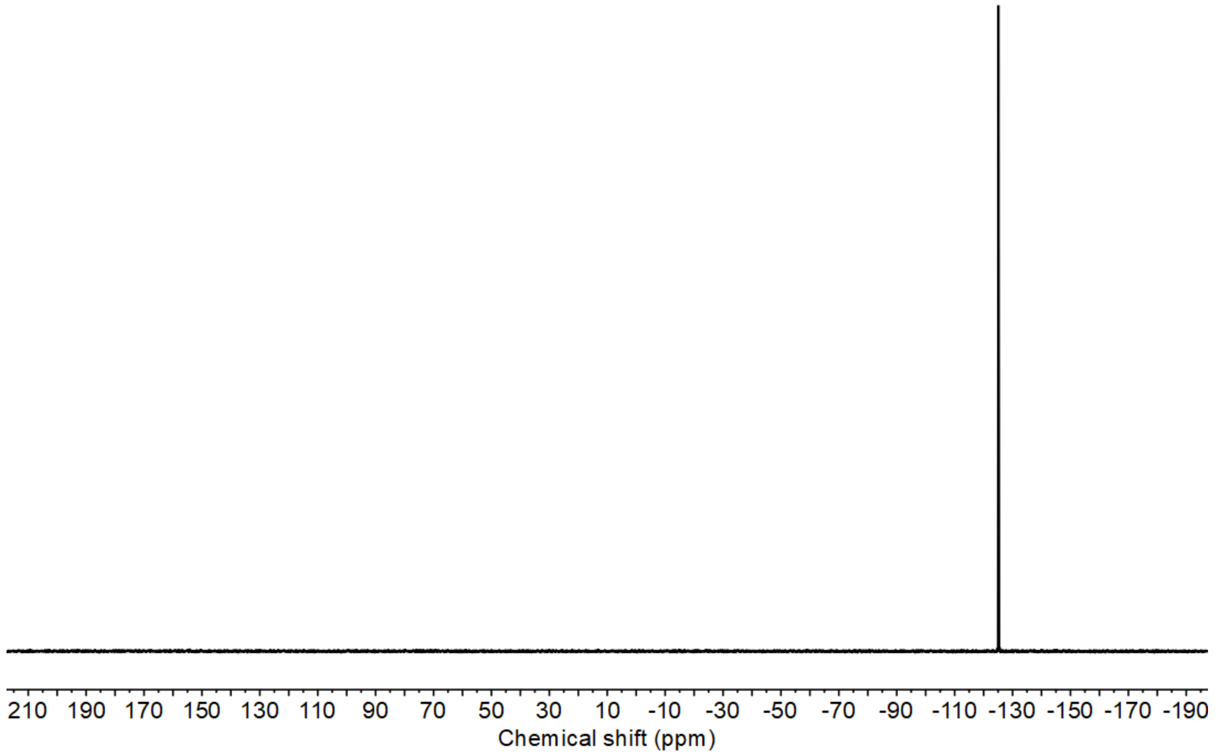

Figure S43 ^{19}F NMR (376 MHz, $CDCl_3$) of 1CE.

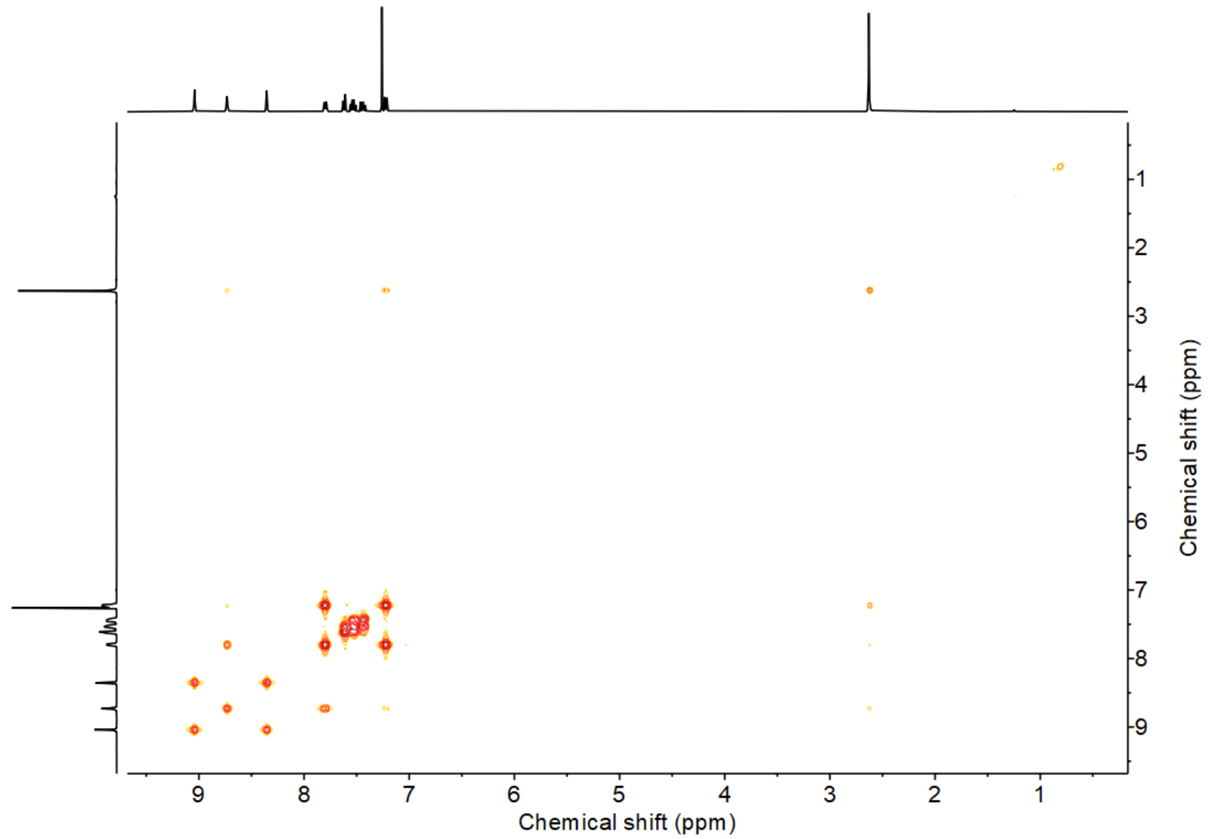

Figure S44 COSY ($CDCl_3$) of 1CE.

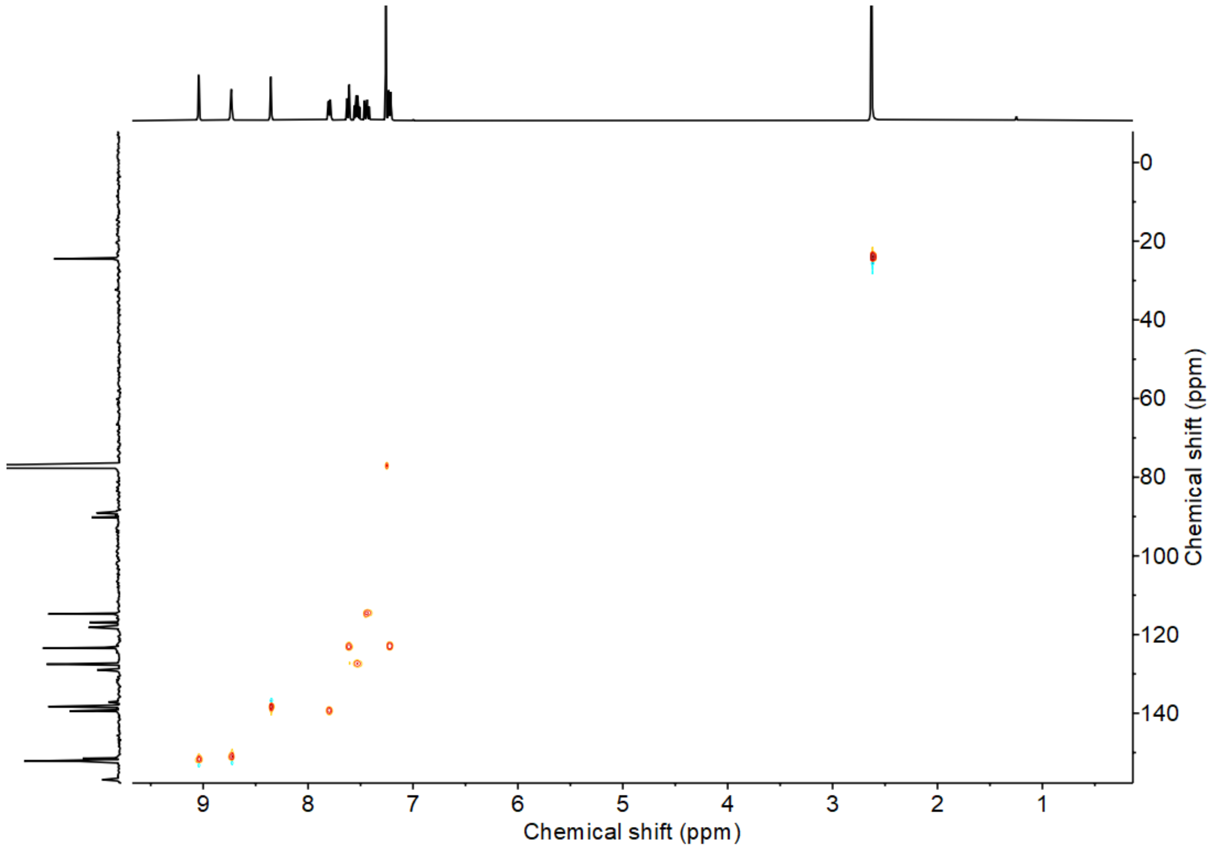

Figure S45 HSQC ($CDCl_3$) of 1CE.

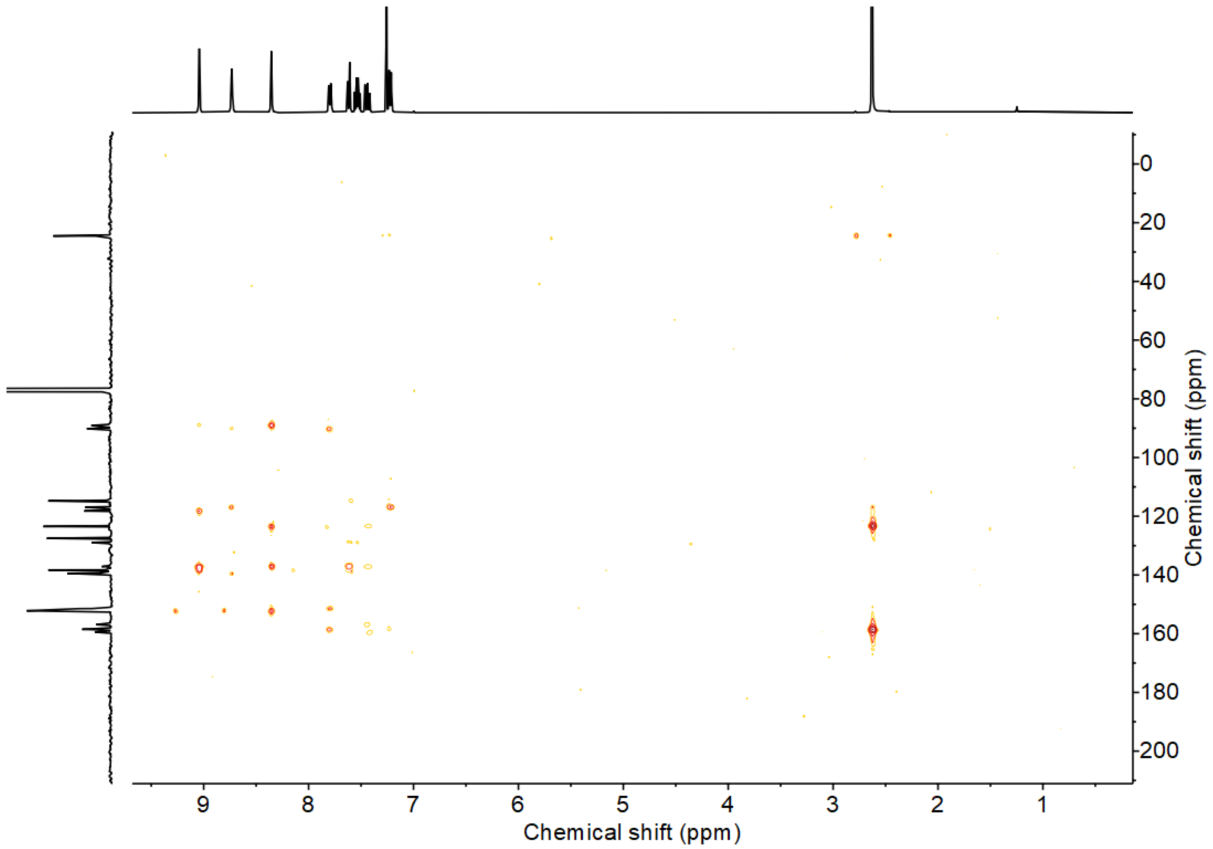

Figure S46 HMBC ($CDCl_3$) of 1CE.

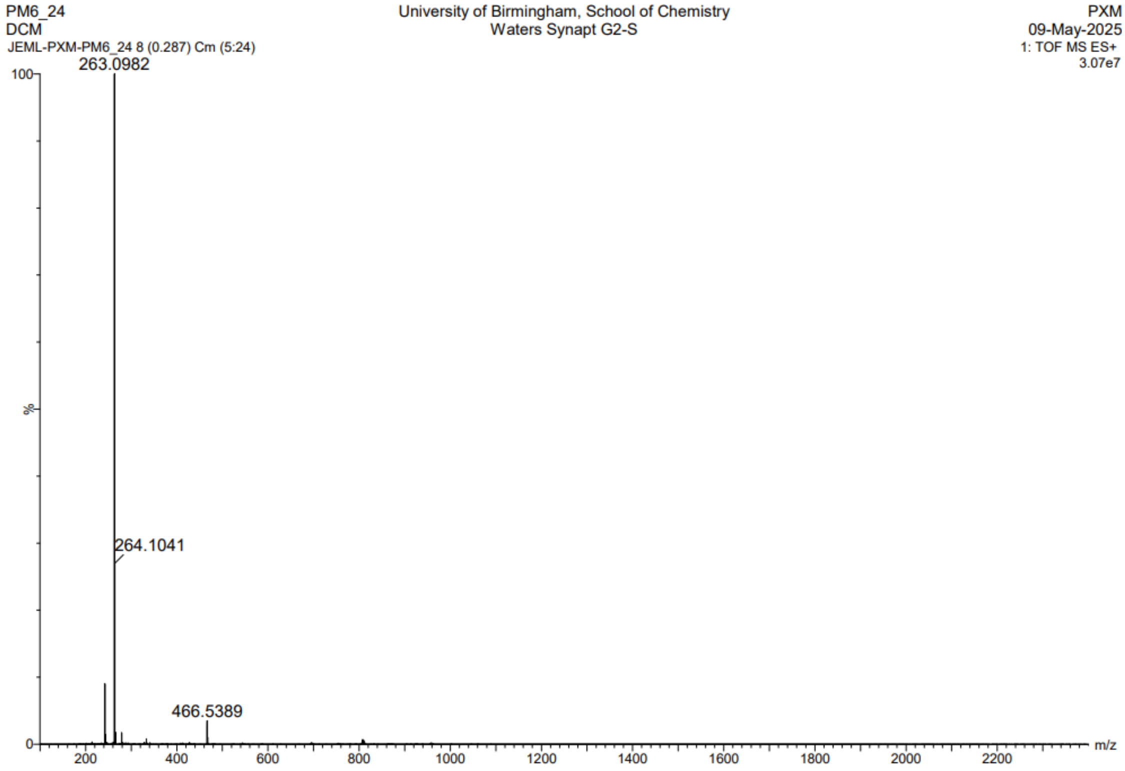

Figure S47 ESI-MS of 1CE.

Synthesis of 2AA

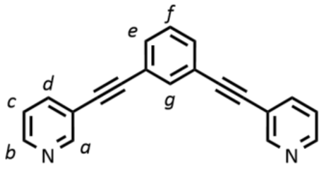

1,3-Diiodobenzene (0.321 g, 1.0 mmol, 1.0 eq.), 3-ethynylpyridine (0.208 g, 2.0 mmol, 2.0 eq.) and Pd(dppf)Cl_2·CH_2Cl_2 (0.040 g, 0.05 mmol, 5 mol%) were stirred at 80 °C in 1.0 M TBAF in THF (5 mL) in a sealed vial for 24 h. Brine (30 mL) was added to the cooled reaction mixture, and the aqueous phase subsequently extracted with Et_2O (3 × 30 mL). The combined organic phases were washed with 5% wt. aq. LiCl solution (2 x 30 mL), dried ($MgSO_4$) and the solvent removed *in vacuo*. After purification by column chromatography on silica gel (gradient 0 to 29% acetone in CH_2Cl_2) the product was obtained as an off-white solid (0.169 g, 60%).

Spectroscopic data were consistent with a previous literature report.[S1]

^{1}H NMR (400 MHz, $CDCl_3$) δ: 8.79 (d, *J* = 1.4 Hz, 2H, H_a), 8.58 (dd, *J* = 5.0, 1.7 Hz, 2H, H_b), 7.82 (app. dt, *J* = 7.9, 1.9 Hz, 2H, H_d), 7.75 (td, *J* = 1.7, 0.6 Hz, 1H, H_g), 7.54 (m, 2H, H_e), 7.38 (m, 1H, H_f), 7.30 (ddd, *J* = 7.8, 4.9, 0.9 Hz, 2H, H_c).

^{13}C NMR (101 MHz, $CDCl_3$) δ: 152.32, 148.83, 138.49, 134.75, 131.85, 128.71, 123.13, 123.07, 120.19, 91.57, 86.75.

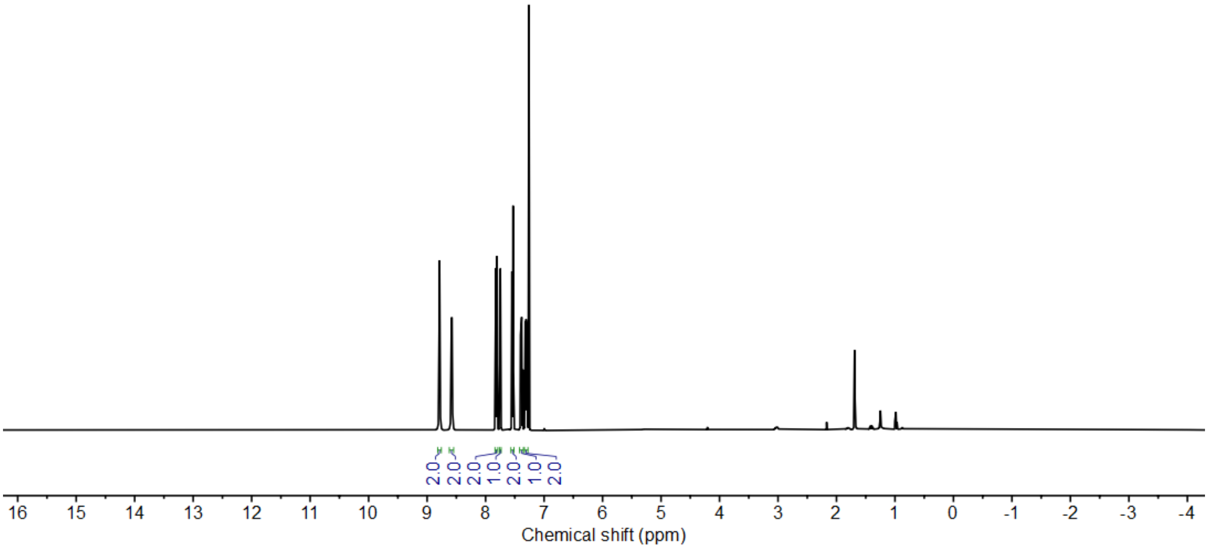

Figure S48 ^{1}H NMR (400 MHz, $CDCl_3$) of 2AA.

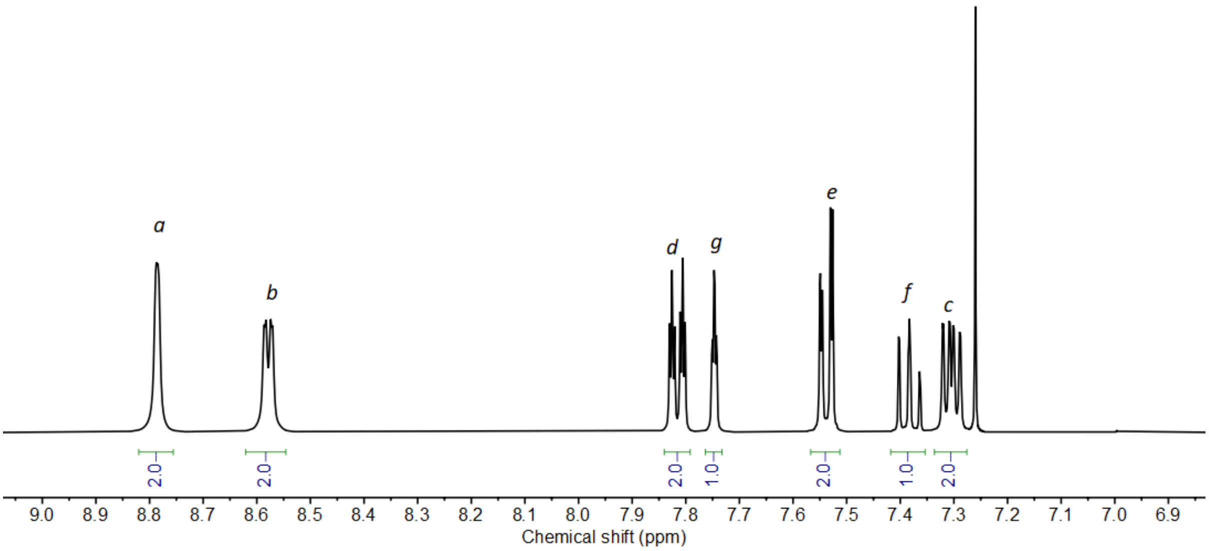

Figure S49 Partial ^{1}H NMR (400 MHz, $CDCl_3$) of 2AA.

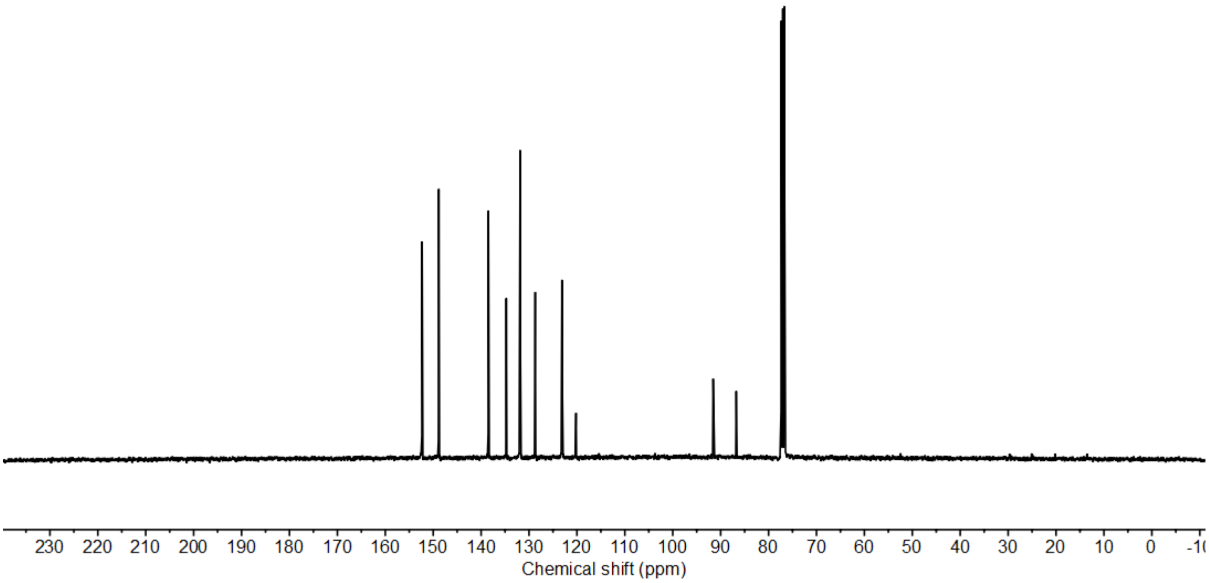

Figure S50 ^{13}C NMR (101 MHz, $CDCl_3$) of 2AA.

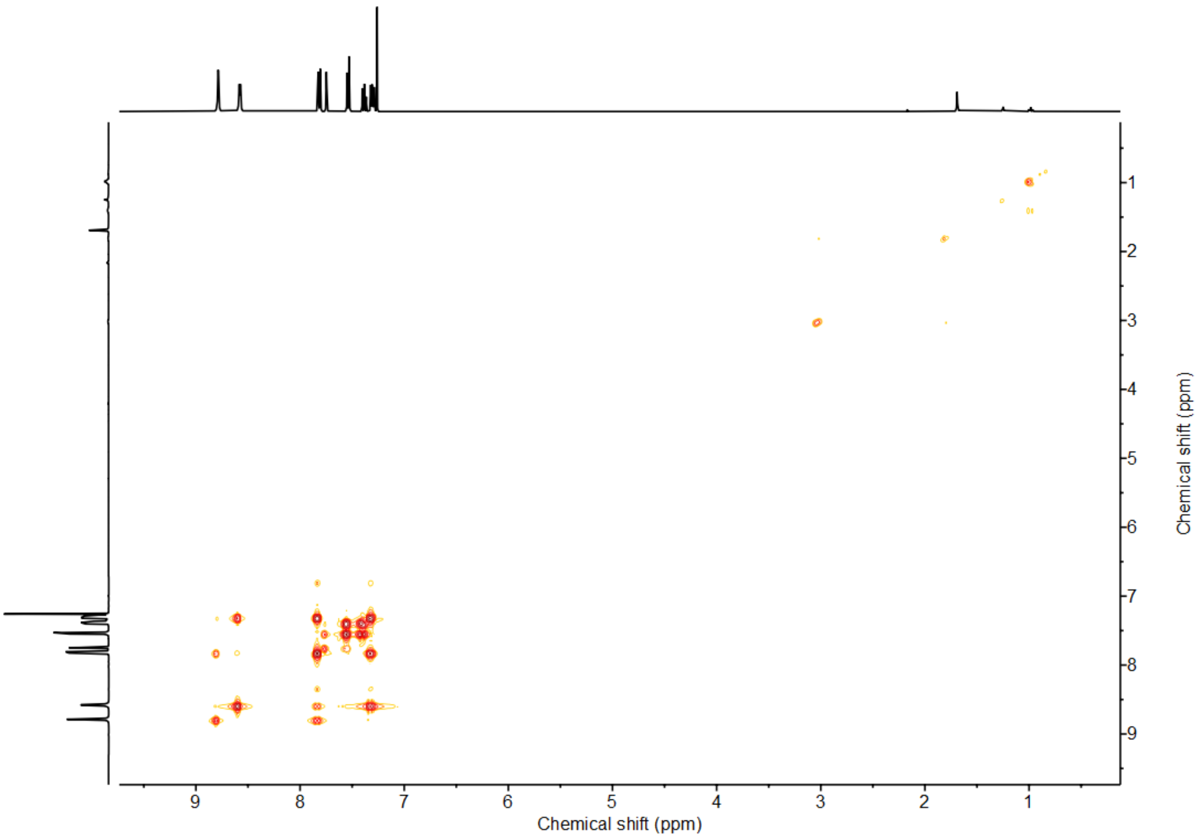

Figure S51 COSY ($CDCl_3$) of 2AA.

Synthesis of 3AA

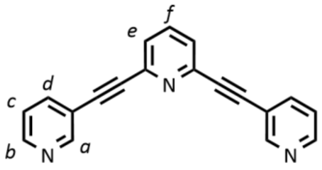

2,6-Dibromopyridine (0.236 g, 1.0 mmol, 1.0 eq.), 3-ethynylpyridine (0.207 g, 2.0 mmol, 2.0 eq.) and Pd(dppf)$Cl_2 \cdot CH_2Cl_2$ (0.041 g, 0.05 mmol, 5 mol%) were stirred at 80 °C in 1.0 M TBAF in THF (5 mL) in a sealed vial for 24 h. H_2O (20 mL) was added to the cooled reaction mixture, and the aqueous phase subsequently extracted with Et_2O (3 × 20 mL). The combined organic phases were dried ($MgSO_4$) and the solvent removed *in vacuo*. After purification by column chromatography on silica gel (step gradient 0 to 30% acetone in CH_2Cl_2 in 5% increments) the product was obtained as an off-white solid (0.128 g, 45%).

Spectroscopic data were consistent with a previous literature report.[S2]

^{1}H NMR (400 MHz, $CDCl_3$) δ: 8.83 (dd, *J* = 2.2, 0.9 Hz, 2H, H_a), 8.60 (dd, *J* = 4.9, 1.7 Hz, 2H,H_b), 7.88 (app. dt, *J* = 7.9, 1.9 Hz, 2H, H_d), 7.74 (app. dd, *J* = 8.2, 7.5 Hz, 1H, H_f), 7.54 (d, *J* = 7.8 Hz, 2H, H_e), 7.31 (ddd, *J* = 7.9, 4.9, 0.9 Hz, 2H, H_c).

^{13}C NMR (101 MHz, $CDCl_3$) δ: 154.11, 149.45, 144.15, 139.35, 135.52, 125.11, 122.53, 115.15, 89.25, 86.27.

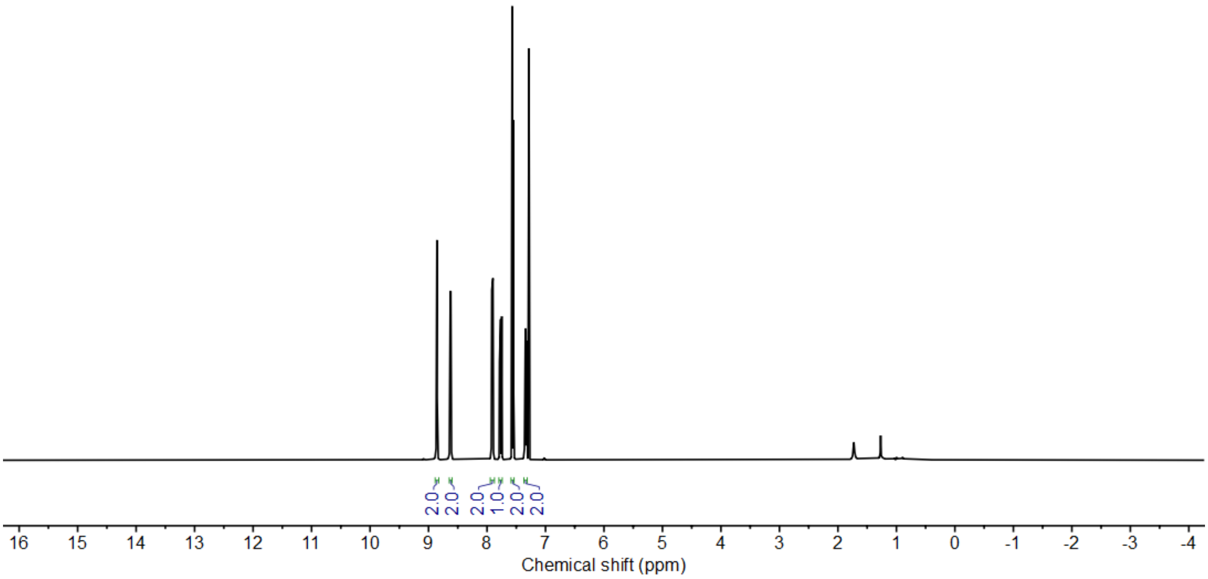

Figure S52 ^{1}H NMR (400 MHz, $CDCl_3$) of 3AA.

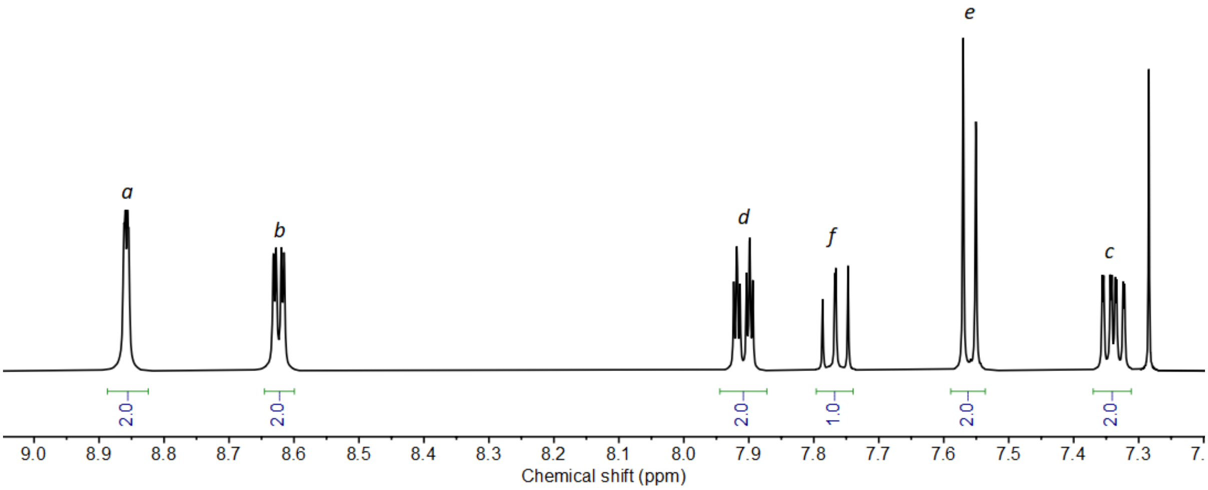

Figure S53 Partial ^{1}H NMR (400 MHz, $CDCl_3$) of 3AA.

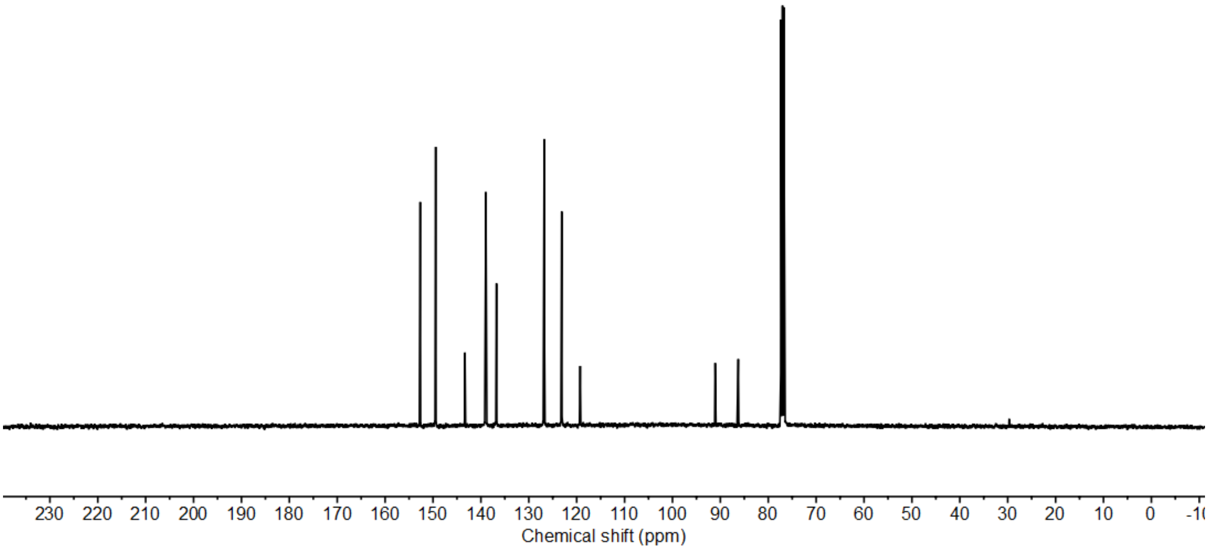

Figure S54 ^{13}C NMR (101 MHz, $CDCl_3$) of 3AA.

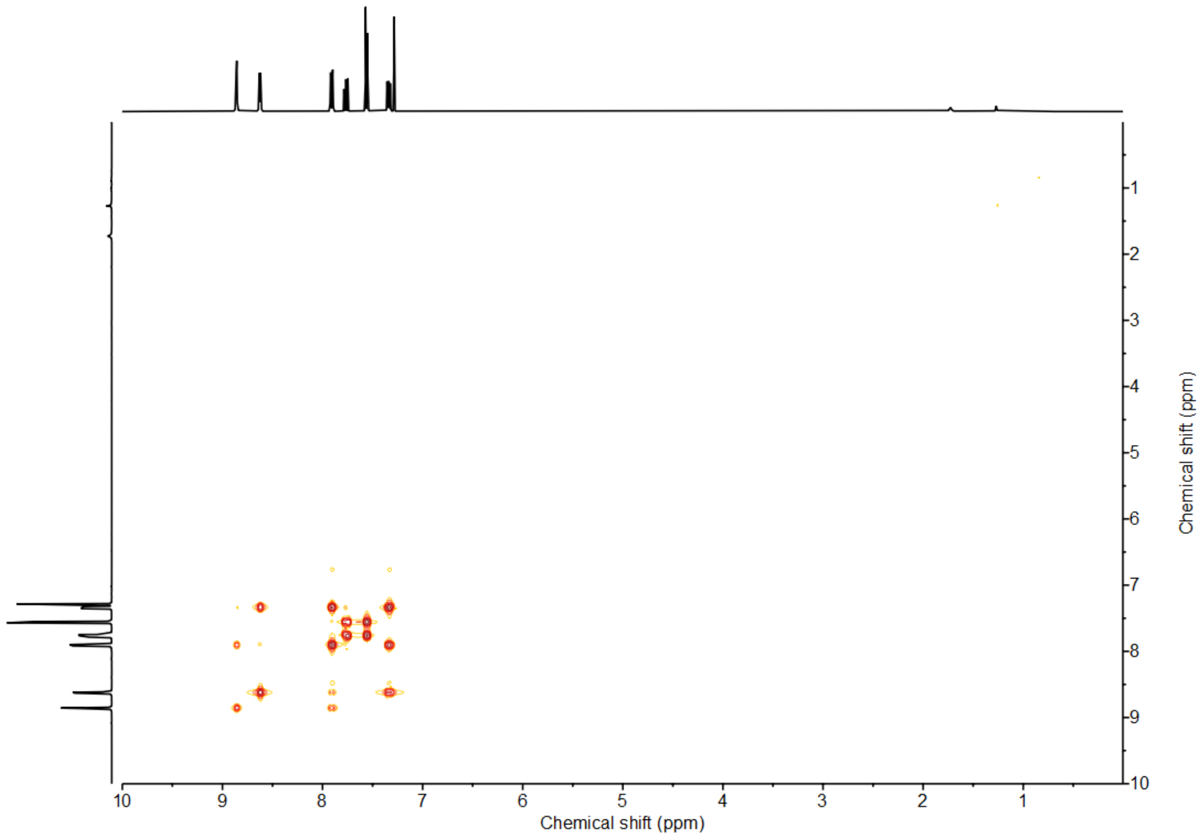

Figure S55 COSY ($CDCl_3$) of 3AA.

Synthesis of 4AA

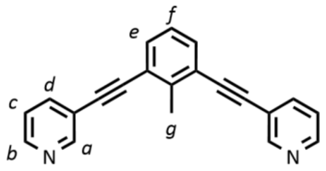

2,6-Dibromotoluene (0.246 g, 1.0 mmol, 1.0 eq.), 3-ethynylpyridine (0.209 g, 2.0 mmol, 2.0 eq.) and Pd(dppf)$Cl_2 \cdot CH_2Cl_2$ (0.041 g, 0.05 mmol, 5 mol%) were stirred at 80 °C in 1.0 M TBAF in THF (5 mL) in a sealed vial for 24 h. Brine (25 mL) was added to the cooled reaction mixture, and the aqueous phase subsequently extracted with Et_2O (3 × 25 mL). The combined organic phases were washed with 5% wt. aq. LiCl solution (2 x 25 mL), dried ($MgSO_4$) and the solvent removed *in vacuo*. After purification by column chromatography on silica gel (gradient 0 to 100% acetone in CH_2Cl_2) the product was obtained as a brown oil that solidified upon standing (0.069 g, 24%).

^{1}H NMR (400 MHz, $CDCl_3$) δ: 8.79 (dd, *J* = 2.2, 0.9 Hz, 2H, H_a), 8.57 (dd, *J* = 4.9, 1.7 Hz, 2H, H_b), 7.83 (app. dt, *J* = 7.9, 1.9 Hz, 2H, H_d), 7.53 (d, *J* = 7.8 Hz, 2H, H_e), 7.31 (ddd, *J* = 7.9, 4.9, 0.9 Hz, 2H, H_c), 7.21 (m, 1H, H_f), 2.72 (s, 3H, H_g).

^{13}C NMR (101 MHz, $CDCl_3$) δ: 152.22 (C_a), 148.77 (C_b), 142.89, 138.00 (C_d), 132.53 (C_e), 126.46 (C_f), 123.09 (C_c), 120.40, 91.02, 89.71, 18.69 (C_g).

HR-ESI-MS *m/z* = 295.1232 $[M+H]^+$ calc. 295.1235 (Δ = 1.0 ppm).

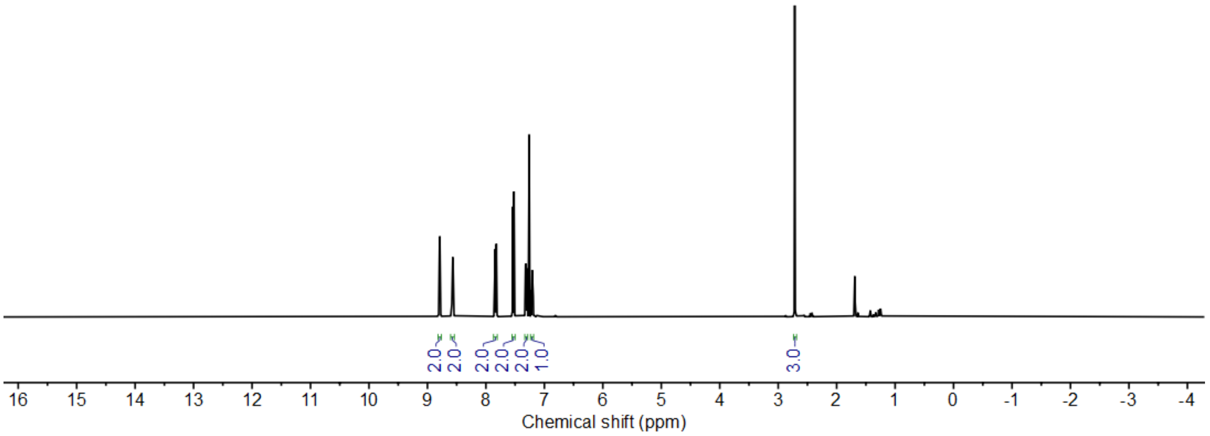

Figure S56 ^{1}H NMR (400 MHz, $CDCl_3$) of 4AA.

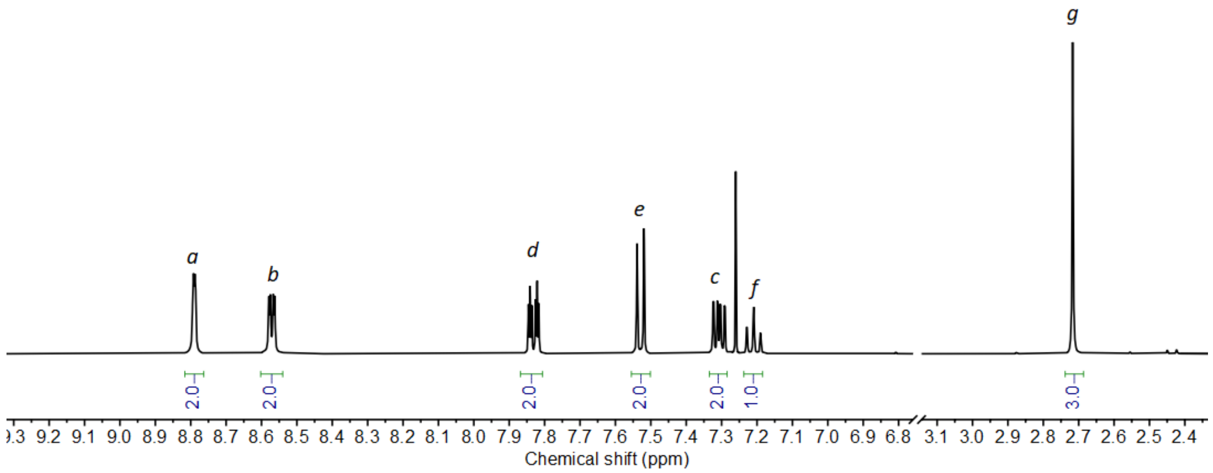

Figure S57 Partial ^{1}H NMR (400 MHz, $CDCl_3$) of 4AA.

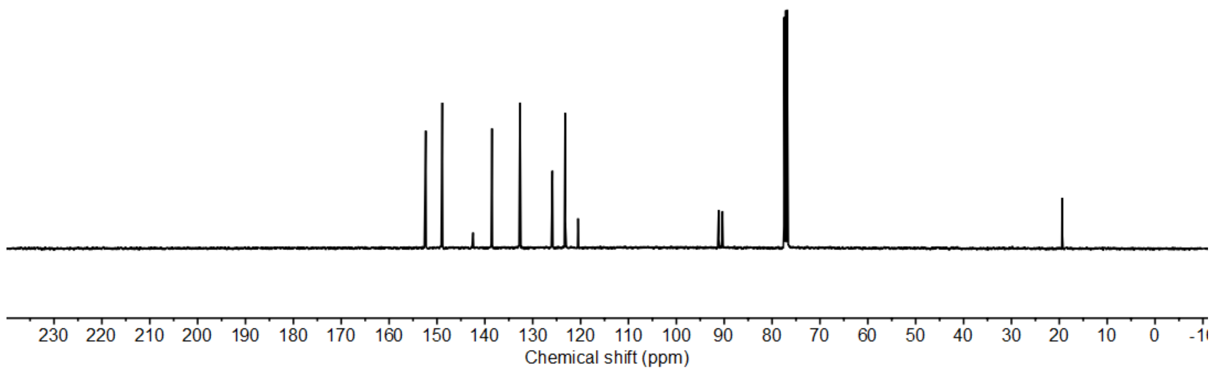

Figure S58 ^{13}C NMR (101 MHz, $CDCl_3$) of 4AA.

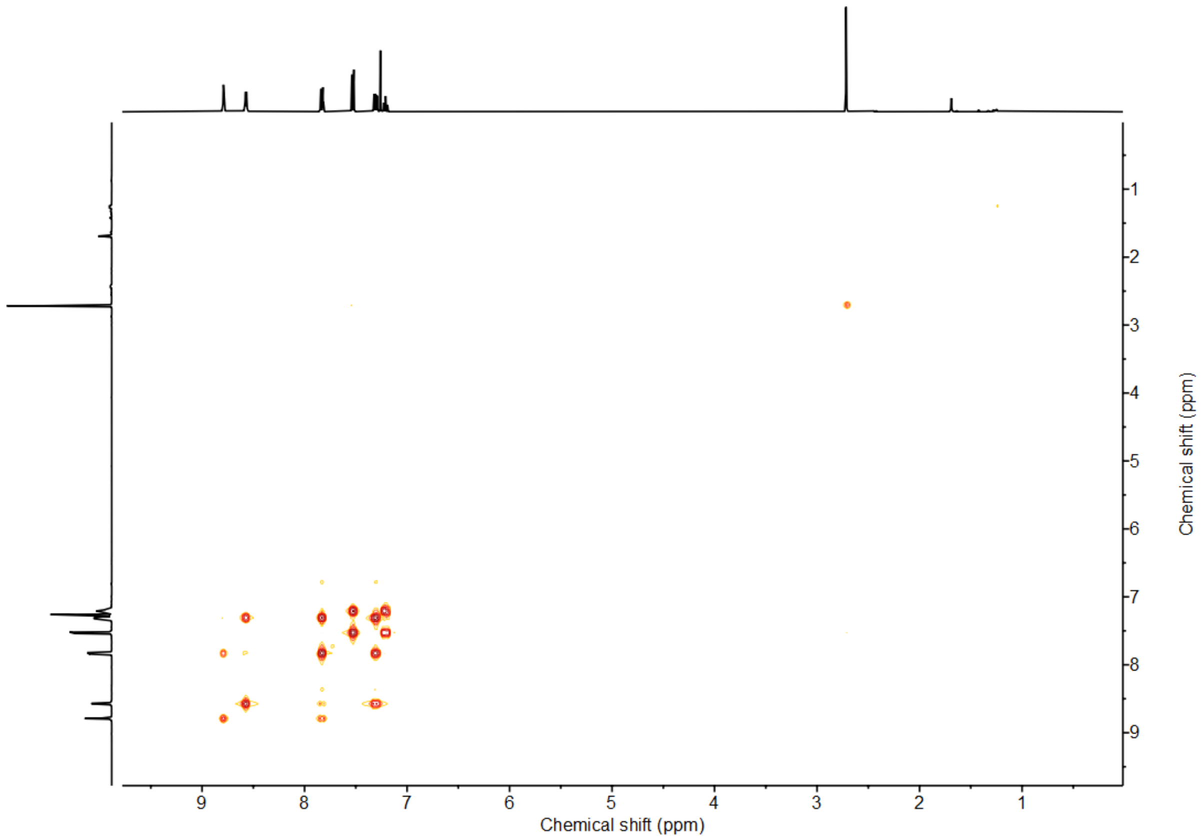

Figure S59 COSY ($CDCl_3$) of 4AA.

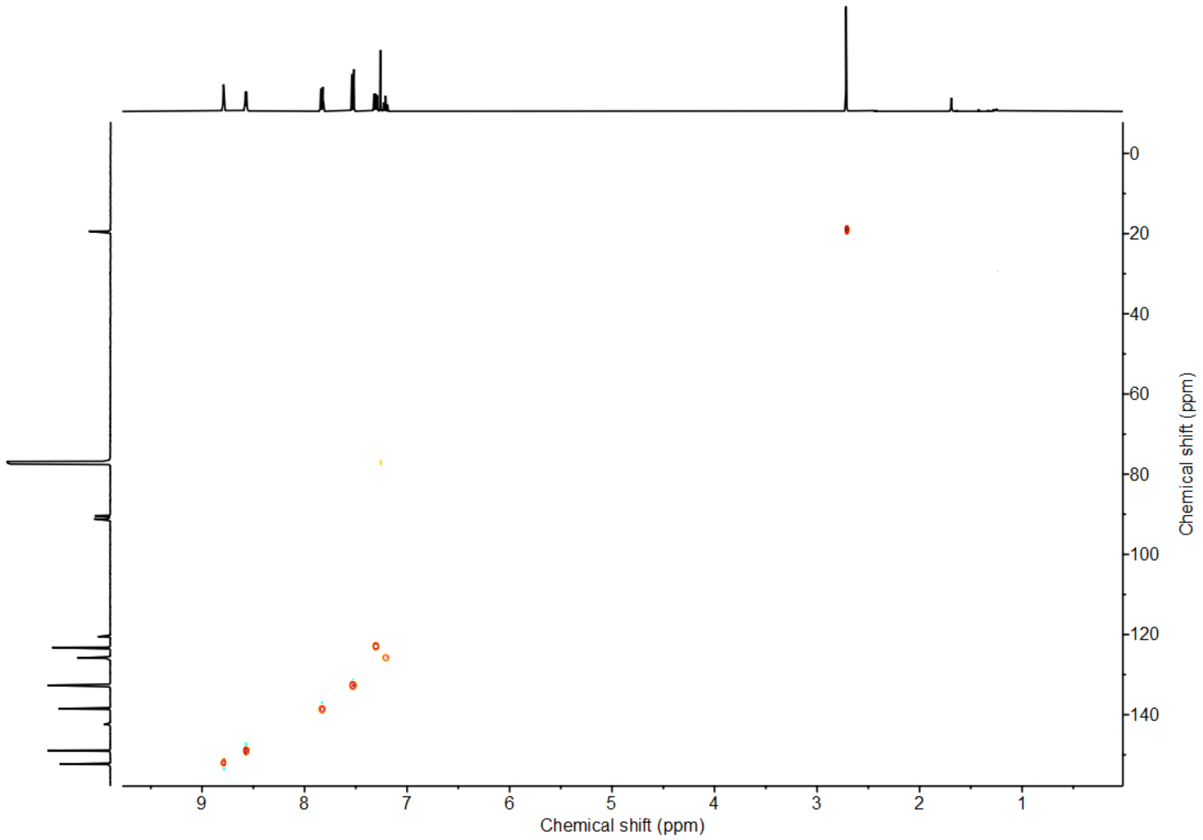

Figure S60 HSQC ($CDCl_3$) of 4AA.

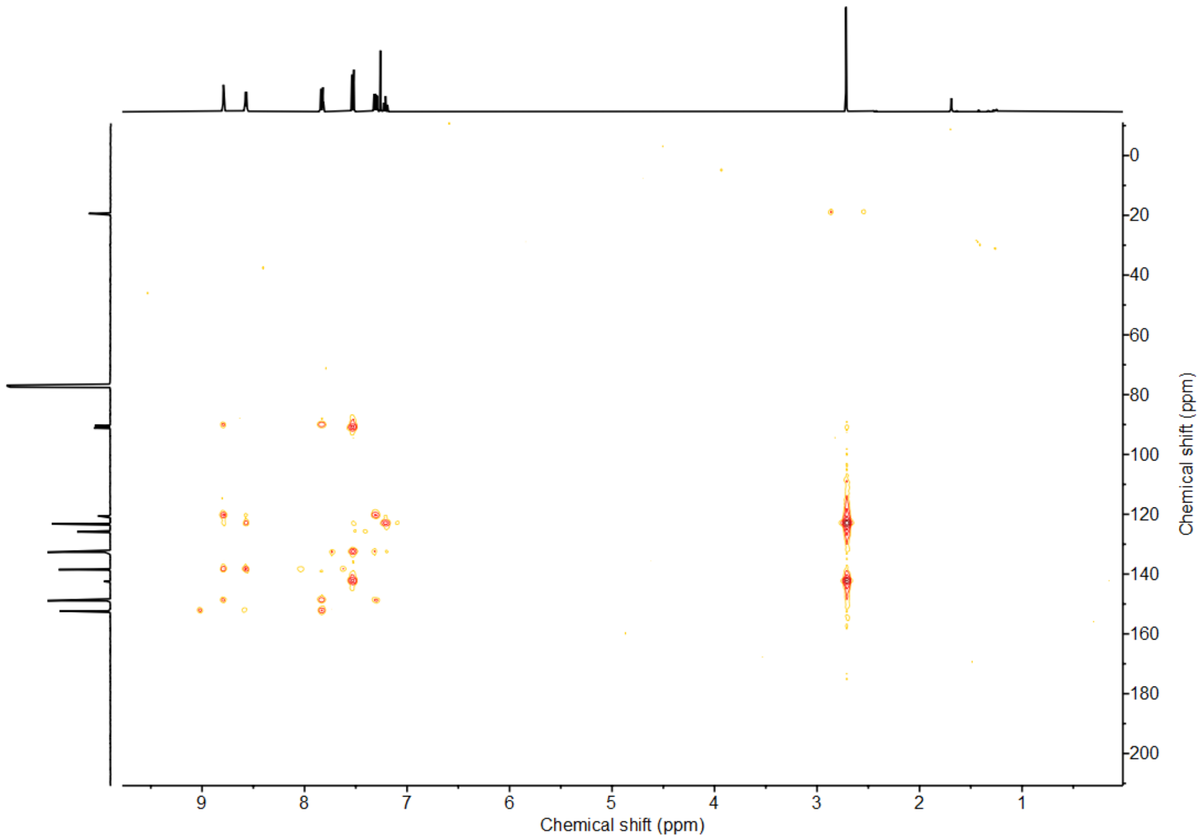

Figure S61 HMBC ($CDCl_3$) of 4AA.

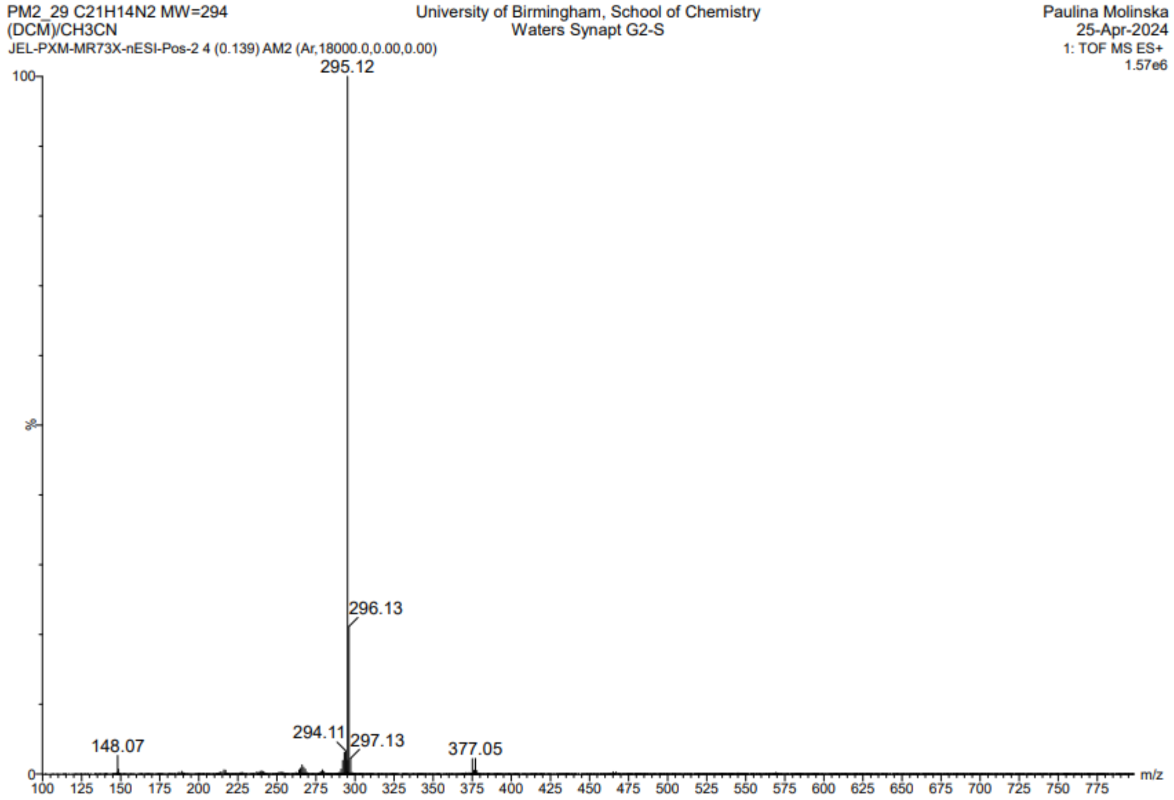

Figure S62 ESI-MS of 4AA.

Synthesis of 2AB

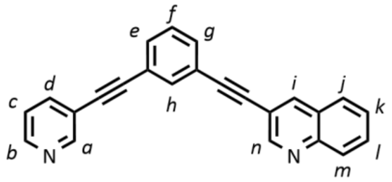

3-((3-Iodophenyl)ethynyl)quinoline (0.076 g, 0.2 mmol, 1.0 eq.), 3-ethynylpyridine (0.033 g, 0.3 mmol, 1.5 eq.) and Pd(dppf)$Cl_2 \cdot CH_2Cl_2$ (0.009 g, 0.01 mmol, 5 mol%) were stirred at 80 °C in 1.0 M TBAF in THF (1 mL) in a sealed vial for 24 h. H_2O (20 mL) and brine (10 mL) were added to the cooled reaction mixture, and the aqueous phase subsequently extracted with Et_2O (3 × 30 mL). The combined organic phases were dried ($MgSO_4$) and the solvent removed *in vacuo*. After purification by column chromatography on silica gel (gradient 0 to 50% EtOAc in hexane) the product obtained as a white solid (0.045 g, 64%).

^{1}H NMR (400 MHz, $CDCl_3$) δ: 9.01 (d, *J* = 2.1 Hz, 1H, H_n), 8.79 (dd, *J* = 2.2, 0.9 Hz, 1H, H_a), 8.58 (dd, *J* = 4.9, 1.6 Hz, 1H, H_b), 8.33 (dd, *J* = 2.1, 0.9 Hz, 1H, H_i), 8.11 (dd, *J* = 8.4, 1.0 Hz, 1H, H_m), 7.85-7.79 (m, 3H, H_d, H_h, H_j), 7.75 (ddd, *J* = 8.4, 6.9, 1.5 Hz, 1H, H_l), 7.62-7.54 (m, 3H, H_e, H_g, H_k), 7.41 (td, *J* = 7.8, 0.6 Hz, 1H, H_f), 7.31 (ddd, *J* = 7.9, 4.9, 0.9 Hz, 1H, H_c).

^{13}C NMR (101 MHz, $CDCl_3$) δ: 152.49, 152.19, 148.99, 147.12, 138.64, 138.60, 134.95, 132.03, 131.99, 130.41, 129.63, 128.89, 127.81, 127.55, 127.38, 123.30, 123.24, 120.30, 117.25, 91.73, 91.68, 87.58, 86.90. One 4° C signal missing.

HR-ESI-MS *m/z* = 331.1234 $[M+H]^+$ calc. 331.1235 (Δ = 0.30 ppm).

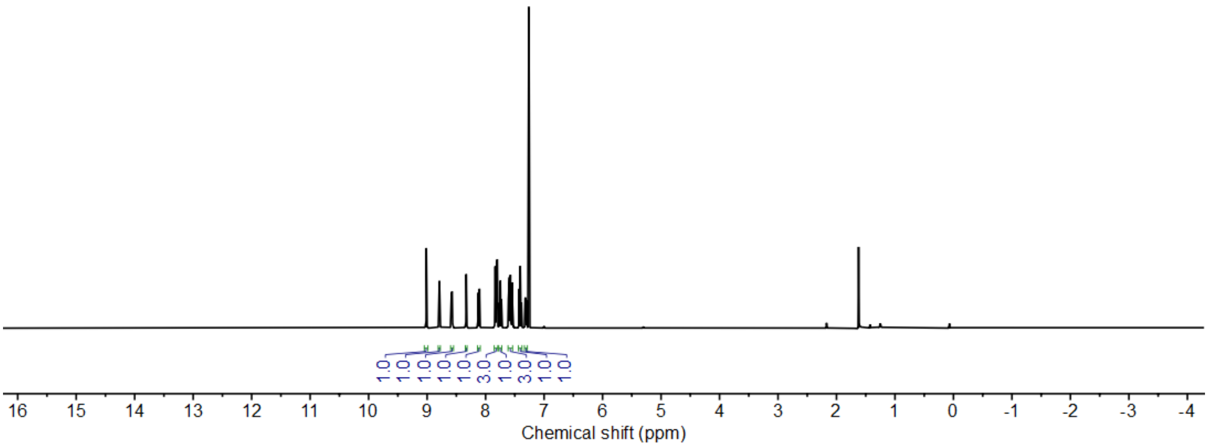

Figure S63 ^{1}H NMR (400 MHz, $CDCl_3$) of 2AB.

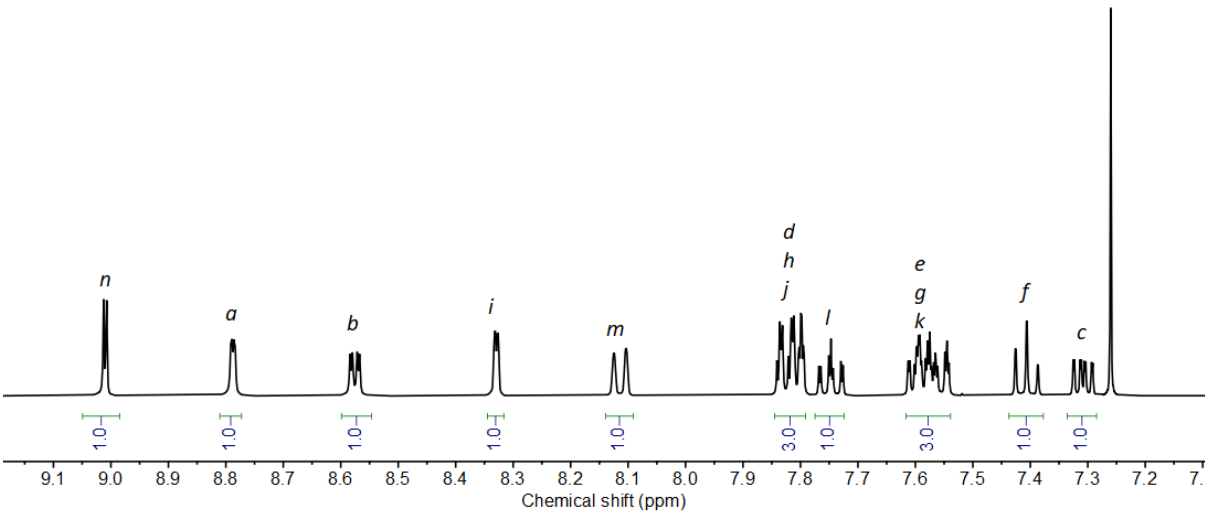

Figure S64 Partial ^{1}H NMR (400 MHz, $CDCl_3$) of 2AB.

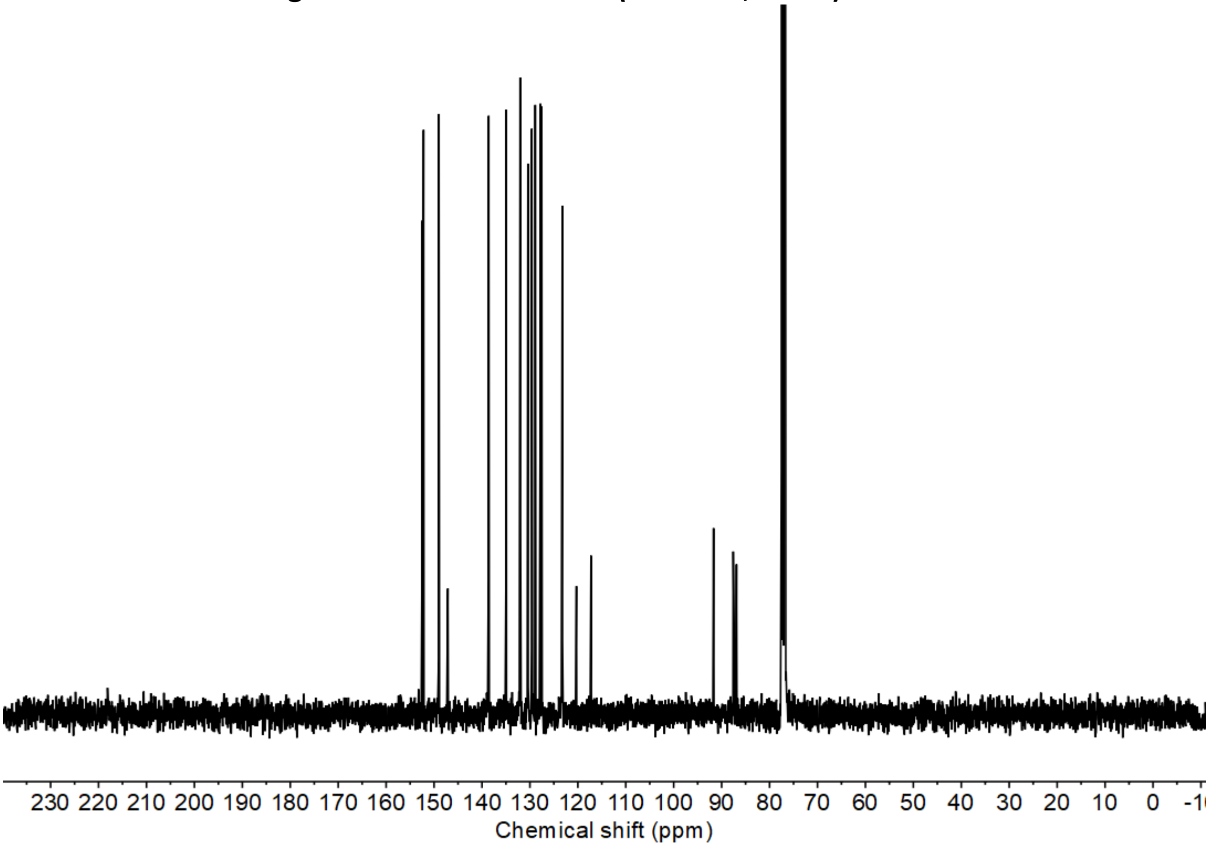

Figure S65 ^{13}C NMR (101 MHz, $CDCl_3$) of 2AB.

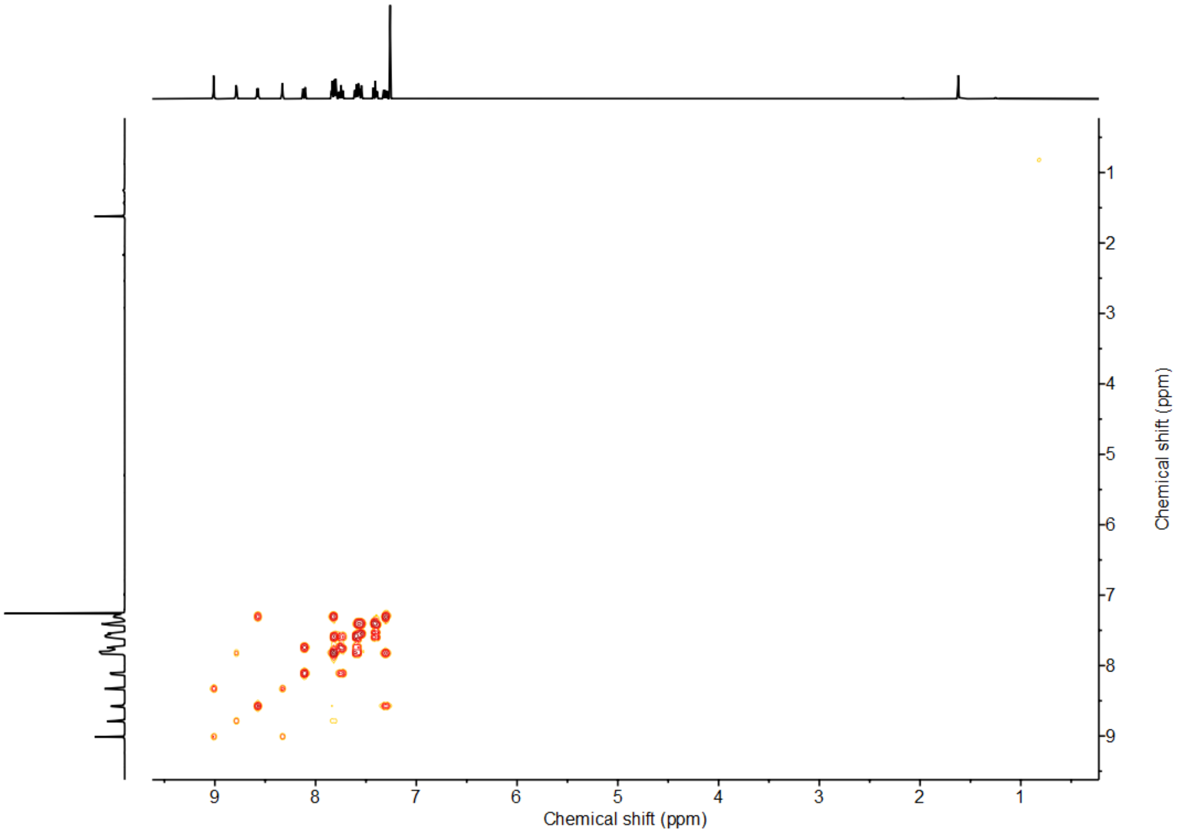

Figure S66 COSY ($CDCl_3$) of 2AB.

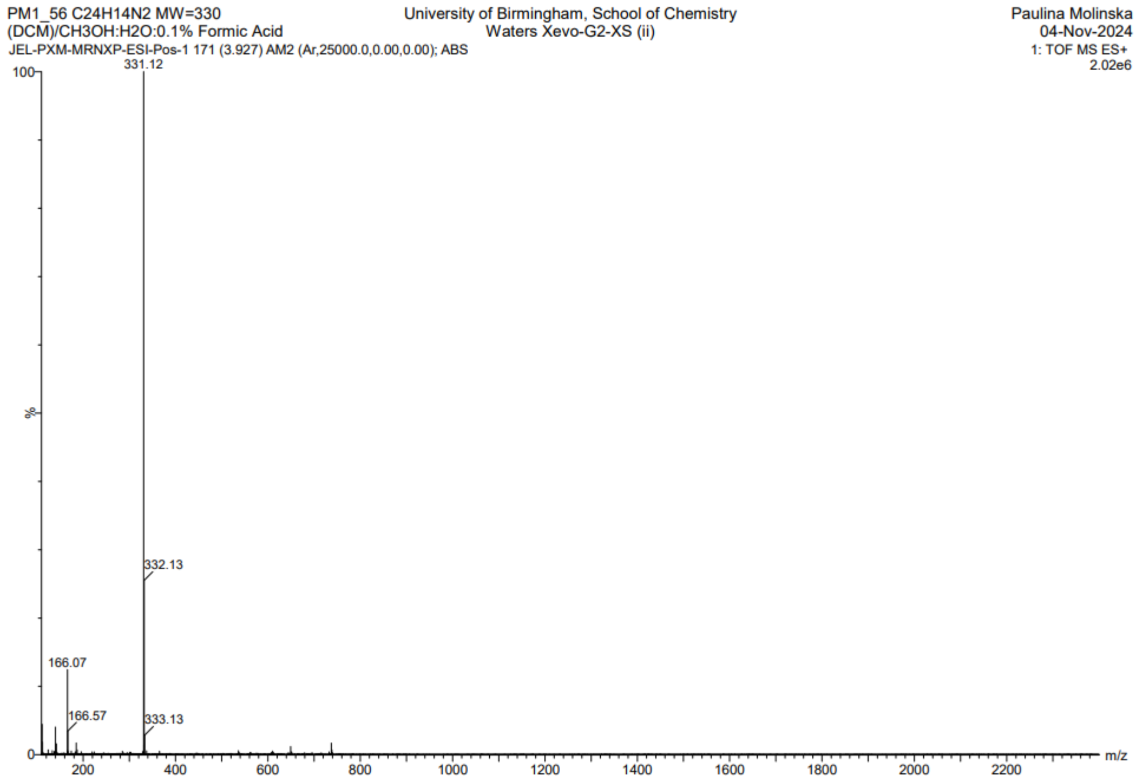

Figure S67 ESI-MS of 2AB.

Synthesis of 2AC

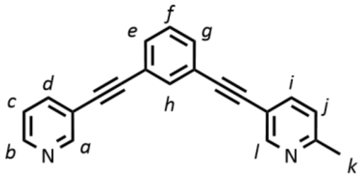

3-((3-iodophenyl)ethynyl)pyridine (0.278 g, 1.0 mmol, 1.0 eq.), **S2** (0.300 g, 1.0 mmol, 1.0 eq.) and Pd(dppf)$Cl_2 \cdot CH_2Cl_2$ (0.041 g, 0.05 mmol, 5 mol%) were stirred at 100 °C in 1.0 M TBAF in THF (5 mL) in a sealed vial for 24 h. Brine (30 mL) was added to the cooled reaction mixture, and the aqueous phase subsequently extracted with Et_2O (3 × 30 mL). The combined organic phases were washed with 5% wt. aq. LiCl solution (2 x 30 mL) then dried ($MgSO_4$), and the solvent removed *in vacuo*. After purification by column chromatography on silica gel (gradient 0 to 38% EtOAc in hexane) the product obtained as an off-white solid (0.234 g, 80%).

Spectroscopic data were consistent with a previous literature report.[14b]

^{1}H NMR (400 MHz, $CDCl_3$) δ: 8.77 (dd, *J* = 2.1, 0.9 Hz, 1H, H_a), 8.66 (app. dd, *J* = 2.2, 0.8 Hz, 1H, H_l), 8.57 (dd, *J* = 4.9, 1.7 Hz, 1H, H_b), 7.81 (app. dt, *J* = 7.9, 1.9 Hz, 1H, H_d), 7.74 (app. td, *J* = 1.7, 0.6 Hz, 1H, H_h), 7.70 (dd, *J* = 8.0, 2.2 Hz, 1H, H_i), 7.56-7.49 (m, 2H, H_e, H_g), 7.37 (ddd, *J* = 8.1, 7.4, 0.6 Hz, 1H, H_f), 7.30 (ddd, *J* = 7.9, 4.9, 0.9 Hz, 1H, H_c), 7.16 (d, *J* = 8.0 Hz, 1H,H_j), 2.59 (s, 3H, H_k).

^{13}C NMR (101 MHz, $CDCl_3$) δ: 158.21, 152.46, 151.81, 148.95, 138.86, 138.62, 134.83, 131.91, 131.77, 128.80, 123.44, 123.21, 123.15, 122.91, 120.31, 117.14, 91.77, 90.94, 87.18, 86.79, 24.69.

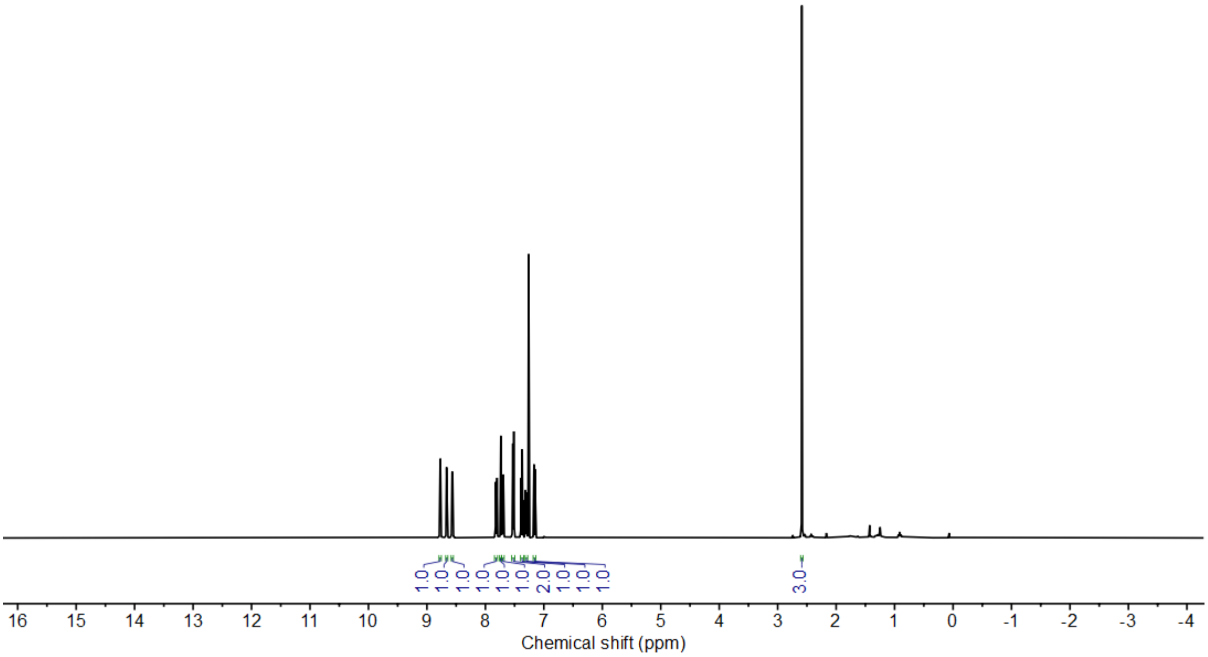

Figure S68 ^{1}H NMR (400 MHz, $CDCl_3$) of 2AC.

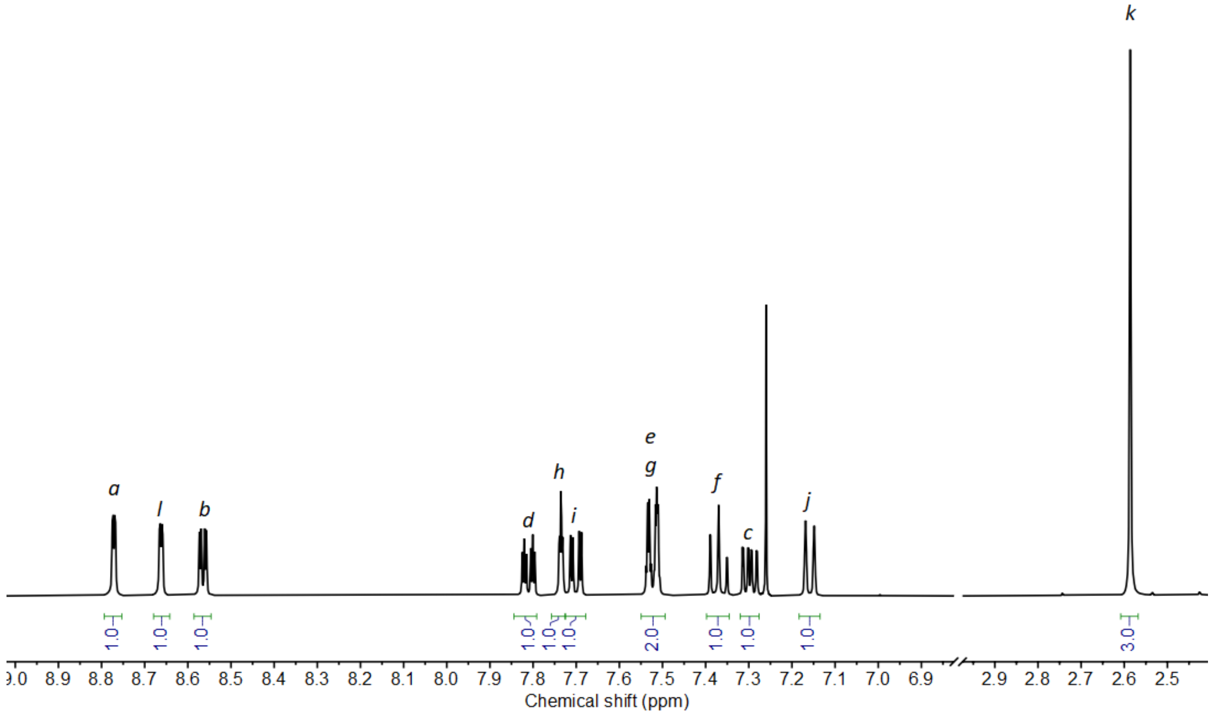

Figure S69 Partial ^{1}H NMR (400 MHz, $CDCl_3$) of 2AC.

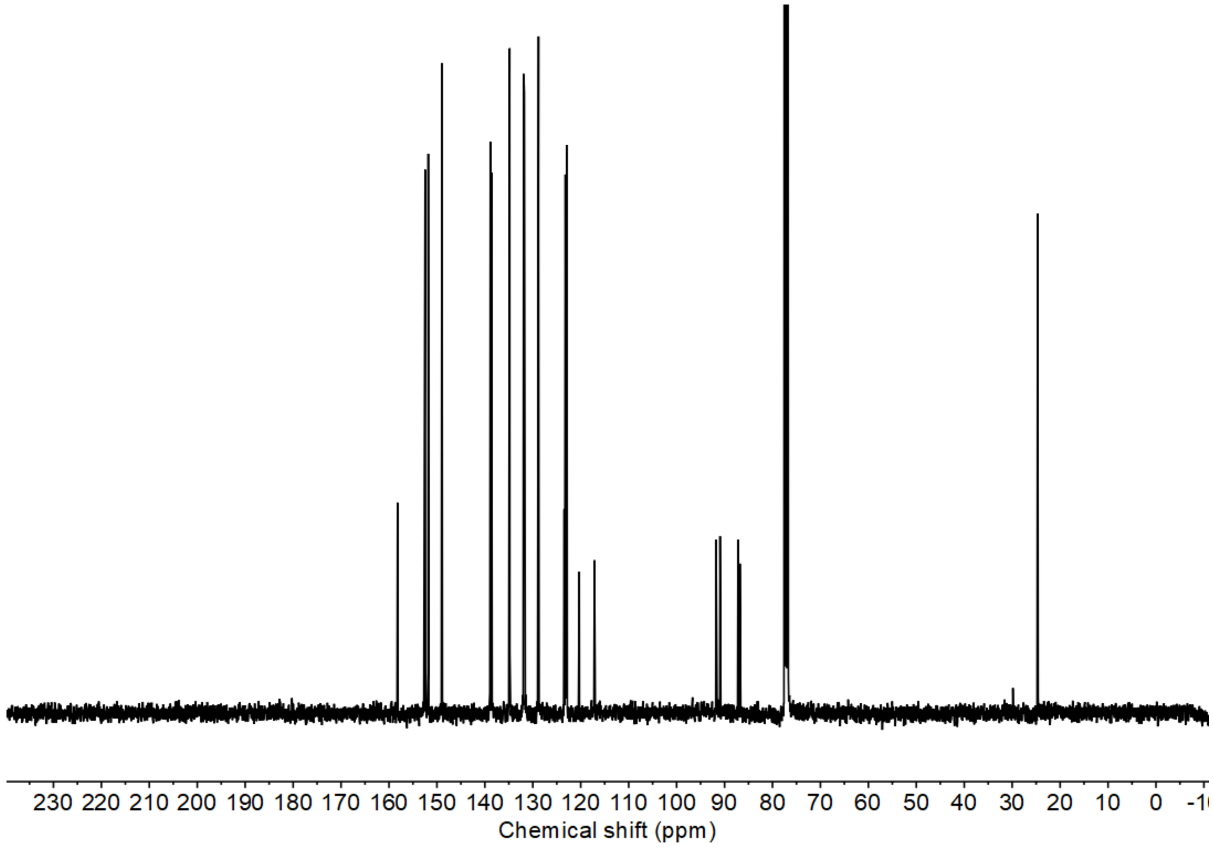

Figure S70 ^{13}C NMR (101 MHz, $CDCl_3$) of 2AC.

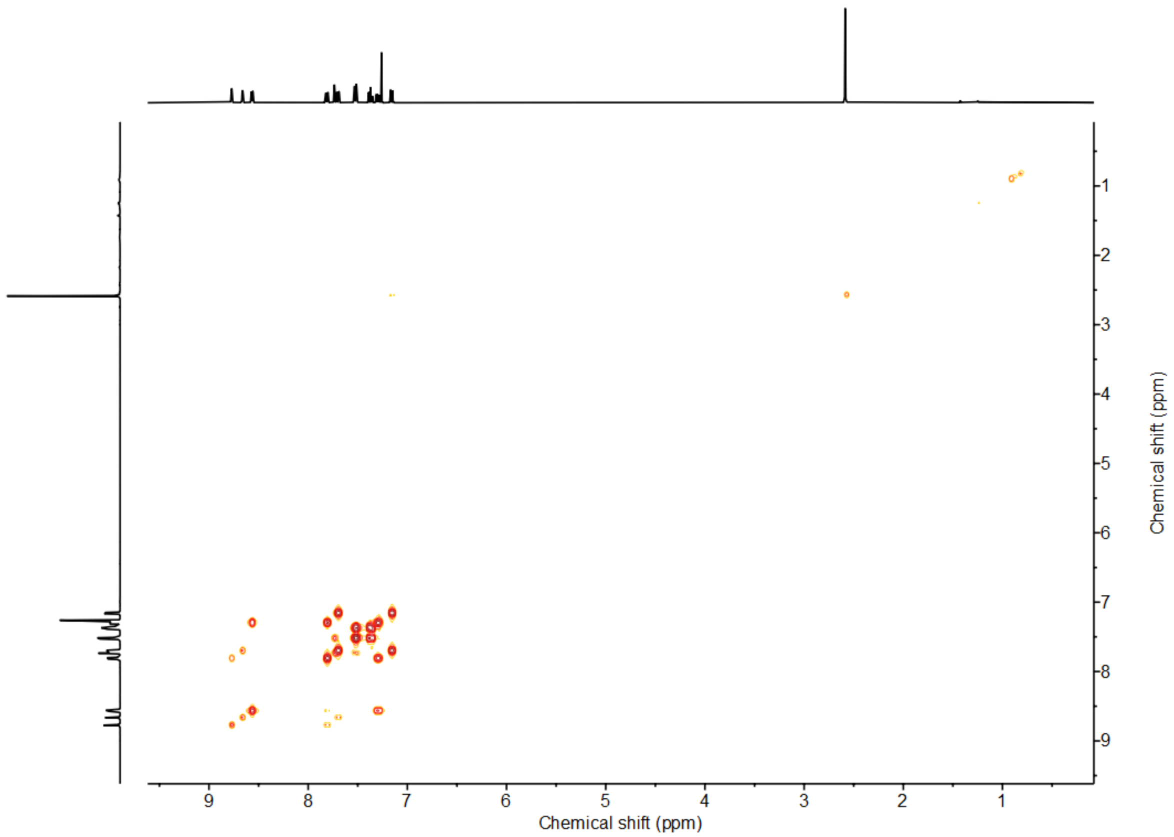

Figure S71 COSY ($CDCl_3$) of 2AC.

Synthesis of $[Pd_2(\mathbf{1AB})_2(\mathbf{2AA})_2](BF_4)_4$

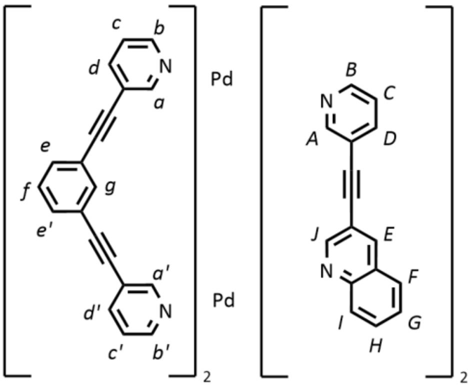

1AB (11.5 mg, 50 µmol, 1 eq.), **2AA** (14.0 mg, 50 µmol, 1 eq.) and $[Pd(CH_3CN)_4](BF_4)_2$ (22.2 mg, 50 µmol, 1 eq.) were stirred at 70 °C in anhydrous CH_3CN (5.0 mL) under N_2 for 24 h. The cooled reaction mixture was filtered through celite. Vapour diffusion of Et_2O into this solution yielded a precipitate. After the mother liquor was decanted, the solid was washed with Et_2O (× 3) and dried *in vacuo* to give the product as a beige solid (29.5 mg, 75%).

ESI-MS *m/z* = 440.06 $\{[Pd_2(\mathbf{1AB})_2(\mathbf{2AA})_2](BF_4)\}^{3+}$ calc. 440.39; 670.09 $\{[Pd_2(\mathbf{1AB})_2(\mathbf{2AA})_2](BF_4)F\}^{2+}$ calc. 670.09.

^{13}C NMR (101 MHz, CD_3CN) δ: 156.50 (*syn*-C_J, *anti*-C_J), 155.28 (*syn*-$C_{a'}$), 155.09 (*anti*-$C_{a'}$), 154.90 (*syn*-C_a, *anti*-C_a), 154.56 (*syn*-C_A, *anti*-C_A), 152.13 (*syn*-C_B), 151.97 (*anti*-C_B), 151.05 (*syn*-C_b), 150.81 (*anti*-C_b), 150.68 (*anti*-$C_{b'}$), 150.35 (*syn*-$C_{b'}$), 146.31, 146.21, 144.16 (*syn*-C_E, *anti*-C_E), 143.03 (*syn*-C_d, *syn*-$C_{d'}$), 142.87, 142.56 (*anti*-C_d /*anti*-$C_{d'}$), 142.35 (*syn*-C_D), 139.39 (*anti*-C_g), 139.28 (*syn*-C_g), 136.05 (*anti*-C_H), 135.73 (*syn*-C_H), 132.59 (*syn*-C_e /*syn*-$C_{e'}$), 132.49 (*anti*-C_e /*anti*-$C_{e'}$), 132.36 (*syn*-C_e /*syn*-$C_{e'}$, *anti*-C_e /*anti*-$C_{e'}$), 131.24 (*anti*-C_D /*anti*-C_F /*anti*-C_G), 131.10 (*syn*-C_F, *syn*-C_G, *anti*-C_D /*anti*-C_F / *anti*-C_G), 130.81 (*syn*-C_f, *anti*-C_f), 130.18, 130.06, 128.86 (*syn*-C_c /*syn*-$C_{c'}$, *anti*-C_c /*anti*-$C_{c'}$), 128.78 (*syn*-C_c /*syn*-$C_{c'}$, *anti*-C_c /*anti*-$C_{c'}$), 128.63, 128.42 (*syn*-C_C, *anti*-C_C), 127.47 (*syn*-C_I, *anti*-C_I), 125.15, 125.07, 124.96, 124.75, 124.50, 124.23, 123.14, 123.11, 123.05, 123.02, 119.2, 119.1, 95.55, 95.47, 95.40, 91.27, 90.89, 89.87, 86.12, 85.91, 85.73.

^{19}F NMR (376 MHz, CD_3CN) δ: -151.38.

^{1}H DOSY (400 MHz, CD_3CN) *D*: 7.9 × 10^{-10} m^2s^{-1}; R_S: 7.7 Å.

Major Isomer *syn*-$[Pd_2(1AB)_2(2AA)_2](BF_4)_4$

^{1}H NMR (400 MHz, CD_3CN) δ: 10.47 (s, 2H, H_J), 10.21 (dd, *J* = 8.7, 0.9 Hz, 2H, H_I), 10.11 (m, 2H, H_A), 9.50 (s, 2H, H_a), 9.48 (m, 2H, H_b), 9.37 (d, *J* = 1.8 Hz, 2H, $H_{a'}$), 9.16 (ddd, *J* = 5.8, 1.3, 0.6 Hz, 2H, $H_{b'}$), 8.99 (dd, *J* = 6.0, 1.4 Hz, 2H, H_B), 8.63 (m, 2H, H_E), 8.35 (ddd, *J* = 8.6, 7.1, 1.5 Hz, 2H, H_H), 8.23 (td, *J* = 1.7, 0.6 Hz, 2H, H_g), 8.08 (m, 2H, H_D), 8.05-7.97 (m, 6H, H_F, H_d, $H_{d'}$), 7. 84 (ddd, *J* = 8.1, 7.1, 0.9 Hz, 2H, H_G), 7.71 (ddd, *J* = 8.1, 5.8, 0.7 Hz, 2H, $H_{c'}$), 7.65 (m, 2H, H_c), 7.64-7.57 (m, 6H, H_C, H_e, $H_{e'}$), 7.49 (ddd, *J* = 8.9, 6.8, 0.6 Hz, 2H, H_f).

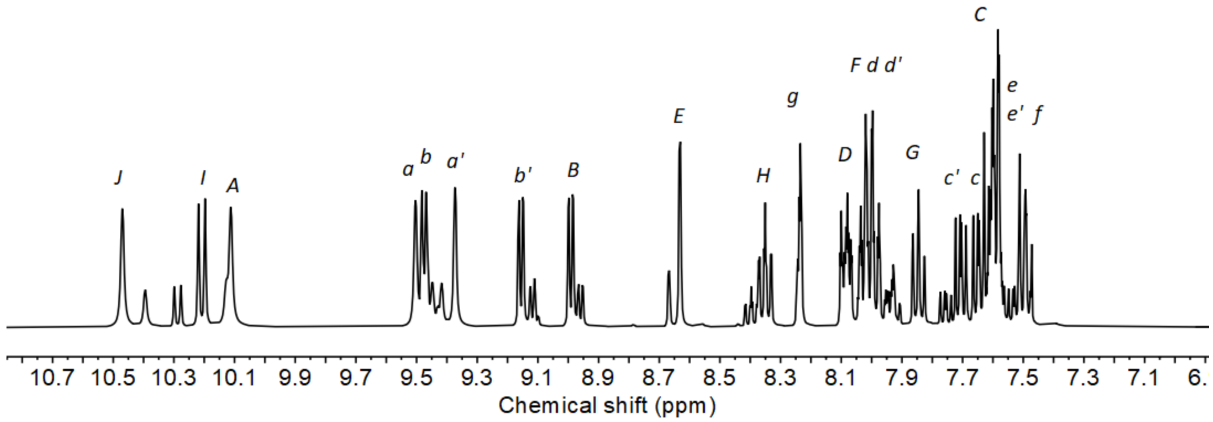

Figure S72 Partial ^{1}H NMR (400 MHz, CD_3CN) of $[Pd_2(1AB)_2(2AA)_2](BF_4)_4$ with peaks of major *syn*-isomer labelled.

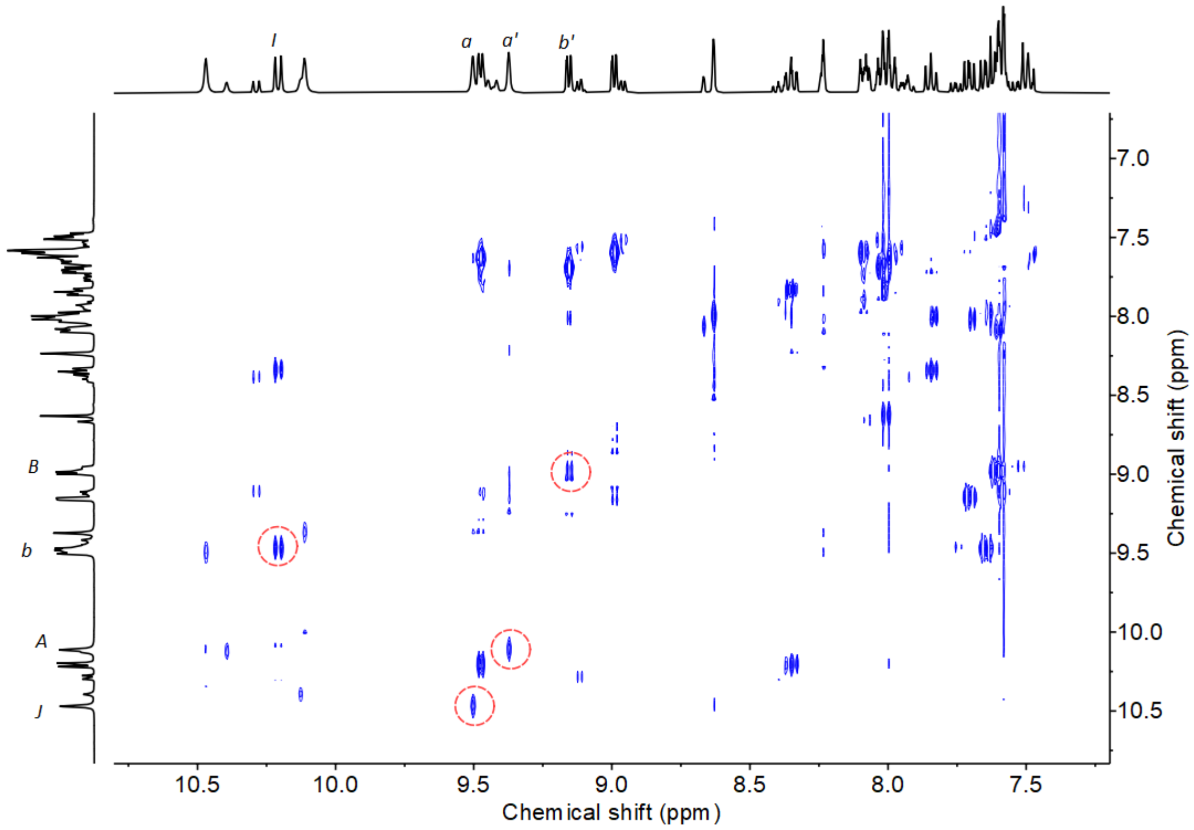

Figure S73 Partial NOESY (400 MHz, CD_3CN) of $[Pd_2(1AB)_2(2AA)_2](BF_4)_4$ with peaks assigned to major *syn*-isomer.

Minor Isomer *anti*-$[Pd_2(1AB)_2(2AA)_2](BF_4)_4$

^{1}H NMR (400 MHz, CD_3CN) δ: 10.39 (s, 2H, H_J), 10.29 (dt, *J* = 8.7, 0.8 Hz, 2H, H_I), 10.13 (s, 2H, H, H_A), 9.43 (m, 4H, H_b, $H_{a'}$), 9.12 (m, 2H, $H_{b'}$), 8.96 (dd, , *J* = 6.3, 1.3 Hz 2H, H_B), 8.67 (m, 2H, H_E), 8.40 (ddd, *J* = 8.6, 7.1, 1.6 Hz, 2H, H_H), 8.25 (app. dd, *J* = 1.7, 0.6 Hz, 2H, H_g), 8.08 (m, 4H, H_D, $H_{d/d'}$), 8.05-7.97 (m, 2H, $H_{d/d'}$), 7.93 (m, 4H, H_F, H_G), 7.76 (ddd, *J* = 8.1, 5.8, 0.7 Hz, 2H, $H_{c'}$), 7.65 (m, 2H, H_c), 7.64-7.57 (m, 4H, H_e, $H_{e'}$), 7.54 (m, 2H, H_C), 7.49 (ddd, *J* = 8.9, 6.8, 0.6 Hz, 2H, H_f).

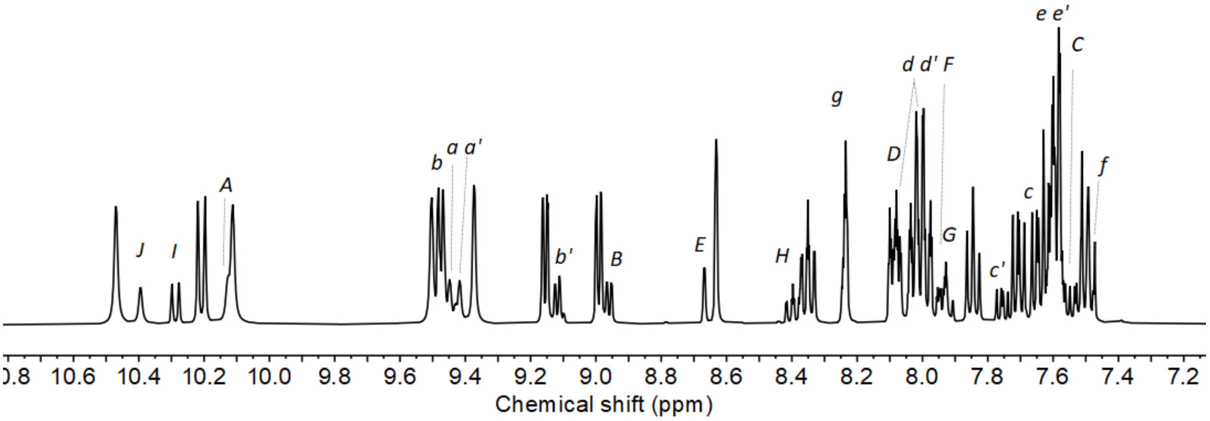

Figure S74 Partial ^{1}H NMR (400 MHz, CD_3CN) of $[Pd_2(1AB)_2(2AA)_2](BF_4)_4$ with peaks of minor *anti*-isomer labelled.

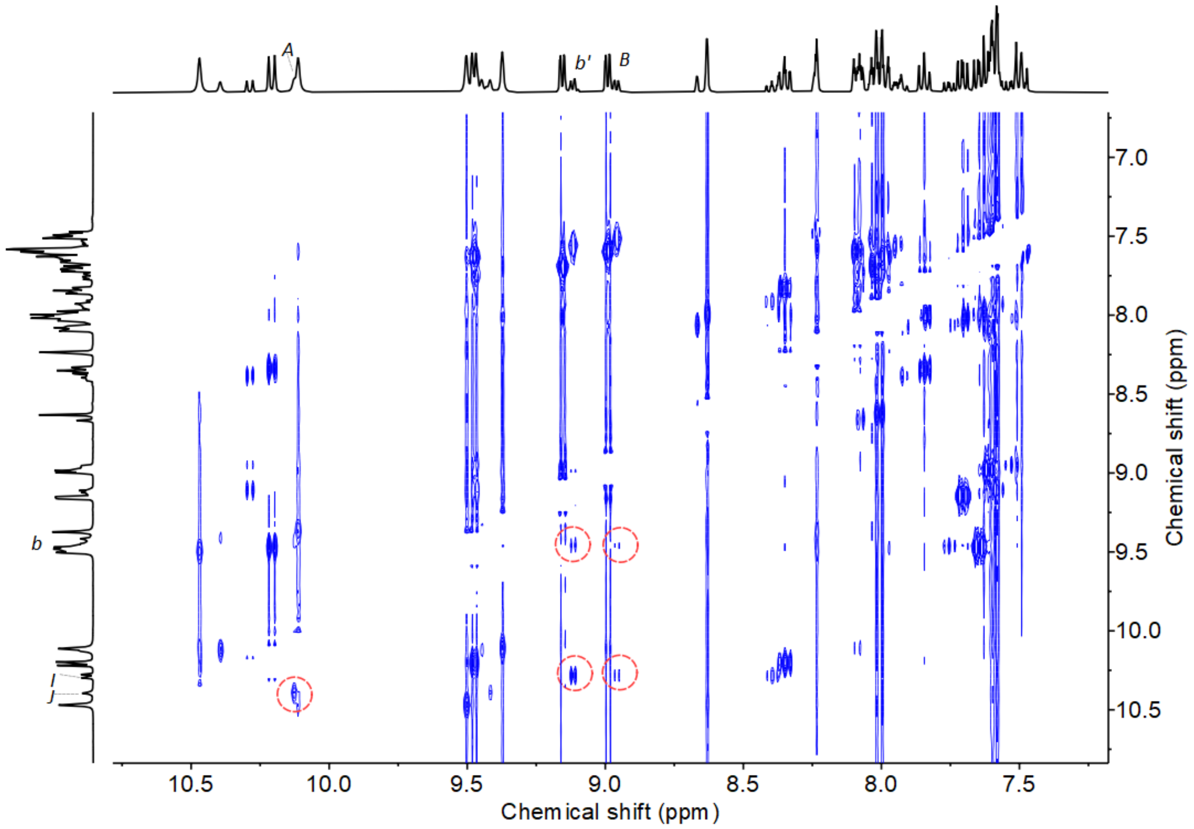

Figure S75 Partial NOESY (400 MHz, CD_3CN) of $[Pd_2(1AB)_2(2AA)_2](BF_4)_4$ with peaks assigned to minor *anti*-isomer.

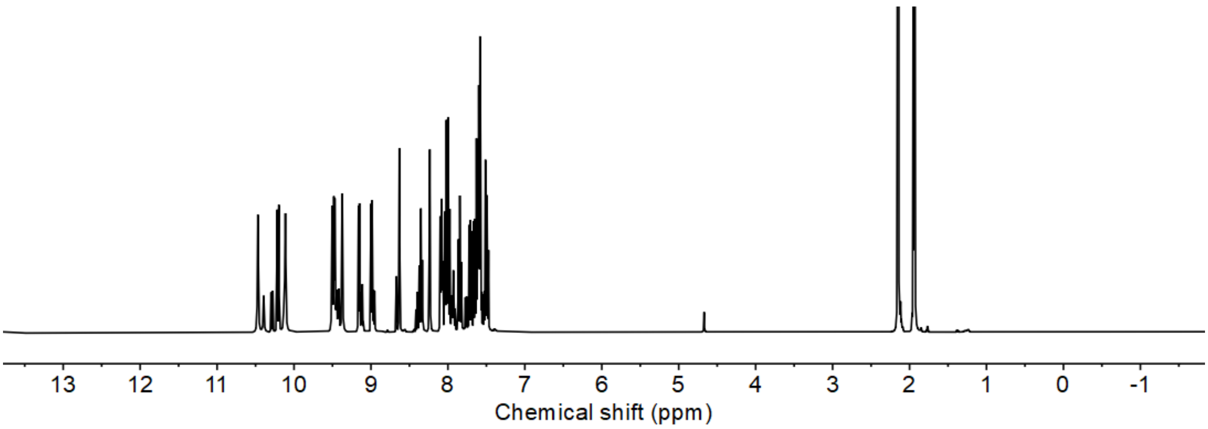

Figure S76 ^{1}H NMR (400 MHz, CD_3CN) of $[Pd_2(1AB)_2(2AA)_2](BF_4)_4$.

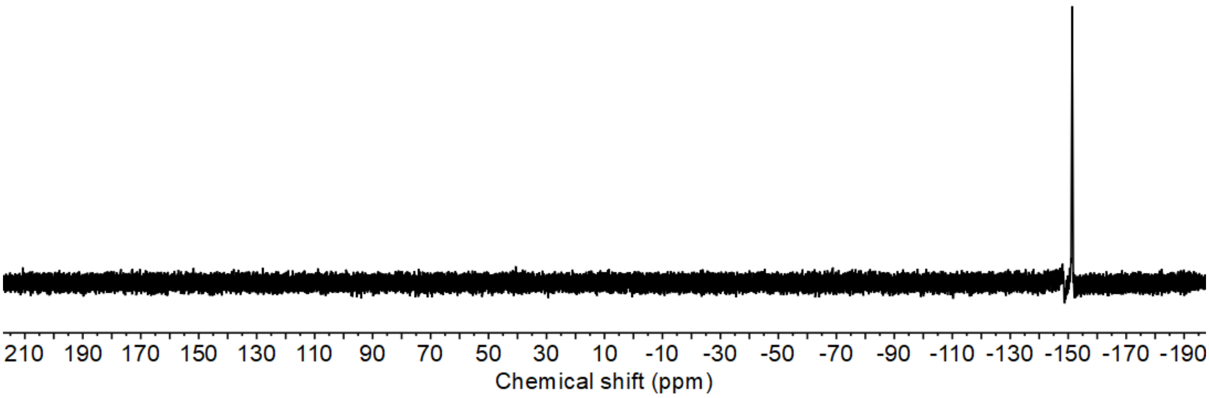

Figure S77 ^{19}F NMR (376 MHz, CD_3CN) of $[Pd_2(1AB)_2(2AA)_2](BF_4)_4$.

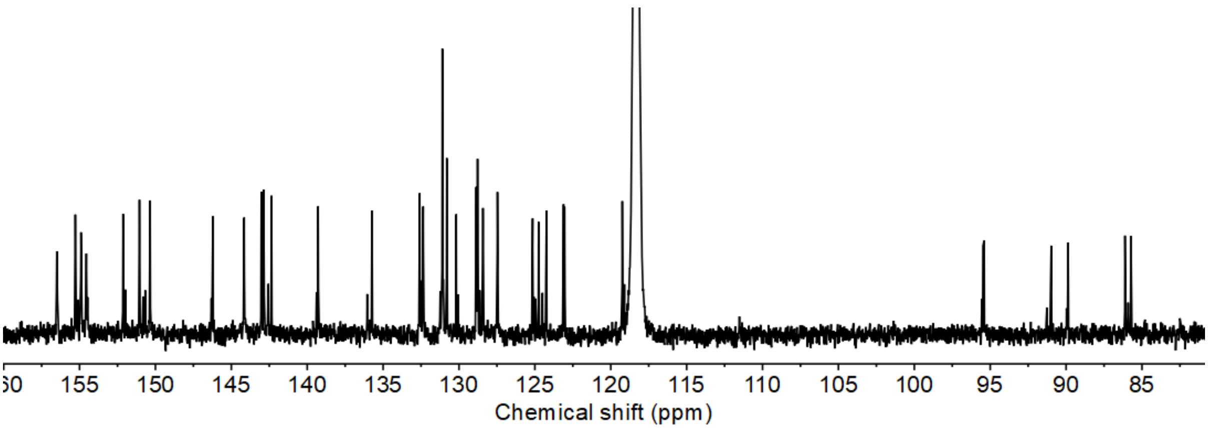

Figure S78 Partial ^{13}C NMR (101 MHz, CD_3CN) of $[Pd_2(1AB)_2(2AA)_2](BF_4)_4$.

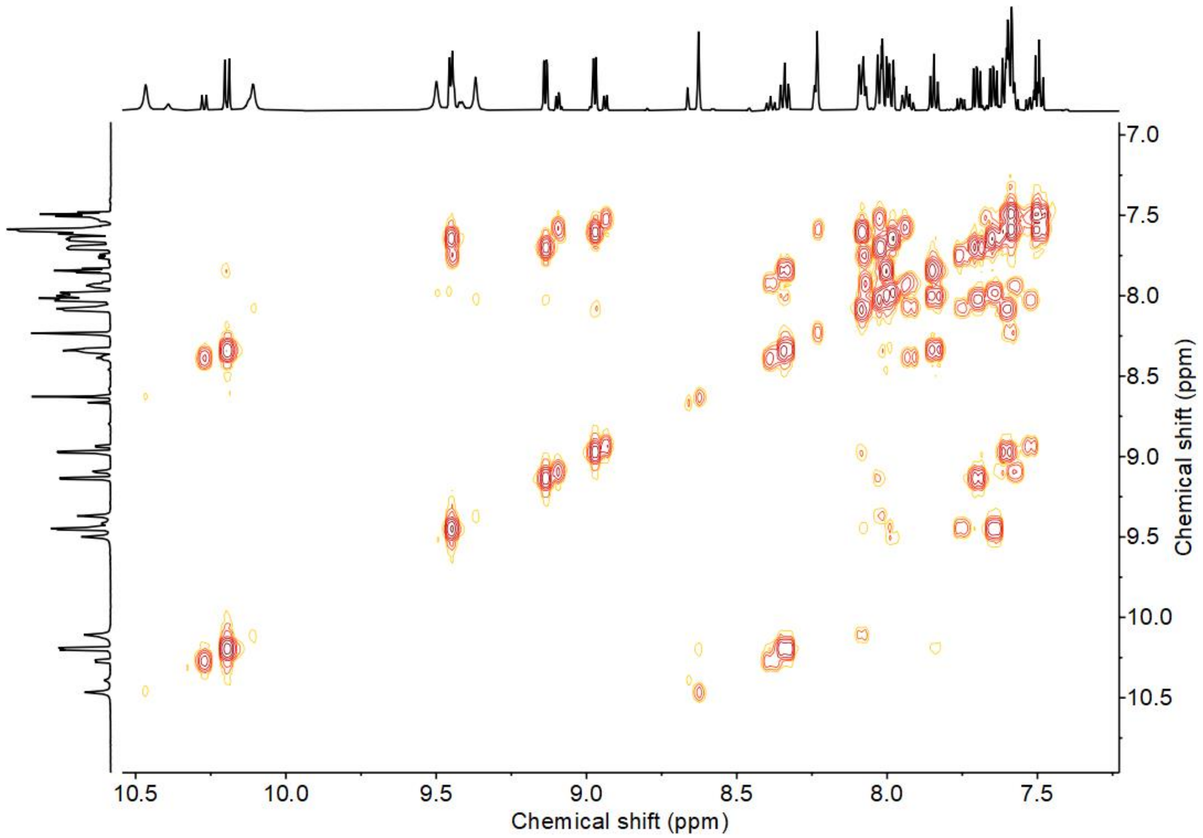

Figure S79 Partial COSY (600 MHz, CD_3CN) of $[Pd_2(1AB)_2(2AA)_2](BF_4)_4$.

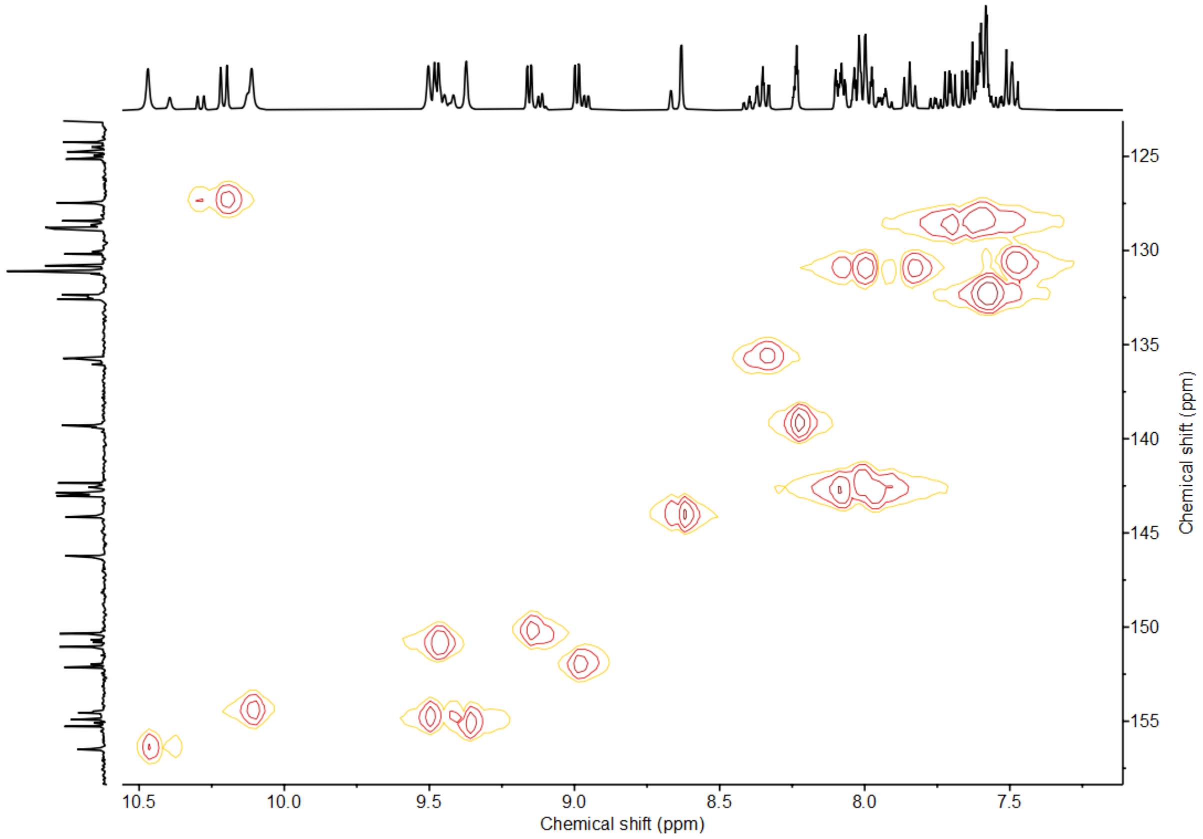

Figure S80 Partial HSQC (CD_3CN) of $[Pd_2(1AB)_2(2AA)_2](BF_4)_4$.

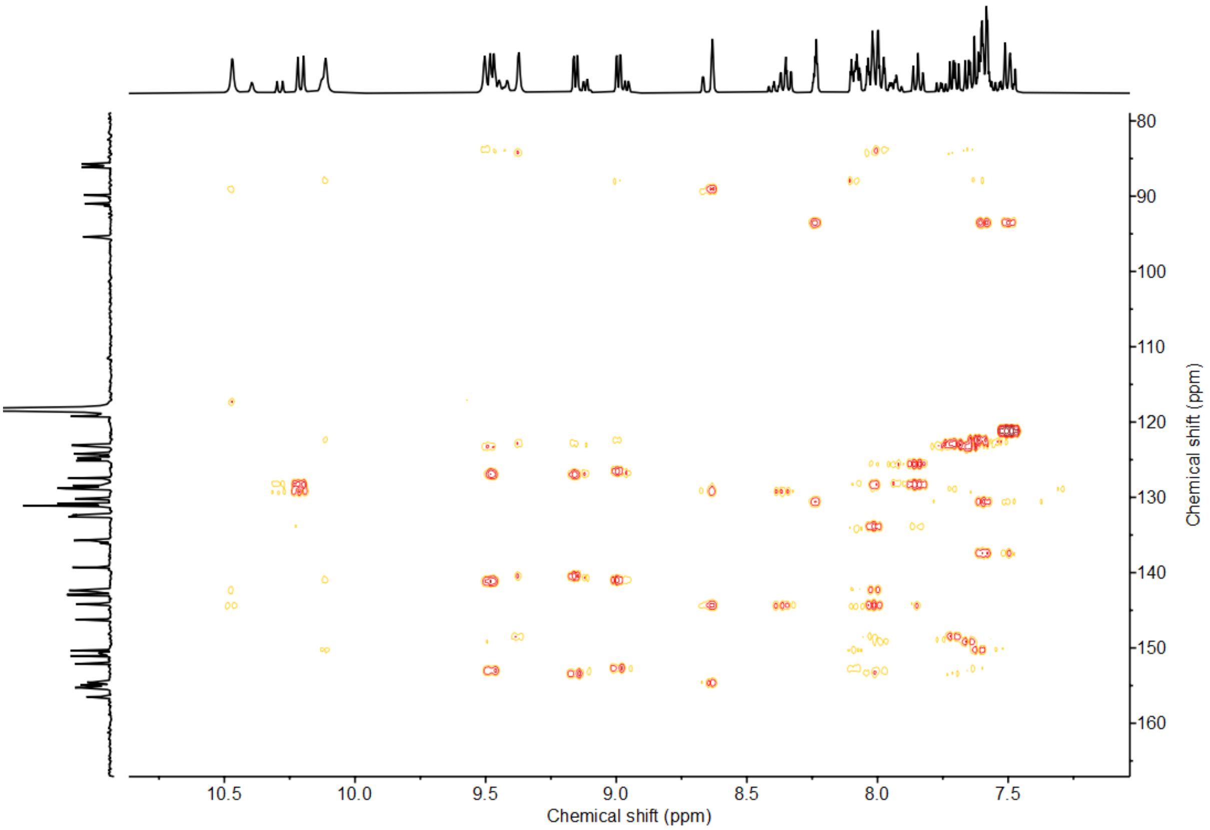

Figure S81 Partial HMBC (CD_3CN) of $[Pd_2(1AB)_2(2AA)_2](BF_4)_4$.

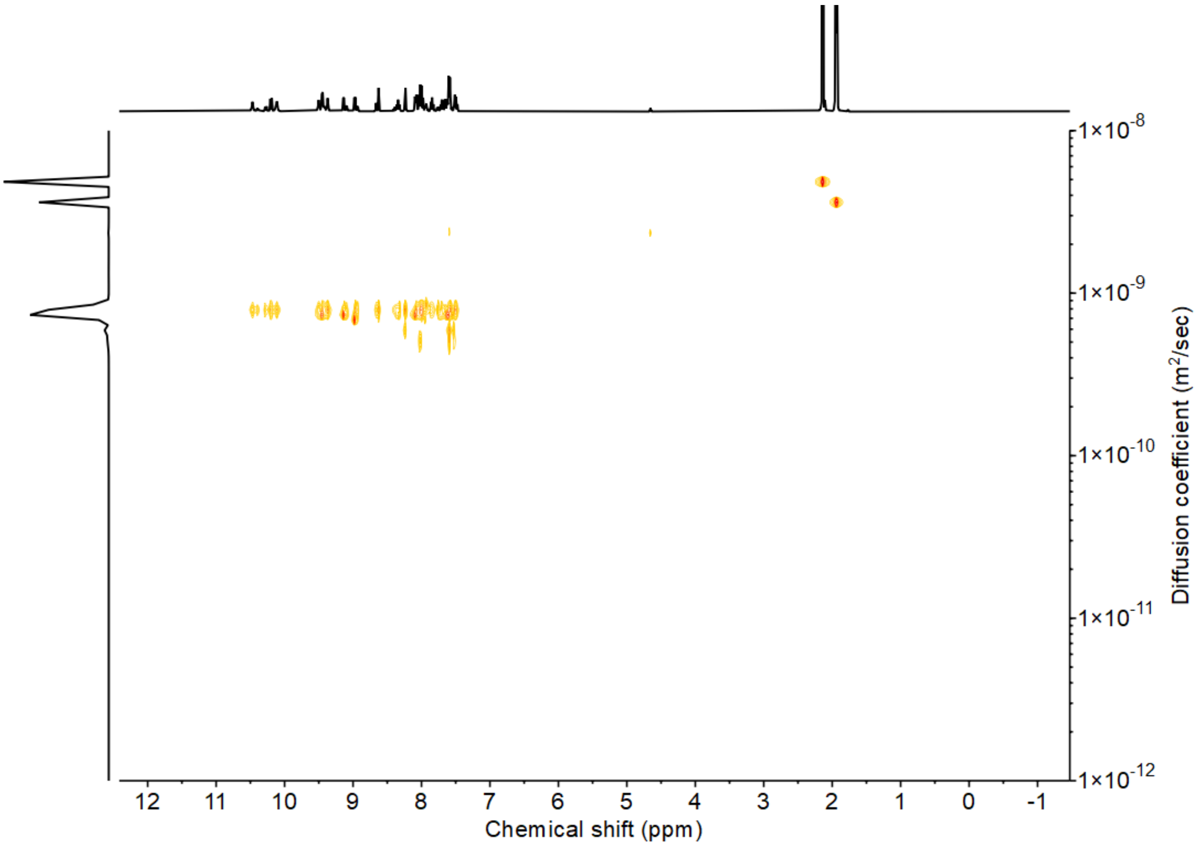

Figure S82 DOSY (CD_3CN) of $[Pd_2(1AB)_2(2AA)_2](BF_4)_4$.

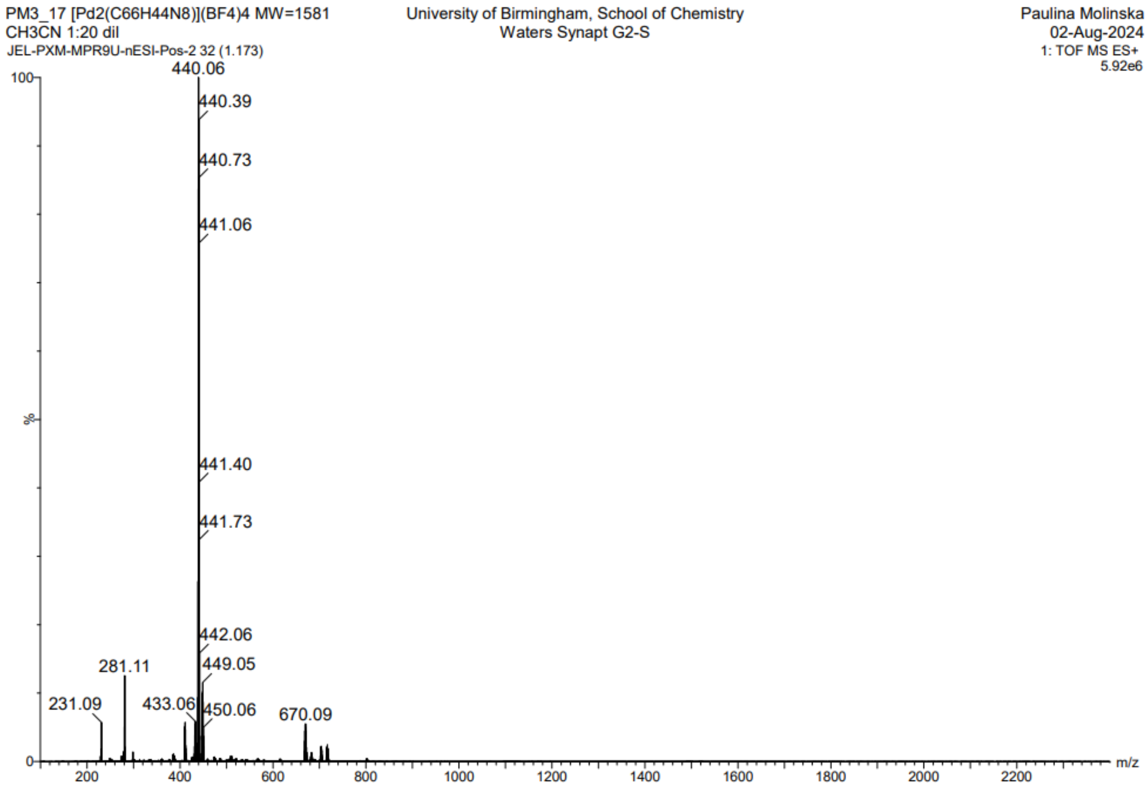

Figure S83 ESI-MS of $[Pd_2(1AB)_2(2AA)_2](BF_4)_4$.

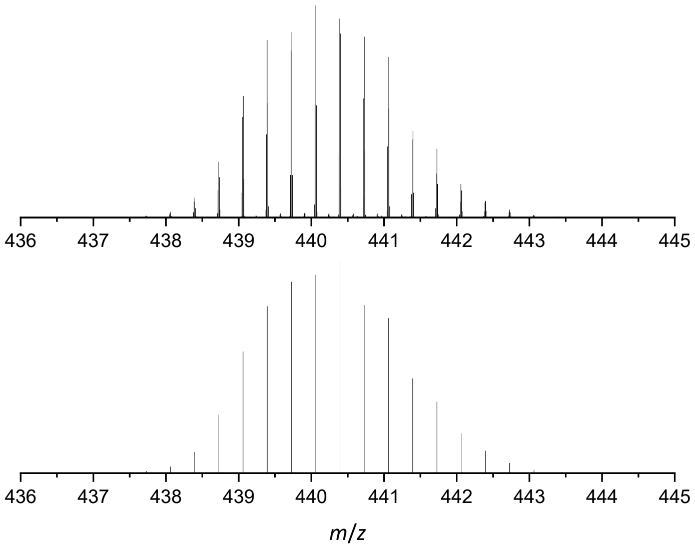

Figure S84 Observed (top) and calculated (bottom) isotopic patterns for $\{[Pd_2(1AB)_2(2AA)_2](BF_4)\}^{3+}$.

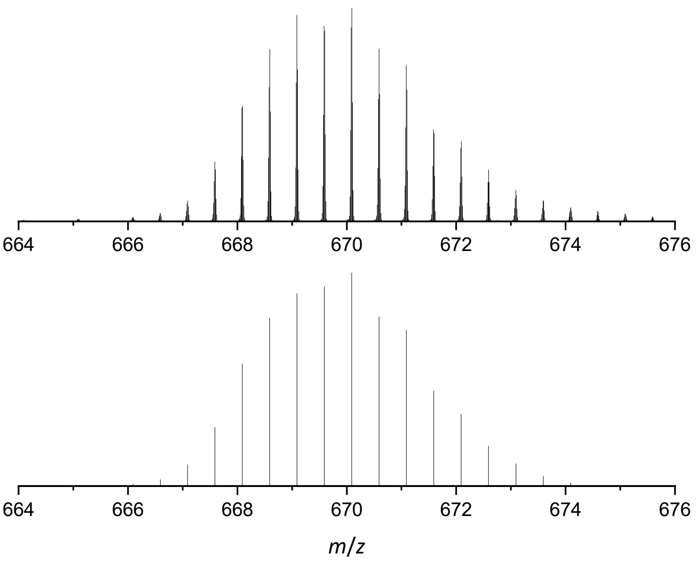

Figure S85 Observed (top) and calculated (bottom) isotopic patterns for $\{[Pd_2(1AB)_2(2AA)_2](BF_4)F\}^{2+}$.

Synthesis of $[Pd_2(\mathbf{1AB})_2(\mathbf{3AA})_2](BF_4)_4$

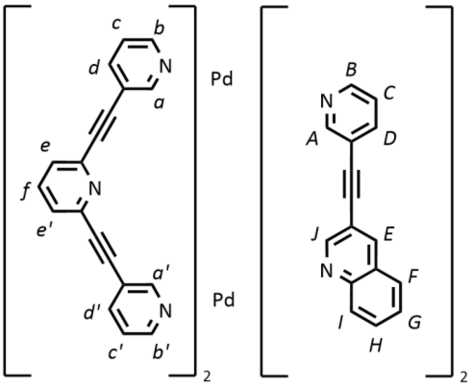

1AB (5.8 mg, 25 µmol, 1 eq.), **3AA** (7.0 mg, 25 µmol, 1 eq.) and a 5.5 mM stock solution of $[Pd(CH_3CN)_4](BF_4)_2$ in CH_3CN (5000 µL, 27.5 µmol, 1.1 eq.) were stirred at 70 °C under N_2 for 24 h. To the cooled reaction mixture was added Et_2O (25 mL) and stirred for 10 mins. The suspension was filtered through a sintered funnel and the precipitate washed with Et_2O (x3). The precipitate was re-dissolved in CH_3CN, and the solvent removed *in vacuo* to give the product as a beige solid (9.8 mg, 49%).

ESI-MS *m/z* = 441.04 $\{[Pd_2(\mathbf{1AB})_2(\mathbf{3AA})_2](BF_4)\}^{3+}$ calc. 441.06; 705.07 $\{[Pd_2(\mathbf{1AB})_2(\mathbf{3AA})_2](BF_4)_2\}^{2+}$ calc. 705.09.

^{13}C NMR (101 MHz, CD_3CN) δ: 157.17 (*syn*-C_J, *anti*-C_J), 155.72 (*syn*-$C_{a'}$/*anti*-$C_{a'}$/*syn*-C_a/*anti*-C_a), 155.57 (*syn*-$C_{a'}$/*anti*-$C_{a'}$/*syn*-C_a/*anti*-C_a), 155.05 (*syn*-C_A), 151.91 (*syn*-C_B), 151.70 (*syn*-C_b), 151.44 (*anti*-C_B), 151.34 (*anti*-$C_{b'}$), 151.03 (*syn*-$C_{b'}$), 146.16, 146.05, 143.88 (*syn*-C_E, *anti*-C_E), 143.73 (*syn*-C_D), 143.64 (*anti*-$C_{d'}$), 143.27, 143.14 , 143.11 (syn-C_d /syn-$C_{d'}$), 143.05 (syn-C_d /syn-$C_{d'}$), 142.65 (*anti*-C_d), 138.98 (*syn*-C_f), 136.00 (*anti*-C_H), 135.64 (*syn*-C_H), 131.13 (syn-C_F /syn-C_G /*anti*-C_G), 131.00 (syn-C_F, *anti*-C_F /syn-C_G), 130.15, 130.05, 128.83 (*syn*-$C_{c'}$, *anti*-$C_{c'}$), 128.69 (*syn*-C_c), 128.58 (*anti*-C_C), 128.38 (*syn*-C_C), 127.66 (*syn*-C_e, *syn*-$C_{e'}$), 127.56 (*anti*-C_I), 127.40 (*syn*-C_I), 124.62, 124.46, 124.23, 119.34, 119.18, 117.14, 95.01, 94.93, 94.85, 91.44, 91.22, 90.03, 83.73, 83.42.

^{19}F NMR (376 MHz, CD_3CN) δ: -144.41, -151.23.

^{1}H DOSY (400 MHz, CD_3CN) D: 7.4 × 10^{-10} m^2s^{-1}; R_S: 8.3 Å.

Major Isomer *syn*-[Pd_2(1AB)$_2$(3AA)$_2$](BF_4)$_4$

^{1}H NMR (400 MHz, CD_3CN) δ: 10.57 (d, *J* = 1.8 Hz, 2H, H_J), 10.21 (m, 4H, H_A, H_I), 9.58 (d, *J* = 1.7 Hz, 2H, H_a), 9.51 (m, 2H, H_b), 9.44 (s, 2H, $H_{a'}$), 9.22 (dd, *J* = 5.8, 1.4 Hz, 2H, $H_{b'}$), 8.99 (dd, *J* = 5.9, 1.3 Hz, 2H, H_B), 8.61 (s, 2H, H_E), 8.35 (ddd, *J* = 8.6, 7.1, 1.4 Hz, 2H, H_H), 8.10-7.97 (m, 8H, H_d, $H_{d'}$, H_D, H_F), 7.89-7.81 (m, 4H, H_G, H_f), 7.72 (ddd, *J* = 8.1, 5.8, 0.7 Hz, 2H, $H_{c'}$), 7.65 (ddd, *J* = 8.0, 5.9, 0.7 Hz, 2H, H_c), 7.63-7.56 (m, 6H, H_C, H_e, $H_{e'}$).

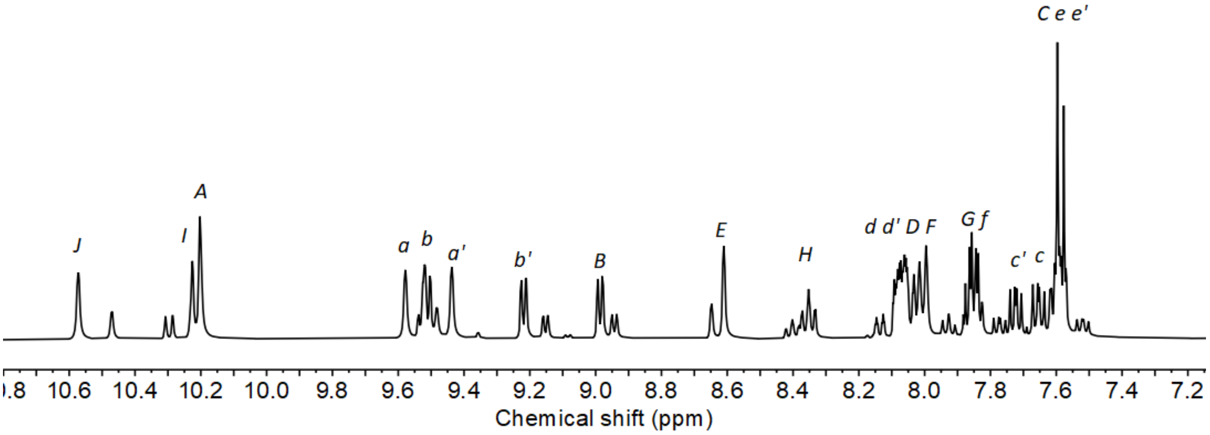

Figure S86 Partial ^{1}H NMR (400 MHz, CD_3CN) of [Pd_2(1AB)$_2$(3AA)$_2$](BF_4)$_4$ with peaks of major *syn*-isomer labelled.

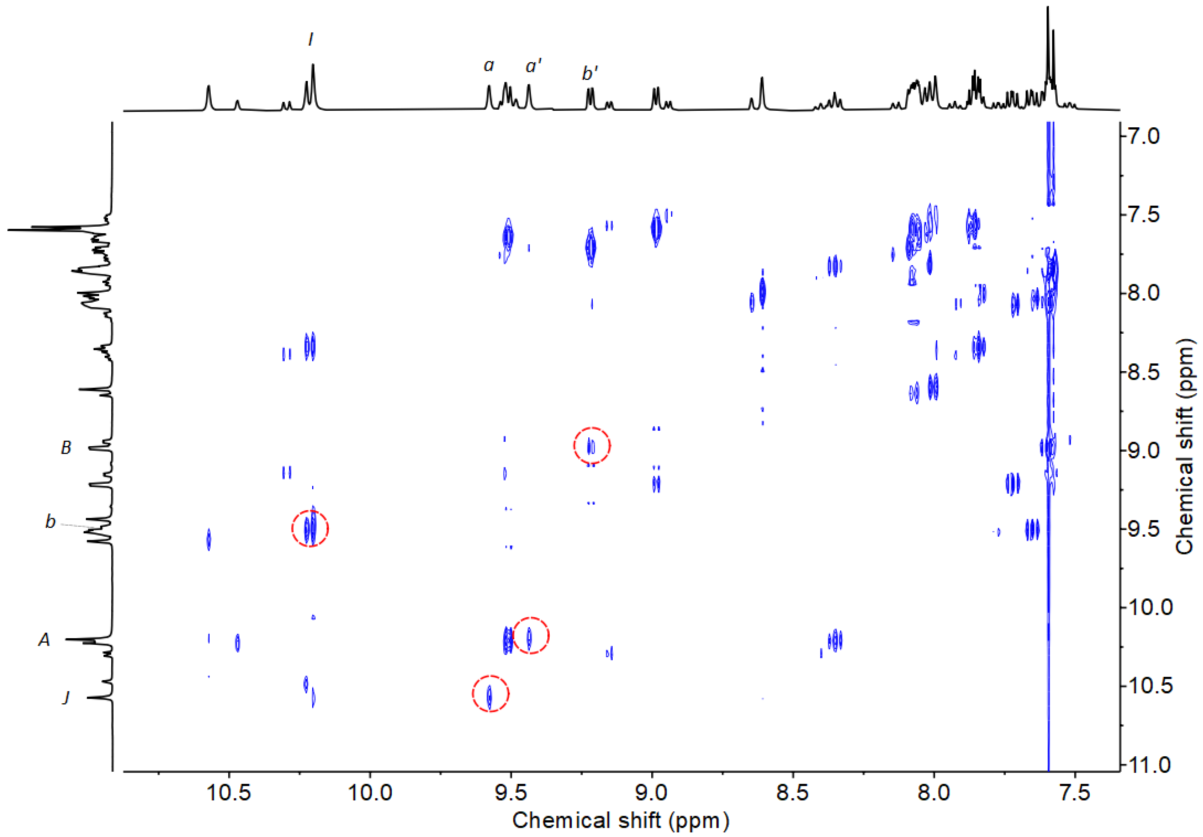

Figure S87 Partial NOESY (400 MHz, CD_3CN) of [Pd_2(1AB)$_2$(3AA)$_2$](BF_4)$_4$ with peaks assigned to major *syn*-isomer.

Minor Isomer *anti*-$[Pd_2(1AB)_2(3AA)_2](BF_4)_4$

^{1}H NMR (400 MHz, CD_3CN) δ: 10.47 (d, J = 1.7 Hz, 2H, H_J), 10.30 (d, J = 8.6 Hz, 2H, H_I), 10.21 (app. d, 2H, H_A), 9.55-9.47 (m, 6H, H_a, H_b, $H_{a'}$), 9.15 (dd, J = 5.4, 1.0 Hz, 2H, $H_{b'}$), 8.94 (dd, J = 5.8, 1.4 Hz, 2H, H_B) 8.65 (s, 2H, H_E), 8.40 (ddd, J = 8.7, 7.1, 1.4 Hz, 2H, H_H), 8.14 (dt, J = 8.1, 1.6 Hz, 2H, $H_{d'}$), 8.10-7.97 (m, 6H, H_d, H_D, H_F), 7.93 (t, J = 7.6 Hz, 2H, H_G), 7.89-7.81 (m, 2H, H_f), 7.77 (dd, J = 8.0, 5.9 Hz, 2H, $H_{c'}$), 7.65 (ddd, J = 8.0, 5.9, 0.7 Hz, 2H, H_c), 7.63-7.56 (m, 4H, H_e, $H_{e'}$), 7.52 (dd, J = 8.0, 5.8 Hz, 2H, H_C).

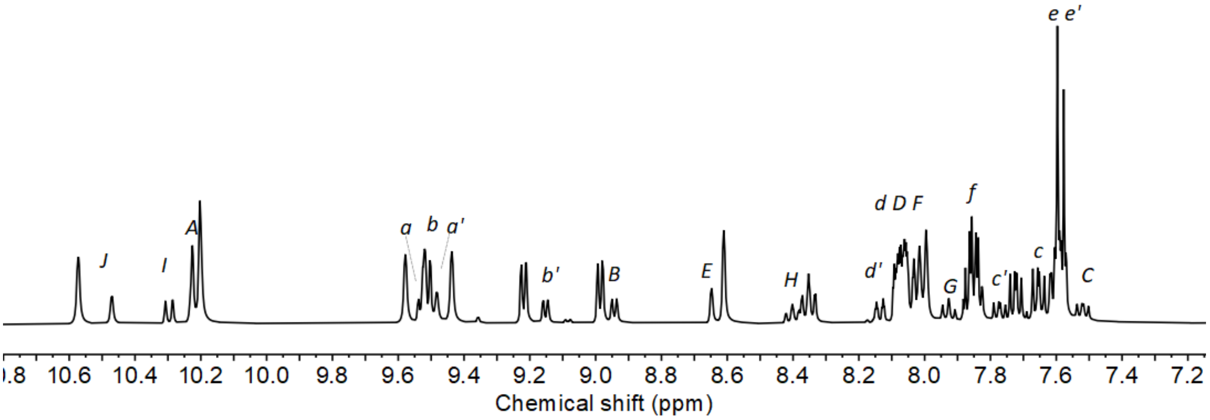

Figure S88 Partial ^{1}H NMR (400 MHz, CD_3CN) of $[Pd_2(1AB)_2(3AA)_2](BF_4)_4$ with peaks of minor *anti*-isomer labelled.

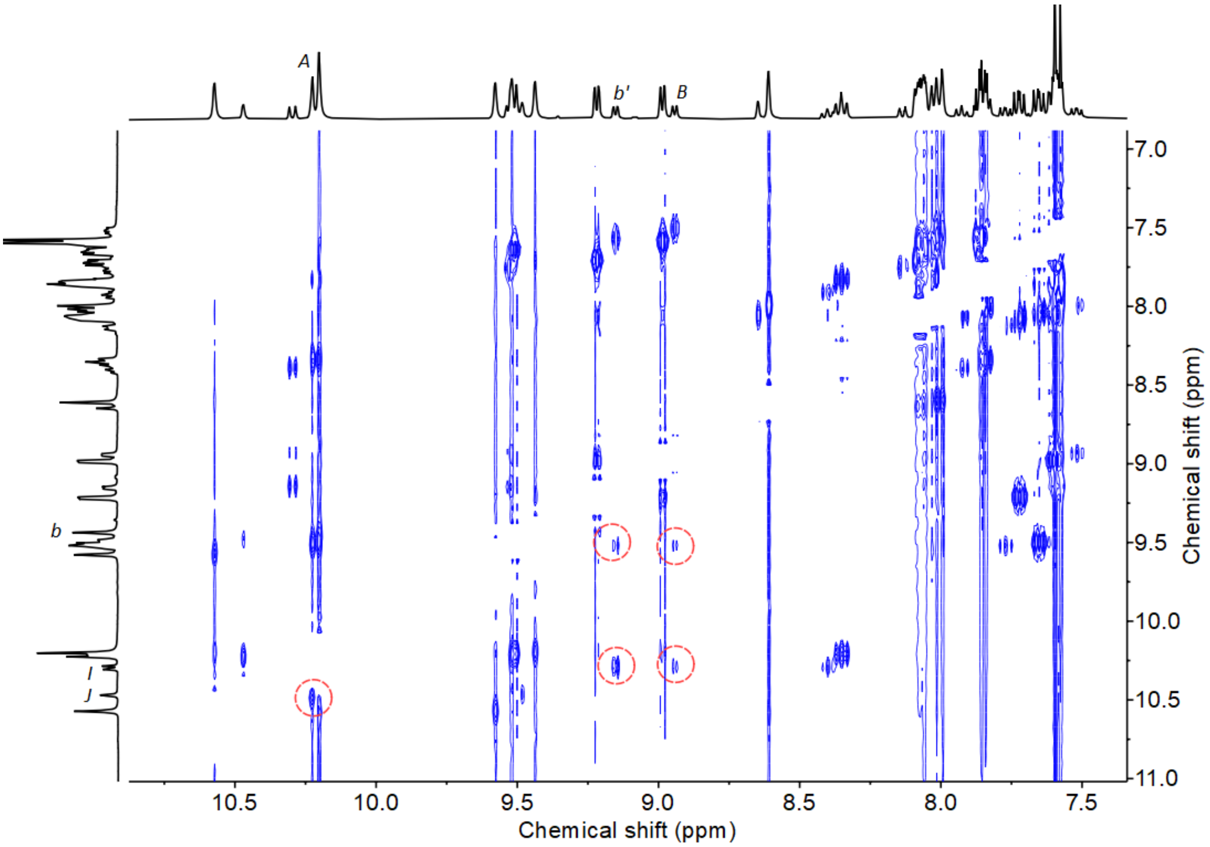

Figure S89 Partial NOESY (400 MHz, CD_3CN) of $[Pd_2(1AB)_2(3AA)_2](BF_4)_4$ with peaks assigned to minor *anti*-isomer.

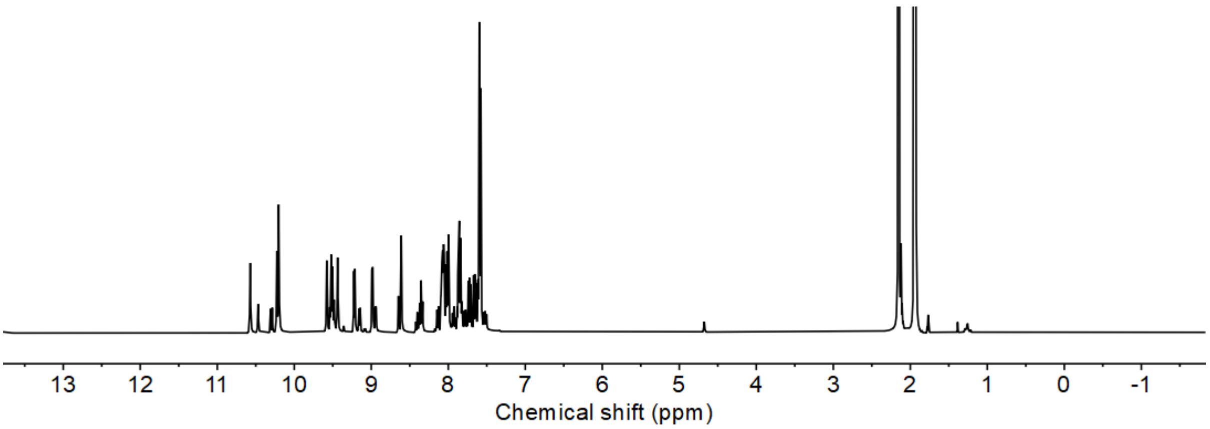

Figure S90 ^{1}H NMR (400 MHz, CD_3CN) of $[Pd_2(1AB)_2(3AA)_2](BF_4)_4$.

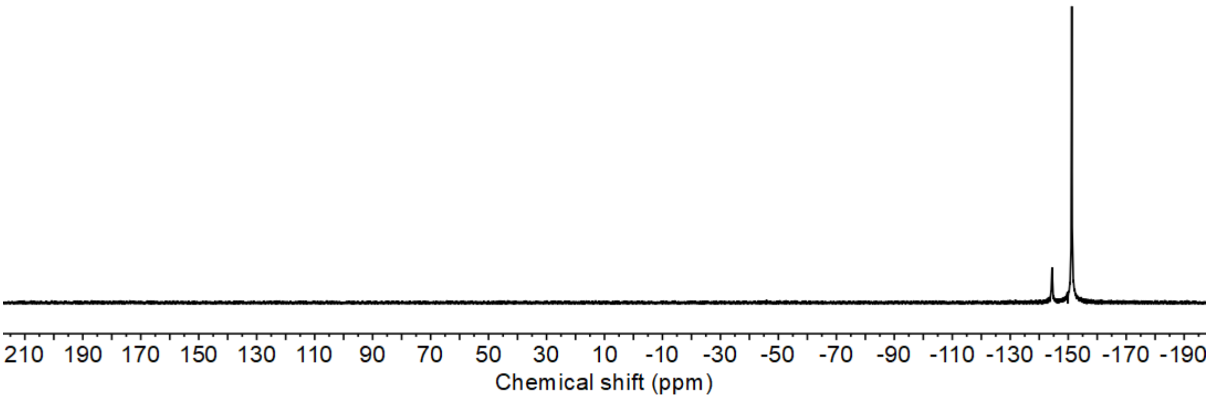

Figure S91 ^{19}F NMR (376 MHz, CD_3CN) of $[Pd_2(1AB)_2(3AA)_2](BF_4)_4$.

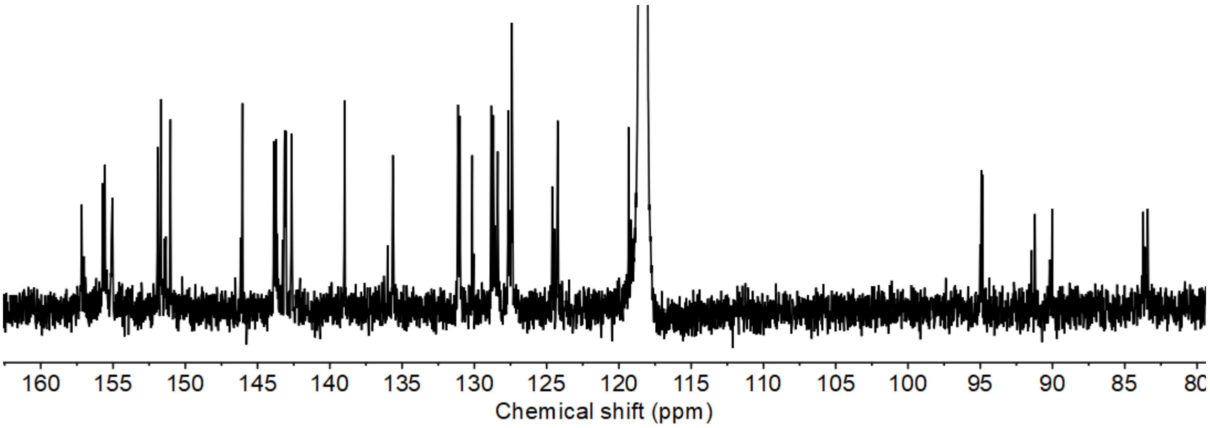

Figure S92 Partial ^{13}C NMR (101 MHz, CD_3CN) of $[Pd_2(1AB)_2(3AA)_2](BF_4)_4$.

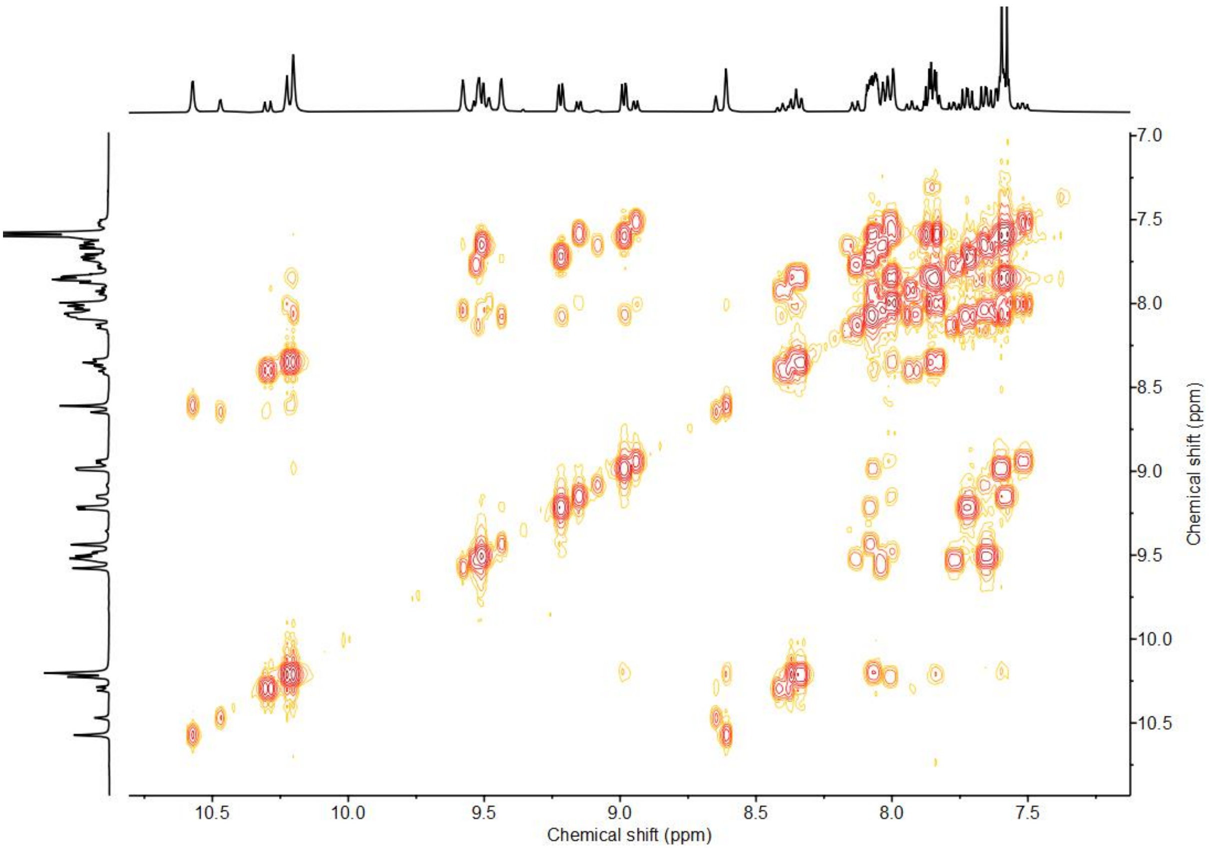

Figure S93 Partial COSY (CD_3CN) of $[Pd_2(1AB)_2(3AA)_2](BF_4)_4$.

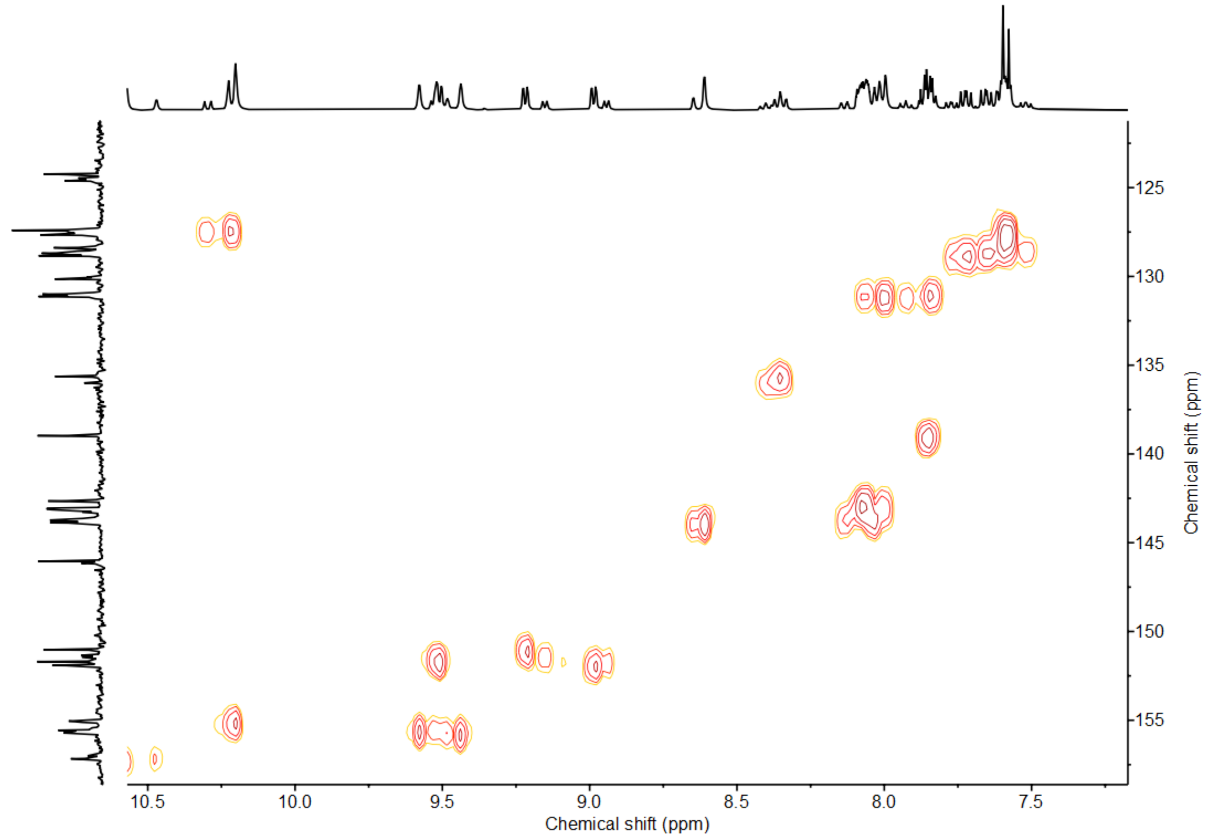

Figure S94 Partial HSQC (CD_3CN) of $[Pd_2(1AB)_2(3AA)_2](BF_4)_4$.

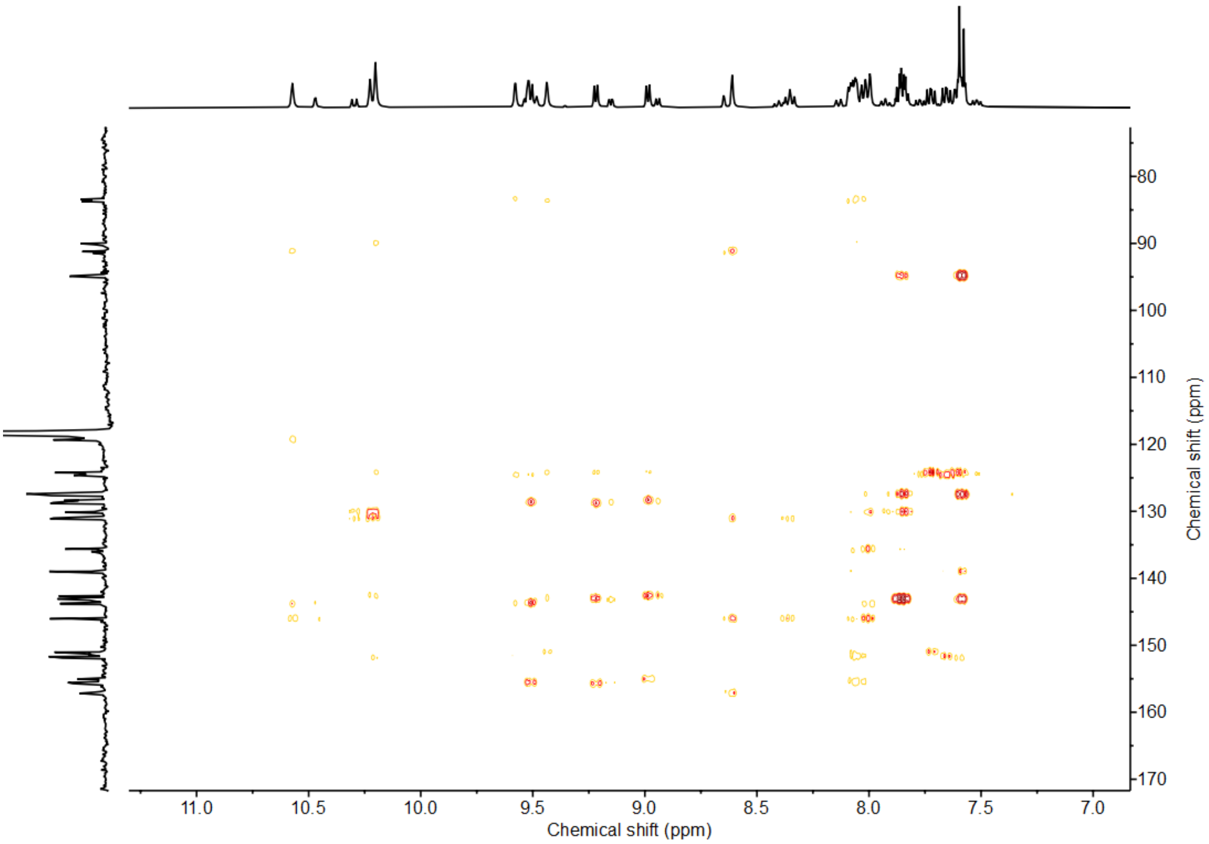

Figure S95 Partial HMBC (CD_3CN) of $[Pd_2(1AB)_2(3AA)_2](BF_4)_4$.

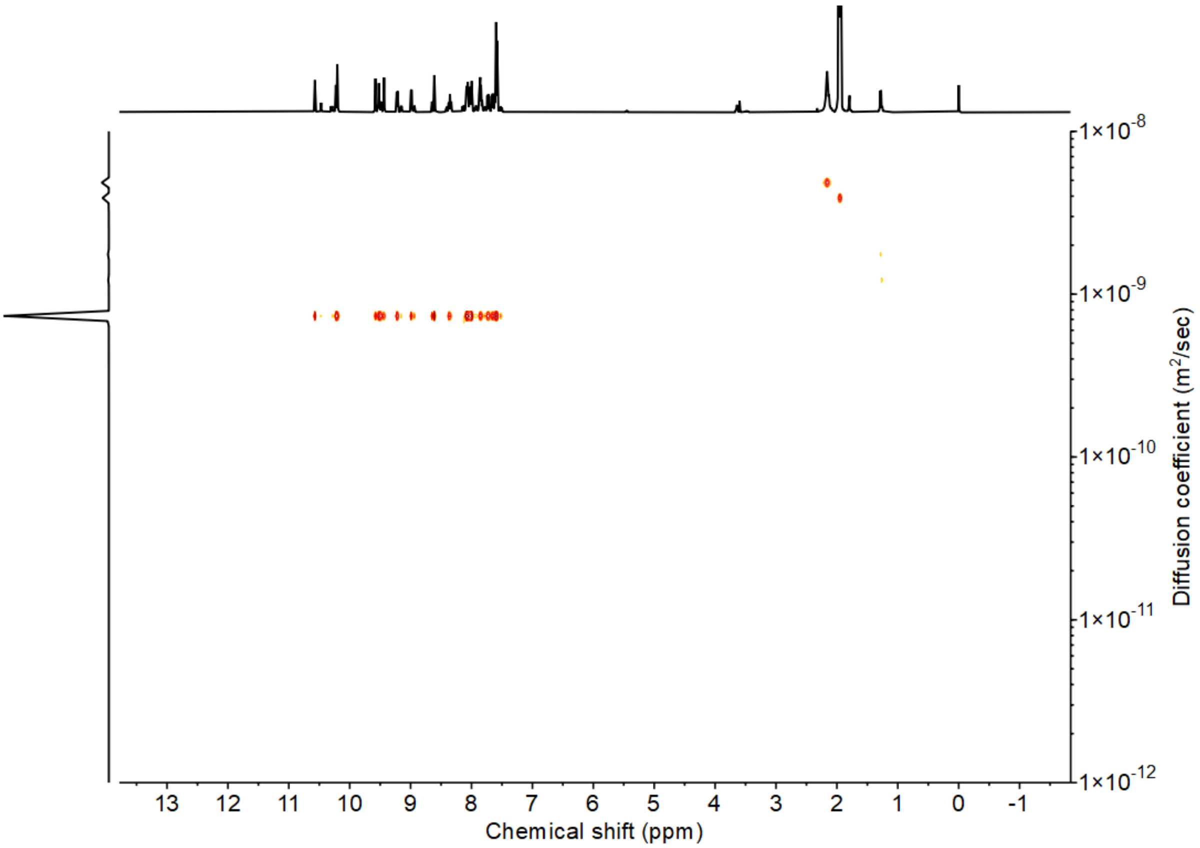

Figure S96 DOSY (CD_3CN) of $[Pd_2(1AB)_2(3AA)_2](BF_4)_4$.

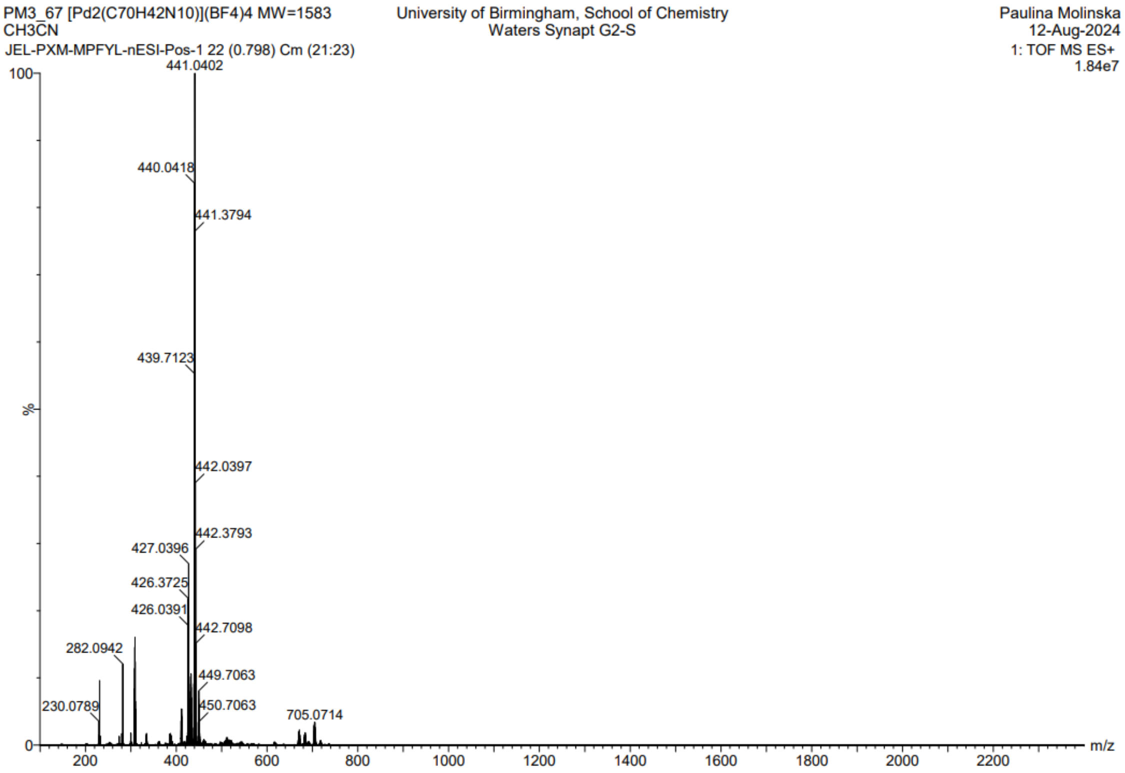

Figure S97 ESI-MS of $[Pd_2(1AB)_2(3AA)_2](BF_4)_4$.

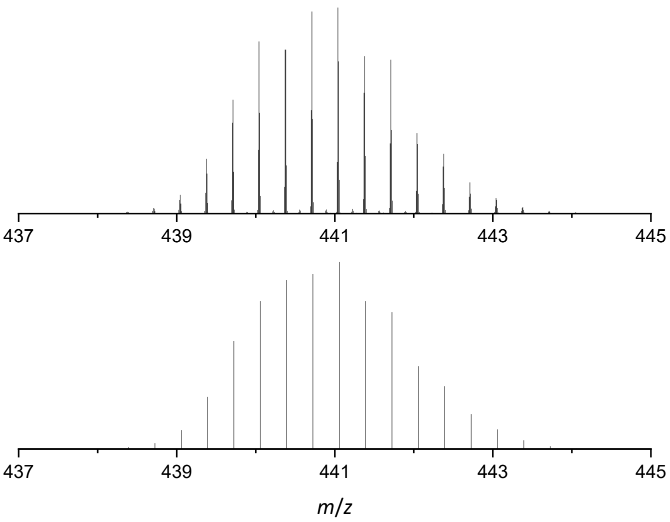

Figure S98 Observed (top) and calculated (bottom) isotopic patterns for $\{[Pd_2(1AB)_2(3AA)_2](BF_4)\}^{3+}$.

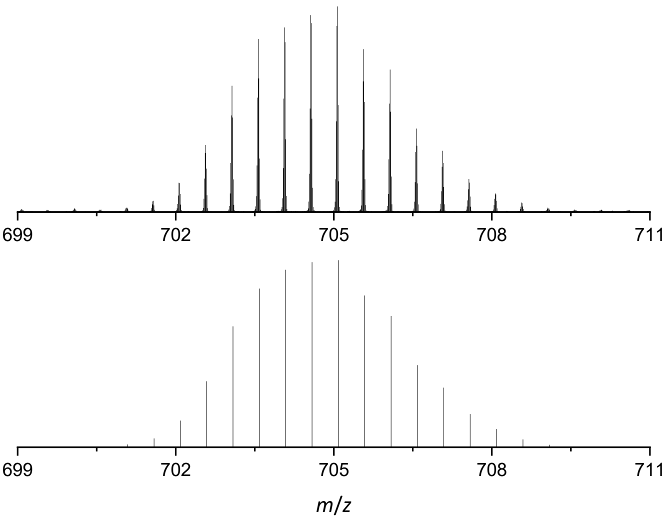

Figure S99 Observed (top) and calculated (bottom) isotopic patterns for $\{[Pd_2(1AB)_2(3AA)_2](BF_4)_2\}^{2+}$.

Synthesis of $[Pd_2(1AB)_2(4AA)_2](BF_4)_4$

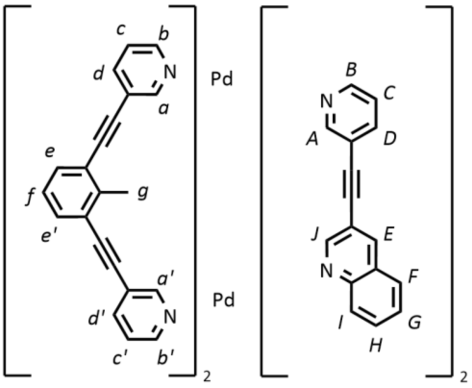

1AB (5.8 mg, 25 µmol, 1 eq.), **4AA** (7.4 mg, 25 µmol, 1 eq.) and a 5.5 mM stock solution of $[Pd(CH_3CN)_4](BF_4)_2$ in CH_3CN (5000 µL, 27.5 µmol, 1.1 eq.) were stirred at 70 °C under N_2 for 24 h. To the cooled reaction mixture was added Et_2O (20 mL) and stirred for 10 mins. The suspension was filtered through a sintered funnel and the precipitate washed with Et_2O (x3). The precipitate was re-dissolved in CH_3CN, and the solvent removed *in vacuo* to give the product as a beige solid (16.4 mg, 82%).

ESI-MS *m/z* = 449.73 $\{[Pd_2(\mathbf{1AB})_2(\mathbf{4AA})_2](BF_4)\}^{3+}$ calc. 449.74; 684.11 $\{[Pd_2(\mathbf{1AB})_2(\mathbf{4AA})_2](BF_4)F\}^{2+}$ calc. 684.11; 718.11 $\{[Pd_2(\mathbf{1AB})_2(\mathbf{4AA})_2](BF_4)_2\}^{2+}$ calc. 718.11.

^{13}C NMR (101 MHz, CD_3CN) δ: 157.15 (*syn*-C_J), 155.13 (*syn*-$C_{a'}$), 154.96 (*anti*-C_a, *anti*-$C_{a'}$), 154.84 (*syn*-C_a, *anti*-C_A), 154.75 (*syn*-C_A), 151.97 (*syn*-C_B), 150.94 (*syn*-C_b), 150.75 (*anti*-C_b), 150.26 (*syn*-$C_{b'}$), 147.47, 146.09, 143.93 (*syn*-C_E, *anti*-C_E), 142.88 (*syn*-C_d/ *syn*-$C_{d'}$), 142.83 (*syn*-C_d /*syn*-$C_{d'}$), 142.75, 142.11 (*syn*-C_D), 135.49 (*syn*-C_H), 132.97, 132.57, 131.08 (*syn*-C_F /*anti*-C_F), 130.94 (*syn*-C_G /*anti*-C_G), 130.15, 128.91 (*syn*-$C_{c'}$), 128.71 (*syn*-$C_{c'}$), 128.45 (*anti*-$C_{c'}$), 128.27 (*syn*-C_C), 127.41 (*anti*-C_I), 127.34 (*syn*-C_I, *syn*-C_f), 125.43, 125.06, 124.46, 122.92, 122.87, 119.44, 95.32, 91.02, 90.39, 89.89, 89.66, 21.31(*syn*-C_g, *anti*-C_g).

^{19}F NMR (376 MHz, CD_3CN) δ: -151.34.

^{1}H DOSY (400 MHz, CD_3CN) *D*: 6.8 × 10^{-10} m^2s^{-1}; R_S: 9.0 Å.

Major Isomer *syn*-[$Pd_2(1AB)_2(4AA)_2$]$(BF_4)_4$

^{1}H NMR (400 MHz, CD_3CN) δ: 10.61 (d, *J* = 1.8 Hz, 2H, H_J), 10.18-10.11 (m, 4H, H_I, H_A), 9.50 (dd, *J* = 5.9, 1.3 Hz, 2H, H_b), 9.47 (d, *J* = 1.8 Hz, 2H, H_a), 9.29 (d, *J* = 1.8 Hz, 2H, $H_{a'}$), 9.18 (dd, *J* = 5.9, 1.3 Hz, 2H, $H_{b'}$), 8.95 (dd, *J* = 5.9, 1.3 Hz, 2H, H_B), 8.59 (s, 2H, H_E), 8.31 (ddd, *J* = 8.6, 7.0, 1.4 Hz, 2H, H_H), 8.12-7.96 (m, 8H), 7.82 (m, 2H, H_G), 7.73 (dd, *J* = 8.1, 5.8 Hz, 2H, $H_{c'}$), 7.67 (dd, *J* = 8.1, 5.8 Hz, 2H, H_c), 7.60-7.56 (m, 2H, H_C), 7.55-7.50 (m, 4H, H_e, $H_{e'}$), 7.28 (m, 2H, H_f), 3.21 (s, 6H, H_g).

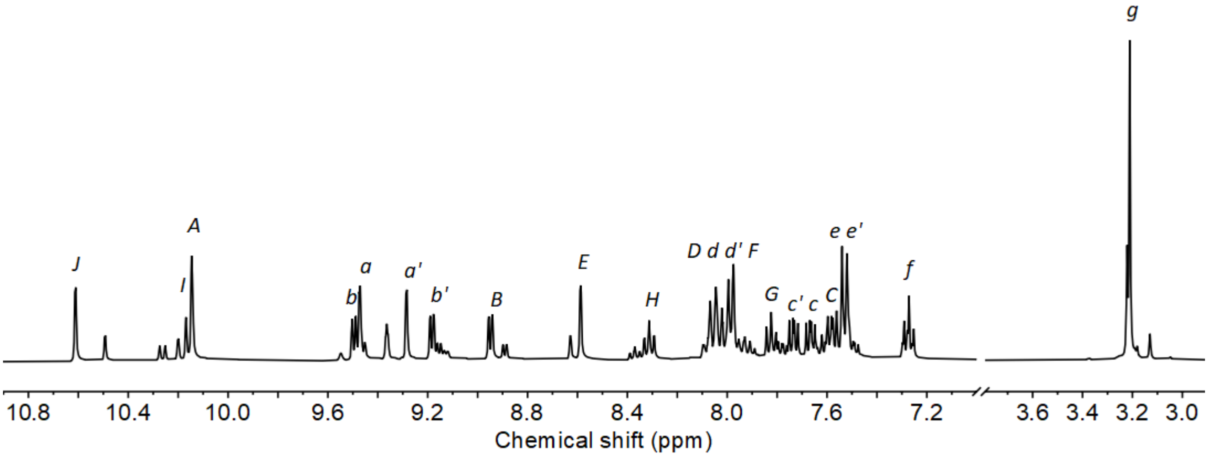

Figure S100 Partial ^{1}H NMR (400 MHz, CD_3CN) of [$Pd_2(1AB)_2(4AA)_2$]$(BF_4)_4$ with peaks of major *syn*-isomer labelled.

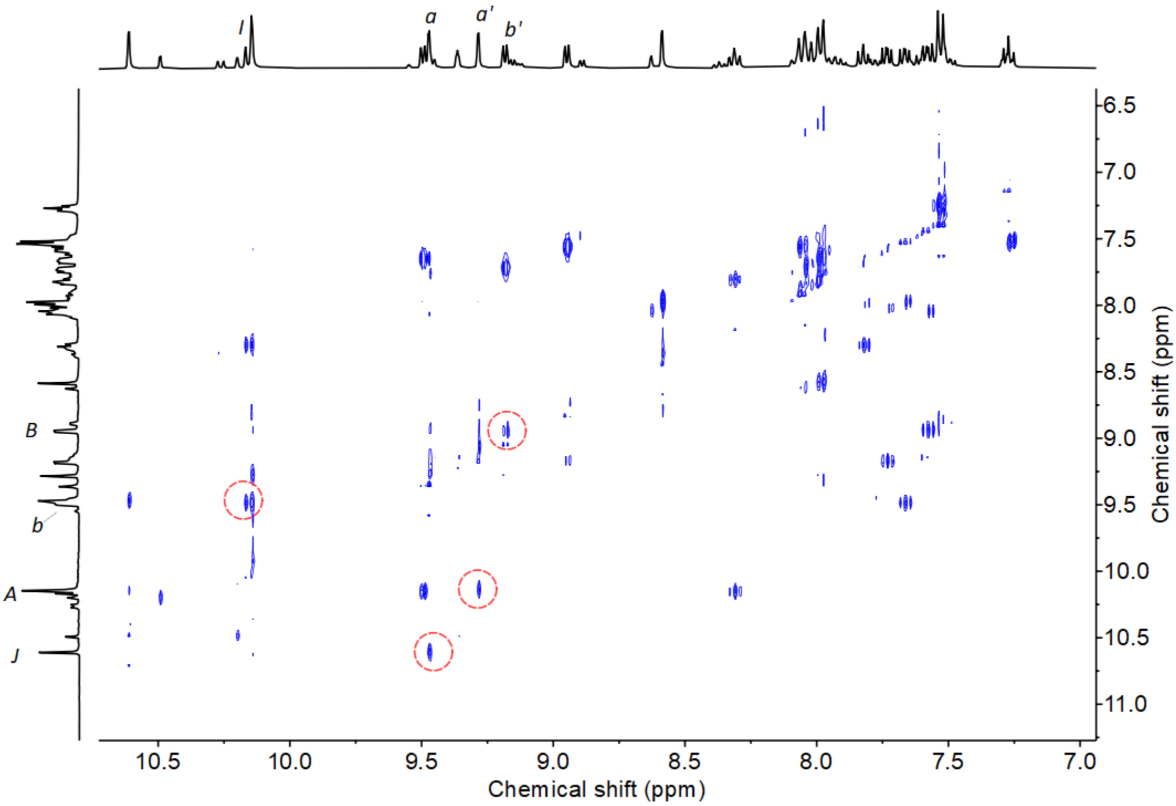

Figure S101 Partial NOESY (400 MHz, CD_3CN) of [$Pd_2(1AB)_2(4AA)_2$]$(BF_4)_4$ with peaks assigned to major *syn*-isomer.

Minor Isomer *anti*-$[Pd_2(1AB)_2(4AA)_2](BF_4)_4$

^{1}H NMR (400 MHz, CD_3CN) δ: 10.49 (d, *J* = 1.7 Hz, 2H, H_J), 10.26 (d, *J* = 8.7 Hz, 2H, H_I), 10.20 (d, *J* = 1.8 Hz, 2H, H_A), 9.45 (d, *J* = 1.2 Hz, 2H, H_b), 9.36 (m, 4H, H_a, $H_{a'}$), 9.15 (dd, *J* = 5.8, 1.1 Hz, 2H, $H_{b'}$), 8.89 (d, *J* = 5.9 Hz, 2H, H_B), 8.63 (d, *J* = 1.6 Hz, 2H, H_E), 8.37 (ddd, *J* = 8.6, 7.0, 1.4 Hz, 2H, H_H), 8.12-7.96 (m, 6H, H_F, H_d, $H_{d'}$), 7.96-7.88 (m, 4H, H_D, H_G), 7.78 (dd, *J* = 8.1, 5.9 Hz, 2H, $H_{c'}$), 7.70-7.55 (m, 4H, H_c, H_C), 7.55-7.46 (m, 4H, H_e, $H_{e'}$), 7.28 (m, 2H, H_f), 3.22 (s, 6H, H_g).

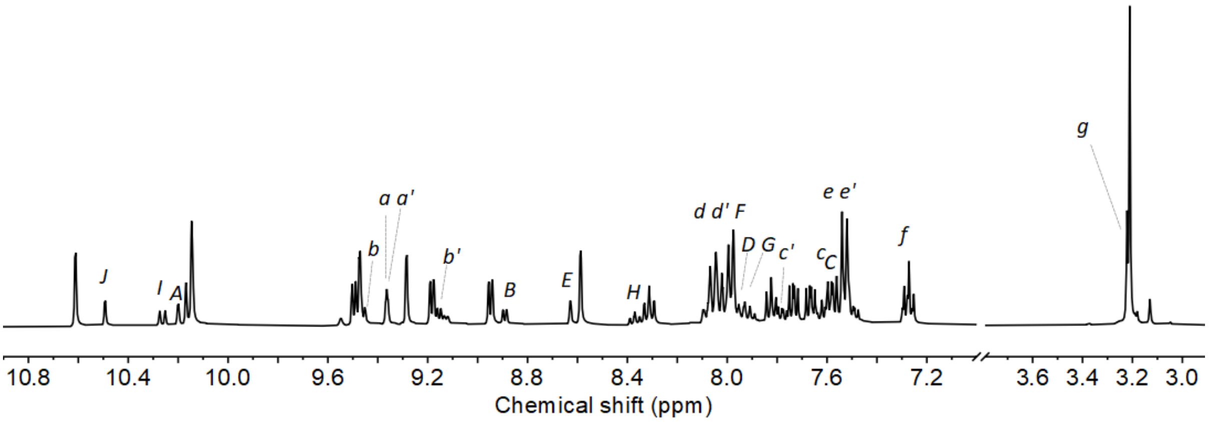

Figure S102 Partial ^{1}H NMR (400 MHz, CD_3CN) of $[Pd_2(1AB)_2(4AA)_2](BF_4)_4$ with peaks of minor *anti*-isomer labelled.

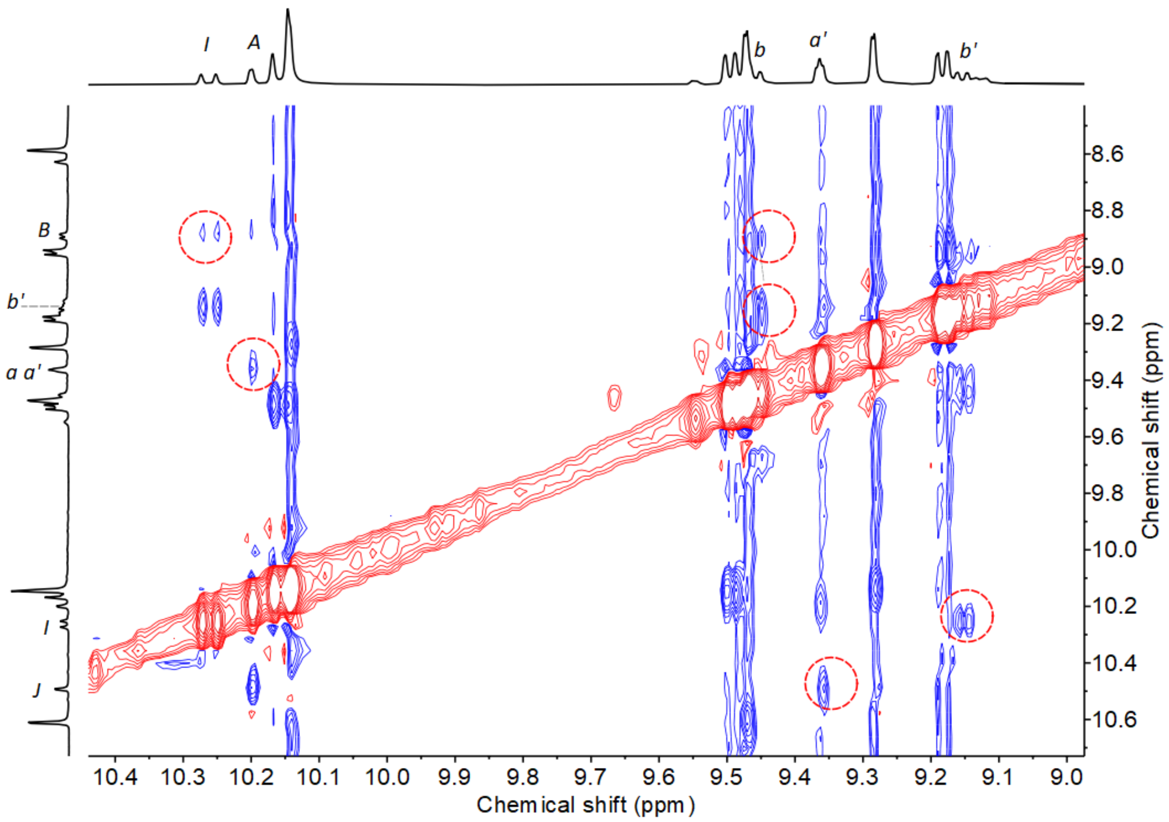

Figure S103 Partial NOESY (400 MHz, CD_3CN) of $[Pd_2(1AB)_2(4AA)_2](BF_4)_4$ with peaks assigned to minor *anti*-isomer.

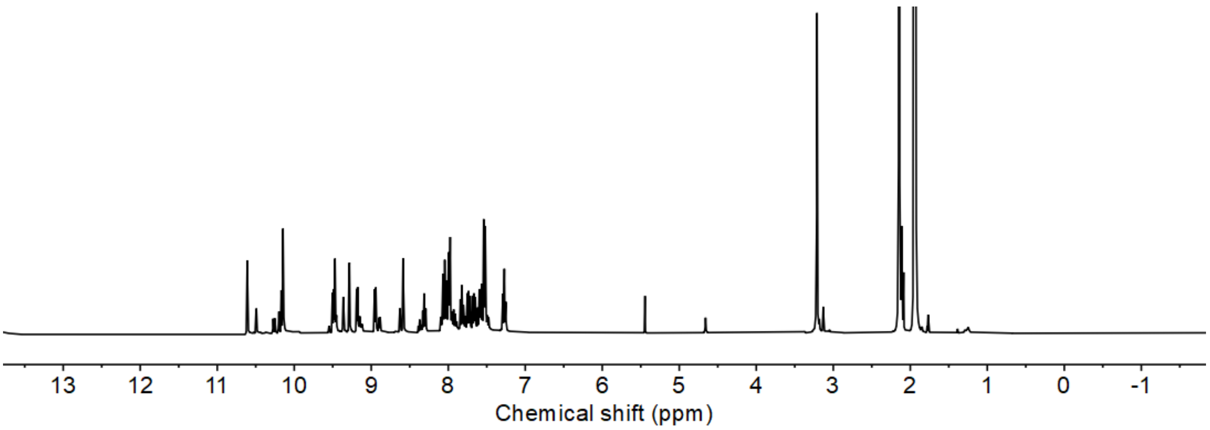

Figure S104 ^{1}H NMR (400 MHz, CD_3CN) of $[Pd_2(1AB)_2(4AA)_2](BF_4)_4$.

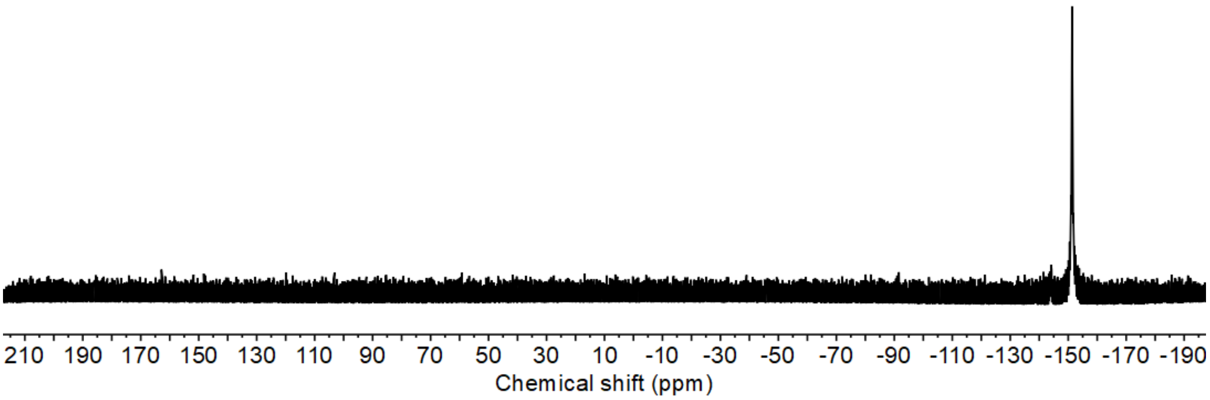

Figure S105 ^{19}F NMR (376 MHz, CD_3CN) of $[Pd_2(1AB)_2(4AA)_2](BF_4)_4$.

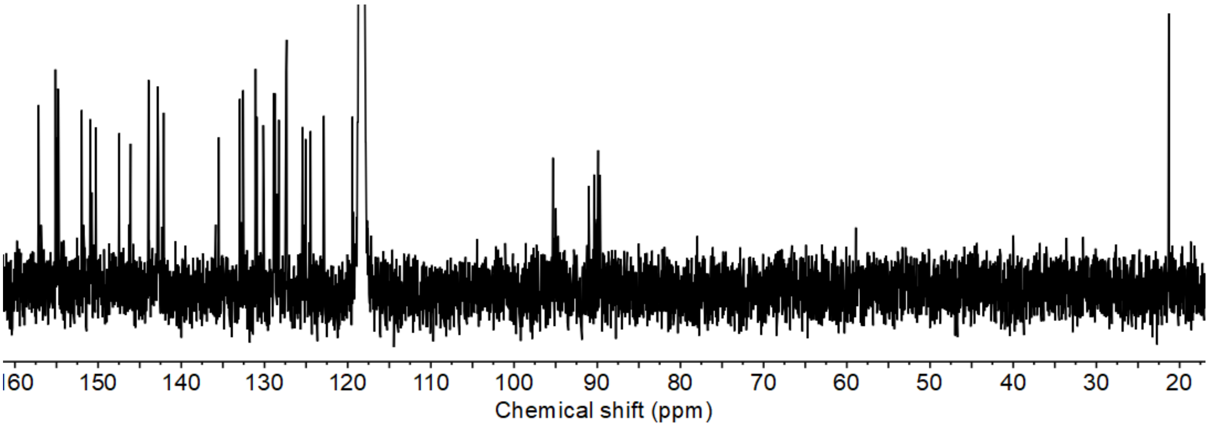

Figure S106 Partial ^{13}C NMR (101 MHz, CD_3CN) of $[Pd_2(1AB)_2(4AA)_2](BF_4)_4$.

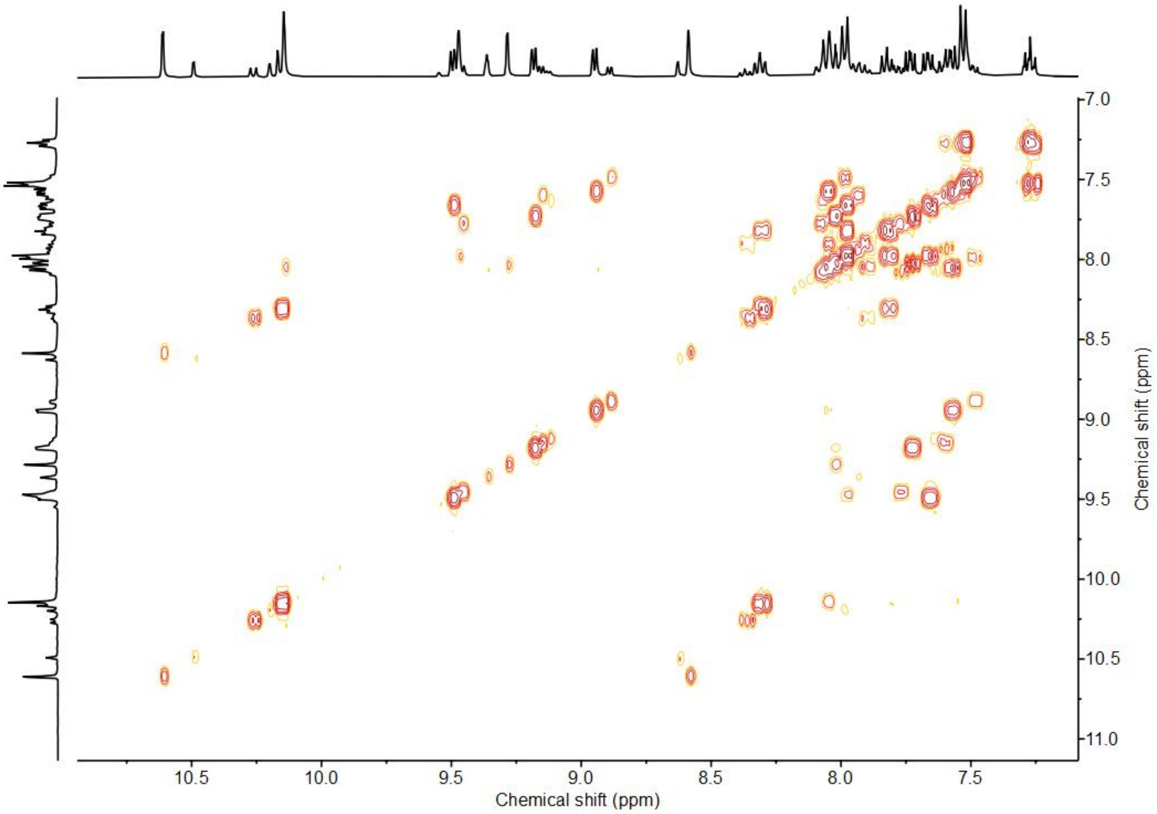

Figure S107 Partial COSY (CD_3CN) of $[Pd_2(1AB)_2(4AA)_2](BF_4)_4$.

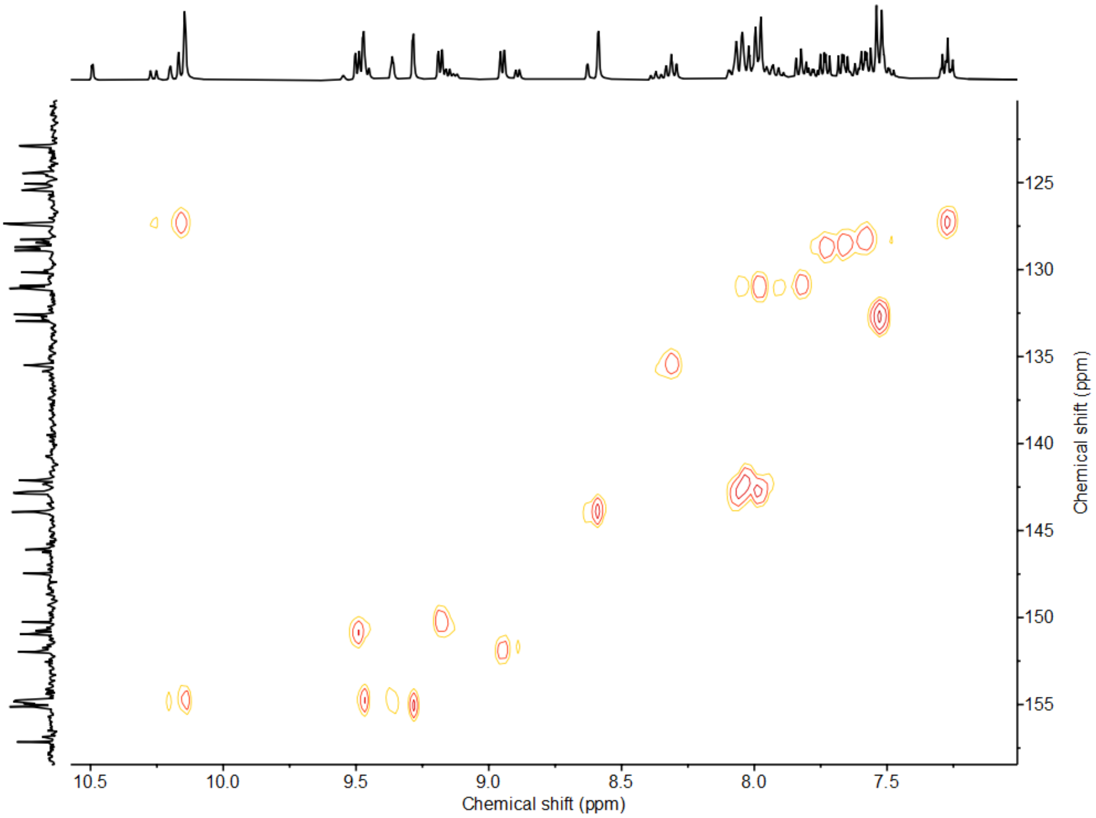

Figure S108 Partial HSQC (CD_3CN) of $[Pd_2(1AB)_2(4AA)_2](BF_4)_4$.

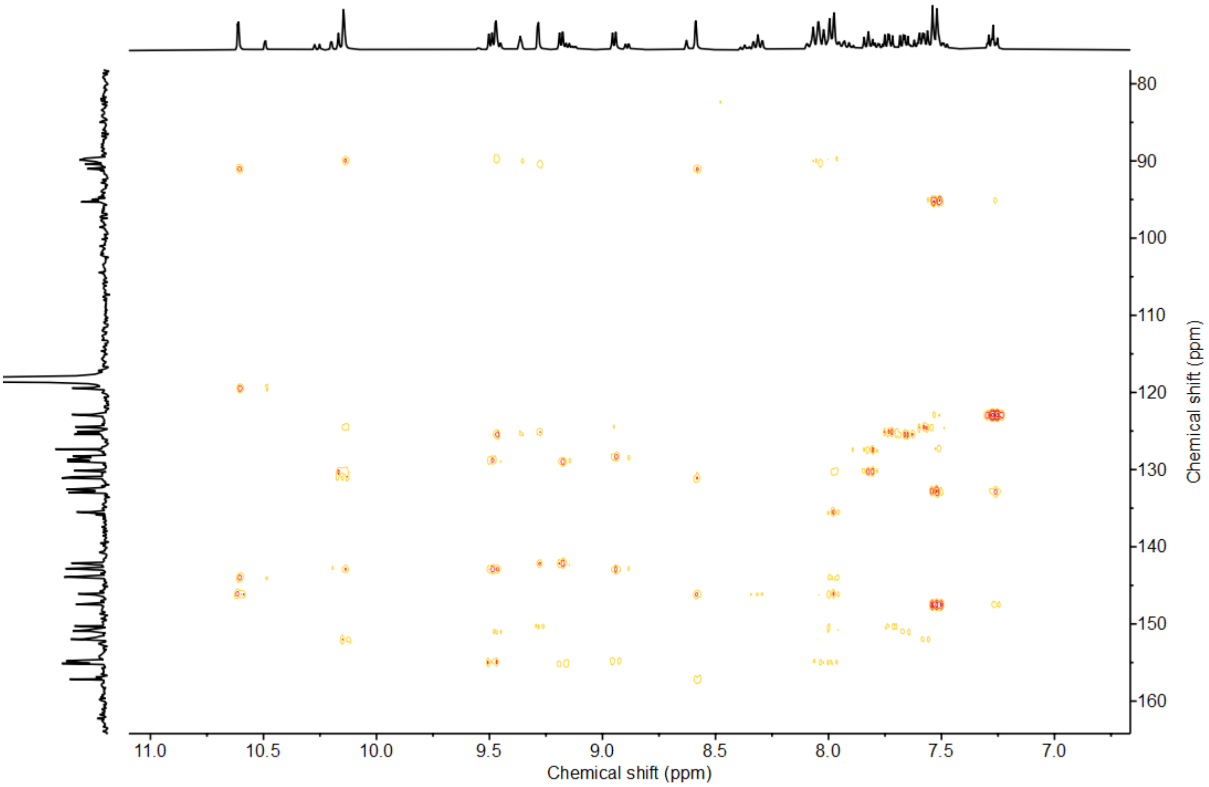

Figure S109 Partial HMBC (CD_3CN) of [$Pd_2(1AB)_2(4AA)_2$](BF_4)$_4$.

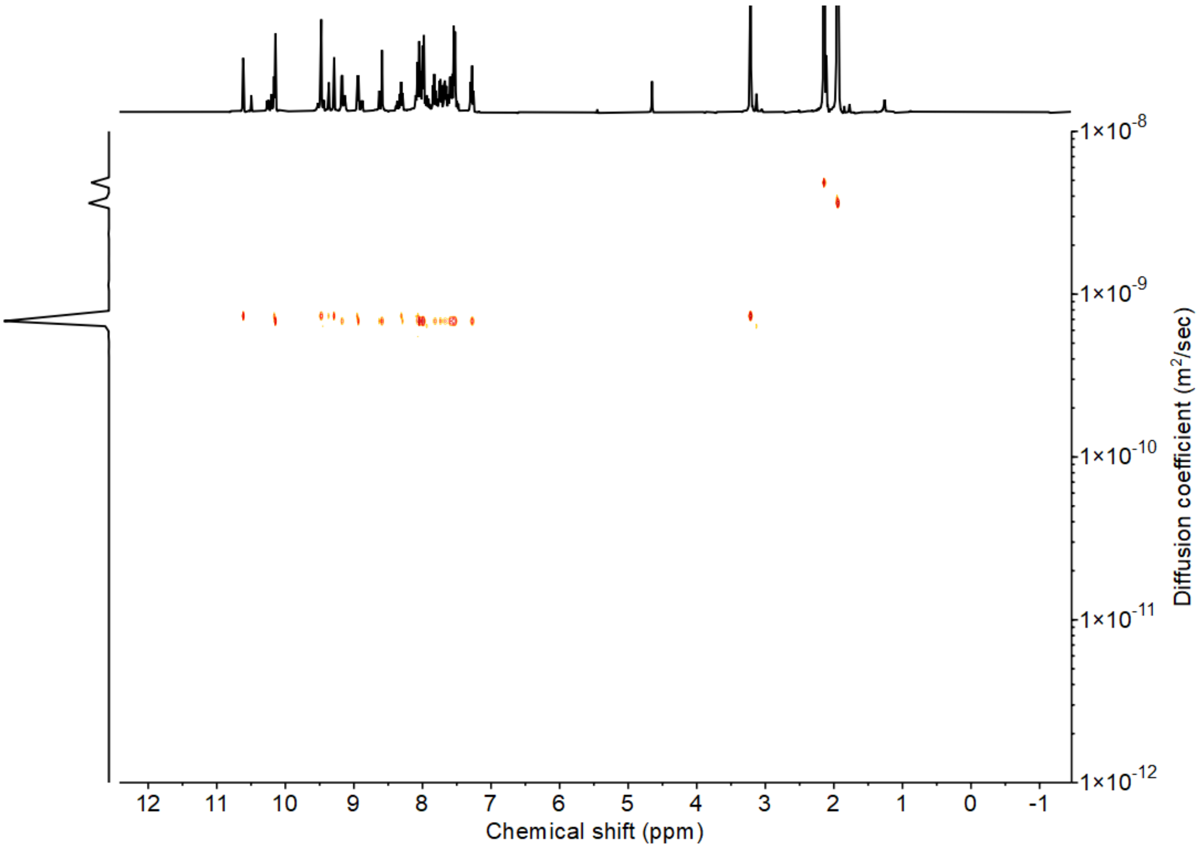

Figure S110 DOSY (CD_3CN) of [$Pd_2(1AB)_2(4AA)_2$](BF_4)$_4$.

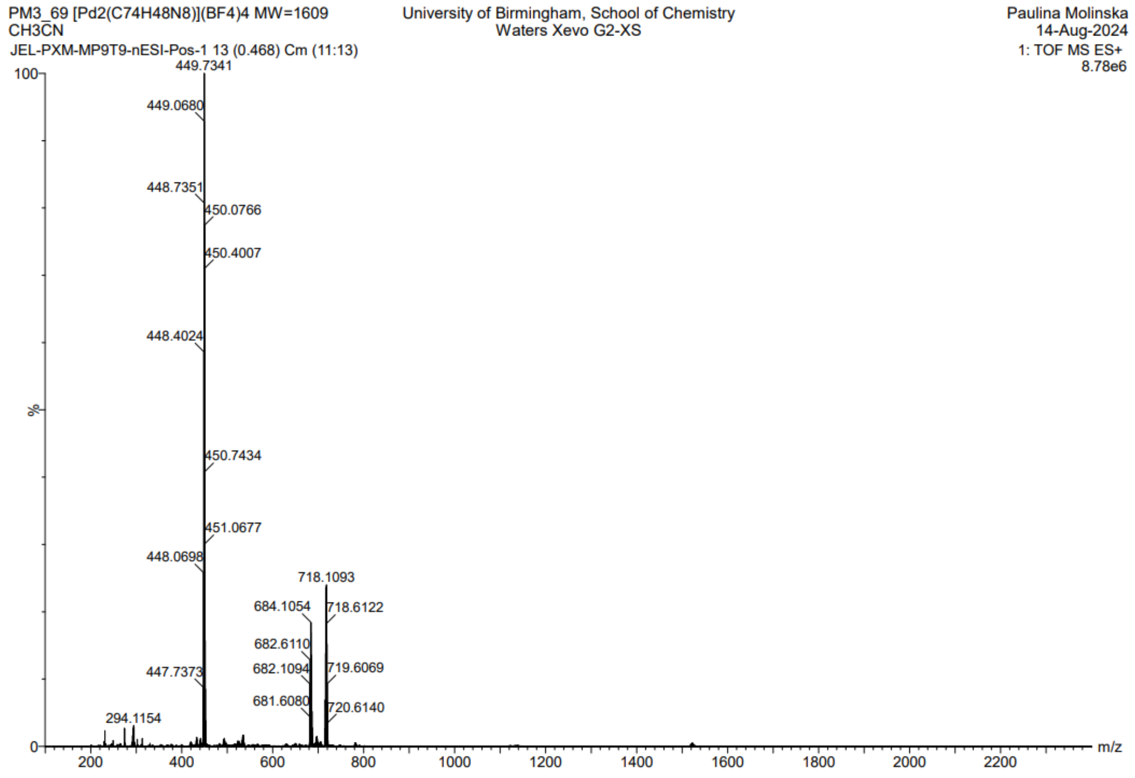

Figure S111 ESI-MS of $[Pd_2(1AB)_2(4AA)_2](BF_4)_4$.

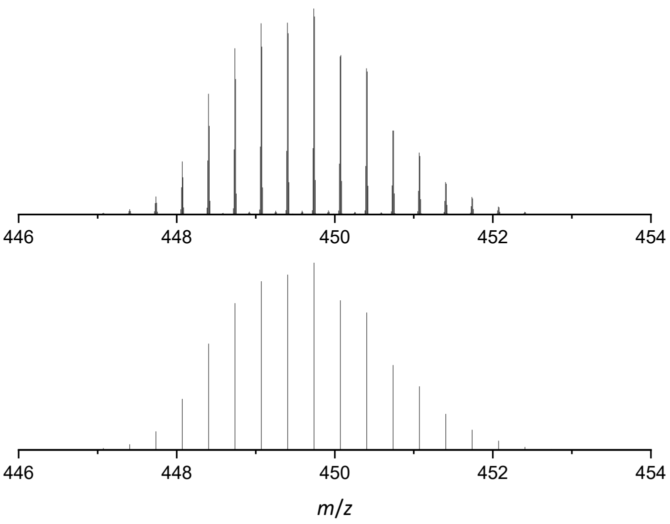

Figure S112 Observed (top) and calculated (bottom) isotopic patterns for $\{[Pd_2(1AB)_2(4AA)_2](BF_4)\}^{3+}$.

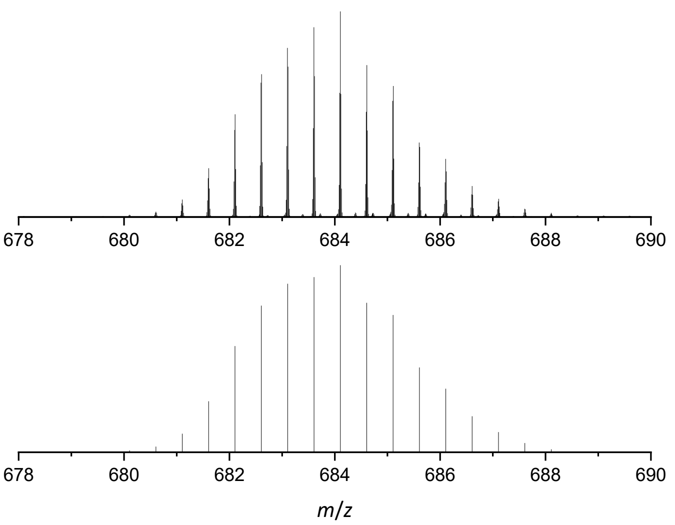

Figure S113 Observed (top) and calculated (bottom) isotopic patterns for $\{[Pd_2(1AB)_2(4AA)_2](BF_4)F\}^{2+}$.

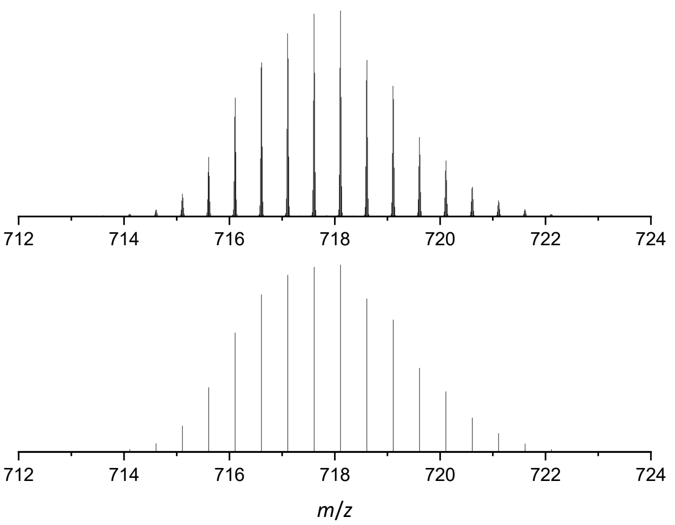

Figure S114 Observed (top) and calculated (bottom) isotopic patterns for $\{[Pd_2(1AB)_2(4AA)_2](BF_4)_2\}^{2+}$.

Synthesis of $[Pd_2(1AC)_2(2AA)_2](BF_4)_4$

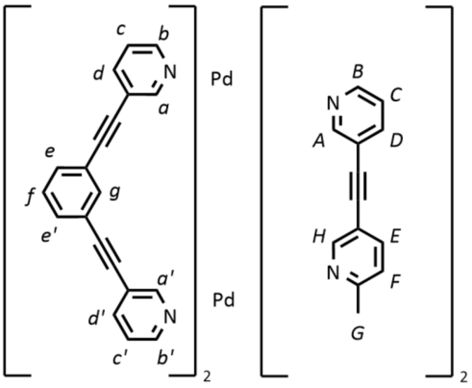

1AC (9.7 mg, 50 µmol, 1 eq.), **2AA** (14.0 mg, 50 µmol, 1 eq.) and a 11 mM stock solution of $[Pd(CH_3CN)_4](BF_4)_2$ in CH_3CN (5000 µL, 55 µmol, 1.1 eq.) were stirred at 70 °C under N_2 for 24 h. To the cooled reaction mixture was added Et_2O (20 mL) and stirred for 10 mins. The suspension was filtered through a sintered funnel and the precipitate washed with Et_2O (x3). The precipitate was re-dissolved in CH_3CN, and the solvent removed *in vacuo* to give the product as a beige solid (37.0 mg, 98%).

ESI-MS *m/z* = 416.39 $\{[Pd_2(\mathbf{1AC})_2(\mathbf{2AA})_2](BF_4)\}^{3+}$ calc. 416.39; 634.09 $\{[Pd_2(\mathbf{1AC})_2(\mathbf{2AA})_2](BF_4)F\}^{2+}$ calc. 634.09.

^{13}C NMR (101 MHz, CD_3CN) δ: 163.15, 162.99, 155.24 (*syn*-C_a, syn-$C_{a'}$), 154.87 (*anti*-C_A), 154.57 (*syn*-C_H, *syn*-C_A), 153.56, 152.09 (*syn*-C_B, *anti*-C_B), 151.15 (*syn*-C_b), 150.93 (*anti*-$C_{b'}$), 150.68 (*anti*-C_b), 150.32 (*syn*-$C_{b'}$), 143.16, 142.90 (*syn*-C_D/*syn*-C_d/*syn*-$C_{d'}$), 142.79 (*syn*-C_D/*syn*-C_d/*syn*-$C_{d'}$), 142.73 (*syn*-C_D/*syn*-C_d/*syn*-$C_{d'}$), 142.60 (*syn*-C_E), 142.31, 139.30 (*anti*-C_g), 139.22 (*syn*-C_g), 132.66, 132.48, 132.38 (*syn*-C_C), 130.79 (*syn*-C_F), 129.02 (*syn*-C_f/*syn*-C_c/*syn*-$C_{c'}$), 128.90 (*syn*-C_f/*syn*-C_c/*syn*-$C_{c'}$), 128.83 (*syn*-C_f/*syn*-C_c/*syn*-$C_{c'}$), 128.38 (*syn*-C_e, *syn*-$C_{e'}$), 125.24, 124.99, 124.73, 124.44, 124.22, 123.08, 123.02, 121.27, 95.47, 95.41, 90.39, 89.71, 86.07, 85.84, 85.62, 27.49 (*syn*-C_G), 26.80 (*anti*-C_G).

^{19}F NMR (376 MHz, CD_3CN) δ: -151.39.

^{1}H DOSY (400 MHz, CD_3CN) *D*: 7.4 × 10^{-10} m^2s^{-1}; R_S: 8.3 Å.

Major Isomer *syn*-[$Pd_2(1AC)_2(2AA)_2](BF_4)_4$

^{1}H NMR (400 MHz, CD_3CN) δ: 10.04 (s, 4H, H_H, H_A), 9.37-9.30 (m, 4H, H_a, $H_{a'}$), 9.15 (dd, J = 5.8, 1.9 Hz, 2H, H_b), 9.12 (dd, J = 5.8, 1.8 Hz, 2H, $H_{b'}$), 8.94 (dd, J = 5.9, 1.3 Hz, 2H, H_B), 8.24-8.19 (m, 2H, H_g), 8.08-7.99 (m, 6H, H_d, $H_{d'}$, H_D), 7.95 (dd, J = 8.2, 1.8 Hz, 2H, H_E), 7.73-7.64 (m, 4H, H_c, $H_{c'}$), 7.63-7.54 (m, 6H, H_e, $H_{e'}$, H_C), 7.54-7.43 (m, 4H, H_f, H_F), 3.45 (s, 6H, H_G).

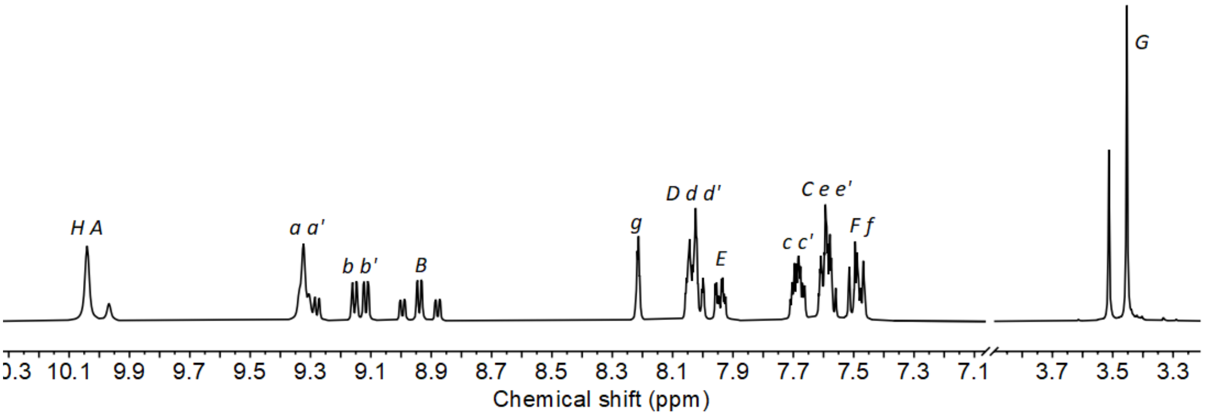

Figure S115 Partial ^{1}H NMR (400 MHz, CD_3CN) of [$Pd_2(1AC)_2(2AA)_2](BF_4)_4$ with peaks of major *syn*-isomer labelled.

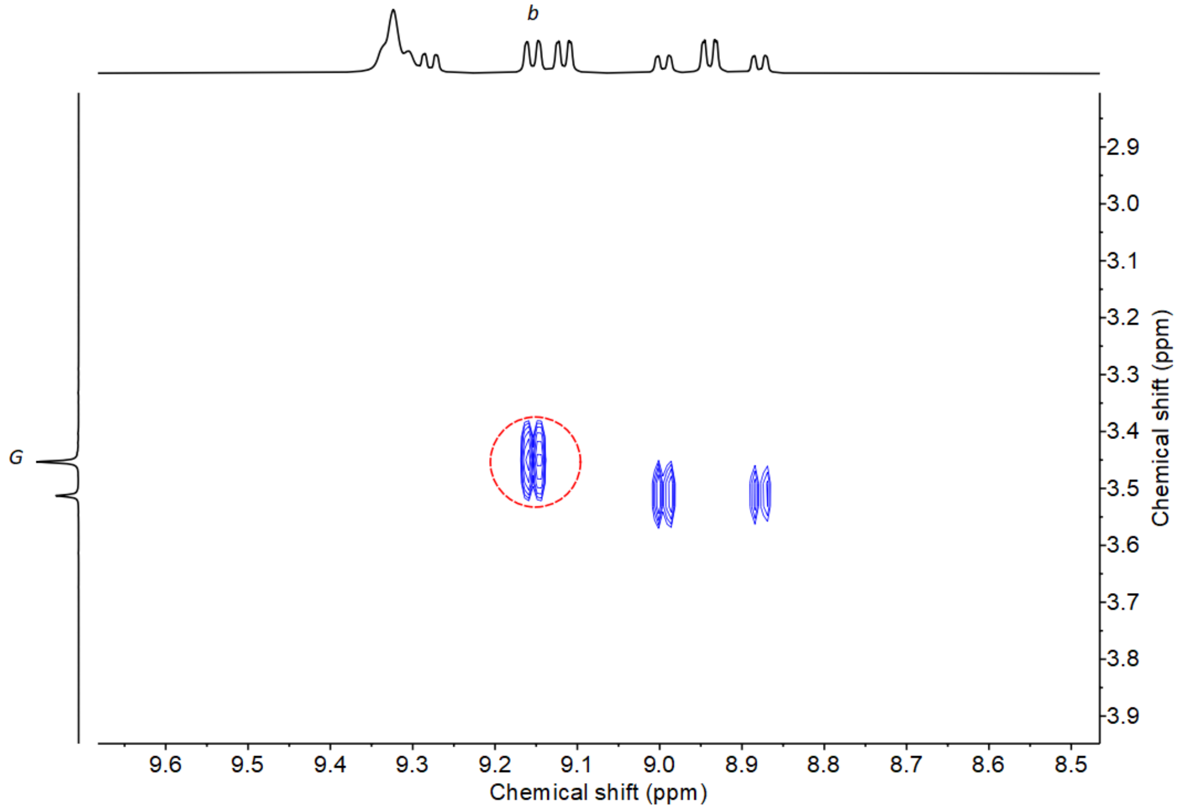

Figure S116 Partial NOESY Partial NOESY (400 MHz, CD_3CN) of [$Pd_2(1AC)_2(2AA)_2](BF_4)_4$ with peaks assigned to major *syn*-isomer.

Minor Isomer *anti*-[$Pd_2(1AC)_2(2AA)_2](BF_4)_4$

^{1}H NMR (400 MHz, CD_3CN) δ: 10.04 (s, 2H, H_H), 9.97 (s, 2H, H_A), 9.37-9.30 (m, 4H H_a, $H_{a'}$), 9.28 (dd, *J* = 6.1, 1.0 Hz, 2H, H_b), 9.00 (dd, *J* = 6.3, 1.1 Hz, 2H, $H_{b'}$), 8.88 (dd, *J* = 6.0, 1.3 Hz, 2H, H_B), 8.24-8.19 (m, 2H, H_g), 8.08-7.98 (m, 6H, H_d, $H_{d'}$, H_D), 7.94 (dd, *J* = 8.2, 1.8 Hz, 2H, H_E), 7.73-7.64 (m, 4H, H_c, $H_{c'}$), 7.64-7.54 (m, 6H, H_e, $H_{e'}$, H_C), 7.53-7.45 (m, 4H, H_f, H_F), 3.51 (s, 6H, H_G).

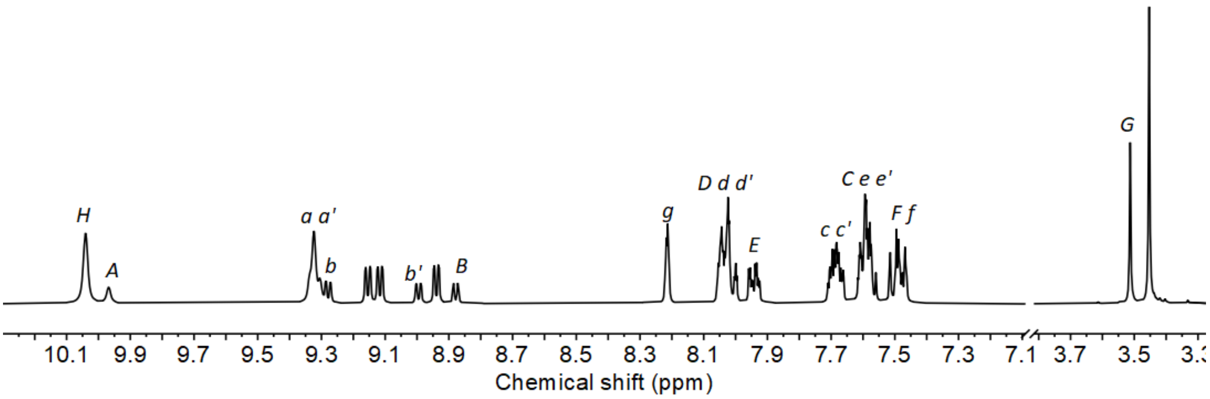

Figure S117 Partial ^{1}H NMR (400 MHz, CD_3CN) of [$Pd_2(1AC)_2(2AA)_2](BF_4)_4$ with peaks of minor *anti*-isomer labelled.

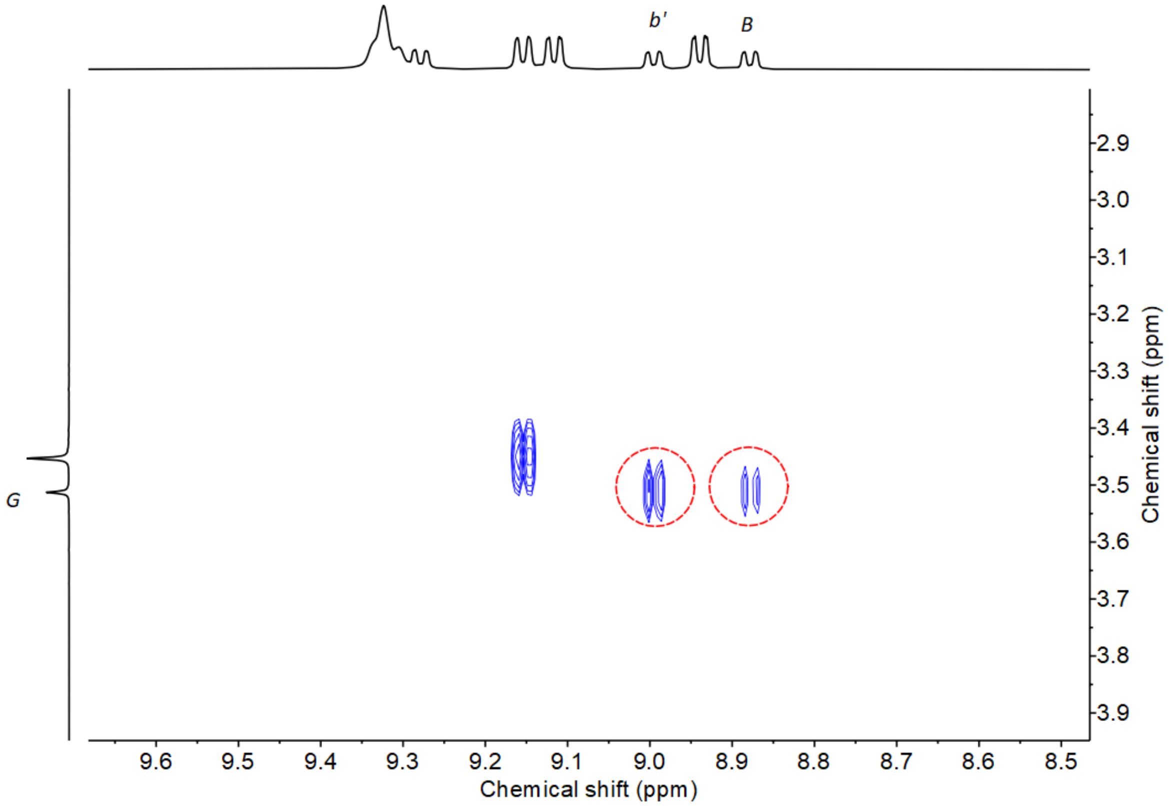

Figure S118 Partial NOESY (400 MHz, CD_3CN) of [$Pd_2(1AC)_2(2AA)_2](BF_4)_4$ with peaks assigned to minor *anti*-isomer.

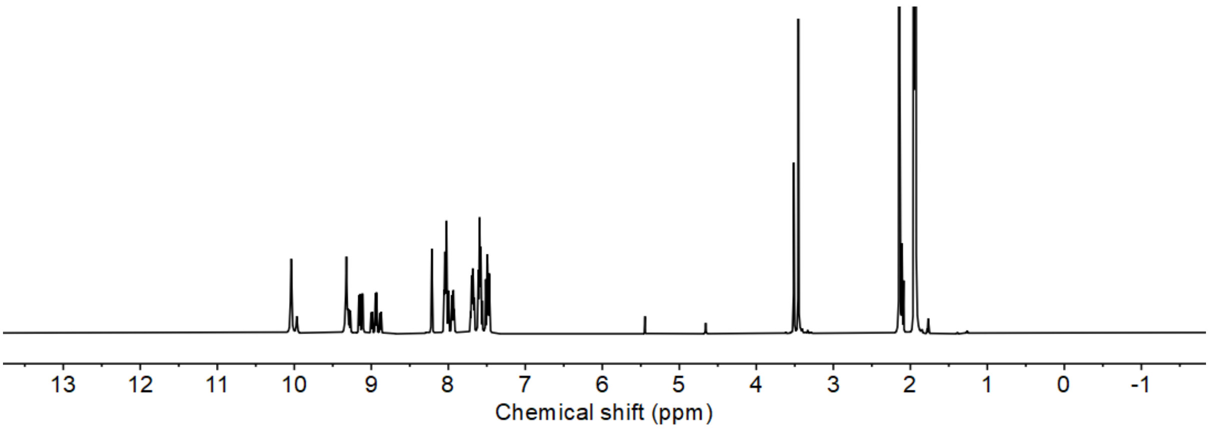

Figure S119 ^{1}H NMR (400 MHz, CD_3CN) of $[Pd_2(1AC)_2(2AA)_2](BF_4)_4$.

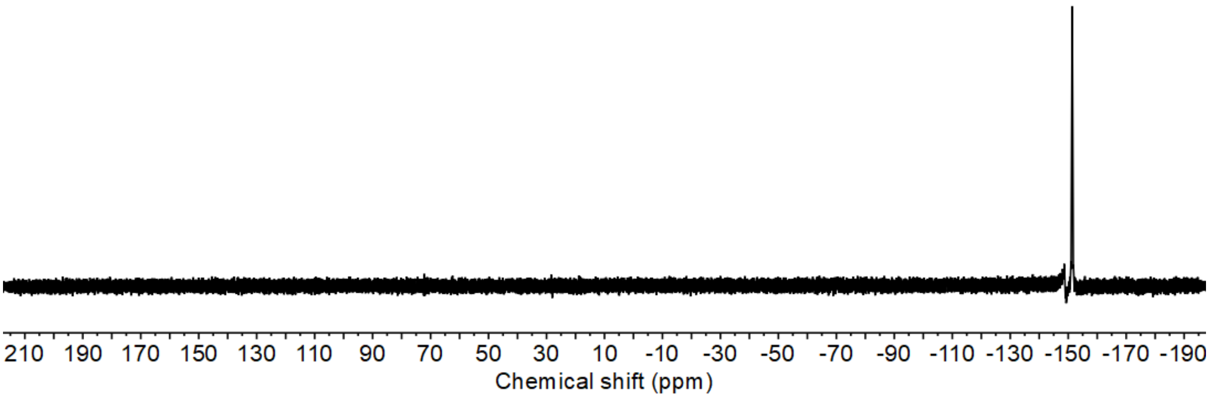

Figure S120 ^{19}F NMR (376 MHz, CD_3CN) of $[Pd_2(1AC)_2(2AA)_2](BF_4)_4$.

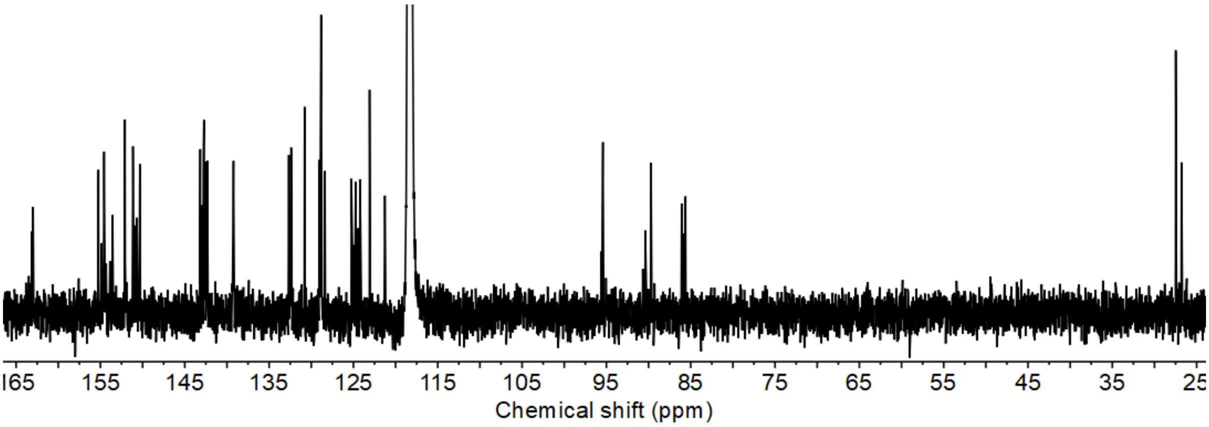

Figure S121 Partial ^{13}C NMR (101 MHz, CD_3CN) of $[Pd_2(1AC)_2(2AA)_2](BF_4)_4$.

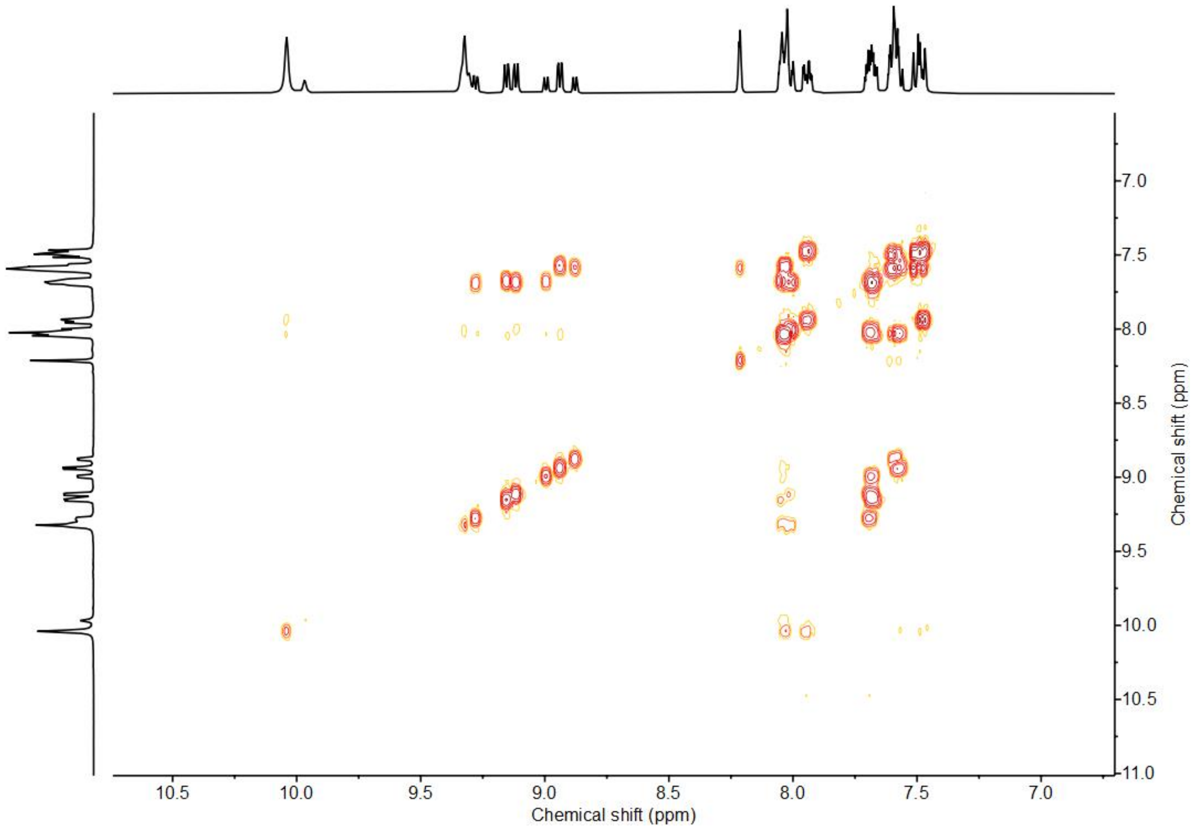

Figure S122 Partial COSY (CD_3CN) of $[Pd_2(1AC)_2(2AA)_2](BF_4)_4$.

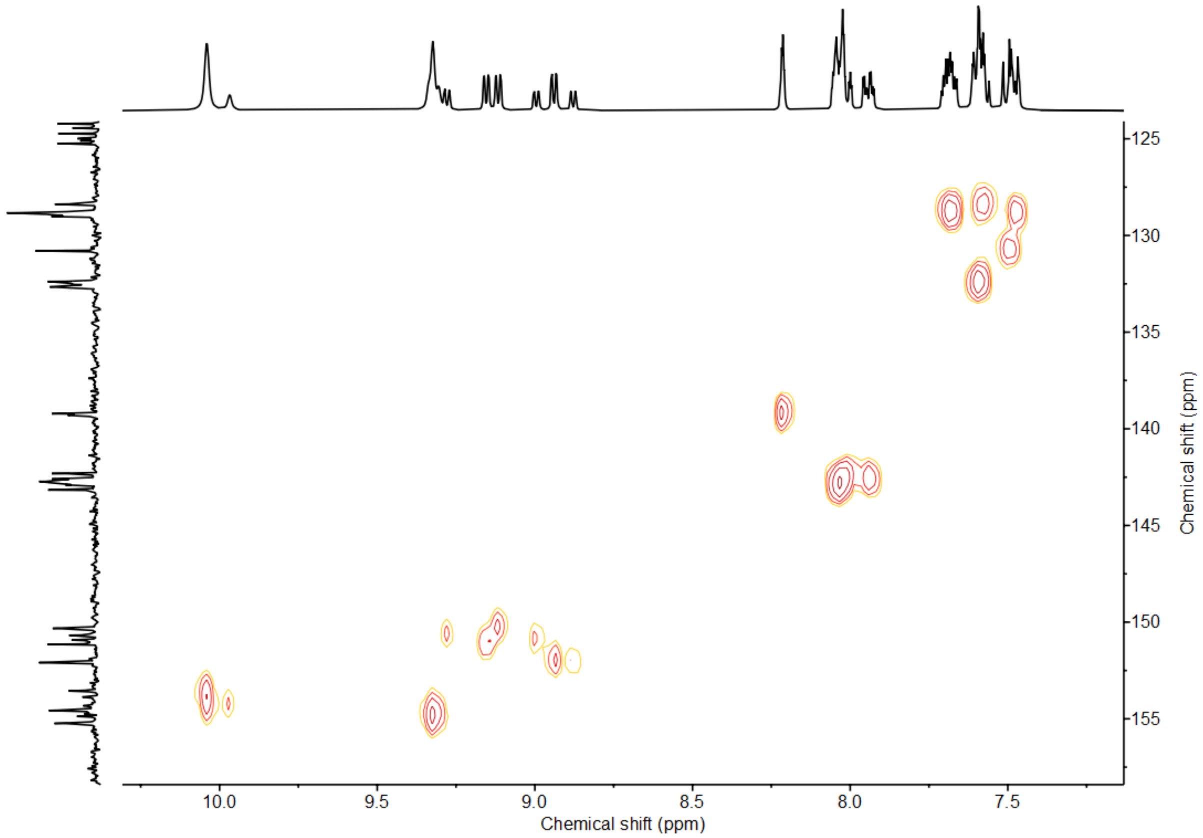

Figure S123 Partial HSQC (CD_3CN) of $[Pd_2(1AC)_2(2AA)_2](BF_4)_4$.

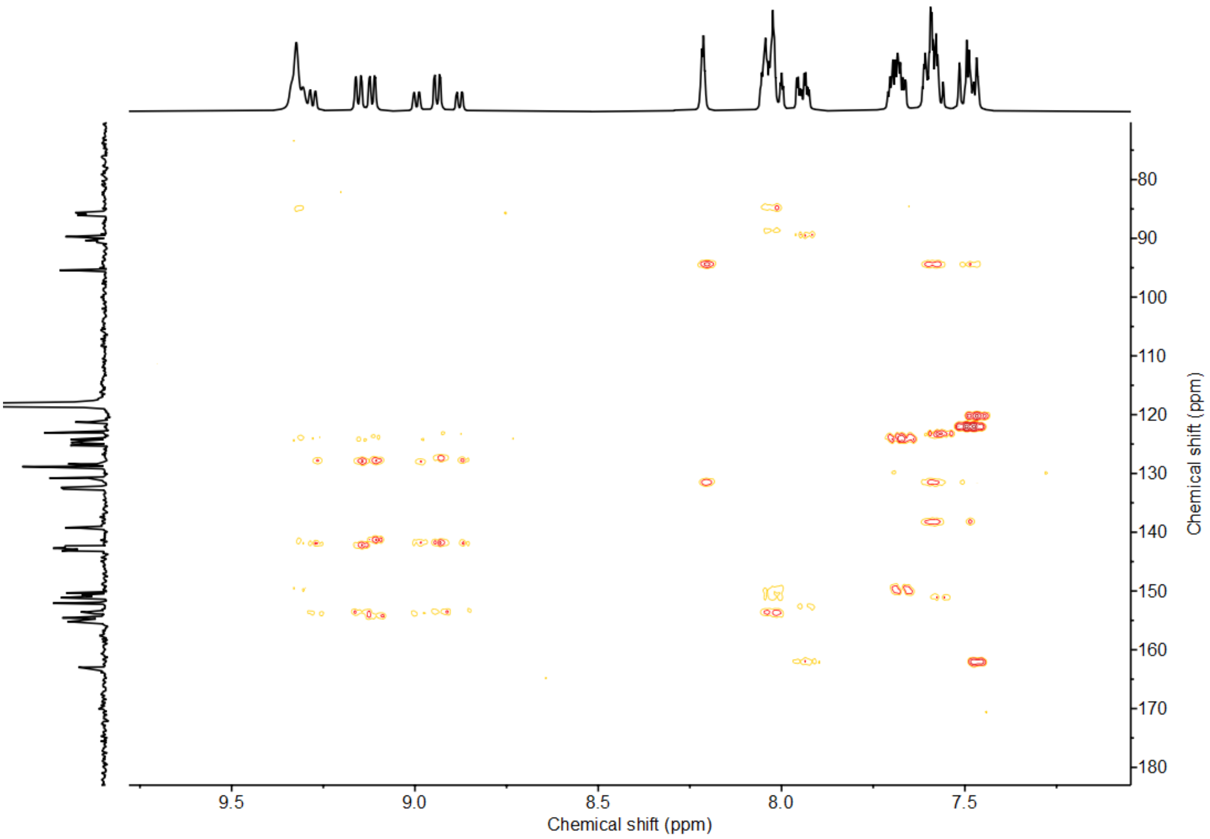

Figure S124 Partial HMBC (CD_3CN) of $[Pd_2(1AC)_2(2AA)_2](BF_4)_4$.

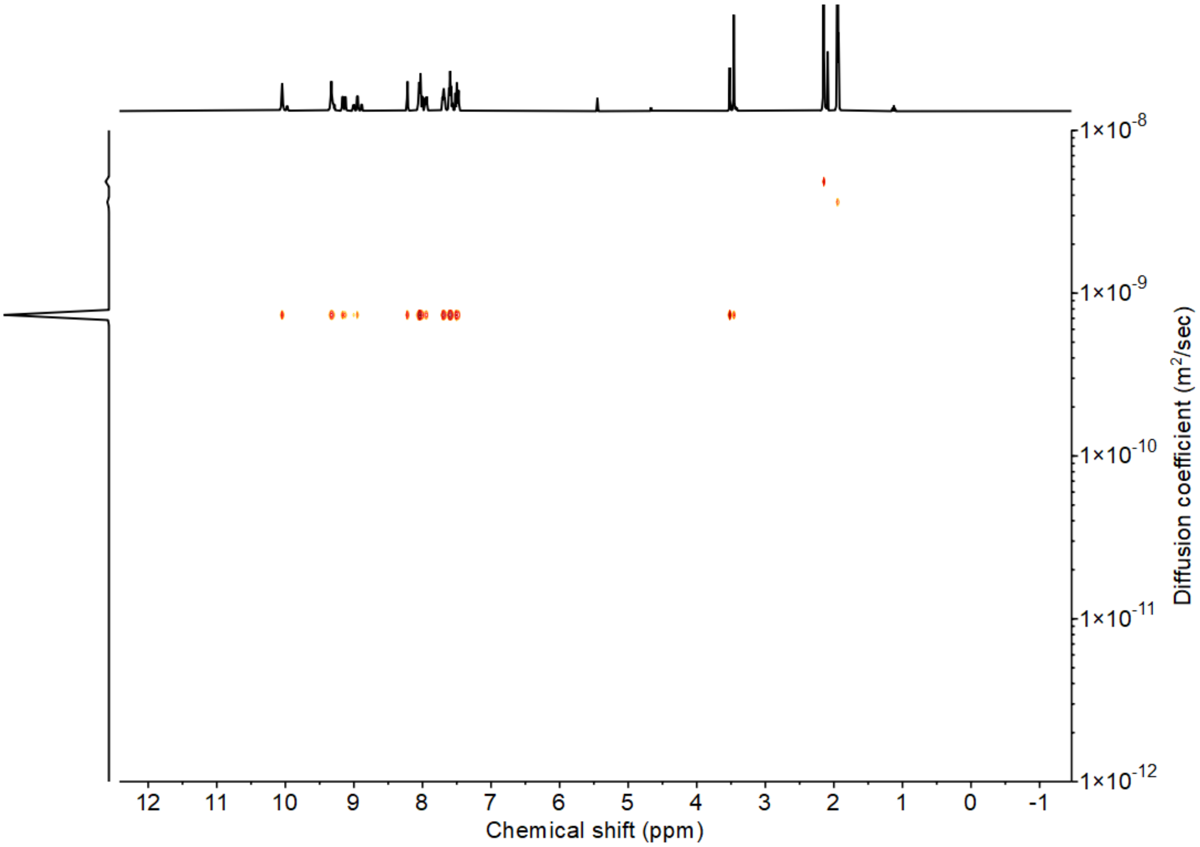

Figure S125 DOSY (CD_3CN) of $[Pd_2(1AC)_2(2AA)_2](BF_4)_4$.

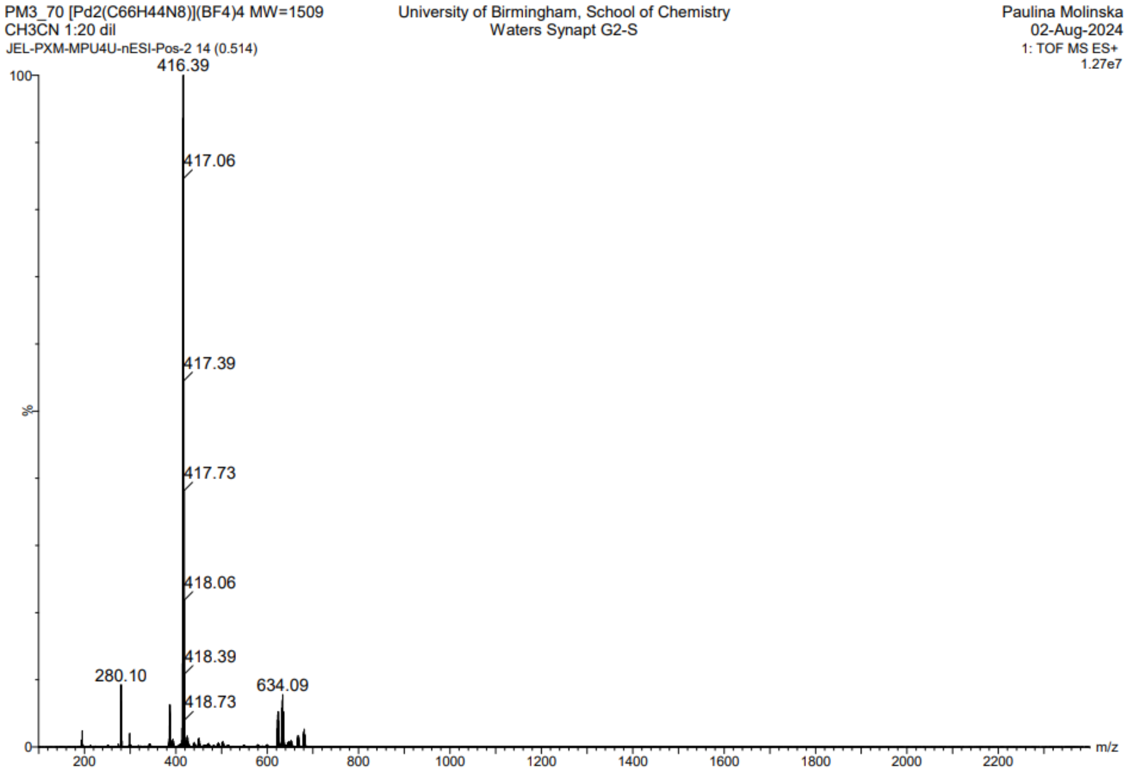

Figure S126 ESI-MS of $[Pd_2(1AC)_2(2AA)_2](BF_4)_4$.

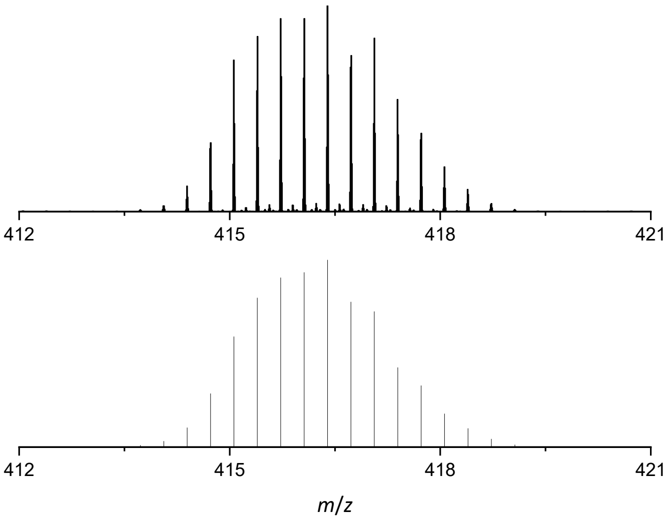

Figure S127 Observed (top) and calculated (bottom) isotopic patterns for $\{[Pd_2(1AC)_2(2AA)_2](BF_4)\}^{3+}$.

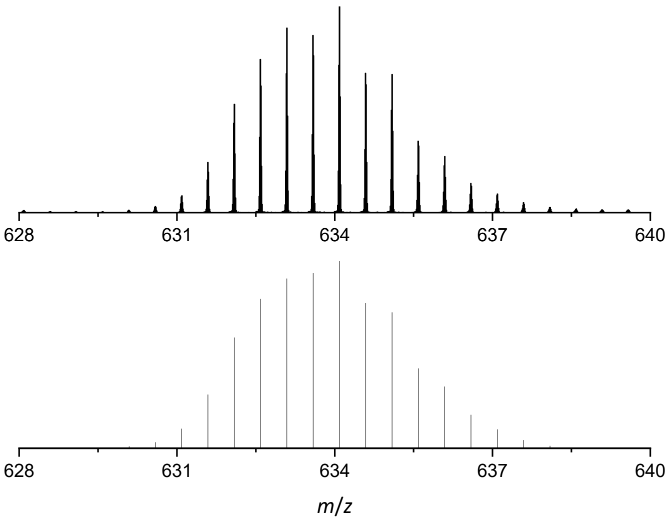

Figure S128 Observed (top) and calculated (bottom) isotopic patterns for {[$Pd_2(1AC)_2(2AA)_2$](BF_4)F}$^{2+}$.

Synthesis of $[Pd_2(1AC)_2(3AA)_2](BF_4)_4$

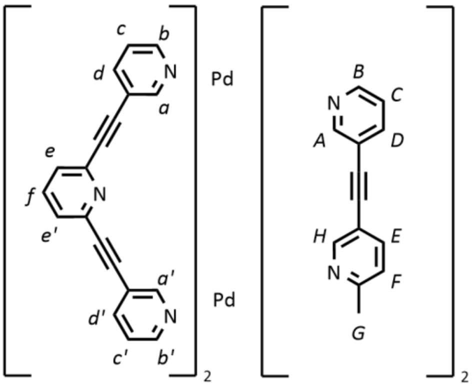

1AC (4.9 mg, 25 µmol, 1 eq.), **3AA** (7.0 mg, 25 µmol, 1 eq.) and a 5.5 mM stock solution of $[Pd(CH_3CN)_4](BF_4)_2$ in CH_3CN (5000 µL, 27.5 µmol, 1.1 eq.) were stirred at 70 °C under N_2 for 24 h. To the cooled reaction mixture was added EtOAc (20 mL) and stirred for 20 mins. The suspension was filtered through a sintered funnel and the precipitate washed with Et_2O (x3). The precipitate was re-dissolved in CH_3CN, and the solvent removed *in vacuo* to give the product as a beige solid (13.3 mg, 70%).

ESI-MS *m/z* = 291.04. $\{[Pd_2(\mathbf{1AC})_2(\mathbf{3AA})_2]\}^{4+}$ calc. 291.04; 416.72 $\{[Pd_2(\mathbf{1AC})_2(\mathbf{3AA})_2](BF_4)\}^{3+}$ calc. 417.06; 669.09 $\{[Pd_2(\mathbf{1AC})_2(\mathbf{3AA})_2](BF_4)_2\}^{2+}$ calc. 669.09.

^{13}C NMR (101 MHz, CD_3CN) δ: 162.93, 162.73, 155.69 (*syn*-C_a, *syn*-$C_{a'}$), 155.36 (*anti*-C_A), 155.22 (*syn*-C_A /*syn*-C_H), 155.07 (*syn*-C_A /*syn*-C_H), 155.00, 154.42, 154.25, 151.86 (*syn*-C_b /*syn*-$C_{b'}$, *syn*-C_B, *anti*-C_B), 151.64 (*anti*-$C_{b'}$), 151.32 (*anti*-C_b), 151.02 (*syn*-C_b /*syn*-$C_{b'}$), 143.88, 143.59 (*syn*-C_d /*syn*-$C_{d'}$), 143.45 (*syn*-C_d /*syn*-$C_{d'}$), 143.14, 143.09, 143.02, 142.67--, 142.56 (*syn*-C_D), 142.41 (*syn*-C_E), 138.96 (*syn*-C_f), 128.92, 128.81 (*syn*-C_F /*syn*-C_c /*syn*-$C_{c'}$), 128.68, 128.35, 127.72 (*syn*-C_C /*syn*-C_e /*syn*-$C_{e'}$), 127.57 (*syn*-C_C /*syn*-C_e /*syn*-$C_{e'}$), 127.41 (*syn*-C_C /*syn*-C_e /*syn*-$C_{e'}$), 124.69, 124.60, 124.44, 124.23, 121.29, 121.23, 95.03, 94.92, 94.84, 90.90, 90.69, 89.95, 89.87, 83.72, 83.50, 83.34, 27.44 (*syn*-C_G), 26.75 (*anti*-C_G).

^{19}F NMR (376 MHz, CD_3CN) δ: -151.43, -144.62.

^{1}H DOSY (400 MHz, CD_3CN) *D*: 6.4×10^{-10} m^2s^{-1}; R_S: 9.6 Å.

Major Isomer *syn*-[$Pd_2(1AC)_2(3AA)_2](BF_4)_4$

^{1}H NMR (400 MHz, CD_3CN) δ: 10.13 (d, *J* = 5.9 Hz, 4H, H_A, H_H), 9.40 (m, 4H, H_a, $H_{a'}$), 9.20 (m, 4H, H_b, $H_{b'}$), 8.95 (d, *J* = 5.8 Hz, 2H, H_B), 8.17-8.04 (m, 4H, H_d, $H_{d'}$), 8.02 (d, *J* = 8.0 Hz, 2H, H_D), 7.93 (m, 2H, H_E), 7.86 (t, *J* = 7.9, 2H, H_f), 7.70 (m, 4H, H_c, $H_{c'}$), 7.63-7.53 (m, 6H, H_C, H_e, $H_{e'}$), 7.47 (d, *J* = 8.2 Hz, 2H, H_F), 3.46 (s, 6H, H_G).

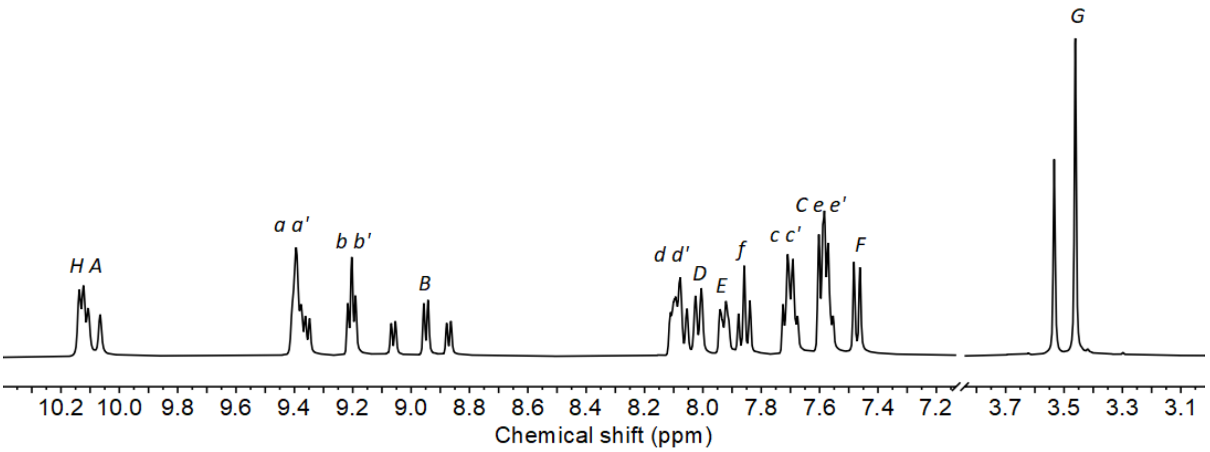

Figure S129 Partial ^{1}H NMR (400 MHz, CD_3CN) of [$Pd_2(1AC)_2(3AA)_2](BF_4)_4$ with peaks of major *syn*-isomer labelled.

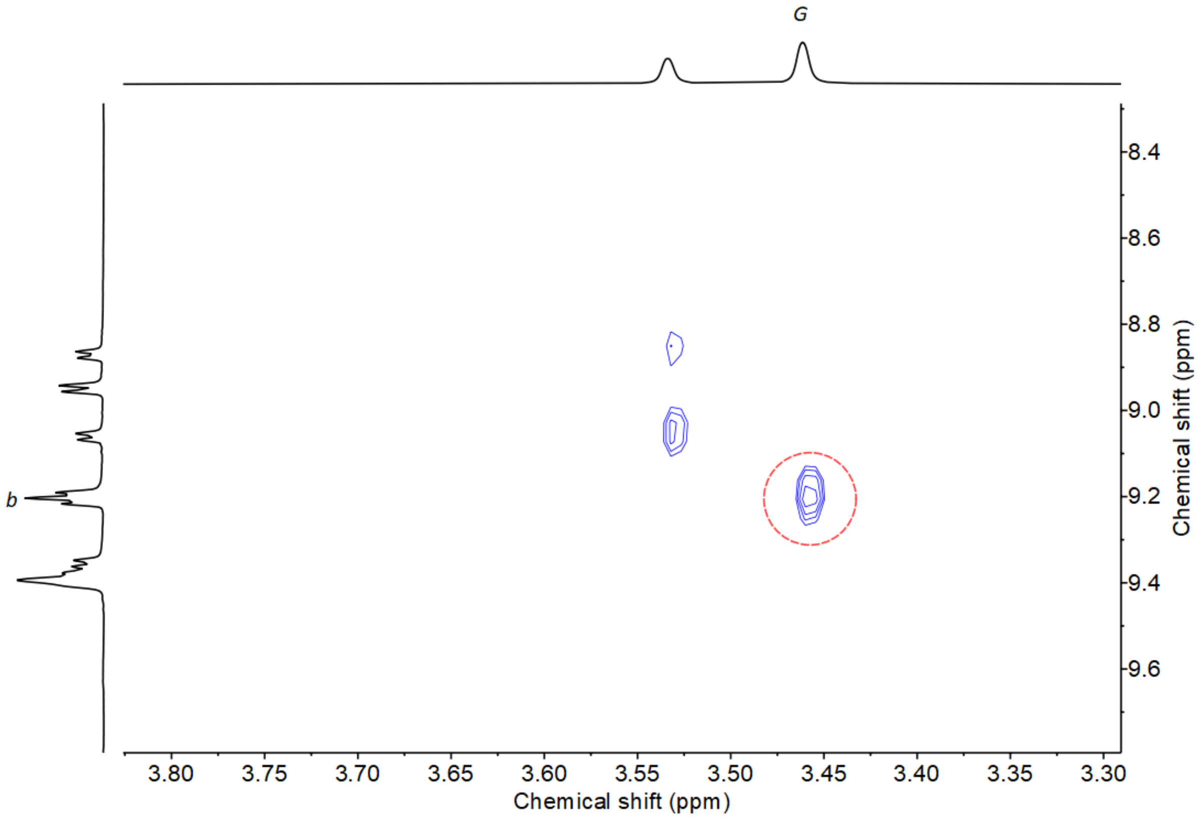

Figure S130 Partial NOESY Partial NOESY (400 MHz, CD_3CN) of [$Pd_2(1AC)_2(3AA)_2](BF_4)_4$ with peaks assigned to major *syn*-isomer.

Minor Isomer *anti*-[$Pd_2(1AC)_2(3AA)_2](BF_4)_4$

^{1}H NMR (400 MHz, CD_3CN) δ: 10.11 (s, 2H, H_H), 10.07 (s, 2H, H_A), 9.39 (m, 4H, H_a, $H_{a'}$), 9.36 (d, *J* = 5.9 Hz, 2H, H_b), 9.06 (d, *J* = 5.8 Hz, 2H, $H_{b'}$), 8.87 (d, *J* = 5.8 Hz, 2H, H_B), 8.17-8.04 (m, 4H, H_d, $H_{d'}$), 8.02 (d, *J* = 8.0 Hz, 2H, H_D), 7.93 (m, 2H, H_E), 7.86 (t, *J* = 7.9, 2H, H_f), 7.70 (m, 4H, H_c, $H_{c'}$), 7.63-7.53 (m, 6H, H_C, H_e, $H_{e'}$), 7.47 (d, *J* = 8.2 Hz, 2H, H_F), 3.53 (s, 3H, H_G).

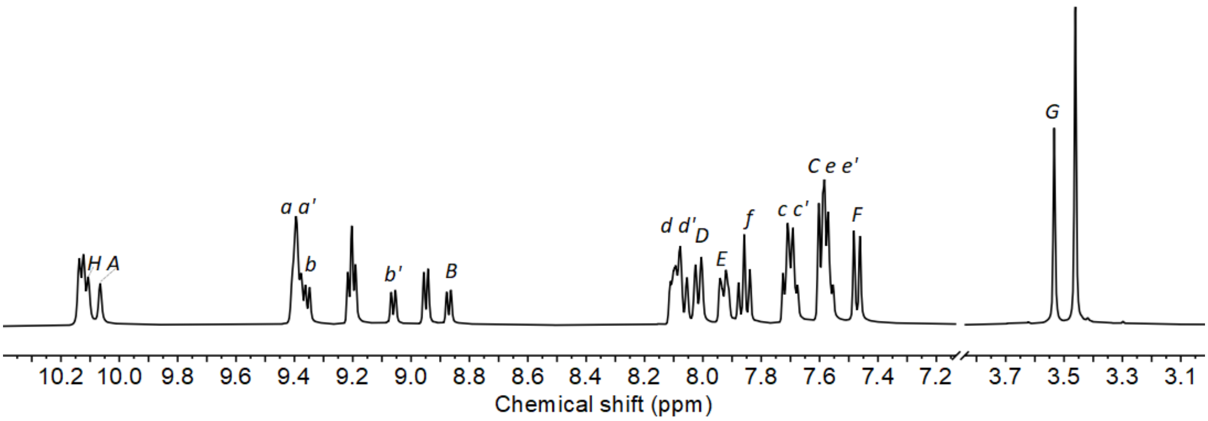

Figure S131 Partial ^{1}H NMR (400 MHz, CD_3CN) of [$Pd_2(1AC)_2(3AA)_2](BF_4)_4$ with peaks of minor *anti*-isomer labelled.

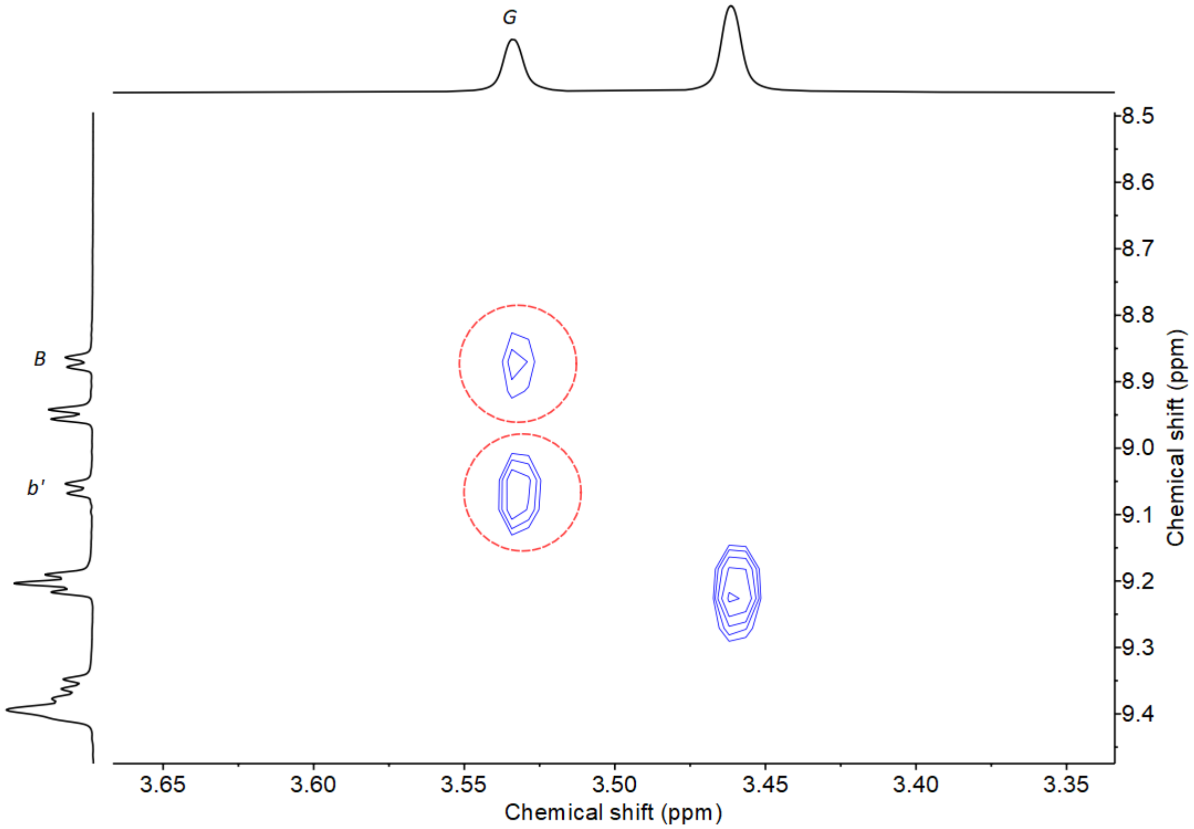

Figure S132 Partial NOESY Partial NOESY (400 MHz, CD_3CN) of [$Pd_2(1AC)_2(3AA)_2](BF_4)_4$ with peaks assigned to minor *anti*-isomer.

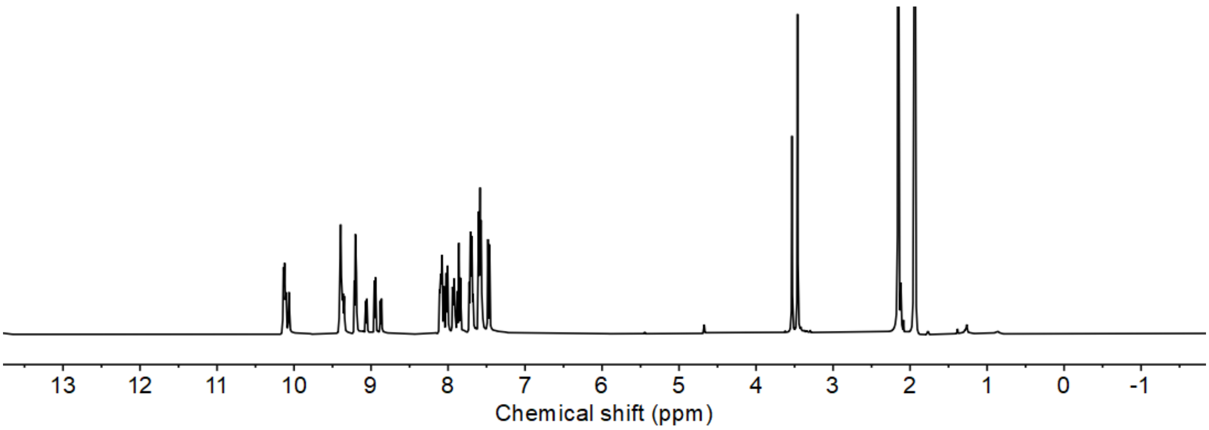

Figure S133 ^{1}H NMR (400 MHz, CD_3CN) of $[Pd_2(1AC)_2(3AA)_2](BF_4)_4$.

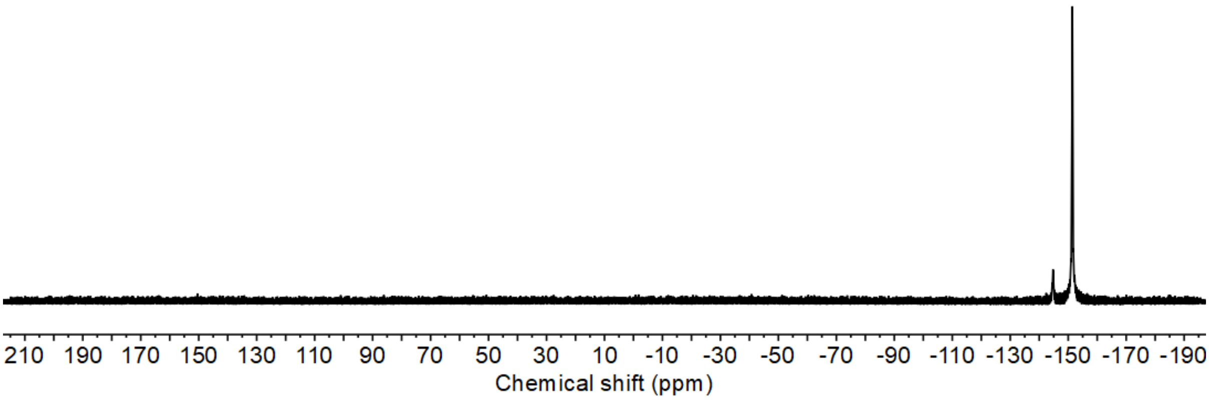

Figure S134 ^{19}F NMR (376 MHz, CD_3CN) of $[Pd_2(1AC)_2(3AA)_2](BF_4)_4$.

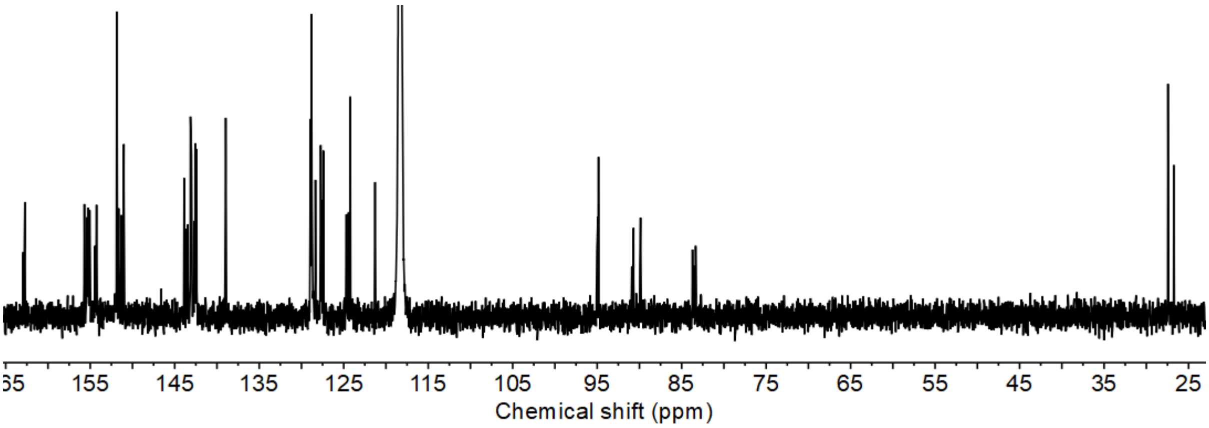

Figure S135 Partial ^{13}C NMR (101 MHz, CD_3CN) of $[Pd_2(1AC)_2(3AA)_2](BF_4)_4$.

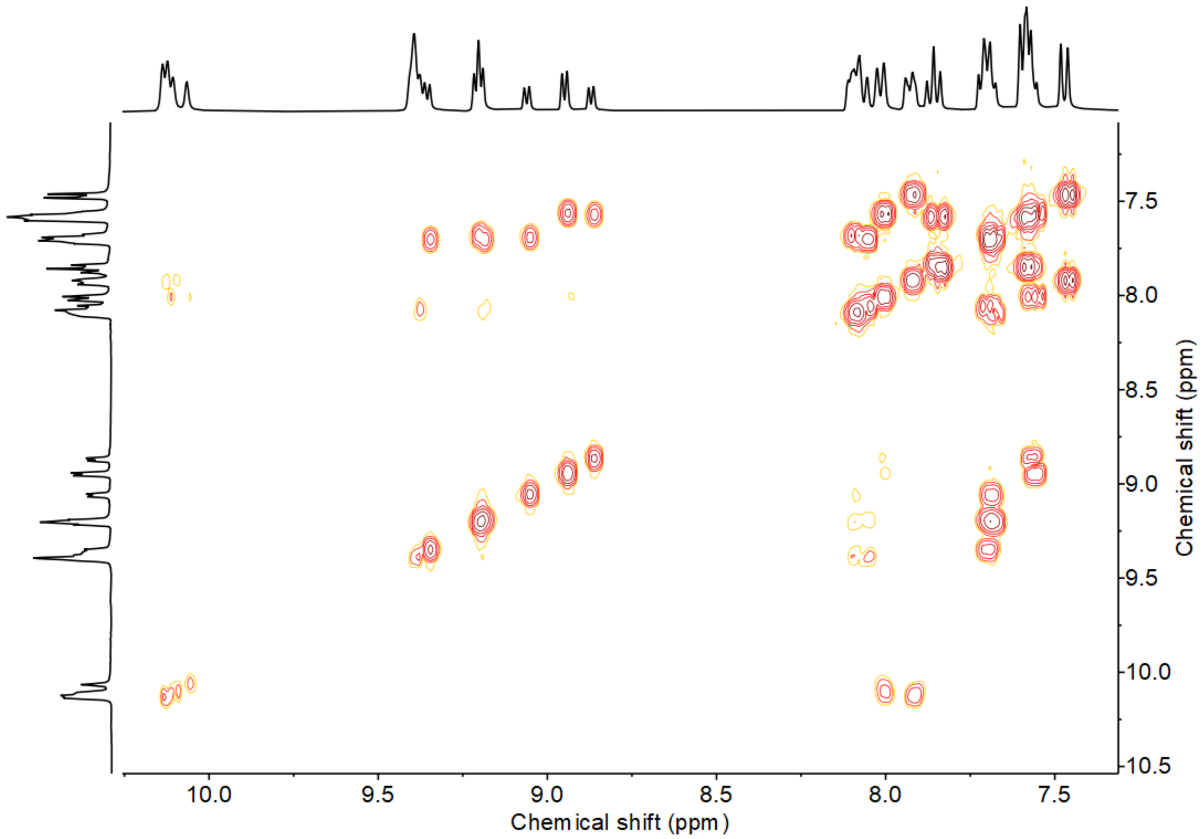

Figure S136 Partial COSY (CD_3CN) of $[Pd_2(1AC)_2(3AA)_2](BF_4)_4$.

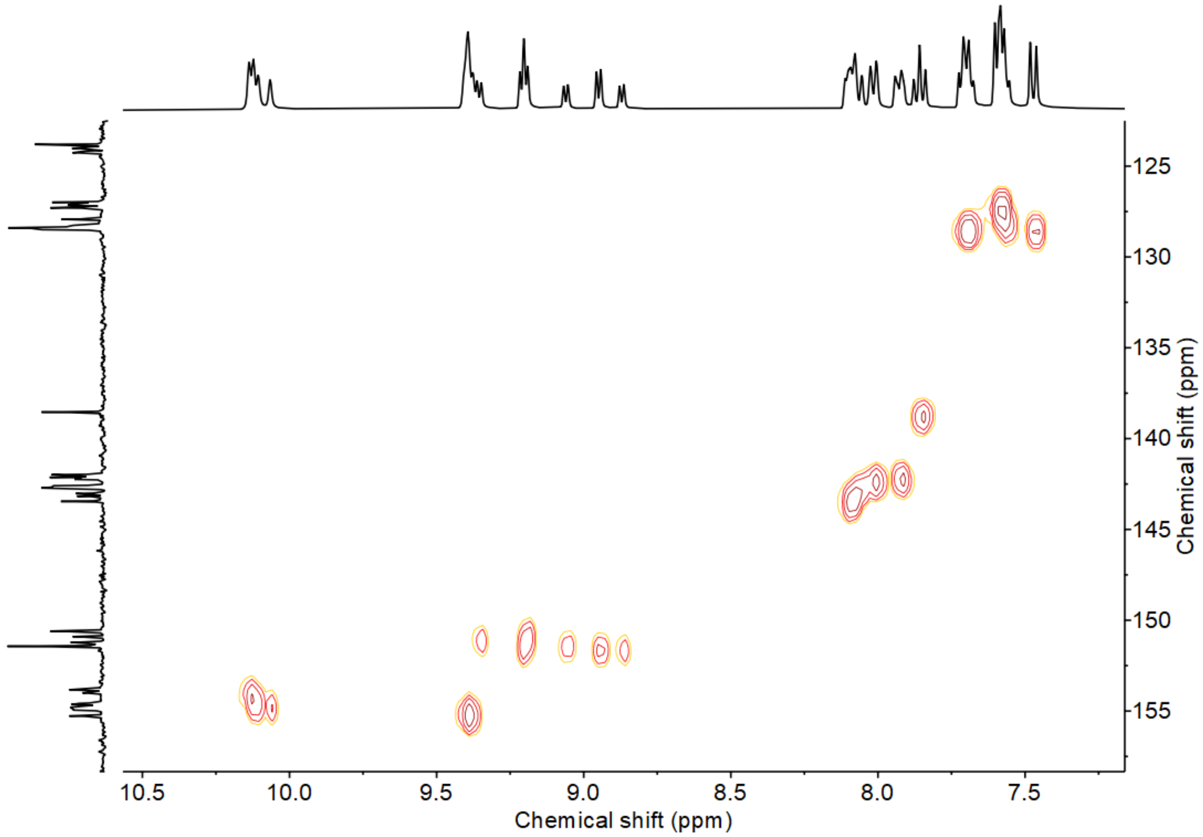

Figure S137 Partial HSQC (CD_3CN) of $[Pd_2(1AC)_2(3AA)_2](BF_4)_4$.

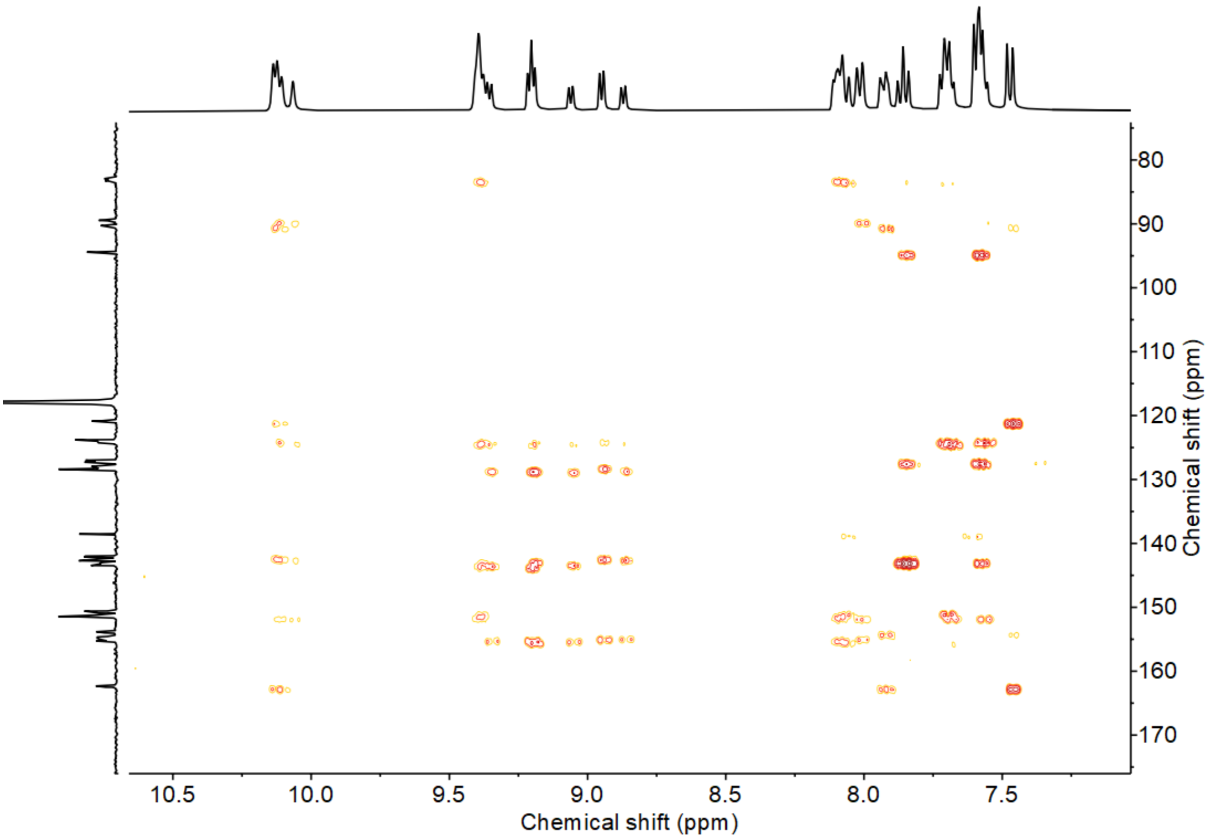

Figure S138 Partial HMBC (CD_3CN) of $[Pd_2(1AC)_2(3AA)_2](BF_4)_4$.

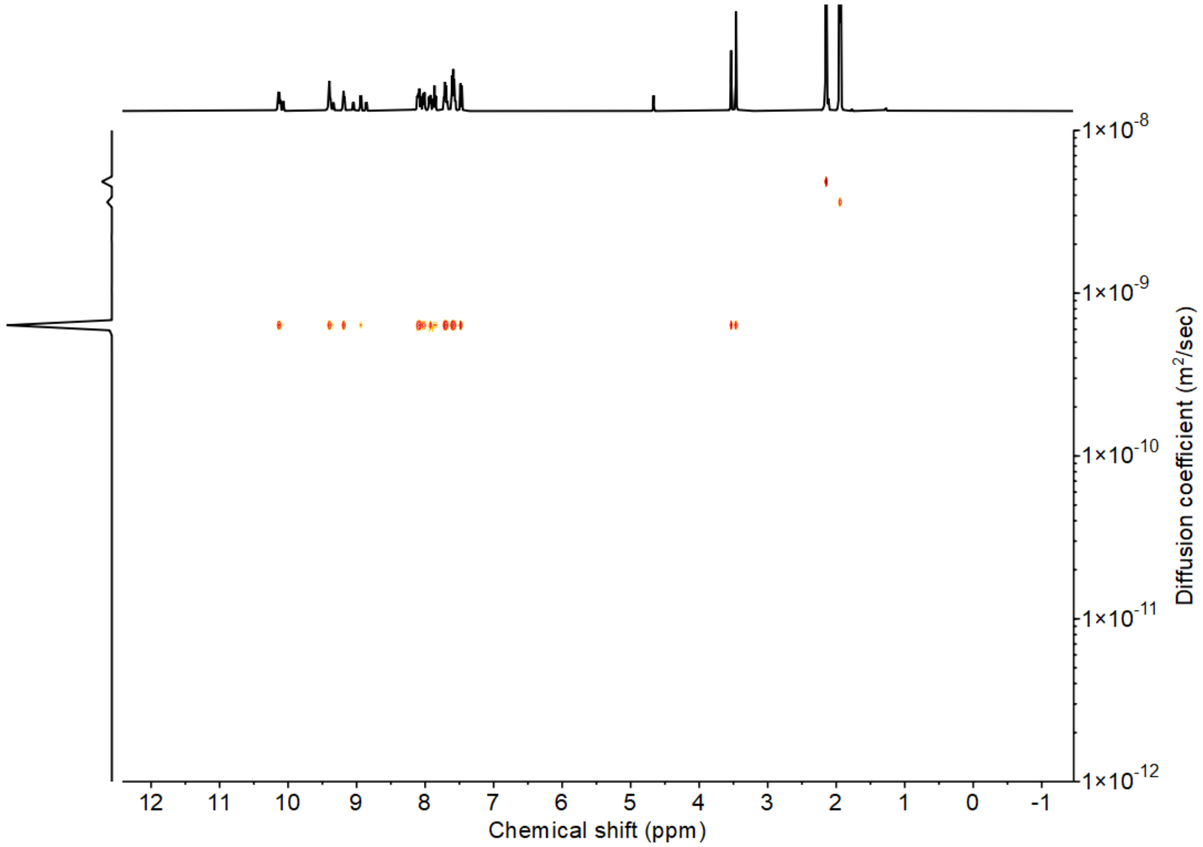

Figure S139 DOSY (CD_3CN) of $[Pd_2(1AC)_2(3AA)_2](BF_4)_4$.

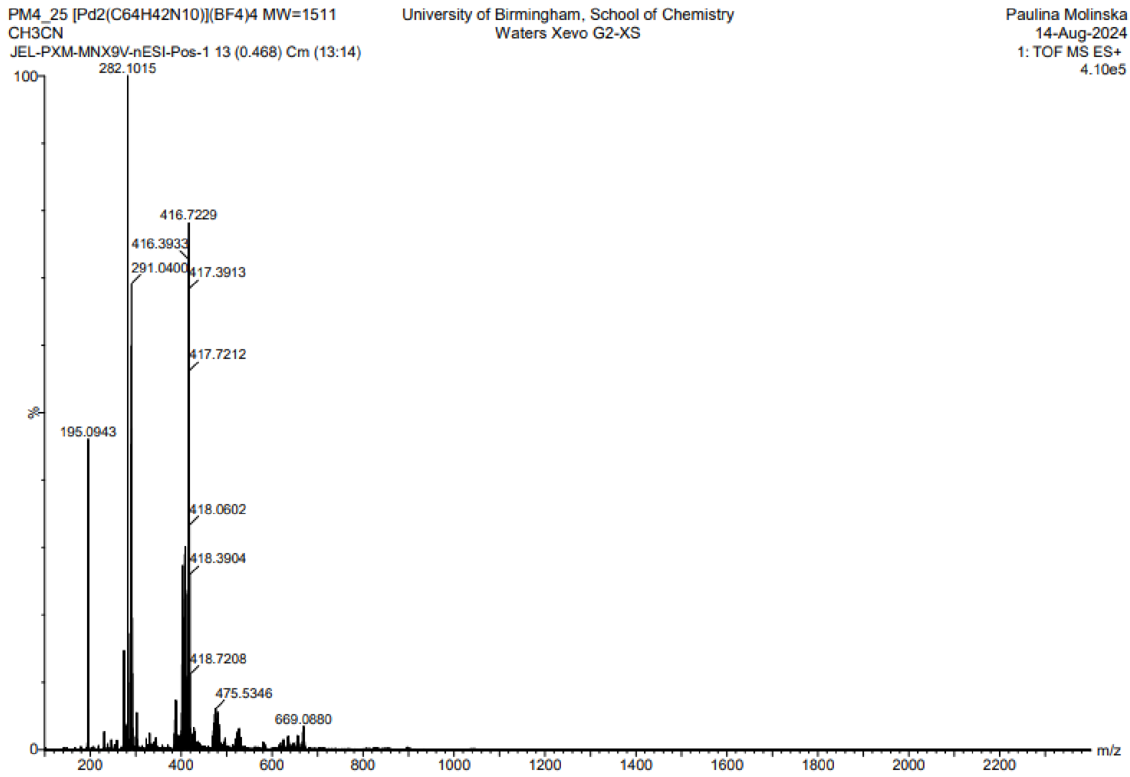

Figure S140 ESI-MS of $[Pd_2(1AC)_2(3AA)_2](BF_4)_4$.

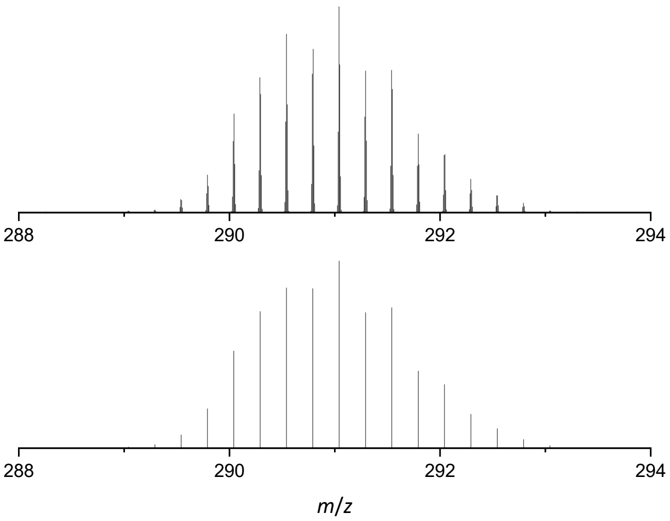

Figure S141 Observed (top) and calculated (bottom) isotopic patterns for $\{[Pd_2(1AC)_2(3AA)_2]\}^{4+}$.

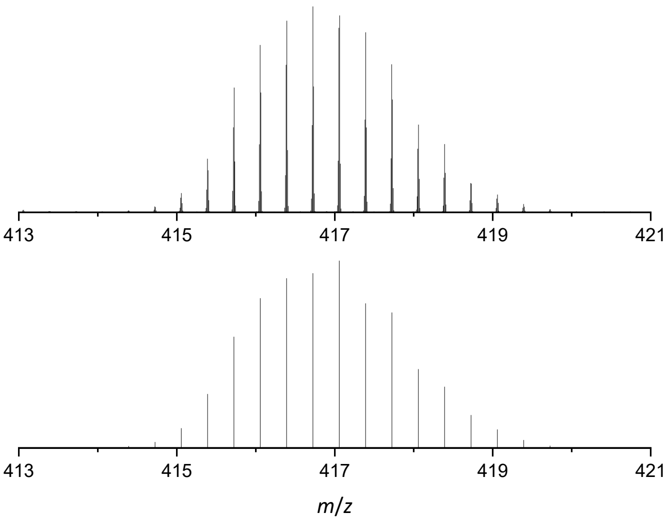

Figure S142 Observed (top) and calculated (bottom) isotopic patterns for {[$Pd_2(1AC)_2(3AA)_2](BF_4)\}^{3+}$.

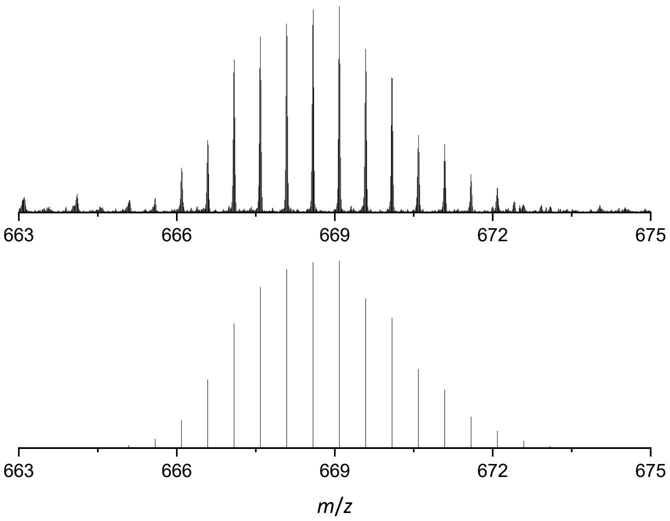

Figure S143 Observed (top) and calculated (bottom) isotopic patterns for {[$Pd_2(1AC)_2(3AA)_2](BF_4)_2\}^{2+}$.

Synthesis of $[Pd_2(\mathbf{1AC})_2(\mathbf{4AA})_2](BF_4)_4$

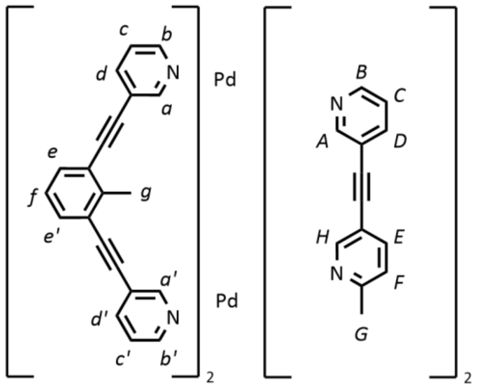

1AC (4.9 mg, 25 µmol, 1 eq.), **4AA** (7.4 mg, 25 µmol, 1 eq.) and a 5.5 mM stock solution of $[Pd(CH_3CN)_4](BF_4)_2$ in CH_3CN (5000 µL, 27.5 µmol, 1.1 eq.) were stirred at 70 °C under N_2 for 24 h. To the cooled reaction mixture was added Et_2O (20 mL) and stirred for 10 mins. The suspension was filtered through a sintered funnel and the precipitate washed with Et_2O (x3). The precipitate was re-dissolved in CH_3CN, and the solvent removed *in vacuo* to give the product as a beige solid (10.8 mg, 56%).

ESI-MS *m/z* = 425.74. $\{[Pd_2(\mathbf{1AC})_2(\mathbf{4AA})_2](BF_4)\}^{3+}$ calc. 425.74; 648.11 $\{[Pd_2(\mathbf{1AC})_2(\mathbf{4AA})_2](BF_4)F\}^{2+}$ calc. 648.11; 682.11 $\{[Pd_2(\mathbf{1AC})_2(\mathbf{4AA})_2](BF_4)_2\}^{2+}$ calc. 682.11.

^{13}C NMR (101 MHz, CD_3CN) δ: 162.95, 162.71, 155.09 (*syn*-$C_{a'}$, *anti*-$C_{a'}$), 154.80 (*anti*-C_a), 154.73 (*syn*-C_A, *anti*-C_A), 154.62, 154.48 (*syn*-C_a), 154.21 (*syn*-C_H, *anti*-C_H), 154.06, 151.95 (*syn*-C_B, *anti*-C_B), 151.08 (*syn*-C_b), 150.82 (*anti*-C_b /*anti*-$C_{b'}$), 150.76 (*anti*-C_b /*anti*-$C_{b'}$), 150.67, 150.26 (*syn*-$C_{b'}$), 147.42, 143.58, 143.00 (*syn*-C_D), 142.79 (*syn*-C_d/ *syn*-$C_{d'}$, *anti*-C_D, *anti*-C_d/ *anti*-$C_{d'}$), 142.65 (*anti*-C_d/ *anti*-$C_{d'}$), 142.51 (*anti*-C_E), 142.44 (*syn*-C_E), 142.08 (*syn*-C_d/ *syn*-$C_{d'}$), 134.75, 133.04, 132.83 (*syn*-C_C /*anti*-C_C), 132.76 (*syn*-C_C /*anti*-C_C), 132.58, 128.99 (*syn*-C_c /*syn*-$C_{c'}$), 128.89 (*syn*-C_c /*syn*-$C_{c'}$), 128.86 (*syn*-C_F, *anti*-C_F), 128.65 (*anti*-$C_{c'}$), 128.40 (*anti*-C_c), 128.25 (*syn*-C_e, *anti*-$C_{e'}$), 127.34 (*syn*-C_f, *anti*-C_f), 125.49, 125.21, 125.00, 124.59, 124.46, 123.13, 122.90, 122.85, 121.45, 121.39, 95.29, 94.96, 94.69, 90.45, 90.37, 90.00, 89.70, 89.53, 88.63, 27.40 (*syn*-C_G), 26.78 (*anti*-C_G), 21.31 (*anti*-C_g), 21.29 (*syn*-C_g), 19.95.

^{19}F NMR (376 MHz, CD_3CN) δ: -151.31.

^{1}H DOSY (400 MHz, CD_3CN) *D*: 7.4×10^{-10} m^2s^{-1}; R_S: 8.3 Å.

Major Isomer *syn*-[$Pd_2(1AC)_2(4AA)_2](BF_4)_4$

^{1}H NMR (400 MHz, CD_3CN) δ: 10.18 (s, 2H, H_H), 10.07 (s, 2H, H_A), 9.30 (s, 2H, H_a), 9.23 (m, 2H, $H_{a'}$), 9.19 (d, J = 5.9 Hz, 2H, H_b), 9.16 (d, J = 5.9 Hz, 2H, $H_{b'}$), 8.91 (d, J = 5.8 Hz, 2H, H_B), 8.11-7.96 (m, 6H, H_d, $H_{d'}$, H_D), 7.95-7.87 (m, 2H, H_E), 7.71 (m, 4H, H_c, $H_{c'}$), 7.60-7.48 (m, 6H, H_e, $H_{e'}$, H_C), 7.44 (d, J = 8.1 Hz, 2H, H_F), 7.27 (t, J = 7.7 Hz, 2H, H_f), 3.41 (s, 6H, H_G), 3.16 (s, 6H, H_g).

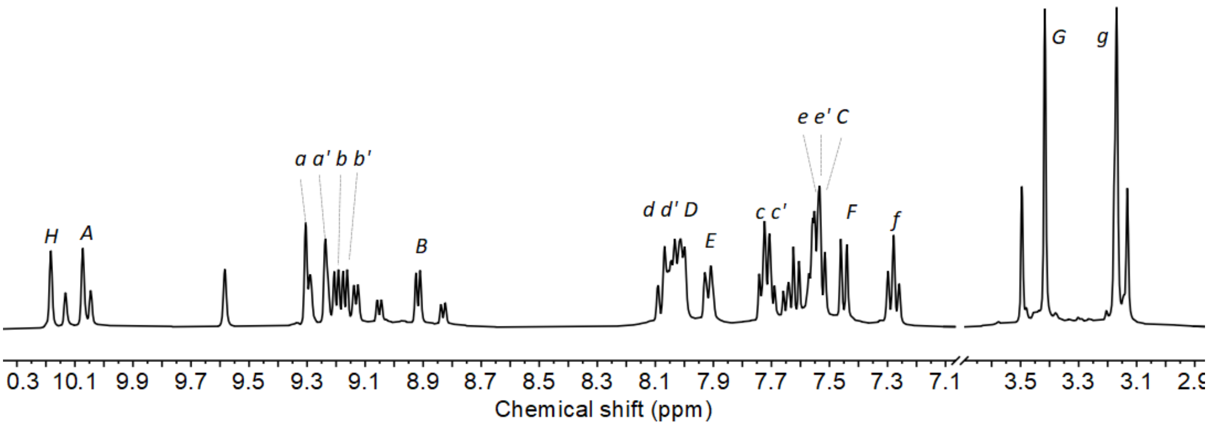

Figure S144 Partial ^{1}H NMR (400 MHz, CD_3CN) of [$Pd_2(1AC)_2(4AA)_2](BF_4)_4$ with peaks of major *syn*-isomer labelled.

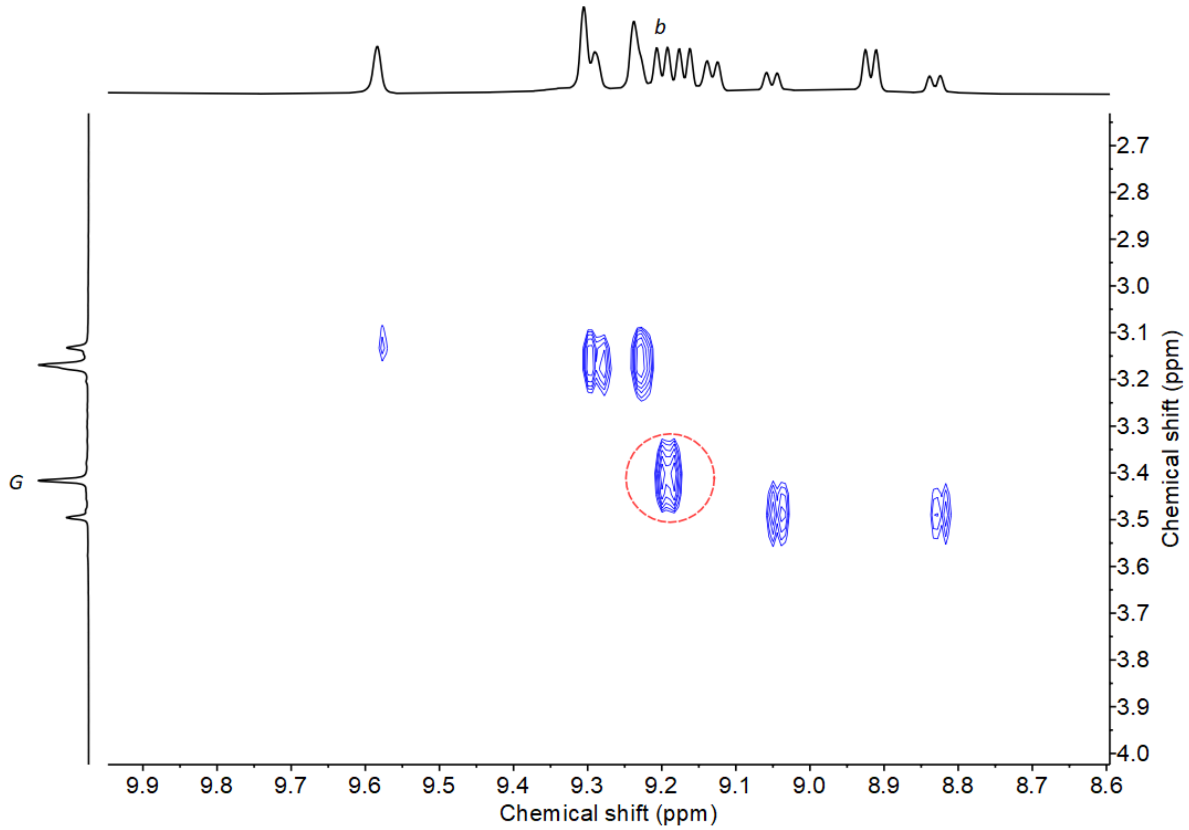

Figure S145 Partial NOESY Partial NOESY (400 MHz, CD_3CN) of [$Pd_2(1AC)_2(4AA)_2](BF_4)_4$ with peaks assigned to major *syn*-isomer.

Minor Isomer *anti*-$[Pd_2(1AC)_2(4AA)_2](BF_4)_4$

^{1}H NMR (400 MHz, CD_3CN) δ: 10.13 (s, 2H, H_H), 10.04 (s, 2H, H_A), 9.28 (s, 2H, H_a), 9.23 (m, 2H, $H_{a'}$), 9.13 (d, *J* = 5.9 Hz, 2H, H_b), 9.05 (d, *J* = 5.9 Hz, 2H, $H_{b'}$), 8.83 (d, *J* = 5.8 Hz, 2H, H_B), 8.11-7.96 (m, 6H, H_d, $H_{d'}$, H_D), 7.95-7.87 (m, 2H, H_E), 7.71 (m, 2H, $H_{c'}$), 7.64 (m, 2H, H_c), 7.60-7.48 (m, 6H, H_e, $H_{e'}$, H_C), 7.44 (d, *J* = 8.1 Hz, 2H, H_F), 7.27 (t, *J* = 7.7 Hz, 2H, H_f), 3.49 (s, 6H, H_G), 3.16 (s, 6H, H_g).

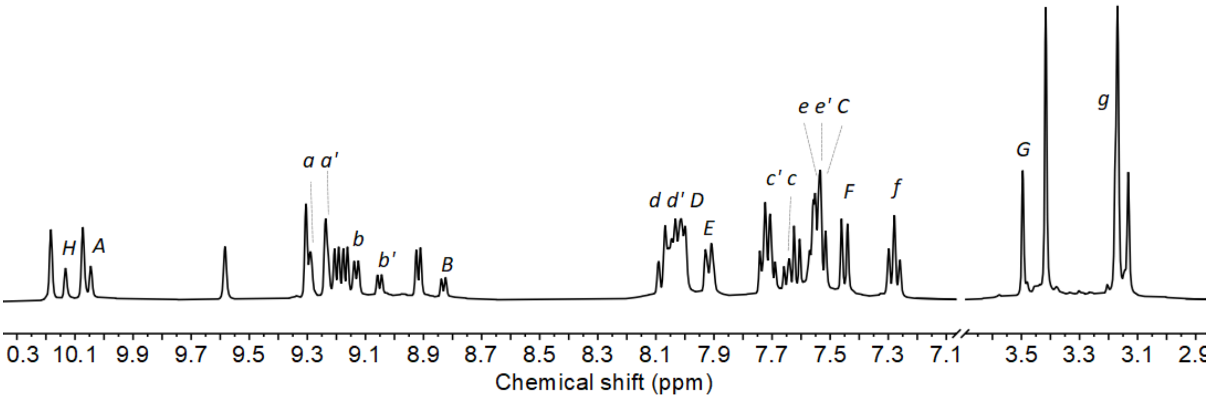

Figure S146 Partial ^{1}H NMR (400 MHz, CD_3CN) of $[Pd_2(1AC)_2(4AA)_2](BF_4)_4$ with peaks of minor *anti*-isomer labelled.

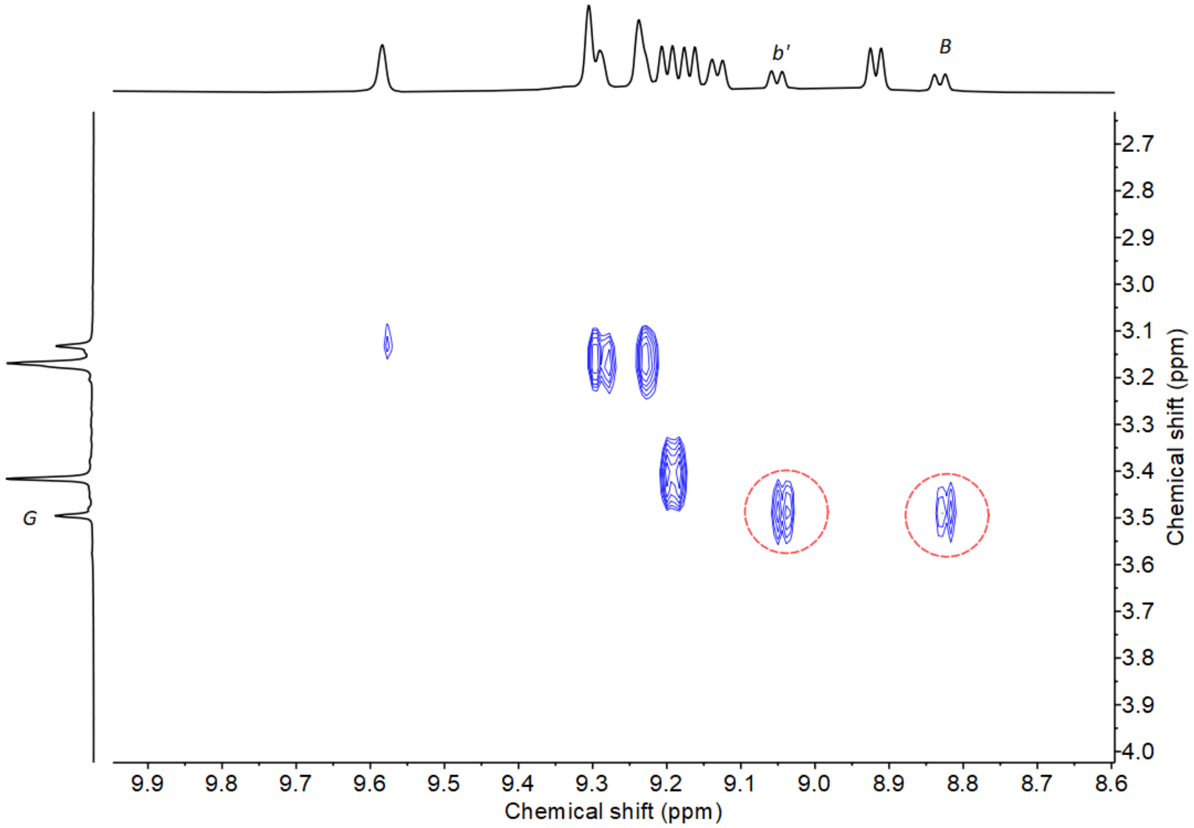

Figure S147 Partial NOESY Partial NOESY (400 MHz, CD_3CN) of $[Pd_2(1AC)_2(4AA)_2](BF_4)_4$ with peaks assigned to minor *anti*-isomer.

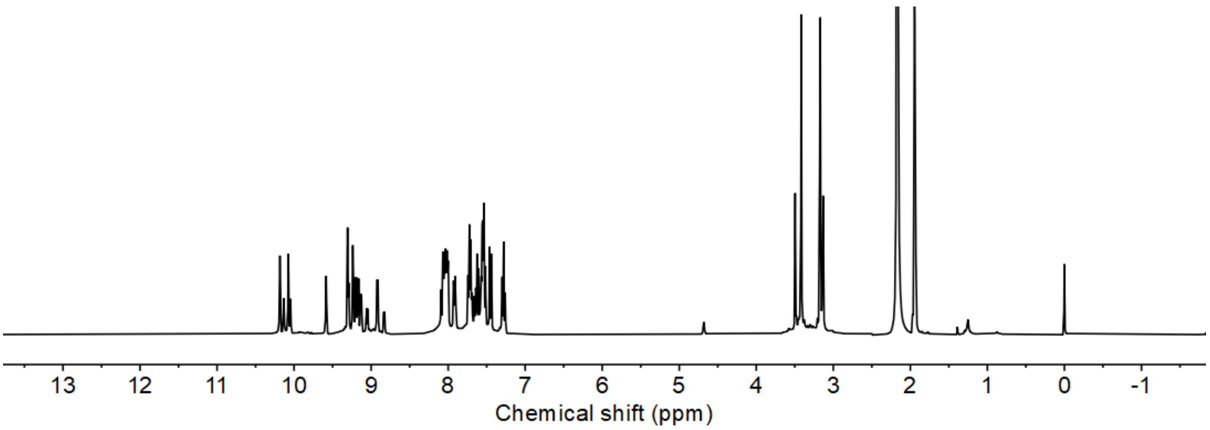

Figure S148 ^{1}H NMR (400 MHz, CD_3CN) of $[Pd_2(1AC)_2(4AA)_2](BF_4)_4$.

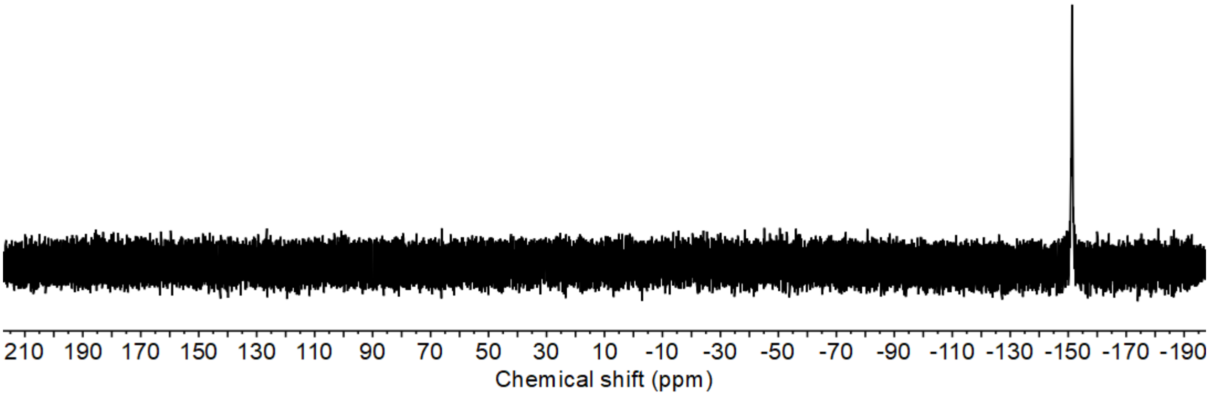

Figure S149 ^{19}F NMR (376 MHz, CD_3CN) of $[Pd_2(1AC)_2(4AA)_2](BF_4)_4$.

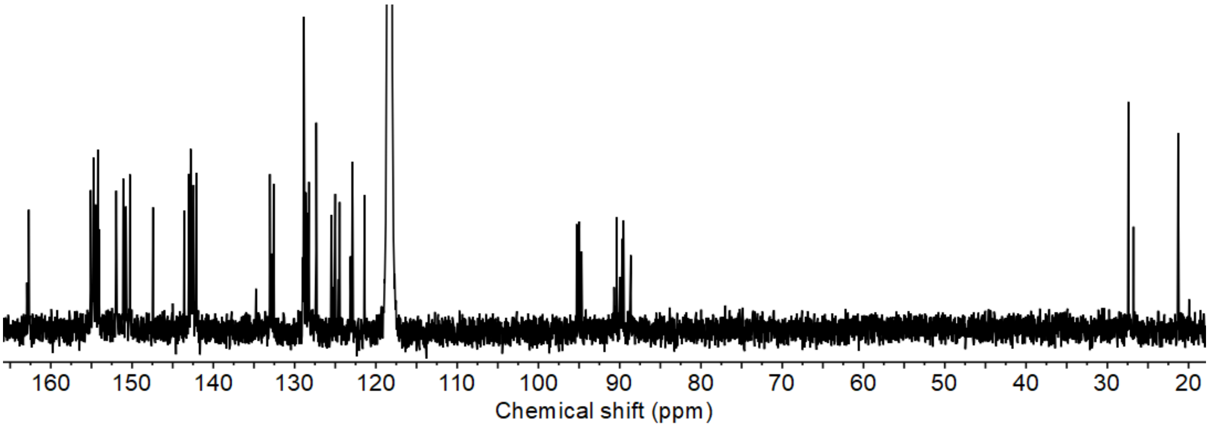

Figure S150 Partial ^{13}C NMR (101 MHz, CD_3CN) of $[Pd_2(1AC)_2(4AA)_2](BF_4)_4$.

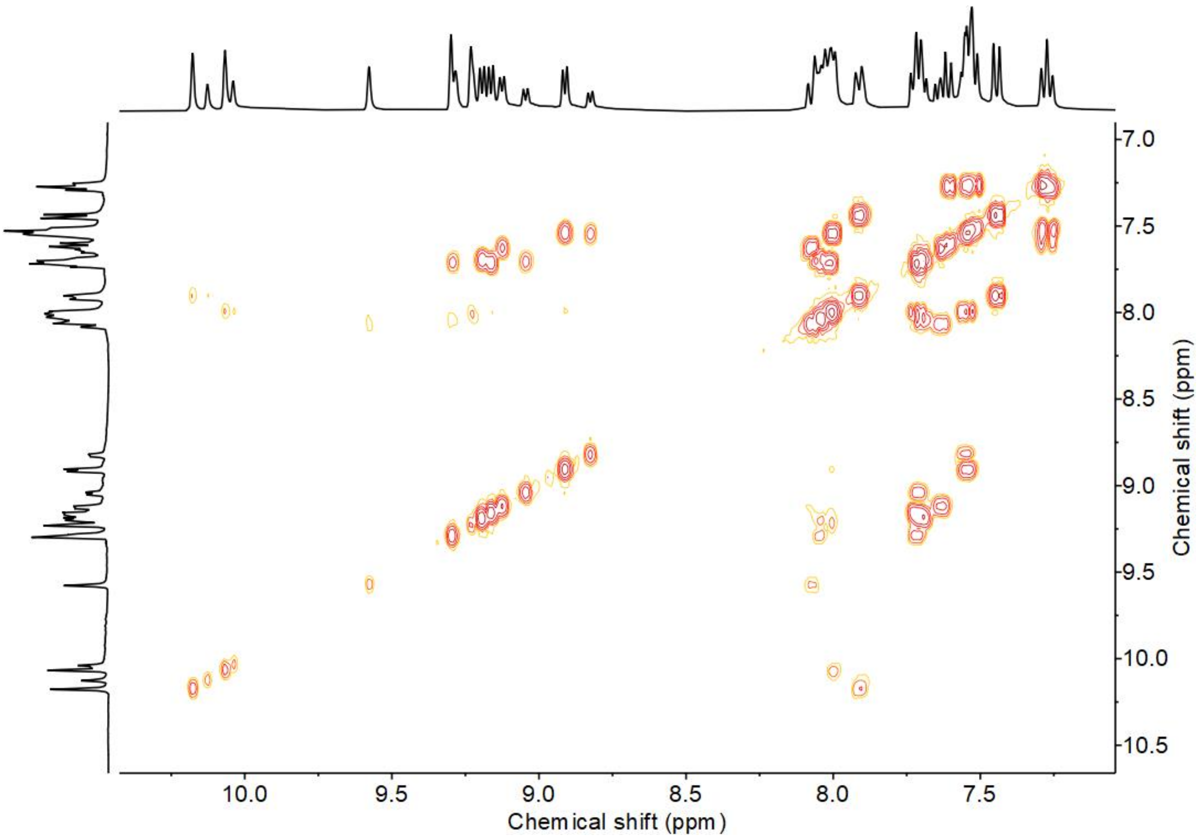

Figure S151 Partial COSY (CD_3CN) of $[Pd_2(1AC)_2(4AA)_2](BF_4)_4$.

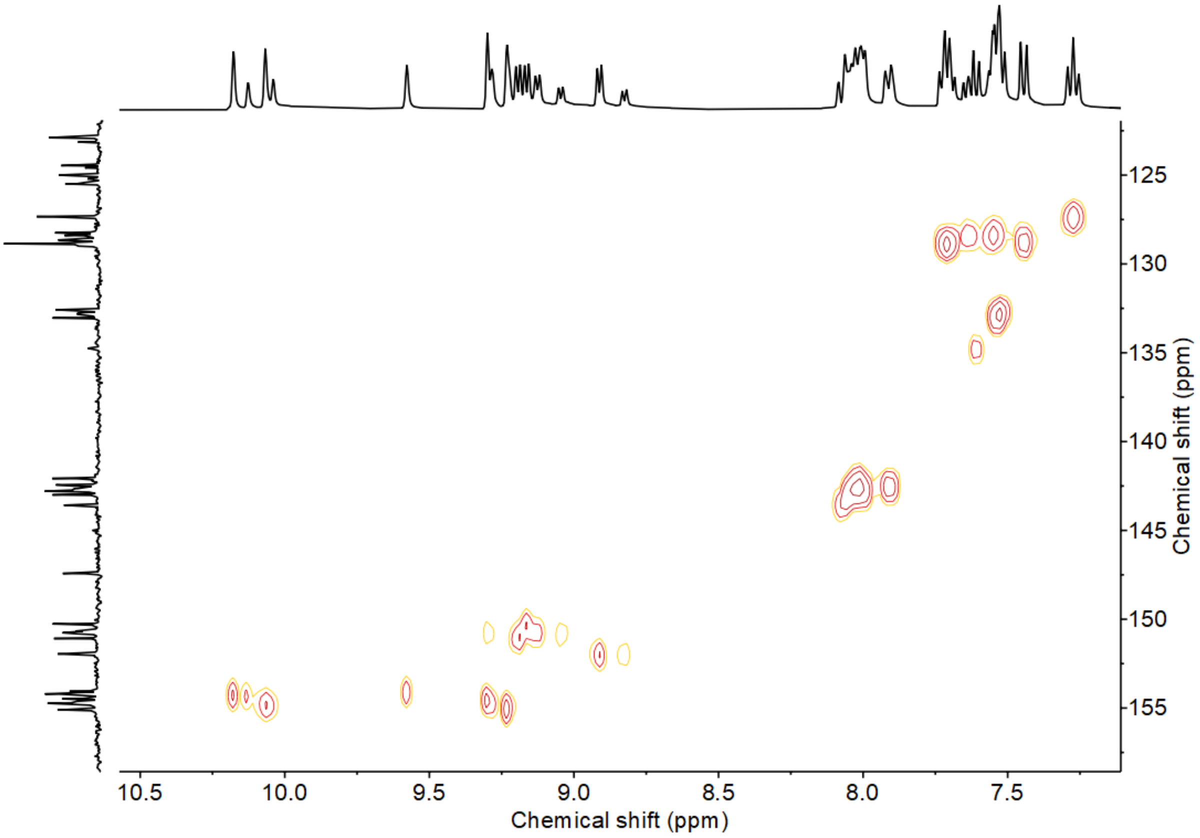

Figure S152 Partial HSQC (CD_3CN) of $[Pd_2(1AC)_2(4AA)_2](BF_4)_4$.

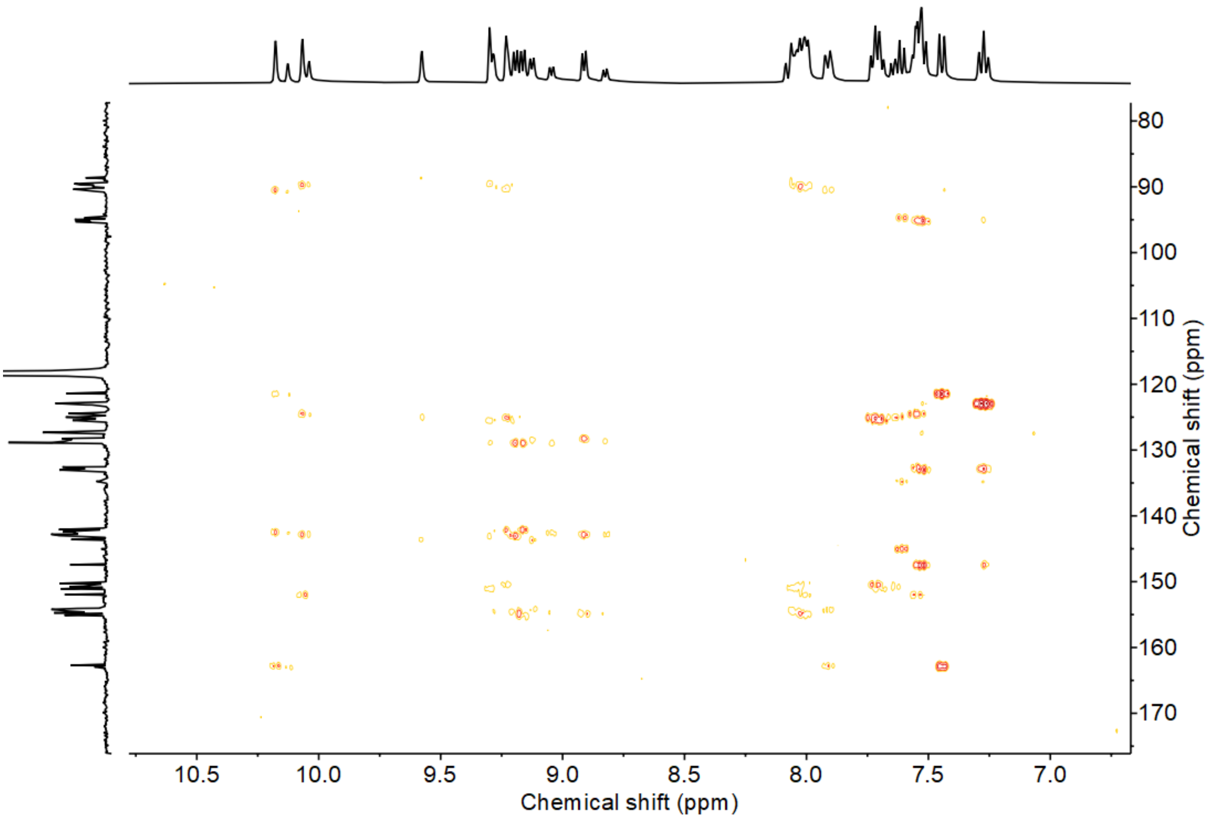

Figure S153 Partial HMBC (CD_3CN) of $[Pd_2(1AC)_2(4AA)_2](BF_4)_4$.

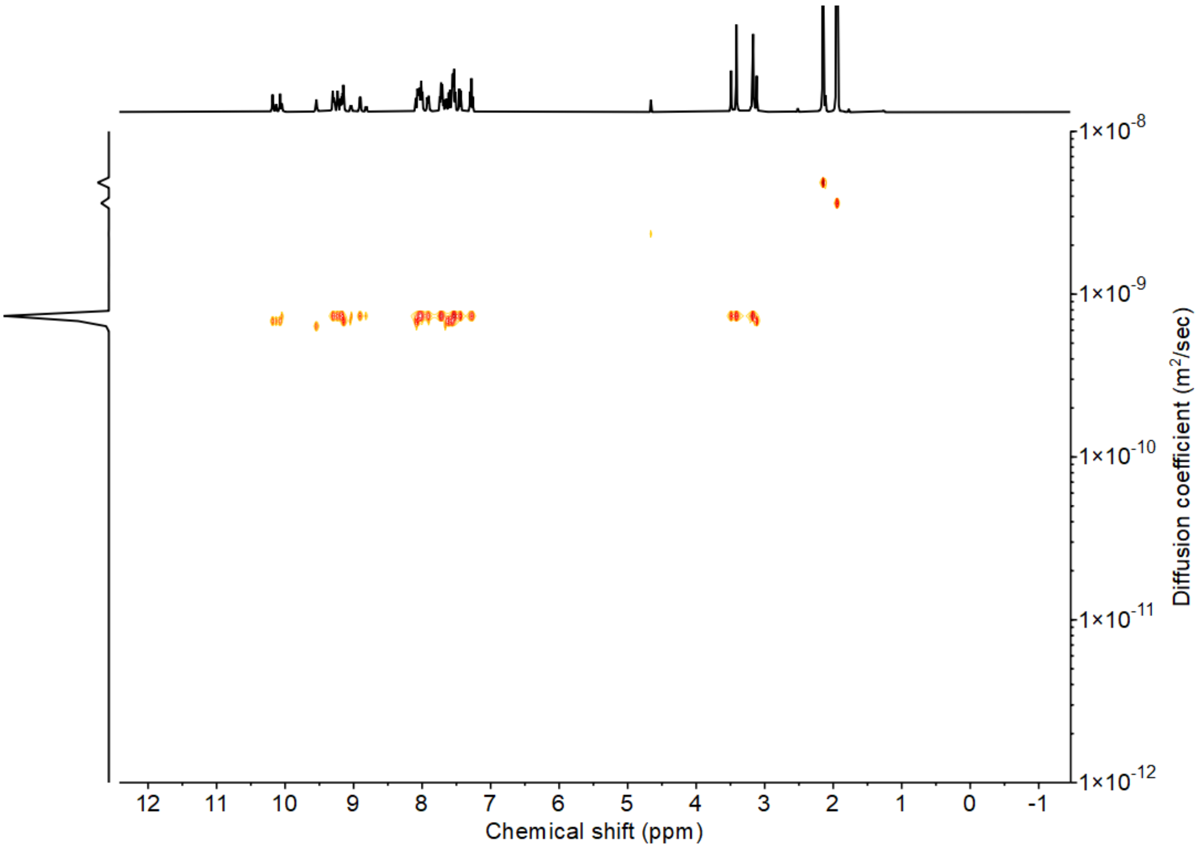

Figure S154 DOSY (CD_3CN) of $[Pd_2(1AC)_2(4AA)_2](BF_4)_4$.

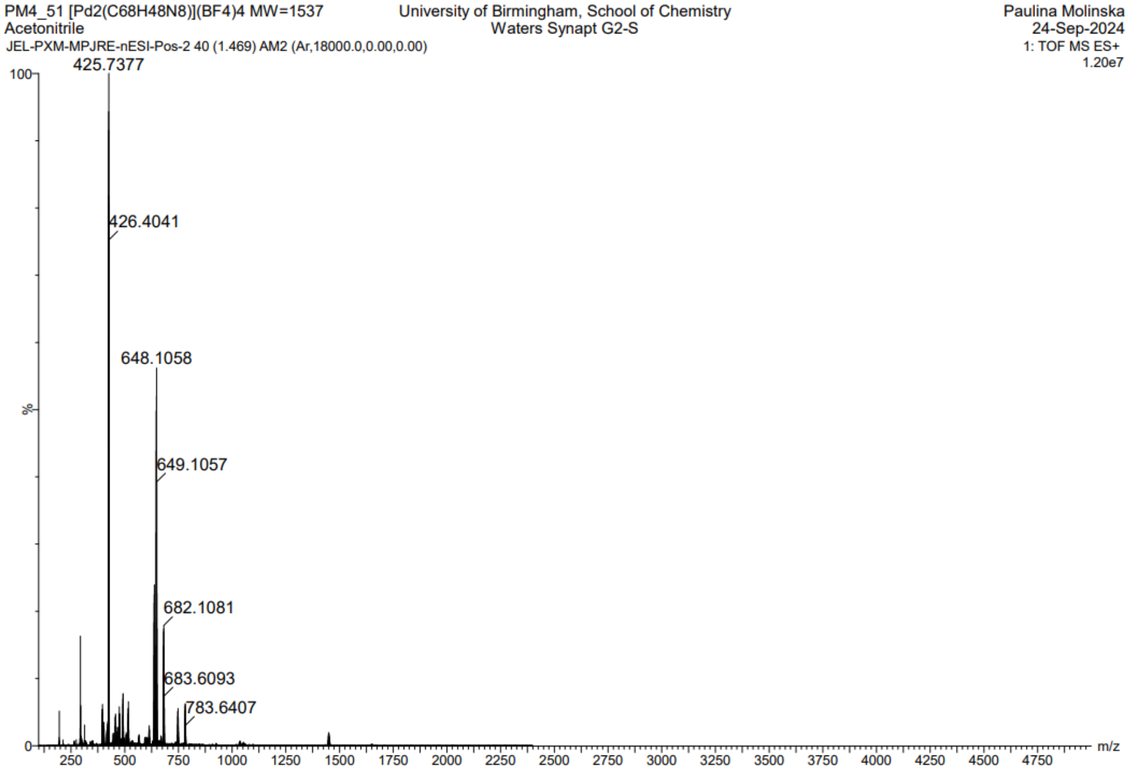

Figure S155 ESI-MS of $[Pd_2(1AC)_2(4AA)_2](BF_4)_4$.

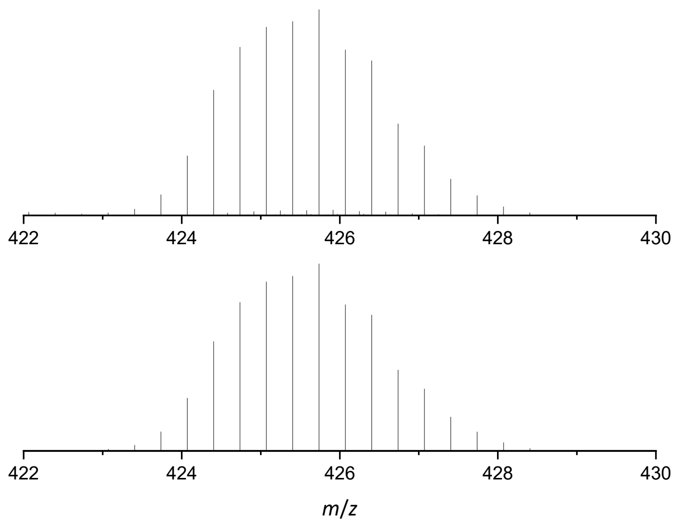

Figure S156 Observed (top) and calculated (bottom) isotopic patterns for $\{[Pd_2(1AC)_2(4AA)_2](BF_4)\}^{3+}$.

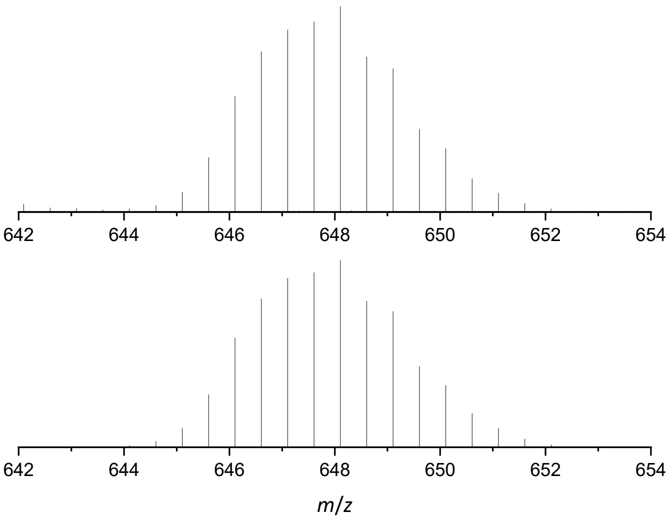

Figure S157 Observed (top) and calculated (bottom) isotopic patterns for {[$Pd_2(1AC)_2(4AA)_2$](BF_4)F}$^{2+}$.

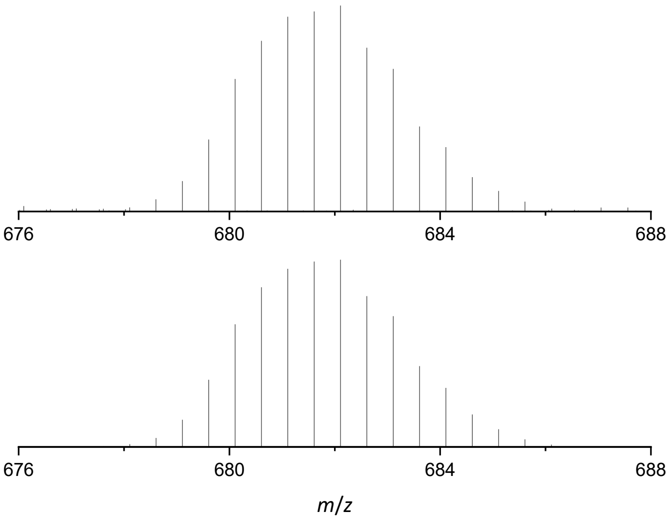

Figure S158 Observed (top) and calculated (bottom) isotopic patterns for {[$Pd_2(1AC)_2(4AA)_2$](BF_4)$_2$}$^{2+}$.

Synthesis of $[Pd_2(\mathbf{1BC})_2(\mathbf{2AA})_2](BF_4)_4$

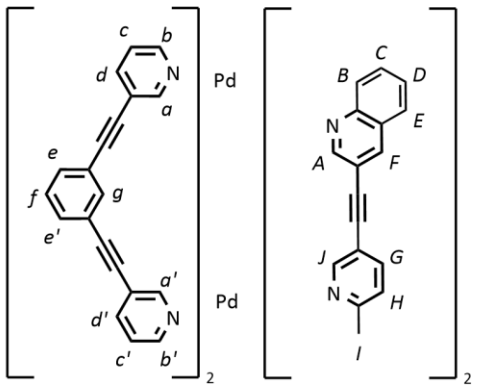

1BC (7.3 mg, 30 µmol, 1 eq.), **2AA** (8.4 mg, 30 µmol, 1 eq.) and $[Pd(CH_3CN)_4](BF_4)_2$ (13.3 mg, 30 µmol, 1 eq.) were stirred at 70 °C in anhydrous CH_3CN (5.0 mL) under N_2 for 24 h. The cooled reaction mixture was filtered through celite. Vapour diffusion of Et_2O into this solution yielded a precipitate. After the mother liquor was decanted, the solid was washed with Et_2O (× 3) and dried *in vacuo* to give the product as a beige solid (18.8 mg, 78%).

ESI-MS *m/z* = 449.07 $\{[Pd_2(\mathbf{1BC})_2(\mathbf{2AA})_2](BF_4)\}^{3+}$ calc.449.74; 684.11 $\{[Pd_2(\mathbf{1BC})_2(\mathbf{2AA})_2](BF_4)F\}^{2+}$ calc. 684.11; 718.11 $\{[Pd_2(\mathbf{1BC})_2(\mathbf{2AA})_2](BF_4)_2\}^{2+}$ calc 718.11.

^{13}C NMR (101 MHz, CD_3CN) δ: 162.78, 156.28 (*syn*-C_A/ *anti*-C_A), 154.96 (*syn*-C_a, *syn*-$C_{a'}$, *anti*-C_a, *anti*-$C_{a'}$), 153.31 (*syn*-C_J, *anti*-C_J), 151.07 (*syn*-$C_{b'}$), 150.97 (*syn*-C_b, *anti*-C_b, *anti*-$C_{b'}$), 146.28, 146.13, 144.11 (*syn*-C_F/ *anti*-C_F), 143.03 (*syn*-C_b/ *syn*-$C_{b'}$/ *anti*-C_b/ *anti*-$C_{b'}$/ *syn*-C_G/ *anti*-C_G), 142.95 (*syn*-C_b/ *syn*-$C_{b'}$/ *anti*-C_b/ *anti*-$C_{b'}$/ *syn*-C_G/ *anti*-C_G), 142.89 (*syn*-C_b/ *syn*-$C_{b'}$/ *anti*-C_b/ *anti*-$C_{b'}$/ *syn*-C_G/ *anti*-C_G), 142.78 (*syn*-C_b/ *syn*-$C_{b'}$/ *anti*-C_b/ *anti*-$C_{b'}$/ *syn*-C_G/ *anti*-C_G), 139.27 (*syn*-C_g/ *anti*-C_g), 135.69 (*syn*-C_C/ *anti*-C_C), 132.58 (*syn*-C_e/ *syn*-$C_{e'}$, *anti*-C_e/ *anti*-$C_{e'}$), 132.54 (*syn*-C_e/ *syn*-$C_{e'}$, *anti*-C_e/ *anti*-$C_{e'}$), 131.13 (*syn*-C_E/ *syn*-C_D/ *anti*-C_E/ *anti*-C_D), 131.01 (*syn*-C_E/ *syn*-C_D/ *anti*-C_E/ *anti*-C_D), 130.79 (*syn*-C_f, *anti*-C_f), 130.29, 130.16, 129.12 (*anti*-C_c), 128.95 (*syn*-$C_{c'}$, *syn*-C_H, *anti*-C_H), 128.82 (*syn*-C_c), 128.66 (*anti*-$C_{c'}$), 127.42 (*syn*-C_B/ *anti*-C_B), 127.37 (*syn*-C_B/ *anti*-C_B), 125.25, 125.07, 125.00, 123.02, 121.61, 121.50, 119.50, 119.38, 95.60, 95.52, 95.43, 90.29, 89.89, 85.82, 85.75, 27.49 (*syn*-C_I/ *anti*-C_I), 27.25 (*syn*-C_I/ *anti*-C_I).

^{19}F NMR (376 MHz, CD_3CN) δ: -151.51.

^{1}H DOSY (400 MHz, CD_3CN) *D*: 7.4×10^{-10} m^2s^{-1}; R_S: 8.3 Å.

Isomer *syn*-[$Pd_2(1BC)_2(2AA)_2$]$(BF_4)_4$

^{1}H NMR (600 MHz, CD_3CN) δ: 10.48 (s, 2H, H_A), 10.17 (d, *J* = 8.7 Hz, 2H, H_B), 9.48-9.43 (m, 4H, H_b, H_a), 9.40 (s, 2H, $H_{a'}$), 9.18 (dd, *J* = 5.9, 1.3 Hz, 2H, $H_{b'}$), 8.62 (s, 2H, H_F), 8.34 (m, 2H, H_C), 8.25 (app. d, *J* = 1.9 Hz, 2H, H_g), 8.06 (app. dt, *J* = 8.0, 1.6 Hz, 2H, $H_{d'}$), 7.99 (m, 6H, H_G, H_d, H_E), 7.86-7.80 (m, 2H, H_D), 7.70 (dd, *J* = 8.1, 5.9 Hz, 2H, $H_{c'}$), 7.66 (dd, *J* = 8.1, 5.9 Hz, 2H, H_c), 7.59 (m, 4H, H_e, $H_{e'}$), 7.51-7.48 (m, 4H, H_f, H_H), 3.46 (s, 6H, H_I).

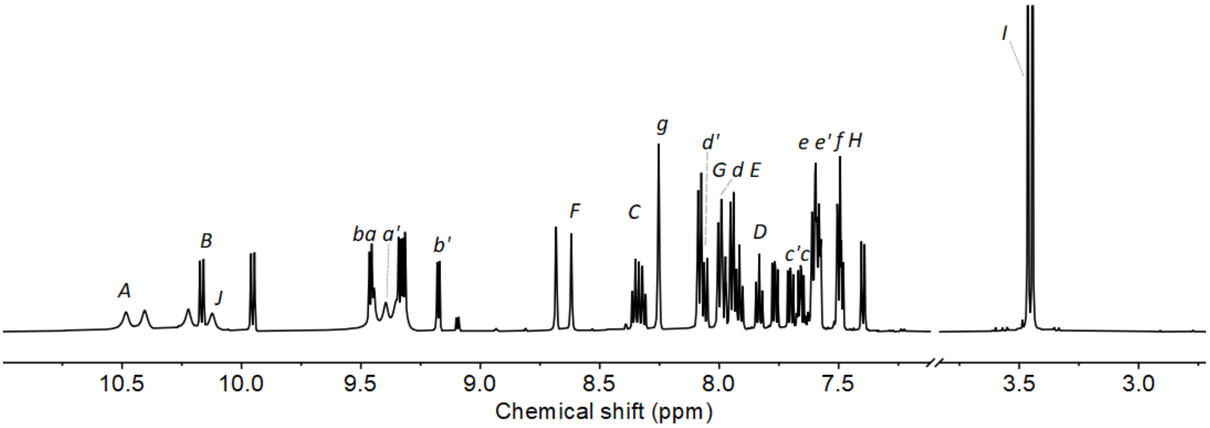

Figure S159 Partial ^{1}H NMR (600 MHz, CD_3CN) of [$Pd_2(1BC)_2(2AA)_2$]$(BF_4)_4$ with peaks of the *syn*-isomer labelled.

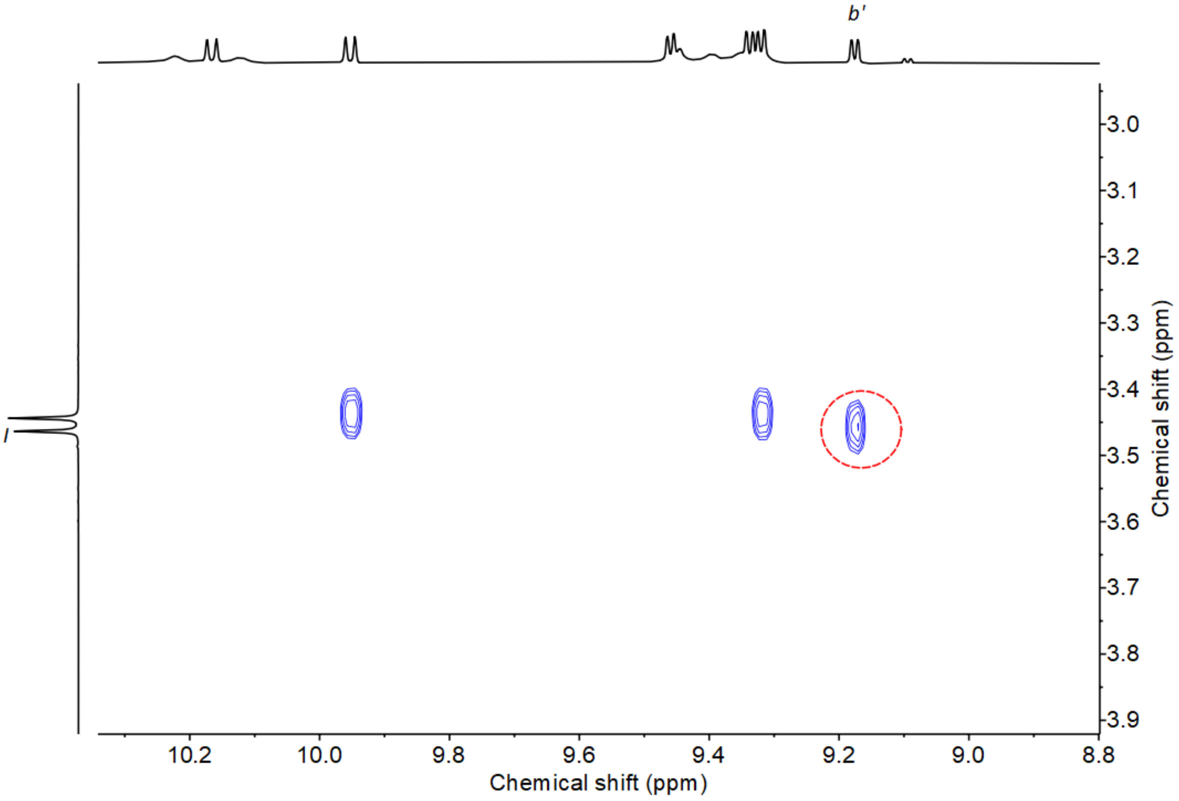

Figure S160 Partial NOESY Partial NOESY (600 MHz, CD_3CN) of [$Pd_2(1BC)_2(2AA)_2$]$(BF_4)_4$ labelled with through-space interactions used to identify it as the *syn*-isomer.

Isomer *anti*-[$Pd_2(1BC)_2(2AA)_2](BF_4)_4$

^{1}H NMR (600 MHz, CD_3CN) δ: 10.41 (s, 2H, H_A), 10.22 (s, 2H, H_J), 9.95 (d, *J* = 8.7 Hz, 2H, H_B), 9.33 (m, 8H, H_b, $H_{b'}$, H_a, $H_{a'}$), 8.70-8.67 (m, 2H, H_F), 8.34 (m, 2H, H_C), 8.25 (app. d, *J* = 1.9 Hz, 2H, H_g), 8.08 (m, 4H, H_d, H_E), 7.95 (m, 4H, H_G, $H_{d'}$), 7.93-7.89 (m, 2H, H_D), 7.77 (dd, *J* = 8.0, 5.8 Hz, 2H, H_c), 7.59 (m, 6H, $H_{c'}$, H_e, $H_{e'}$), 7.51-7.48 (m, 2H, H_f), 7.40 (d, *J* = 8.1 Hz, 2H, H_H), 3.44 (s, 6H, H_I).

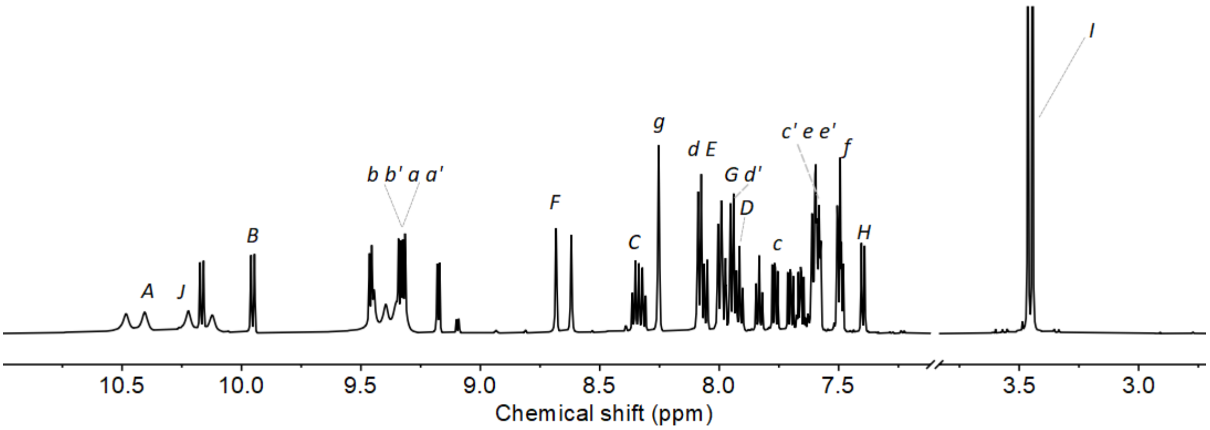

Figure S161 Partial ^{1}H NMR (600 MHz, CD_3CN) of [$Pd_2(1BC)_2(2AA)_2](BF_4)_4$ with peaks of the *anti*-isomer labelled.

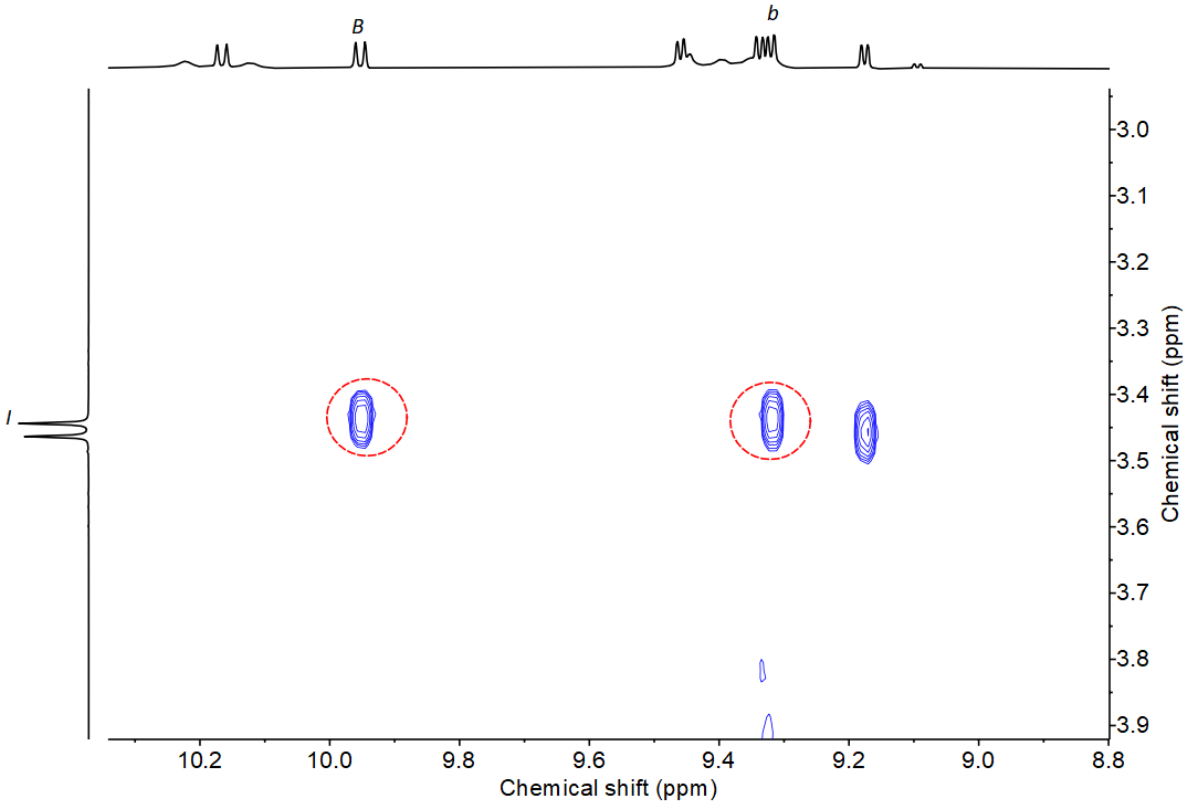

Figure S162 Partial NOESY Partial NOESY (600 MHz, CD_3CN) of [$Pd_2(1BC)_2(2AA)_2](BF_4)_4$ labelled with through-space interactions used to identify it as the *anti*-isomer.

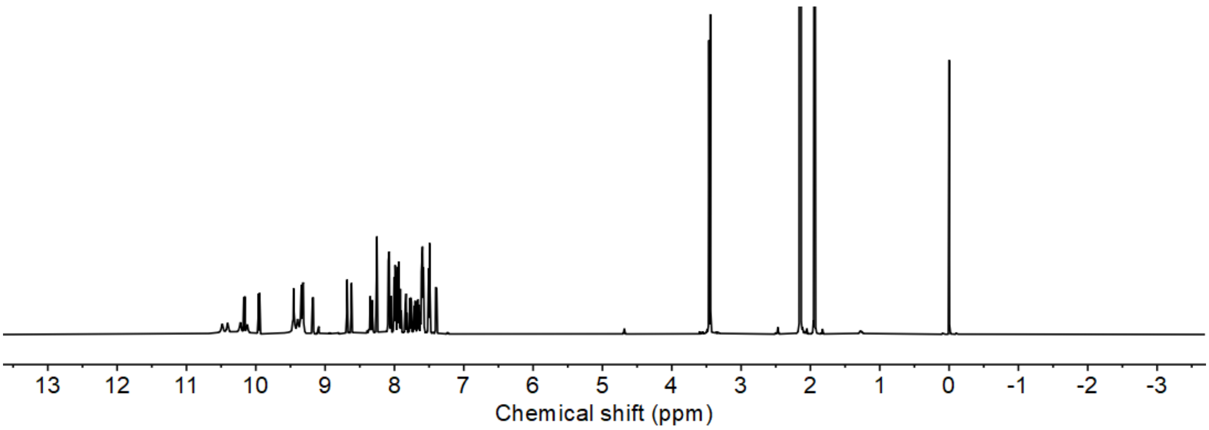

Figure S163 ^{1}H **NMR (600 MHz, CD_3CN) of [$Pd_2(1BC)_2(2AA)_2$]$(BF_4)_4$.**

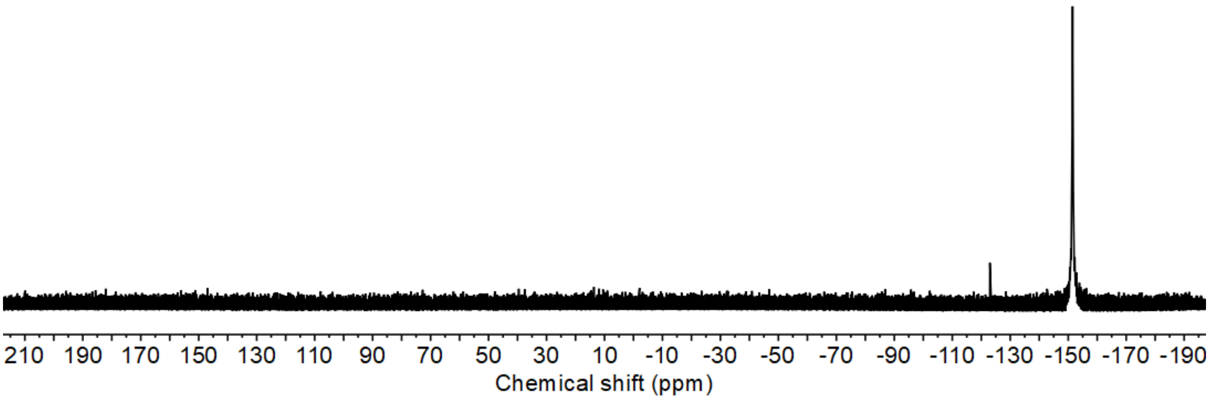

Figure S164 ^{19}F **NMR (376 MHz, CD_3CN) of [$Pd_2(1BC)_2(2AA)_2$]$(BF_4)_4$.**

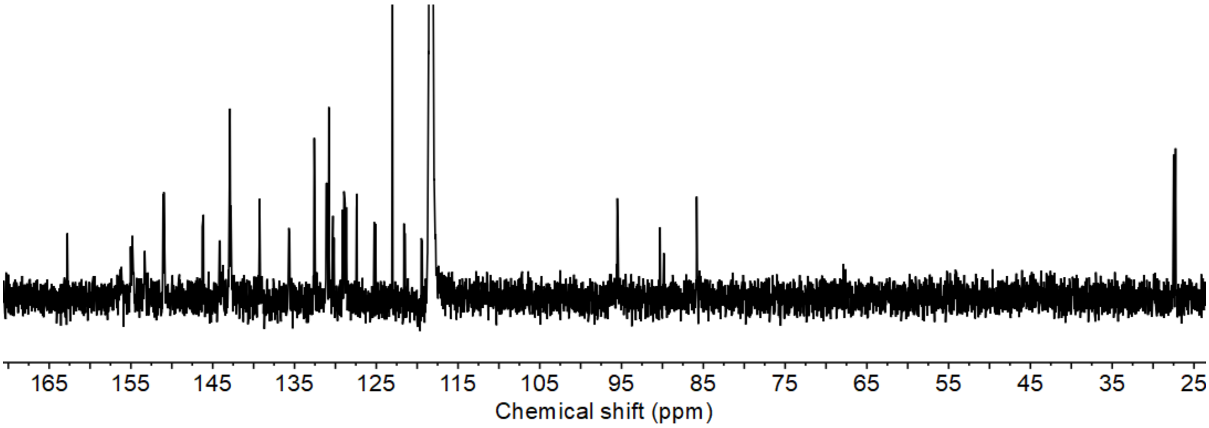

Figure S165 Partial ^{13}C **NMR (101 MHz, CD_3CN) of [$Pd_2(1BC)_2(2AA)_2$]$(BF_4)_4$.**

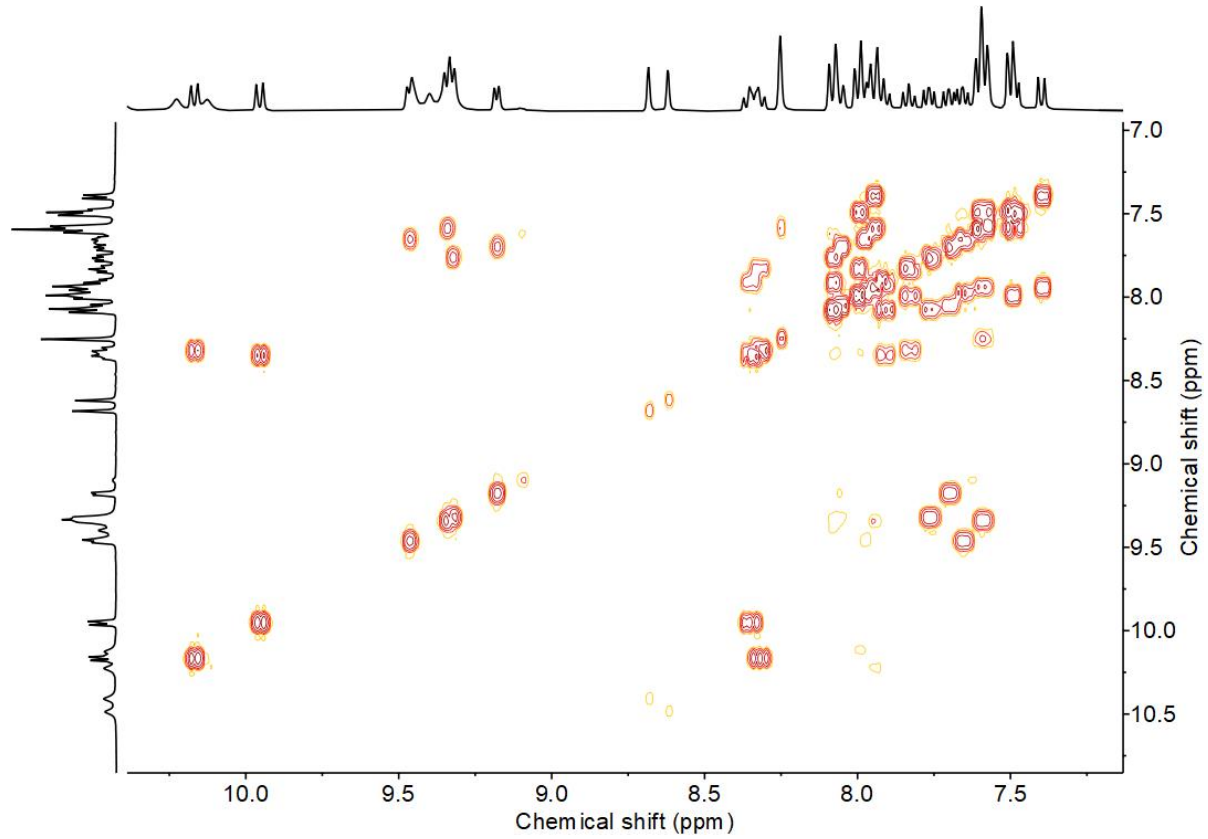

Figure S166 Partial COSY (CD_3CN) of $[Pd_2(1BC)_2(2AA)_2](BF_4)_4$.

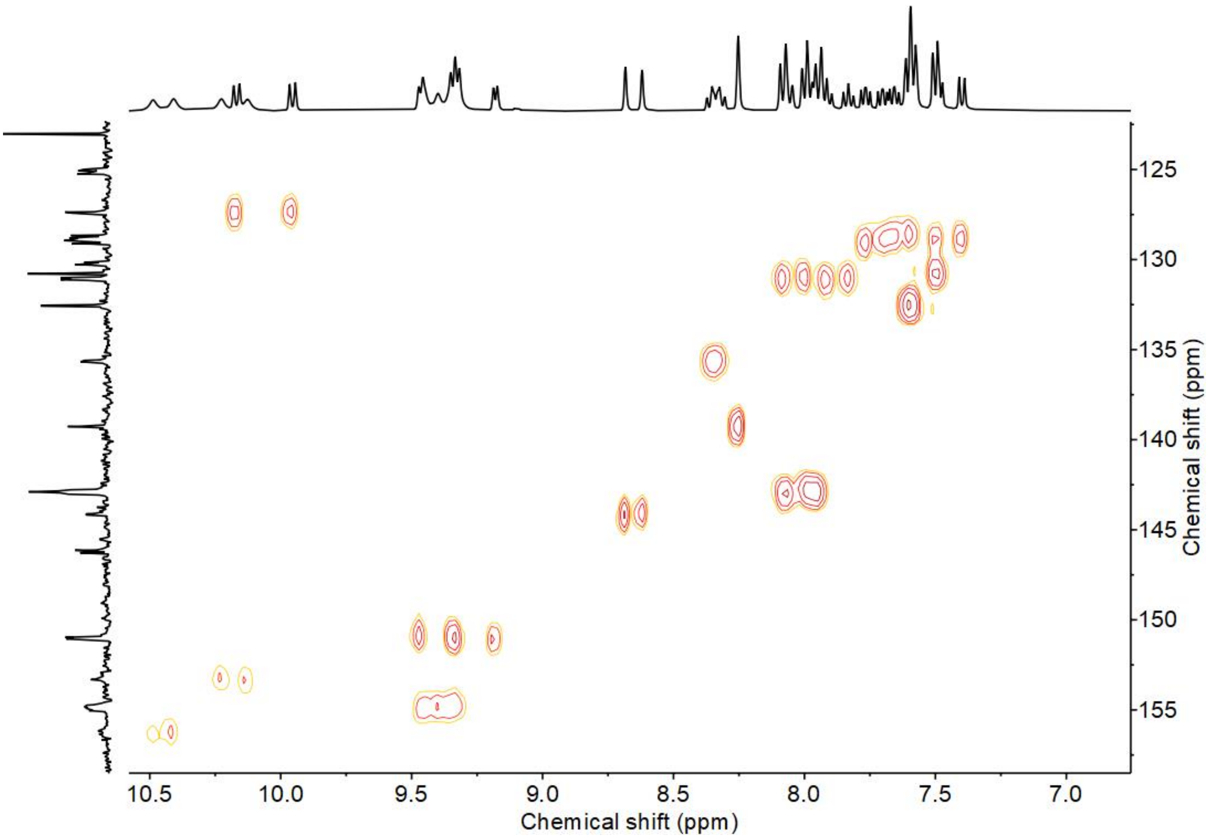

Figure S167 Partial HSQC (CD_3CN) of $[Pd_2(1BC)_2(2AA)_2](BF_4)_4$.

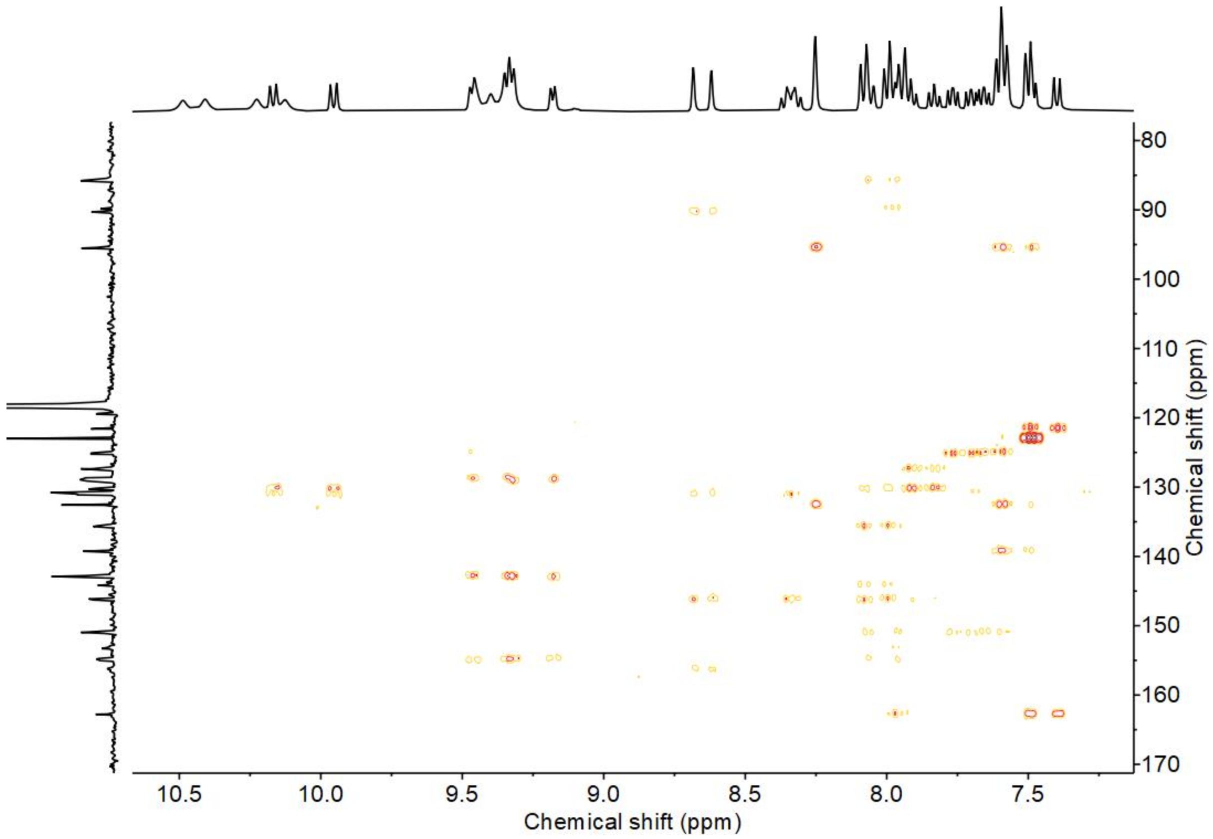

Figure S168 Partial HMBC (CD_3CN) of $[Pd_2(1BC)_2(2AA)_2](BF_4)_4$.

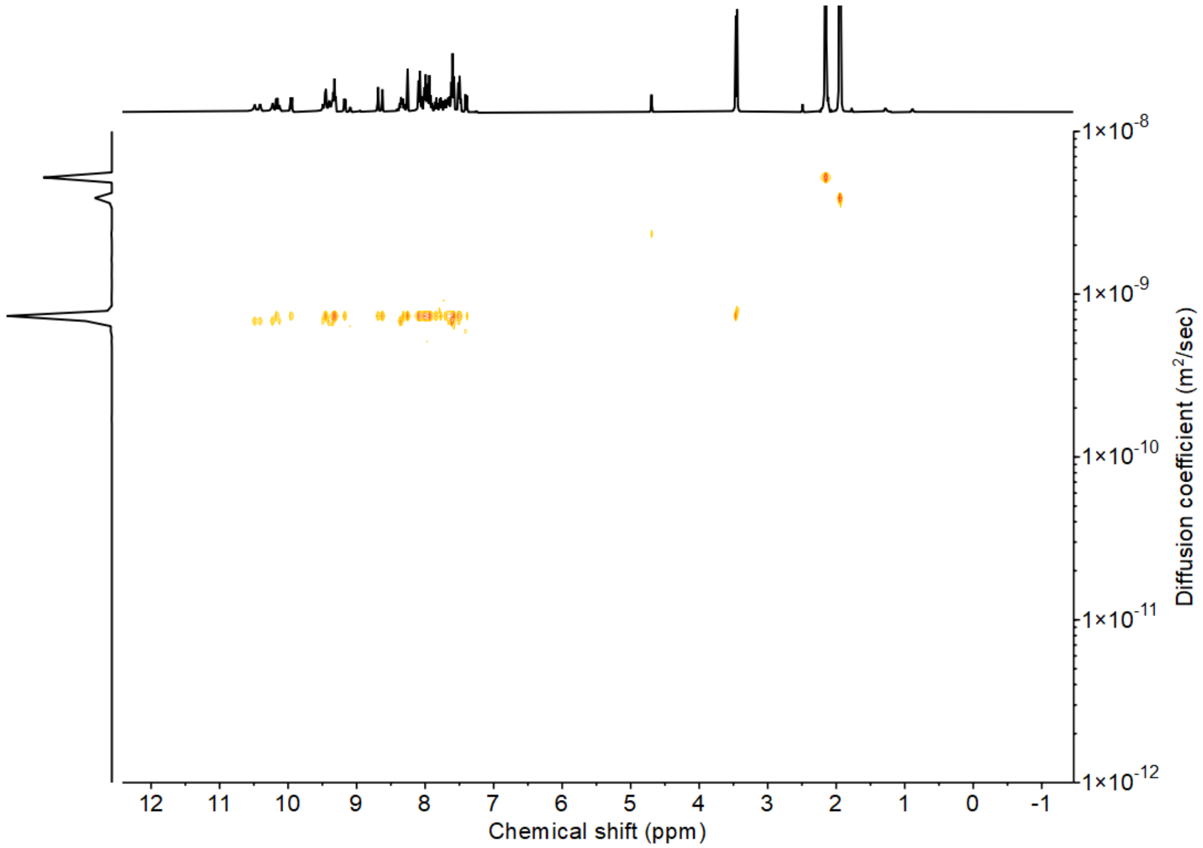

Figure S169 DOSY (CD_3CN) of $[Pd_2(1BC)_2(2AA)_2](BF_4)_4$.

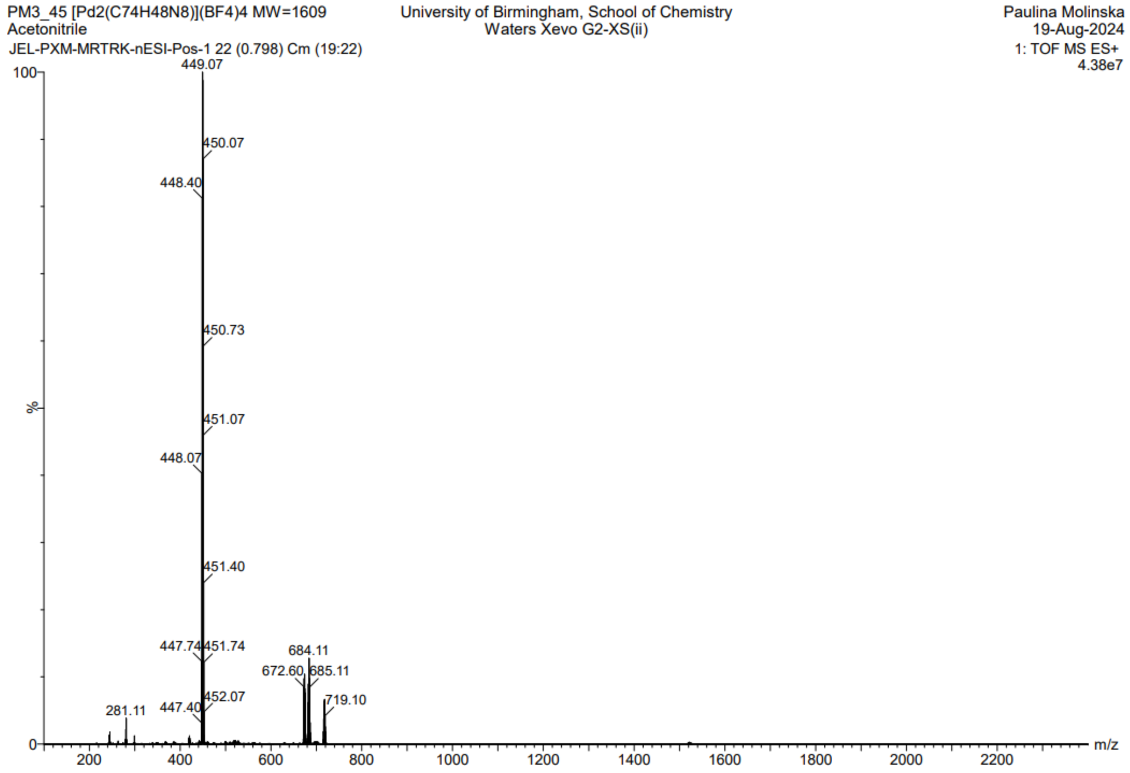

Figure S170 ESI-MS of $[Pd_2(1BC)_2(2AA)_2](BF_4)_4$.

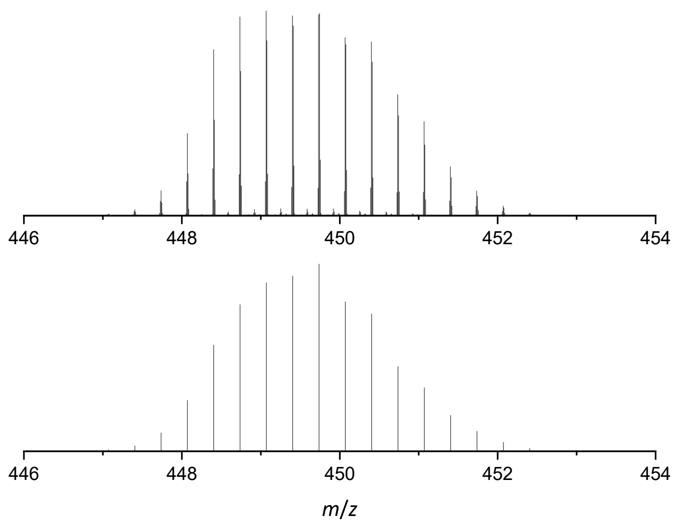

Figure S171 Observed (top) and calculated (bottom) isotopic patterns for $\{[Pd_2(1BC)_2(2AA)_2](BF_4)\}^{3+}$.

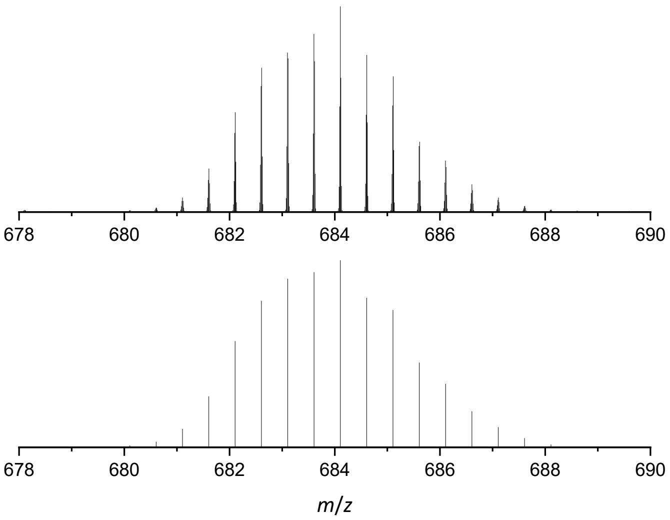

Figure S172 Observed (top) and calculated (bottom) isotopic patterns for $\{[Pd_2(1BC)_2(2AA)_2](BF_4)F\}^{2+}$.

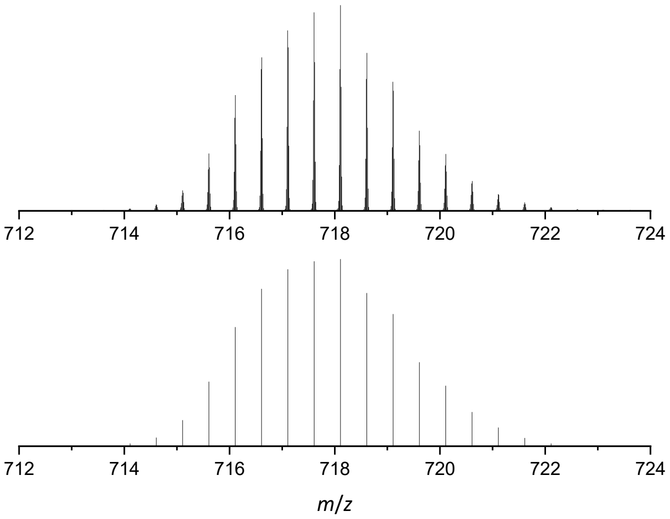

Figure S173 Observed (top) and calculated (bottom) isotopic patterns $\{[Pd_2(1BC)_2(2AA)_2](BF_4)_2\}^{2+}$.

Synthesis of $[Pd_2(\mathbf{1BD})_2(\mathbf{2AA})_2](BF_4)_4$

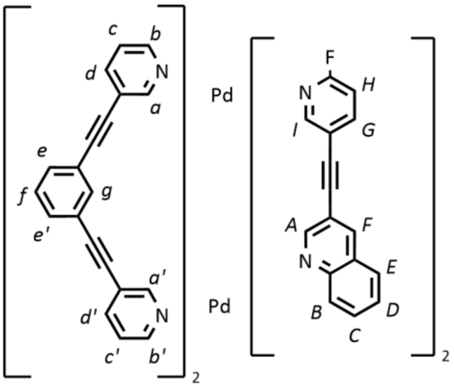

2AA (8.4 mg, 30 µmol, 1 eq.), **1BD** (7.4 mg, 30 µmol, 1 eq.) were combined in a vial to which a 40 mM stock solution of $[Pd(CH_3CN)_4](BF_4)_2$ (750 µL, 30 µmol, 1 eq.) in CD_3CN was added. A homogenous solution was obtained following sonication which was then transferred to a 5 mm NMR tube. The reaction was heated at 70 °C for 17 h. The formation of multiple species was observed.

ESI-MS *m/z* = 452.39 $\{[Pd_2(\mathbf{1BD})_2(\mathbf{2AA})_2](BF_4)\}^{3+}$ calc. 452.39; 688.08 $\{[Pd_2(\mathbf{1BD})_2(\mathbf{2AA})_2](BF_4)F\}^{2+}$ calc. 688.08.

^{19}F NMR (376 MHz, CD_3CN) δ: -60.98 (d, *J* = 8.4 Hz), -61.15 (d, *J* = 7.8 Hz), -66.10, -66.17--66.58 (m), -150.90.

Major Isomer *anti*-$[Pd_2(1BD)_2(2AA)_2](BF_4)_4$:

^{1}H NMR (600 MHz, CD_3CN) δ: 10.29 (d, *J* = 1.7 Hz, 2H, H_A), 10.25-10.21 (m, 2H, H_B), 9.94 (d, *J* = 2.2 Hz, 2H, H_I), 9.45 (d, *J* = 1.7 Hz, 2H, H_a), 9.42 (d, *J* = 5.9 Hz, 2H, $H_{b'}$), 9.37 (d, *J* = 1.7 Hz, 2H, $H_{a'}$), 9.24 (ddd, *J* = 6.0, 1.3, 0.6 Hz, 2H, H_b), 8.68 (m, 2H, H_F), 8.41 (ddd, *J* = 8.6, 7.1, 1.4 Hz, 2H, H_C), 8.24 (td, *J* = 1.7, 0.6 Hz, 2H, H_g), 8.20 (ddd, *J* = 8.7, 6.5, 2.2 Hz, 2H, H_G), 8.11-8.07 (m, 4H, H_E, $H_{d'}$), 7.98-7.93 (m, 4H, H_d, H_D), 7.77 (ddd, *J* = 8.0, 5.9, 0.6 Hz, 2H, $H_{c'}$), 7.61-7.57 (m, 6H, H_e, $H_{e'}$, H_c), 7.50 (m, 2H, H_f), 7.34 (d, *J* = 8.6 Hz, 2H, H_H).

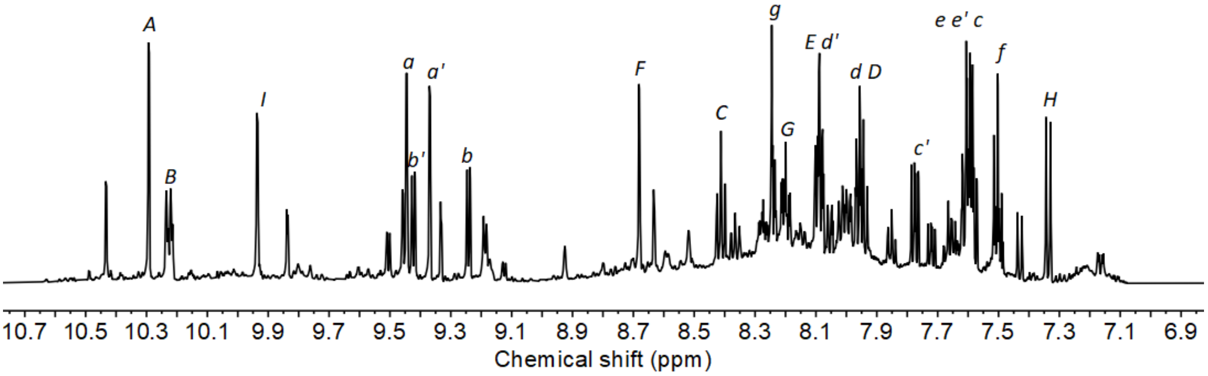

Figure S174 Partial ^{1}H NMR (600 MHz, CD_3CN) of $[Pd_2(1BD)_2(2AA)_2](BF_4)_4$ with peaks of major *anti*-isomer labelled.

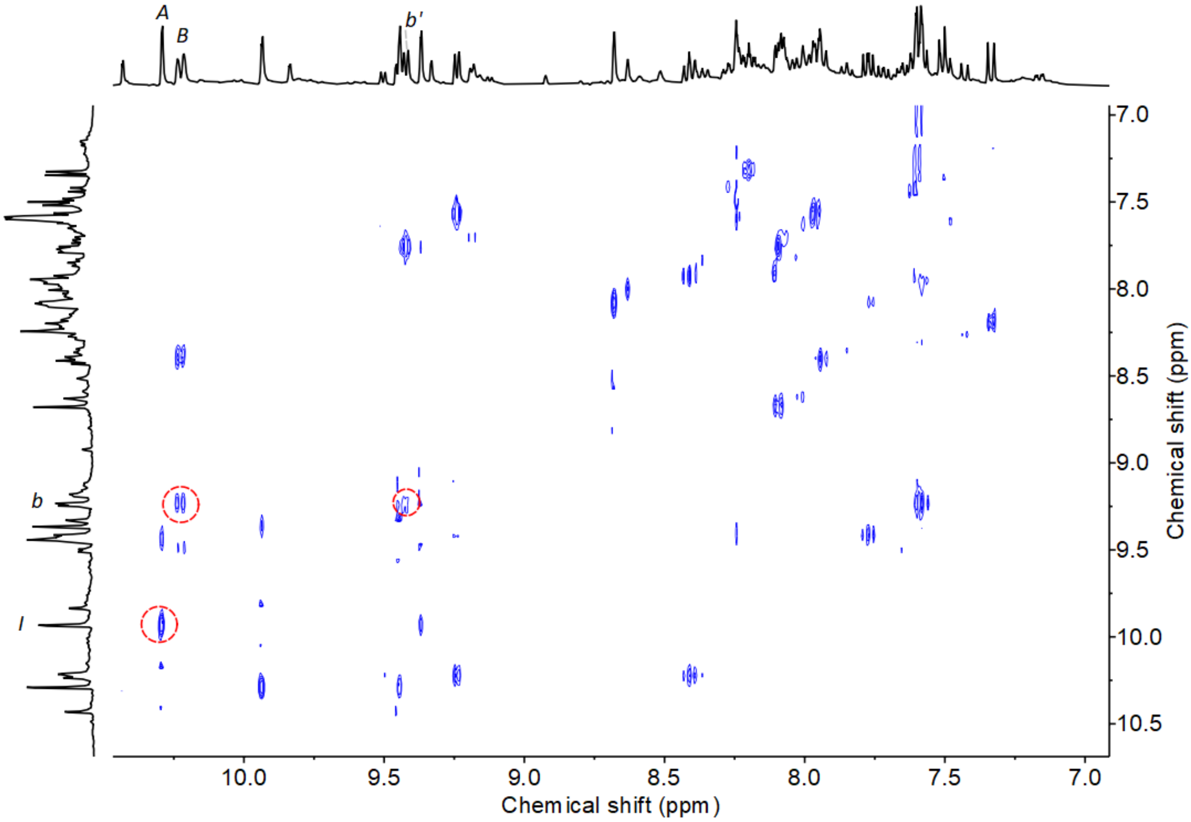

Figure S175 Partial NOESY (400 MHz, CD_3CN) of $[Pd_2(1BD)_2(2AA)_2](BF_4)_4$ with peaks assigned to major *anti*-isomer.

Minor Isomer *syn*-[$Pd_2(1BD)_2(2AA)_2](BF_4)_4$

^{1}H NMR (600 MHz, CD_3CN) δ: 10.43 (d, *J* = 1.8 Hz, 2H, H_A), 10.25-10.21 (m, 2H, H_B), 9.84 (d, *J* = 2.2 Hz, 2H, H_I), 9.50 (ddd, *J* = 5.9, 1.3, 0.6 Hz, 2H, $H_{b'}$), 9.47-9.45 (m, 2H, $H_{a'}$), 9.33 (d, *J* = 1.8 Hz, 2H, H_a), 9.19 (d, *J* = 5.7 Hz, 2H, H_b), 8.63 (m, 2H, H_F), 8.37 (ddd, *J* = 8.6, 7.1, 1.4 Hz, 2H, H_C), 8.27 (ddd, *J* = 8.6, 6.5, 2.2 Hz, 2H, H_G), 8.23 (td, *J* = 1.7, 0.6 Hz, 2H, H_g), 8.05 (ddd, *J* = 8.0, 1.8, 1.3 Hz, 2H, H_d), 8.03-8.01 (m, 2H, H_E), 7.99 (m, 2H, $H_{d'}$), 7.85 (ddd, *J* = 8.1, 7.1, 1.0 Hz, 2H, H_D), 7.72 (ddd, *J* = 8.0, 5.9, 0.6 Hz, 2H, $H_{c'}$), 7.69-7.63 (m, 4H, $H_{e/e'}$, H_c), 7.62 (m, 2H, $H_{e/e'}$), 7.50 (m, 2H, H_f), 7.43 (d, *J* = 8.6 Hz, 2H, H_H).

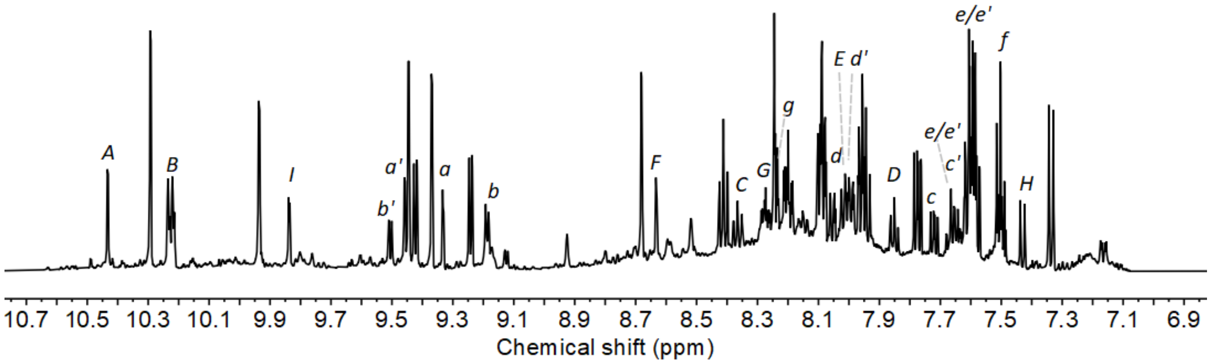

Figure S176 Partial ^{1}H NMR (600 MHz, CD_3CN) of [$Pd_2(1BD)_2(2AA)_2](BF_4)_4$ with peaks of minor *syn*-isomer labelled.

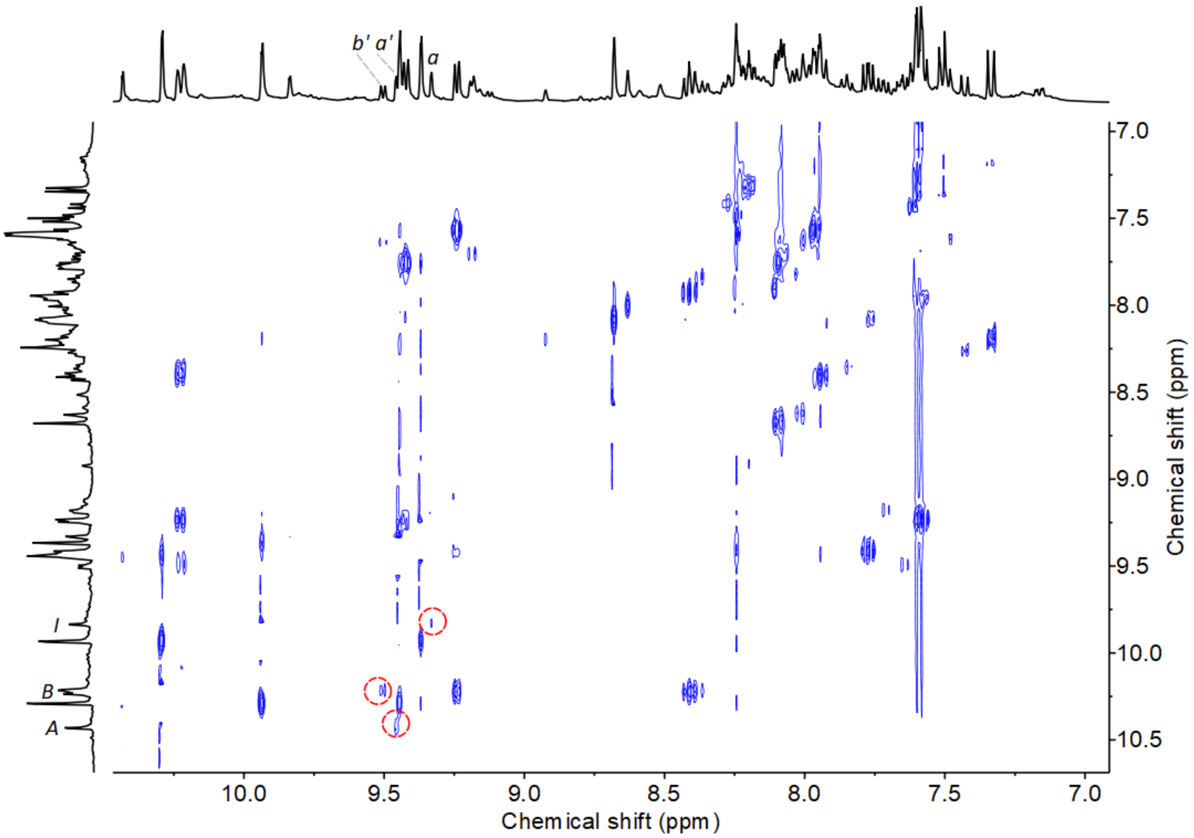

Figure S177 Partial NOESY (400 MHz, CD_3CN) of [$Pd_2(1BD)_2(2AA)_2](BF_4)_4$ with peaks assigned to minor *syn*-isomer.

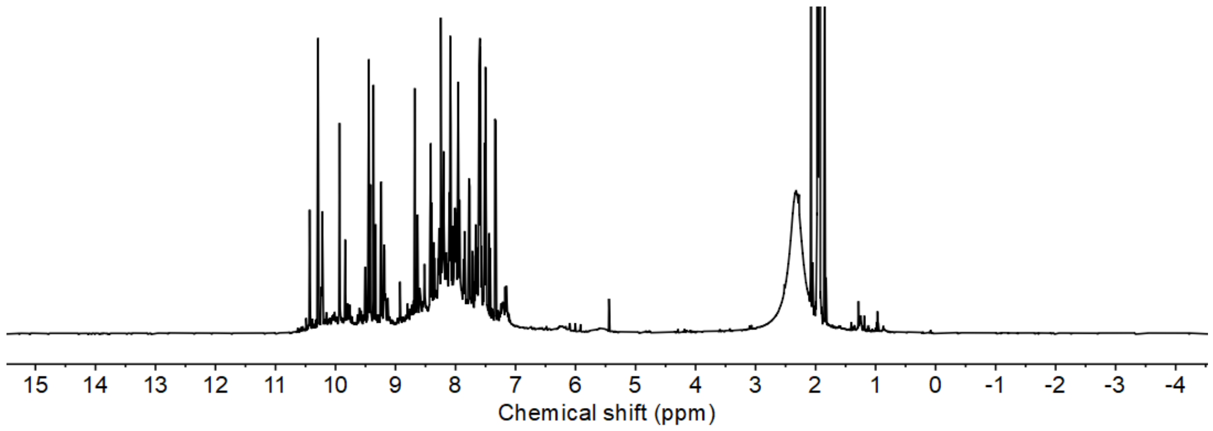

Figure S178 ^{1}H NMR (600 MHz, CD_3CN) of $[Pd_2(1BD)_2(2AA)_2](BF_4)_4$.

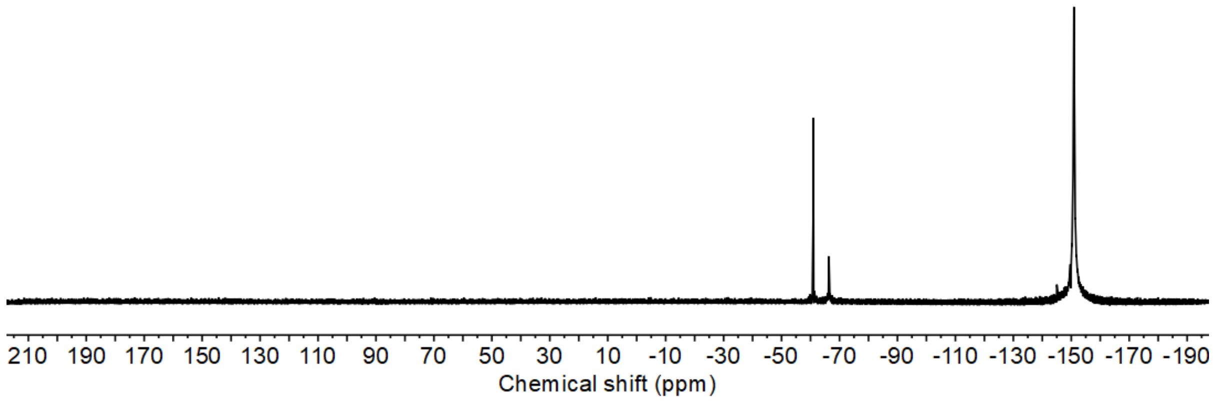

Figure S179 ^{19}F NMR (376 MHz, CD_3CN) of $[Pd_2(1BD)_2(2AA)_2](BF_4)_4$.

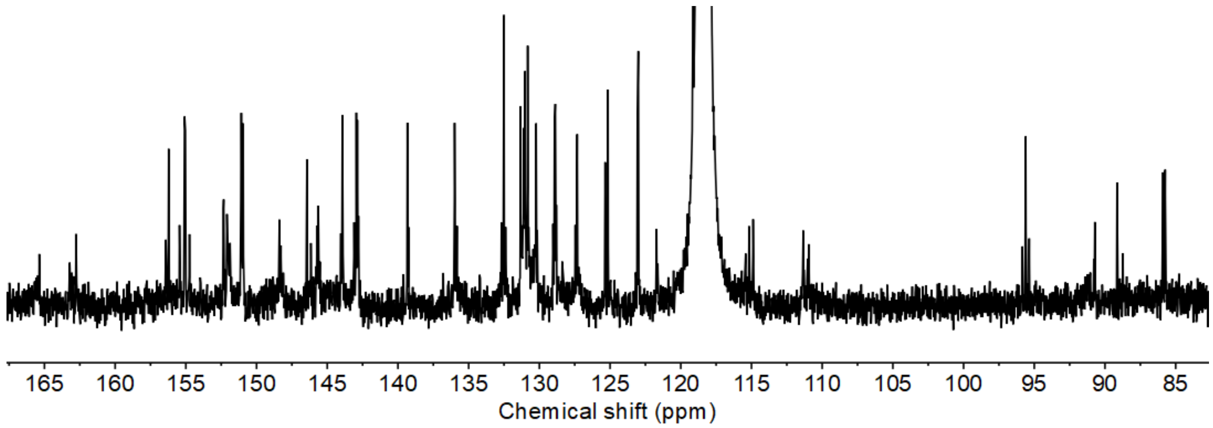

Figure S180 Partial ^{13}C NMR (101 MHz, CD_3CN) of $[Pd_2(1BD)_2(2AA)_2](BF_4)_4$.

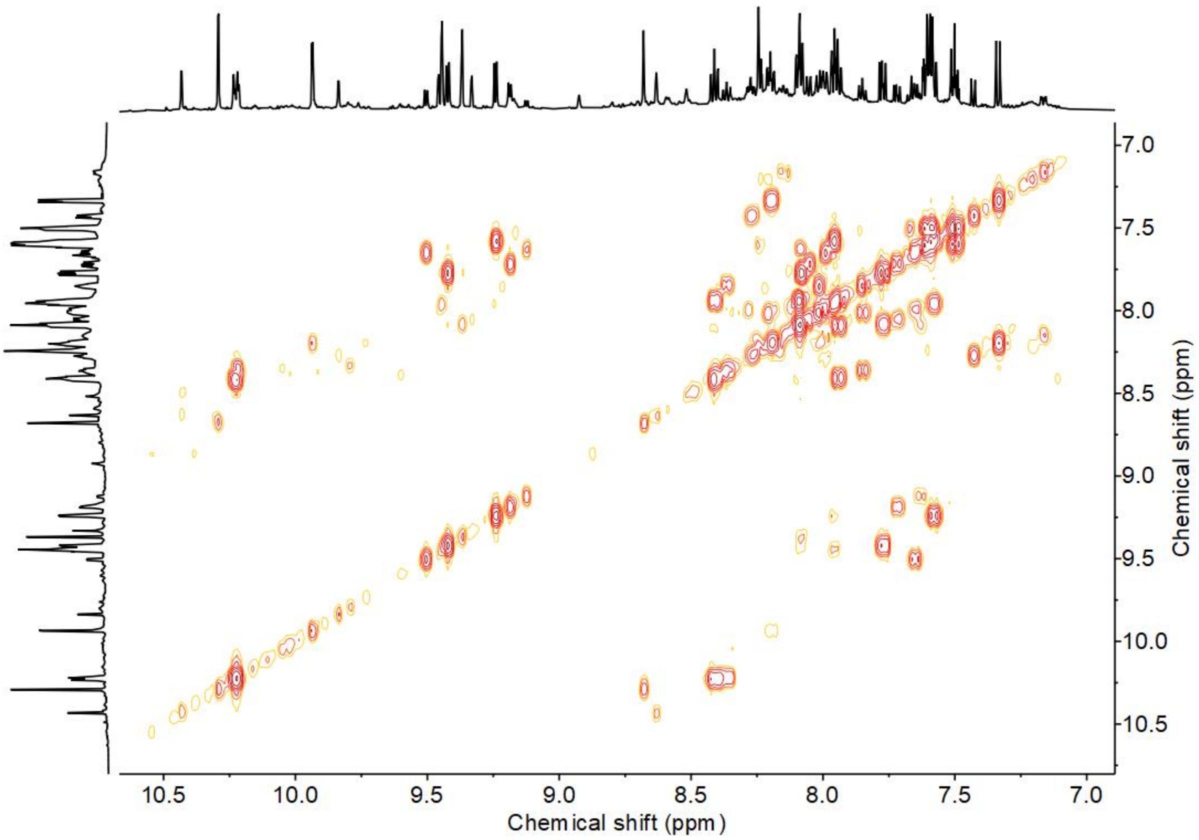

Figure S181 Partial COSY (CD_3CN) of $[Pd_2(1BD)_2(2AA)_2](BF_4)_4$.

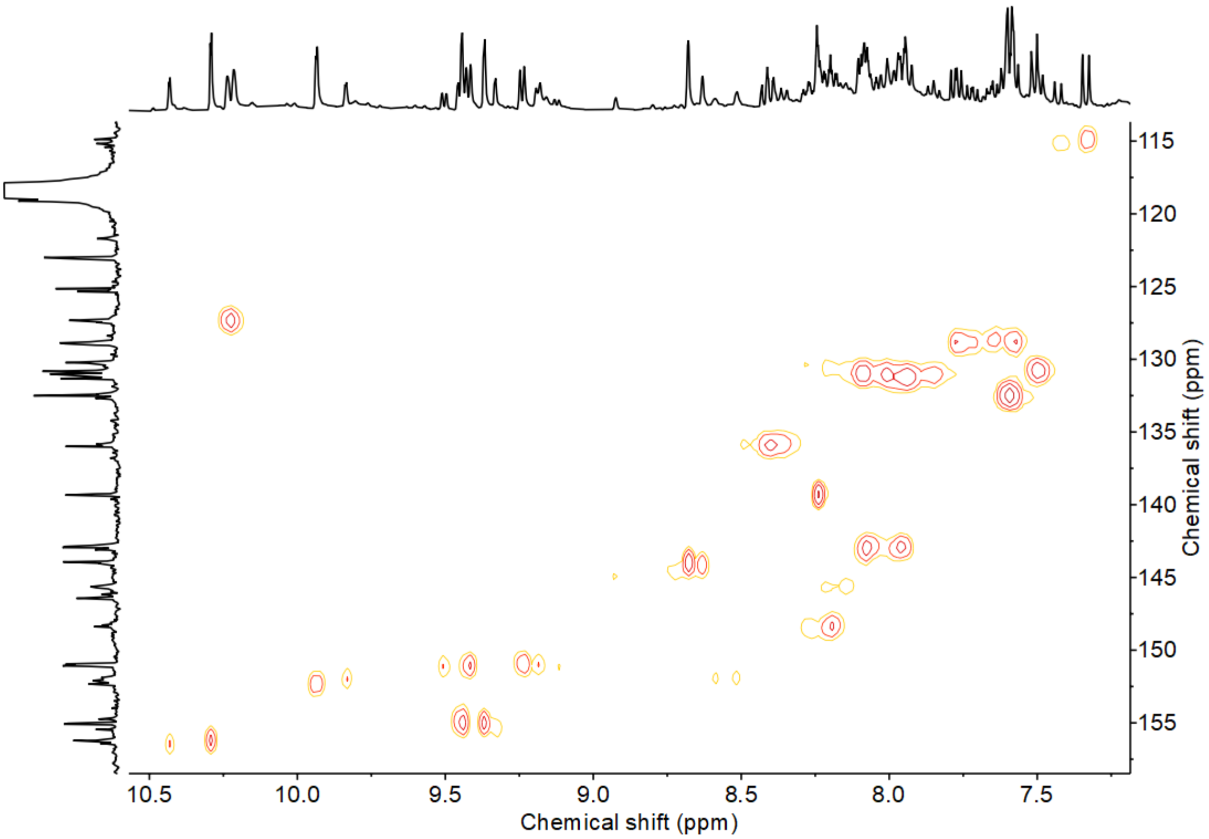

Figure S182 Partial HSQC (CD_3CN) of $[Pd_2(1BD)_2(2AA)_2](BF_4)_4$.

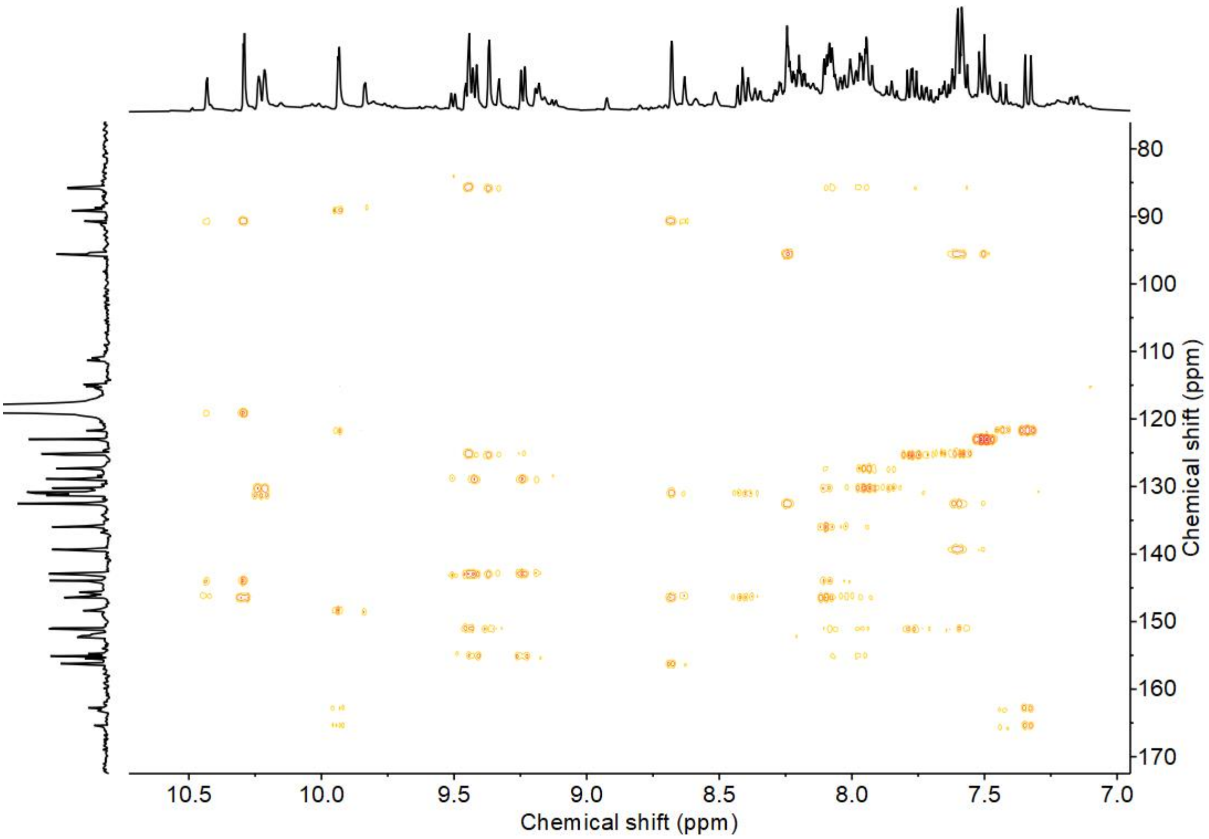

Figure S183 Partial HMBC (CD_3CN) of $[Pd_2(1BD)_2(2AA)_2](BF_4)_4$.

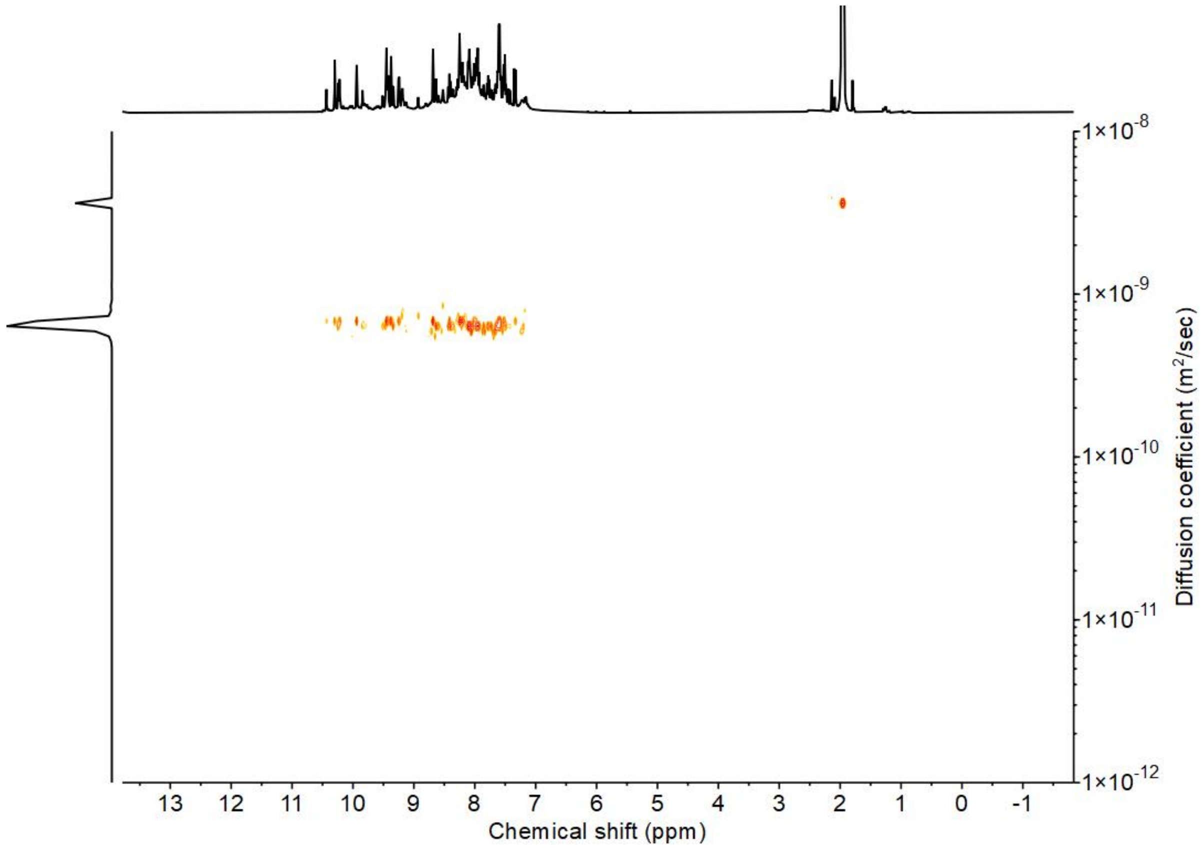

Figure S184 DOSY (CD_3CN) of $[Pd_2(1BD)_2(2AA)_2](BF_4)_4$.

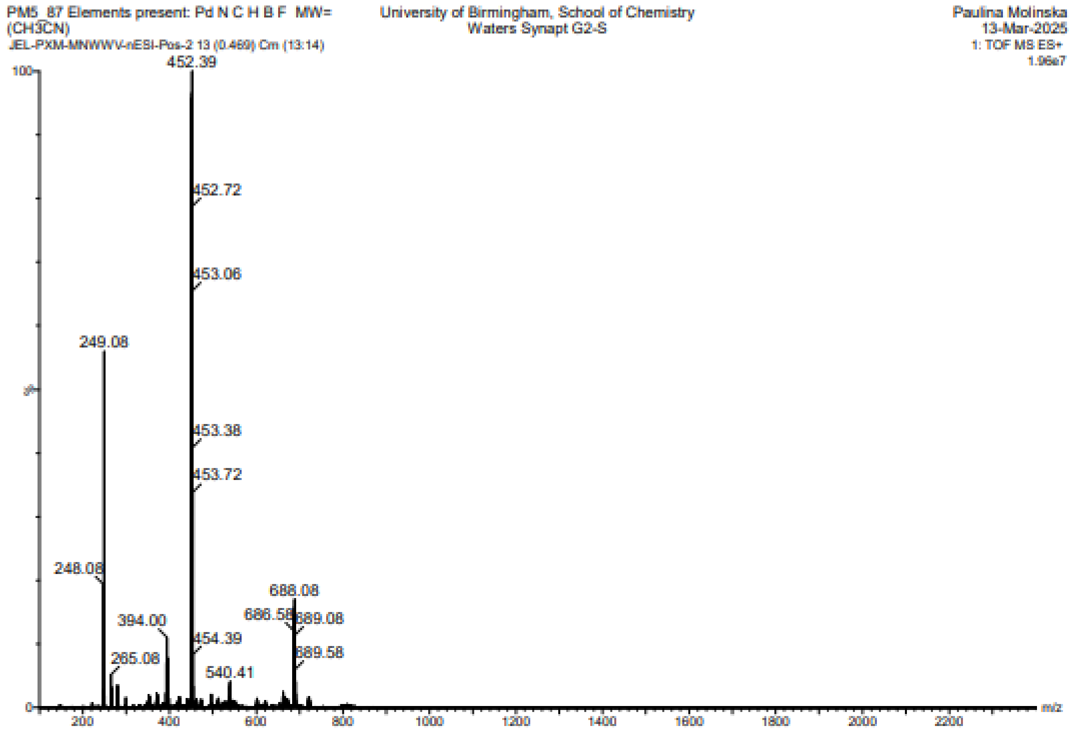

Figure S185 ESI-MS of $[Pd_2(1BD)_2(2AA)_2](BF_4)_4$.

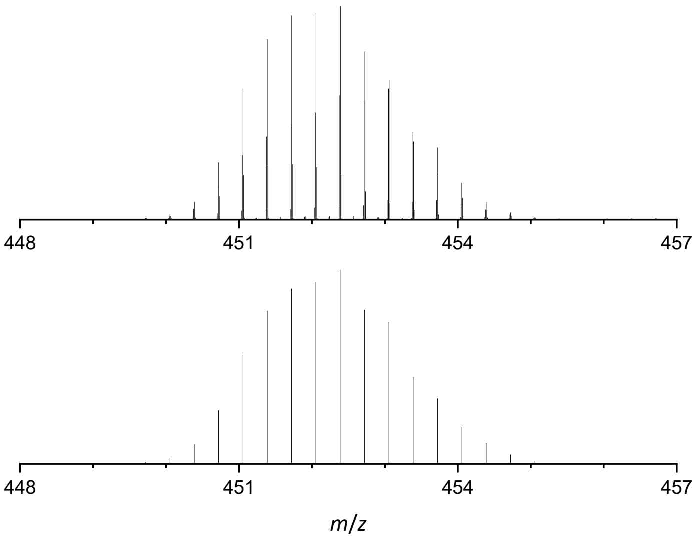

Figure S186 Observed (top) and calculated (bottom) isotopic patterns for $\{[Pd_2(1BD)_2(2AA)_2](BF_4)\}^{3+}$.

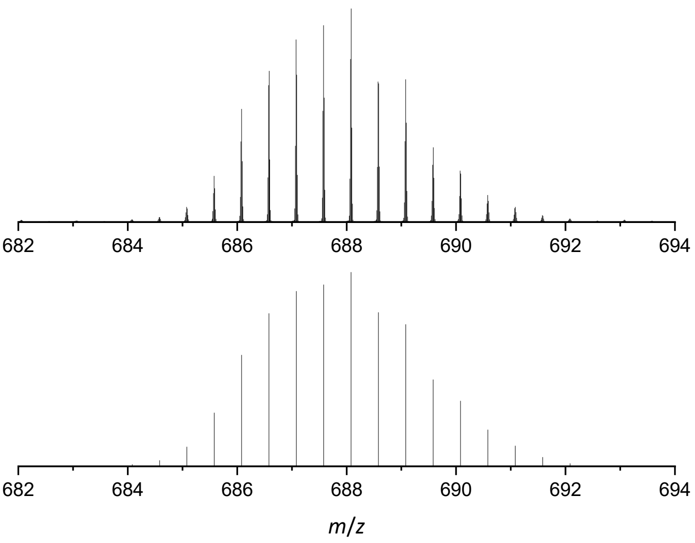

Figure S187 Observed (top) and calculated (bottom) isotopic patterns for $\{[Pd_2(1BD)_2(2AA)_2](BF_4)F\}^{2+}$.

Synthesis of $[Pd_2(1AE)_2(2AA)_2](BF_4)_4$

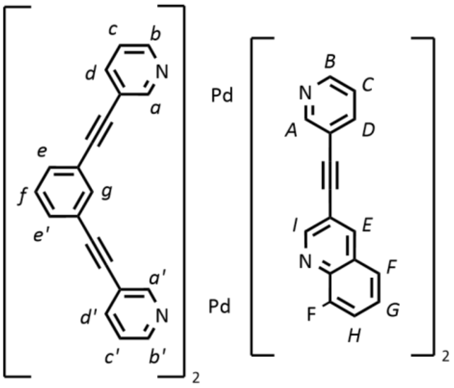

2AA (8.4 mg, 30 µmol, 1 eq.), **1AE** (7.4 mg, 30 µmol, 1 eq.) were combined in a vial to which a 40 mM stock solution of $[Pd(CH_3CN)_4](BF_4)_2$ (750 µL , 30 µmol, 1 eq.) in CD_3CN was added. A homogenous solution was obtained following sonication which was then transferred to a 5 mm NMR tube. The reaction was heated at 70 °C for 17 h.

ESI-MS *m/z* = 452.39 $\{[Pd_2(\mathbf{1AE})_2(\mathbf{2AA})_2](BF_4)\}^{3+}$ calc. 452.39.

^{19}**F NMR** (376 MHz, CD_3CN) δ: -122.37, -126.86, -151.22.

^{1}**H DOSY** (400 MHz, CD_3CN) D: 7.4 × 10^{-10} m^2s^{-1}; R_S: 8.3 Å.

Major Isomer *anti*-[$Pd_2(1AE)_2(2AA)_2](BF_4)_4$

^{1}H NMR (600 MHz, CD_3CN) δ: 10.53 (d, J = 1.7 Hz, 2H, H_I), 10.15 (d, J = 1.7 Hz, 2H, H_A), 9.46 (d, J = 1.7 Hz, 2H, $H_{a'}$), 9.38 (d, J = 1.7 Hz, 2H, H_a), 9.21 (ddd, J = 5.8, 1.4, 0.6 Hz, 2H, H_b), 8.97 (ddd, J = 5.8, 1.3, 0.6 Hz, 2H, $H_{b'}$), 8.87 (dd, J = 6.0, 1.2 Hz, 2H, H_B), 8.74-8.72 (m, 2H, H_E), 8.24 (app. td, J = 1.7, 0.6 Hz, 2H, H_g), 8.17-8.14 (m, 2H, H_H), 8.07 (m, 4H, H_D, H_d), 7.98-7.93 (m, 4H, $H_{d'}$, H_F), 7.91 (dd, J = 7.9, 4.7 Hz, 2H, H_G), 7.73 (ddd, J = 8.1, 5.9, 0.7 Hz, 2H, H_c), 7.60-7.56 (m, 6H, H_e, $H_{e'}$, $H_{c'}$), 7.55 (ddd, J = 7.9, 5.8, 0.7 Hz, 2H, H_C), 7.50 (app. tt, J = 7.7, 0.6 Hz, 2H, H_f).

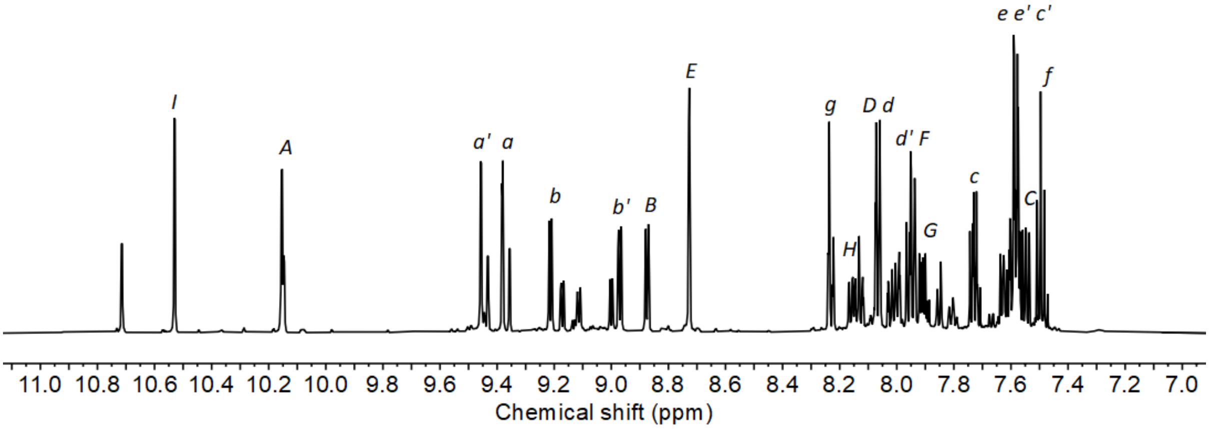

Figure S188 Partial ^{1}H NMR (600 MHz, CD_3CN) of [$Pd_2(1AE)_2(2AA)_2](BF_4)_4$ with peaks of major *anti*-isomer labelled.

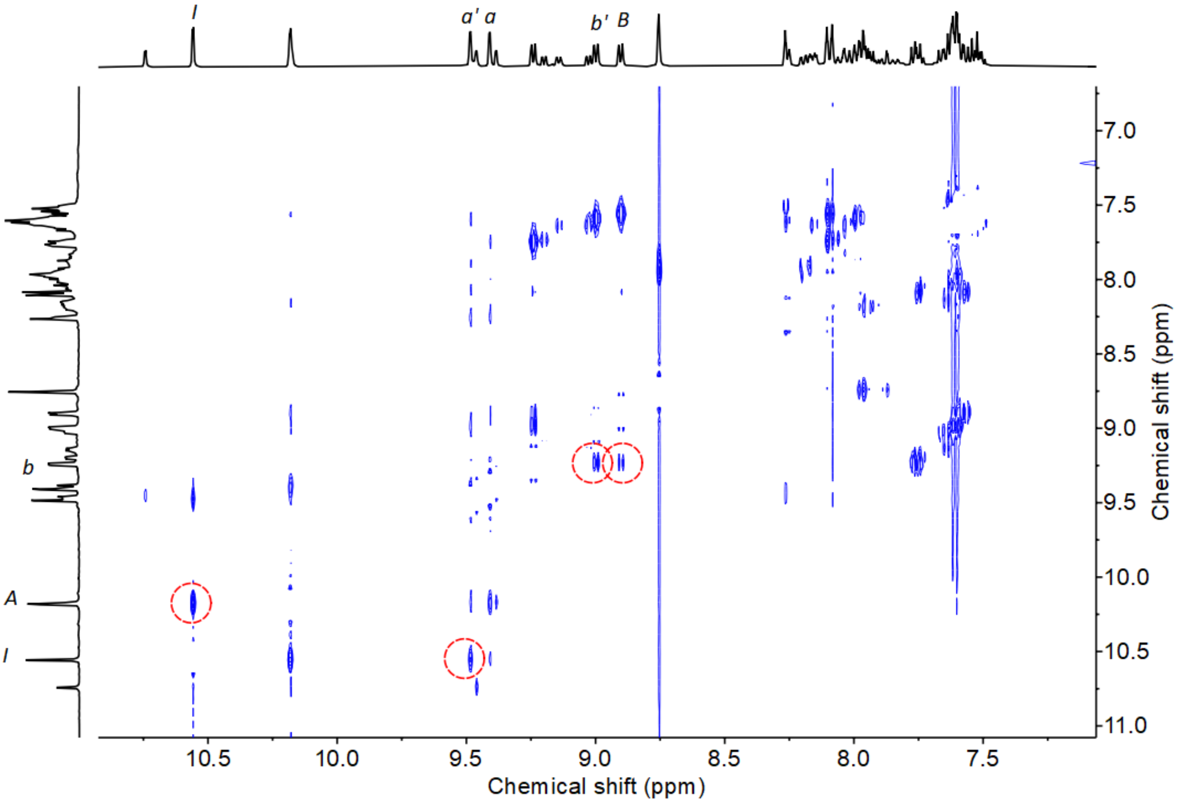

Figure S189 Partial NOESY (400 MHz, CD_3CN) of [$Pd_2(1AE)_2(2AA)_2](BF_4)_4$ with peaks assigned to major *anti*-isomer.

Minor Isomer *syn*-$[Pd_2(1AE)_2(2AA)_2](BF_4)_4$

^{1}H NMR (600 MHz, CD_3CN) δ: 10.71 (d, *J* = 1.8 Hz, 2H, H_I), 10.15 (d, *J* = 1.7 Hz, 2H, H_A), 9.43 (d, *J* = 1.6 Hz, 2H, H_a), 9.36 (dd, *J* = 1.6, 0.8 Hz, 2H, $H_{a'}$), 9.17 (ddd, *J* = 5.8, 1.4, 0.6 Hz, 2H, $H_{b'}$), 9.11 (d, *J* = 5.9 Hz, 2H, H_b), 9.00 (ddd, *J* = 5.9, 1.3, 0.5 Hz, 2H, H_B), 8.22 (app. td, *J* = 1.7, 0.6 Hz, 2H, H_I), 8.04 – 7.98 (m, 3H), 7.85 (ddd, *J* = 8.0, 1.4, 0.6 Hz, 1H), 7.82 – 7.78 (m, 1H), 7.64 – 7.60 (m, 3H).

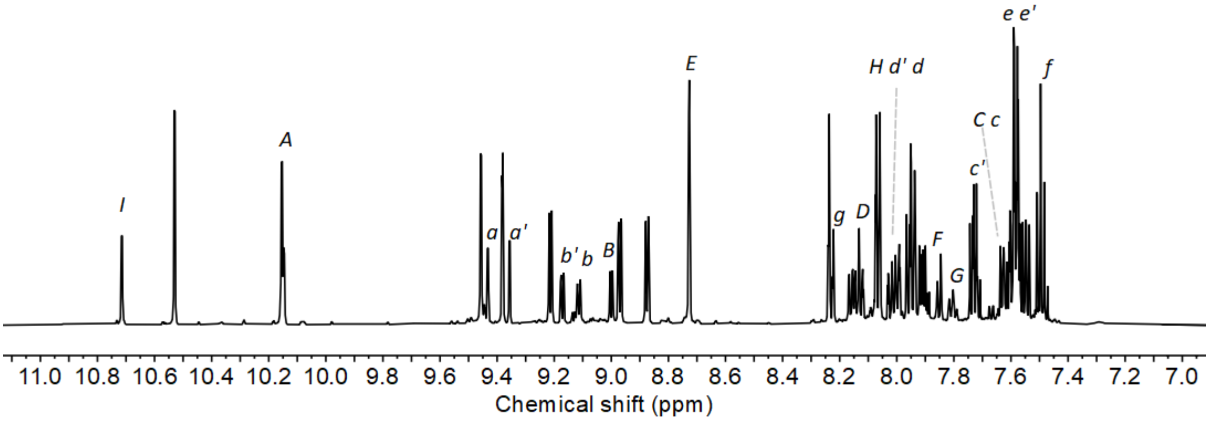

Figure S190 Partial ^{1}H NMR (600 MHz, CD_3CN) of $[Pd_2(1AE)_2(2AA)_2](BF_4)_4$ with peaks of minor *syn*-isomer labelled.

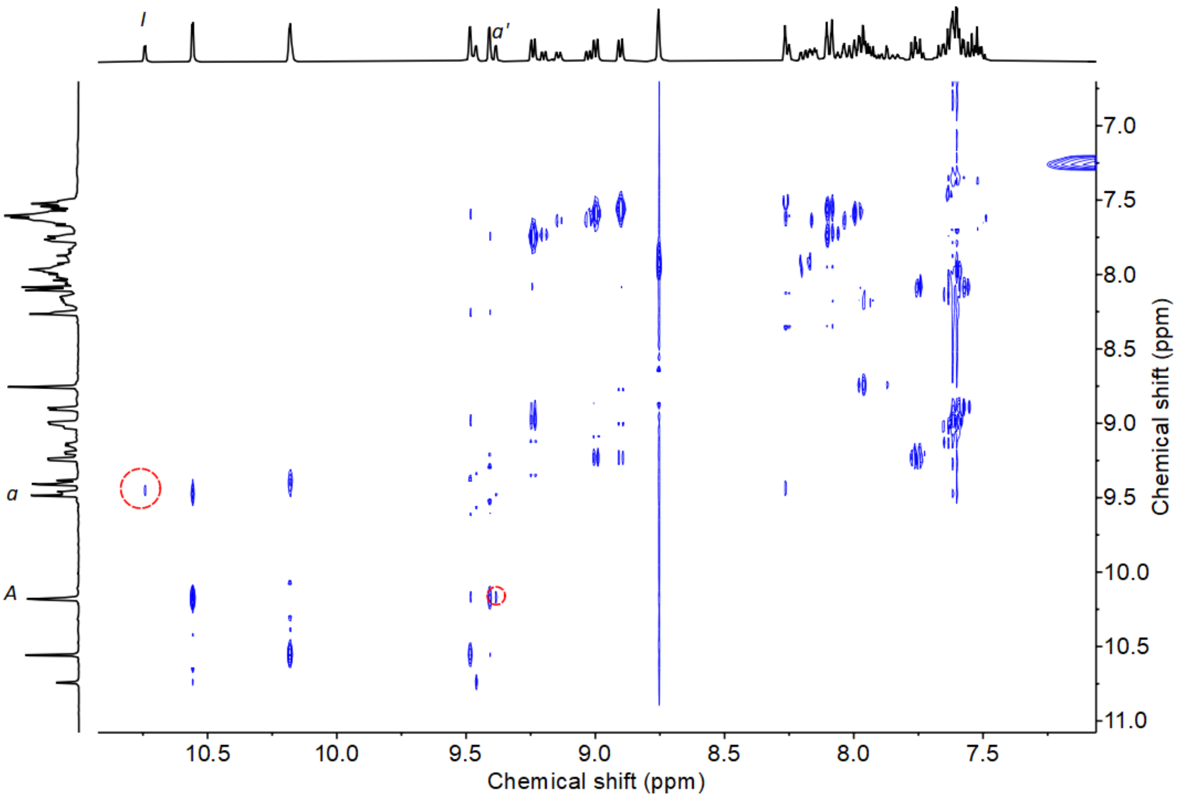

Figure S191 Partial NOESY (400 MHz, CD_3CN) of $[Pd_2(1AE)_2(2AA)_2](BF_4)_4$ with peaks assigned to minor *syn*-isomer.

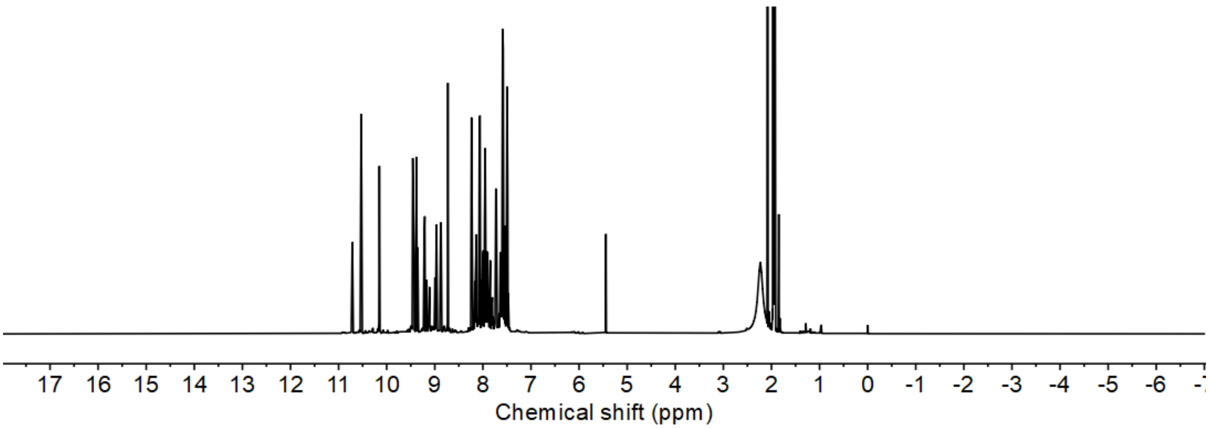

Figure S192 ^{1}H NMR (600 MHz, CD_3CN) of $[Pd_2(1AE)_2(2AA)_2](BF_4)_4$.

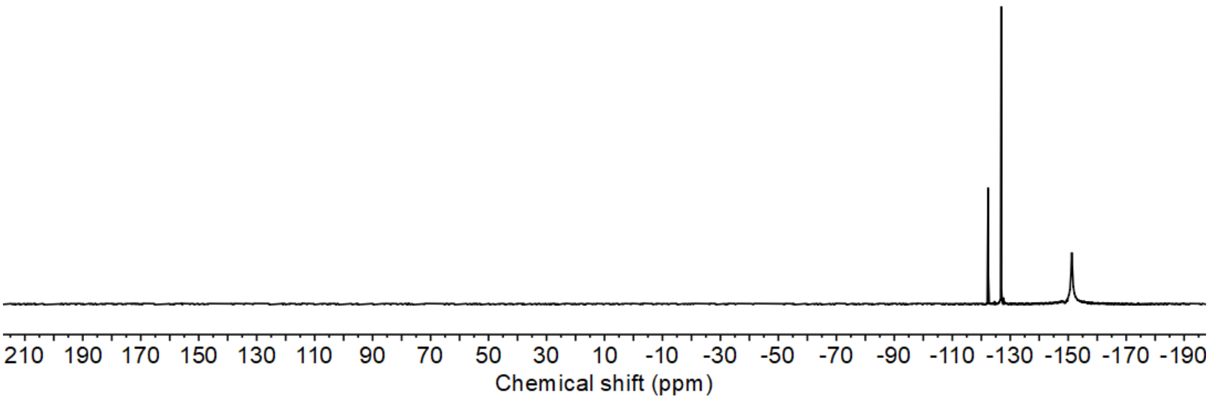

Figure S193 ^{19}F NMR (376 MHz, CD_3CN) of $[Pd_2(1AE)_2(2AA)_2](BF_4)_4$.

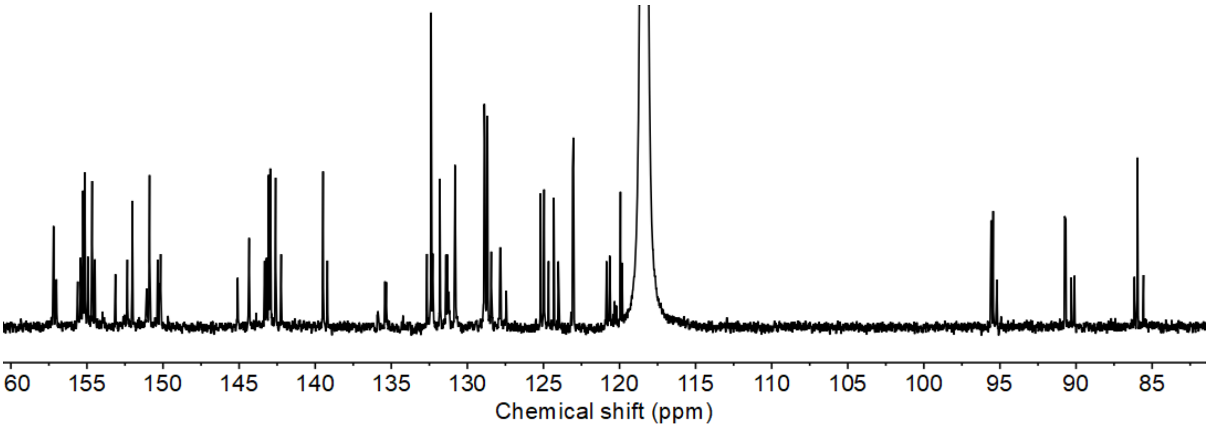

Figure S194 Partial ^{13}C NMR (101 MHz, CD_3CN) of $[Pd_2(1AE)_2(2AA)_2](BF_4)_4$.

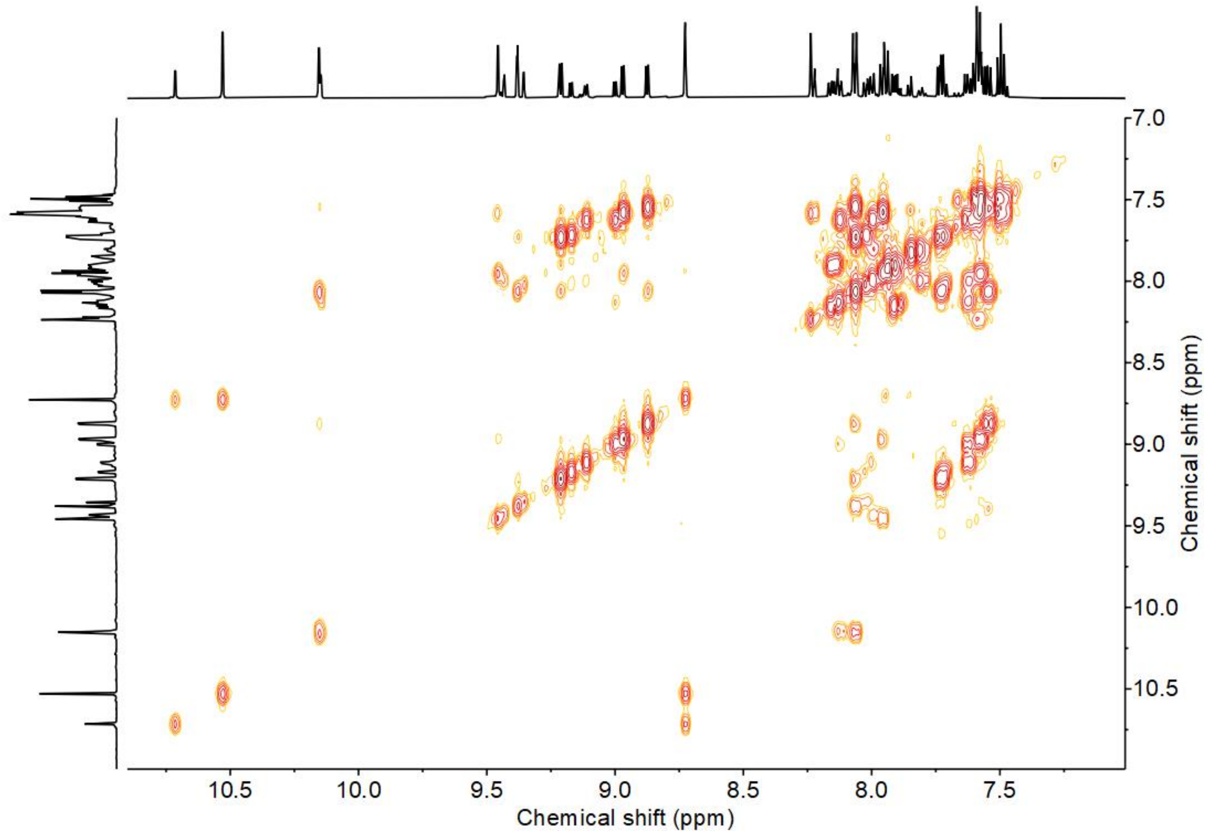

Figure S195 Partial COSY (CD_3CN) of $[Pd_2(1AE)_2(2AA)_2](BF_4)_4$.

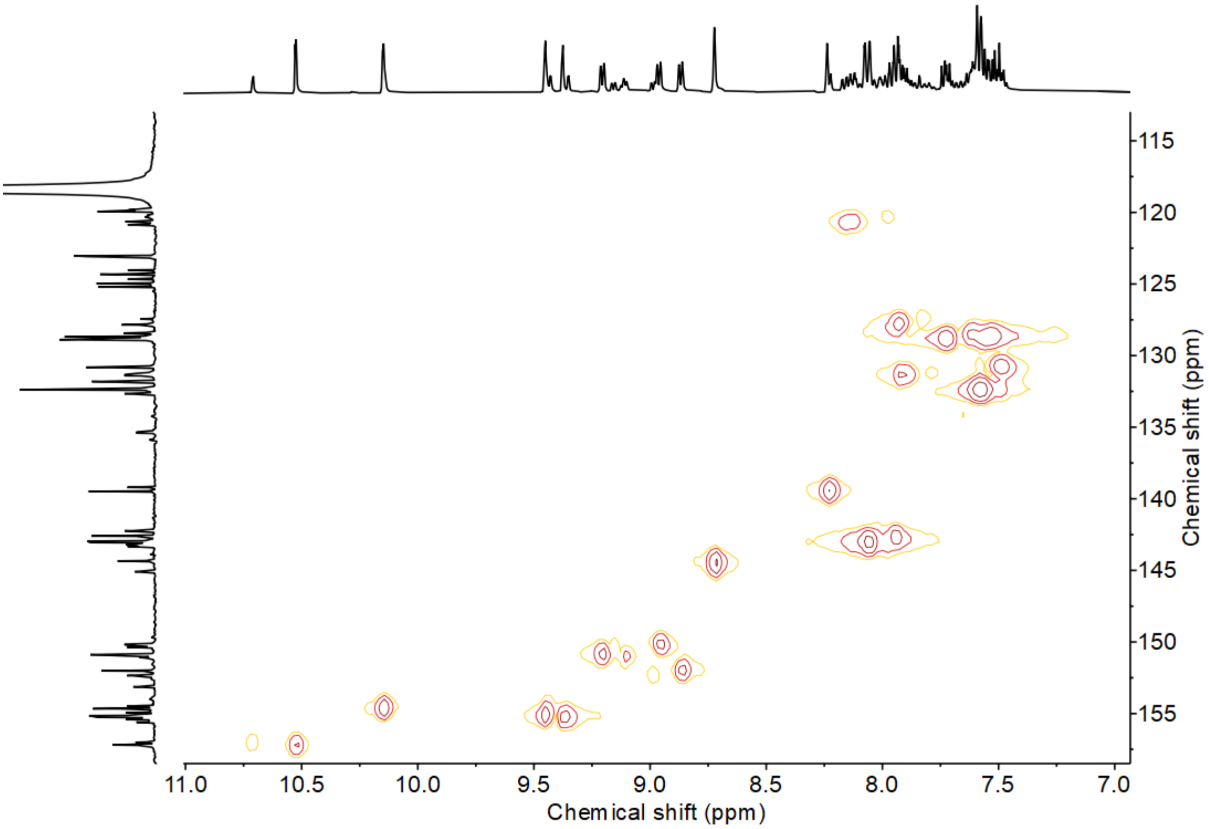

Figure S196 Partial HSQC (CD_3CN) of $[Pd_2(1AE)_2(2AA)_2](BF_4)_4$.

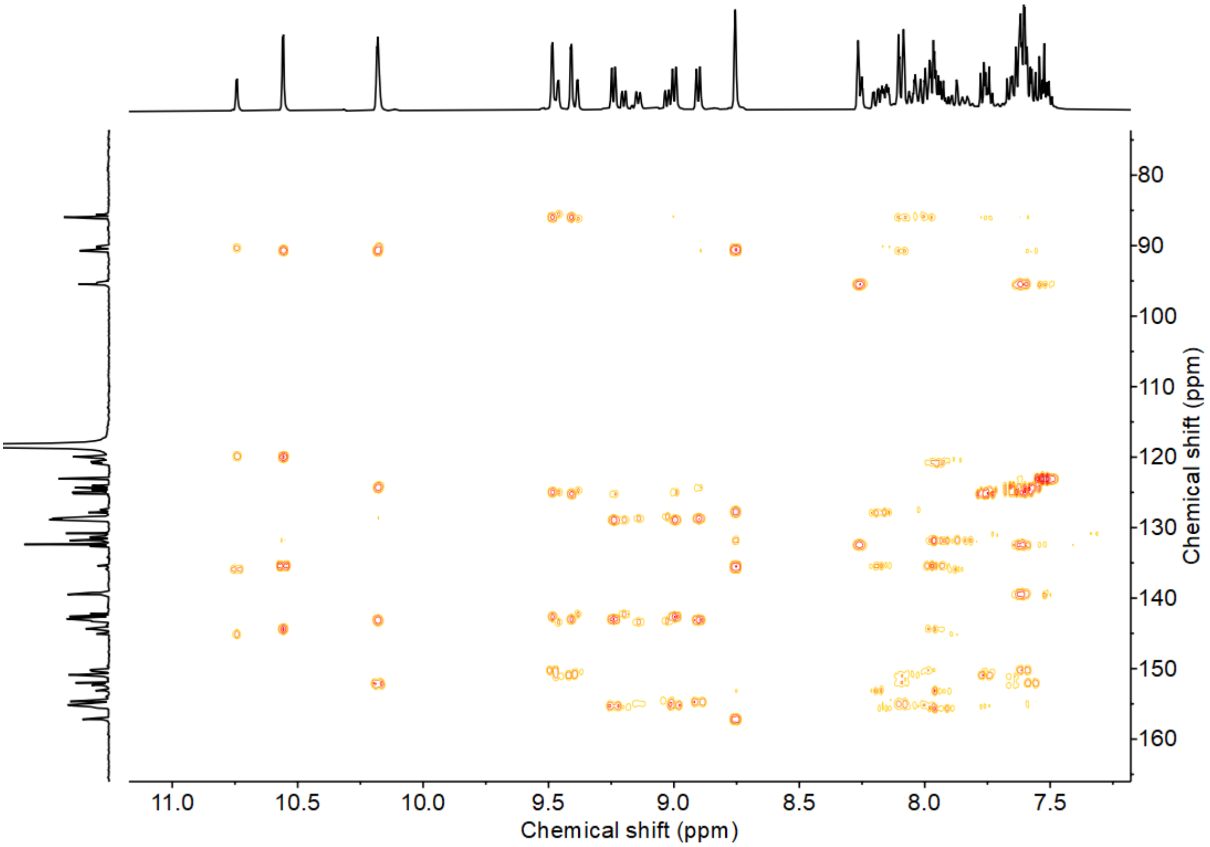

Figure S197 Partial HMBC (CD_3CN) of $[Pd_2(1AE)_2(2AA)_2](BF_4)_4$.

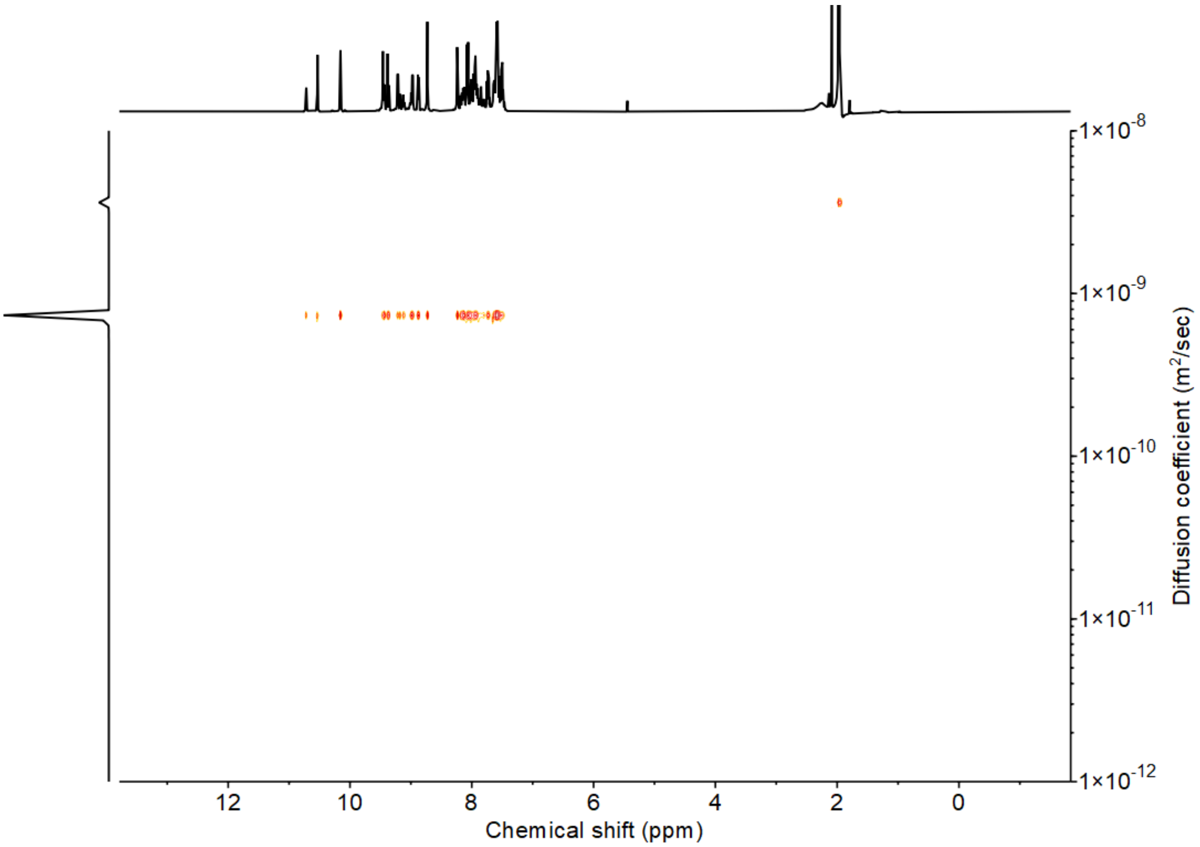

Figure S198 DOSY (CD_3CN) of $[Pd_2(1AE)_2(2AA)_2](BF_4)_4$.

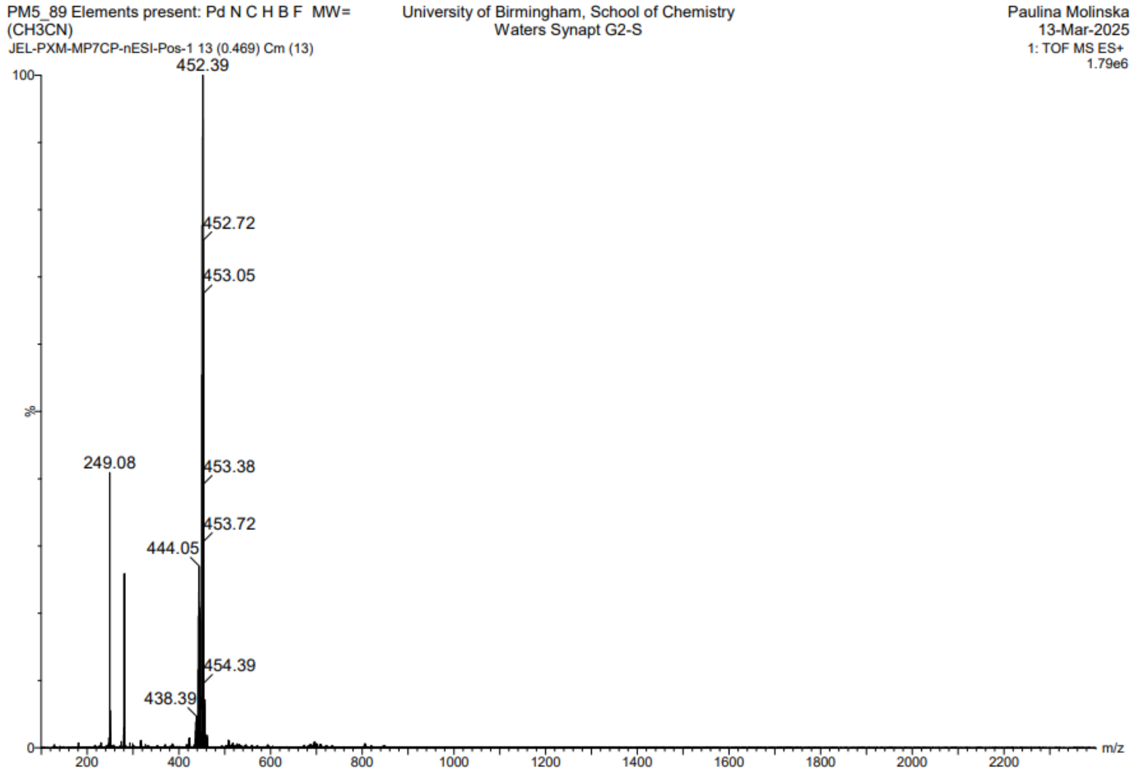

Figure S199 ESI-MS of $[Pd_2(1AE)_2(2AA)_2](BF_4)_4$.

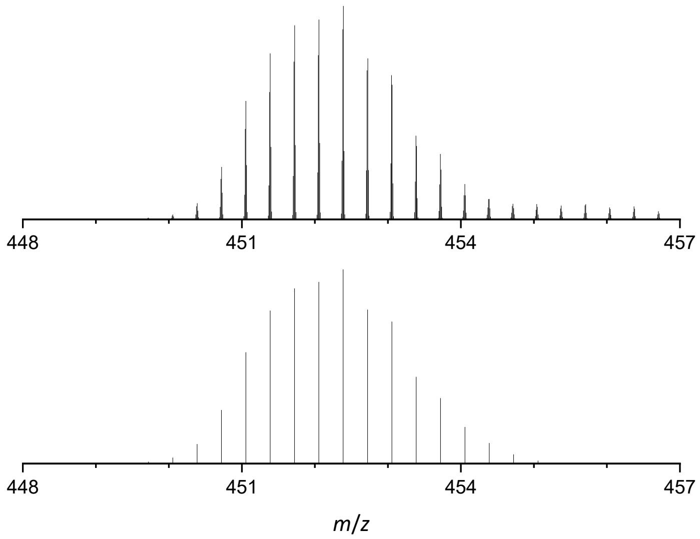

Figure S200 Observed (top) and calculated (bottom) isotopic patterns for $\{[Pd_2(1AE)_2(2AA)_2](BF_4)\}^{3+}$.

Synthesis of $[Pd_2(1CE)_2(2AA)_2](BF_4)_4$

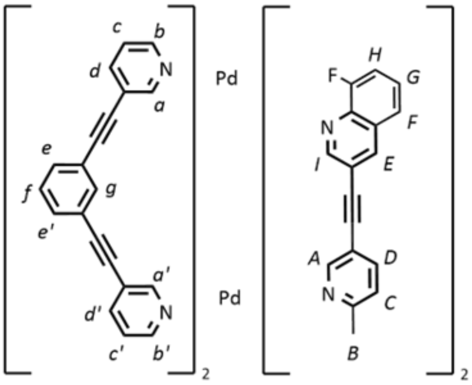

2AA (5.6 mg, 20 µmol, 1 eq.), **1CE** (5.2 mg, 20 µmol, 1 eq.) were combined in a vial to which a 27 mM stock solution of $[Pd(CH_3CN)_4](BF_4)_2$ (750 µL, 20 µmol, 1 eq.) in CD_3CN was added. A homogenous solution was obtained following sonication which was then transferred to a 5 mm NMR tube. The reaction was heated at 70 °C for 17 h.

ESI-MS *m/z* = 461.74 {[Pd$_2$(**1CE**)$_2$(**2AA**)$_2$](BF$_4$)}$^{3+}$ calc. 461.73, 702.09 {[Pd$_2$(**1CE**)$_2$(**2AA**)$_2$](BF$_4$)F}$^{2+}$ calc. 702.10, 736.10 {[Pd$_2$(**1CE**)$_2$(**2AA**)$_2$](BF$_4$)$_2$}$^{2+}$ calc. 736.10.

^{19}F NMR (376 MHz, CD_3CN) δ: -122.53, -151.58.

^{1}H DOSY (400 MHz, CD_3CN) *D*: 6.4×10^{-10} m^{2}s^{-1}; R_S: 9.6 Å.

Major Isomer *anti*-[$Pd_2(1CE)_2(2AA)_2](BF_4)_4$:

^{1}H NMR (400 MHz, CD_3CN) δ: 10.61 (s, 2H, H_I), 10.25 (s, 2H, H_A), 9.36 (s, 2H, $H_{a'}$), 9.32 (s, 2H, H_a), 9.22 (d, *J* = 5.9 Hz, 2H, H_b), 9.05 (d, *J* = 5.8 Hz, 2H, $H_{b'}$), 8.76 (s, 2H, H_E), 8.25 (s, 2H, H_g), 8.10-8.02 (m, 4H, H_G, H_d), 8.00-7.93 (m, 6H, H_D, H_F, $H_{d'}$), 7.89 (dd, *J* = 7.9, 4.8 Hz, 2H, H_H), 7.71 (dd, *J* = 7.8, 6.0 Hz, 2H, H_c), 7.64-7.57 (m, 6H, $H_{c'}$, H_e, $H_{e'}$), 7.49 (m, H_f), 7.42 (d, *J* = 8.2 Hz, 2H, H_C), 3.36 (s, 6H, H_B).

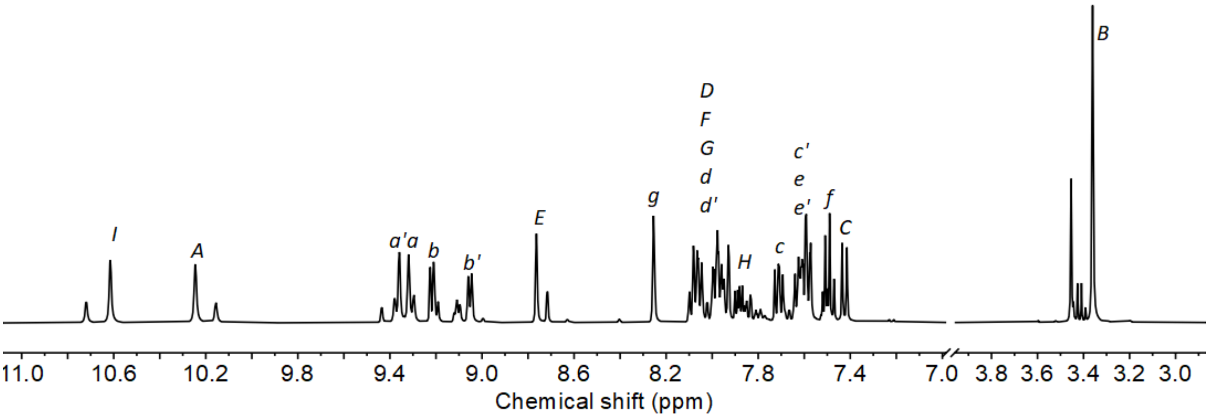

Figure S201 Partial ^{1}H NMR (400 MHz, CD_3CN) of [$Pd_2(1CE)_2(2AA)_2](BF_4)_4$ with peaks of major *anti*-isomer labelled.

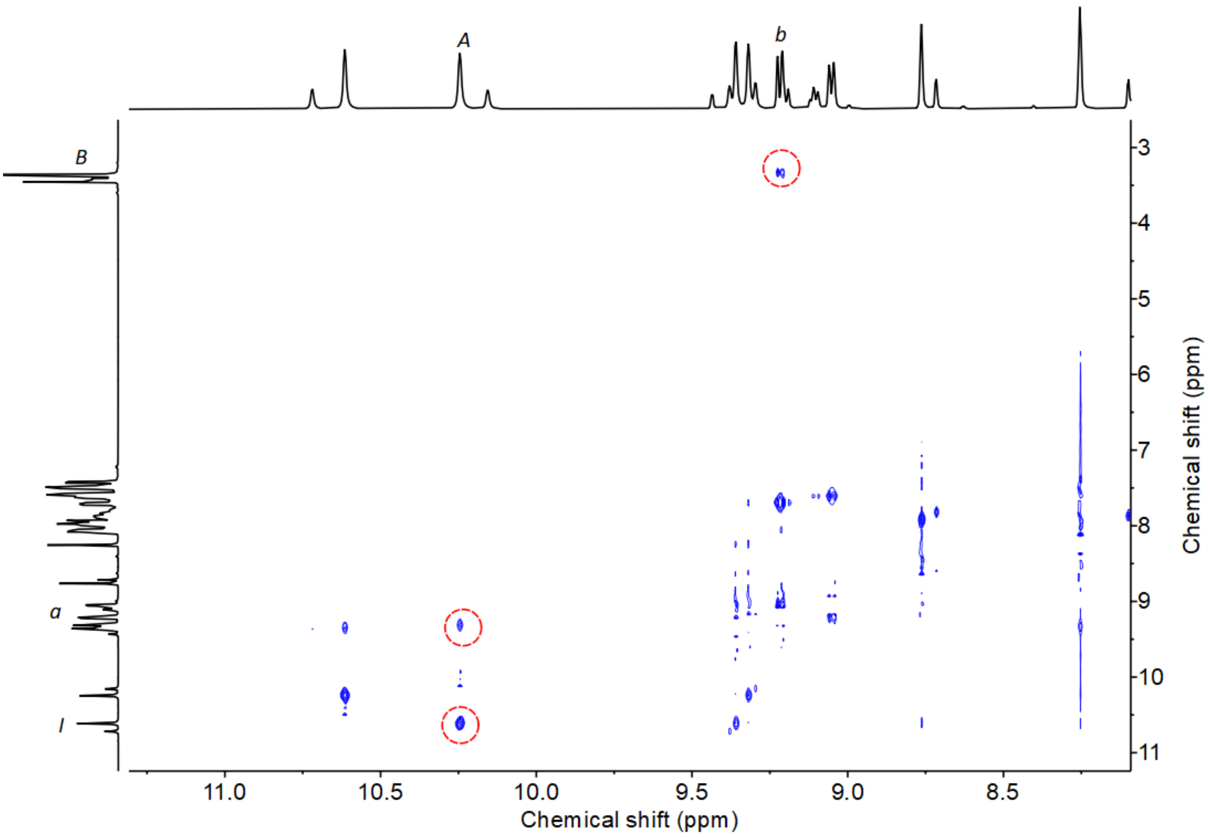

Figure S202 Partial NOESY (400 MHz, CD_3CN) of [$Pd_2(1CE)_2(2AA)_2](BF_4)_4$ labelled for major isomer with through-space interactions used to identify it as the *anti*-isomer.

Minor Isomer *syn*-$[Pd_2(1CE)_2(2AA)_2](BF_4)_4$:

^{1}H NMR (400 MHz, CD_3CN) δ: 10.72 (d, *J* = 1.8 Hz, 2H, H_I), 10.16 (s, 2H, H_A), 9.38 (s, 2H, H_a), 9.30 (d, *J* = 1.6 Hz, 2H, $H_{a'}$), 9.19 (d, *J* = 1.3 Hz, 2H, $H_{b'}$), 9.11 (m, 2H, H_b), 8.72 (d, *J* = 1.6 Hz, 2H, H_E), 8.25 (s, 2H, H_g), 8.10-8.02 (m, 4H, $H_{d'}$, H_D), 8.00-7.93 (m, 4H, H_d, H_G), 7.88-7.81 (m, 2H, H_H), 7.79 (ddd, *J* = 10.0, 4.9, 2.1 Hz, 2H, H_F), 7.72-7.64 (m, 2H, $H_{c'}$), 7.64-7.57 (m, 6H, H_c, H_e, $H_{e'}$), 7.54-7.45 (m, 4H, H_C, H_f), 3.45 (s, 6H, H_B).

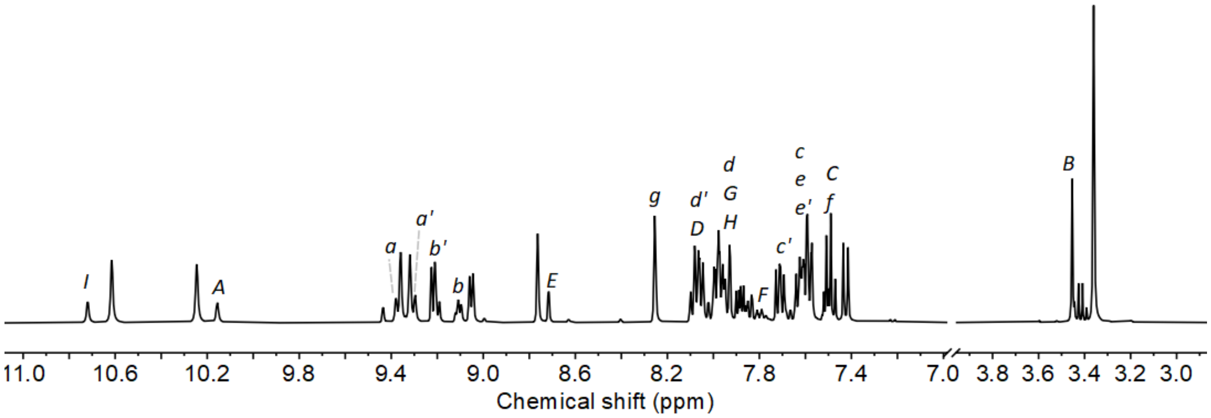

Figure S203 Partial ^{1}H NMR (400 MHz, CD_3CN) of $[Pd_2(1CE)_2(2AA)_2](BF_4)_4$ with peaks of minor *syn*-isomer labelled.

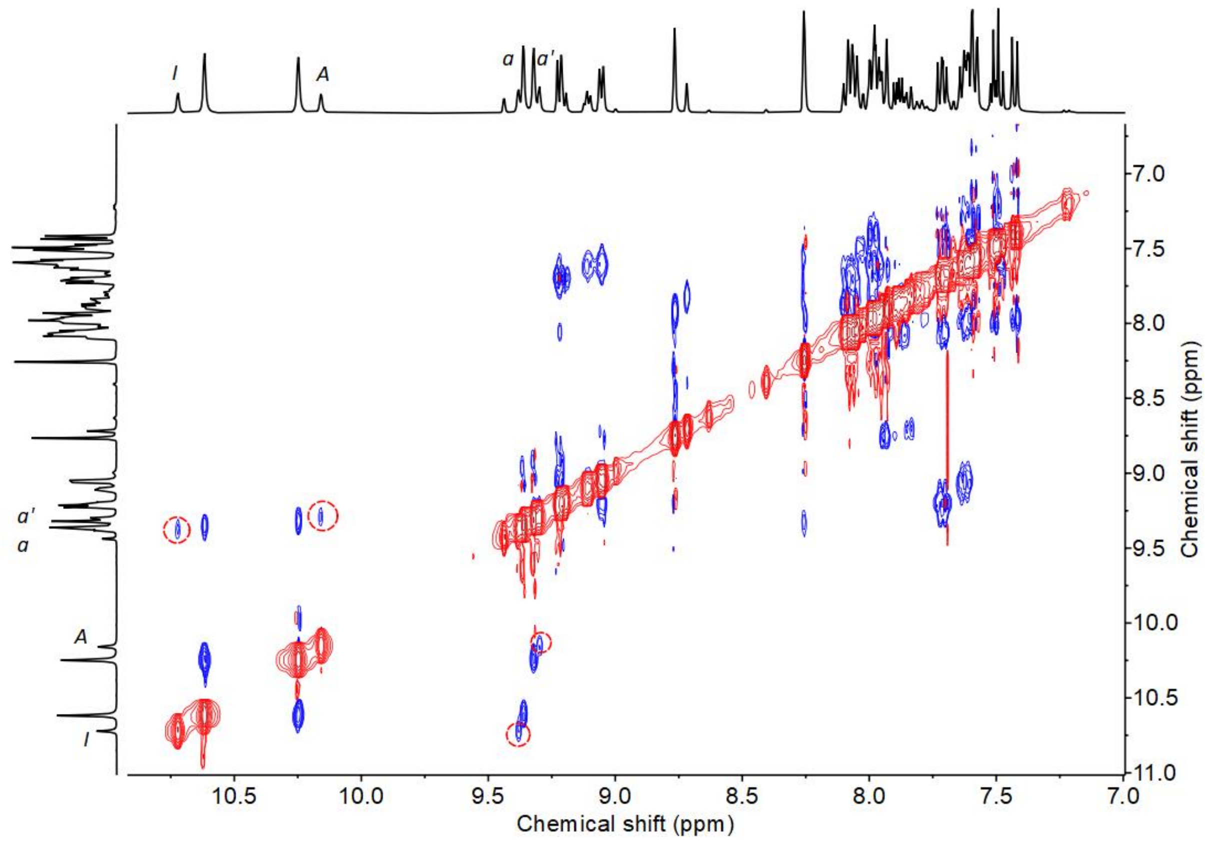

Figure S204 Partial NOESY (400 MHz, CD_3CN) of $[Pd_2(1CE)_2(2AA)_2](BF_4)_4$ labelled for minor isomer with through-space interactions used to identify it as the *syn*-isomer.

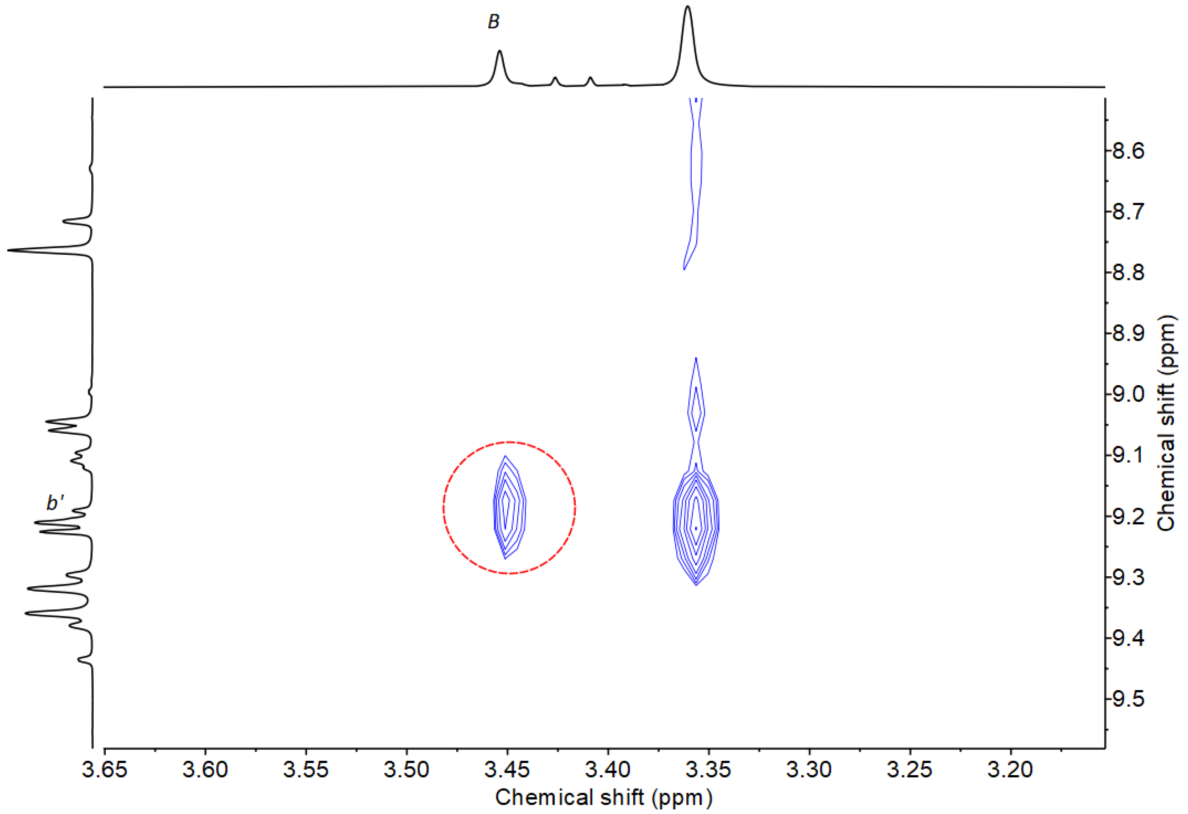

Figure S205 Partial NOESY (400 MHz, CD_3CN) of $[Pd_2(1CE)_2(2AA)_2](BF_4)_4$ labelled for minor isomer with through-space interactions used to identify it as the *syn*-isomer.

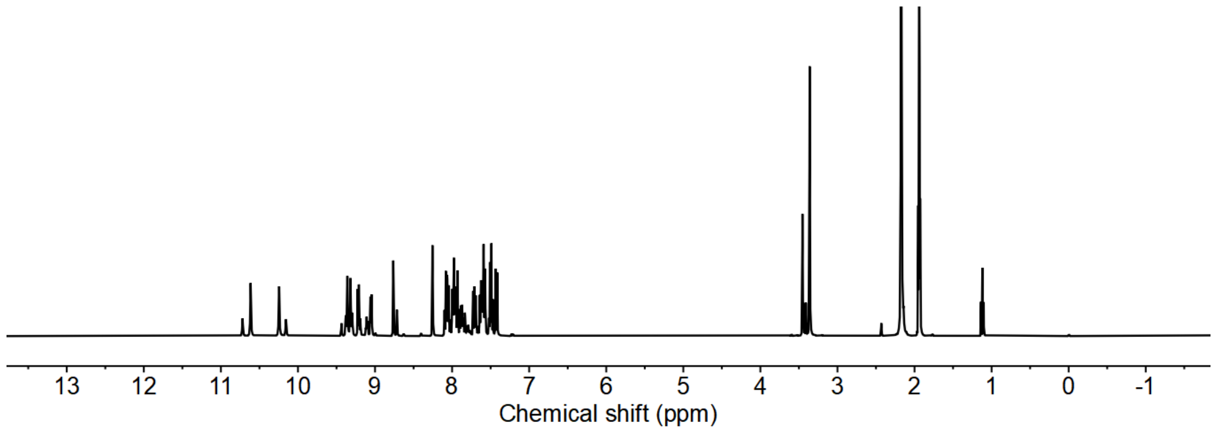

Figure S206 ^{1}H NMR (400 MHz, CD_3CN) of $[Pd_2(1CE)_2(2AA)_2](BF_4)_4$.

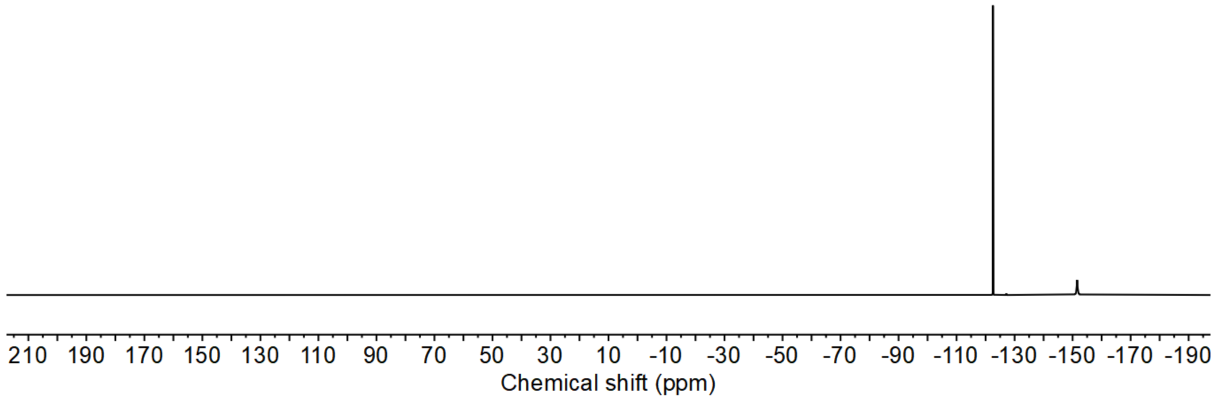

Figure 207 ^{19}F NMR (376 MHz, CD_3CN) of $[Pd_2(1CE)_2(2AA)_2](BF_4)_4$.

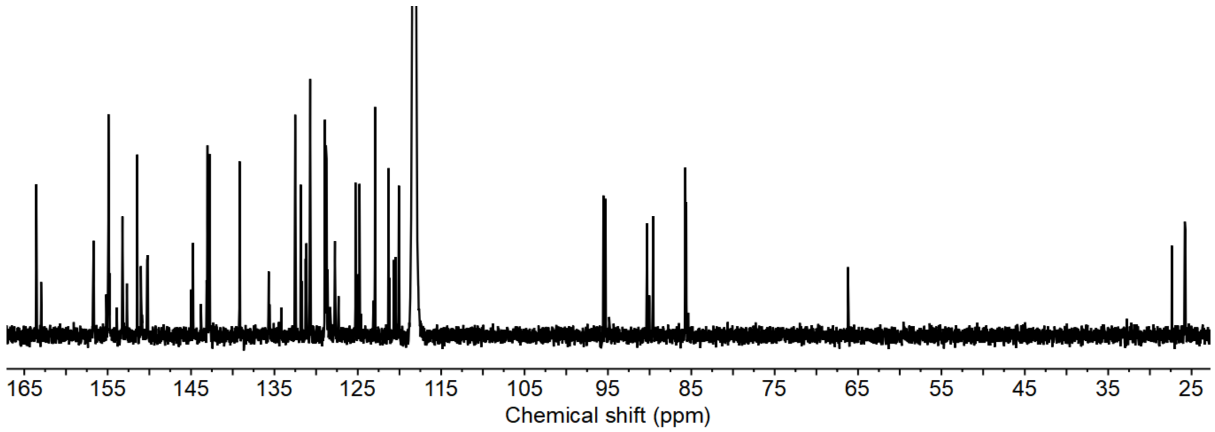

Figure S208 ^{13}C NMR (101 MHz, CD_3CN) of $[Pd_2(1CE)_2(2AA)_2](BF_4)_4$.

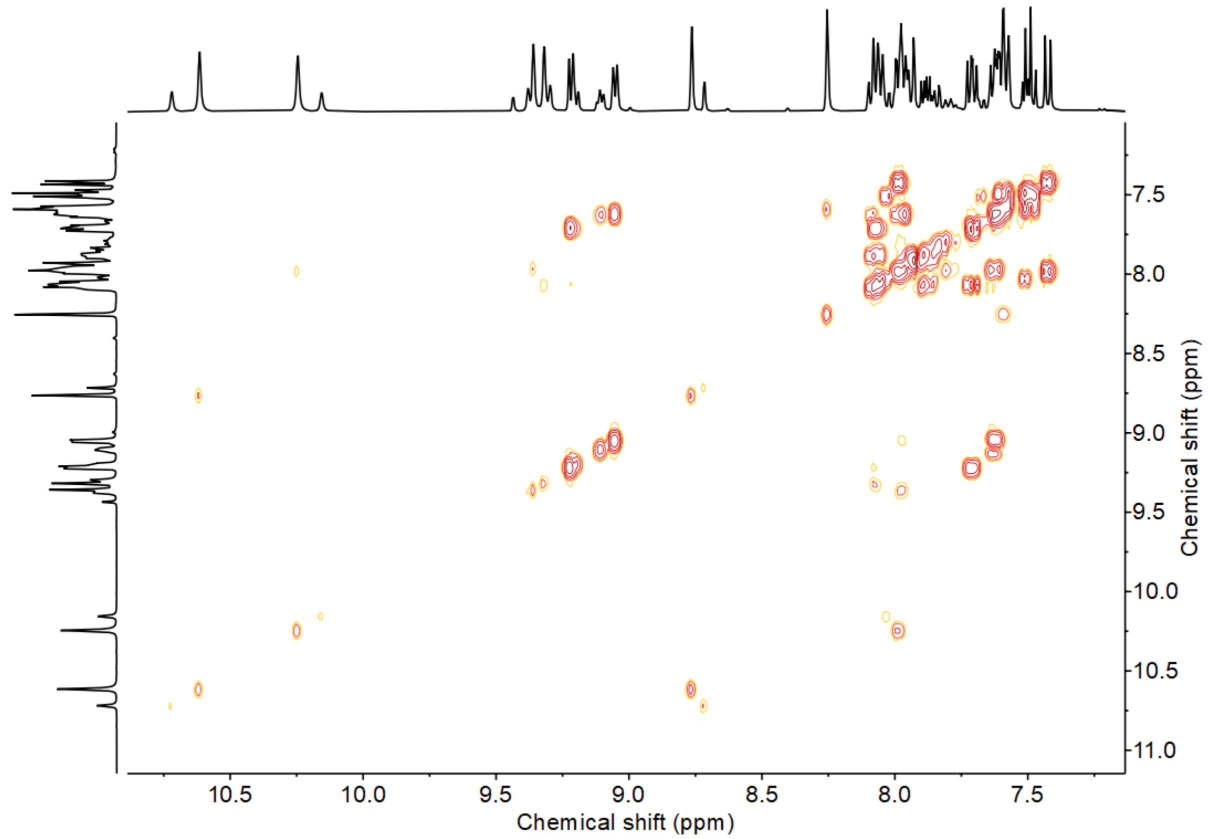

Figure S209 Partial COSY NMR (CD_3CN) of $[Pd_2(1CE)_2(2AA)_2](BF_4)_4$.

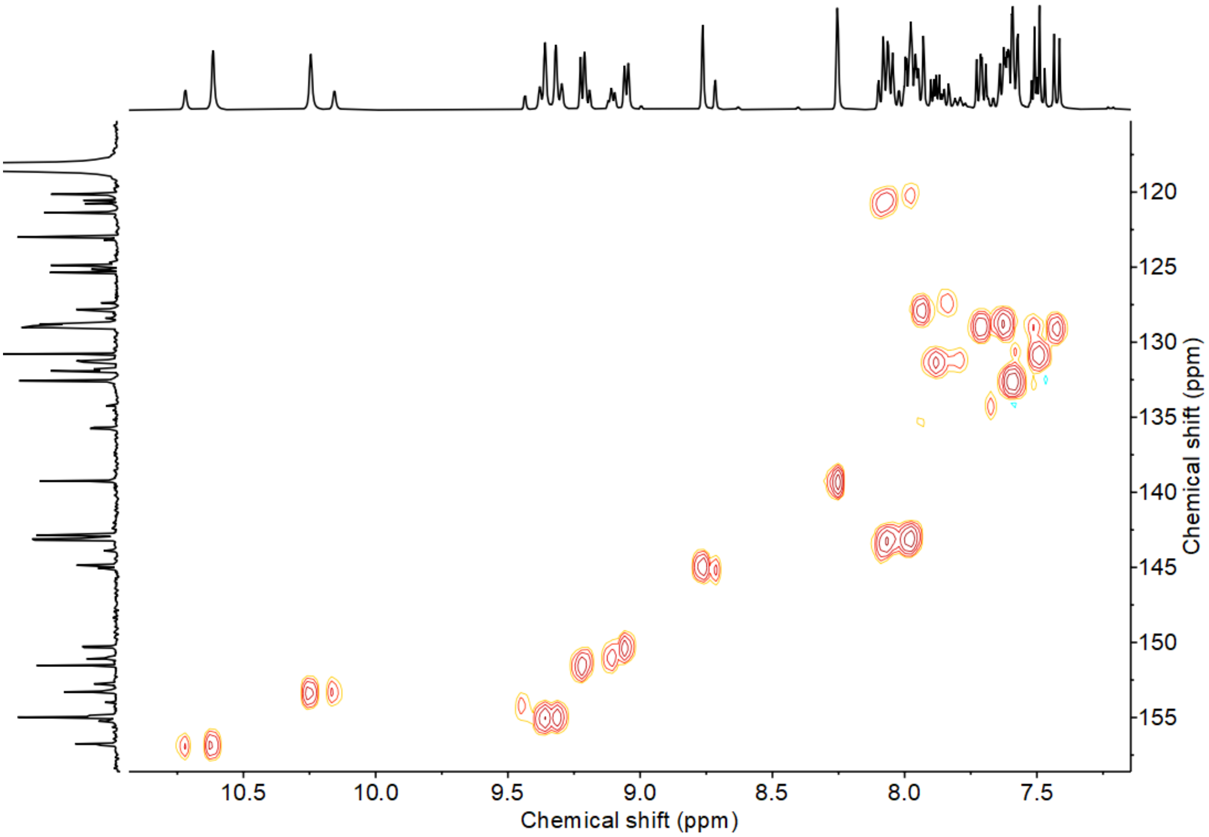

Figure S210 Partial HSQC NMR (CD_3CN) of $[Pd_2(1CE)_2(2AA)_2](BF_4)_4$.

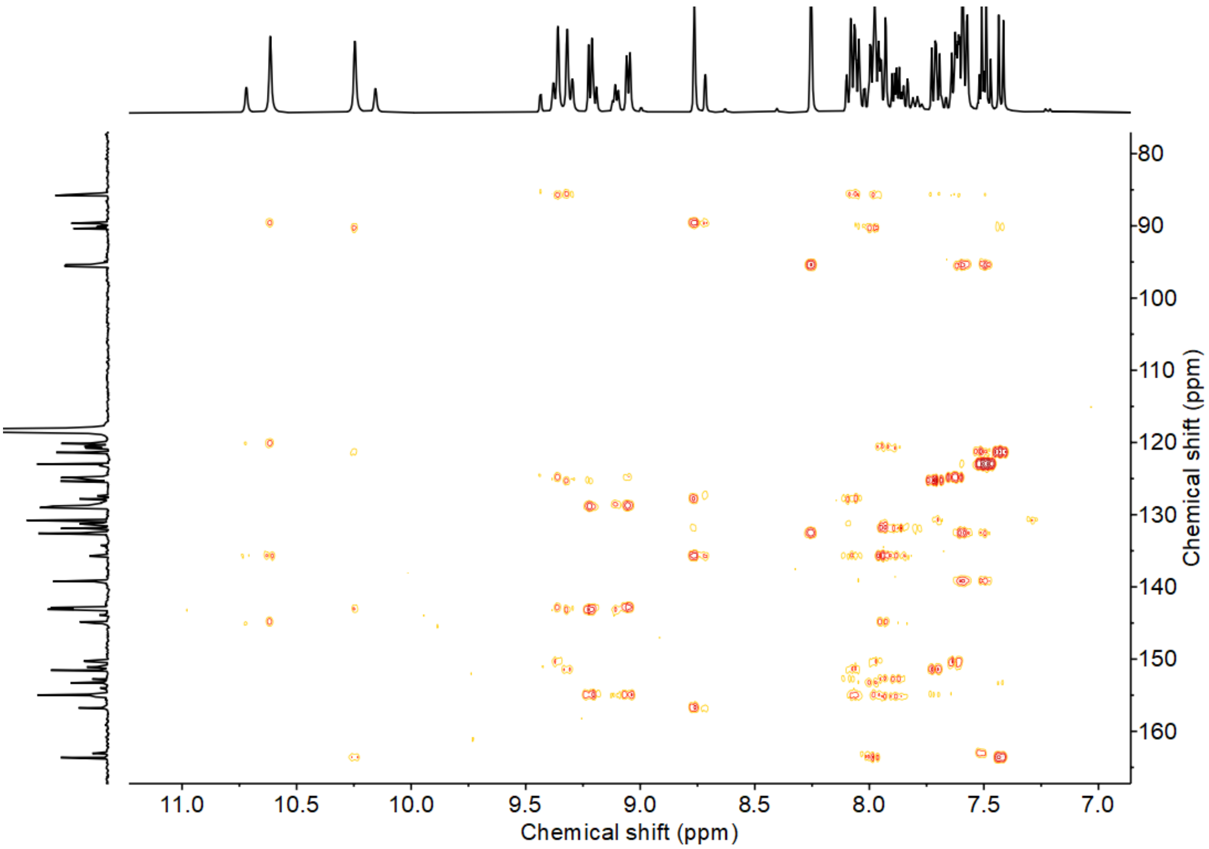

Figure S211 Partial HMBC NMR (CD_3CN) of [$Pd_2(1CE)_2(2AA)_2$](BF_4)$_4$.

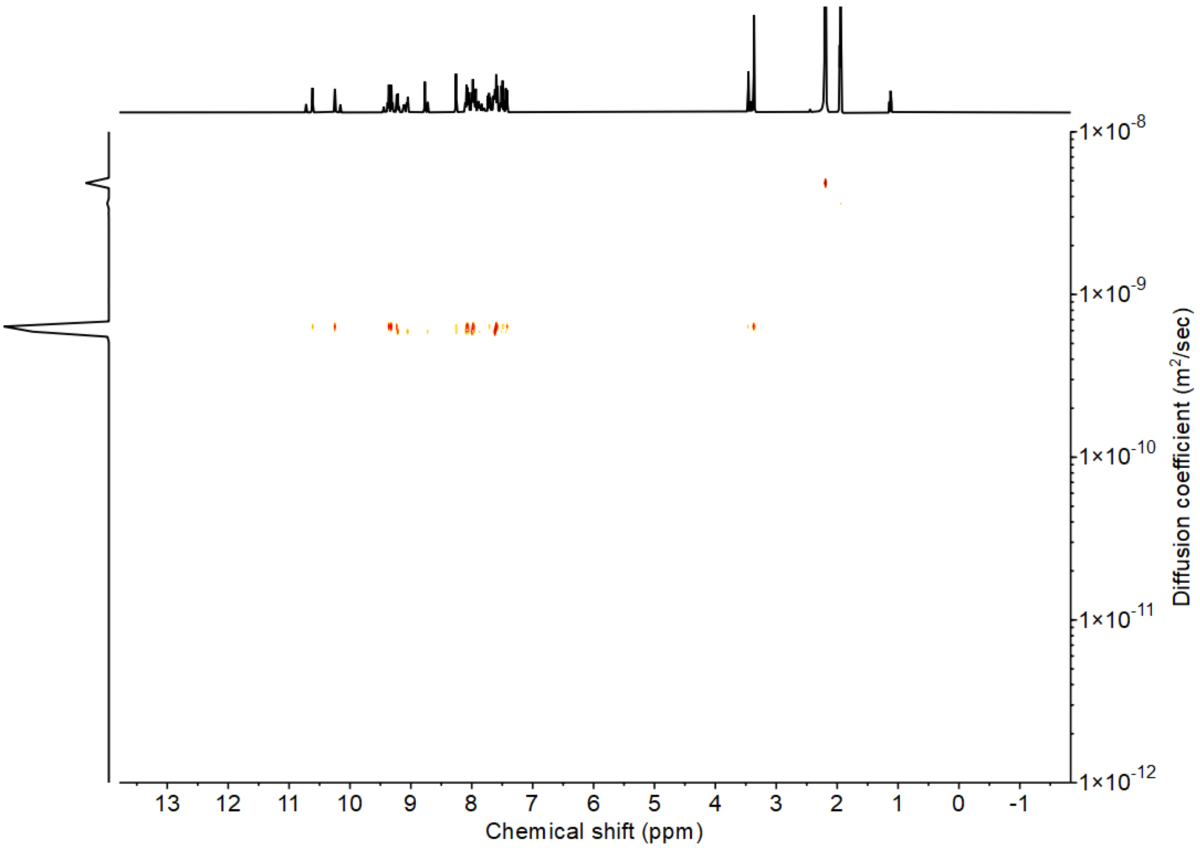

Figure S212 DOSY (CD_3CN) of [$Pd_2(1CE)_2(2AA)_2$](BF_4)$_4$.

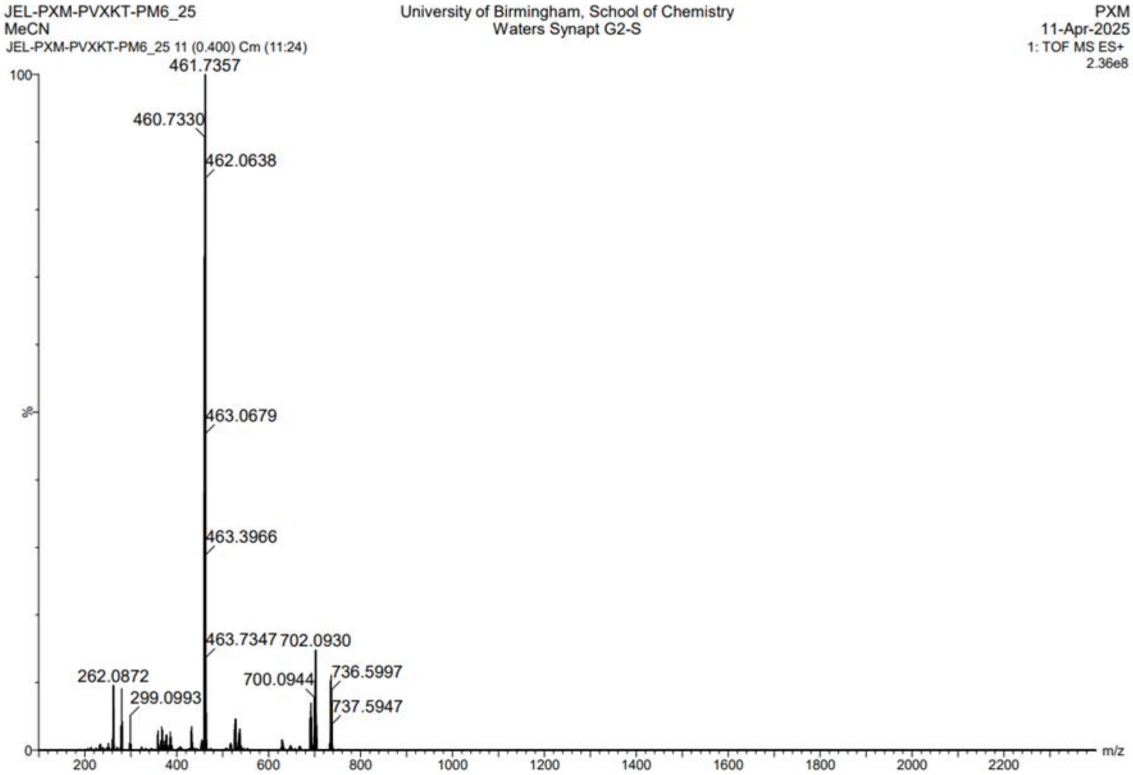

Figure S213 ESI-MS of $[Pd_2(1CE)_2(2AA)_2](BF_4)_4$.

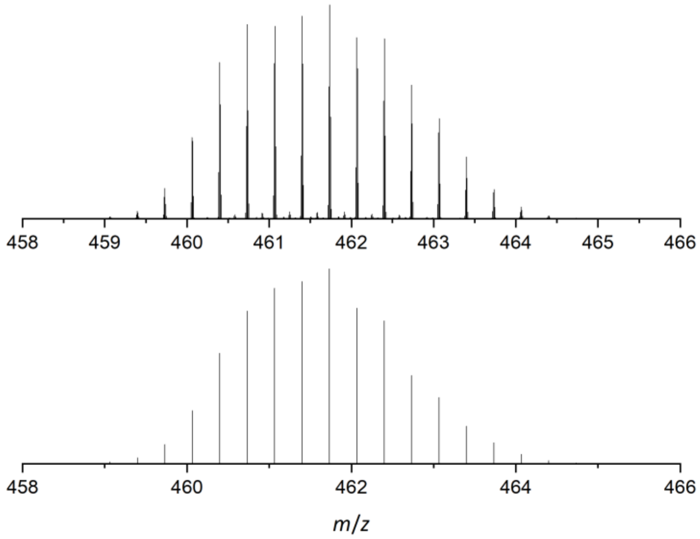

Figure S214 Observed (top) and calculated (bottom) isotopic patterns for $\{[Pd_2(1CE)_2(2AA)_2](BF_4)\}^{3+}$.

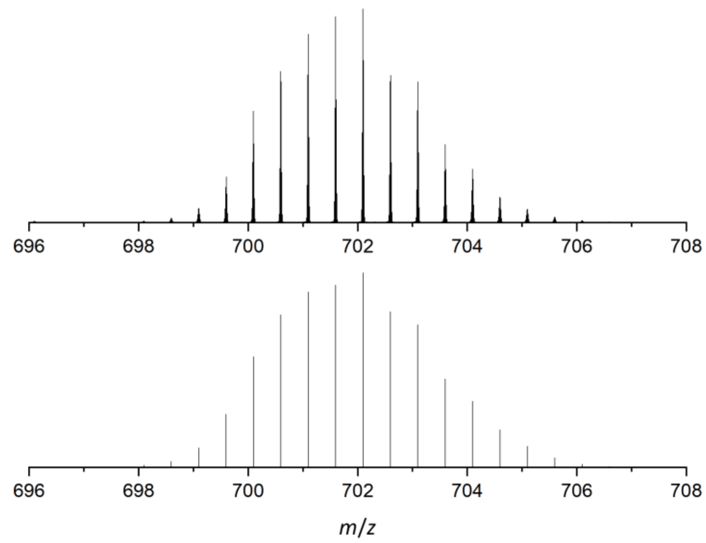

Figure S215 Observed (top) and calculated (bottom) isotopic patterns for $\{[Pd_2(1CE)_2(2AA)_2](BF_4)F\}^{2+}$.

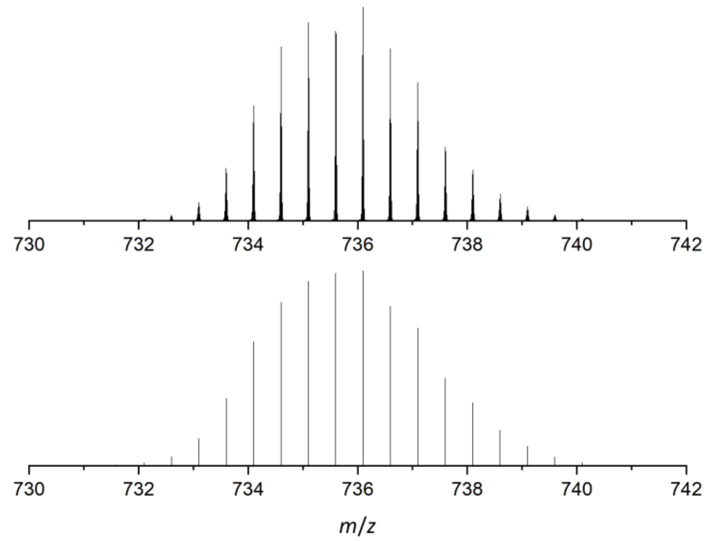

Figure S216 Observed (top) and calculated (bottom) isotopic patterns for $\{[Pd_2(1CE)_2(2AA)_2](BF_4)_2\}^{2+}$.

Synthesis of $[Pd_2(\mathbf{1AA})_2(\mathbf{2AB})_2](BF_4)_4$

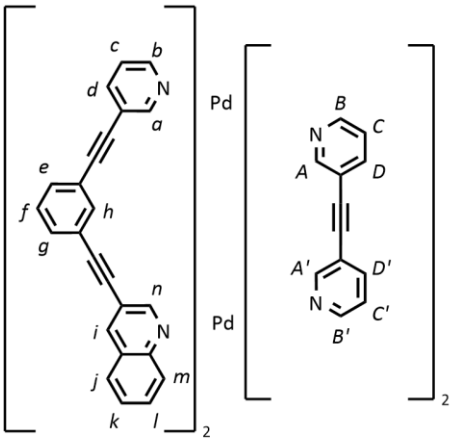

1AA (5.4 mg, 30 µmol, 1 eq.), **2AB** (9.9 mg, 30 µmol, 1 eq.) and $[Pd(CH_3CN)_4](BF_4)_2$ (13.3 mg, 30 µmol, 1 eq.) were stirred at 70 °C in anhydrous CH_3CN (5.0 mL) under N_2 for 24 h. The cooled reaction mixture was filtered through celite. Vapour diffusion of Et_2O into this solution yielded a precipitate. After the mother liquor was decanted, the solid was washed with Et_2O (× 3) and dried *in vacuo* to give the product as a beige solid (16.9 mg, 71%).

ESI-MS *m/z* = 440.06 $\{[Pd_2(\mathbf{1AA})_2(\mathbf{2AB})_2](BF_4)\}^{3+}$ calc. 440.39; 670.09 $\{[Pd_2(\mathbf{1AA})_2(\mathbf{2AB})_2](BF_4)F\}^{2+}$ calc. 670.09.

^{13}C NMR (101 MHz, CD_3CN) δ: 157.20 (*anti*-C_n), 156.78 (*syn*-C_n), 155.28, 155.00 (*anti*-C_a, *syn*-C_a), 154.58 (*anti*-C_A, *anti*-$C_{A'}$), 153.02 (*syn*-C_B), 152.60 (*anti*-C_B, *anti*-$C_{B'}$), 152.29 (*syn*-C_b), 150.71 (*anti*-C_b), 150.36 (*syn*-$C_{B'}$), 145.13, 144.86, 143.76 (*syn*-C_i), 143.31, 143.18 (*anti*-C_i), 143.14 (*anti*-C_D /*anti*-$C_{D'}$), 143.03 (*anti*-C_D /*anti*-$C_{D'}$), 142.87, 142.57 (*anti*-C_d), 142.21, 139.12 (*anti*-C_h), 136.03 (*anti*-C_l), 135.05 (*syn*-C_l), 132.57 (*anti*-C_e /*anti*-C_g), 132.25 (*anti*-C_e /*anti*-C_g), 132.11, 131.22 (*anti*-C_j), 131.19 (*anti*-C_k), 131.01 (*syn*-C_k), 130.78 (*anti*-C_f), 130.20, 128.85, 128.71 (*anti*-C_c), 128.52 (*anti*-C_C, *anti*-$C_{C'}$), 127.79 (*syn*-C_m), 127.10 (*anti*-C_m), 125.11, 124.87, 124.23, 124.14, 123.96, 123.29, 123.06, 120.28, 118.32, 95.71, 94.60, 90.69, 90.33, 86.52, 85.97.

^{19}F NMR (376 MHz, CD_3CN) δ: -151.22, -148.70.

^{1}H DOSY (400 MHz, CD_3CN) *D*: 6.8×10^{-10} m^2s^{-1}; R_S: 9.0 Å.

Major Isomer *anti*-[Pd$_2$(1AA)$_2$(2AB)$_2$](BF$_4$)$_4$

^{1}H NMR (600 MHz, CD$_3$CN) δ: 10.46 (dd, *J* = 8.5, 0.9 Hz, 2H, H$_m$), 10.08 (s, 2H, H$_{A'}$), 10.04 (s, 2H, H$_A$), 9.82 (s, 2H, H$_n$), 9.45 (s, 2H, H$_a$), 9.28 (ddd, *J* = 5.9, 1.3, 0.6 Hz, 2H, H$_B$), 8.98 (ddd, *J* = 5.8, 1.3, 0.6 Hz, 2H, H$_b$), 8.88 (ddd, *J* = 5.9, 1.3, 0.6 Hz, 2H, H$_{B'}$), 8.64 (app. dt, *J* = 1.6, 0.7 Hz, 2H, H$_i$), 8.50 (ddd, *J* = 8.6, 7.1, 1.4 Hz, 2H, H$_l$), 8.31 (app. td, *J* = 1.7, 0.6 Hz, 2H, H$_h$), 8.13 (app. ddt, *J* = 8.3, 1.3, 0.6 Hz, 2H, H$_j$), 8.08 (ddd, *J* = 7.9, 1.8, 1.3 Hz, 2H, H$_D$), 8.03-7.97 (m, 4H, H$_k$, H$_{D'}$), 7.94 (ddd, *J* = 8.0, 1.8, 1.3 Hz, 2H, H$_d$), 7.68-7.63 (m, 4H, H$_C$, H$_{e/g}$), 7.59 (m, 2H, H$_{e/g}$), 7.53-7.50 (m, 4H, H$_f$, H$_c$), 7.47 (ddd, *J* = 7.9, 5.9, 0.6 Hz, 2H, H$_{C'}$).

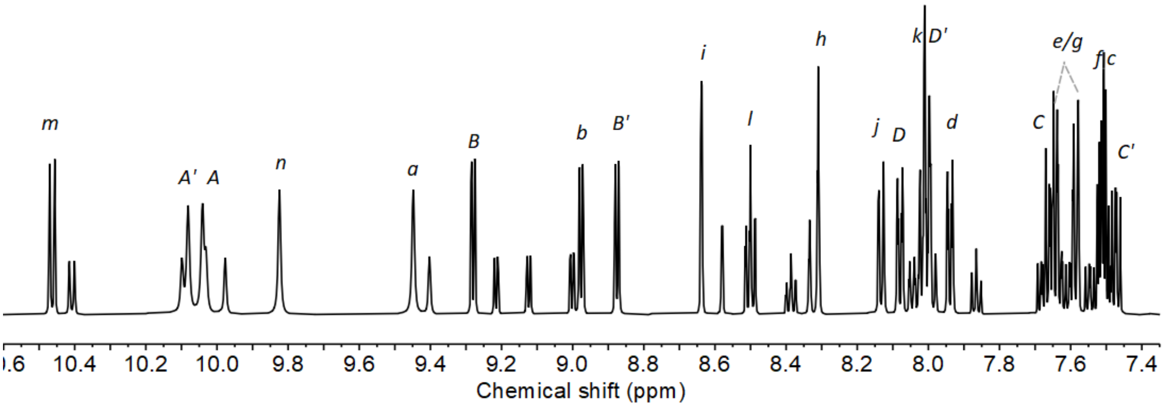

Figure S217 Partial ^{1}H NMR (600 MHz, CD$_3$CN) of [Pd$_2$(1AA)$_2$(2AB)$_2$](BF$_4$)$_4$ with peaks of major *anti*-isomer labelled.

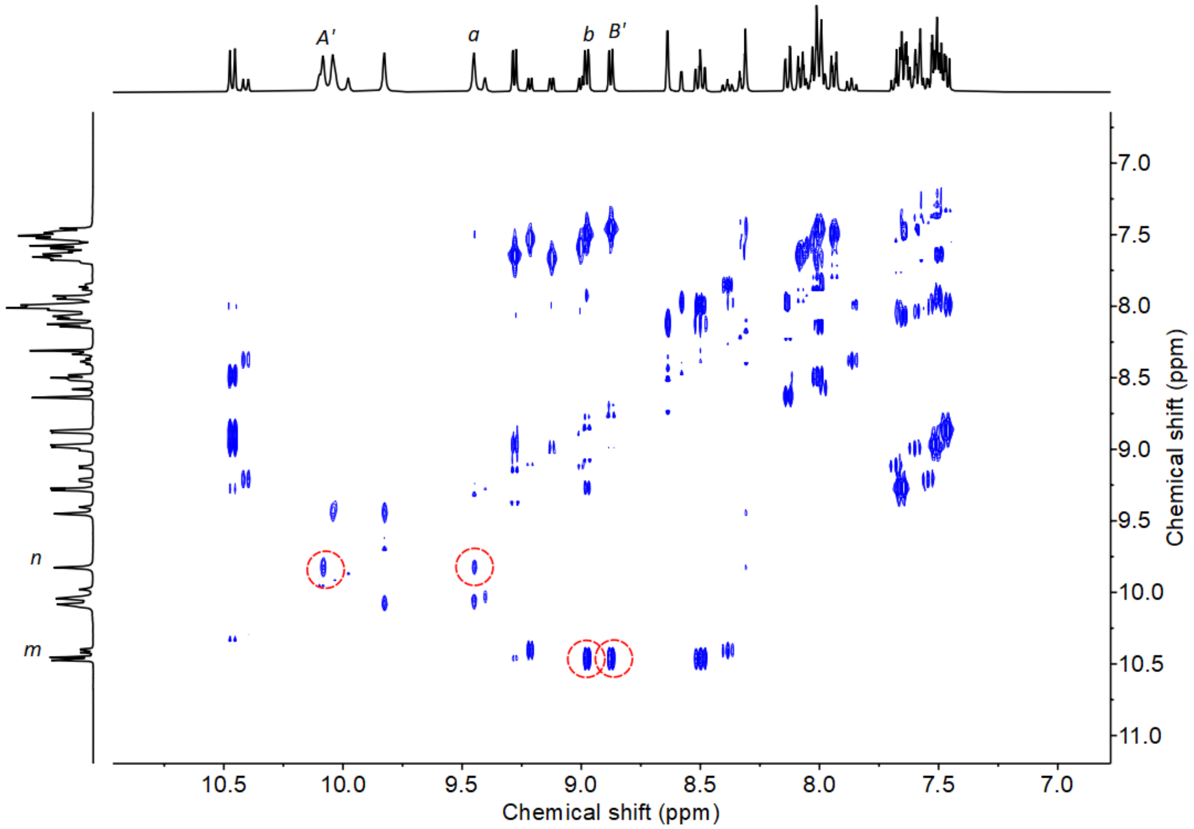

Figure S218 Partial NOESY (400 MHz, CD$_3$CN) of [Pd$_2$(1AA)$_2$(2AB)$_2$](BF$_4$)$_4$ labelled for minor isomer with through-space interactions used to identify it as the *anti*-isomer.

Minor Isomer *syn*-$[Pd_2(1AA)_2(2AB)_2](BF_4)_4$

^{1}H NMR (600 MHz, CD_3CN) δ: 10.41 (dd, *J* = 8.6, 1.0 Hz, 2H, H_m), 10.10 (s, 2H, H_A), 10.03 (s, 2H, $H_{A'}$), 9.98 (s, 2H, H_n), 9.42-9.38 (m, 2H, H_a), 9.22 (ddd, *J* = 6.0, 1.3, 0.6 Hz, 2H, H_B), 9.12 (ddd, *J* = 5.8, 1.3, 0.6 Hz, 2H, $H_{B'}$), 9.00 (ddd, *J* = 5.8, 1.4, 0.5 Hz, 2H, H_b), 8.58 (dd, *J* = 1.8, 0.8 Hz, 2H, H_i), 8.39 (ddd, *J* = 8.6, 7.1, 1.4 Hz, 2H, H_l), 8.33 (app. td, *J* = 1.7, 0.6 Hz, 2H, H_h), 8.05 (dt, *J* = 8.0, 1.5 Hz, 2H, H_d), 8.03-7.97 (m, 6H, H_D, $H_{D'}$, H_j), 7.87 (ddd, *J* = 8.1, 7.1, 0.9 Hz, 2H, H_k), 7.70-7.67 (m, 2H, H_C), 7.68-7.63 (m, 2H, $H_{e/g}$), 7.62-7.60 (m, 2H, H_c), 7.59 (m, 2H, $H_{e/g}$), 7.55 (ddd, *J* = 7.9, 5.9, 0.6 Hz, 2H, H_C), 7.53-7.50 (m, 2H, H_f).

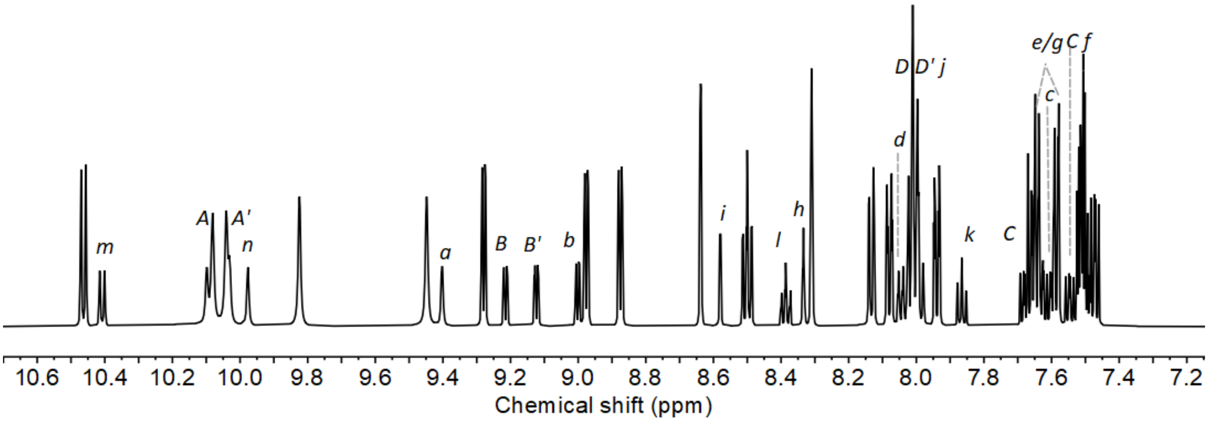

Figure S219 Partial ^{1}H NMR (600 MHz, CD_3CN) of $[Pd_2(1AA)_2(2AB)_2](BF_4)_4$ with peaks of minor *syn*-isomer labelled.

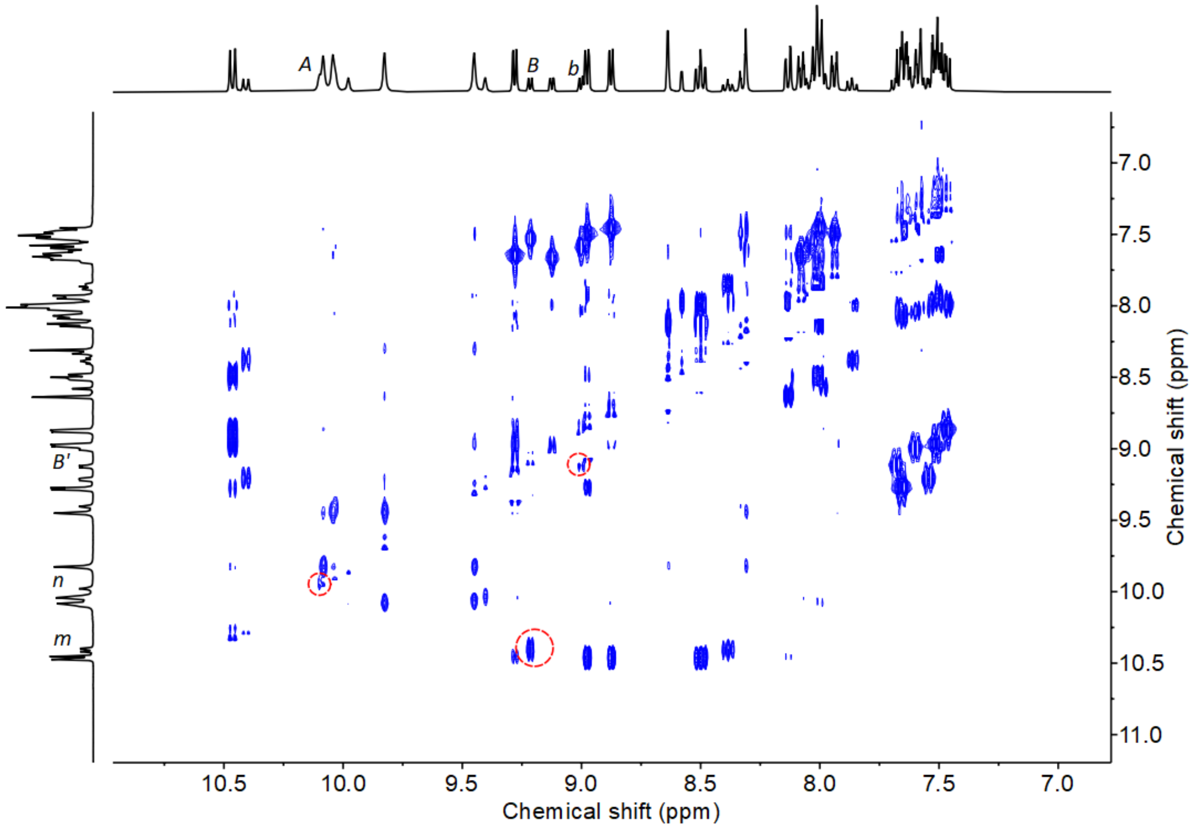

Figure S220 Partial NOESY (400 MHz, CD_3CN) of $[Pd_2(1AA)_2(2AB)_2](BF_4)_4$ labelled for minor isomer with through-space interactions used to identify it as the *syn*-isomer.

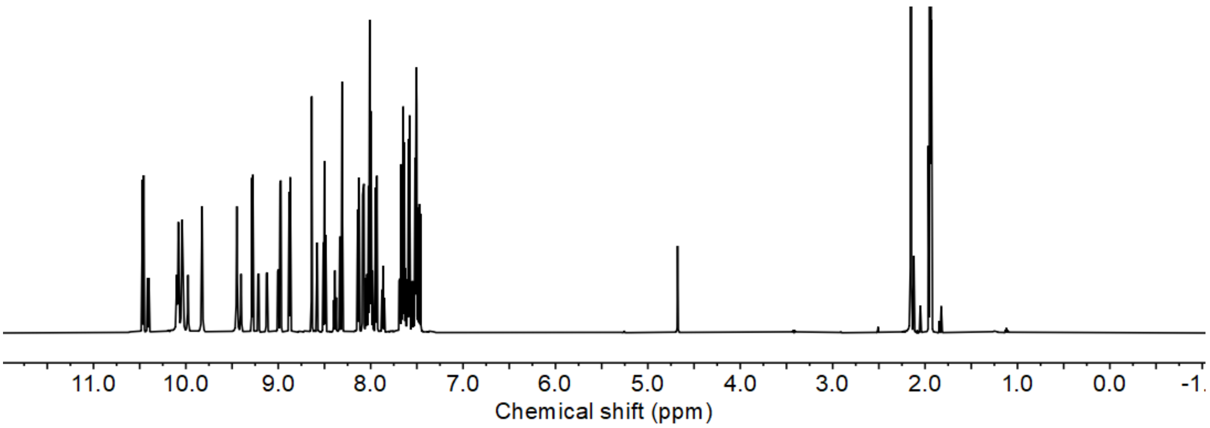

Figure S221 ^{1}H NMR (600 MHz, CD_3CN) of $[Pd_2(1AA)_2(2AB)_2](BF_4)_4$.

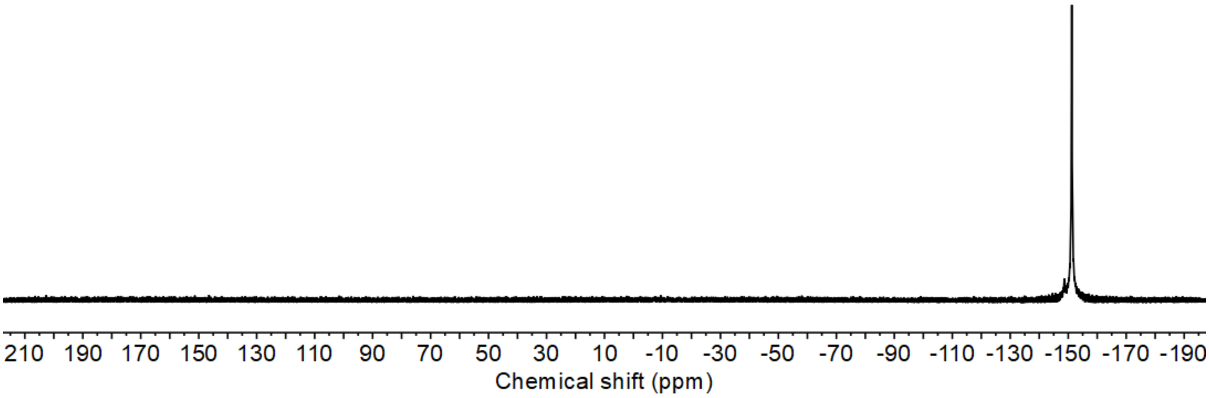

Figure S222 ^{19}F NMR (376 MHz, CD_3CN) of $[Pd_2(1AA)_2(2AB)_2](BF_4)_4$.

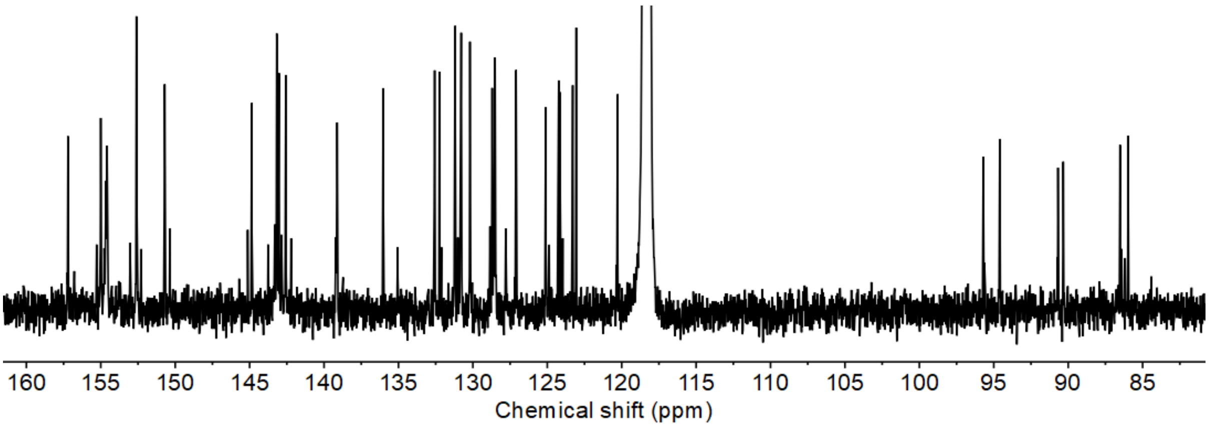

Figure S223 Partial ^{13}C NMR (101 MHz, CD_3CN) of $[Pd_2(1AA)_2(2AB)_2](BF_4)_4$.

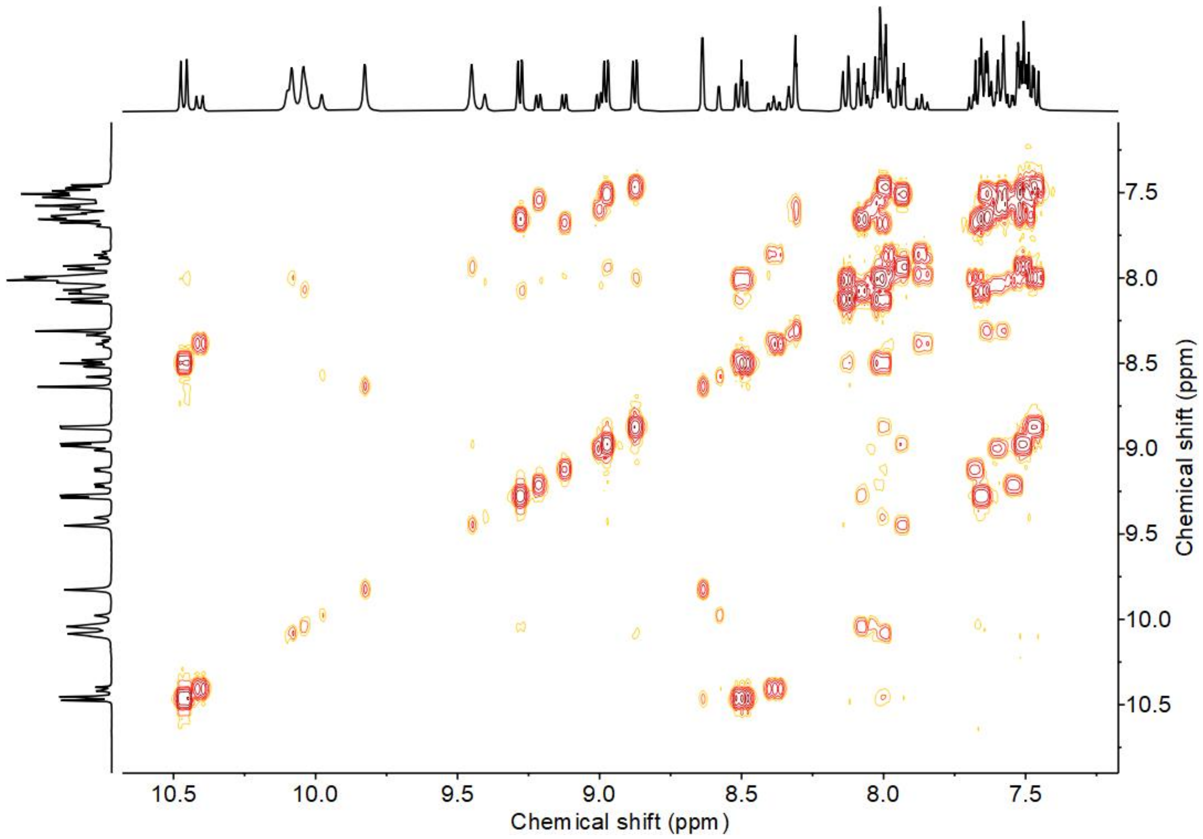

Figure S224 Partial COSY (CD_3CN) of [$Pd_2(1AA)_2(2AB)_2$](BF_4)$_4$.

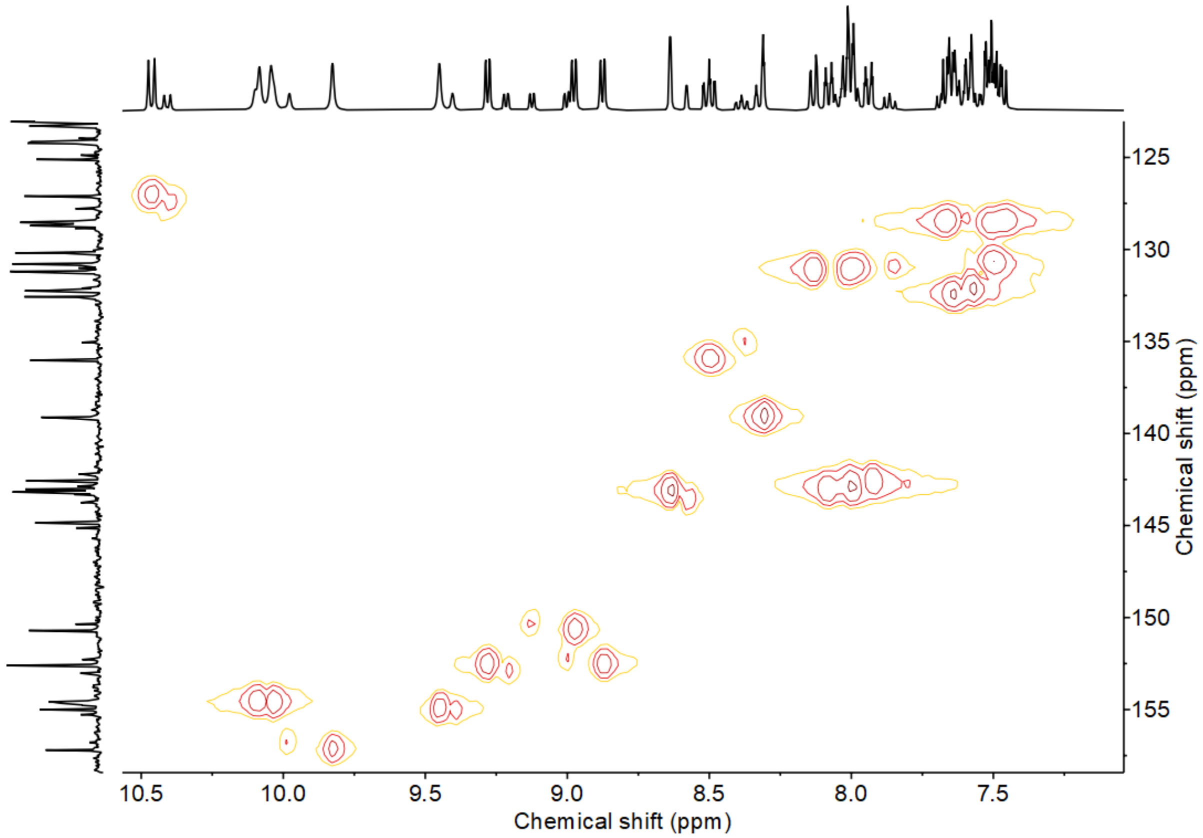

Figure S225 Partial HSQC (CD_3CN) of [$Pd_2(1AA)_2(2AB)_2$](BF_4)$_4$.

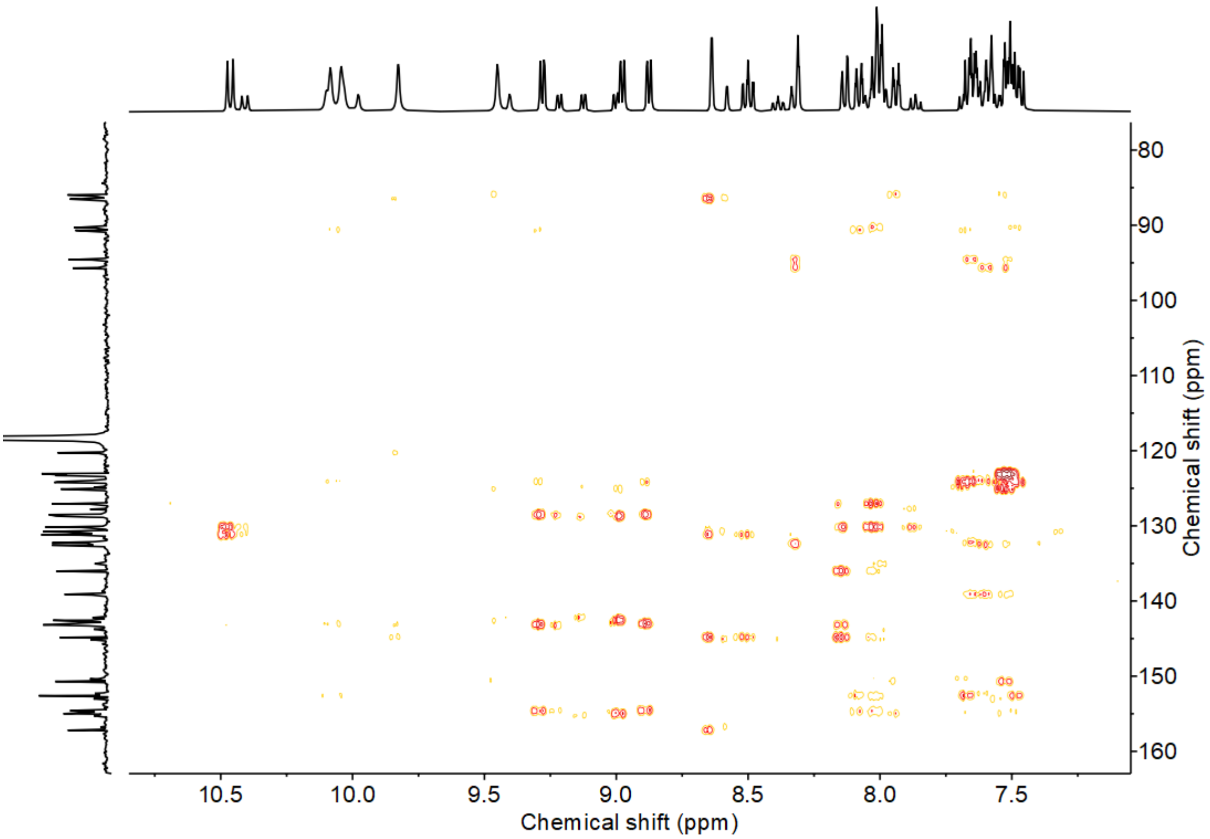

Figure S226 Partial HMBC (CD_3CN) of $[Pd_2(1AA)_2(2AB)_2](BF_4)_4$.

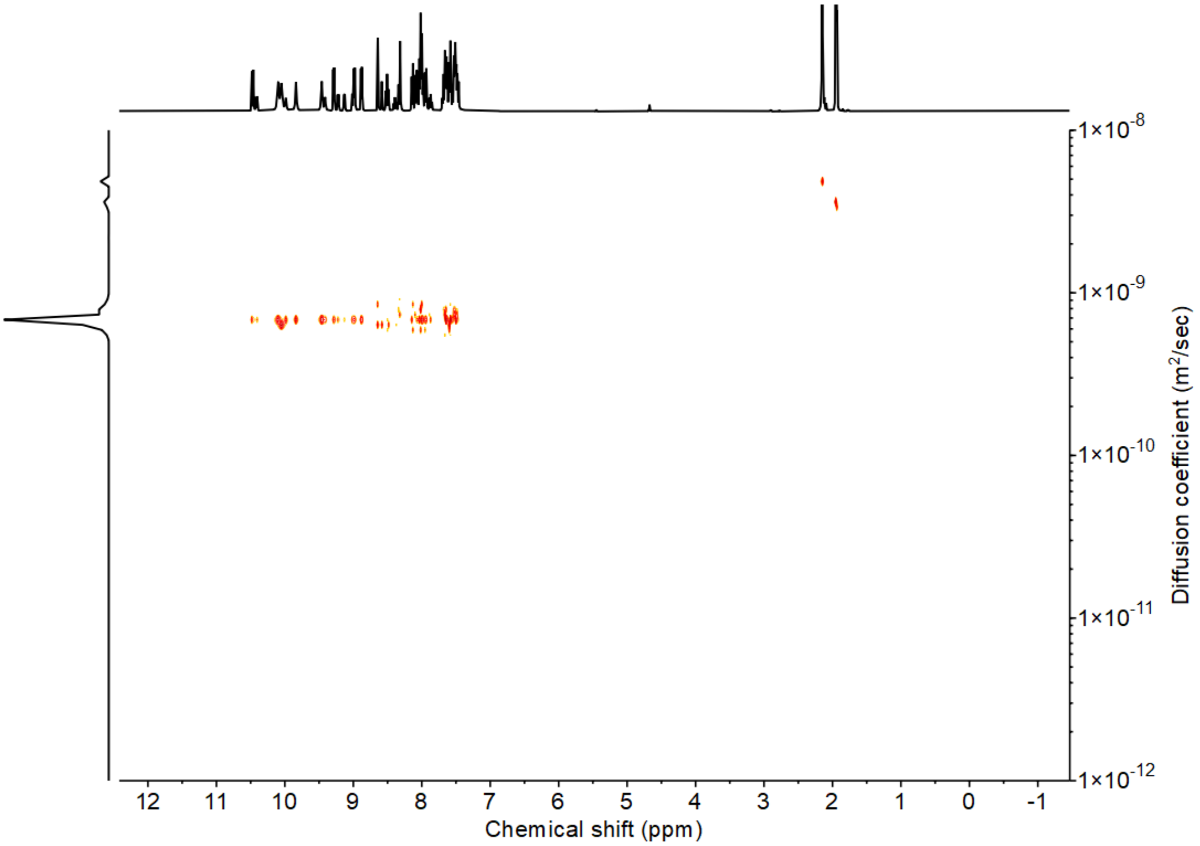

Figure S227 DOSY (CD_3CN) of $[Pd_2(1AA)_2(2AB)_2](BF_4)_4$.

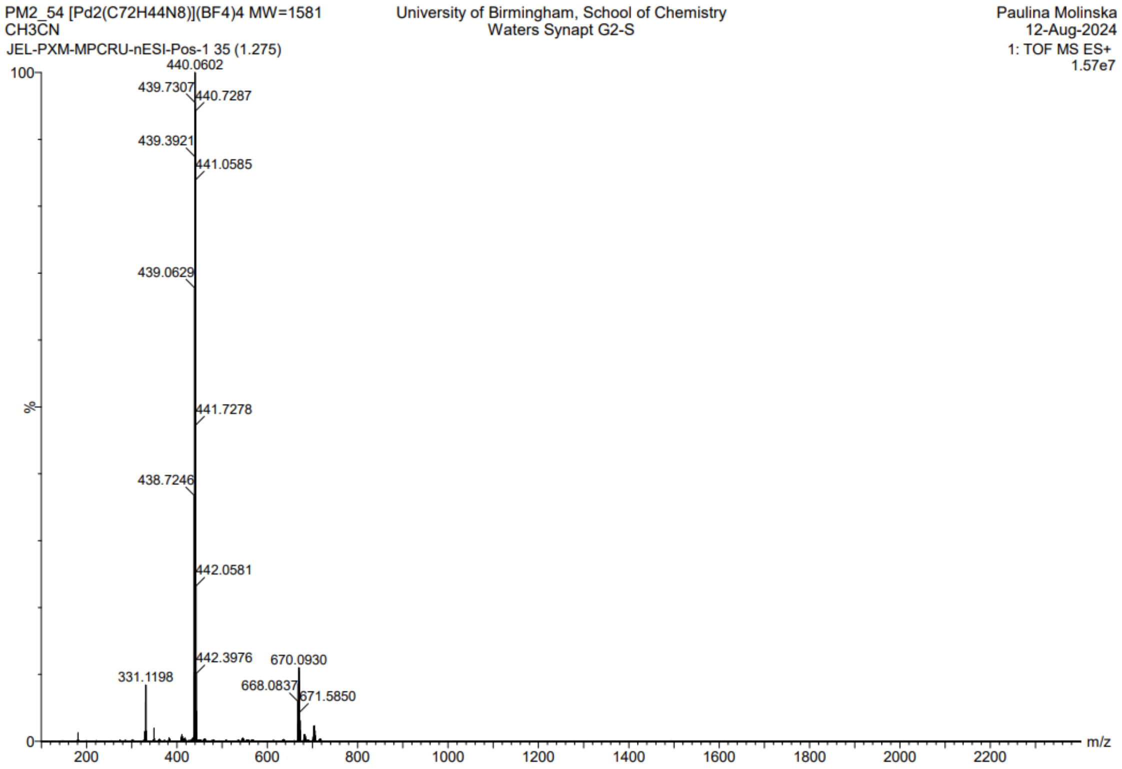

Figure S228 ESI-MS of $[Pd_2(1AA)_2(2AB)_2](BF_4)_4$.

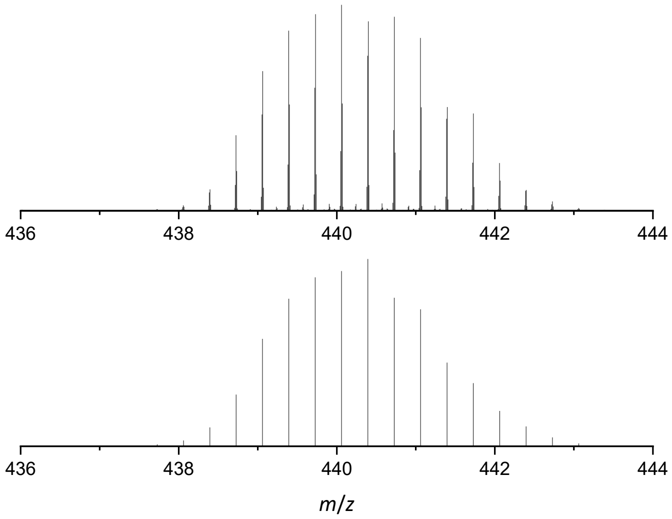

Figure S229 Observed (top) and calculated (bottom) isotopic patterns for $\{[Pd_2(1AA)_2(2AB)_2](BF_4)\}^{3+}$.

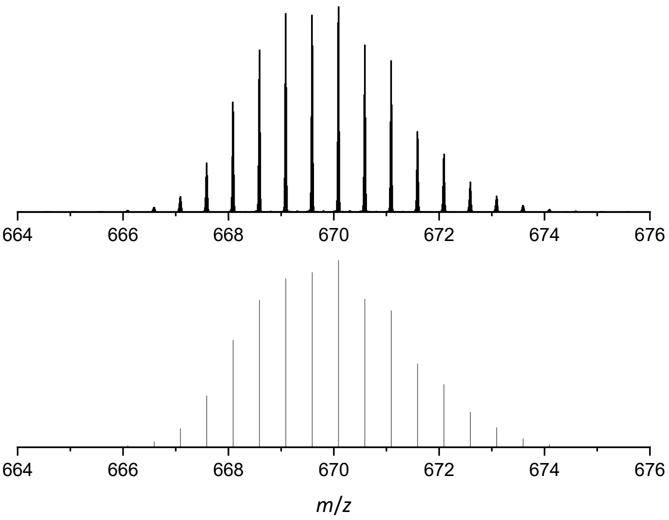

Figure S230 Observed (top) and calculated (bottom) isotopic patterns for $\{[Pd_2(1AA)_2(2AB)_2](BF_4)F\}^{2+}$.

Synthesis of $[Pd_2(\mathbf{1AA})_2(\mathbf{2AC})_2](BF_4)_4$

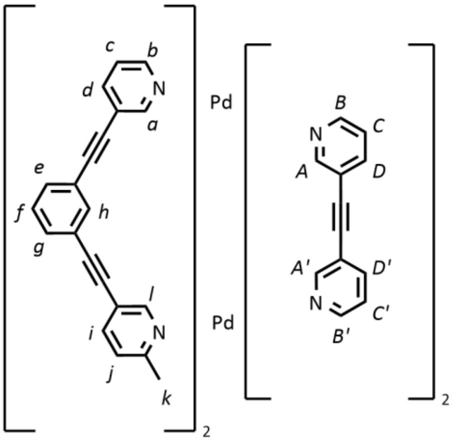

1AA (5.4 mg, 30 µmol, 1 eq.), **2AC** (8.8 mg, 30 µmol, 1 eq.) and $[Pd(CH_3CN)_4](BF_4)_2$ (13.3 mg, 30 µmol, 1 eq.) were stirred at 70 °C in anhydrous CH_3CN (5.0 mL) under N_2 for 24 h. The cooled reaction mixture was filtered through celite. Vapour diffusion of Et_2O into this solution yielded a precipitate. After the mother liquor was decanted, the solid was washed with Et_2O (× 3) and dried *in vacuo*. The solid was re-dissolved in DMF. Vapour diffusion of Et_2O into this solution yielded a precipitate. After the mother liquor was decanted, the solid was washed with Et_2O (× 3) and dried *in vacuo* to give the product as a beige solid (15.2 mg, 67%).

ESI-MS m/z = $\{[Pd_2(\mathbf{1AA})_2(\mathbf{2AC})_2](BF_4)\}^{3+}$ calc. 416.39; 634.09 $\{[Pd_2(\mathbf{1AA})_2(\mathbf{2AC})_2](BF_4)F\}^{2+}$ calc. 634.09.

^{13}C NMR (101 MHz, CD_3CN) δ: 161.16, 155.34 (*syn*-C_a), 154.78 (*anti*-C_a), 154.57 (*anti*-C_A), 154.34 (*anti*-$C_{A'}$), 153.67 (*anti*-C_l), 153.14 (*syn*-C_l), 152.92 (*anti*-$C_{B'}$, *syn*-C_B, *syn*-$C_{B'}$), 152.49 (*anti*-C_B), 150.94 (*anti*-C_b), 150.31 (*syn*-C_b), 143.45, 143.21 (*anti*-C_d /*anti*-C_D /*anti*-$C_{D'}$), 143.15 (*anti*-C_d /*anti*-C_D /*anti*-$C_{D'}$), 142.86, 142.67 (*anti*-C_d /*anti*-C_D /*anti*-$C_{D'}$), 142.48 (*anti*-C_i), 142.13, 139.24 (*anti*-C_h, *syn*-C_h), 132.54 (*anti*-C_C /*anti*-$C_{C'}$), 132.09 (*anti*-C_C /*anti*-$C_{C'}$), 130.76 (*anti*-C_f), 129.22, 129.06 (*anti*-C_c /*anti*-C_j), 128.98 (*anti*-C_c /*anti*-C_j), 128.84, 128.70 (*anti*-C_e /*anti*-C_g), 128.47 (*anti*-C_e /*anti*-C_g), 125.20, 124.24, 124.02, 123.31, 123.00, 122.37, 122.29, 95.84, 94.49, 90.67, 90.22, 85.99, 85.83, 27.80 (*syn*-C_k), 26.59 (*anti*-C_k).

^{19}F NMR (376 MHz, CD_3CN) δ: -151.44.

^{1}H DOSY (400 MHz, CD_3CN) *D*: 7.9 × 10^{-10} m^2s^{-1}; R_S: 7.7 Å.

Major Isomer *anti*-[Pd_2(1AA)$_2$(2AC)$_2$](BF_4)$_4$

^{1}H NMR (400 MHz, CD_3CN) δ: 9.96 (d, *J* = 1.8 Hz, 2H, $H_{A'}$), 9.91 (d, *J* = 1.7 Hz, 2H, H_A), 9.51 (d, *J* = 1.8 Hz, 2H, H_l), 9.35-9.32 (m, 2H, H_a), 9.12-9.08 (m, 2H, H_B), 9.00-8.94 (m, 2H, H_b), 8.87 (ddd, *J* = 5.8, 1.3, 0.6 Hz, 2H, $H_{B'}$), 8.24 (app. td, *J* = 1.7, 0.6 Hz, 2H, H_h), 8.08-8.01 (m, 6H, H_D, $H_{D'}$, H_d), 7.92 (dd, *J* = 8.2, 1.9 Hz, 2H, H_i), 7.69 (ddd, *J* = 8.0, 5.8, 0.7 Hz, 2H, H_c), 7.62-7.56 (m, 8H, H_C, $H_{C'}$, H_e, H_g), 7.56-7.53 (m, 2H, H_j), 7.49 (m, 2H, H_f), 3.75 (s, 6H, H_k).

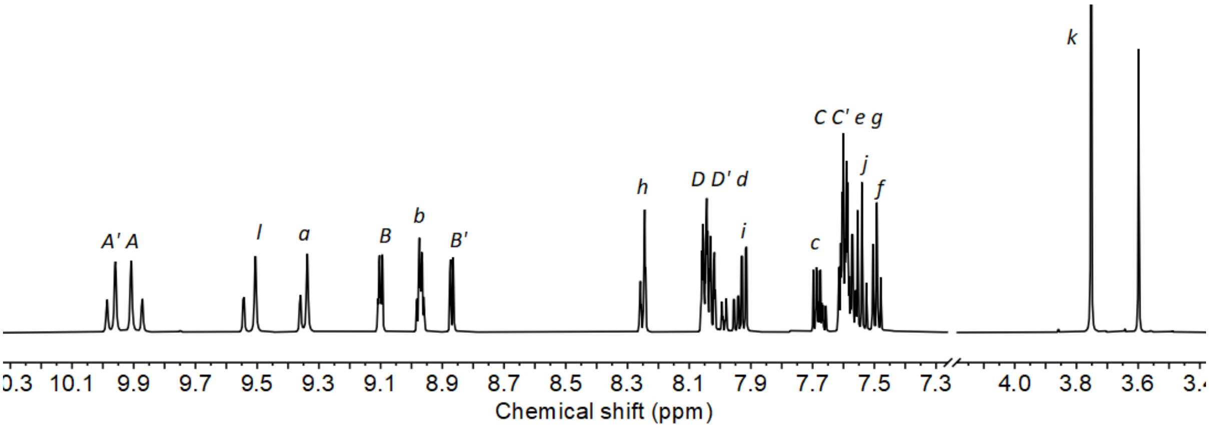

Figure S231 Partial ^{1}H NMR (600 MHz, CD_3CN) of [Pd_2(1AA)$_2$(2AC)$_2$](BF_4)$_4$ with peaks of major *anti*-isomer labelled.

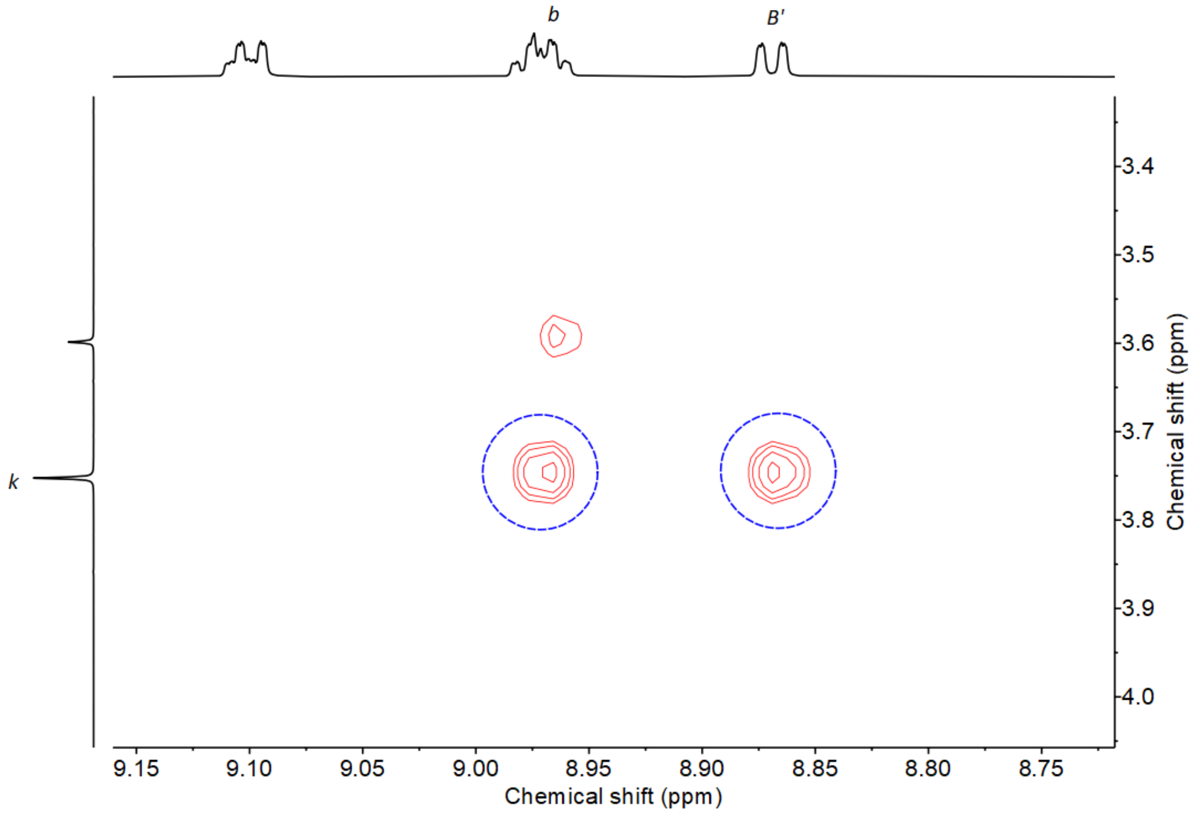

Figure S232 Partial NOESY Partial NOESY (600 MHz, CD_3CN) of [Pd_2(1AA)$_2$(2AC)$_2$](BF_4)$_4$ labelled for major isomer with through-space interactions used to identify it as the *anti*-isomer.

Minor Isomer *syn*-$[Pd_2(1AA)_2(2AC)_2](BF_4)_4$

^{1}H NMR (400 MHz, CD_3CN) δ: 9.99 (d, *J* = 1.8 Hz, 2H, $H_{A'}$), 9.87 (d, *J* = 1.7 Hz, 2H, H_A), 9.54 (d, *J* = 1.8 Hz, 2H, H_l), 9.36 (d, *J* = 1.8 Hz, 2H, H_a), 9.12-9.08 (m, 2H, H_b), 9.00-8.94 (m, 4H, H_B, $H_{B'}$), 8.26 (app. td, *J* = 1.7, 0.6 Hz, 2H, H_h), 8.08-8.01 (m, 6H, H_D, $H_{D'}$), 7.99 (ddd, *J* = 8.0, 1.8, 1.4 Hz, 2H, H_d), 7.95 (dd, *J* = 8.2, 1.8 Hz, 2H, H_d), 7.67 (ddd, *J* = 8.0, 5.9, 0.7 Hz, 2H, H_c), 7.62-7.56 (m, 8H, H_C, $H_{C'}$, H_e, H_g), 7.56-7.53 (m, 2H, H_j), 7.49 (m, 2H, H_f), 3.60 (s, 6H, H_k).

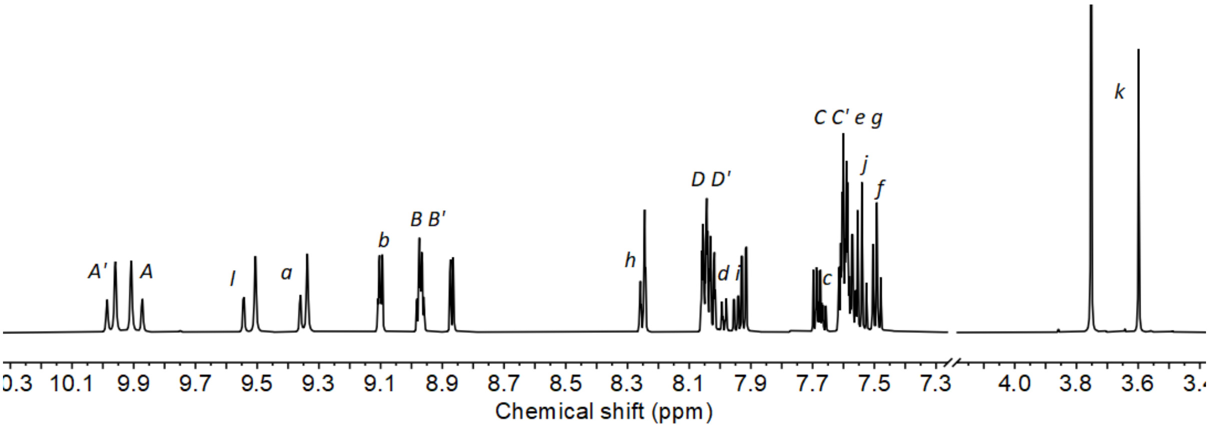

Figure S233 Partial ^{1}H NMR (400 MHz, CD_3CN) of $[Pd_2(1AA)_2(2AC)_2](BF_4)_4$ with peaks of minor *syn*-isomer labelled.

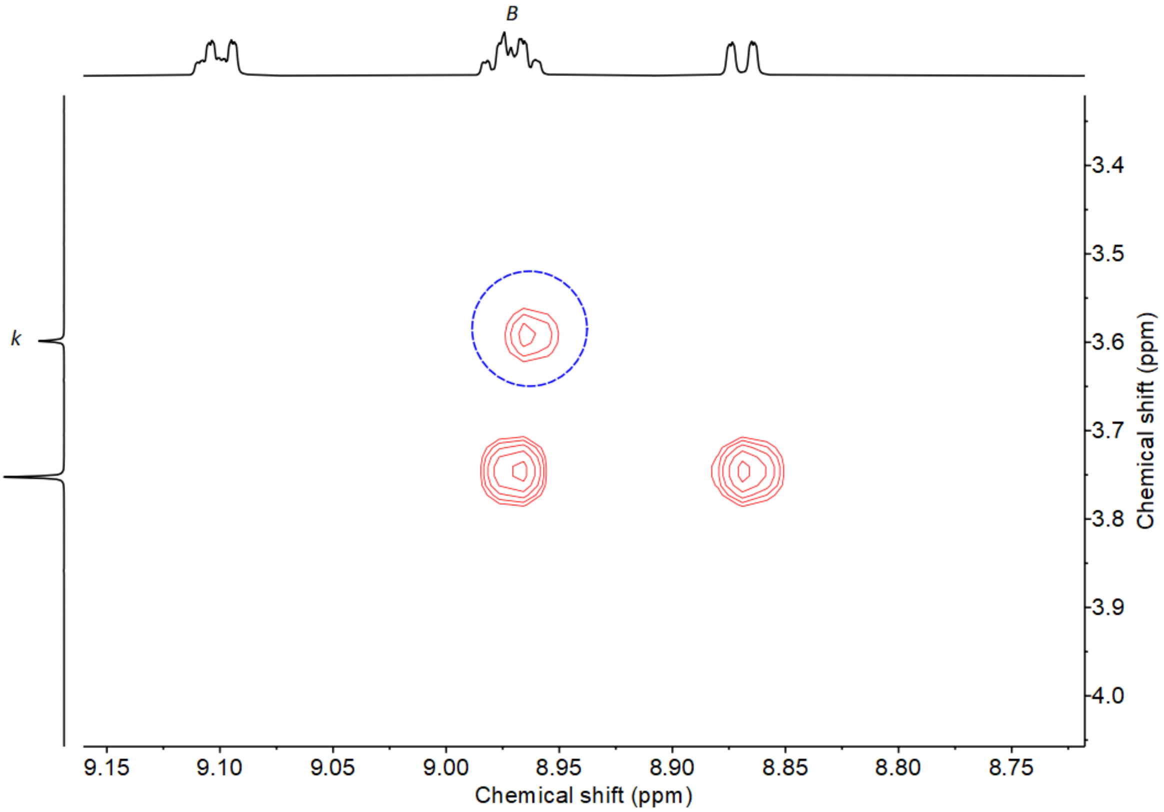

Figure S234 Partial NOESY Partial NOESY (600 MHz, CD_3CN) of $[Pd_2(1AA)_2(2AC)_2](BF_4)_4$ labelled for minor isomer with through-space interactions used to identify it as the *syn*-isomer.

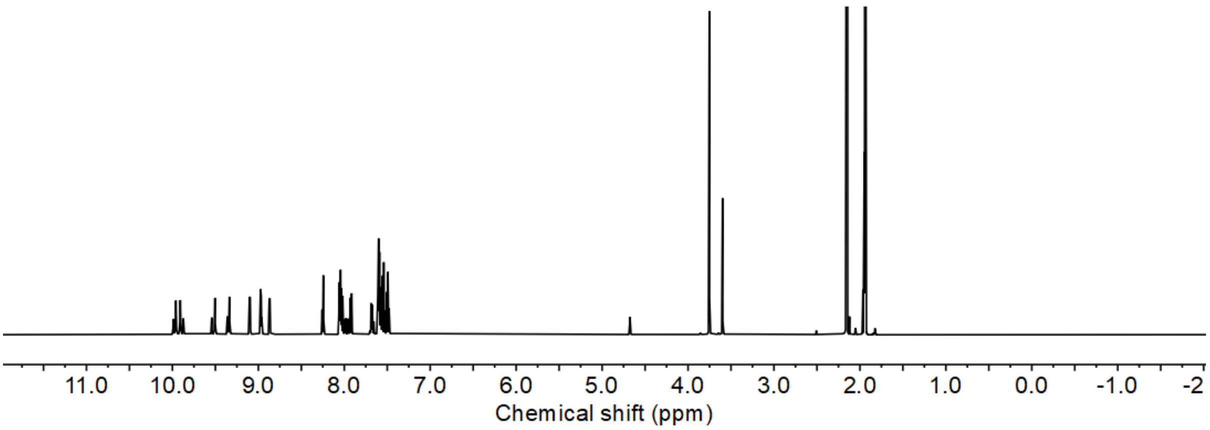

Figure S235 ^{1}H **NMR (600 MHz,** CD_3CN**) of** $[Pd_2(1AA)_2(2AC)_2](BF_4)_4$**.**

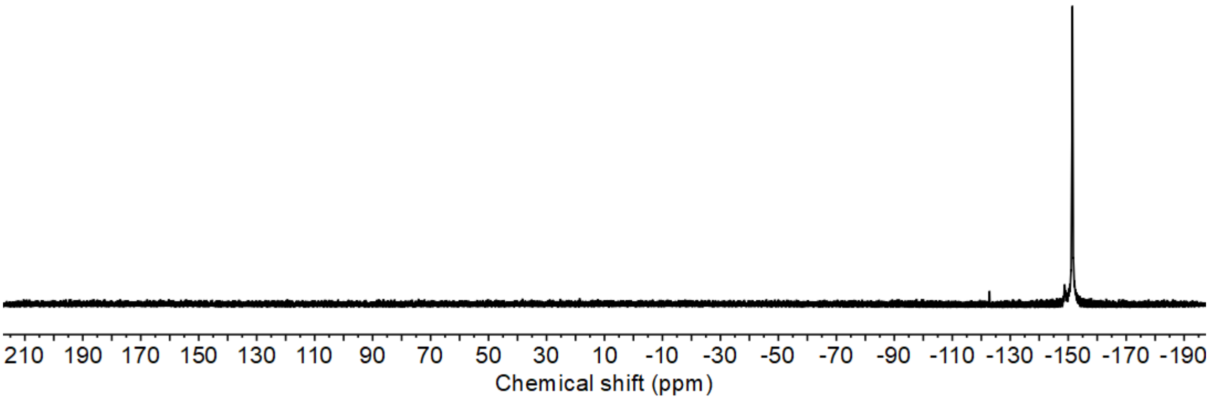

Figure S236 ^{19}F **NMR (376 MHz,** CD_3CN**) of** $[Pd_2(1AA)_2(2AC)_2](BF_4)_4$**.**

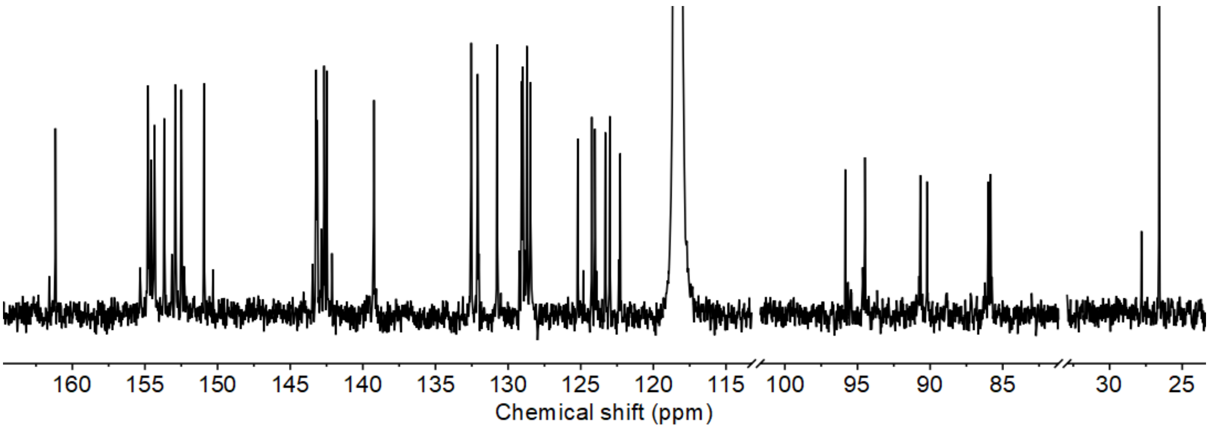

Figure S237 Partial ^{13}C **NMR (101 MHz,** CD_3CN**) of** $[Pd_2(1AA)_2(2AC)_2](BF_4)_4$**.**

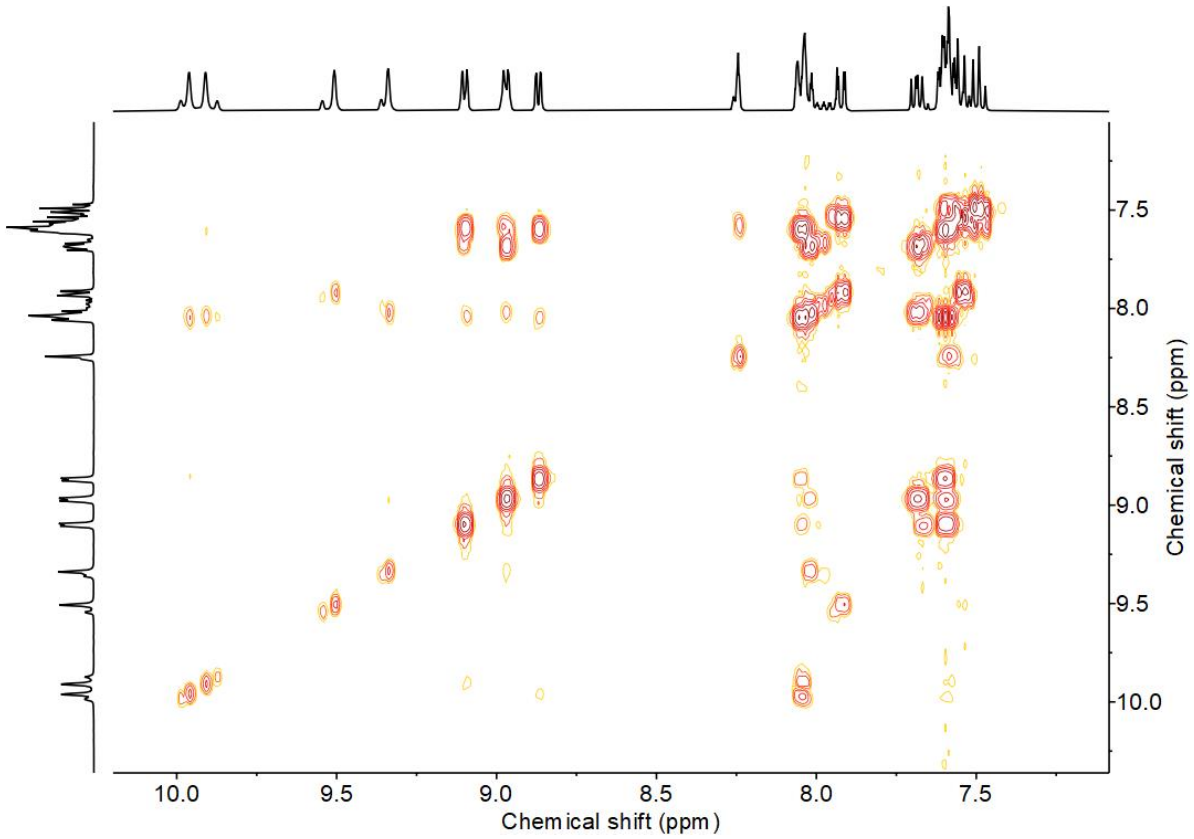

Figure S238 Partial COSY (CD_3CN) of $[Pd_2(1AA)_2(2AC)_2](BF_4)_4$.

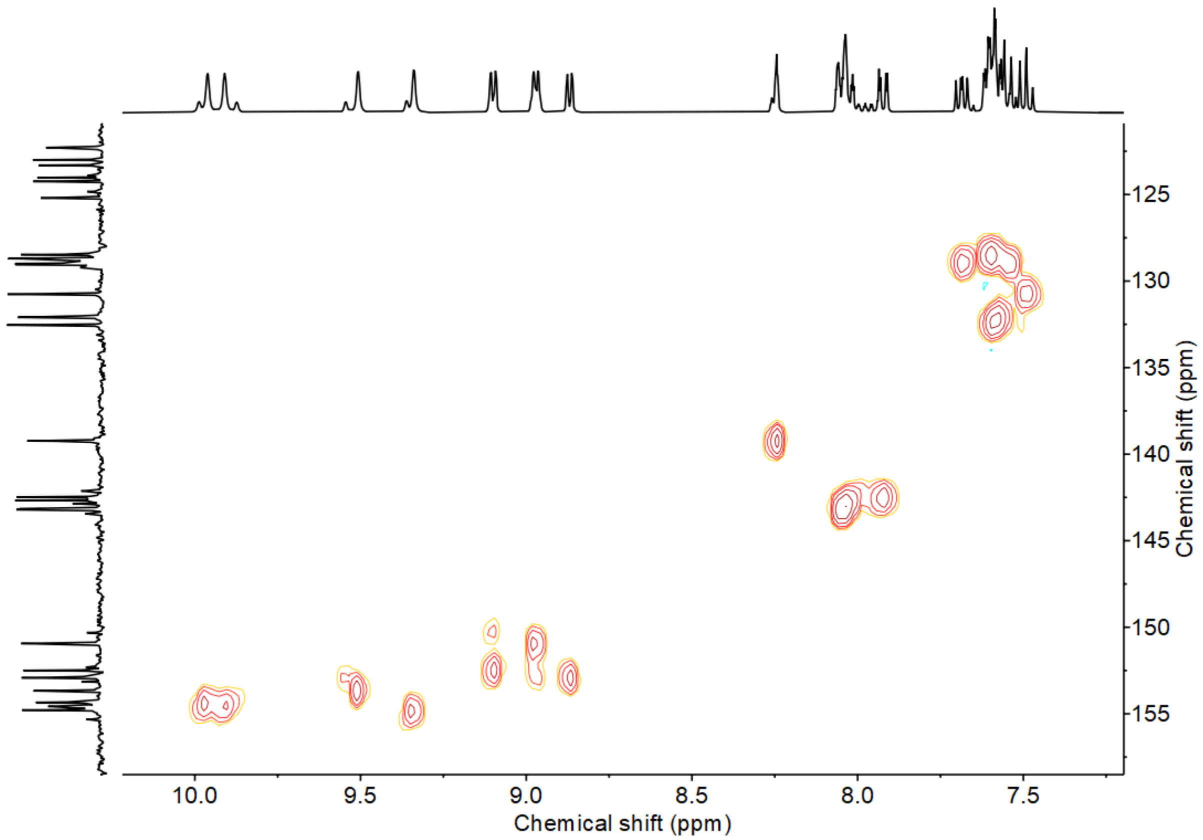

Figure S239 Partial HSQC (CD_3CN) of $[Pd_2(1AA)_2(2AC)_2](BF_4)_4$.

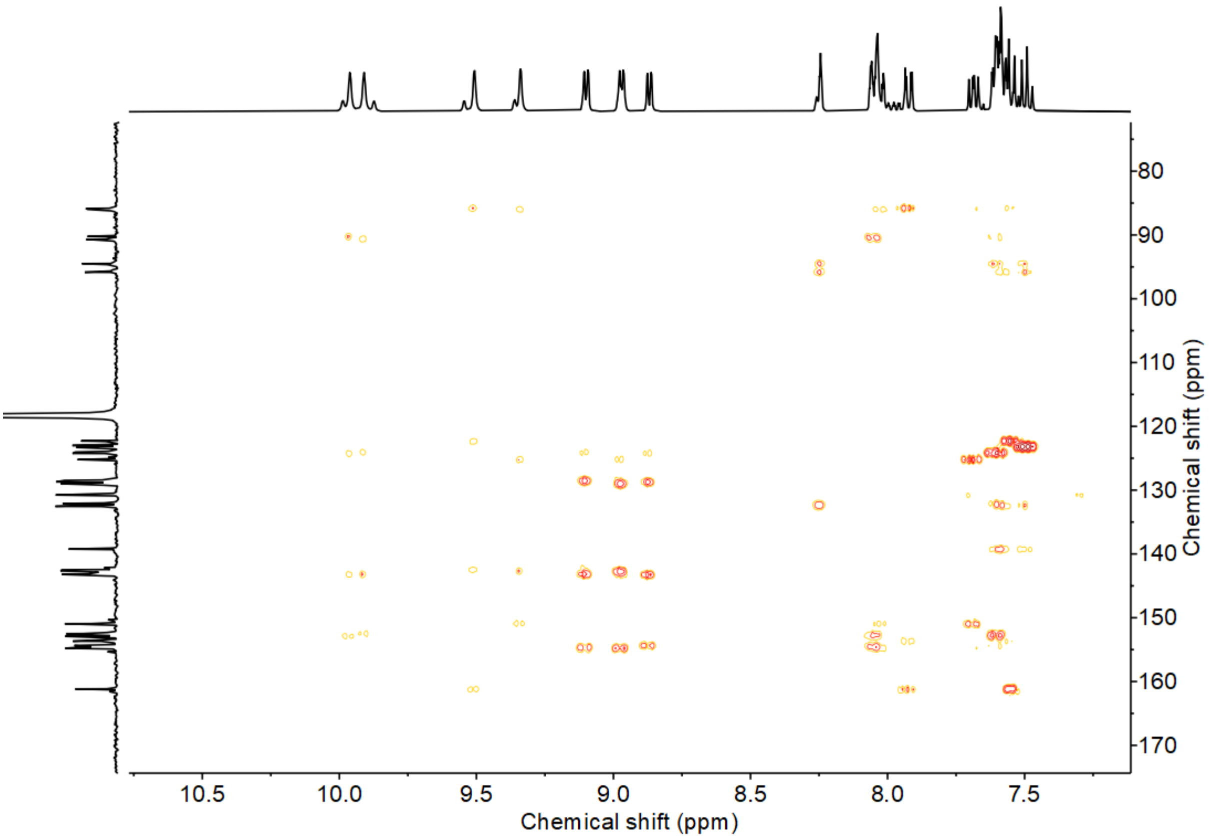

Figure S240 Partial HMBC (CD_3CN) of $[Pd_2(1AA)_2(2AC)_2](BF_4)_4$.

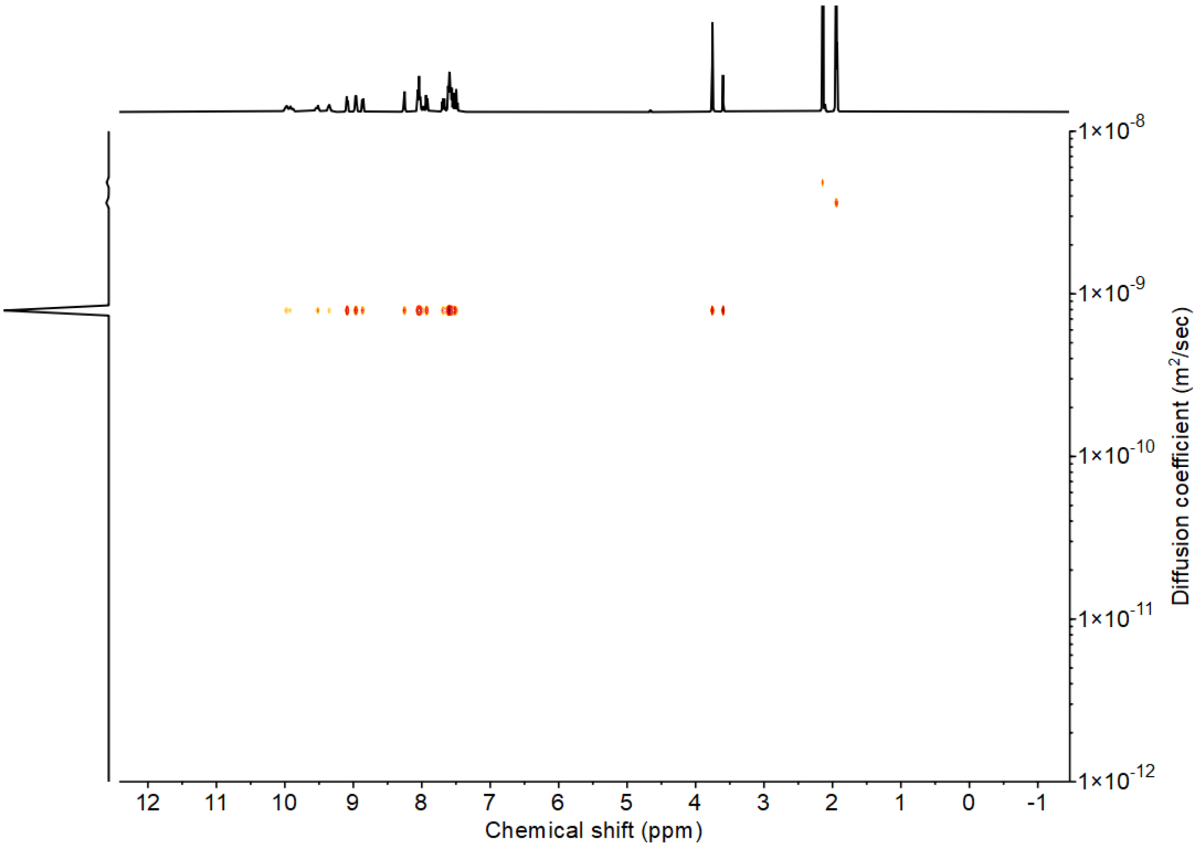

Figure S241 DOSY (CD_3CN) of $[Pd_2(1AA)_2(2AC)_2](BF_4)_4$.

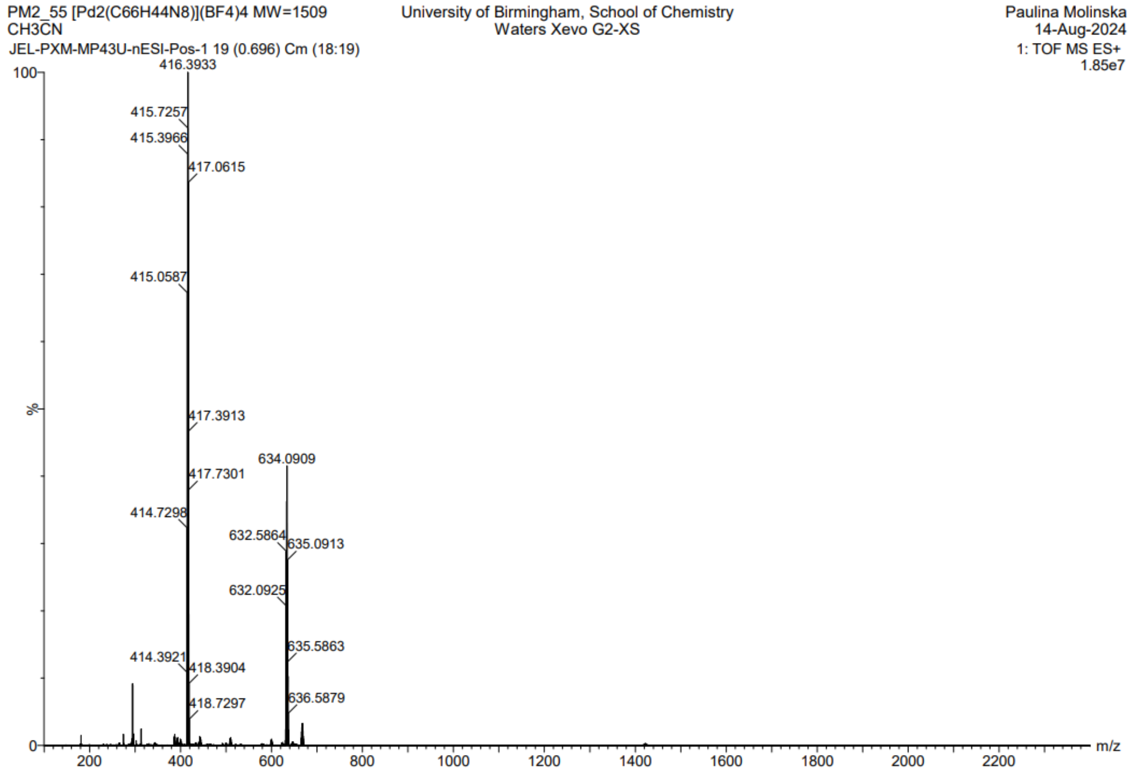

Figure S242 ESI-MS of [$Pd_2(1AA)_2(2AC)_2](BF_4)_4$.

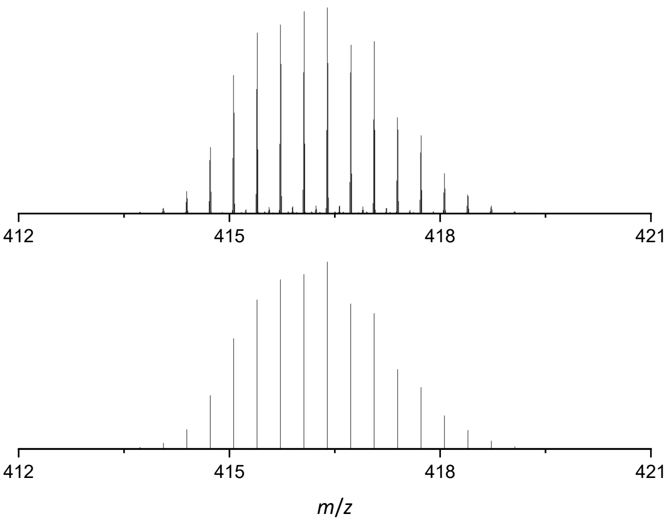

Figure S243 Observed (top) and calculated (bottom) isotopic patterns for {[$Pd_2(1AA)_2(2AC)_2](BF_4)\}^{3+}$.

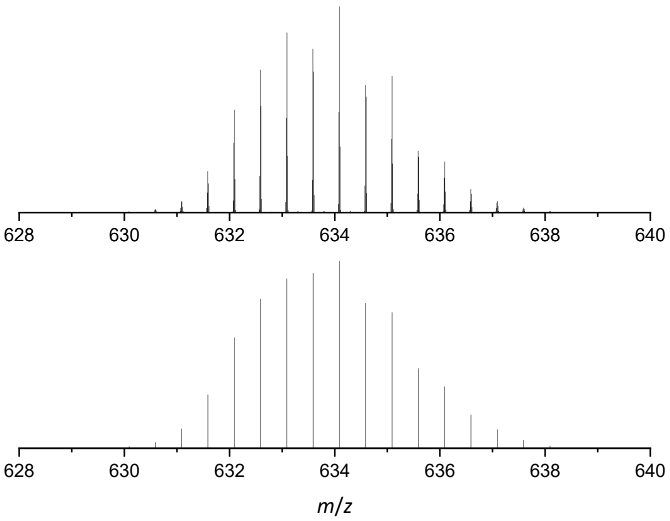

Figure S244 Observed (top) and calculated (bottom) isotopic patterns for {[Pd_2(1AA)$_2$(2AC)$_2$](BF_4)F}$^{2+}$.

Synthesis of $[Pd_2(\mathbf{1AB})_2(\mathbf{2AB})_2](BF_4)_4$

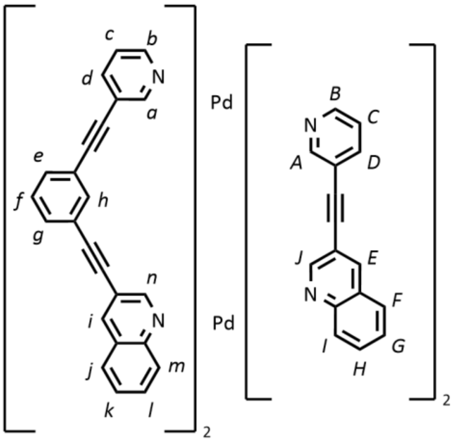

1AB (4.6 mg, 20 µmol, 1 eq.) and **2AB** (6.6 mg, 20 µmol, 1 eq.) were combined in a vial to which a 29.3 mM stock solution of $[Pd(CH_3CN)_4](BF_4)_2$ (750 µL, 22 µmol, 1.1 eq.) in d_6-DMSO was added. A homogenous solution was obtained following sonication which was then transferred to a 5 mm NMR tube before heating at 50 °C for 24 h. The formation of multiple species was observed by ^{1}H NMR.

ESI-MS *m/z* = 383.57 $\{[Pd_2(\mathbf{2AB})_4]\}^{4+}$ calc. 383.57; 473.74 $\{[Pd_2(\mathbf{1AB})_2(\mathbf{2AB})_2](BF_4)\}^{3+}$ calc. 473.74; 540.43 $\{[Pd_2(\mathbf{2AB})_4](BF_4)\}^{3+}$ calc. 540.43; 720.11 $\{[Pd_2(\mathbf{1AB})_2(\mathbf{2AB})_2](BF_4)F\}^{2+}$ calc. 720.11; 820.14 $\{[Pd_2(\mathbf{2AB})_4](BF_4)F\}^{2+}$ calc. 820.14.

^{19}F NMR (376 MHz, d_6-DMSO) δ: -148.10.

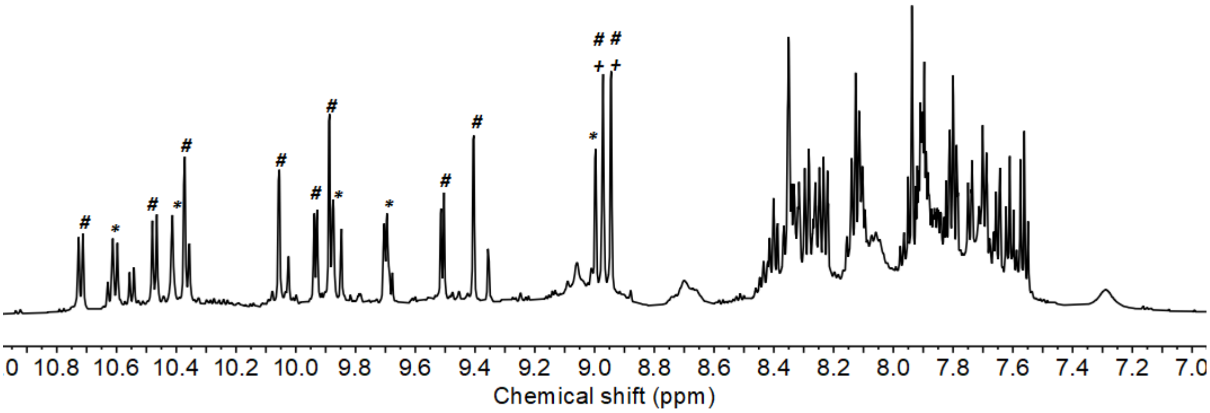

Figure S245 Partial ^{1}H NMR (600 MHz, d_6-DMSO) of equilibrated mixture of 1AB, 2AB and $[Pd(CH_3CN)_4](BF_4)_2$ with labelled signals corresponding to homoleptic cage $[Pd_2(2AB)_4](BF_4)_4$ (*), the major heteroleptic cage isomer (#) and the minor heteroleptic cage isomer (+).

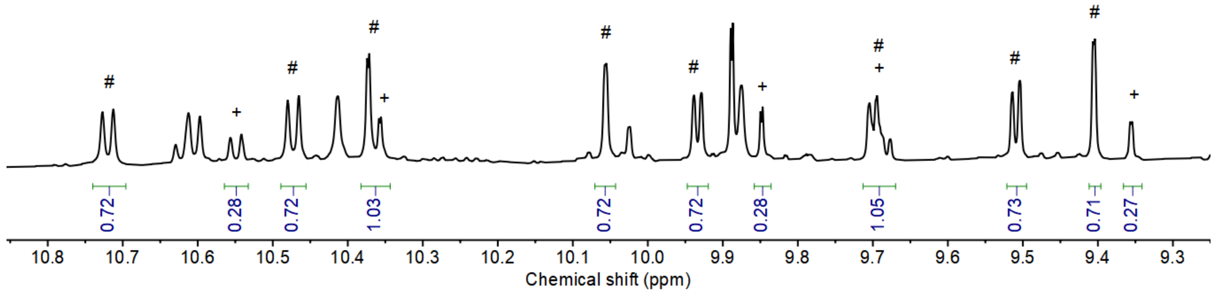

Figure S246 Partial ^{1}H NMR (600 MHz, d_6-DMSO) of equilibrated mixture of 1AB, 2AB and $[Pd(CH_3CN)_4](BF_4)_2$ with integrations of select peaks identified for the major heteroleptic cage isomer (#) and the minor heteroleptic cage isomer (+).

Identification of major isomer V of $[Pd_2(1AB)_2(2AB)_2](BF_4)_4$

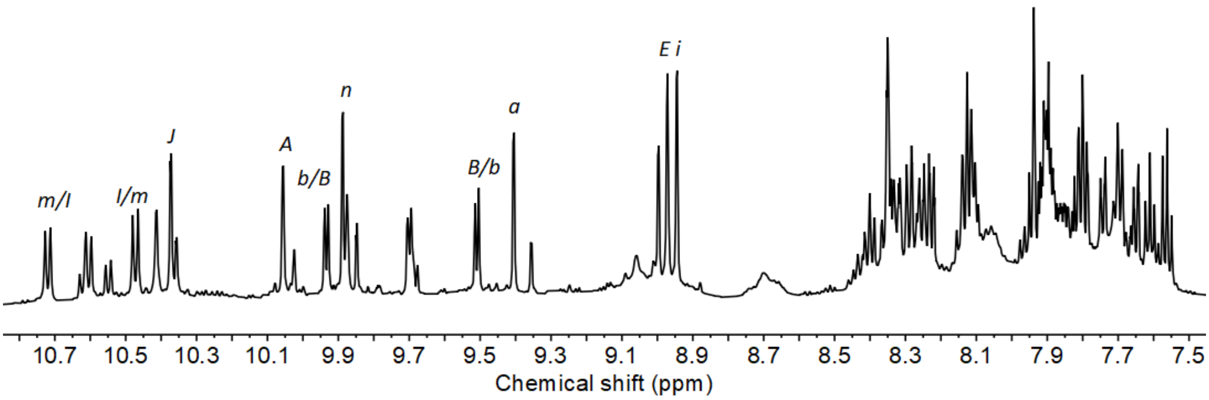

Figure S247 Partial ^{1}H NMR (600 MHz, d_6-DMSO) with peaks of major isomer labelled.

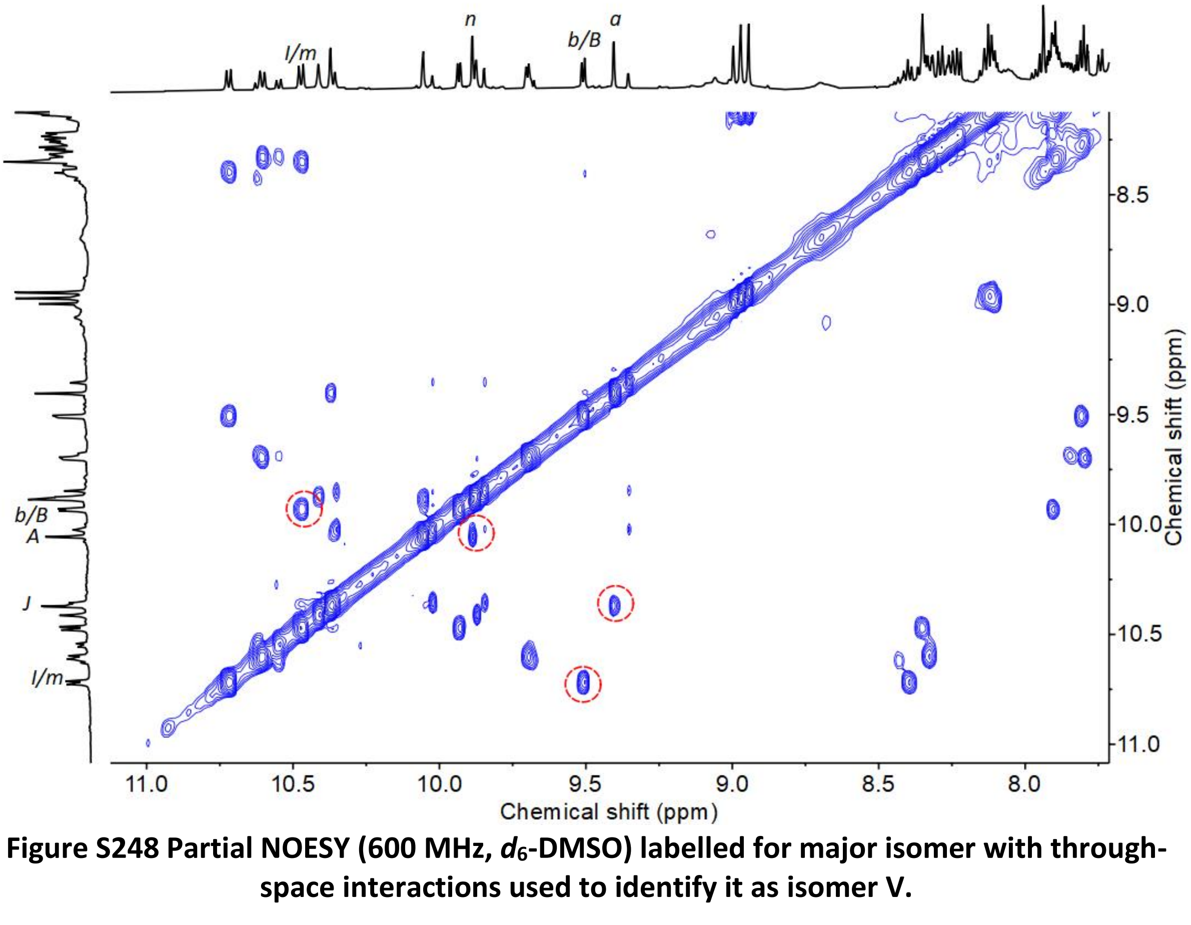

Figure S248 Partial NOESY (600 MHz, d_6-DMSO) labelled for major isomer with through-space interactions used to identify it as isomer V.

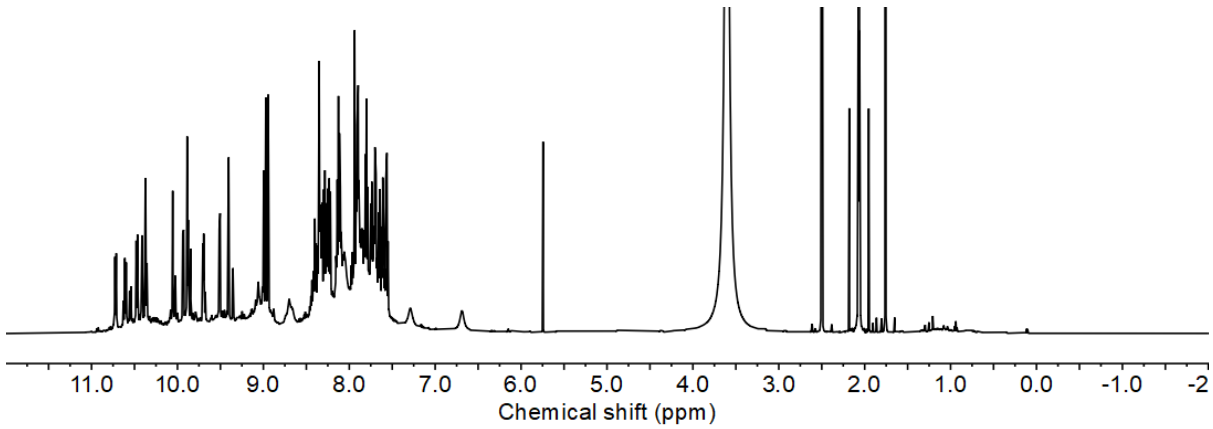

Figure S249 ^{1}H NMR (600 MHz, *d*$_6$-DMSO) of [Pd$_2$(1AB)$_2$(2AB)$_2$](BF$_4$)$_4$.

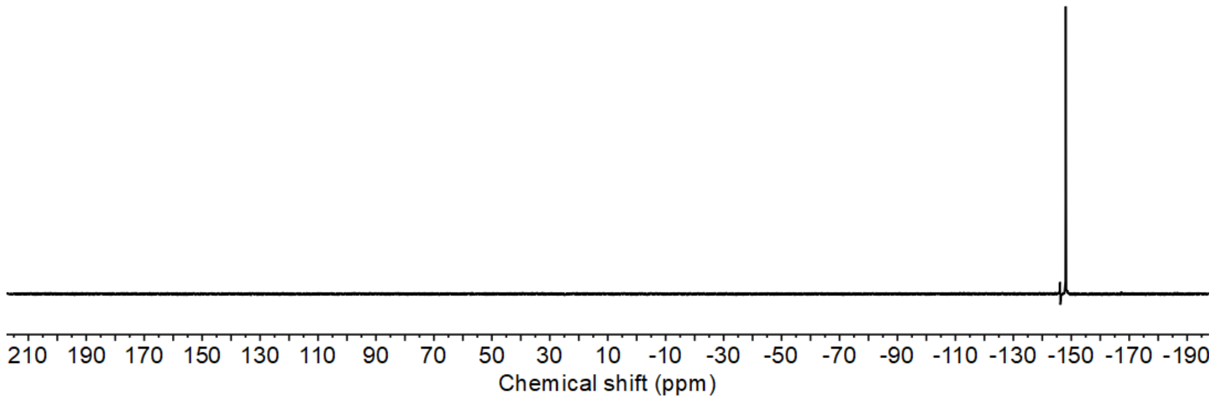

Figure S250 ^{19}F NMR (376 MHz, *d*$_6$-DMSO) of [Pd$_2$(1AB)$_2$(2AB)$_2$](BF$_4$)$_4$.

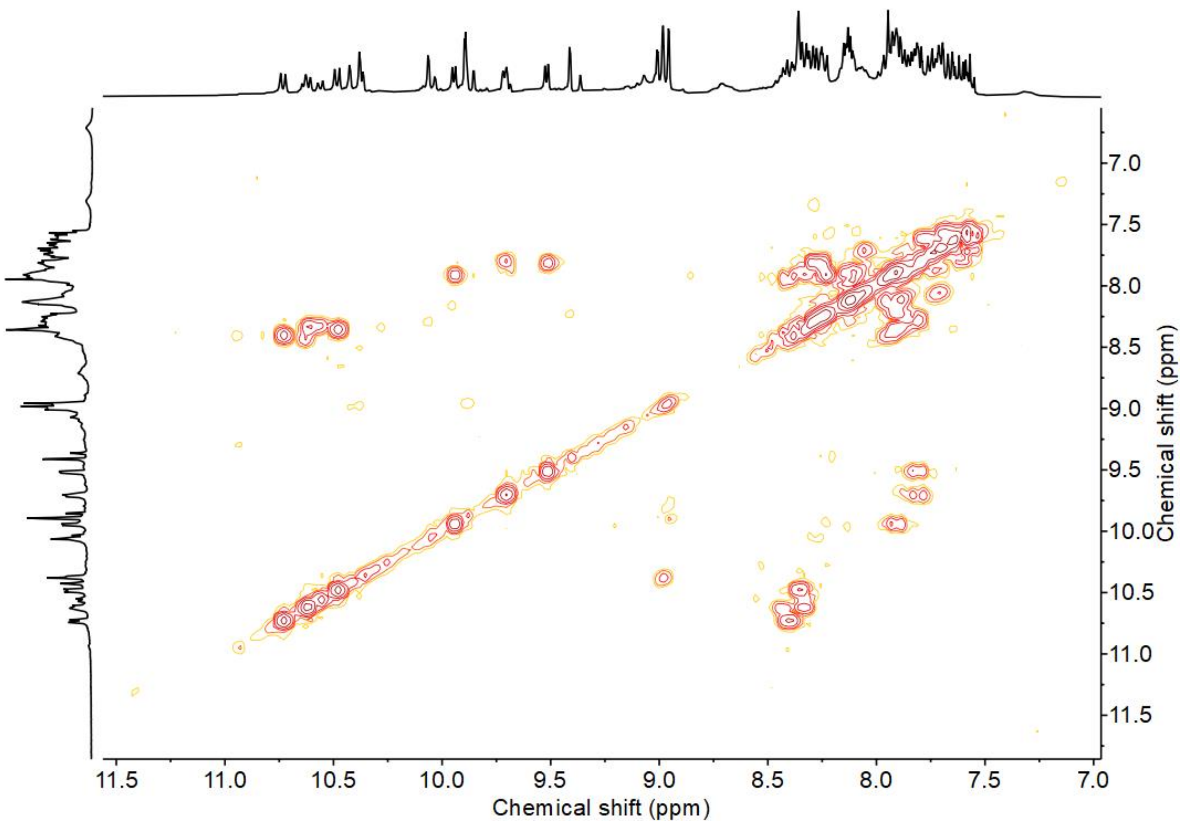

Figure S251 Partial COSY (d_6-DMSO) of $[Pd_2(1AB)_2(2AB)_2](BF_4)_4$.

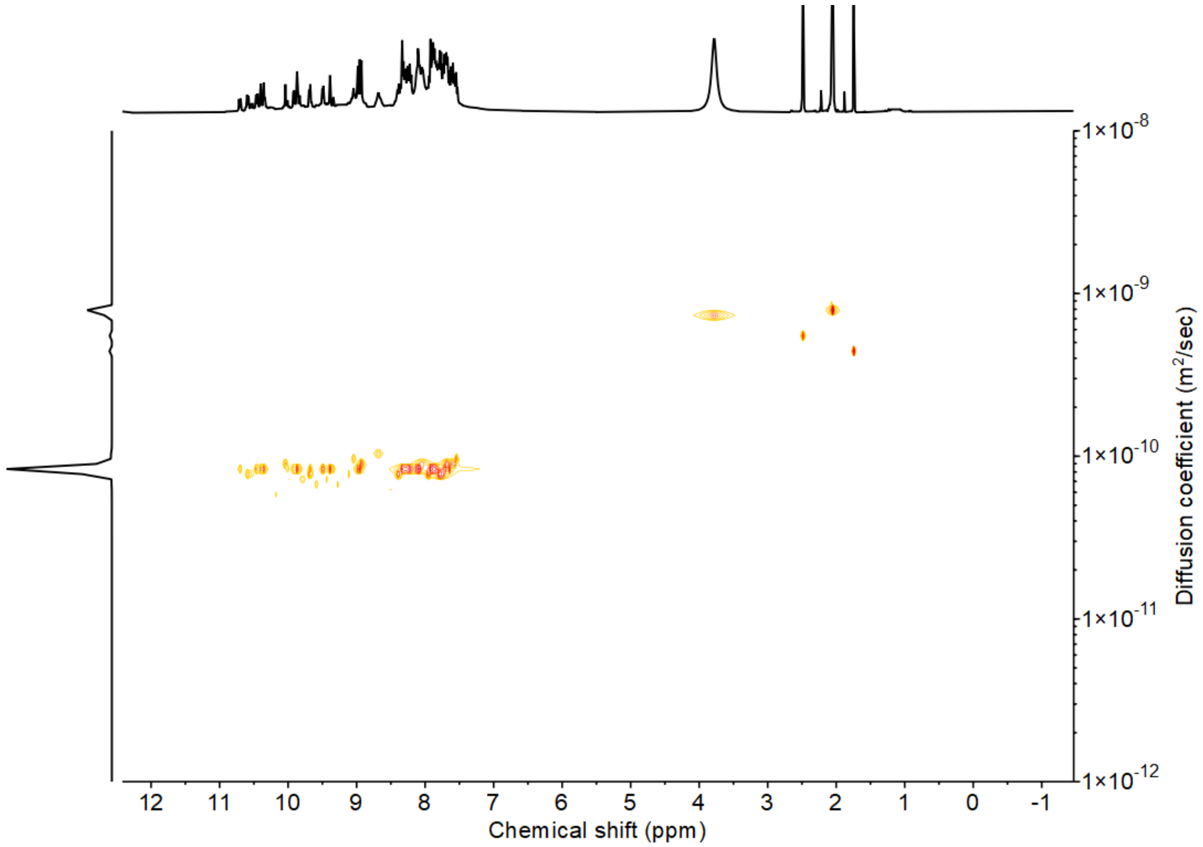

Figure S252 DOSY (d_6-DMSO) of $[Pd_2(1AB)_2(2AB)_2](BF_4)_4$.

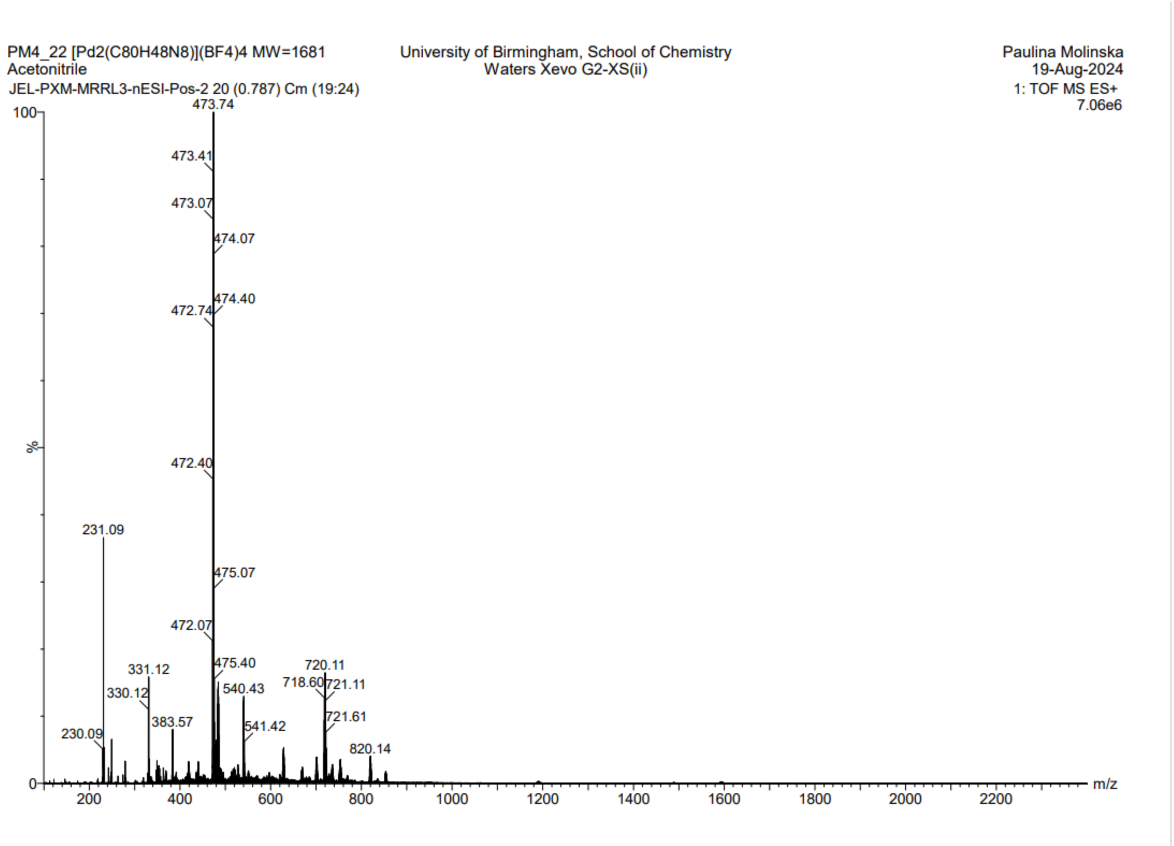

Figure S253 ESI-MS of $[Pd_2(1AB)_2(2AB)_2](BF_4)_4$.

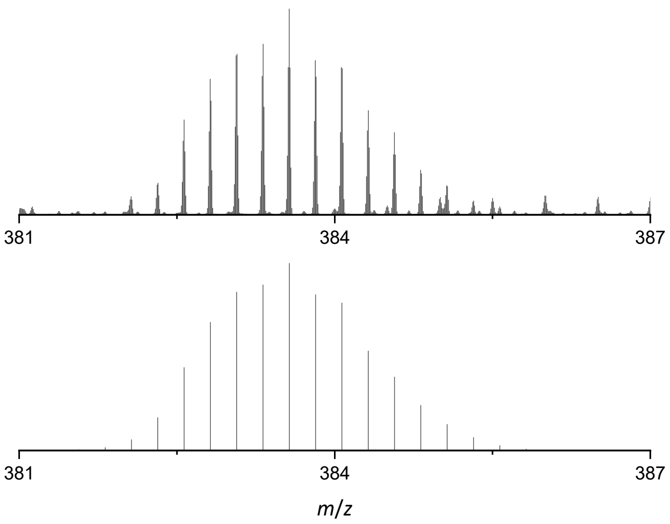

Figure S254 Observed (top) and calculated (bottom) isotopic patterns for Homoleptic $\{[Pd_2(2AB)_4]\}^{4+}$.

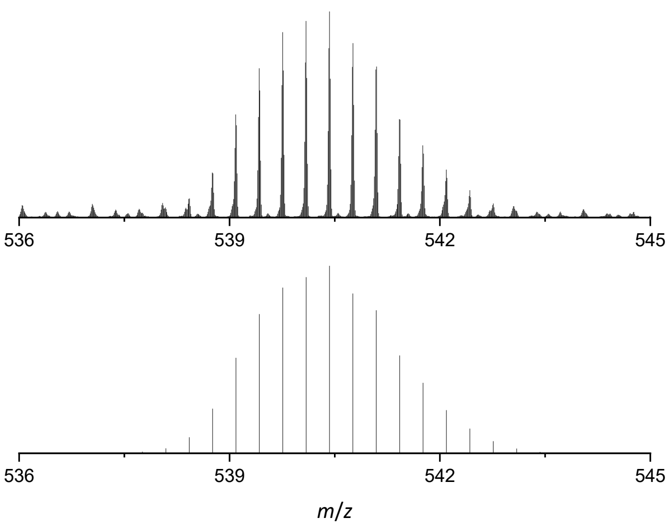

Figure S255 Observed (top) and calculated (bottom) isotopic patterns for Homoleptic $\{[Pd_2(2AB)_4](BF_4)\}^{3+}$.

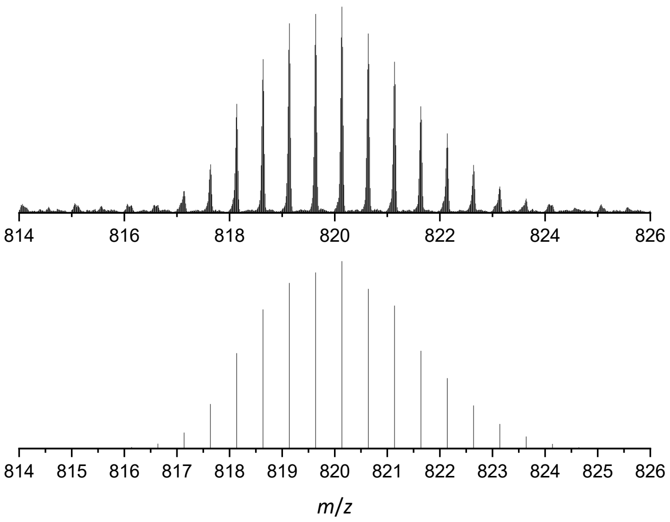

Figure S256 Observed (top) and calculated (bottom) isotopic patterns for Homoleptic $\{[Pd_2(2AB)_4](BF_4)F\}^{2+}$

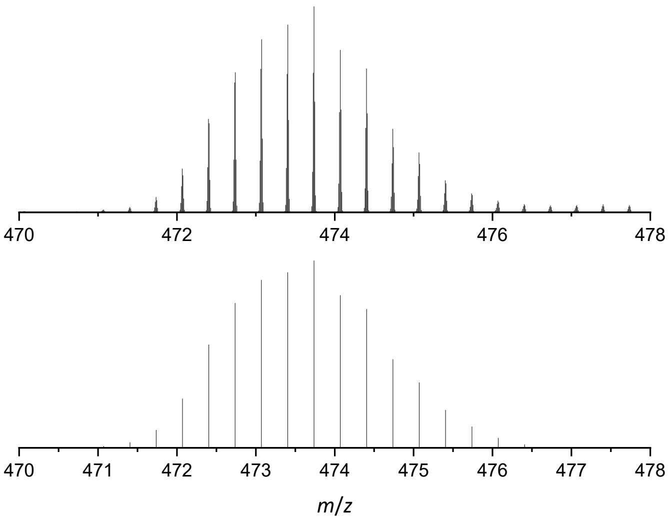

Figure S257 Observed (top) and calculated (bottom) isotopic patterns for Heteroleptic $\{[Pd_2(1AB)_2(2AB)_2](BF_4)\}^{3+}$.

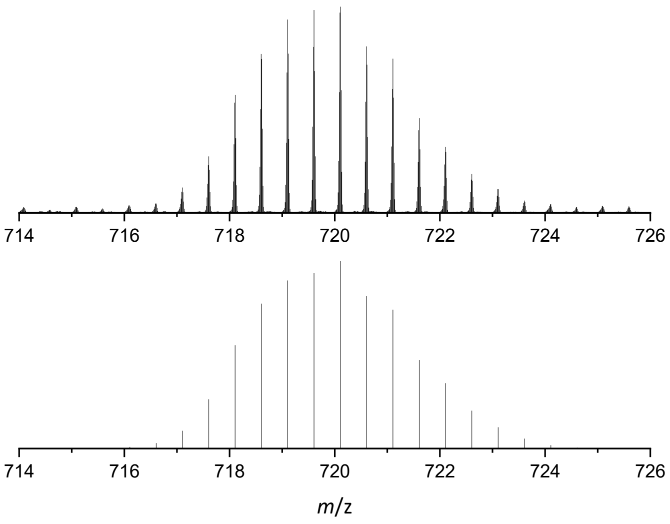

Figure S258 Observed (top) and calculated (bottom) isotopic patterns for Heteroleptic $\{[Pd_2(1AB)_2(2AB)_2](BF_4)F\}^{2+}$.

Synthesis of $[Pd_2(1AC)_2(2AC)_2](BF_4)_4$

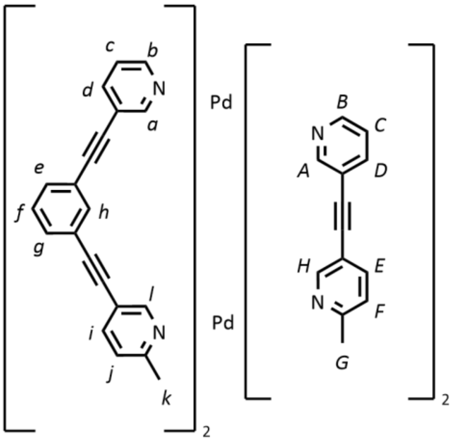

1AC (3.9 mg, 20 µmol, 1 eq.), **2AC** (5.9 mg, 20 µmol, 1 eq.) were combined in a vial to which a 26.7 mM stock solution of $[Pd(CH_3CN)_4](BF_4)_2$ in CD_3CN (750 µL , 20 µmol, 1 eq.) was added. A homogenous solution was obtained following sonication which was then transferred to a 5 mm NMR tube before heating at 70 °C for 24 h.

Only peaks for the major species are reported.

^{1}H NMR (600 MHz, CD_3CN) δ: 9.93 (d, *J* = 1.4 Hz, 2H, H_A), 9.93 (d, *J* = 1.5 Hz, 2H, H_H), 9.41 (d, *J* = 1.8 Hz, 2H, H_a), 9.36 (d, *J* = 1.8 Hz, 2H, H_l), 8.87 (d, 5.7 Hz 2H, H_b), 8.76 (d, *J* = 5.6 Hz, 2H, H_B), 8.27 (dd, *J* = 1.7, 0.6 Hz, 2H, H_h), 8.05 (m, 4H, H_d, H_D), 7.95 (dd, *J* = 8.1, 1.8 Hz, 2H, H_i), 7.93 (dd, *J* = 8.2, 1.9 Hz, 2H, H_E),7.65 (dd, *J* = 7.5, 5.8 Hz, 2H, H_c), 7.61-7.56 (m, 8H, H_C, H_e, H_g, H_j), 7.52-7.48 (m, 4H, H_f, H_F), 3.81 (s, 6H, H_k), 3.56 (s, 6H, H_G).

^{13}C NMR (101 MHz, CD_3CN) δ: 162.81, 160.62, 154.25 (C_a), 154.06 (C_A), 153.78 (C_H), 153.68 (C_l), 152.74 (C_B), 151.78 (C_b), 143.32 (C_d/C_D), 143.08 (C_d/C_D), 143.06 (C_i), 142.42 (C_E), 139.28 (C_h), 132.47 (C_e /C_g), 132.33 (C_e /C_g), 130.76 (C_F), 129.31 (C_f), 129.08 (C_j), 129.00 (C_c, C_C), 125.Z47, 124.52, 123.26, 122.98, 122.48, 121.42, 95.79, 94.75, 90.74, 89.76, 85.86, 85.74, 26.56 (C_k, C_G).

^{19}F NMR (376 MHz, CD_3CN) δ: -151.51, -148.82.

^{1}H DOSY (400 MHz, CD_3CN) *D*: 6.4 × 10^{-10} m^2s^{-1}; R_S: 9.6 Å.

ESI-MS *m/z* = 425.73 of $\{[Pd_2(\mathbf{1AC})_2(\mathbf{2AC})_2](BF_4)\}^{3+}$ calc. 475.74; 648.10 of $\{[Pd_2(\mathbf{1AC})_2(\mathbf{2AC})_2](BF_4)F\}^{2+}$ calc. 648.11.

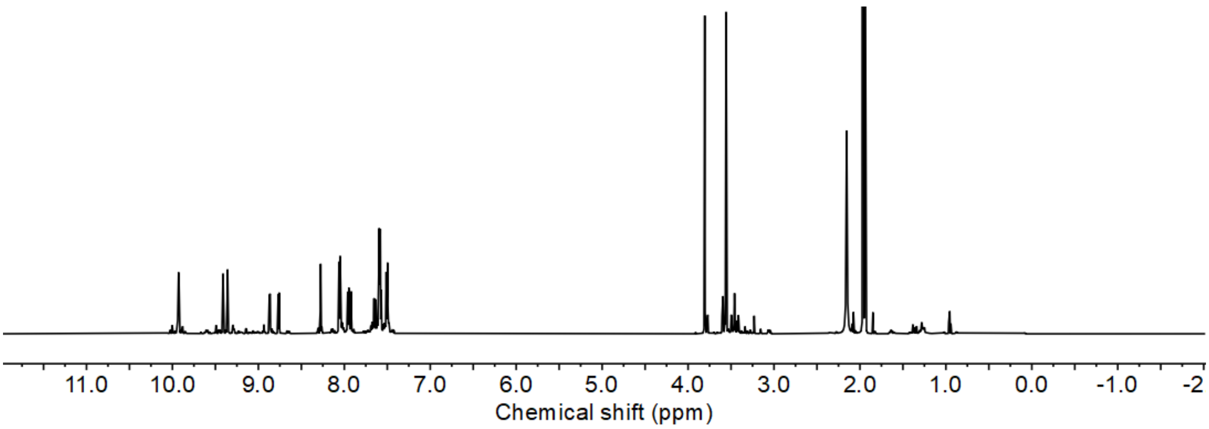

Figure S259 ^{1}H NMR (600 MHz, CD_3CN) of $[Pd_2(1AC)_2(2AC)_2](BF_4)_4$.

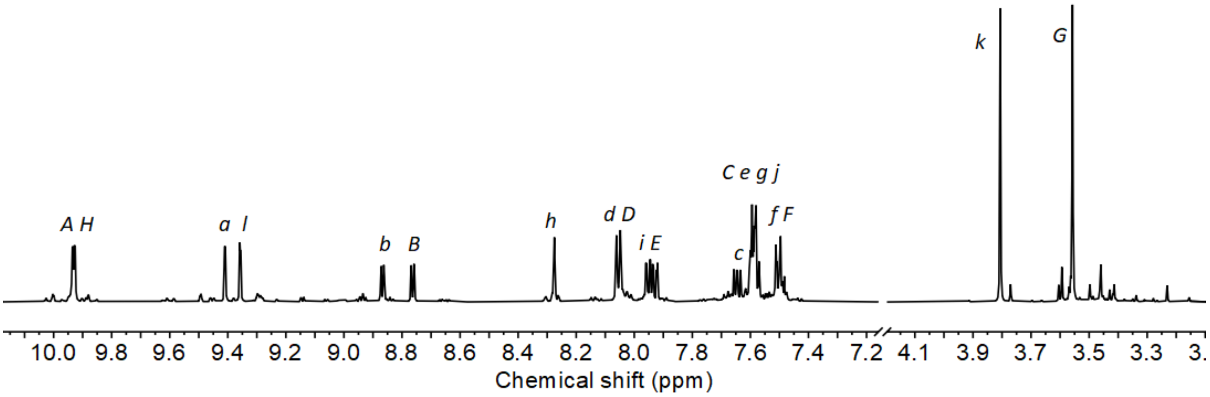

Figure S260 Partial ^{1}H NMR (600 MHz, CD_3CN) of $[Pd_2(1AC)_2(2AC)_2](BF_4)_4$.

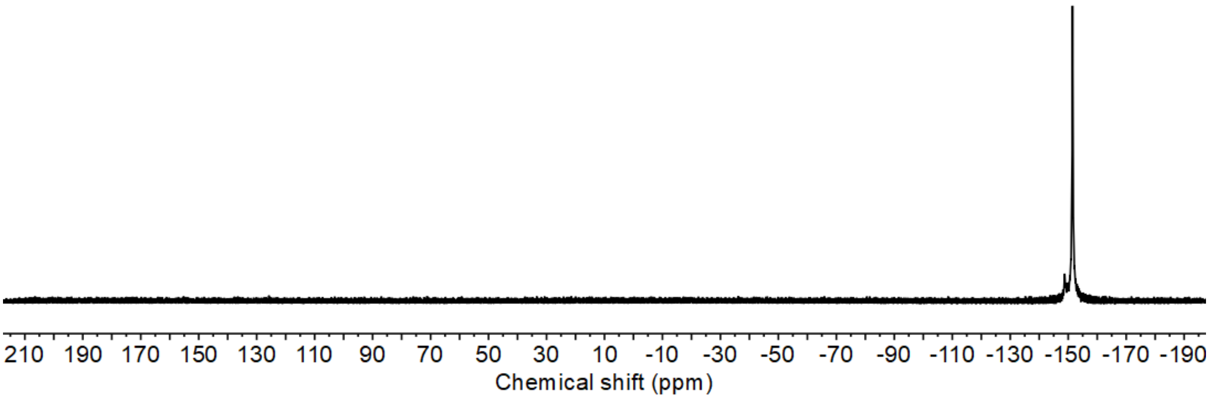

Figure S261 ^{19}F NMR (376 MHz, CD_3CN) of $[Pd_2(1AC)_2(2AC)_2](BF_4)_4$.

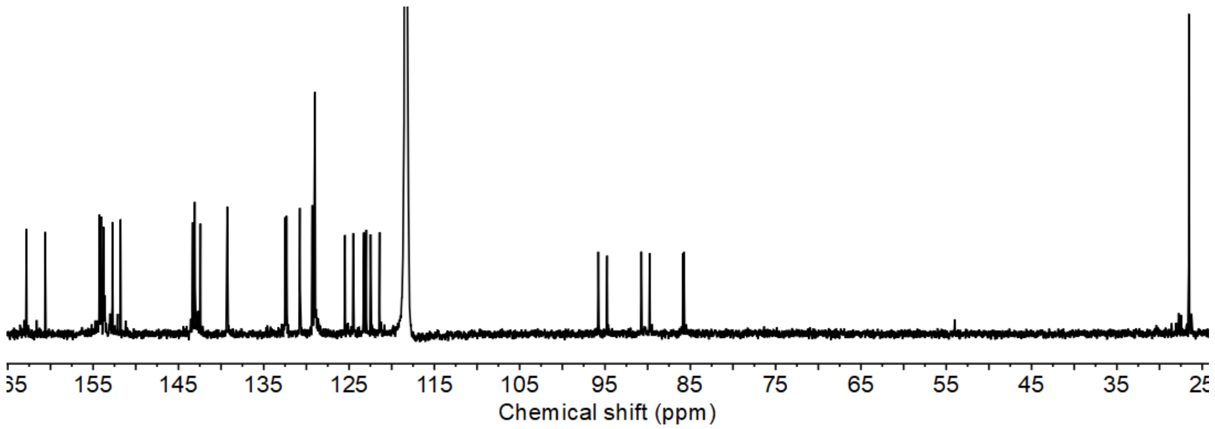

Figure S262 Partial ^{13}C NMR (101 MHz, CD_3CN) of [$Pd_2(1AC)_2(2AC)_2$](BF_4)$_4$.

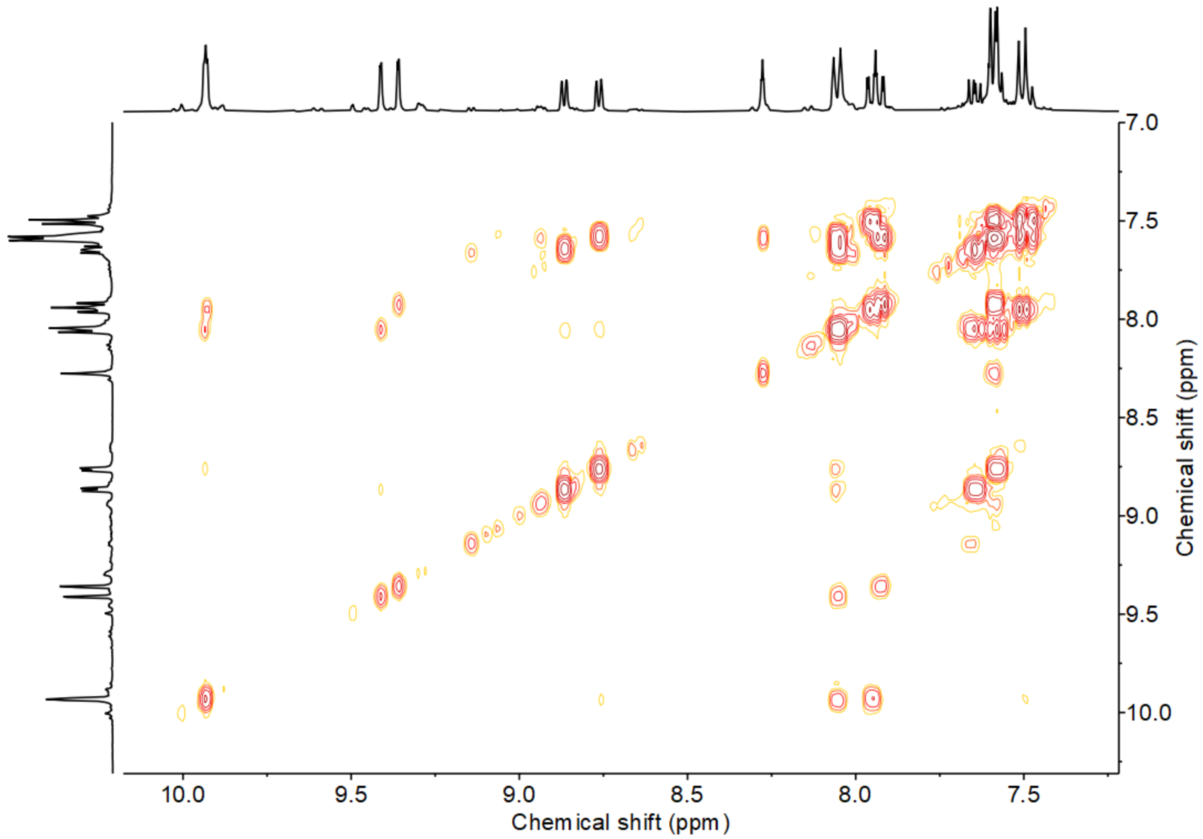

Figure S263 Partial COSY (CD_3CN) of [$Pd_2(1AC)_2(2AC)_2$](BF_4)$_4$.

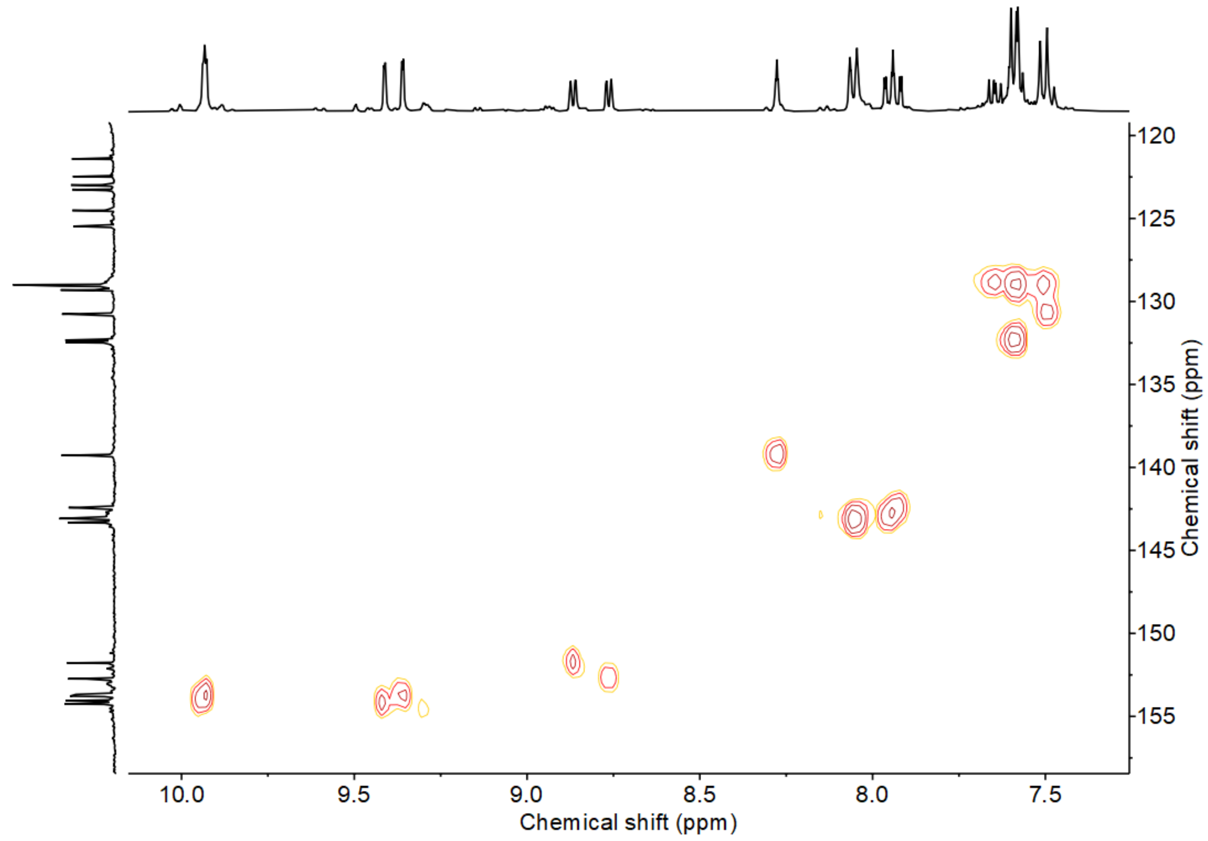

Figure S264 Partial HSQC (CD_3CN) of $[Pd_2(1AC)_2(2AC)_2](BF_4)_4$.

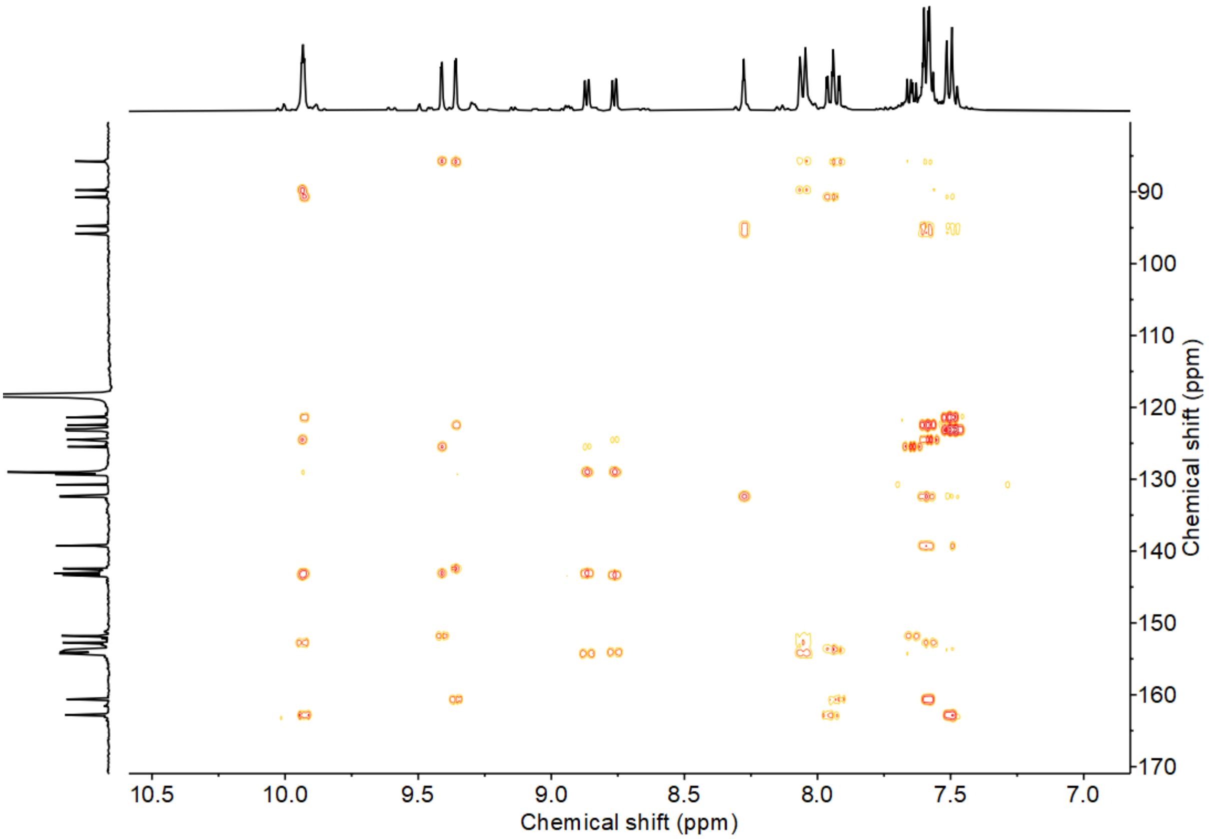

Figure S265 Partial HMBC (CD_3CN) of $[Pd_2(1AC)_2(2AC)_2](BF_4)_4$.

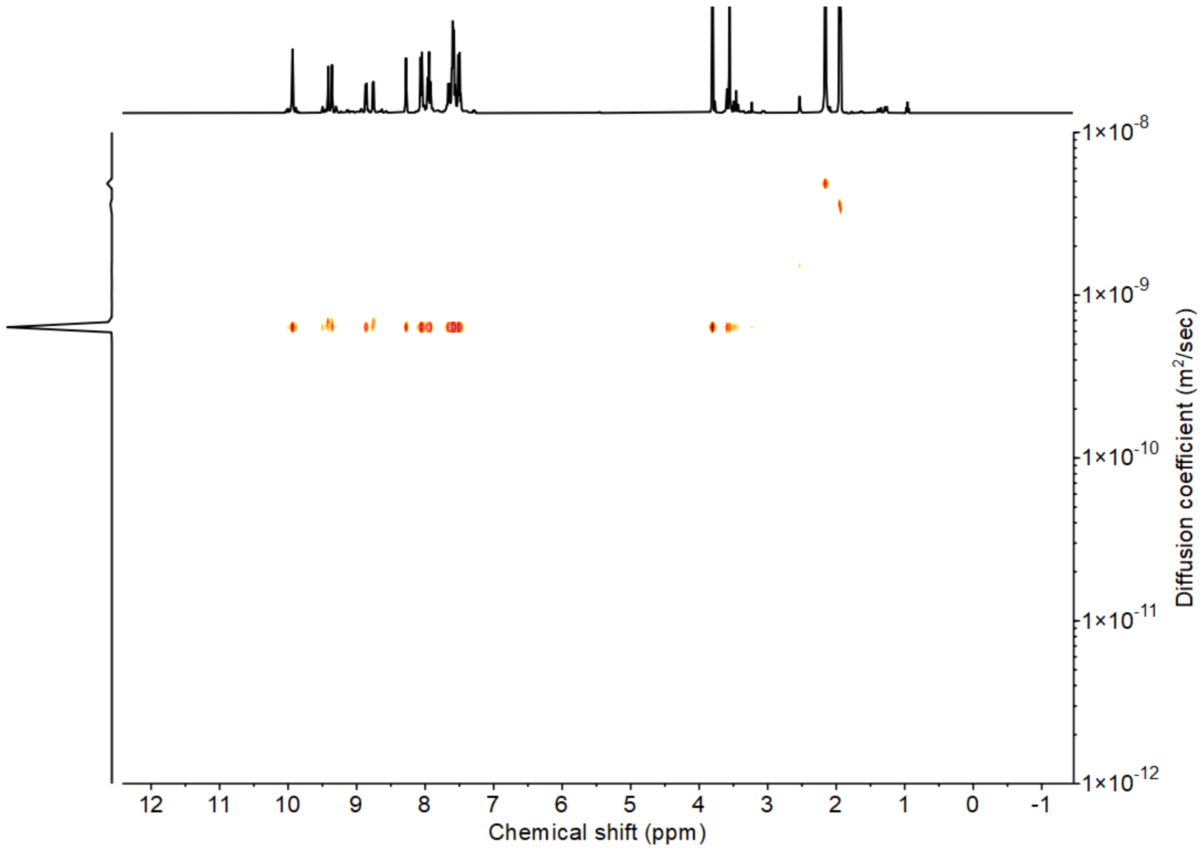

Figure S266 DOSY (CD_3CN) of $[Pd_2(1AC)_2(2AC)_2](BF_4)_4$.

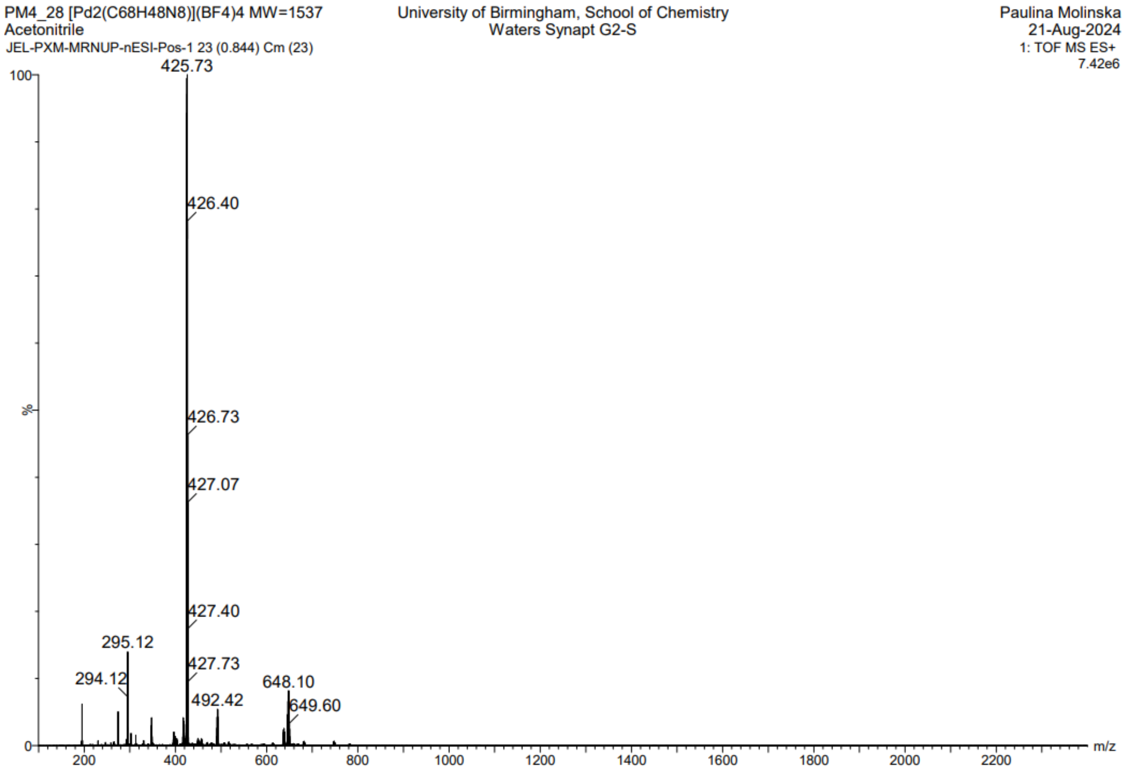

Figure S267 ESI-MS of $[Pd_2(1AC)_2(2AC)_2](BF_4)_4$.

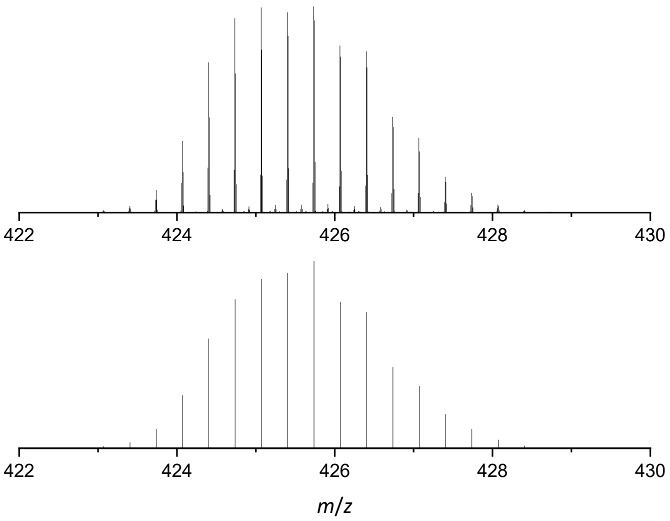

Figure S268 Observed (top) and calculated (bottom) isotopic patterns for $\{[Pd_2(1AC)_2(2AC)_2](BF_4)\}^{3+}$.

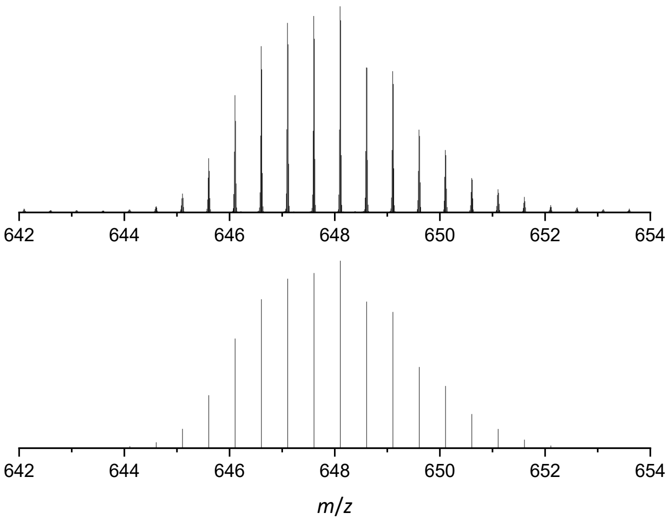

Figure S269 Observed (top) and calculated (bottom) isotopic patterns for $\{[Pd_2(1AC)_2(2AC)_2](BF_4)F\}^{2+}$.

Synthesis of $[Pd_2(1AC)_2(2AB)_2](BF_4)_4$

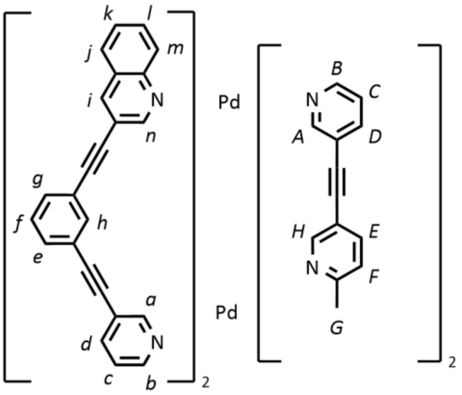

2AB (9.9 mg, 30 µmol, 1 eq.), **1AC** (5.8 mg, 30 µmol, 1 eq.) were combined in a vial to which a 40 mM stock solution of $[Pd(CH_3CN)_4](BF_4)_2$ (750 µL , 30 µmol, 1 eq.) in CD_3CN was added. A homogenous solution was obtained following sonication which was then transferred to a 5 mm NMR tube. The reaction was heated at 70 °C for 19 h.

Only peaks for the major species are reported.

^{1}H NMR (600 MHz, CD_3CN) δ: 10.08 (dd, J = 8.3, 1.0 Hz, 2H, H_m), 10.06-10.05 (m, 4H, H_H, H_A), 9.70 (d, J = 1.8 Hz, 2H, H_n), 9.49 (d, J = 1.8 Hz, 2H, H_a), 8.86 (ddd, J = 5.9, 1.3, 0.6 Hz, 2H, H_B/H_b), 8.78 (ddd, J = 5.9, 1.3, 0.6 Hz, 2H, H_B/H_b), 8.65 (m, 2H, H_i), 8.57 (m, 2H, H_l), 8.34 (dd, J = 1.7, 0.6 Hz, 2H, H_h), 8.17 (m, 2H, H_j), 8.06-7.96 (m, 8H, H_k, H_E, H_d, H_D), 7.63 (ddd, J = 7.7, 1.7, 1.1 Hz, 2H, $H_{g/e}$), 7.60 (ddd, J = 7.7, 1.7, 1.1 Hz, 2H, $H_{g/e}$), 7.56 (d, J = 8.2 Hz, 2H, H_F), 7.51 (m, 2H, H_f), 7.46-7.43 (m, 4H, H_c, H_C), 3.73 (s, 6H, H_G).

^{13}C NMR (101 MHz, CD_3CN) δ: 163.06, 157.23 (C_n), 154.38 (C_a), 154.07, 153.61, 152.45 (C_b/C_B), 151.55 (C_b/C_B), 144.57, 143.20, 143.06, 142.85, 139.01 (C_h), 136.75 (C_l), 132.46, 132.37, 131.50 (C_j), 131.22, 130.69, 130.65, 130.32, 129.04, 128.67, 128.48, 125.88 (C_m), 125.27, 124.44, 123.16, 122.93, 121.38, 120.24, 95.61, 94.72, 90.67, 89.69, 86.40, 85.66, 26.83 (C_G).

^{19}F NMR (376 MHz, CD_3CN) δ: -151.13.

^{1}H DOSY (400 MHz, CD_3CN) D: 6.8×10^{-10} m^2s^{-1}; R_S: 9.0 Å.

ESI-MS m/z = 449.74 $\{[Pd_2(\mathbf{2AB})_2(\mathbf{1AC})_2](BF_4)\}^{3+}$ calc. 449.74.

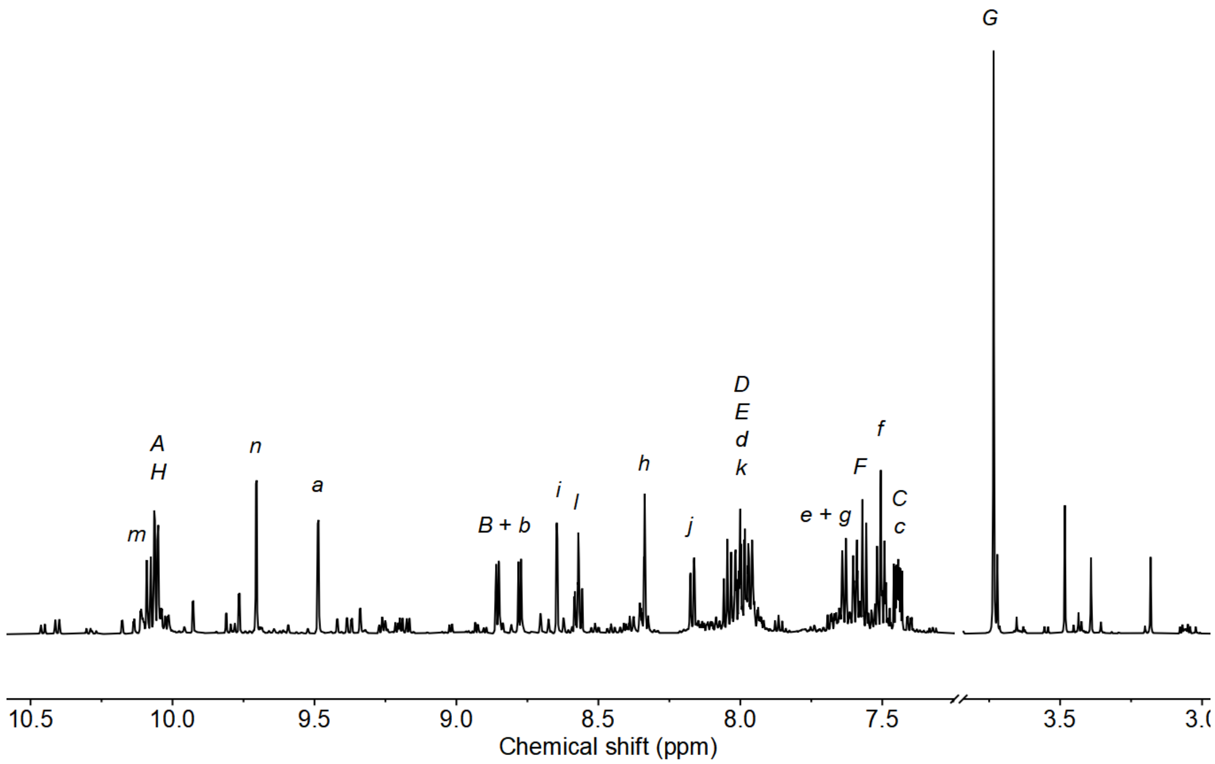

Figure S270 Partial ^{1}H NMR (600 MHz, CD_3CN) of $[Pd_2(1AC)_2(2AB)_2](BF_4)_4$.

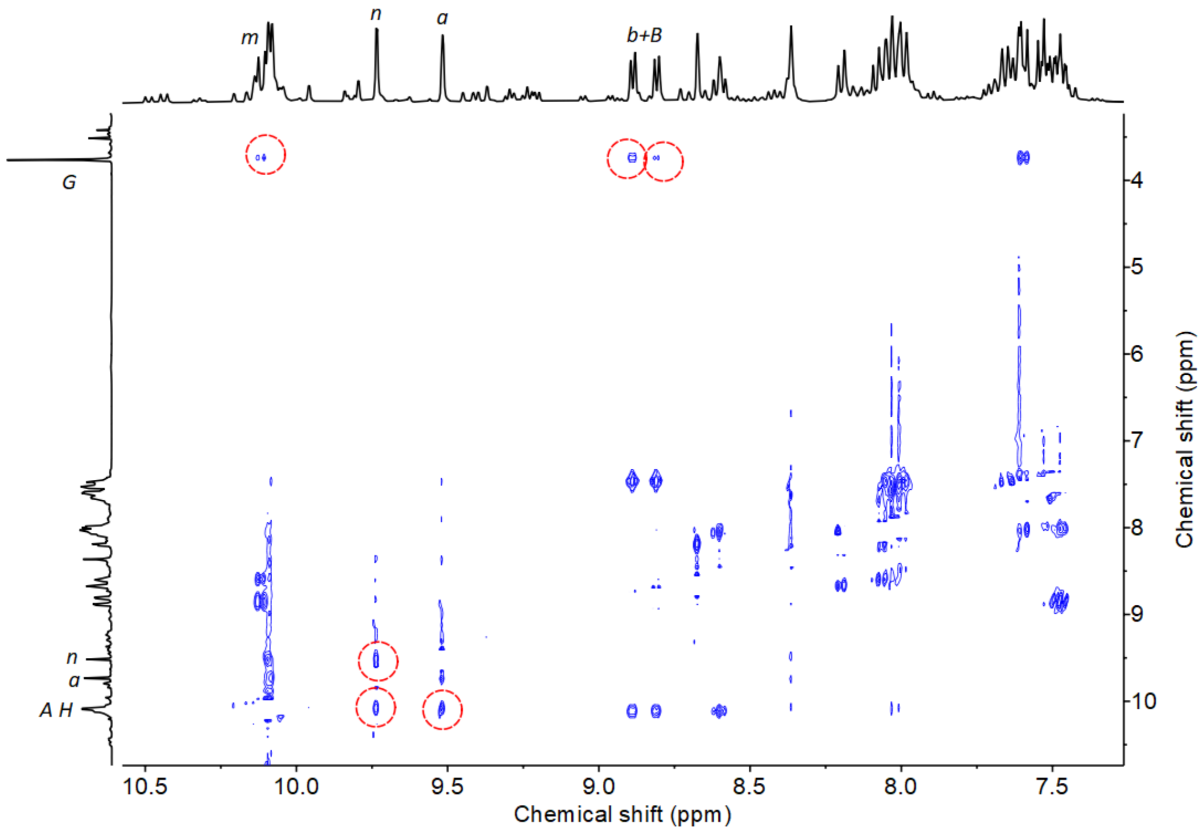

Figure S271 Partial NOESY (400 MHz, CD_3CN) of $[Pd_2(1AC)_2(2AB)_2](BF_4)_4$ with peaks assigned to isomer VI.

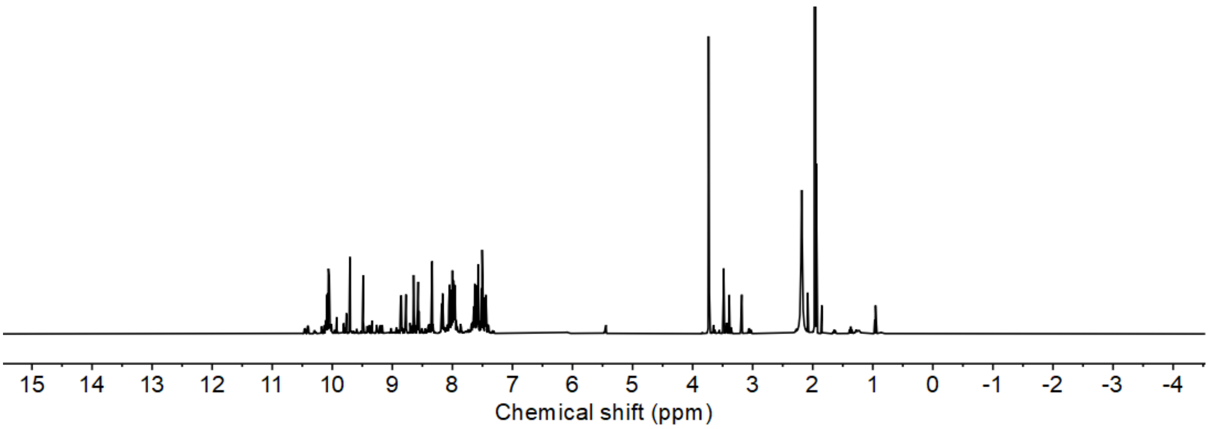

Figure S272 ^{1}H NMR (600 MHz, CD_3CN) of $[Pd_2(1AC)_2(2AB)_2](BF_4)_4$.

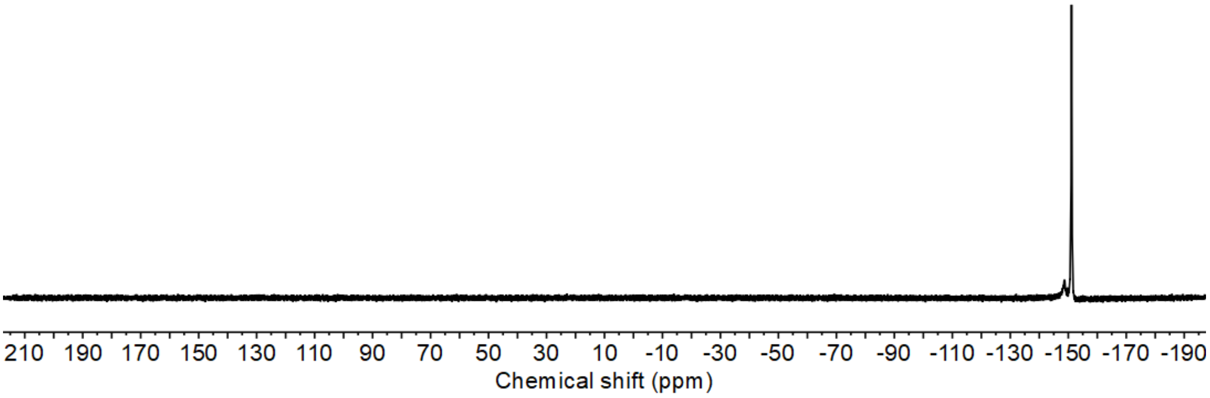

Figure S273 ^{19}F NMR (376 MHz, CD_3CN) of $[Pd_2(1AC)_2(2AB)_2](BF_4)_4$.

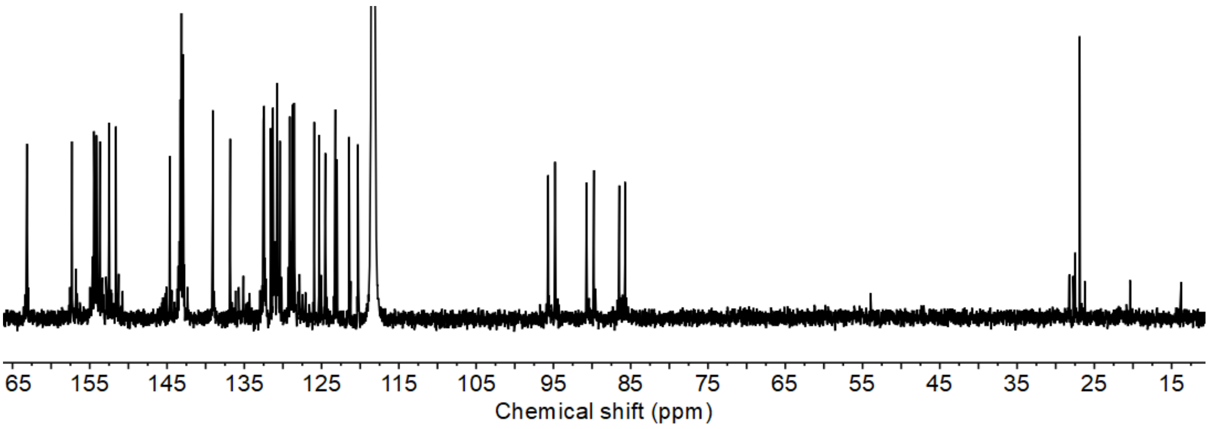

Figure S274 Partial ^{13}C NMR (101 MHz, CD_3CN) of $[Pd_2(1AC)_2(2AB)_2](BF_4)_4$.

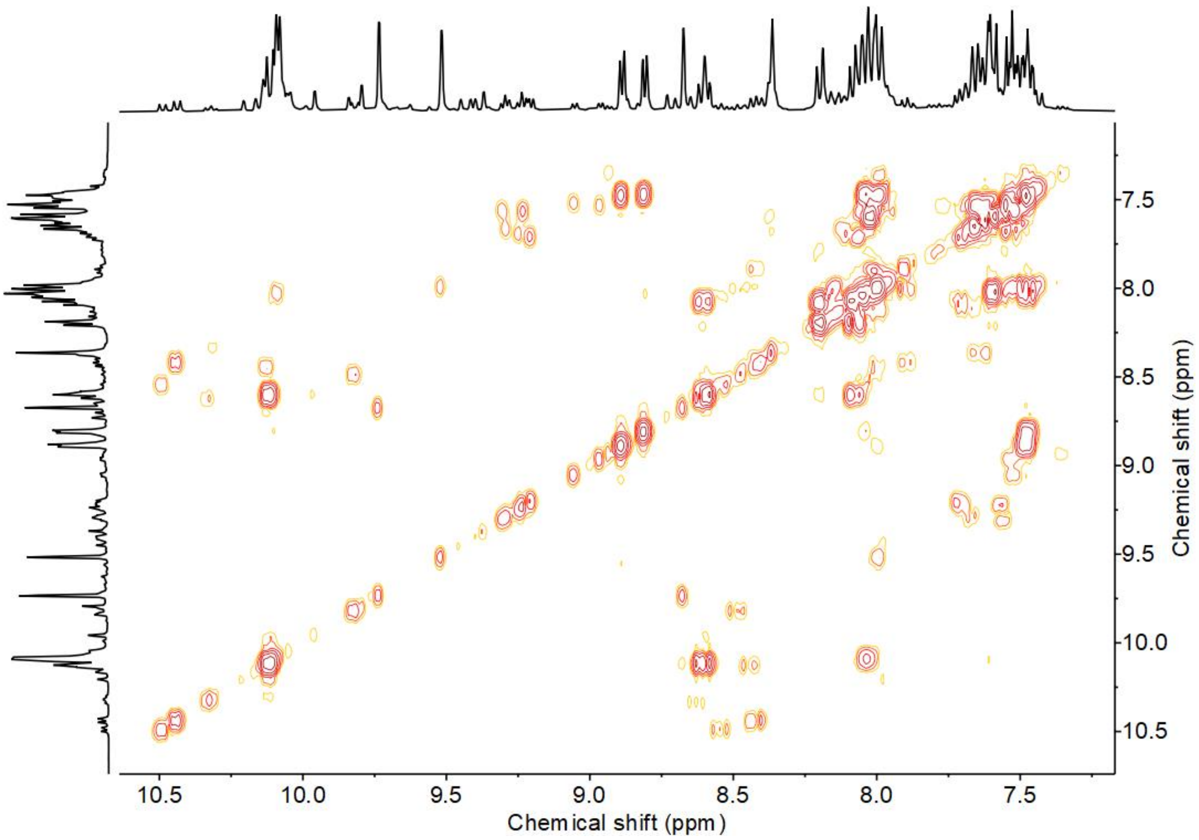

Figure S275 Partial COSY (CD_3CN) of $[Pd_2(1AC)_2(2AB)_2](BF_4)_4$.

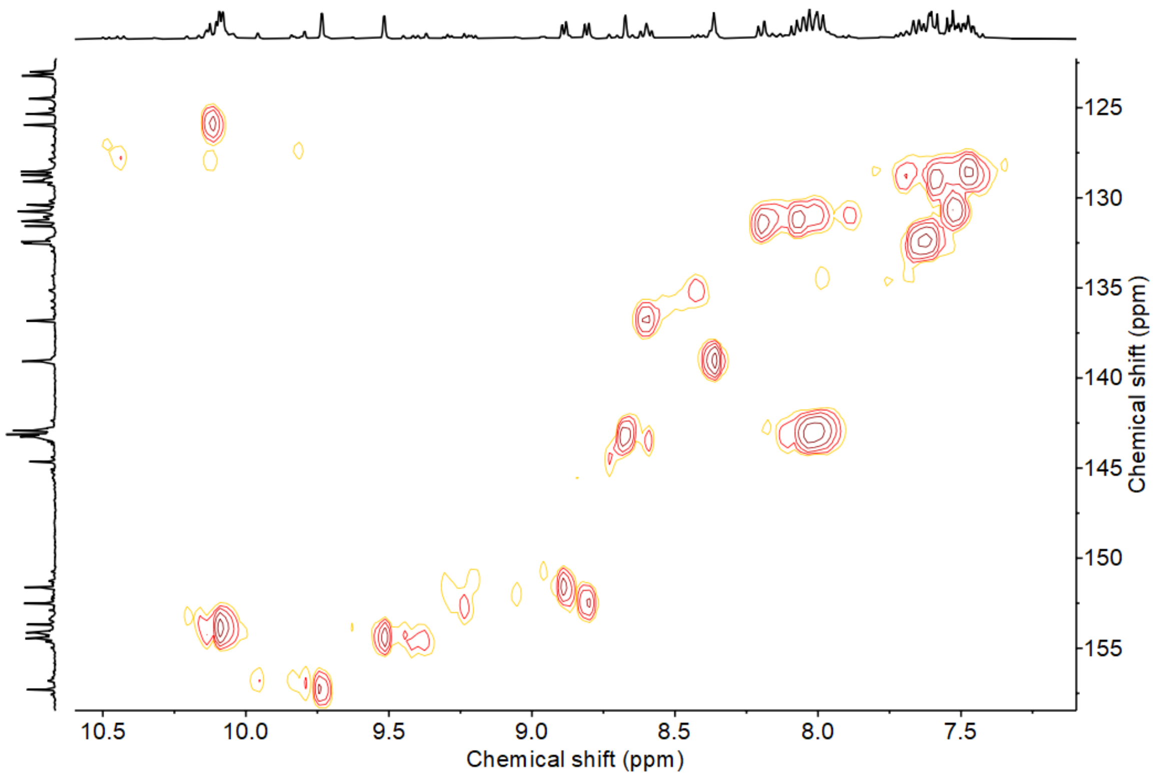

Figure S276 Partial HSQC (CD_3CN) of $[Pd_2(1AC)_2(2AB)_2](BF_4)_4$.

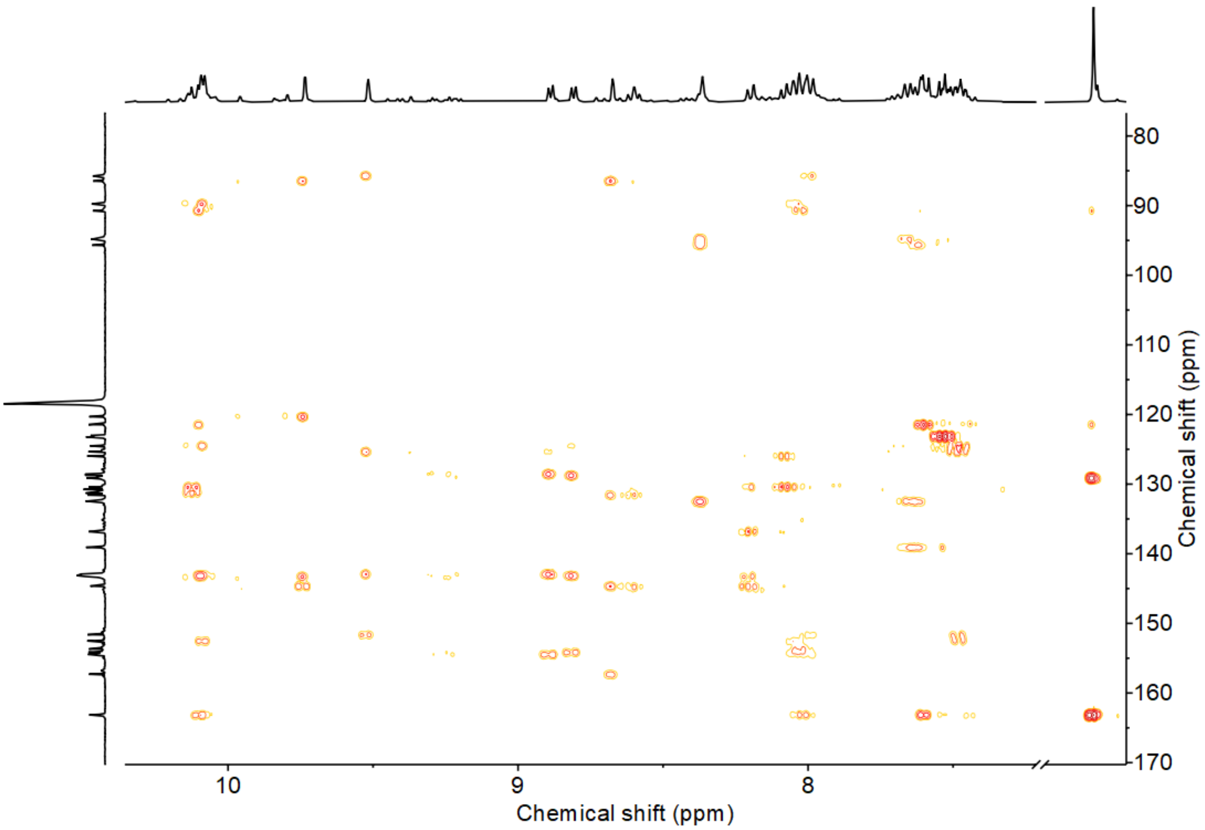

Figure S277 Partial HMBC (CD_3CN) of $[Pd_2(1AC)_2(2AB)_2](BF_4)_4$.

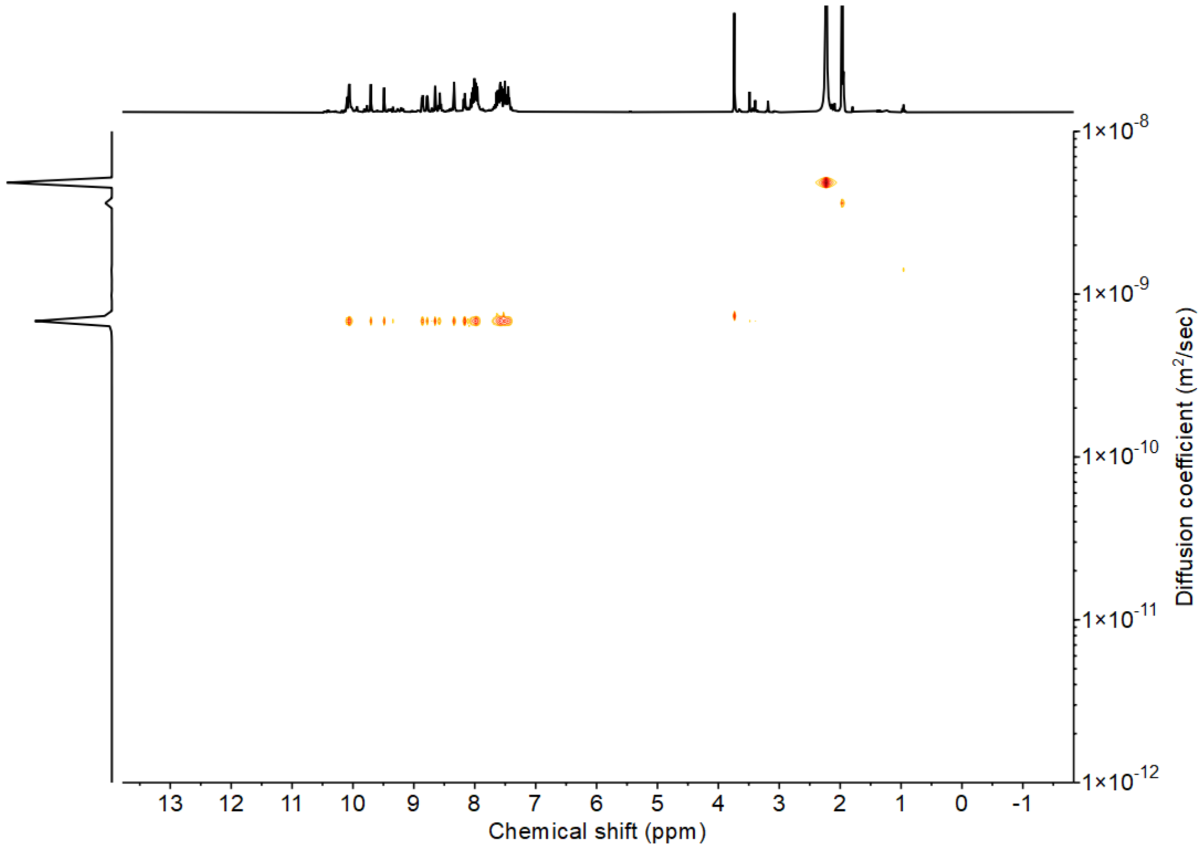

Figure S278 DOSY (CD_3CN) of $[Pd_2(1AC)_2(2AB)_2](BF_4)_4$.

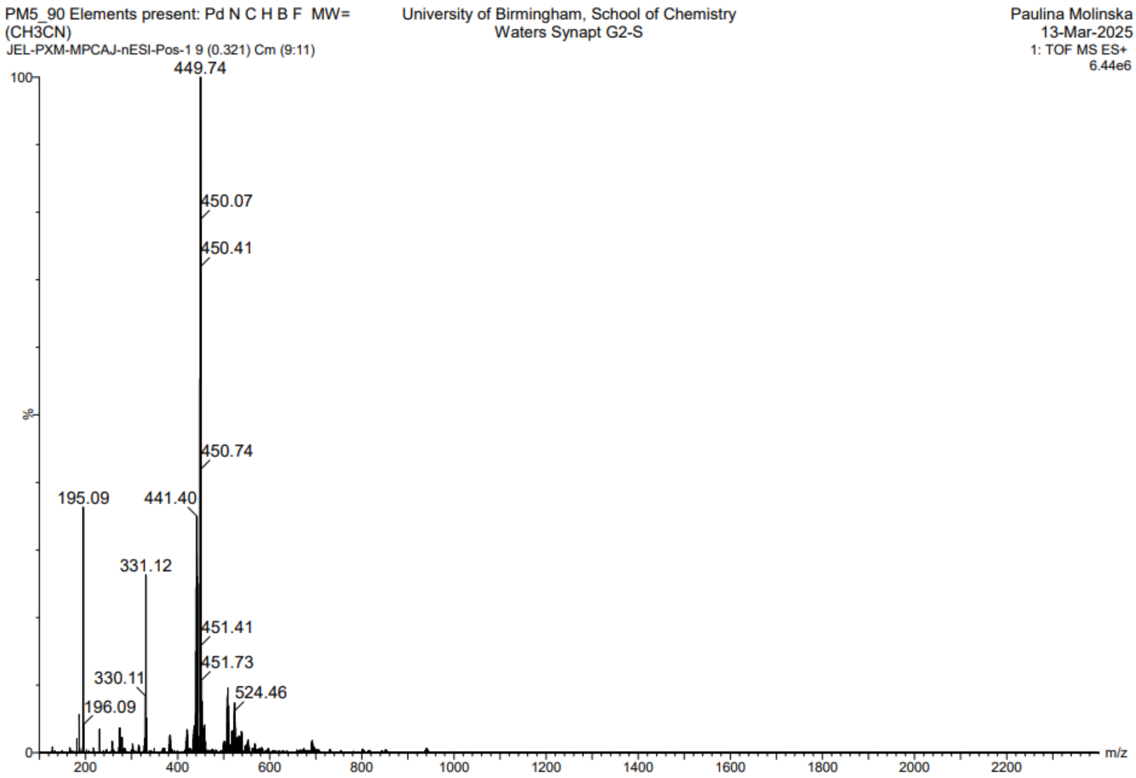

Figure S279 ESI-MS of $[Pd_2(1AC)_2(2AB)_2](BF_4)_4$.

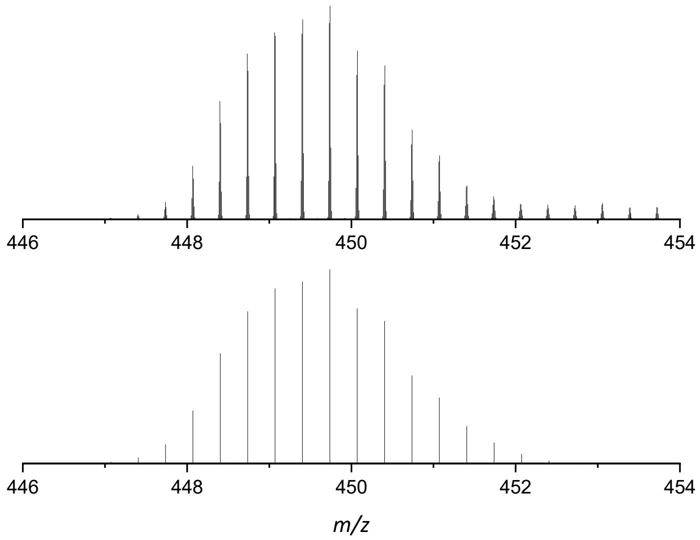

Figure S280 Observed (top) and calculated (bottom) isotopic patterns for $\{[Pd_2(1AC)_2(2AB)_2](BF_4)\}^{3+}$.

Synthesis of $[Pd_2(1AB)_2(2AC)_2](BF_4)_4$

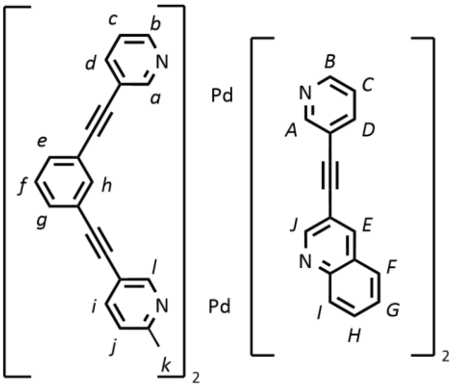

2AC (8.8 mg, 30 µmol, 1 eq.), **1AB** (6.9 mg, 30 µmol, 1 eq.) were combined in a vial to which a 40 mM stock solution of $[Pd(CH_3CN)_4](BF_4)_2$ (750 µL , 30 µmol, 1 eq.) in CD_3CN was added. A homogenous solution was obtained following sonication which was then transferred to a 5 mm NMR tube. The reaction was heated at 70 °C for 19 h.

Only peaks for the major species are reported.

^{1}H NMR (600 MHz, CD_3CN) δ: 10.30 (d, J = 1.8 Hz, 2H, H_J), 10.09 (d, J = 1.9 Hz, 2H, H_A), 9.93 (dd, J = 8.6, 0.9 Hz, 2H, H_I), 9.54 (dd, J = 1.9, 0.6 Hz, 2H, H_l), 9.46 (d, J = 1.8 Hz, 2H, H_a), 8.97 (ddd, J = 5.9, 1.3, 0.6 Hz, 2H, H_b), 8.86 (ddd, J = 5.8, 1.3, 0.6 Hz, 2H, H_B), 8.68 (m, 2H, H_E), 8.46 (ddd, J = 8.6, 7.1, 1.4 Hz, 2H, H_H), 8.30 (td, J = 1.7, 0.6 Hz, 2H, H_h), 8.11 (m, 2H, H_F), 8.05 (m, 2H, H_d), 7.99-7.94 (m, 6H, H_i, H_D, H_G), 7.64 (m, 2H, H_j), 7.61-7.55 (m, 4H, H_e, H_g), 7.53-7.48 (m, 6H, H_c, H_C, H_f), 3.96 (s, 6H, H_k).

^{13}C NMR (101 MHz, CD_3CN) δ: 160.96, 156.28 (C_J), 154.38 (C_a), 153.95 (C_A), 153.61 (C_l), 152.64 (C_B), 151.54 (C_b), 146.06, 144.41 (C_E), 143.20, 142.72, 142.52, 139.26 (C_h), 136.69 (C_H), 132.37, 132.06, 131.29, 131.18, 130.66, 130.63, 130.13, 129.23, 128.62, 128.56, 126.29, 125.20, 124.48, 123.20, 122.85, 122.43, 95.71, 94.59, 91.20, 89.78, 85.82, 85.75, 26.87 (C_k).

^{19}F NMR (376 MHz, CD_3CN) δ: -148.52, -151.19.

^{1}H DOSY (400 MHz, CD_3CN) D: 6.4 × 10^{-10} m^2s^{-1}; R_S: 9.6 Å.

ESI-MS m/z = 449.74 {[Pd_2(**1AB**)$_2$(**2AC**)$_2$](BF_4)}$^{3+}$ calc. 449.74.

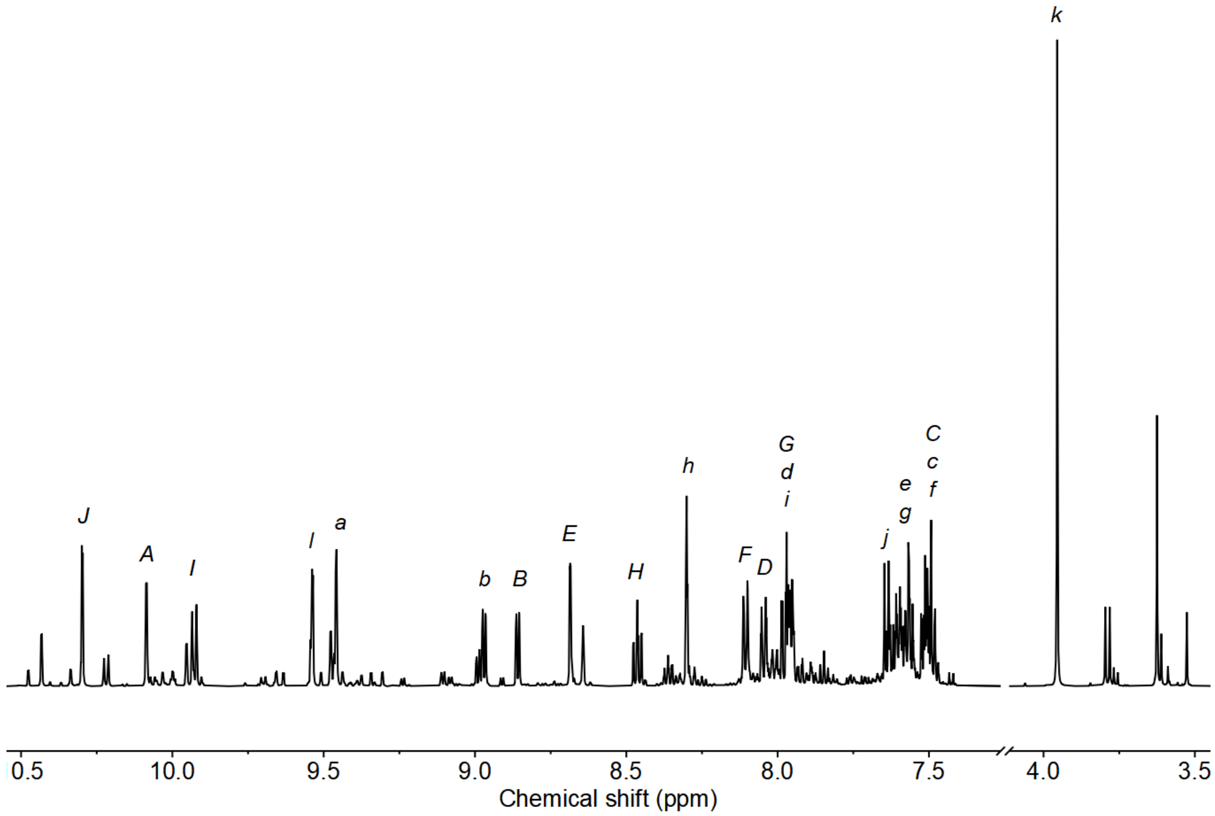

Figure S281 Partial ^{1}H NMR (600 MHz, CD_3CN) of $[Pd_2(1AB)_2(2AC)_2](BF_4)_4$ with peaks of major *VI*-isomer labelled.

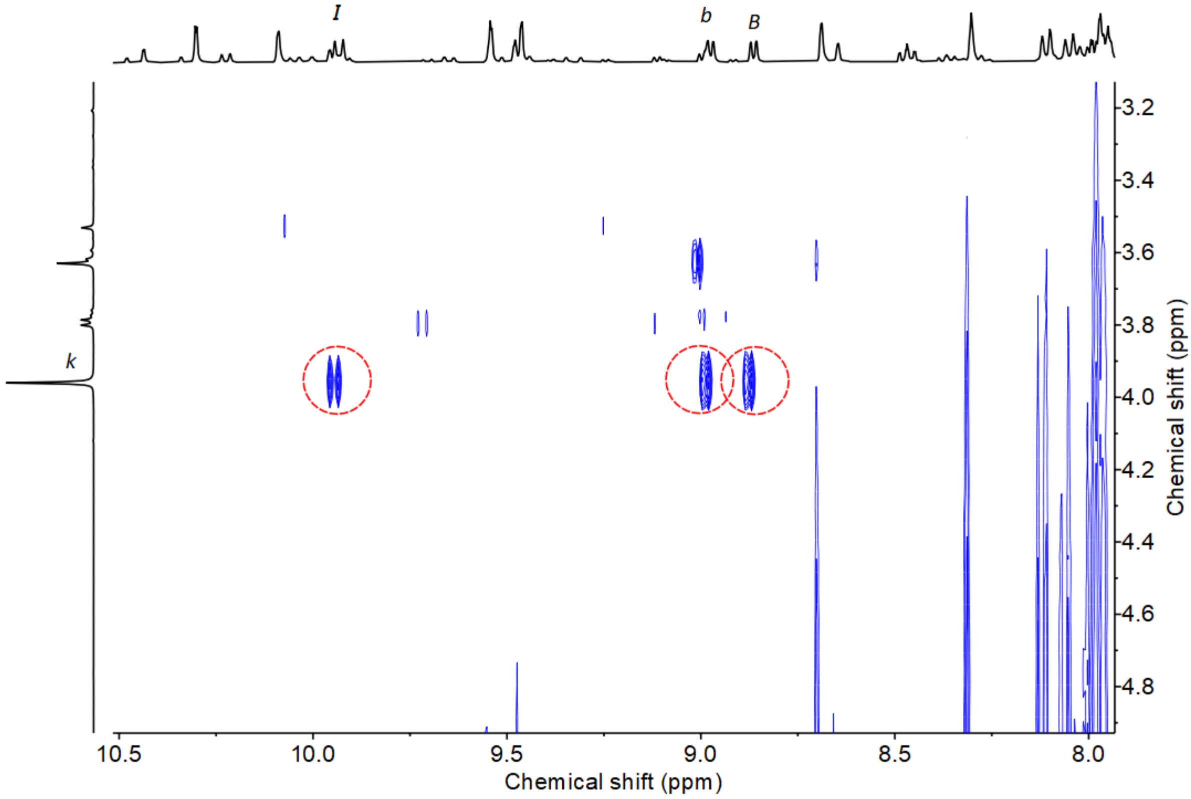

Figure S282 Partial NOESY Partial NOESY (400 MHz, CD_3CN) of $[Pd_2(1AB)_2(2AC)_2](BF_4)_4$ labelled for major isomer with through-space interactions used to identify it as the *VI*-isomer.

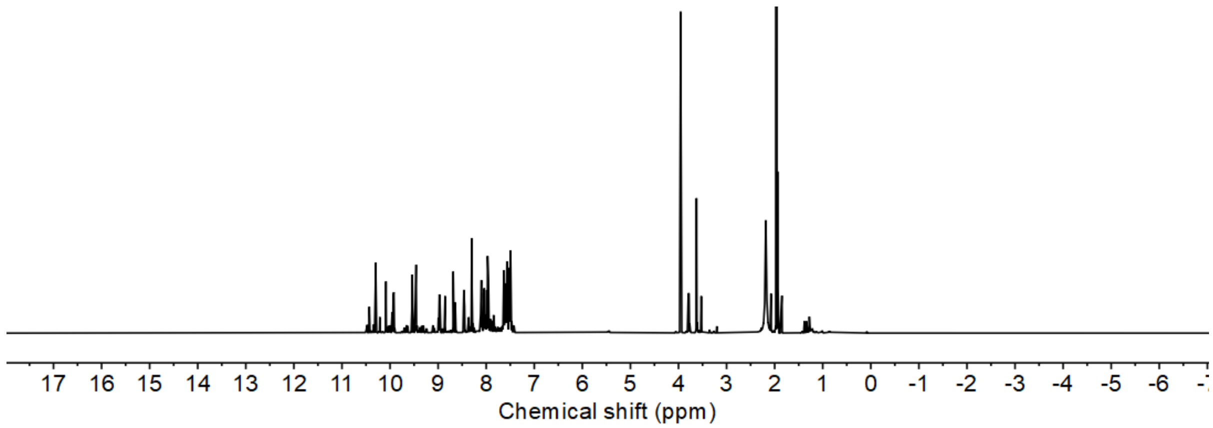

Figure S283 ^{1}H NMR (600 MHz, CD_3CN) of $[Pd_2(1AB)_2(2AC)_2](BF_4)_4$.

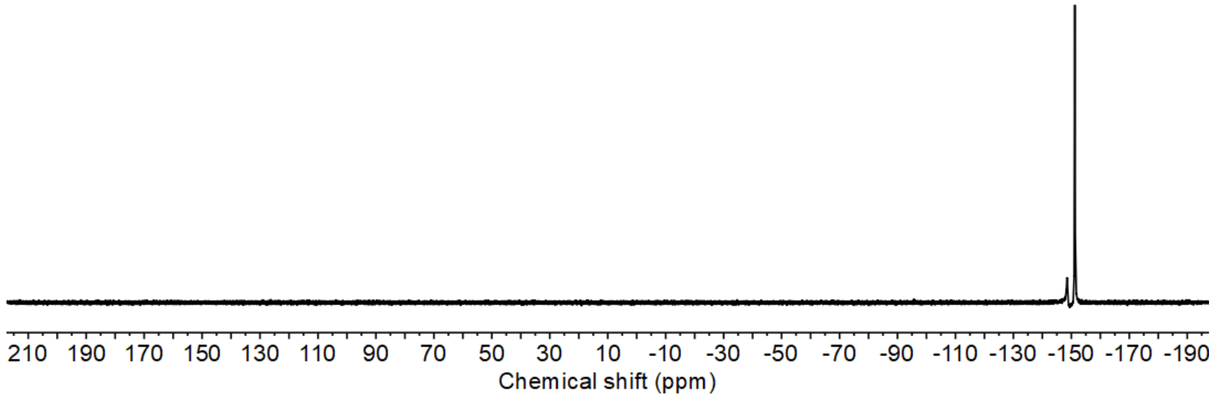

Figure S284 ^{19}F NMR (376 MHz, CD_3CN) of $[Pd_2(1AB)_2(2AC)_2](BF_4)_4$.

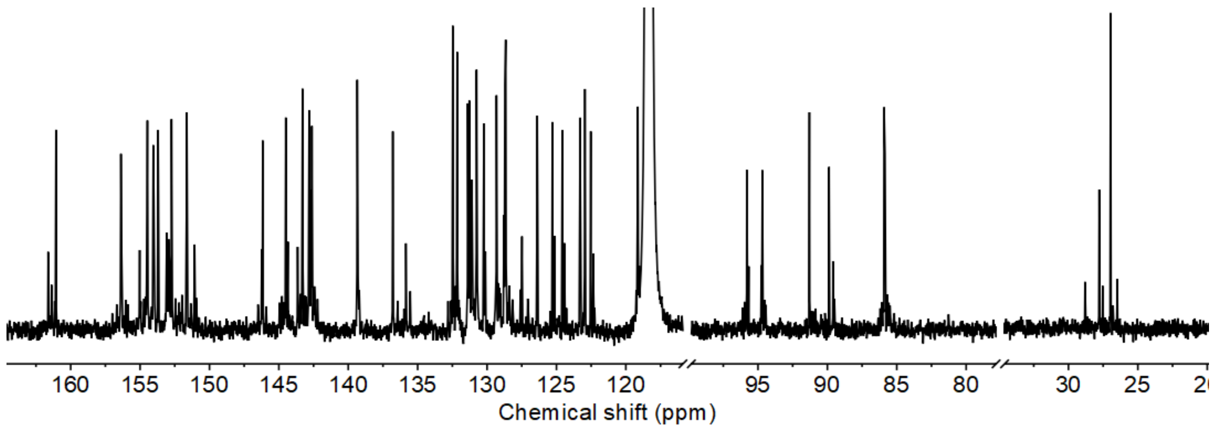

Figure S285 Partial ^{13}C NMR (101 MHz, CD_3CN) of $[Pd_2(1AB)_2(2AC)_2](BF_4)_4$.

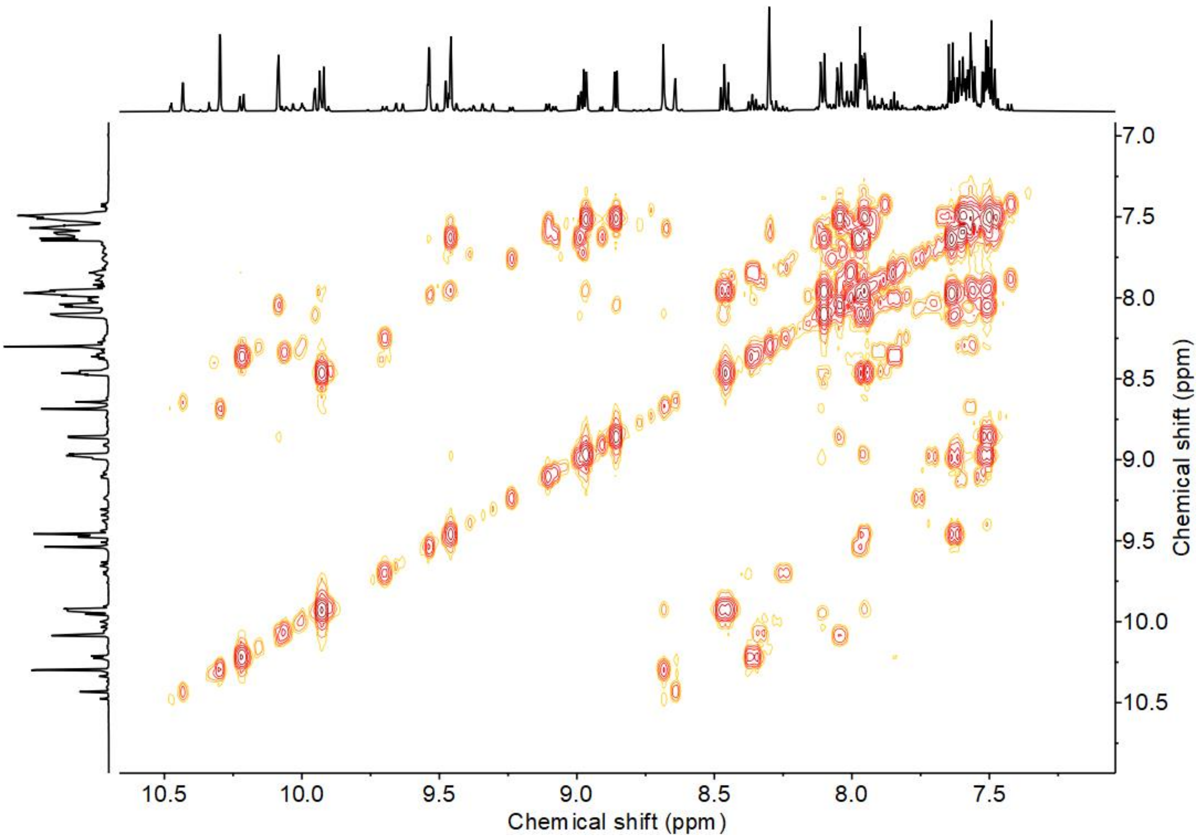

Figure S286 Partial COSY (CD_3CN) of $[Pd_2(1AB)_2(2AC)_2](BF_4)_4$.

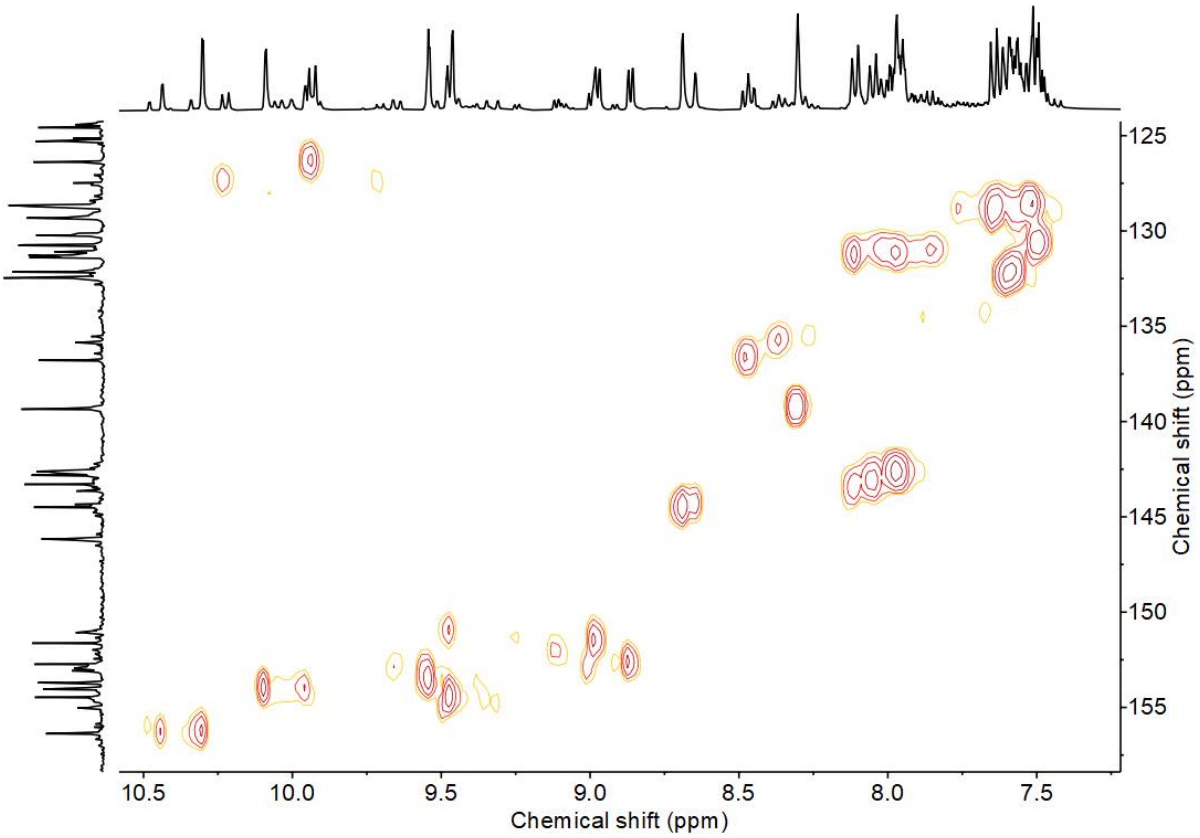

Figure S287 Partial HSQC (CD_3CN) of $[Pd_2(1AB)_2(2AC)_2](BF_4)_4$.

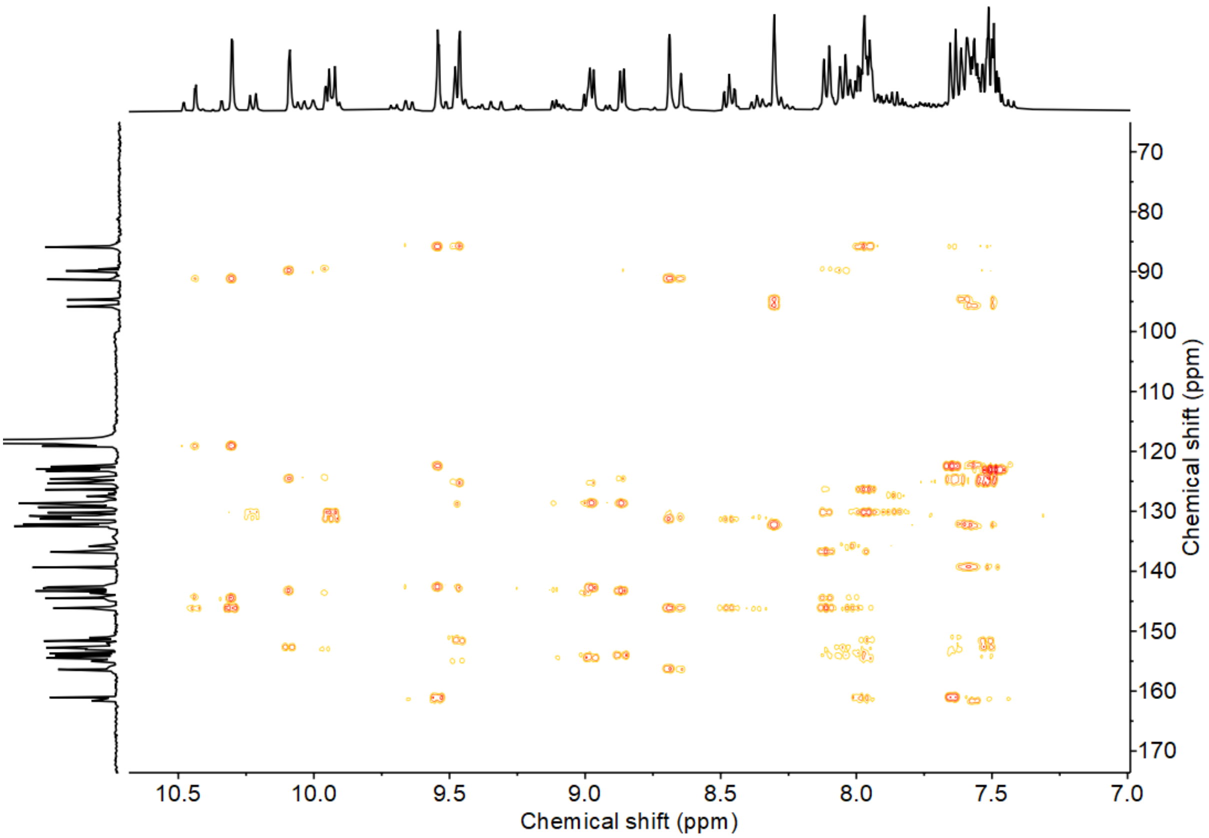

Figure S288 Partial HMBC (CD_3CN) of $[Pd_2(1AB)_2(2AC)_2](BF_4)_4$.

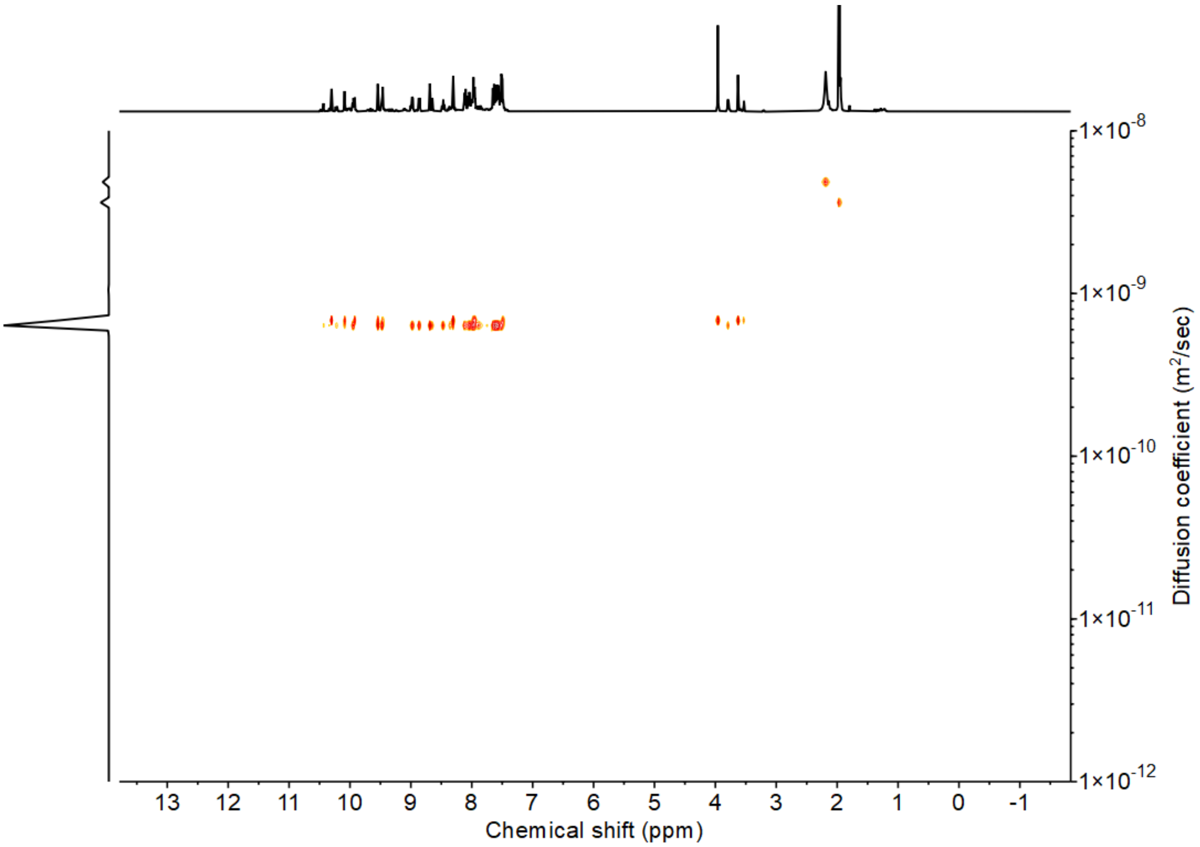

Figure S289 DOSY (CD_3CN) of $[Pd_2(1AB)_2(2AC)_2](BF_4)_4$.

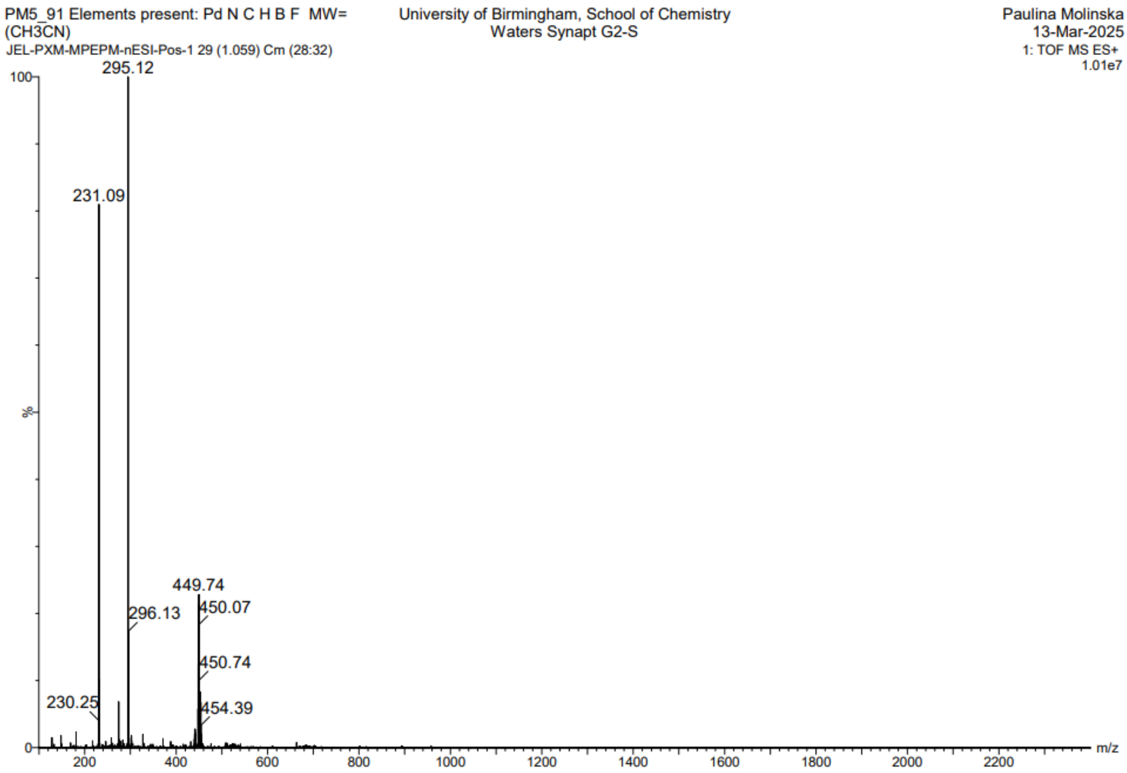

Figure S290 ESI-MS of $[Pd_2(1AB)_2(2AC)_2](BF_4)_4$.

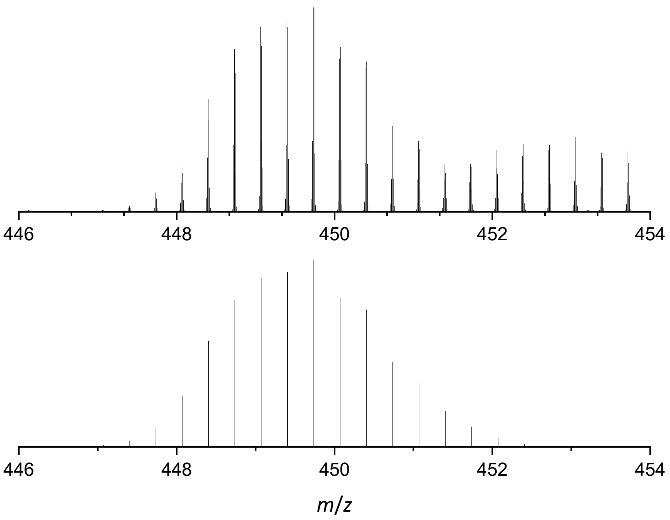

Figure S291 Observed (top) and calculated (bottom) isotopic patterns for $\{[Pd_2(1AB)_2(2AC)_2](BF_4)\}^{3+}$.

S3. Isomer distribution comparisons

$[Pd_2(1AB)_2(2/3/4AA)_2](BF_4)_4$

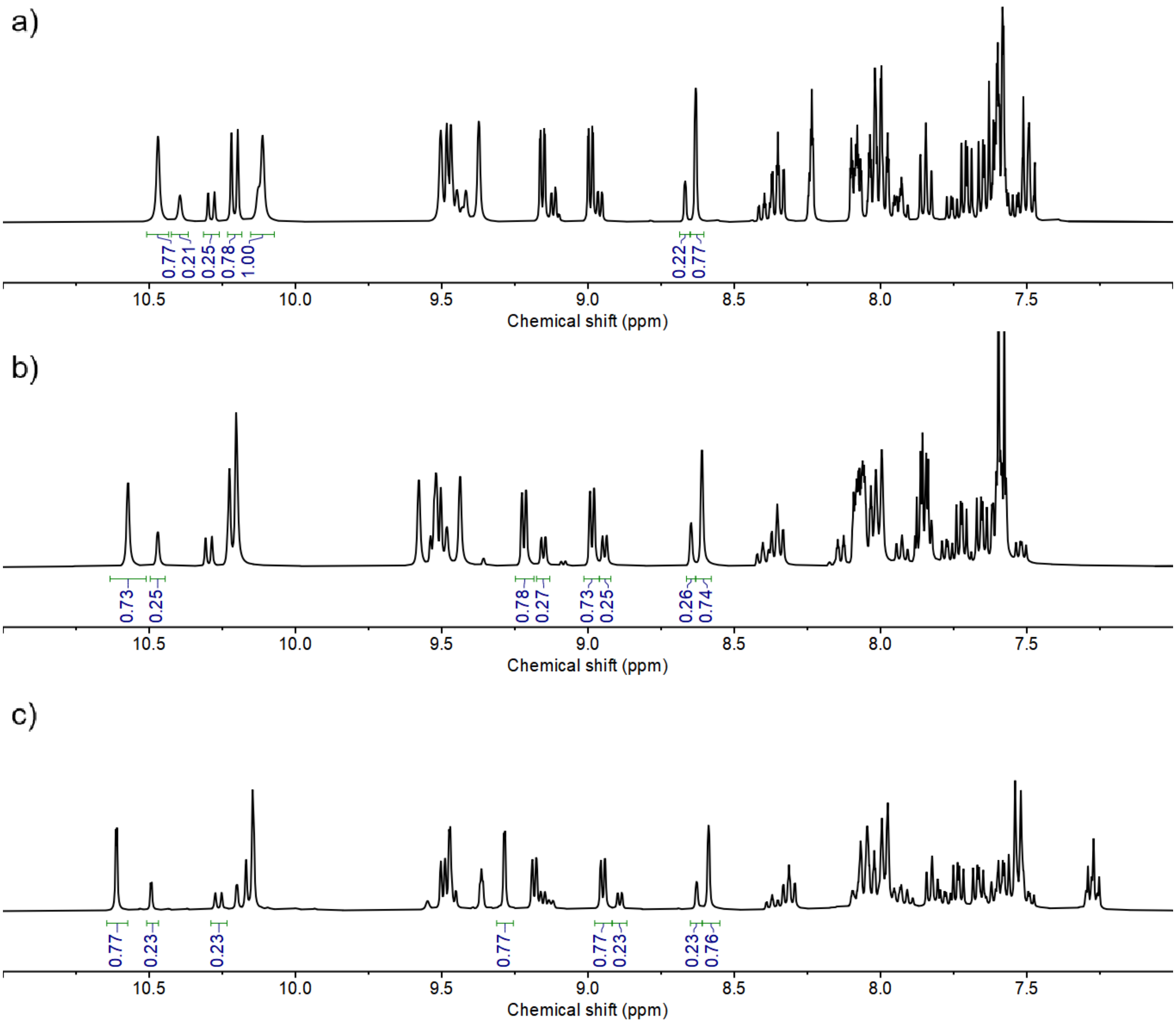

Figure S292 Partial ^1H NMR spectra (400 MHz, CD_3CN) of a) $[Pd_2(1AB)_2(2AA)_2](BF_4)_4$, b) $[Pd_2(1AB)_2(3AA)_2](BF_4)_4$, and c) $[Pd_2(1AB)_2(4AA)_2](BF_4)_4$, with integrations for select peaks of both major and minor isomers.

$[Pd_2(1AC)_2(2/3/4AA)_2](BF_4)_4$

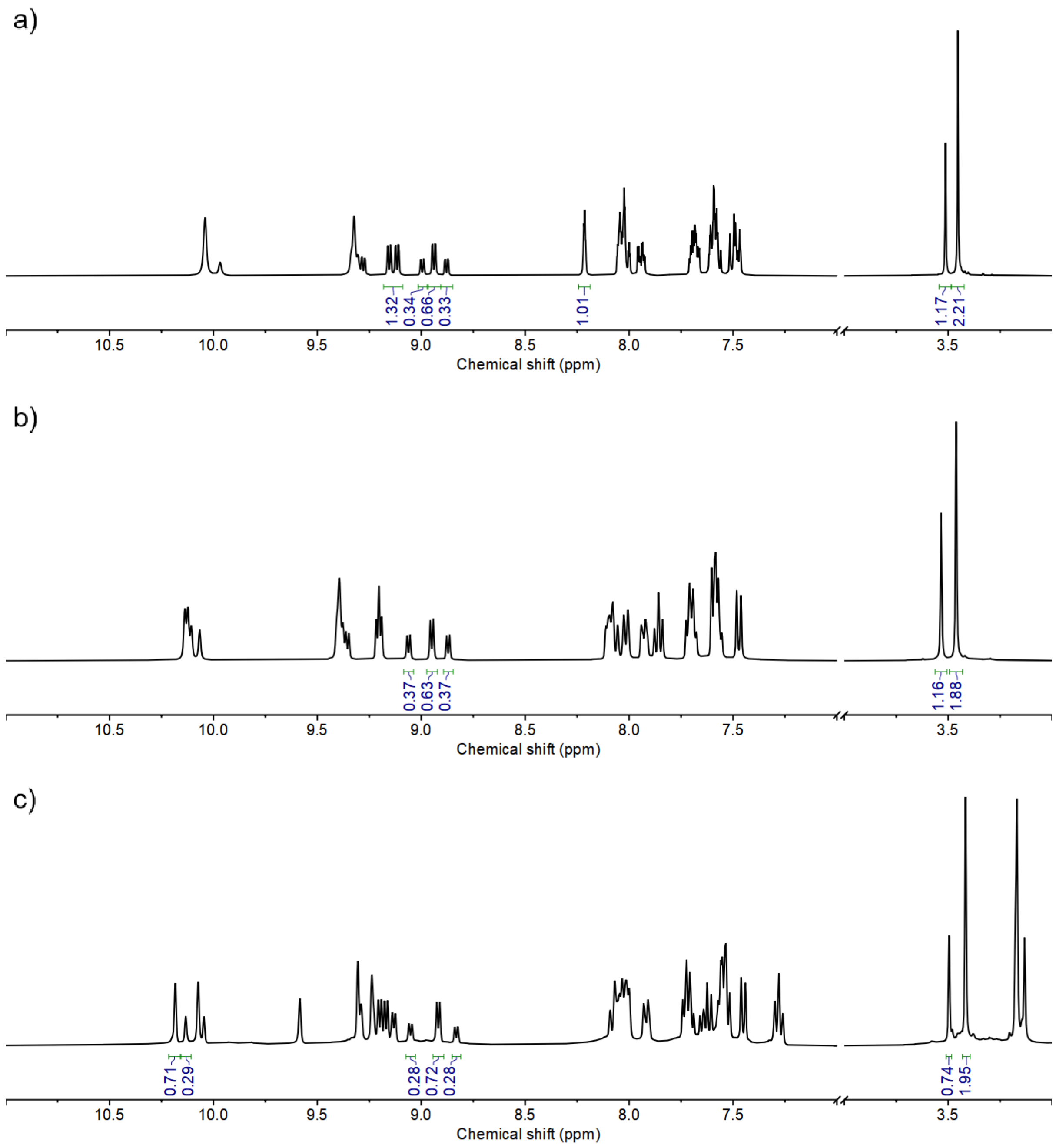

Figure S293 Partial ^{1}H NMR spectra (400 MHz, CD_3CN) of a) $[Pd_2(1AC)_2(2AA)_2](BF_4)_4$, b) $[Pd_2(1AC)_2(3AA)_2](BF_4)_4$, and c) $[Pd_2(1AC)_2(4AA)_2](BF_4)_4$, with integrations for select peaks of both major and minor isomers.

[Pd$_2$(1BC)$_2$(2AA)$_2$](BF$_4$)$_4$

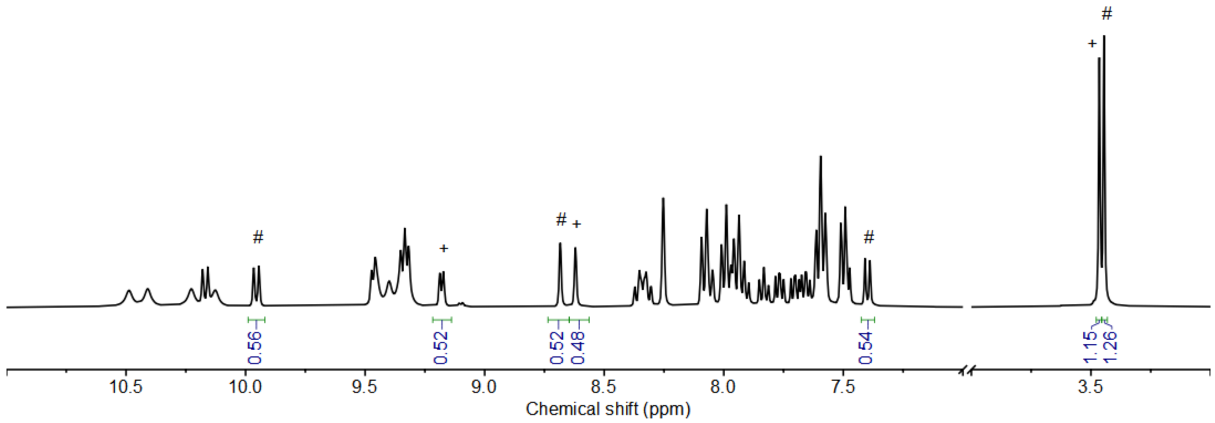

Figure S294 Partial ^{1}H NMR spectrum (400 MHz, CD$_3$CN) of [Pd$_2$(1BC)$_2$(2AA)$_2$](BF$_4$)$_4$ with integrations for select peaks of both *syn* (+) and *anti* (#) isomers.

[Pd$_2$(1BD)$_2$(2AA)$_2$](BF$_4$)$_4$

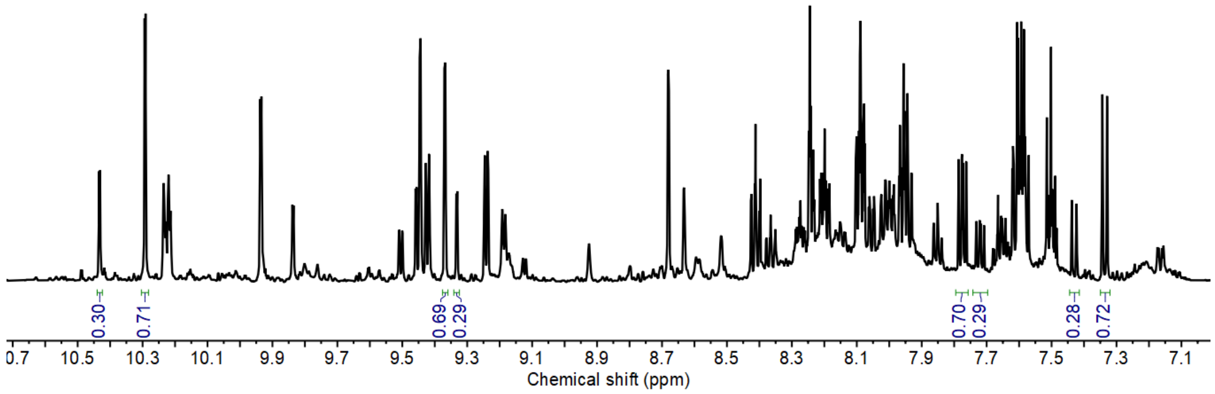

Figure S295 Partial ^{1}H NMR spectrum (600 MHz, CD$_3$CN) of [Pd$_2$(1BD)$_2$(2AA)$_2$](BF$_4$)$_4$ with integrations for select peaks of both major and minor isomers.

$[Pd_2(1AE)_2(2AA)_2](BF_4)_4$

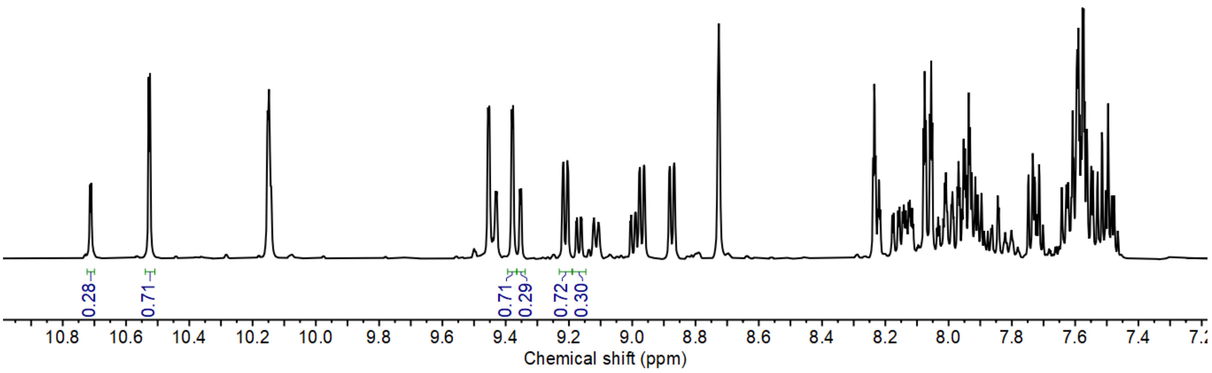

Figure S296 Partial ^1H NMR spectrum (400 MHz, CD_3CN) of $[Pd_2(1AE)_2(2AA)_2](BF_4)_4$ with integrations for select peaks of both major and minor isomers.

$[Pd_2(1CE)_2(2AA)_2](BF_4)_4$

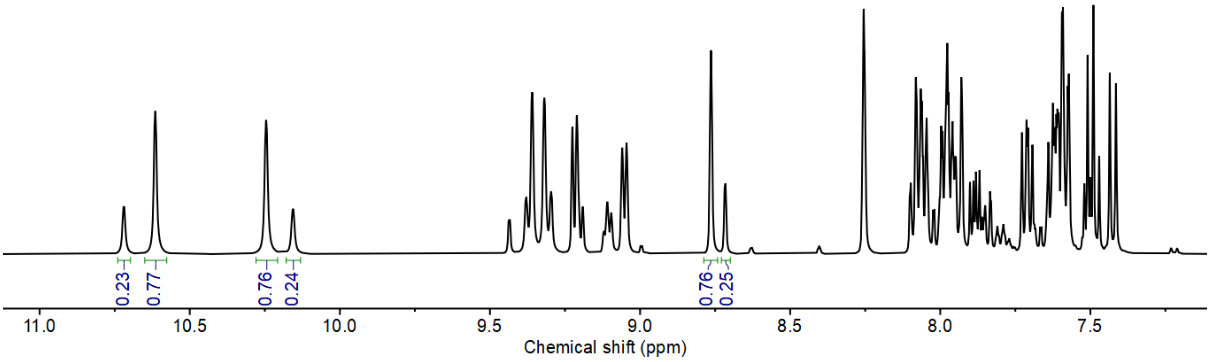

Figure S297 Partial ^1H NMR spectrum (400 MHz, CD_3CN) of $[Pd_2(1CE)_2(2AA)_2](BF_4)_4$ with integrations for select peaks of both major and minor isomers.

$[Pd_2(1AA)_2(2AB)_2](BF_4)_4$

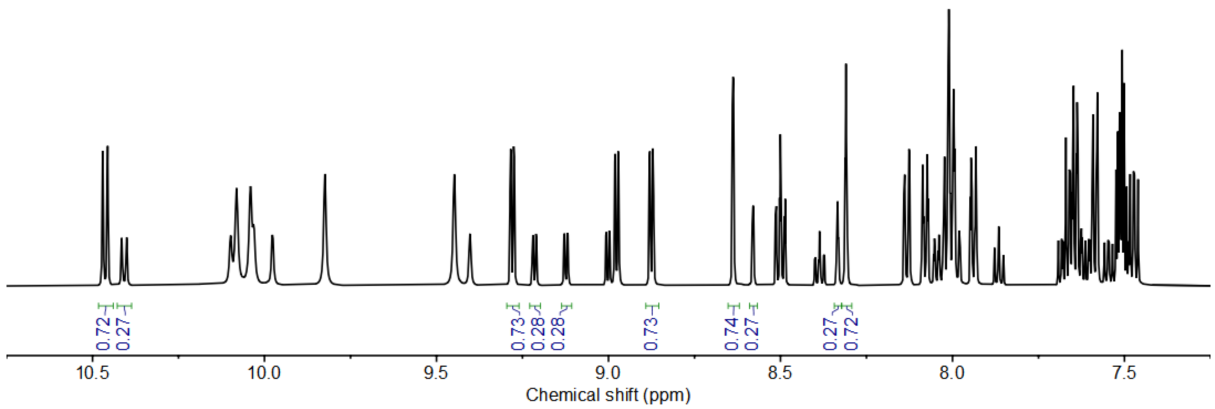

Figure S298 Partial ^{1}H NMR spectrum (600 MHz, CD_3CN) of $[Pd_2(1AA)_2(2AB)_2](BF_4)_4$ with integrations for select peaks of both major and minor isomers.

$[Pd_2(1AA)_2(2AC)_2](BF_4)_4$

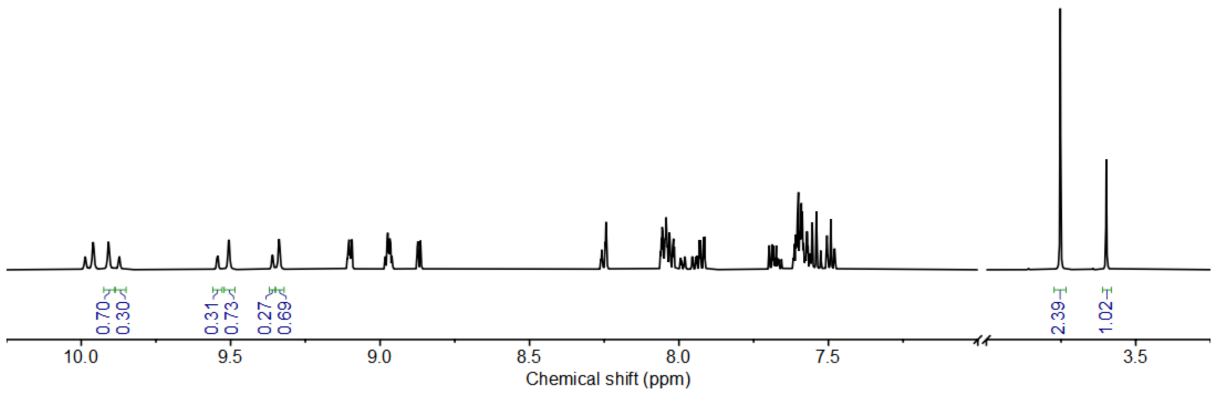

Figure S299 Partial ^{1}H NMR spectrum (600 MHz, CD_3CN) of $[Pd_2(1AA)_2(2AC)_2](BF_4)_4$ with integrations for select peaks of both major and minor isomers.

$[Pd_2(1AB)_2(2AB)_2](BF_4)_4$

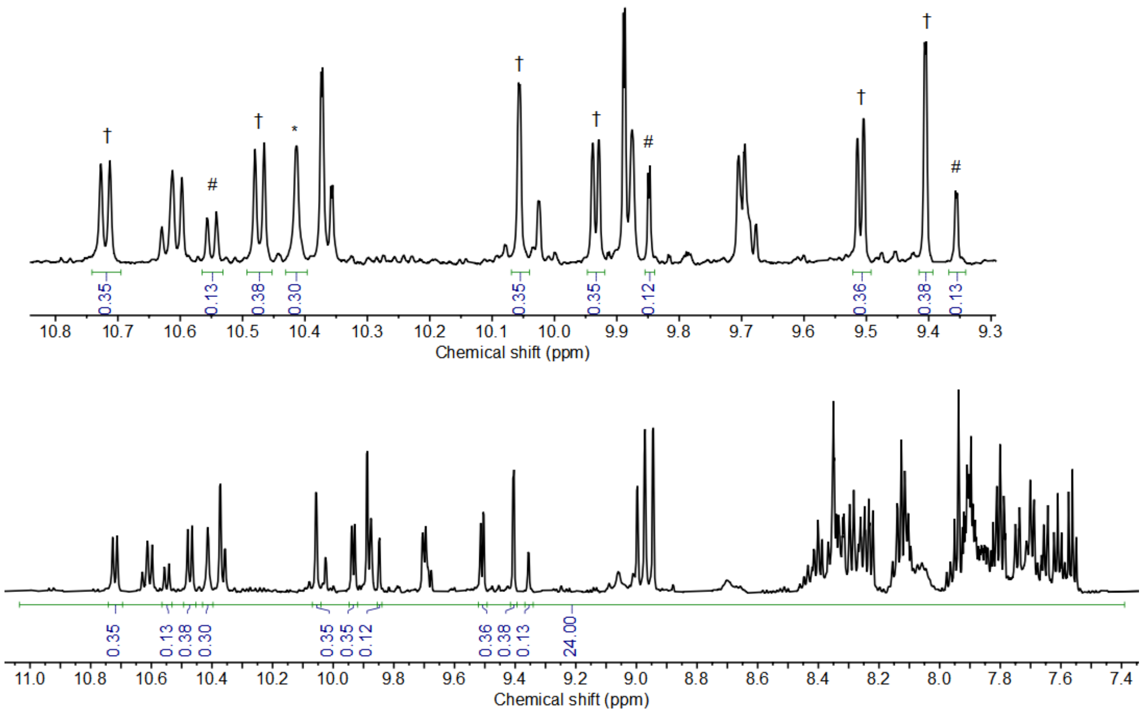

Figure S300 Partial ^1H NMR spectrum (600 MHz, CD_3CN) of $[Pd_2(1AB)_2(2AB)_2](BF_4)_4$ with integrations for select peaks of major (†) and minor (#) isomers and $[Pd_2(2AB)_4]^{4+}$ (*).

$[Pd_2(1AC)_2(2AC)_2](BF_4)_4$

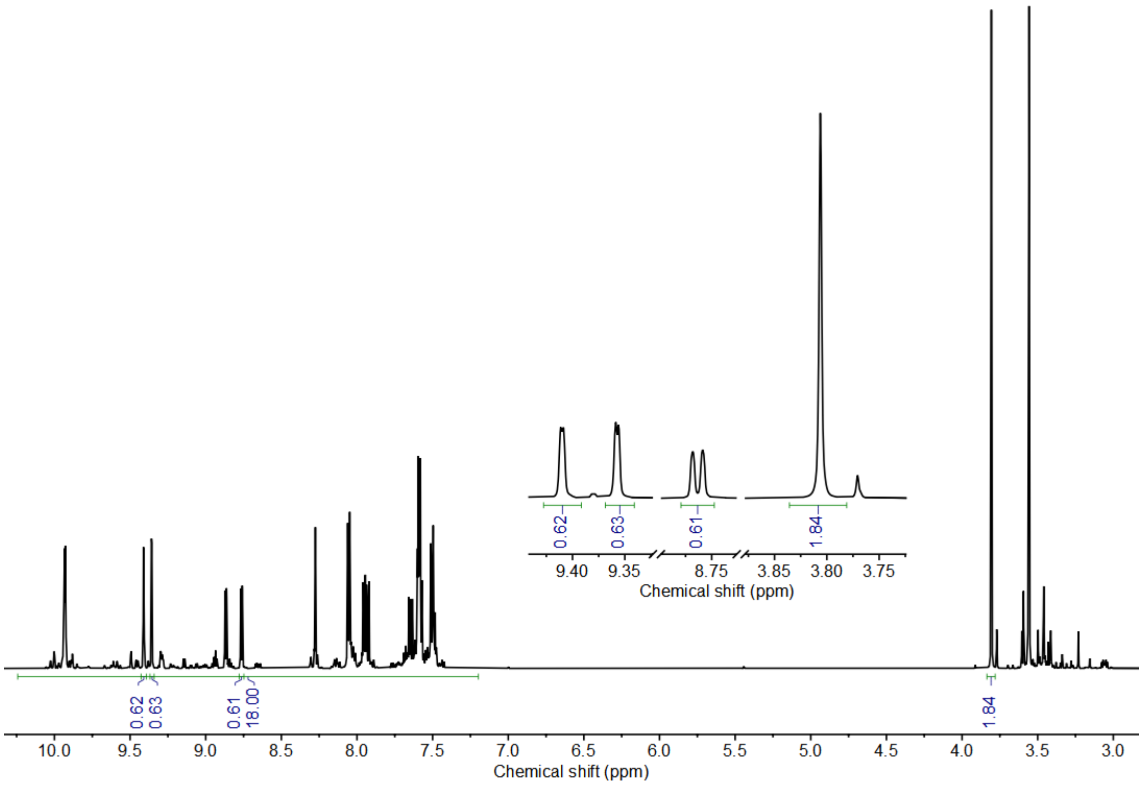

Figure S301 Partial ^1H NMR spectrum (600 MHz, CD_3CN) of $[Pd_2(1AC)_2(2AC)_2](BF_4)_4$ with integrations for select peaks of major isomer.

$[Pd_2(1AB)_2(2AC)_2](BF_4)_4$

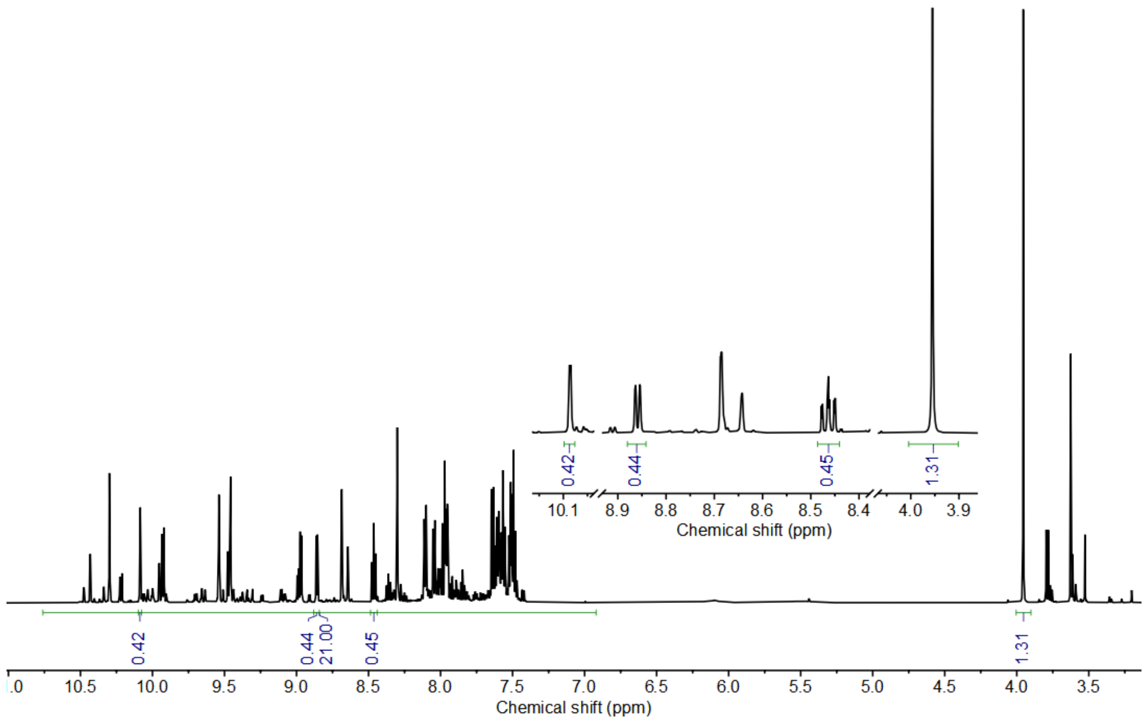

Figure S302 Partial ^1H NMR spectrum (600 MHz, CD_3CN) of $[Pd_2(1AB)_2(2AC)_2](BF_4)_4$ with integrations for select peaks of major isomer.

$[Pd_2(1AC)_2(2AB)_2](BF_4)_4$

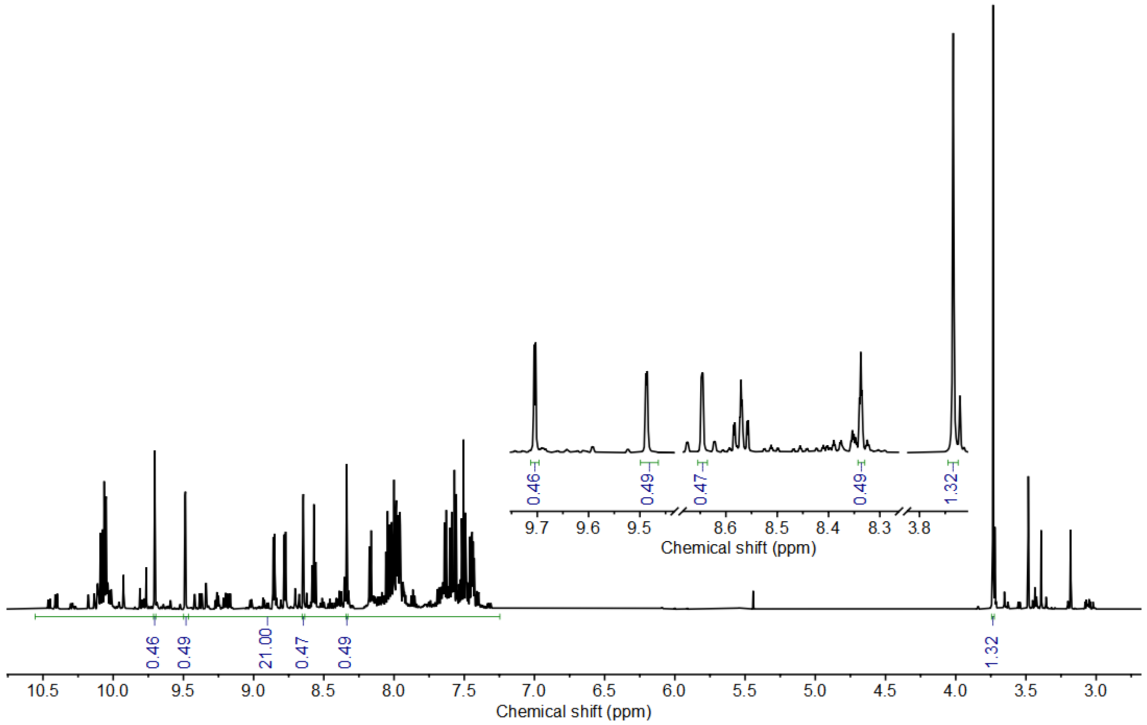

Figure S303 Partial ^1H NMR spectrum (600 MHz, CD_3CN) of $[Pd_2(1AC)_2(2AB)_2](BF_4)_4$ with integrations for select peaks of major isomer.

S4. Solvent and Anion Studies

To probe the impact of solvent on isomer distribution, Pd_2**1AB**$_2$**2AA**$_2$ and Pd_2**1AC**$_2$**2AA**$_2$ were equilibrated and compared in CD_3NO_2, CD_3CN, *d*$_7$-DMF, and *d*$_6$-DMSO that have a range of hydrogen bond acceptor parameter (β) values (3.7, 5.1, 7.4, and 8.8, respectively).[S3] It had been presumed that increasing the strength of HB interactions between solvent and cages would promote formation of the *syn*-isomers that apparently provided superior HB donor sites compared to the anti.

For both Pd_2**1AB**$_2$**2AA**$_2$ and Pd_2**1AC**$_2$**2AA**$_2$, small differences in isomer distribution were observed (Table S1), although no clear correlation between the β value of the solvent and isomer selectivity could be discerned. *d*$_7$-DMF and CD_3CN solutions gave similar proportions of the *syn*-isomer for both quinoline and picoline systems, with a slight reduction observed in CD_3NO_2, and an enhancement seen in *d*$_6$-DMSO.

Table S1 Isomer distributions for $Pd_2$1AB$_2$2AA$_2$ and $Pd_2$1AC$_2$2AA$_2$ across solvents with a range of β values.

Solvent	Solvent β value[Error! Bookmark not defined.]	*syn*-isomer composition (%)[a]	
		Pd_2**1AB**$_2$**2AA**$_2$	Pd_2**1AC**$_2$**2AA**$_2$
CD_3NO_2	3.7	71±1	61±2
CD_3CN	5.1	77±2	65±2
d$_7$-DMF	7.4	75±3	66±2
d$_6$-DMSO	8.8	86±1	81±1

[a]determined from integration of select isolated peaks in ^{1}H NMR spectra.

It is noted that, when equilibrating the cages in *d*$_6$-DMSO, signals for the homoleptic species Pd_2**2AA**$_4$ could be seen to emerge by ^{1}H NMR spectroscopy.

[Pd_2(**1AC**)$_2$(**2AA**)$_2$](BF_4)$_4$

[Pd_2(**1AC**)$_2$(**2AA**)$_2$](BF_4)$_4$ was dissolved in CD_3NO_2, CD_3CN, d_7-DMF or d6-DMSO and stood at 70 °C (for CD_3NO_2, CD_3CN, d_7-DMF) or 50 °C (d_6-DMSO) for 24 h, after which no further changes were observed by ^1H NMR. The sample was stood at room temperature for 24 h prior to data collection

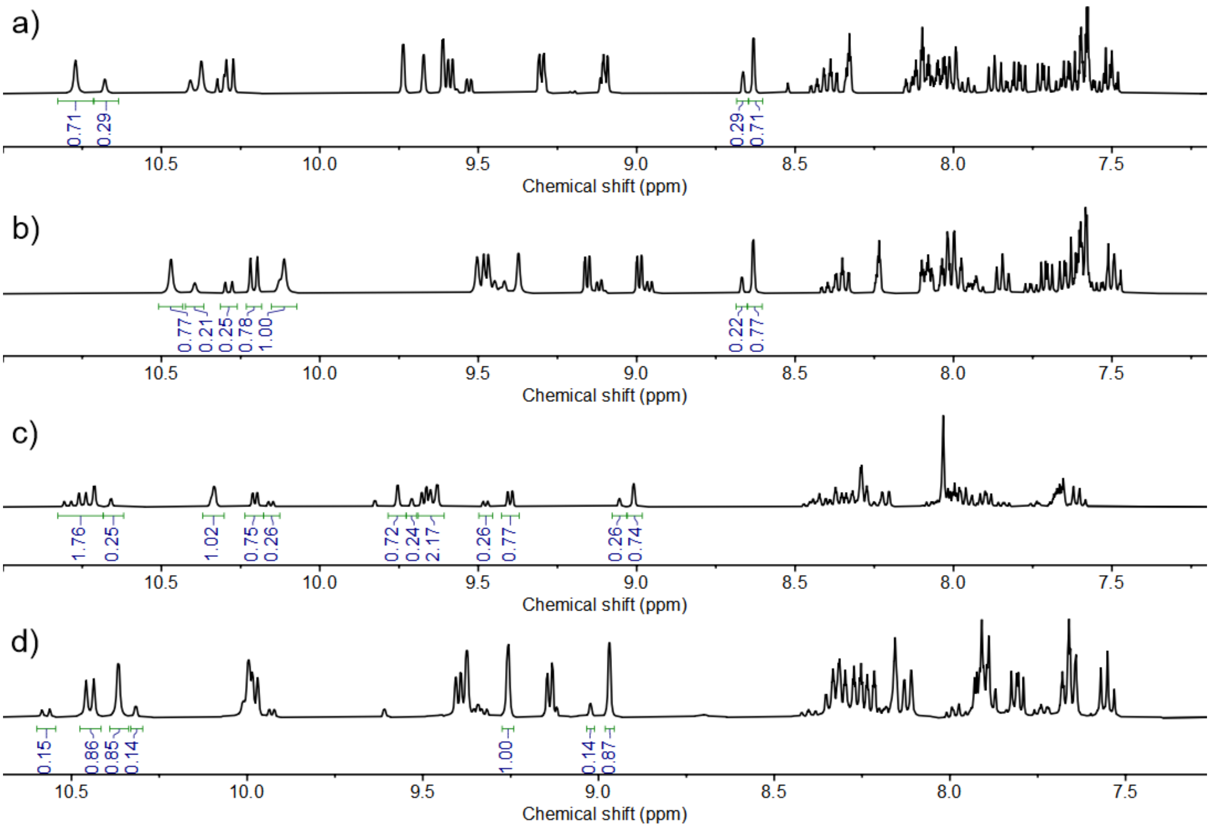

Figure S304 Partial ^1H NMR spectra (400 MHz) of [Pd_2(1AC)$_2$(2AA)$_2$](BF_4)$_4$ in a) CD_3NO_2, b) CD_3CN, c) d_7-DMF, and d) d_6-DMSO.

[Pd$_2$(**1AB**)$_2$(**2AA**)$_2$](BF$_4$)$_4$

[Pd$_2$(**1AB**)$_2$(**2AA**)$_2$](BF$_4$)$_4$ was dissolved in CD_3NO_2, CD_3CN, *d*$_7$-DMF or d6-DMSO and stood at 70 °C (for CD_3NO_2, CD_3CN, *d*$_7$-DMF) or 50 °C (*d*$_6$-DMSO) for 24 h, after which no further changes were observed by ^{1}H NMR. The sample was stood at room temperature for 24 h prior to data collection

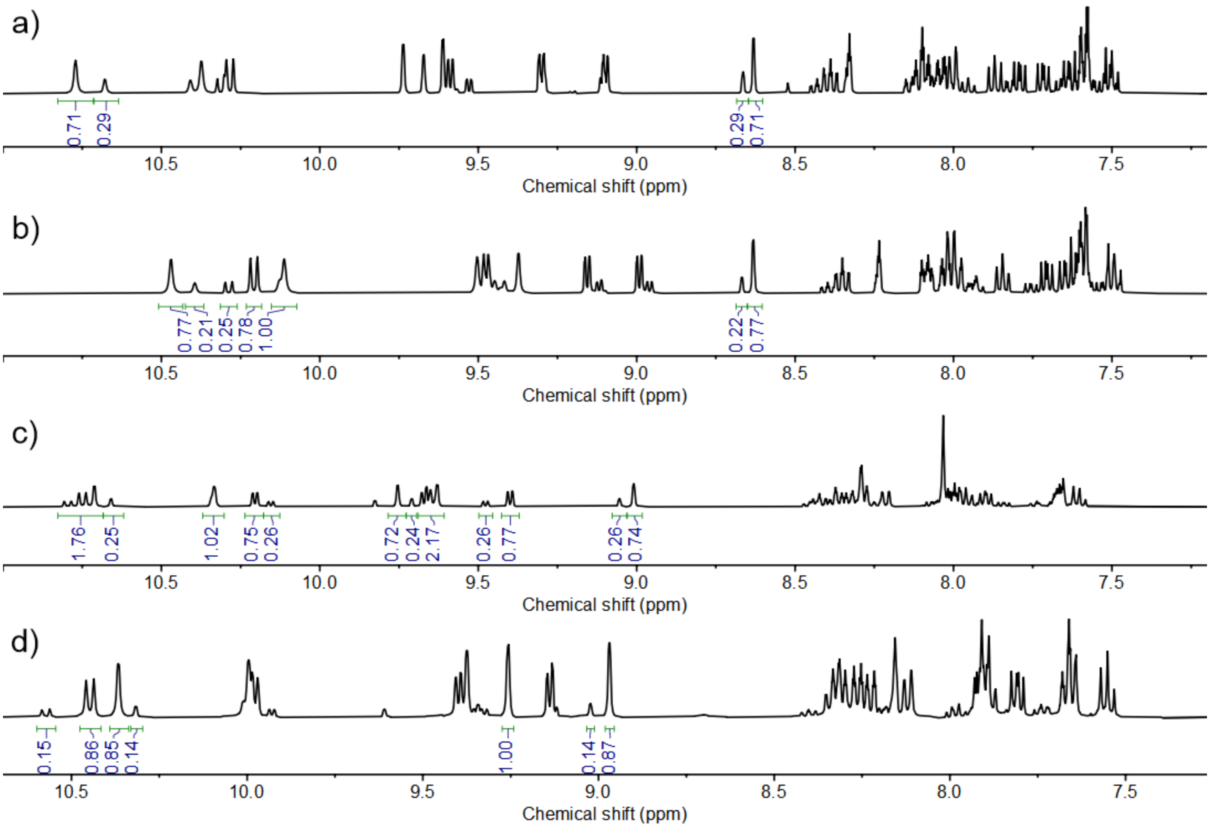

Figure S305 Partial ^{1}H NMR spectra (400 MHz) of [Pd$_2$(1AB)$_2$(2AA)$_2$](BF$_4$)$_4$ in a) CD_3NO_2, b) CD_3CN, c) *d*$_7$-DMF, and d) *d*$_6$-DMSO.

To probe differences in interactions between the external coordination spheres of the *syn*- and *anti*-isomers with anions, Bu_4NOTs was titrated into ~20 mM solutions of Pd_2**1AB**$_2$**2AA**$_2$ and Pd_2**1AC**$_2$**2AA**$_2$ in CD_3CN. For both systems, initially, formation of a host-guest adduct in slow exchange on the NMR timescale was observed until 1 eq. of Bu_4NOTs had been added. Further addition of 2 eq. of Bu_4NOTs resulted in downfield shifts of exohedral pyridyl protons *B*, *b* and *b'*, indicating interactions of the OTs anion with the external faces of the cages. Greater downfield shifts were observed for the *syn*-isomers compared to the *anti*.

Pd_2(**1AB**)2(**2AA**)$_2$

Table S2 Chemical shifts of protons H_B, H_b and $H_{b'}$ for $[Pd_2(1AB)_2(2AA)_2](BF_4)_4$ upon addition of 1 eq. of Bu_4NOTs (forming a 1:1 host-guest adduct) and 3 eq. of Bu_4NOTs.

	δ (ppm)					
	H_B		H_b		$H_{b'}$	
	syn	*anti*	*syn*	*anti*	*syn*	*anti*
Pd_2**1AB**$_2$**2AA**$_2$⊃OTs	9.039	9.002	9.569	9.544	-	-
Pd_2**1AB**$_2$**2AA**$_2$⊃OTs + 2 OTs	9.429	9.358	10.117	10.037	9.634	9.556
Δ	0.390	0.356	0.548	0.493	-	-

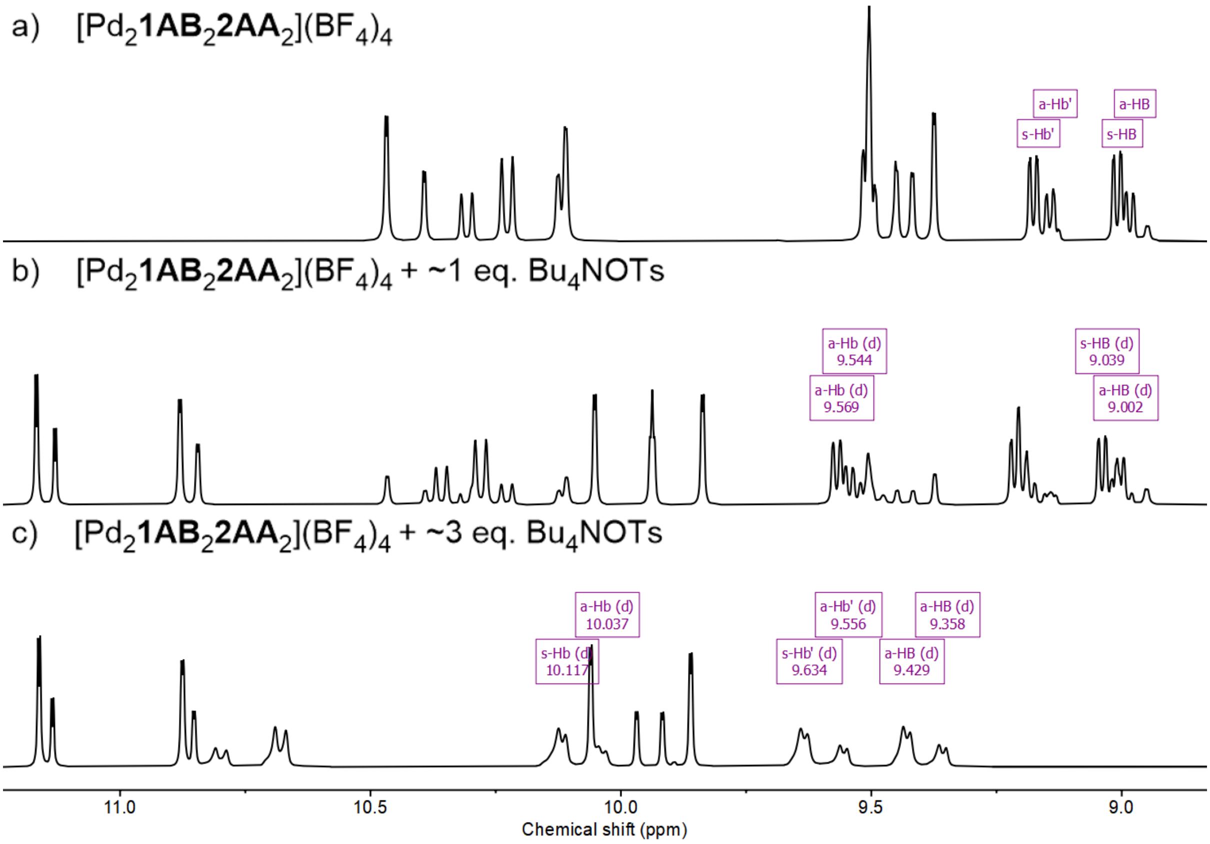

Figure S306 ^{1}H NMR spectra (400 MHz, CD_3CN) of $[Pd_2(1AB)_2(2AA)_2](BF_4)_4$ with a) 0 eq., b) 1 eq., and c) 3 eq. of Bu_4NOTs.

$Pd_2(\mathbf{1AC})_2(\mathbf{2AA})_2$

Table S3 Chemical shifts of protons H_B, H_b and $H_{b'}$ for $[Pd_2(1AC)_2(2AA)_2](BF_4)_4$ upon addition of 1 eq. of Bu_4NOTs (forming a 1:1 host-guest adduct) and 3 eq. of Bu_4NOTs.

	δ (ppm)					
	H_B		H_b		$H_{b'}$	
	syn	*anti*	*syn*	*anti*	*syn*	*anti*
$Pd_2\mathbf{1AC}_2\mathbf{2AA}_2$⊃OTs	9.003	8.953	9.261	9.371	9.194	9.084
$Pd_2\mathbf{1AC}_2\mathbf{2AA}_2$⊃OTs + 2 OTs	9.413	9.218	9.636	9.733	9.524	9.316
Δ	0.410	0.265	0.375	0.362	0.330	0.232

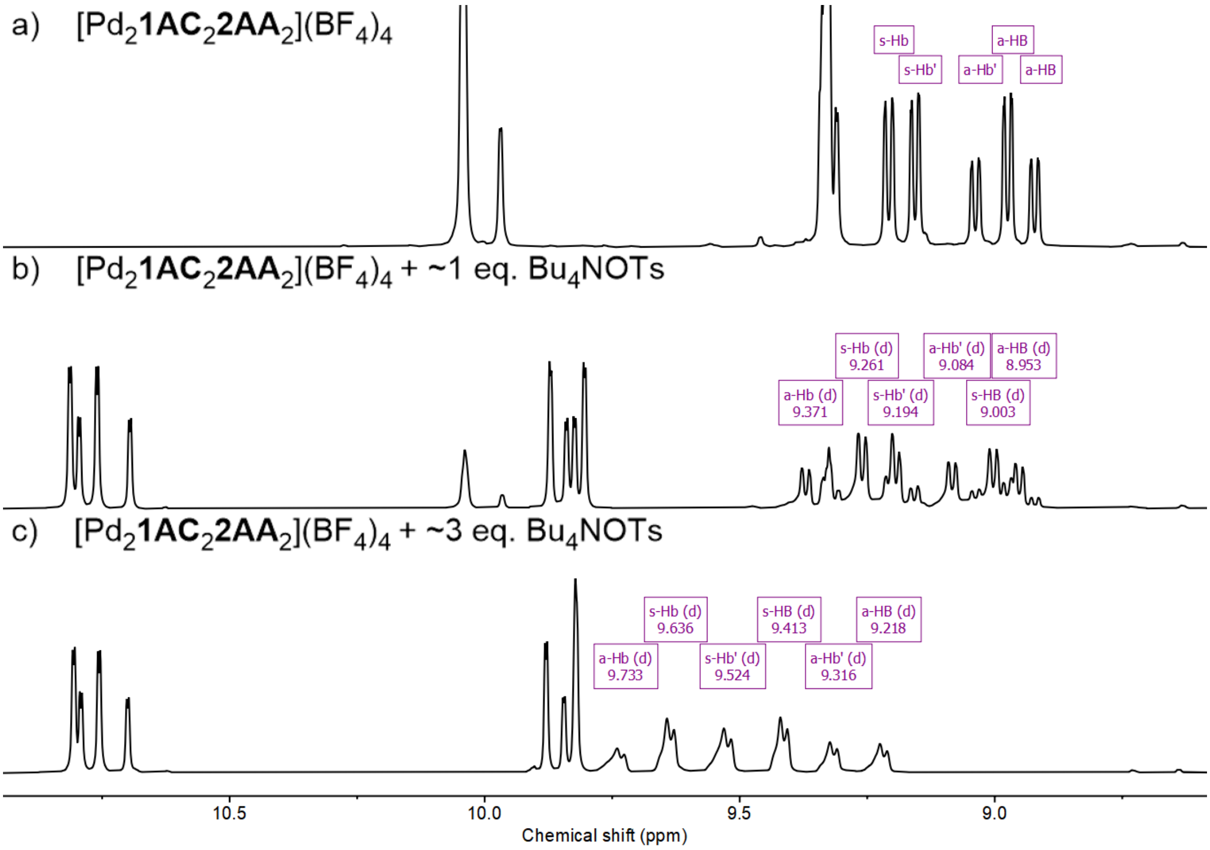

Figure S307 ^{1}H NMR spectra (400 MHz, CD_3CN) of $[Pd_2(1AC)_2(2AA)_2](BF_4)_4$ with a) 0 eq., b) 1 eq., and c) 3 eq. of Bu_4NOTs.

S5. Concentration Studies

1mM

$[Pd_2(\mathbf{1AB})_2(\mathbf{2AA})_2](BF_4)_4$ (1.2 mg, 0.76 µmol, 1 eq.) was dissolved in CD_3CN (0.75 mL) to obtain a 1 mM solution. The sample was equilibrated at 70 °C for 24 h, then stood at room temperature for 24 h.

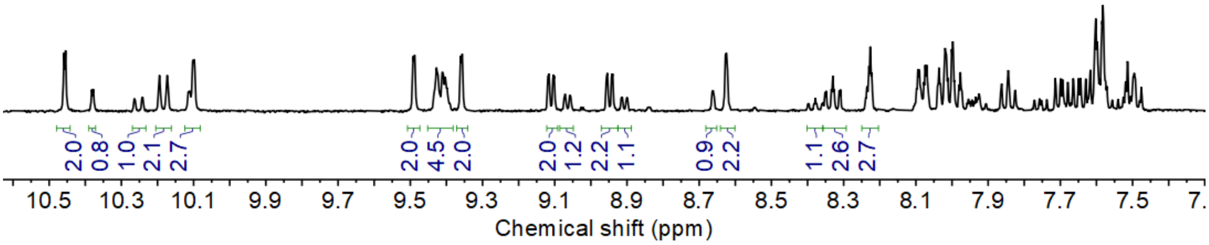

Figure S308 Partial ^1H NMR (400 MHz, CD_3CN) of 1 mM $[Pd_2(1AB)_2(2AA)_2](BF_4)_4$ with integrations relative to the protons in the major isomer cage.

5mM

$[Pd_2(\mathbf{1AB})_2(\mathbf{2AA})_2](BF_4)_4$ (5.9 mg, 3.7 µmol, 1 eq.) was dissolved in CD_3CN (0.75 mL) to obtain a 5 mM solution. The sample was equilibrated at 70 °C for 24 h, then stood at room temperature for 24 h.

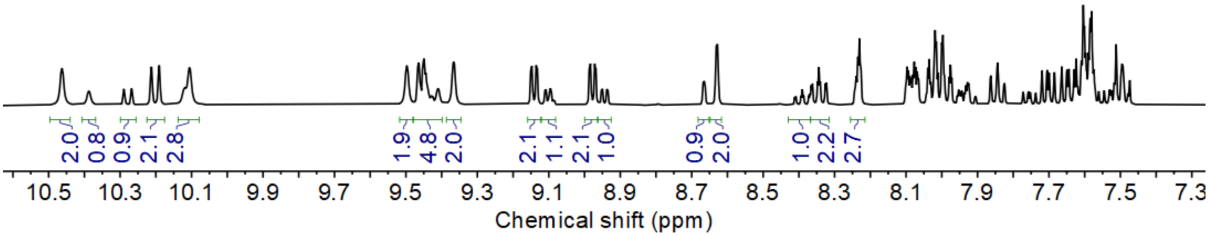

Figure S309 Partial ^1H NMR (400 MHz, CD_3CN) of 5 mM $[Pd_2(1AB)_2(2AA)_2](BF_4)_4$ with integrations relative to the protons in the major isomer cage.

10mM

$[Pd_2(\mathbf{1AB})_2(\mathbf{2AA})_2](BF_4)_4$ (11.9 mg, 7.5 µmol, 1 eq.) was dissolved in CD_3CN (0.75 mL) to obtain a 10 mM solution. The sample was equilibrated at 70 °C for 24 h, then stood at room temperature for 24 h.

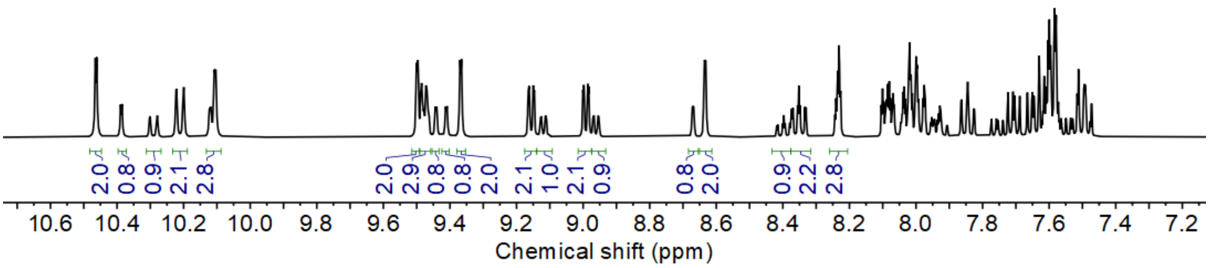

Figure S310 Partial ^1H NMR (400 MHz, CD_3CN) of 10 mM $[Pd_2(1AB)_2(2AA)_2](BF_4)_4$ with integrations relative to the protons in the major isomer cage.

S6. Geometry Optimised Structures

All models were initially constructed using Avogadro,[S4] starting from the crystal structure of *cis*-[Pd$_2$**1AA**$_2$**2AA**$_2$](BF$_4$)$_4$ (CCDC# 2237144) and optimised with UFF[S5] to provide initial atom coordinates. Geometry optimisation of these structures in implicit solvent (acetonitrile or DMSO) using the CPCM model was subsequently performed using the semi-empirical method GFN2-xTB[20] within Orca 4.2.1[21] with default SCF convergence criteria.

Table S4 Single point and relative energies of Pd$_2$1AB$_2$2/3/4AA$_2$ cage *syn*- and *anti*-isomers (acetonitrile).

Cage	Pd$_2$**1AB**$_2$**2AA**$_2$		Pd$_2$**1AB**$_2$**3AA**$_2$		Pd$_2$**1AB**$_2$**4AA**$_2$	
Isomer	*syn*	*anti*	*syn*	*anti*	*syn*	*anti*
E (Ha)	-207.07872874694	-207.0824135378	-207.61830596046	-207.62209872895	-213.4217960078	-213.42533841781
ΔE (Ha)	0.003684791		0.003792768		0.00354241	
ΔE (kJ mol^{-1})	9.7		10.0		9.3	

Table S5 Single point and relative energies of Pd$_2$1$^P{}_2$2$_2$ cage *syn*- and *anti*-isomers (acetonitrile)

Cage	Pd$_2$**1AC**$_2$**2AA**$_2$		Pd$_2$**1AC**$_2$**3AA**$_2$		Pd$_2$**1AC**$_2$**4AA**$_2$	
Isomer	*syn*	*anti*	*syn*	*anti*	*syn*	*anti*
E (Ha)	-194.21943458161	-194.22317177262	-194.75912119632	-194.76295067675	-200.56225214694	-200.56604600809
ΔE (Ha)	0.003737191		0.00382948		0.003793861	
ΔE (kJ mol^{-1})	9.8		10.1		10.0	

Table S6 Single point and relative energies of Pd$_2$1BC$_2$2AA$_2$ *syn*- and *anti*-isomers (acetonitrile).

Isomer	*syn*	*anti*
E (Ha)	-213.45146015594	-213.45184787254
ΔE (Ha)	0.000387717	
ΔE (kJ mol^{-1})	1.0	

Table S7 Single point and relative energies of *syn*- and *anti*-isomers of Pd$_2$1AA$_2$2AB$_2$ and Pd$_2$1AA$_2$2AC$_2$ cages (acetonitrile).

Cage	Pd$_2$**1AA**$_2$**2AB**$_2$		Pd$_2$**1AA**$_2$**2AC**$_2$	
Isomer	*syn*	*anti*	*syn*	*anti*
E (Ha)	-207.07051110287	-207.07747520702	-194.21312166286	-194.21924908402
ΔE (Ha)	0.006964104		0.006127421	
ΔE (kJ mol^{-1})	18.3		16.1	

Table S8 Single point and relative energies of Pd$_2$1AC$_2$2AC$_2$ isomers (acetonitrile).

Isomer	**I**<br>*syn*-(*syn*-**1**,*syn*-**2**)	**II**<br>(*anti*-**1**,*syn*-**2**)	**III**<br>*cis*-(*anti*-**1**,*anti*-**2**)	**IV**<br>(*syn*-**1**,*anti*-**2**)	**V**<br>*anti*-(*syn*-**1**,*syn*-**2**)	**VI**<br>*trans*-(*anti*-**1**,*anti*-**2**)
Isomer diagram	**1↑↑1<br>2↑↑2**	**1↑↓1<br>2↑↑2**	**1↑↓1<br>2↑↓2**	**1↑↑1<br>2↑↓2**	**1↑↑1<br>2↓↓2**	**1↑↓1<br>2↓↑2**
GFN2-xTB geometry-optimised model	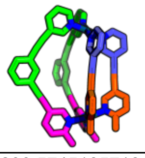	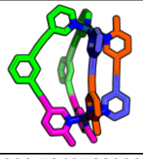	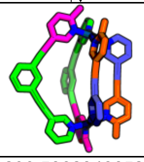	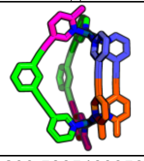	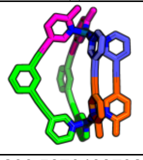	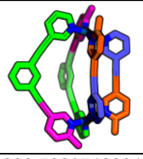
E (Ha)	-200.57451857164	-200.58437689808	-200.58628466522	-200.58654603562	-200.58704087921	-200.59207123918
ΔE (Ha)	0.017552668	0.007694341	0.005786574	0.005525204	0.00503036	0
ΔE (kJ mol^{-1})	46.1	20.2	15.2	14.5	13.2	0

Table S9 Single point and relative energies of $Pd_21AB_22AB_2$ isomers (acetonitrile/DMSO).

Isomer	I	II	III	IV	V	VI
	syn-(*syn*-**1**,*syn*-**2**)	(*anti*-**1**,*syn*-**2**)	*cis*-(*anti*-**1**,*anti*-**2**)	(*syn*-**1**,*anti*-**2**)	*anti*-(*syn*-**1**,*syn*-**2**)	*trans*-(*anti*-**1**,*anti*-**2**)
Isomer diagram	**1↑↑1**<br>**2↑↑2**	**1↑↓1**<br>**2↑↑2**	**1↑↓1**<br>**2↑↓2**	**1↑↑1**<br>**2↑↓2**	**1↑↑1**<br>**2↓↓2**	**1↑↓1**<br>**2↓↑2**
GFN2-xTB geometry-optimised model	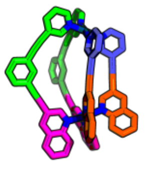	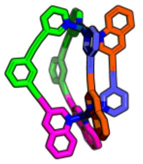	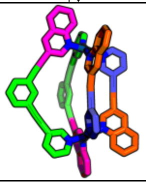	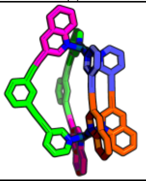	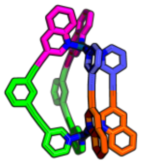	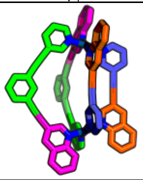
E (Ha)[a]	-226.29134497975	-226.30014427207	-226.30119738053	-226.30295848543	-226.302927585390	-226.3085151112
ΔE (Ha)	0.017170131	0.008370839	0.007317731	0.005556626	0.005587526	0
ΔE (kJ mol^{-1})	45.1	22.0	19.2	14.6	14.7	0

[a]identical energies were obtained in both implicit acetonitrile and DMSO.

Table S10 Single point and relative energies of $Pd_21AC_22AB_2$ isomers (acetonitrile).

Isomer	I	II	III	IV	V	VI
	syn-(*syn*-**1**,*syn*-**2**)	(*anti*-**1**,*syn*-**2**)	*cis*-(*anti*-**1**,*anti*-**2**)	(*syn*-**1**,*anti*-**2**)	*anti*-(*syn*-**1**,*syn*-**2**)	*trans*-(*anti*-**1**,*anti*-**2**)
Isomer diagram	**1↑↑1**<br>**2↑↑2**	**1↑↓1**<br>**2↑↑2**	**1↑↓1**<br>**2↑↓2**	**1↑↑1**<br>**2↑↓2**	**1↑↑1**<br>**2↓↓2**	**1↑↓1**<br>**2↓↑2**
E (Ha)[a]	-213.4325126	-213.4421149	-213.4444555	-213.444646	-213.4441544	-213.450065
ΔE (Ha)	0.017552383	0.007950123	0.00560945	0.00541924	0.005910574	0
ΔE (kJ mol^{-1})	46.1	20.9	14.7	14.2	15.5	0.0

Table S11 Single point and relative energies of $Pd_21AB_22AC_2$ isomers (acetonitrile).

Isomer	I	II	III	IV	V	VI
	syn-(*syn*-**1**,*syn*-**2**)	(*anti*-**1**,*syn*-**2**)	*cis*-(*anti*-**1**,*anti*-**2**)	(*syn*-**1**,*anti*-**2**)	*anti*-(*syn*-**1**,*syn*-**2**)	*trans*-(*anti*-**1**,*anti*-**2**)
Isomer diagram	**1↑↑1**<br>**2↑↑2**	**1↑↓1**<br>**2↑↑2**	**1↑↓1**<br>**2↑↓2**	**1↑↑1**<br>**2↑↓2**	**1↑↑1**<br>**2↓↓2**	**1↑↓1**<br>**2↓↑2**
E (Ha)[a]	-213.4333147	-213.4431283	-213.4439463	-213.445557	-213.4460557	-213.451127
ΔE (Ha)	0.017812335	0.007998731	0.007180721	0.005570052	0.005071303	0
ΔE (kJ mol^{-1})	46.8	21.0	18.9	14.6	13.3	0.0

S7. X-ray Crystallography

X-ray quality crystals of *anti*-[Pd_2(**1AA**)$_2$(**2AB**)$_2$](BF_4)$_4$, *anti*-[Pd_2(**1AC**)$_2$(**4AA**)$_2$](BF_4)$_4$, and *syn*-[Pd_2(**1AC**)$_2$(**3AA**)$_2$](BF_4)$_4$ were all grown using the following general procedure:

1 (30 µmol), **2/3/4** (30 µmol) and [Pd(CH_3CN)$_4$](BF_4)$_2$ (30 µmol) were stirred in MeCN (dry, 5 mL) under N_2 for 24 h. The cooled reaction mixture was filtered through celite and left for vapour diffusion of Et_2O.

The datasets for *anti*-[Pd_2(**1AA**)$_2$(**2AB**)$_2$](BF_4)$_4$ and *anti*-[Pd_2(**1AC**)$_2$(**4AA**)$_2$](BF_4)$_4$ were measured on an Agilent SuperNova diffractometer using an Atlas detector. The dataset for *syn*-[Pd_2(**1AC**)$_2$(**3AA**)$_2$](BF_4)$_4$ was measured on a Rigaku XtaLAB Synergy diffractometer using a HyPix detector. These data collections were driven and processed and absorption corrections were applied using CrysAlisPro.[S6] Using OLEX2,[S7] the structures were solved using ShelXT,[S8] and were refined by a full-matrix least-squares procedure on F^2 in ShelXL.[S9] All non-hydrogen atoms were refined with anisotropic displacement parameters. Hydrogen atoms were fixed as riding models and the isotropic thermal parameters (U_{iso}) were based on the U_{eq} of the parent atom.

CCDC 2390755, 2390756 and 2390758 contain the supplementary crystallographic data for this paper. These data can be obtained free of charge from The Cambridge Crystallographic Data Centre via www.ccdc.cam.ac.uk/data_request/cif.

anti-[Pd_2(1AA)$_2$(2AB)$_2$](BF_4)$_4$

$C_{80}H_{56}B_4F_{16}N_{12}Pd_2$ (*M* =1745.40 g/mol): triclinic, space group P-1 (no. 2), a = 12.9423(2) Å, b = 19.0119(4) Å, c = 20.2700(3) Å, α = 109.672(2)°, β = 93.5810(10)°, γ = 105.223(2)°, V = 4468.80(15) Å^3, Z = 2, T = 100.00(10) K, µ(Cu Kα) = 3.931 mm^{-1}, *Dcalc* = 1.297 g/cm^3, 90234 reflections measured (7.178° ≤ 2Θ ≤ 154.68°), 18676 unique (R_{int} = 0.0448, R_{sigma} = 0.0331) which were used in all calculations. The final R_1 was 0.0613 (I > 2σ(I)) and wR_2 was 0.1862 (all data).

The structure contains one palladium dimer with four BF_4 anions and four molecules of acetonitrile. In one anion, B(3), F(9)-F(12) / B(3), F(9), F(10A)-F(12A), three of the fluorine atoms are disordered over two positions each at a refined percentage occupancy ratio of 50.0 (11) : 50.0 (11). Another anion, B(4), F(13)-F(16) / B(4A), F(13A)-F(16A), is disordered over two positions at a refined percentage occupancy ratio of 52.4 (5) : 47.6 (5). The acetonitrile molecule N(12), C(79)-C(80) / N(12A), C(79A)-C(80A) is disordered over two positions at a refined percentage occupancy ratio of 51.8 (7) : 48.2 (7). The structure contains solvent accessible voids. It was not possible to successfully refine the disordered solvent located in these voids and so a solvent mask has been applied.

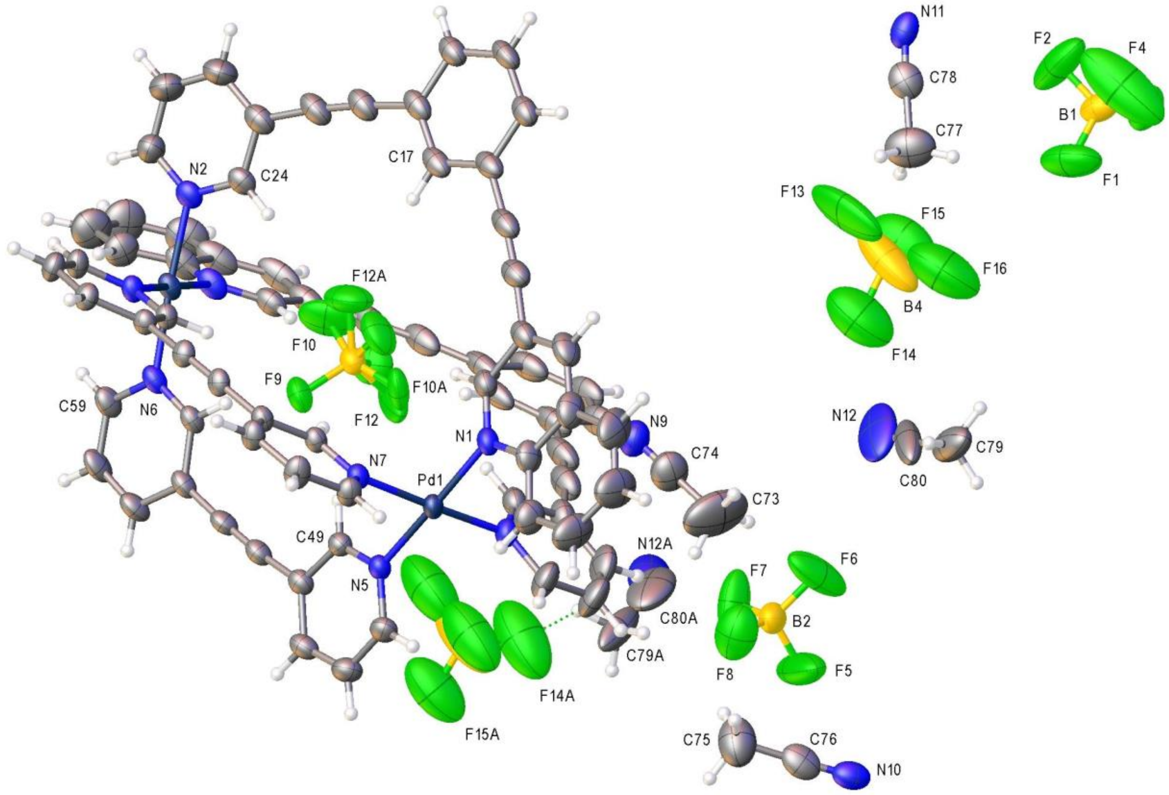

Figure S311 Crystal structure of *anti*-[$Pd_2(1AA)_2(2AB)_2](BF_4)_4$ with ellipsoids drawn at the 50% probability level. The structure contains one palladium dimer with four BF_4 anions and four molecules of acetonitrile. In one anion, B(3), F(9)-F(12) / B(3), F(9), F(10A)-F(12A), three of the fluorine atoms are disordered over two positions each. Another anion, B(4), F(13)-F(16) / B(4A), F(13A)-F(16A), is disordered over two positions, as is the acetonitrile molecule N(12), C(79)-C(80) / C(12A), C(79A)-C(80A). The structure contains solvent accessible voids. It was not possible to successfully refine the disordered solvent located in these voids and so a solvent mask has been applied.

anti-$[Pd_2(1AC)_2(4AA)_2](BF_4)_4$

$C_{74}H_{59}B_4F_{16}N_9OPd_2$ (*M* =1650.34 g/mol): monoclinic, space group C2/m (no. 12), *a* = 12.5045(4) Å, *b* = 42.5947(13) Å, *c* = 15.1704(5) Å, *β* = 100.318(3)°, *V* = 7949.5(4) Å^3, *Z* = 4, *T* = 100.00(10) K, μ(Cu Kα) = 4.382 mm^{-1}, *Dcalc* = 1.379 g/cm^3, 33019 reflections measured (7.232° ≤ 2Θ ≤ 154.506°), 8391 unique (R_{int} = 0.0443, R_{sigma} = 0.0392) which were used in all calculations. The final R_1 was 0.0658 (I > 2σ(I)) and wR_2 was 0.2019 (all data).

The structure contains one palladium dimer, of which only half is crystallographically-unique. The methyl group C(32)-C(34)/C(23)-C(34A) is disordered over two positions at opposite ends of the ligand, at a refined percentage occupancy ratio of 59.0 (14) : 41.0 (14).

The structure contains four BF_4 anions per palladium dimer, one of which, B(1), F(1)-F(3), F(1)_$1, is located on a mirror plane running through B(1), F(2) and F(3). The remaining three BF_4 anions are located on two-fold rotation axes. There is one molecule of diethyl ether per palladium dimer, O(1), C(35)-C(38) / O(1A), C(35A)-C(38A) disordered over two positions at set occupancies of 1/3 : 1/6 due to symmetry. There is also one molecule of acetonitrile per palladium dimer with the non-hydrogen atoms located on a mirror plane. The structure contains a large solvent accessible void. It was not possible to successfully refine the disordered solvent (thought to be another molecule of acetonitrile) located in this void and so a solvent mask has been applied. Symmetry codes used to general equivalent atoms: $1 1-x, y, 1-z.

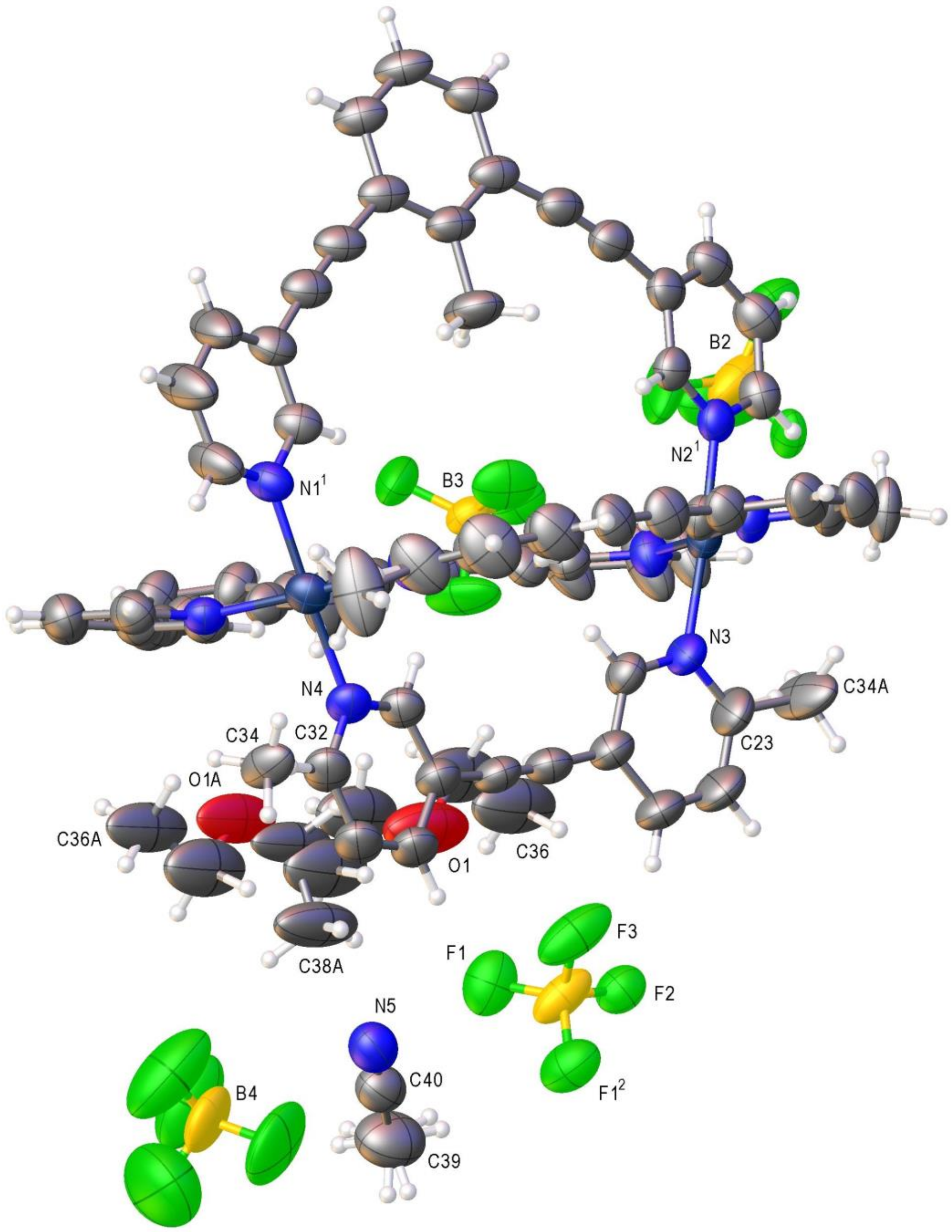

Figure S312 Crystal structure of *anti*-[$Pd_2(1AC)_2(4AA)_2](BF_4)_4$ with ellipsoids drawn at the 50% probability level. Only half of the palladium dimer is crystallographically-unique. The methyl group C(32)-C(34)/C(23)-C(34A) is disordered over two positions. There are four BF_4 anions per palladium dimer, one of which, B(1), F(1)-F(3), F(1)_$1, is located on a mirror plane running through B(1), F(2) and F(3). The remaining three BF_4 anions are located on two-fold rotation axes. There is one molecule of diethyl ether per palladium dimer, O(1), C(35)-C(38) / O(1A), C(35A)-C(38A) disordered over two positions. There is also one molecule of acetonitrile per palladium dimer with the non-hydrogen atoms located on a mirror plane. The structure contains a large solvent accessible void. It was not possible to successfully refine the disordered solvent (thought to be another molecule of acetonitrile) located in this void and so a solvent mask has been applied. Symmetry codes used to general equivalent atoms: $1 1-x, y, 1-z, $2 x, 1-y, z.

syn-[$Pd_2(1AC)_2(3AA)_2](BF_4)_4$

$C_{72}H_{54}B_4F_{16}N_{14}Pd_2$ (*M* =1675.33 g/mol): triclinic, space group P-1 (no. 2), *a* = 11.4465(2) Å, *b* = 18.8695(4) Å, *c* = 20.7359(4) Å, *α* = 63.129(2)°, *β* = 85.660(2)°, *γ* = 82.854(2)°, *V* = 3963.17(15) Å^3, *Z* = 2, *T* = 100.15 K, μ(CuKα) = 4.414 mm^{-1}, *Dcalc* = 1.404 g/cm^3, 68565 reflections measured (4.778° ≤ 2Θ ≤ 156.574°), 15631 unique (R_{int} = 0.0797, R_{sigma} = 0.0558) which were used in all calculations. The final R_1 was 0.0789 (I > 2σ(I)) and wR_2 was 0.2323 (all data).

The structure contains one palladium dimer with four BF_4 anions and four molecules of acetonitrile. The acetonitrile molecule N(14), C(71)-C(72) / N(14A), C(71A)-C(72A) is disordered over two positions at a refined percentage occupancy ratio of 51.1 (12) : 48.9 (12). The structure contains solvent accessible voids. It was not possible to successfully refine the disordered solvent located in these voids and so a solvent mask has been applied.

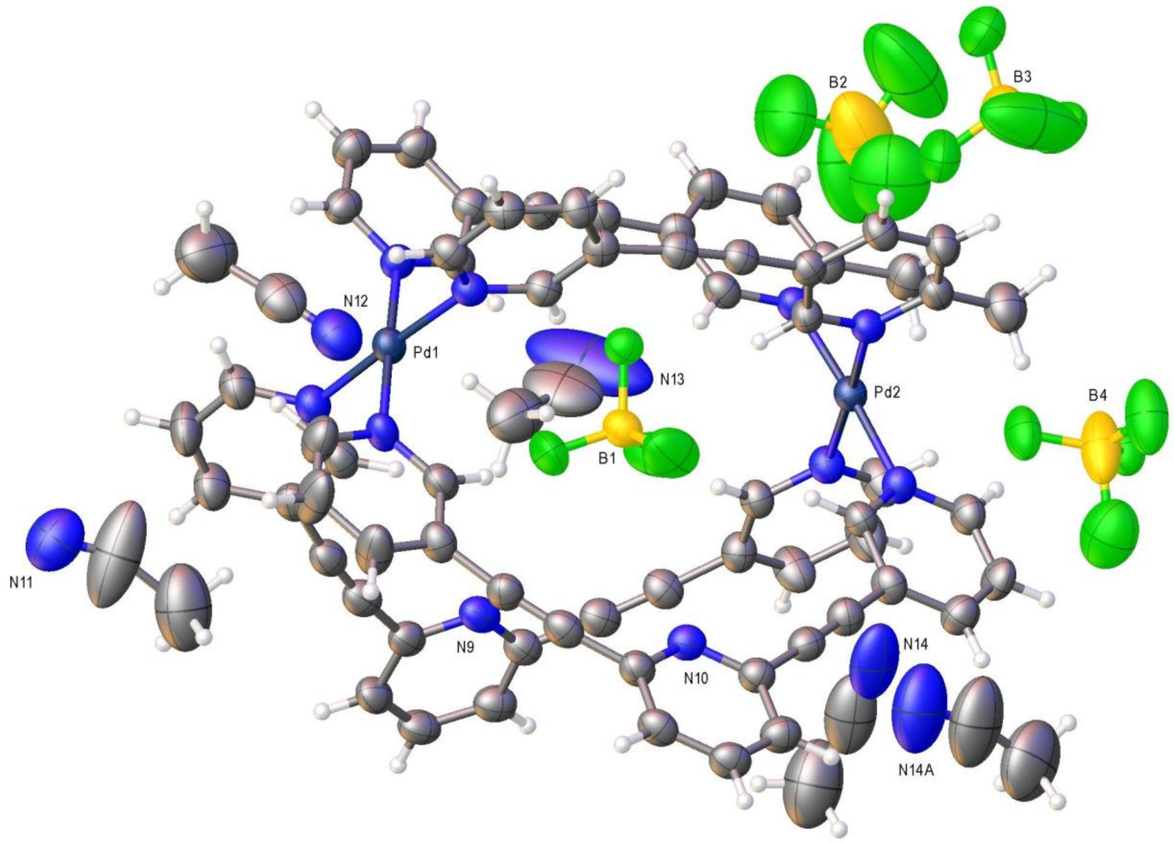

Figure S313 Crystal structure of *syn*-[$Pd_2(1AC)_2(3AA)_2](BF_4)_4$ with ellipsoids drawn at the 50% probability level. The structure contains one palladium dimer with four BF_4 anions and four molecules of acetonitrile, one of which is disordered over two positions. The structure contains solvent accessible voids. It was not possible to successfully refine the disordered solvent located in these voids and so a solvent mask has been applied.

S8. References

[S1] P. Liao, B. W. Langloss, A. M. Johnson, E. R. Knudsen, F. S. Tham, R. R. Julian, R. J. Hooley, *Chem. Commun*. **2010**, *46*, 4932.

[S2] K. J. Kilpin, M. L. Gower, S. G. Telfer, G. B. Jameson, J. D. Crowley, *Inorg. Chem.* **2011**, *50*, 1123.

[S3] M. C. Storer, C. A. Hunter, *Chem. Soc. Rev*., **2022**, *51*, 10064.

[S4] M. D. Hanwell, D. E. Curtis, D. C. Lonie, T. Vandermeersch, E. Zurek, G. R. Hutchison, *J. Cheminf*. **2012**, *4*, 17.

[S5] A. K. Rappé, C. J. Casewit, K. S. Colwell, W. A. Goddard III, W. M. Skiff, *J. Am. Chem. Soc*. **1992**, *114*, 10024.

[S6] CrysAlisPro, Rigaku Oxford Diffraction, 2020 & 2021.

[S7] O. V. Dolomanov, L. J. Bourhis, R. J. Gildea, J. A. K. Howard, H. Puschmann, *J. Appl. Crystallogr.* **2009**, *42*, 339-341.

[S8] G. M. Sheldrick, *Acta Cryst.* **2015**, *A71*, 3-8.

[S9] G. M. Sheldrick, *Acta Cryst.* **2015**, *C71*, 3-8.
